# Regiodivergent Interrupted Ni-Catalyzed Chain-Walking of Unsaturated Alcohol Side-Chains via Traceless Activating Groups

**DOI:** 10.1021/jacs.5c09426

**Published:** 2025-07-30

**Authors:** Hao Wang, Huihui Zhang, Marta Martínez-Belmonte, Jordi Benet-Buchholz, Ruben Martin

**Affiliations:** † Institute of Chemical Research of Catalonia (ICIQ), The Barcelona Institute of Science and Technology, Av. Països Catalans 16, 43007 Tarragona, Spain; ¶ 202569 Universitat Rovira i Virgili, Departament de Química Orgánica, c/Marcel·lí Domingo 1, 43007 Tarragona, Spain; ∥ ICREA, Passeig Lluís Companys, 23, 08010 Barcelona, Spain

## Abstract

Herein, we describe a regiodivergent interrupted chain-walking of unsaturated aliphatic alcohols. The method leverages the potential of traceless groups to forge C­(sp^3^)–C­(sp^3^) and C­(sp^3^)–nitrogen bonds at distal methylene sp^3^ C–H sites, with site selectivity dictated by a judicious choice of the ligand. The protocol is distinguished by its broad applicability, offering unconventional disconnections to access β/γ-substituted aliphatic alcohols that are difficult to reach otherwise.

## Graphical abstract

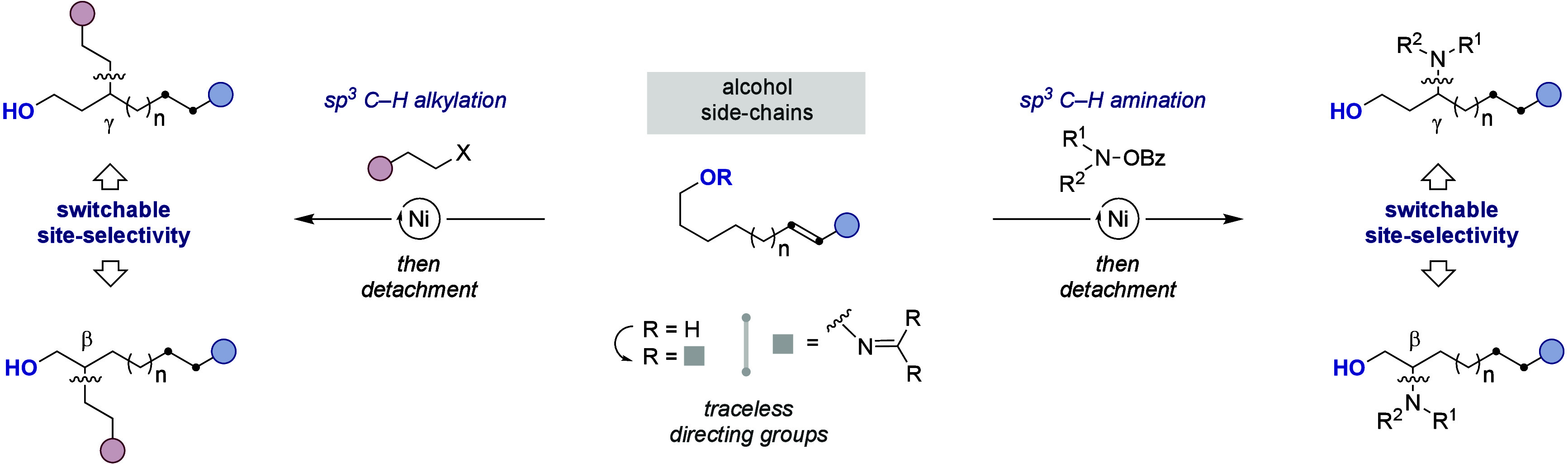

Driven by the prevalence of aliphatic alcohols in a myriad of pharmaceuticals,^1,2^ chemists have been challenged to design catalytic techniques that leverage the potential of aliphatic alcohols as linchpins for further elaboration. Unlike deoxygenative events via sp^3^ C–O scission aided by preactivation strategies (Scheme 1, *path a*),^3^ catalytic sp^3^ C–H functionalization techniques offer the advantage of retaining the alcohol function, thus representing a valuable bonus from both a conceptual and synthetic standpoint.^4–7^ While light-induced processes have shown the viability for enabling α/δ-functionalization via hydrogen-atom transfer or ligand-to-metal charge transfer (*path b*),^8,9^ a regiodivergent blueprint capable of dictating the site-selective incorporation of carbon or heteroatom fragments at either β- or γ- sp^3^ C–H sites with earth abundant metal catalysts still constitutes an uncharted cartography in the cross-coupling arena (*path c*).^10^

**Scheme 1:**
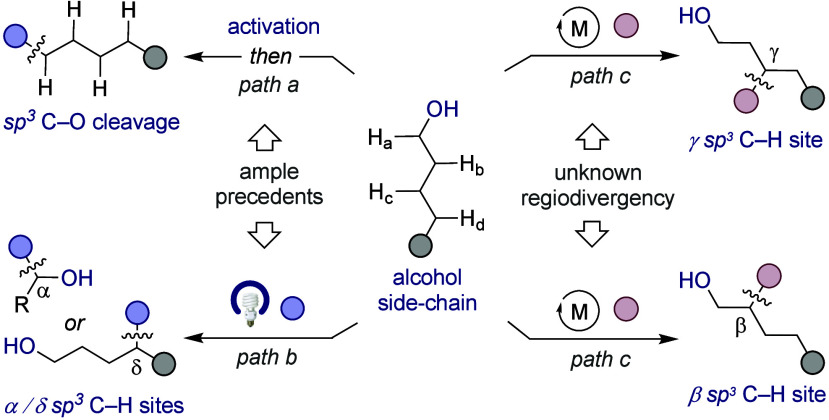
Aliphatic Alcohols in Cross-Coupling Reactions

On the basis of recent investigations into Ni-catalyzed interrupted chain-walking by our group^11^ and others,^12^ we wondered whether it would be possible to design a catalytic strategy that enables bond formation at β- and γ- sp^3^ C–H sites of unsaturated aliphatic alcohols. If successful, such a scenario could offer an unconventional new entry point to functionalize alcohol side-chains that would be difficult to reach otherwise. Given that the high polarizability of the O–H bond^13^ and the presence of adjacent hydrogen atoms might compromise both reactivity and site-selectivity,^8,9^ we recognized that it would be necessary to conceive an alcohol activation strategy prior to cross-coupling. Such activation should (a) be readily installed and detached at later stages with high chemoselectivity and (b) be amenable to predict the selectivity pattern. We envisioned that this could be fulfilled by incorporating oximes as traceless groups,^14,15^ with site-selectivity being dictated by preferential formation of five- or six-membered nickelacycles (**I** and **II**) prior to reaction with an electrophilic partner (Scheme 2).^16^ Herein, we report the successful realization of this goal, culminating in a widely applicable interrupted chain-walking protocol that incorporates amine or aliphatic carbon architectures at β or γ sp^3^ C–H sites of alcohol side-chains with an exquisite site-selectivity pattern.

**Scheme 2:**
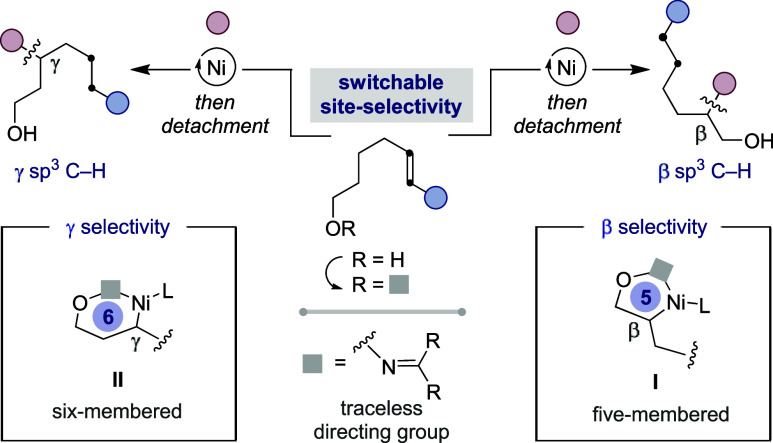
Interrupted Chain-Walking in Unsaturated Alcohol Side-Chains^a^

a TDG = Traceless Directing Group.

We began our investigations by studying the Ni-catalyzed interrupted chain-walking of **1a**—readily prepared on a gram scale in a two-step, one-pot process from but-3-en-1-ol^17^—with **2a** and **3a** (Table 1). After systematic evaluation of all reaction parameters,^18^ a protocol consisting of NiI_2_ (10 mol %), **L1** (20 mol %), (MeO)_3_SiH (2 equiv), and KF (2 equiv) in MeCN (0.1 M) at 40 °C delivered **4a** in 87% yield with exquisite β-selectivity (entry 1). In sharp contrast, a Ni/**L6** regime was particularly suited for obtaining **5a** in 80% isolated yield and excellent β-selectivity (entry 12). As anticipated, the nature of the ligand was critical for success. Specifically, the tetrahydroquinoline backbone in **L1** showed superior selectivities and yields compared to pyrox-type ligands bearing either pyridine (**L3**, **L4**) or quinoline fragments (**L2**) en route to **4a**, whereas substituents adjacent to the nitrogen atom in the 1,10-phenanthroline series (**L5**–**L8**) outperformed otherwise related 2,2′-bipyridines when obtaining **5a**. Notably, the utilization of solvents, nickel precatalysts, silanes, and bases other than MeCN, NiI_2_, (MeO)_3_SiH, and KF had a deleterious effect en route to **4a** (entries 7–10). The same holds true when promoting the reaction en route to **5a** (entries 13–16), thus revealing the subtle interplay that other reaction parameters have on both the reactivity and site-selectivity. Prompted by the low site-selectivity exerted by **L3** (entry 3), we wondered whether further optimization might lead to a γ-selective protocol for obtaining **6a** instead. Gratifyingly, this was the case, and the combination of NiBr_2_·DME (10 mol %) and **L3** (20 mol %) in DMF:DME (1:1) at rt resulted in a selectivity switch, obtaining **6a** in 89% isolated yield with excellent γ-selectivity (entry 11). Importantly, a similar switch could be implemented for obtaining a γ sp^3^ C–H amination of alcohol side-chains, with a Ni/**L11** regime providing the best results that led to 90% isolated yield of **7a** and excellent 11:1 γ-selectivity (entry 22). In view of these results, we believe site-selectivity is enabled by subtle stereoelectronic effects at the ligand that result in the formation of five- or six-membered nickelacycles (Scheme 2, **I** and **II**). Specifically, β-selectivity is favored by sterically encumbered ligands (**L1**) or rigid backbones bearing two electron-donating motifs (**L6**) via **I**, whereas less-hindered pyrox-type ligands (**L3**) or 2,2′-bipyridine motifs bearing a single electron-donating group at C4 (**L11**) might facilitate γ-selectivity via the intermediacy of more flexible six-membered nickelacycles (**II**).

**Table 1:**
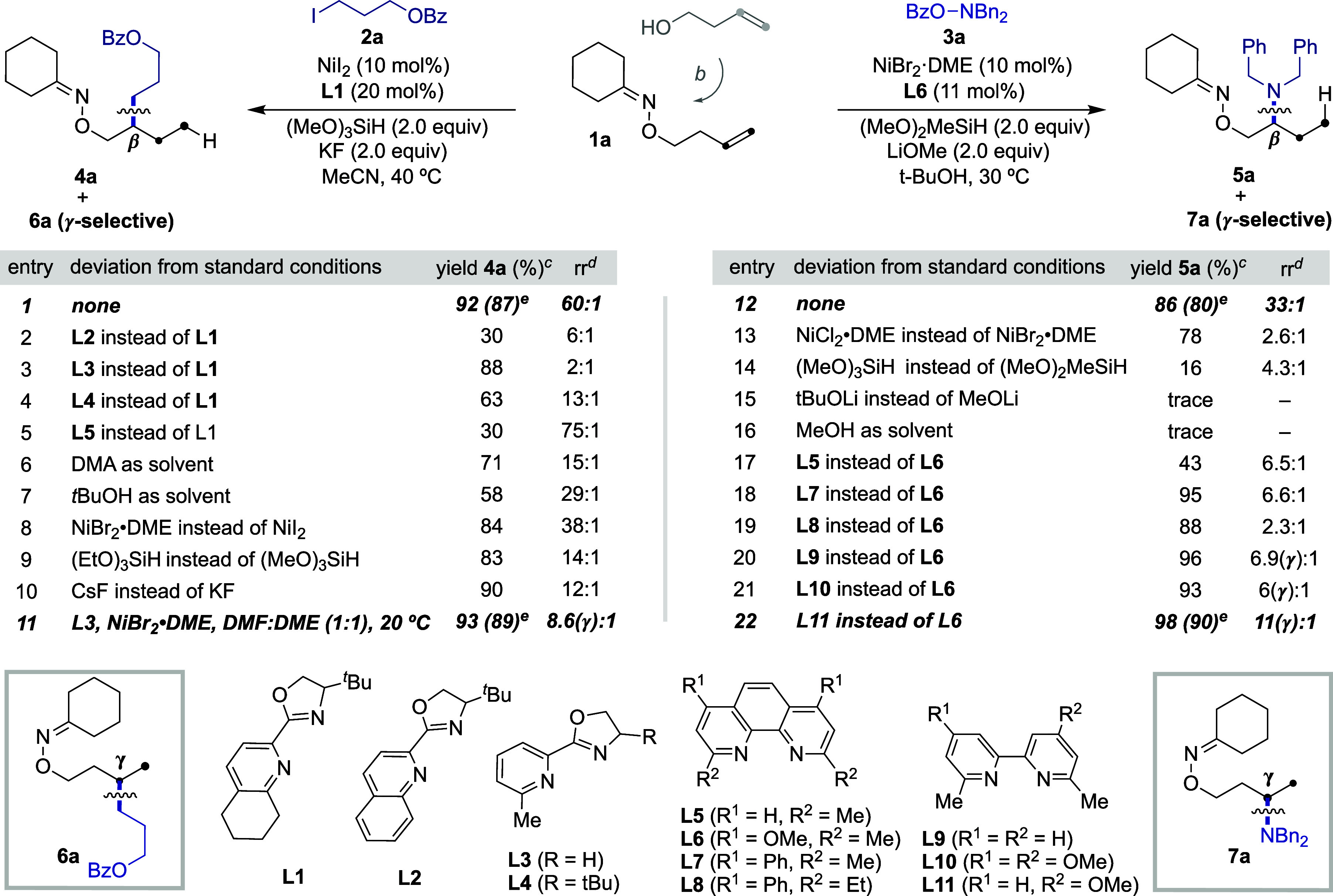
Optimization of the Reaction Conditions^a^

a Conditions A (β-alkylation): **1a** (0.15 mmol), **2a** (0.10 mmol), NiI_2_ (10 mol %), **L1** (20 mol %), (MeO)_3_SiH (0.20 mmol), KF (0.20 mmol) in MeCN (1.0 mL), 40 °C, 24 h. Conditions B (β-amination): **1a** (0.10 mmol), **3a** (0.15 mmol), NiBr_2_·DME (10 mol %), **L6** (11 mol %), (MeO)_2_MeSiH (0.20 mmol), LiOMe (0.20 mmol) in *t*BuOH (1.0 mL), 30 °C, 36 h.

b 3-Butenol (10 mmol), NHPI (11 mmol), Ph_3_P (11 mmol), DIAD (11 mmol) in THF for 6 h, then hydrazine (12 mmol) and cyclohexanone (12 mmol), 12 h, rt.

c GC yields using dodecane as internal standard.

d rr refers to the ratio of the major regioisomer to all other possible regioisomers.

e Isolated yield, average of two independent runs. NHPI = 2-hydroxyisoindoline-1,3-dione. DIAD = diisopropyl diazene-1,2-dicarboxylate.

With optimized conditions in hand, we turned our attention to studying the generality of the regiodivergent β C­(sp^*3*^)–H alkylation and β C­(sp^*3*^)–H amination of unsaturated alcohol derivatives (Table 2). As shown, the protocol was found to be applicable to a wide variety of substrates. In particular, high yields and selectivities for both β-alkylation and β-amination were found regardless of the traceless oxime directing group utilized, thus reinforcing the notion that β selectivity is dictated by the ligand backbone. The β-selectivity was unequivocally confirmed by X-ray crystallography of **5a** and **5c**.^18^ Long-range interrupted chain-walking scenarios are within reach with exclusive β-selectivity (**3a** and **5a** vs **3l**–**p** and **5k**–**5l**). A simple comparison of both protocols, however, indicates that yields are slightly attenuated at long range, particularly when conducting β C­(sp^3^)–H amination (**5a** vs **5k**–**l**). Particularly noteworthy was the observation that the interrupted chain-walking could also be applied to internal olefins. It is worth noting that high yields were obtained regardless of whether *E*- or *Z*-internal olefins were utilized in the β C­(sp^3^)–H alkylation (**3m**), whereas only *E*-olefins could be employed in the β C­(sp^3^)–H amination (**5l**). Even trisubstituted or 1,1-disubstituted olefins could be employed as substrates for the targeted C­(sp^3^)–C­(sp^3^) or C­(sp^3^)–N bond formations, albeit in lower yields (**3q**–**3r** and **5m**–**5n**). Equally interesting was the ability to extend these conceptions to unsaturated alcohols possessing substituents adjacent to the alcohol function, leading to the corresponding β isomers with exquisite site-selectivity (**3s**–**3t** and **5o**–**5q**). However, statistical mixtures of diastereoisomers were obtained when attempting the reaction with unactivated alkyl iodides (**3s**–**3u**), whereas a high diastereoselectivity was observed for β C­(sp^3^)–H amination (**5o**–**5q**), thus showing the intricacies of the protocol.^18^ Desymmetrization could be accomplished when utilizing hepta-1,6-dien-4-ol as the substrate en route to **5q** with high diastereomeric ratios, thus leaving ample room for further derivatization of the remaining olefin backbone. The successful preparation of **3v**–**3ao** and **5s**–**5ai** further highlights the generality for incorporating primary and secondary alkyl fragments with equal ease at the β sp^3^ C–H site of unsaturated alcohols. Moreover, both β-amination and β-alkylation protocols exhibited an excellent chemoselectivity profile, as esters (**3z**, **3ab**, **5x** and **5ag**), carbamates (**3ag** and **3aj**), nitriles (**3ac** and **5y**), ketones (**3ad**), sulfones (**5t**), and acetals (**5z**) were all well accommodated. Even alkyl chlorides could be tolerated (**3aa**), thus leaving ample room for further elaboration via cross-coupling.^19^ In addition, the size of the substituents at the amine function had a non-negligible impact on the reactivity. Specifically, piperidine analogues gave consistently high yields (**5ac**), whereas the inclusion of either pyrrolidine or azepane motifs had a deleterious effect on reactivity (**5ad**, **5ae**). The preparation of **5ah** containing a pyrimidine backbone is particularly noteworthy, suggesting that the presence of additional nitrogen donors might not compromise the reactivity or site-selectivity. The applicability is further illustrated in the preparation of derivatives from Phytol (**3u** and **5r**), Galactopyranose (**3ak**), Oxaprozin (**3al**), Isoxepac (**3am**), Tezacaftor intermediate (**3an**), Indometacin (**3ao**), and Paroxetine (**5ai**), among others.^20^

**Table 2:**
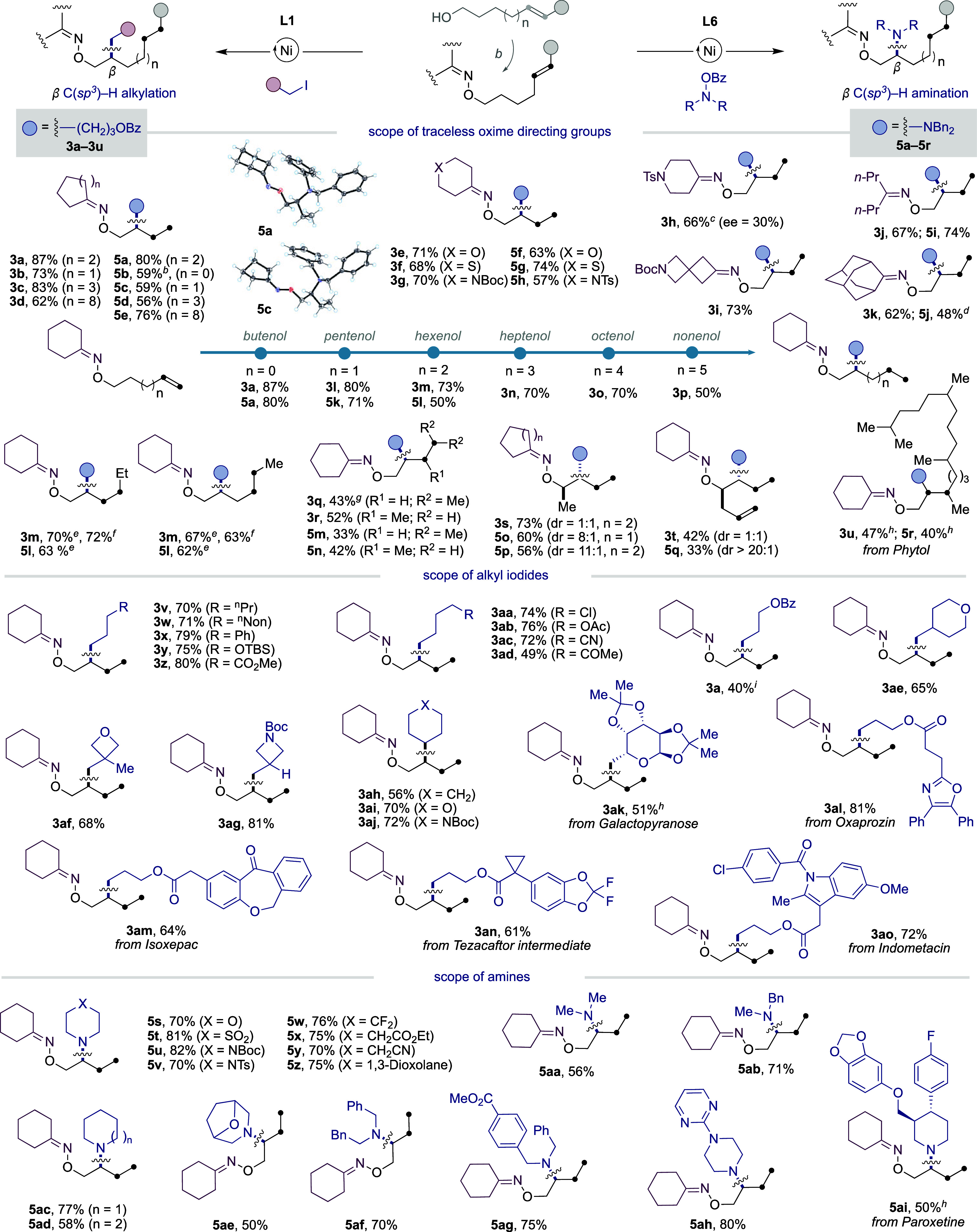
Ni-Catalyzed β-Alkylation and β-Amination of Unsaturated Alcohols via Interrupted Chain-Walking^a^

a As in Table 1 (entries 1 and 12). Yields of isolated compounds, average of at least two independent runs.

b Alcohol (1.0 equiv), NHPI (1.1 equiv), Ph_3_P (1.1 equiv), DIAD (1.1 equiv) in THF for 6 h, then hydrazine (1.2 equiv) and cyclohexanone (1.2 equiv), 12 h, rt.

c With (*S*)-**L1**, 30% ee.

d rr = 8:1.

e From *E*-isomer.

f From *Z*-isomer.

g rr = 9:1.

h dr = 1:1.

i Utilizing alkyl bromide as substrate. NHPI = 2-hydroxyisoindoline-1,3-dione. DIAD = diisopropyl diazene-1,2-dicarboxylate.

On the basis of the results shown in Table 1 (entries 11 and 22), we next evaluated the generality of a regiodivergent γ-alkylation/γ-amination of unsaturated alcohol side-chains. As shown in Table 3, the combination of 3-butenol, 4-hexenol, or 5-heptanol with a variety of unactivated alkyl iodides and electrophilic amine partners delivered the targeted products in good yields and regioselectivities (**6a**–**6g**, **7a**–**7d**, **6h**, **7f**–**7i**). Even α-branched alcohols or substrates bearing internal olefins could be utilized as substrates (**7e** and **7i**). Notably, high diastereoselectivities were observed in the former, whereas significant γ-selectivity was found in the latter. Although γ-selectivities were generally modest when compared to the exquisite β-functionalization found in Table 2, these results should be interpreted against the challenge that is addressed, offering not only a platform for controlling the motion at which Ni catalysts enable C­(sp^3^)–C­(sp^3^) and C­(sp^3^)–N bonds via chain-walking reactions of unsaturated alcohols enabled by traceless directing groups but also a complementary technique to existing hydroalkylation or hydroamination reactions of olefin-containing precursors.^16^

**Table 3:**
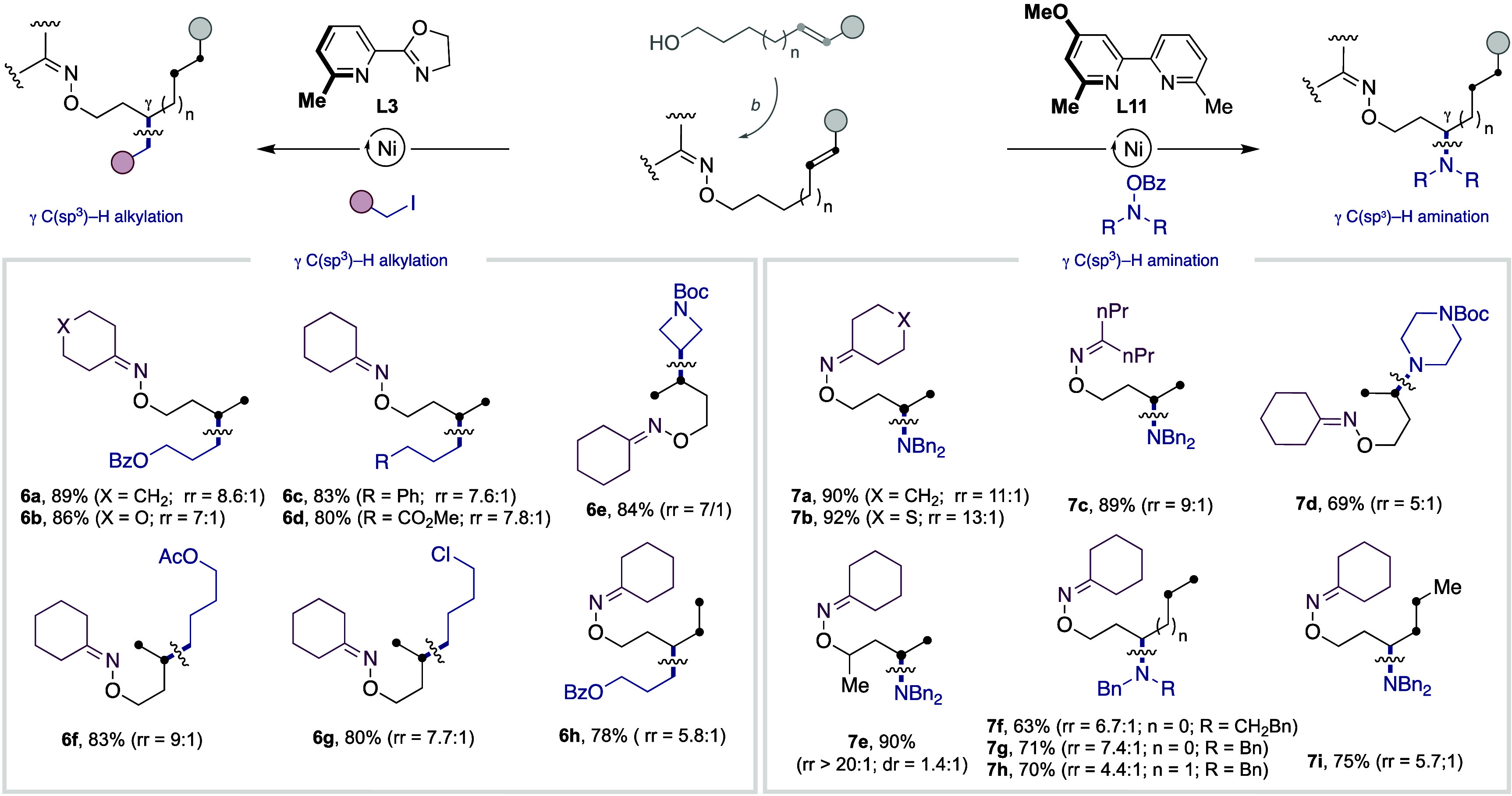
Ni-Catalyzed γ-Alkylation and γ-Amination of Unsaturated Alcohols via Interrupted Chain-Walking^a^

a As in Table 1 (entries 11 and 22). Yields of isolated compounds, average of at least two independent runs.

b Alcohol (1.0 equiv), NHPI (1.1 equiv), Ph_3_P (1.1 equiv), DIAD (1.1 equiv) in THF for 6 h, then hydrazine (1.2 equiv) and cyclohexanone (1.2 equiv), 12 h, rt. NHPI = 2-hydroxyisoindoline-1,3-dione. DIAD = diisopropyl diazene-1,2-dicarboxylate.

The synthetic potential is further illustrated in Scheme 3. Specifically, we found that the traceless oxime directing group is detached from the alkyl side chain either by simple exposure to Pd/C under H_2_ (**8a**–**8g** and **8k**)^5k^ or by reaction with an appropriate hydride source (**8h**–**8j**).^6a,6b^ X-ray diffraction of **8j** and **8i′** unambiguously confirmed both the γ-selectivity observed for a Ni/**L11** regime and the antistereochemistry shown for **8i**.^18^ While one might argue that β-selectivity might arise from an olefin isomerization en route to vinyl ether intermediates^21^ followed by reaction with an unactivated alkyl halide or an electrophilic amine source, not even traces of vinyl ethers were detected by monitoring the reaction of **1a** and **2a** or **1a** and **4a** by ^1^H NMR spectroscopy.^22^ This notion gains credence by the lack of deuterium incorporation at the β-position in **3a-** *d*_2_ and **5a-** *d*_2_ when utilizing **1a-** *d*_2_ as the substrate, thus reinforcing the notion that β-selectivity arises from five-membered nickelacycles, whereas a six-membered nickelacycle might be responsible for γ-selectivity instead (Scheme 3, *bottom left*).^16^ On the other hand, the intermediacy of open-shell species was indirectly assessed by exposure of **2ap** to **1a** under our optimized β-alkylation conditions. Under the limits of detection, traces of **3aq** were obtained in the crude reaction mixtures, obtaining **3ap** in 43% yield (Scheme 3, *bottom right*).

**Scheme 3:**
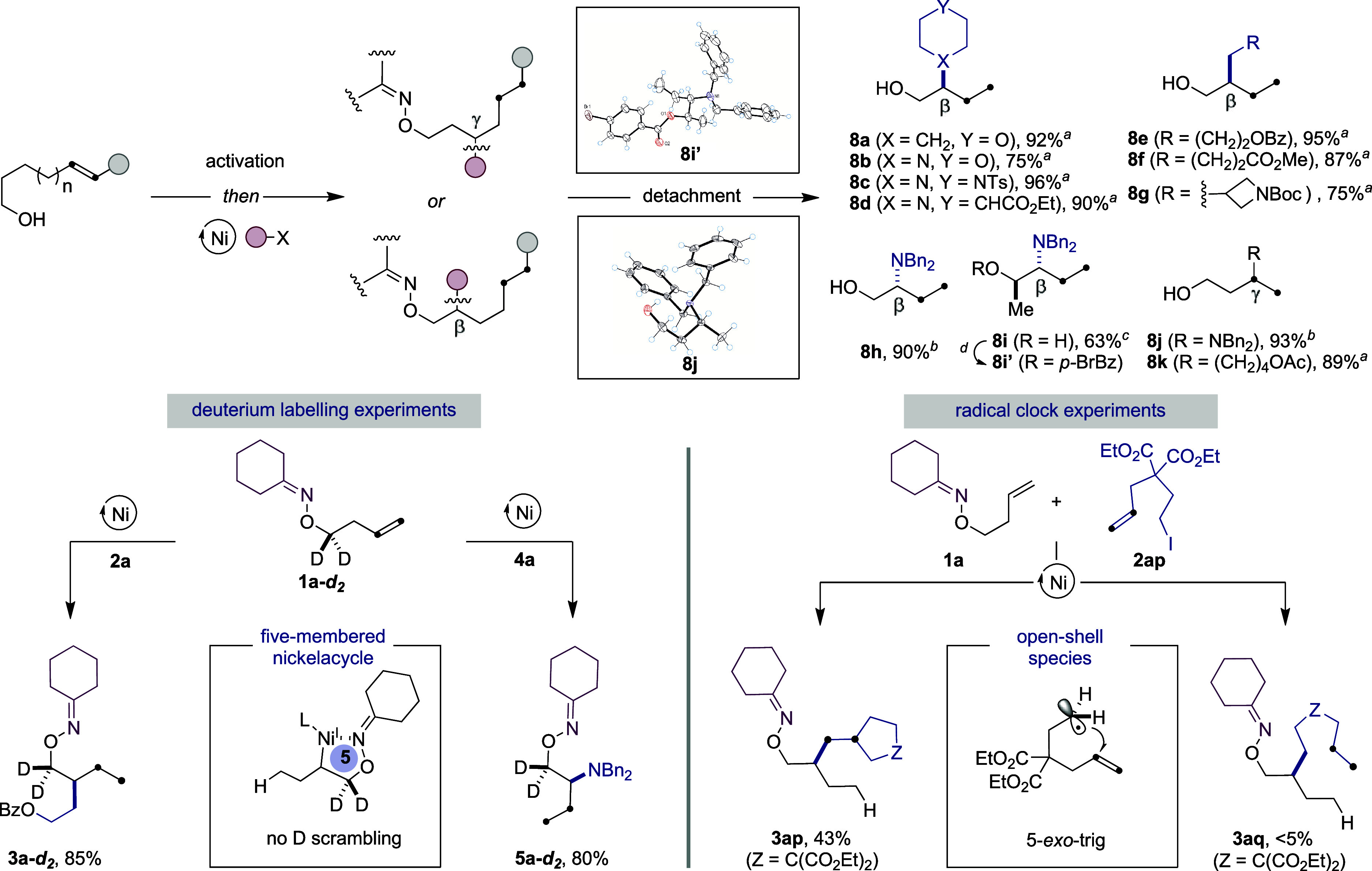
Detachment of Directing Group and Preliminary Mechanistic Studies

a Conditions: oxime ether (0.20 mmol, 1.0 equiv), Pd/C (5% Pd on activated carbon, 80 mg), H_2_ balloon, and MeOH (2.0 mL) at rt for 24 h.
b Oxime ether (0.20 mmol, 1.0 equiv), LiAlH_4_ (5.0 equiv) in Et_2_O (2.0 mL) at rt for 48 h.
c Oxime ether (0.20 mmol, 1.0 equiv), NaBH_4_ (5.0 equiv) and I_2_ (2.0 equiv) in THF (2.0 mL), reflux for 16 h.
d
**8i** (0.10 mmol, 1.0 equiv), 4-bromobenzoyl chloride (0.11 mmol, 1.1 equiv), DMAP (0.01 mmol, 0.1 equiv), and DCC (0.12 mmol, 1.2 equiv) in CH_2_Cl_2_ (2.0 mL), 0 °C to rt, 72 h.

In summary, we have developed a switchable yet predictable interrupted chain-walking scenario of unsaturated alcohol side-chains with oximes as traceless directing groups. Site-selectivity is dictated by a judicious choice of the ligand backbone, offering a new entry point for incorporating sp^3^ architectures at distal, yet previously unfunctionalized, sp^3^ C–H reaction sites. The broad applicability and versatility of the protocol not only expand our repertoire in chain-walking reactions but also offer unconventional retrosynthetic disconnections to access compounds of interest in medicinal chemistry.

## Supplementary Material



## Notes

Experimental procedures and spectral and crystallographic data (PDF)
