# Supplementary material for: Regiodivergent Interrupted Ni-Catalyzed Chain-Walking of Unsaturated Alcohol Side-Chains via Traceless Activating Groups
Source: J Am Chem Soc. 2025 Jul 30;147(32):28610–6. doi: 10.1021/jacs.5c09426 (PMC12439304; doi:10.1021/jacs.5c09426)
Supplement: Supplementary file 1 [file ja5c09426_si_001.pdf]

Supporting Information

Regiodivergent Interrupted Ni-Catalyzed Chain-Walking of Unsaturated Alcohol Side-Chains via Traceless Activating Groups

Hao Wang,[†¶§] Huihui Zhang,[†¶§] Marta Martínez-Belmonte,[†] Jordi Benet-Buchholz,[†] and Ruben Martin*[†£]

[†] Institute of Chemical Research of Catalonia (ICIQ), The Barcelona Institute of Science and Technology, Av. Països Catalans 16, 43007 Tarragona, Spain

[¶] Universitat Rovira i Virgili, Departament de Química Orgànica, c/Marcel·lí Domingo 1, 43007 Tarragona, Spain.

[£] ICREA, Passeig Lluís Companys, 23, 08010 Barcelona, Spain

[§] These authors contributed equally to this work

E-mail: rmartinromo@iciq.es

Contents

1. *General Information* 2
2. *Reaction optimization* 3
3. *Synthesis of Starting Materials* 6
4. *Ni-catalyzed interrupted chain-walking of unsaturated aliphatic alcohols* 19
5. *Unsuccessful or low yielding substrates* 60
6. *Deprotection of the traceless directing group* 61
7. *Crystallographic Data* 67
8. *NMR Spectra* 73

1. General Information

Reagents: Commercially available materials were used as received without further purification. Nickel(II) iodide (NiI_2) was purchased from Strem or Thermo Scientific. Nickel(II) bromide ethylene glycol dimethyl ether complex ($NiBr_2$·DME) was purchased from Sigma-Aldrich. Potassium fluoride (KF, anhydrous) was purchased from Thermo Scientific. Lithium methoxide (LiOMe) was purchased from Sigma-Aldrich. NiI_2, $NiBr_2$·DME, KF and LiOMe were stored in the glovebox. Trimethoxysilane [$(MeO)_3SiH$] was purchased from Thermo Scientific. Dimethoxymethylsilane [$(MeO)_2MeSiH$] was purchased from TCI. Acetonitrile (ACN, anhydrous, 99.9+%) and Dimethylformamide (DMF, anhydrous, 99.5%) were purchased from Thermo Scientific. 1,2-Dimethoxyethane (DME, anhydrous, 99.5%) and *tert*-Butyl alcohol (*t*BuOH, anhydrous, ≥99.5%) were purchased from Sigma-Aldrich.

Analytical methods: ^1H and ^{13}C spectra were recorded on Bruker 400 MHz and Bruker 500 MHz at 20°C. All ^1H and ^{13}C NMR spectra are reported in parts per million (ppm) downfield of TMS and were calibrated using the corresponding residual solvent peak. Coupling constants, *J*, are reported in Hertz. DEPT135 and 2-dimensional experiments (COSY, HMBC, HSQC and NOESY) were used to support assignments when appropriate but were not included herein. Gas chromatography was performed on Agilent 7890A with FID detector and a fused silica column HP–5 (19091J–413; 30m x 0.32mm x 0.25μm), analysis method: (A). 1.9 mL/min flow, 9.9276 psi, 70 °C for 1 min, 70–220 °C, 20 °C/min for 2 min; 220–300 °C, 35 °C/min for 2 min; (B). 1.9 mL/min flow, 9.9276 psi, 70 °C for 1 min, 70–220 °C, 35 °C/min for 2min; 220–300 °C, 35 °C/min for 6 min; dodecane as internal standard. Flash chromatography was performed with Sigma-Aldrich silica gel, pore size 60 Å (230-400 mesh). TLC silica gel F_{254} aluminium sheets from Sigma-Aldrich were used with visualization through UV irradiation and/or staining with basic $KMnO_4$ solution. The isolated yields reported represent an average of two independent runs.

2. Reaction optimization

2.1 Optimization of the β-alkylation of aliphatic alcohols decorated with oximes

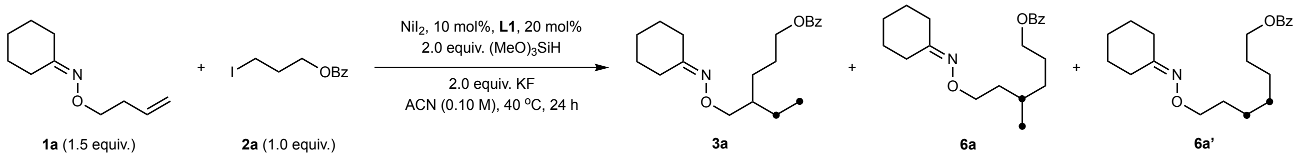

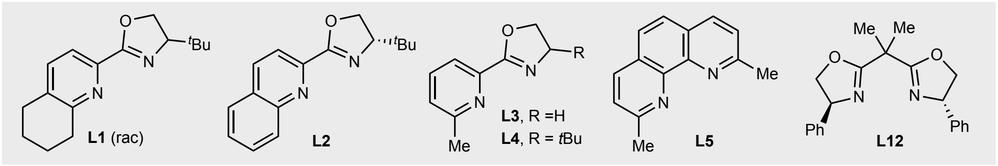

entry	deviation from standard conditions	yield (%) **(3a+6a+6a')**	ratio **3a/(6a+6a')**
1	none	**92 (87)**	60/1
2	$NiCl_2$ instead of NiI_2	85	19/1
3	$NiBr_2$·DME instead of NiI_2	84	38/1
4	**L2** instead of **L1**	30	6/1
5	**L3** instead of **L1**	88	2/1
6	**L4** instead of **L1**	63	13/1
7	**L5** instead of **L1**	30	75/1
8	**L12** instead of **L1**	60	7/1
9	DMF as solvent	71	4/1
10	DMA as solvent	71	15/1
11	*t*BuOH as solvent	58	29/1
12	$(EtO)_3MeSiH$ instead of $(MeO)_3SiH$	83	14/1
13	$(MeO)_2MeSiH$ instead of $(MeO)_3SiH$	78	20/1
14	CsF instead of KF	90	12/1

Conditions: **1a** (1.2 equiv.), **2a** (0.1 mmol, 1.0 equiv.), Ni cat. (10 mol%), ligand (20 mol%), R_3SiH (2.0 equiv.), KF (2.0 equiv.) and solvent (0.1 M) at 40 °C for 24 h. Yields and regioisomers determined by GC-FID using dodecane as internal standard. (DMA = dimethylacetamide, DMF = dimethylformamide. ACN = acetonitrile.)

2.2 Optimization of the γ-alkylation of aliphatic alcohols decorated with oximes

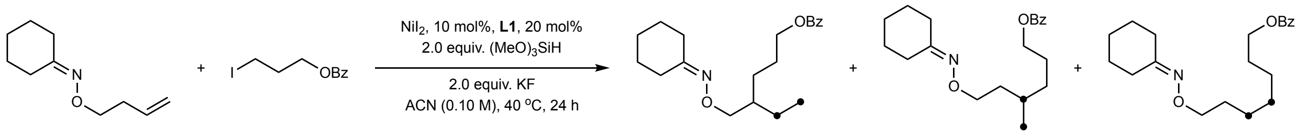

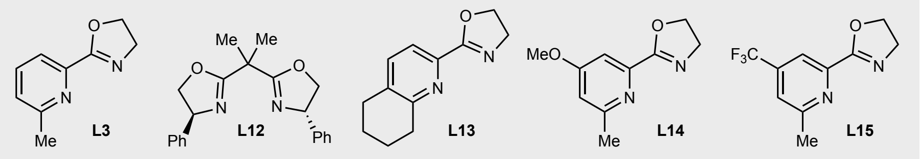

entry	deviation from standard conditions	yield (%) **(3a+6a+6a')**	ratio **6a/(3a+6a')**
1	DMA as solvent	89	1.6/1
2	DMF as solvent	93	4.1/1
3	DME as solvent	34	0.3/1
4	DMF as solvent, **L12** instead of **L3**	52	1.8/1
5	DMF as solvent, **L13** instead of **L3**	84	3.7/1
6	DMF as solvent, **L14** instead of **L3**	87	3.9/1
7	DMF as solvent, **L15** instead of **L3**	20	4.8/1
8	DMF/DME = 1/1	91	3.0/1
**9**	**DMF/DME = 1/1 at 20 °C**	**93(89)**	**8.6/1**
10	DMF/DME = 1/1 at 10 °C	75	2.2/1
11	DMF/DME = 7/3 at 20 °C	30	2.5/1
12	DMF/DME = 3/7 at 20 °C	98	4.3/1
13	DMF/DME = 1/1, 20 °C; $NiCl_2$ instead of $NiBr_2$•DME	89	4.7/1
14	DMF/DME = 1/1, 20 °C; NiI_2 instead of $NiBr_2$•DME	90	2.2/1
15	DMF/DME = 1/1, 20 °C; $(MeO)_2MeSiH$ instead of $(MeO)_3SiH$	88	6.4/1

Conditions: **1a** (1.2 equiv.), **2a** (0.1 mmol, 1.0 equiv.), Ni cat. (10 mol%), ligand (20 mol%), R_3SiH (2.0 equiv.), KF (2.0 equiv.) and solvent (0.1 M) at 40 °C for 16 h. Yields and regioisomers determined by GC-FID using dodecane as internal standard. (DMA = dimethylacetamide, DMF = dimethylformamide, DME = dimethoxyethane.)

2.3 Optimization of the β- & γ-amination of aliphatic alcohols decorated with oximes

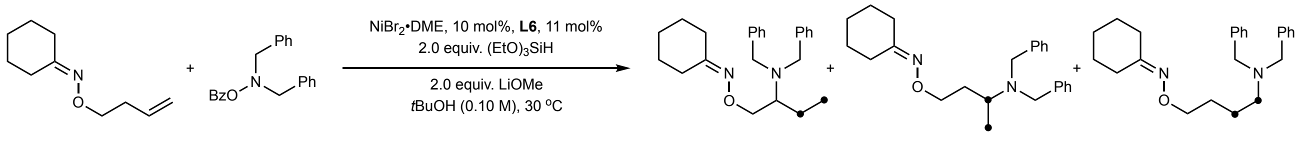

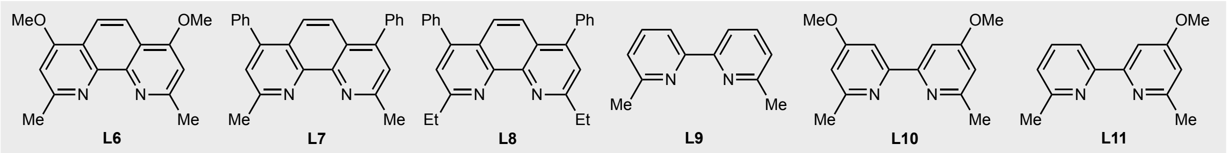

entry	deviation from standard conditions	yield (%) (5a+7a+7a')	r.r.[a]
1	none	76	20.7(β)/1
2	$NiBr_2$•diglyme instead of $NiBr_2$•DME	93	10.9(β)/1
3	$NiCl_2$•DME instead of $NiBr_2$•DME	78	2.6(β)/1
4	$(MeO)_3SiH$ instead of $(EtO)_3SiH$	16	4.3(β)/1
**5**	**$(MeO)_2MeSiH$** instead of **$(EtO)_3SiH$**	**86**	**33.4(β)/1**
6	$(EtO)_2MeSiH$ instead of $(EtO)_3SiH$	74	22(β)/1
7	$(MeO)_2MeSiH$, **L5** instead of **L6**	43	6.5(β)/1
8	$(MeO)_2MeSiH$, **L7** instead of **L6**	95	6.6(β)/1
9	$(MeO)_2MeSiH$, **L8** instead of **L6**	88	2.3(β)/1
10	$(MeO)_2MeSiH$, **L9** instead of **L6**	96	6.9(γ)/1
11	$(MeO)_2MeSiH$, **L10** instead of **L6**	93	6.0(γ)/1
**12**	**$(MeO)_2MeSiH$, L11** instead of **L6**	**98**	**11.1(γ)/1**
13	$(MeO)_2MeSiH$, dioxane as solvent	14	4.5(β)/1
14	$(MeO)_2MeSiH$, toluene as solvent	8	1.7(β)/1
15	$(MeO)_2MeSiH$, *t*AmylOH as solvent	74	18.1(β)/1
16	$(MeO)_2MeSiH$, MeONa instead of MeOLi	-	-
17	$(MeO)_2MeSiH$, *t*BuOLi instead of MeOLi	-	-
18	$(MeO)_2MeSiH$ at 40 °C	82	20.1(β)/1

Conditions: **1a** (0.1 mmol, 1.0 equiv.), **4a** (1.5 equiv.), Ni cat. (10 mol%), ligand (11 mol%), R_3SiH (2.0 equiv.), MeOLi (2.0 equiv.) and solvent (0.1 M) at 30 °C for 36 h. Yields and regioisomers determined by GC-FID using dodecane as internal standard. (*t*AmylOH = *tert*-Amyl alcohol.). [a] r.r. refers to the ratio of the major regioisomer to all other regioisomers.

3. Synthesis of Starting Materials

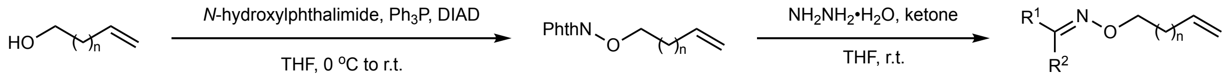

General procedure A.[1,2] To a dry round-bottom flask were charged a stir bar, the alcohol substrate (1.0 equiv.), *N*-hydroxyphthalimide (1.1 equiv.), PPh_3 (1.1 equiv.) and THF (0.5 M). After the reaction mixture was cooled to 0 °C (ice bath), DIAD (1.1 equiv.) was added dropwise. The resulting mixture was allowed to stir at the room temperature for 3 hours followed by addition of hydrazine monohydrate ($NH_2NH_2 \cdot H_2O$, 1.2 equiv.). After being stirred for another 30 minutes, ketone (1.2 equiv.) was added to the reaction mixture, which was then allowed to stir overnight. The reaction was quenched by the addition of excess ketone, followed by filtration through celite. The filtrate was then concentrated *in vacuo* and purified by flash column chromatography on silica gel to afford the oxime ether.

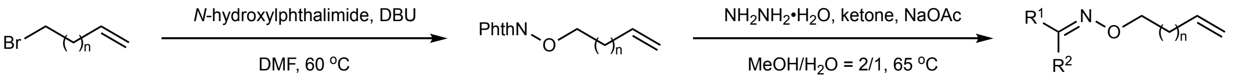

General procedure B.[1] To a stirred solution of *N*-hydroxyphthalimide (1.1 equiv.) and alkyl bromide (1.0 equiv.) in DMF (1.0 M) was added dropwise DBU (1.1 equiv.) at room temperature. After stirring for 2 h at 60 °C, the reaction mixture was poured into cold 1 N HCl solution and extracted by EtOAc. The organic layered was washed with water and brine, followed by drying over Na_2SO_4 and concentration *in vacuo* afforded the corresponding crude *N*-alkoxyphthalimides. To a solution of above *N*-alkoxyphthalimide (1.0 equiv.) in MeOH (0.5 M) was added hydrazine monohydrate ($NH_2NH_2 \cdot H_2O$, 1.0 equiv.) at room temperature slowly. After stirred for 30 minutes, ketone (3.0 equiv.), sodium acetate (5.0 equiv.) and water (half volume as MeOH) were added to the reaction mixture. The resulting mixture was heated at 65 °C for 5 h. The mixture was cooled to room temperature, followed by drying over Na_2SO_4, filtered, and concentrated *in vacuo*. The residue was purified by column chromatography on silica del to give the corresponding oxime ether.

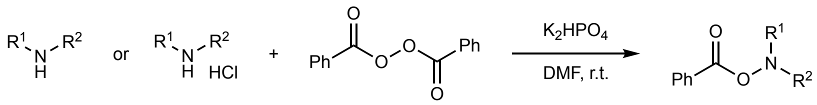

General procedure C.[3] To a solution of the alkyl amine or its hydrochloride salt (1.0 equiv.) and K_2HPO_4 (2.0 equiv.) in DMF (0.2 M) was added benzoyl peroxide (1.2 equiv.) at room temperature. After stirring for 18 hours, the mixture was diluted with H_2O and extracted with EtOAc. The combined organic layers were washed with brine and aqueous NH_4Cl, dried over Na_2SO_4, filtered, and evaporated. The residue was purified by column chromatography on silica del to give the corresponding amination precursors.

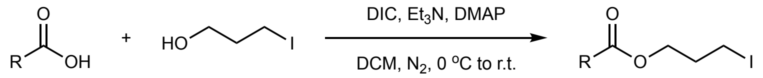

General procedure D. To a solution of the acid (1.0 equiv.) in anhydrous DCM at 0 °C under N_2 atmosphere was added *N,N'*-diisopropylcarbodiimide (DIC, 1.1 equiv.) and DMAP (0.2 equiv.). After 30 minutes, 3-iodo-1-propanol (1.1 equiv.) and Et_3N (1.1 equiv.) were added subsequently to the mixture. The resulting reaction mixture was allowed to warm up to room temperature and stirring overnight. The solution was diluted with DCM and filtered a plug of silica gel. The clear filtrate was collected, evaporated under reduced pressure and the crude product was purified by column chromatography on silica gel to obtain the desired product.

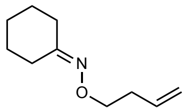

cyclohexanone *O*-but-3-en-1-yl oxime (1a). General procedure A was applied to cyclohexanone and but-3-en-1-ol (30.0 mmol) to afford **1a** by column chromatography as a colorless oil (4.01 g, 80%). **^{1}H NMR** (400 MHz, $CDCl_3$) δ 5.83 (ddt, *J* = 17.1, 10.2, 6.8 Hz, 1H), 5.12 – 5.05 (m, 1H), 5.03 (ddt, *J* = 10.23, 2.2, 1.2 Hz, 1H), 4.04 (t, *J* = 6.8 Hz, 2H), 2.47 – 2.43 (m, 2H), 2.40 (qt, *J* = 6.8, 1.4 Hz, 2H), 2.22 – 2.15 (m, 2H), 1.70 – 1.63 (m, 2H), 1.62 – 1.55 (m, 4H) ppm. **^{13}C NMR** (101 MHz, $CDCl_3$) δ 160.5, 135.3, 116.5, 72.4, 33.8, 32.4, 27.2, 26.0, 25.9, 25.5 ppm. **IR** (neat, cm^{-1}): 3078, 2929, 2859, 1641, 1449, 1435, 1049, 989, 939, 913, 872, 839. **HRMS** (ESI+) *calcd.* for ($C_{10}H_{18}NO$) $[M+H]^+$: 168.1383; *found* 168.1381.

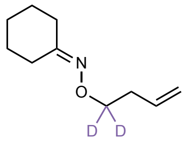

cyclohexanone *O*-but-3-en-1-yl-1,1-*d*$_2$ oxime (1a-*d*$_2$). General procedure A was applied to cyclohexanone and but-3-en-1,1-*d*$_2$-1-ol (5.0 mmol) to afford **1a-*d*$_2$** by column chromatography as a colorless oil (0.72 g, 85%). **^{1}H NMR** (400 MHz, $CDCl_3$) 5.83 (ddt, *J* = 17.1, 10.2, 6.8 Hz, 1H), 5.13 – 5.00 (m, 2H), 2.51 – 2.42 (m, 2H), 2.39 (d, *J* = 6.8 Hz, 2H), 2.23 – 2.16 (m, 2H), 1.73 – 1.62 (m, 2H), 1.61 – 1.55 (m, 4H) ppm. **^{13}C NMR** (101 MHz, $CDCl_3$) 160.4, 135.2, 116.5, 33.6, 32.4, 27.2, 26.0, 25.9, 25.4 ppm; **IR** (neat, cm^{-1}): 3078, 2978, 2930, 2858, 2209, 2099, 1641, 1448, 1436, 1128, 999, 966, 942, 911, 824. **HRMS** (ESI+) *calcd.* for ($C_{10}H_{16}D_2NO$) $[M+H]^+$: 170.1511; *found* 170.1508.

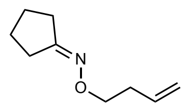

cyclopentanone *O*-but-3-en-1-yl oxime (1b). General procedure A was applied to cyclopentanone and but-3-en-1-ol (5.0 mmol) to afford **1b** by column chromatography as a colorless oil (0.64 g, 83%). **^{1}H NMR** (400 MHz, $CDCl_3$) δ 5.83 (ddt, *J* = 17.1, 10.2, 6.8 Hz, 1H), 5.12 – 5.05 (m, 1H), 5.03 (ddt, *J* = 10.2, 2.2, 1.2 Hz, 1H), 4.06 (t, *J* = 6.8 Hz, 2H), 2.4 – 2.3 (m, 6H), 1.8 – 1.7 (m, 4H) ppm. **^{13}C NMR** (101 MHz, $CDCl_3$) δ 166.6, 135.2,

116.5, 72.8, 34.0, 31.1, 27.8, 25.3, 24.8 ppm. **IR** (neat, cm^{-1}): 3077, 2961, 2871, 1641, 1430, 1371, 1212, 1048, 988, 958, 911, 877, 841. **HRMS** (ESI+) *calcd.* for ($C_9H_{16}NO$) $[M+H]^+$: 154.1226; *found* 154.1229.

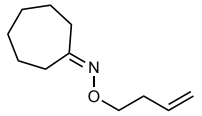

cycloheptanone *O*-but-3-en-1-yl oxime (1c). General procedure A was applied to cycloheptanone and but-3-en-1-ol (5.0 mmol) to afford **1c** by column chromatography as a colorless oil (0.78 g, 87%). **^{1}H NMR** (400 MHz, $CDCl_3$) δ 5.83 (ddt, *J* = 17.1, 10.2, 6.8 Hz, 1H), 5.13 – 5.04 (m, 1H), 5.03 (ddt, *J* = 10.2, 2.2, 1.2 Hz, 1H), 4.06 (t, *J* = 6.8 Hz, 2H), 2.54 – 2.46 (m, 2H), 2.44 – 2.29 (m, 4H) 1.68 – 1.59 (m, 4H), 1.61 – 1.49 (m, 4H) ppm. **^{13}C NMR** (101 MHz, $CDCl_3$) δ 164.0, 135.3, 116.5, 72.6, 34.0, 33.9, 30.6, 30.5, 29.3, 27.9, 24.8 ppm. **IR** (neat, cm^{-1}): 3077, 2923, 2853, 1641, 1454, 1443, 1045, 987, 937, 910. **HRMS** (ESI+) *calcd.* for ($C_{11}H_{20}NO$) $[M+H]^+$: 182.1539; *found* 182.1542.

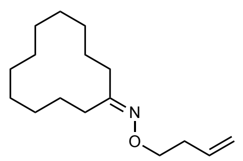

cyclododecanone *O*-but-3-en-1-yl oxime (1d). General procedure A was applied to cyclododecanone and but-3-en-1-ol (5.0 mmol) to afford **1d** by column chromatography as a colorless oil (0.87 g, 69%). **^{1}H NMR** (400 MHz, $CDCl_3$) δ 5.84 (ddt, *J* = 17.06, 10.24, 6.75 Hz, 1H), 5.12 – 5.05 (m, 1H), 5.03 (ddt, *J* = 10.3, 2.3, 1.3 Hz, 1H), 4.05 (t, *J* = 6.8 Hz, 2H), 2.40 (qt, *J* = 6.8, 1.5 Hz, 2H), 2.37 – 2.32 (m, 2H), 2.25 – 2.19 (m, 2H), 1.64 (p, *J* = 6.3 Hz, 2H), 1.55 (p, *J* = 6.8 Hz, 2H), 1.41 – 1.28 (m, 14H) ppm. **^{13}C NMR** (101 MHz, $CDCl_3$) δ 160.2, 135.5, 116.4, 72.5, 34.0, 31.1, 26.8, 25.4, 25.2, 24.9, 24.4, 23.9, 23.5, 23.5, 23.5, 23.1 ppm. **IR** (neat, cm^{-1}): 3077, 2927, 2863, 1641, 1469, 1443, 1369, 1050, 986, 911. **HRMS** (ESI+) *calcd.* for ($C_{16}H_{30}NO$) $[M+H]^+$: 252.2322; *found* 252.2323.

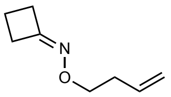

cyclobutanone *O*-but-3-en-1-yl oxime (1e). General procedure A was applied to cyclobutanone and but-3-en-1-ol (5.0 mmol) to afford **1e** by column chromatography as a colorless oil (0.37 g, 53%). **^{1}H NMR** (400 MHz, $CDCl_3$) δ 5.82 (ddt, *J* = 17.1, 10.3, 6.8 Hz, 1H), 5.14 – 5.06 (m, 1H), 5.03 (ddt, *J* = 10.2, 2.2, 1.2 Hz, 1H), 4.03 (t, *J* = 6.9 Hz, 2H), 2.96 – 2.78 (m, 4H), 2.40 (qt, *J* = 6.8, 1.4 Hz, 2H), 2.07 – 1.92 (m, 2H) ppm. **^{13}C NMR** (101 MHz, $CDCl_3$) δ 158.7, 134.9, 116.5, 72.8, 33.7, 31.6, 31.1, 14.6 ppm. **IR** (neat, cm^{-1}): 3077, 2959, 2928, 2870, 1641, 1370, 1042, 988, 914, 886, 866. **HRMS** (ESI+) *calcd.* for ($C_8H_{14}NO$) $[M+H]^+$: 140.1070; *found* 140.1071.

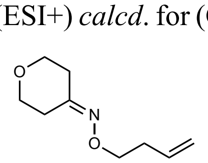

tetrahydro-4*H*-pyran-4-one *O*-but-3-en-1-yl oxime (1f). General procedure A was applied to tetrahydro-4*H*-pyran-4-one and but-3-en-1-ol (5.0 mmol) to afford **1f** by column chromatography as a colorless oil (0.66 g,

78%). **^{1}H NMR** (400 MHz, $CDCl_3$) δ 5.82 (ddt, *J* = 17.06, 10.23, 6.77 Hz, 1H), 5.12 – 5.05 (m, 1H), 5.04 (ddt, *J* = 10.2, 2.1, 1.2 Hz, 1H), 4.07 (t, *J* = 6.8 Hz, 2H), 3.80 (t, *J* = 5.7 Hz, 2H), 3.72 (t, *J* = 5.9 Hz, 2H), 2.61 (t, *J* = 5.8 Hz, 2H), 2.40 (qt, *J* = 6.8, 1.4 Hz, 2H), 2.35 (t, *J* = 5.7 Hz, 2H) ppm. **^{13}C NMR** (101 MHz, $CDCl_3$) δ 155.2, 135.1, 116.6, 72.6, 68.5, 66.9, 33.7, 32.5, 26.9 ppm. **IR** (neat, cm^{-1}): 3078, 2963, 2917, 2852, 1641, 1430, 1375, 1287, 1096, 1048, 997, 944, 913, 849, 688. **HRMS** (ESI+) *calcd.* for ($C_9H_{16}NO_2$) $[M+H]^+$: 170.1176; *found* 170.1180.

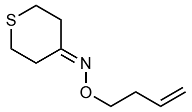

tetrahydro-4*H*-thiopyran-4-one *O*-but-3-en-1-yl oxime (1g). General procedure A was applied to tetrahydro-4*H*-thiopyran-4-one and but-3-en-1-ol (5.0 mmol) to afford **1g** by column chromatography as a colorless oil (0.68 g, 73%). **^{1}H NMR** (400 MHz, $CDCl_3$) δ 5.81 (ddt, *J* = 17.1, 10.2, 6.8 Hz, 1H), 5.12 – 5.05 (m, 1H), 5.03 (ddt, *J* = 10.2, 2.0, 1.2 Hz, 1H), 4.05 (t, *J* = 6.8 Hz, 2H), 2.84 – 2.74 (m, 4H), 2.73 – 2.67 (m, 2H), 2.57 – 2.50 (m, 2H), 2.39 (qt, *J* = 6.8, 1.4 Hz, 2H) ppm. **^{13}C NMR** (101 MHz, $CDCl_3$) δ 157.3, 135.0, 116.6, 72.7, 34.1, 33.7, 29.9, 28.5, 27.6 ppm. **IR** (neat, cm^{-1}): 3076, 2914, 2872, 2830, 1640, 1426, 1372, 1268, 1047, 984, 941, 913, 892, 879, 654. **HRMS** (ESI+) *calcd.* for ($C_9H_{16}NOS$) $[M+H]^+$: 186.0947; *found* 186.0943.

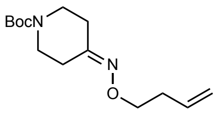

***tert*-butyl 4-((but-3-en-1-yloxy)imino)piperidine-1-carboxylate (1h).** General procedure A was applied to *tert*-butyl 4-oxopiperidine-1-carboxylate and but-3-en-1-ol (5.0 mmol) to afford **1h** by column chromatography as a colorless oil (0.94 g, 70%). **^{1}H NMR** (400 MHz, $CDCl_3$) δ 5.81 (ddt, *J* = 17.0, 10.2, 6.7 Hz, 1H), 5.11 – 5.05 (m, 1H), 5.05 – 5.00 (m, 1H), 4.06 (t, *J* = 6.8 Hz, 2H), 3.53 (t, *J* = 6.1 Hz, 2H), 3.48 (t, *J* = 6.1 Hz, 2H), 2.56 (t, *J* = 6.1 Hz, 2H), 2.45 – 2.34 (m, 2H), 2.32 (t, *J* = 6.1 Hz, 2H), 1.46 (s, 9H) ppm. **^{13}C NMR** (101 MHz, $CDCl_3$) δ 156.2, 154.8, 135.0, 116.6, 80.1, 72.7, 33.7, 31.2, 28.6, 25.7 ppm. **IR** (neat, cm^{-1}): 2976, 2929, 2871, 1694, 1477, 1417, 1365, 1275, 1237, 1162, 1115, 1048, 988, 915, 865. **HRMS** (ESI+) *calcd.* for ($C_{14}H_{24}N_2NaO_3$) $[M+Na]^+$: 291.1679; *found* 291.1686.

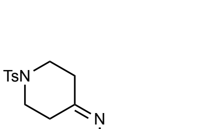

1-tosylpiperidin-4-one *O*-but-3-en-1-yl oxime (1i). General procedure A was applied to 1-tosylpiperidin-4-one and but-3-en-1-ol (5.0 mmol) to afford **1i** by column chromatography as a white solid (1.02 g, 63%). **^{1}H NMR** (400 MHz, $CDCl_3$) δ 7.67 – 7.61 (m, 2H), 7.35 – 7.29 (m, 2H), 5.77 (ddt, *J* = 17.1, 10.2, 6.8 Hz, 1H), 5.08 – 4.97 (m, 2H), 4.00 (t, *J* = 6.8 Hz, 2H), 3.18 (t, *J* = 5.9 Hz, 2H), 3.10 (t, *J* = 6.1 Hz, 2H), 2.66 (t, *J* = 6.1 Hz, 2H), 2.45 – 2.38 (m, 5H), 2.34 (qt, *J* = 6.8, 1.4 Hz, 2H) ppm. **^{13}C NMR** (101 MHz, $CDCl_3$) δ 154.2, 143.9, 134.9, 133.4, 129.9, 127.7, 116.7, 91.9, 35.4 (m), 33.6, 31.1, 24.9, 21.7 ppm. **IR** (neat, cm^{-1}): 3068, 2919, 2858, 1642, 1359, 1334, 1298, 1611, 1102, 1036, 995, 923, 876, 816, 728, 684, 647, 544. **HRMS** (ESI+) *calcd.* for ($C_{16}H_{22}N_2NaO_3S$) $[M+Na]^+$: 345.1243; *found* 345.1243.

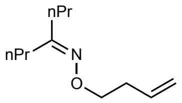

heptan-4-one *O*-but-3-en-1-yl oxime (1j). General procedure A was applied to heptan-4-one and but-3-en-1-ol (5.0 mmol) to afford **1j** by column chromatography as a colorless oil (0.81 g, 88%). **^{1}H NMR** (400 MHz, $CDCl_3$) δ 5.83 (ddt, *J* = 17.1, 10.2, 6.8 Hz, 1H), 5.11 – 5.04 (m, 1H), 5.02 (ddt, *J* = 10.2, 2.2, 1.2 Hz, 1H), 4.04 (t, *J* = 6.8 Hz, 2H), 2.39 (qt, *J* = 6.8, 1.4 Hz, 2H), 2.27 – 2.20 (m, 2H), 2.16 – 2.09 (m, 2H), 1.58 – 1.45 (m, 4H), 0.92 (t, *J* = 7.4 Hz, 6H) ppm. **^{13}C NMR** (101 MHz, $CDCl_3$) δ 161.4, 135.4, 116.4, 72.4, 36.3, 33.9, 30.2, 20.2, 19.4, 14.5, 14.0 ppm. **IR** (neat, cm^{-1}): 3080, 2961, 2932, 2873, 1641, 1457, 1433, 1372, 1051, 987, 947, 909. **HRMS** (ESI+) *calcd.* for ($C_{11}H_{22}NO$) $[M+H]^+$: 184.1696; *found* 184.1699.

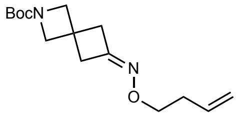

***tert*-butyl 6-((but-3-en-1-yloxy)imino)-2-azaspiro[3.3]heptane-2-carboxylate (1k).** General procedure A was applied to *tert*-butyl 6-oxo-2-azaspiro[3.3]heptane-2-carboxylate and but-3-en-1-ol (5.0 mmol) to afford **1k** by column chromatography as a colorless oil (0.81 g, 58%). **^{1}H NMR** (400 MHz, $CDCl_3$) δ 5.80 (ddt, *J* = 17.1, 10.2, 6.8 Hz, 1H), 5.11 – 5.05 (m, 1H), 5.05 – 5.00 (m, 1H), 4.04 (t, *J* = 6.9 Hz, 2H), 4.01 – 3.96 (m, 4H), 3.09 – 3.05 (m, 4H), 2.39 (qt, *J* = 6.9, 1.4 Hz, 2H), 1.43 (s, 9H) ppm. **^{13}C NMR** (101 MHz, $CDCl_3$) δ 156.2, 152.5, 134.8, 116.8, 79.8, 73.2, 43.0, 42.3, 33.8, 32.0, 28.5 ppm. **IR** (neat, cm^{-1}): 3077, 2976, 2931, 2873, 1698, 1642, 1478, 1391, 1365, 1253, 1156, 1095, 1038, 915. **HRMS** (ESI+) *calcd.* for ($C_{15}H_{24}N_2NaO_3$) $[M+Na]^+$: 303.1679; *found* 303.1683.

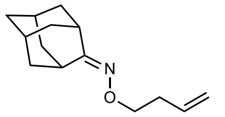

adamantan-2-one *O*-but-3-en-1-yl oxime (1l). General procedure A was applied to adamantan-2-one and but-3-en-1-ol (5.0 mmol) to afford **1l** by column chromatography as a colorless oil (0.74 g, 67%). **^{1}H NMR** (400 MHz, $CDCl_3$) δ 5.83 (ddt, *J* = 17.1, 10.3, 6.8 Hz, 1H), 5.11 – 5.04 (m, 1H), 5.02 (ddt, *J* = 10.2, 2.2, 1.3 Hz, 1H), 4.04 (t, *J* = 6.8 Hz, 2H), 3.48 (p, *J* = 3.3 Hz, 1H), 2.57 – 2.50 (m, 1H), 2.40 (qt, *J* = 6.8, 1.4 Hz, 2H), 2.00 – 1.92 (m, 4H), 1.91 – 1.75 (m, 8H) ppm. **^{13}C NMR** (101 MHz, $CDCl_3$) δ 167.0, 135.3, 116.4, 72.2, 39.2, 37.7, 36.7, 36.4, 33.9, 29.7, 28.0 ppm. **IR** (neat, cm^{-1}): 3077, 2911, 2852, 1641, 1450, 1086, 1047, 986, 894, 870, 823. **HRMS** (ESI+) *calcd.* for ($C_{14}H_{22}NO$) $[M+H]^+$: 220.1696; *found* 220.1696.

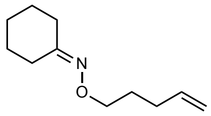

cyclohexanone *O*-pent-4-en-1-yl oxime (1m). General procedure A was applied to cyclohexanone and pent-4-en-1-ol (20.0 mmol) to afford **1m** by column chromatography as a colorless oil (2.93 g, 81%). **^{1}H NMR** (400 MHz, $CDCl_3$) δ 5.84 (ddt, *J* = 16.9, 10.2, 6.6 Hz, 1H), 5.06 – 4.99 (m, 1H), 4.96 (ddt, *J* = 10.2, 2.3, 1.3 Hz, 1H),

4.01 (t, *J* = 6.6 Hz, 2H), 2.46 (t, *J* = 6.0 Hz, 2H), 2.23 – 2.17 (m, 2H), 2.17 – 2.07 (m, 2H), 1.80 – 1.69 (m, 2H), 1.69 – 1.63 (m, 2H), 1.63 – 1.54 (m, 4H) ppm. **^{13}C NMR** (101 MHz, $CDCl_3$) δ 160.2, 138.5, 114.8, 72.7, 32.4, 30.4, 28.5, 27.2, 26.0, 25.9, 25.4 ppm. **IR** (neat, cm^{-1}): 3076, 2929, 2859, 1640, 1448, 1056, 1021, 990, 931, 912, 880, 837. **HRMS** (ESI+) *calcd.* for ($C_{11}H_{20}NO$) $[M+H]^+$: 182.1539; *found* 182.1532.

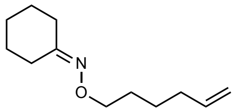

cyclohexanone *O*-hex-5-en-1-yl oxime (1n). General procedure A was applied to cyclohexanone and hex-5-en-1-ol (10.0 mmol) to afford **1n** by column chromatography as a colorless oil (1.46 g, 75%). **^{1}H NMR** (400 MHz, $CDCl_3$) δ 5.81 (ddt, *J* = 16.9, 10.2, 6.6 Hz, 1H), 5.05 – 4.98 (m, 1H), 4.94 (ddt, *J* = 10.2, 2.3, 1.3 Hz, 1H), 4.00 (t, *J* = 6.6 Hz, 2H), 2.50 – 2.40 (m, 2H), 2.24 – 2.15 (m, 2H), 2.14 – 2.01 (m, 2H), 1.72 – 1.61 (m, 4H), 1.64 – 1.55 (m, 4H), 1.51 – 1.40 (m, 2H) ppm. **^{13}C NMR** (101 MHz, $CDCl_3$) ^{13}C NMR (101 MHz, $CDCl_3$) δ 160.1, 139.0, 114.6, 73.2, 33.7, 32.4, 28.7, 27.2, 26.0, 25.9, 25.5, 25.4 ppm; **IR** (neat, cm^{-1}): 3078, 2929, 2859, 1640, 1440, 1436, 1377, 1046, 992, 933, 910. **HRMS** (ESI+) *calcd.* for ($C_{12}H_{22}NO$) $[M+H]^+$: 196. 1696; *found* 196. 1701.

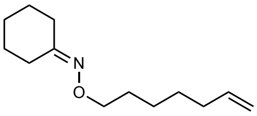

cyclohexanone *O*-hept-6-en-1-yl oxime (1o). General procedure A was applied to cyclohexanone and hept-6-en-1-ol (5.0 mmol) to afford **1o** by column chromatography as a colorless oil (0.82 g, 78%). **^{1}H NMR** (500 MHz, $CDCl_3$) δ 5.81 (ddt, *J* = 16.9, 10.2, 6.7 Hz, 1H), 4.99 (ddt, *J* = 17.1, 2.2, 1.6 Hz, 1H), 4.93 (ddt, *J* = 10.2, 2.3, 1.2 Hz, 1H), 3.99 (t, *J* = 6.7 Hz, 2H), 2.49 – 2.43 (m, 2H), 2.23 – 2.16 (m, 2H), 2.06 (tdd, *J* = 6.6, 5.4, 1.4 Hz, 2H), 1.69 – 1.62 (m, 4H), 1.64 – 1.54 (m, 4H), 1.46 – 1.32 (m, 4H) ppm. **^{13}C NMR** (126 MHz, CDC13) δ 160.2, 139.2, 114.4, 73.3, 33.9, 32.4, 29.1, 28.9, 27.2, 26.0, 25.9, 25.7, 25.4 ppm. **IR** (neat, cm^{-1}): 3076, 2928, 2857, 1640, 1448, 1053, 991, 937, 910, 874. **HRMS** *calcd.* for ($C_{13}H_{24}NO$) $[M+H]^+$: 210.1852; *found* 210.1853.

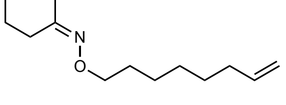

cyclohexanone *O*-oct-7-en-1-yl oxime (1p). General procedure B was applied to cyclohexanone and 8-Bromo-1-octene (5.0 mmol) to afford **1p** by column chromatography as a colorless oil (0.78 g, 70%). **^{1}H NMR** (400 MHz, $CDCl_3$) δ 5.8 (ddt, *J* = 16.9, 10.2, 6.7 Hz, 1H), 5.02 – 4.94 (m, 1H), 4.92 (ddt, *J* = 10.2, 2.3, 1.2 Hz, 1H), 3.98 (t, *J* = 6.7 Hz, 2H), 2.45 (td, *J* = 5.8, 2.6 Hz, 2H), 2.23 – 2.14 (m, 2H), 2.04 (tdd, *J* = 6.6, 5.3, 1.5 Hz, 2H), 1.70 – 1.54 (m, 8H), 1.45 – 1.28 (m, 6H) ppm. **^{13}C NMR** (101 MHz, $CDCl_3$) δ 160.0, 139.2, 114.3, 73.4, 33.9, 32.4, 29.1, 29.1, 29.0, 27.2, 26.0, 26.0, 25.9, 25.4 ppm. **IR** (neat, cm^{-1}): 3076, 2927, 2856, 1640, 1448, 1055, 992, 938, 910, 885, 838. **HRMS** (ESI+) *calcd.* for ($C_{14}H_{26}NO$) $[M+H]^+$: 224.2009; *found* 224.2016.

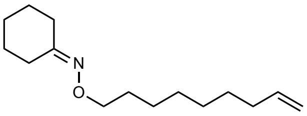

cyclohexanone O-non-8-en-1-yl oxime (1q). General procedure B was applied to cyclohexanone and 9-Bromo-1-octene (5.0 mmol) to afford **1q** by column chromatography as a colorless oil (0.85 g, 72%). **^{1}H NMR** (400 MHz, $CDCl_3$) δ 5.79 (ddt, *J* = 16.9, 10.1, 6.6 Hz, 1H), 5.02 – 4.94 (m, 1H), 4.91 (ddt, *J* = 10.1, 2.9, 1.3 Hz, 1H), 3.97 (t, *J* = 6.7 Hz, 2H), 2.44 (t, *J* = 6.0 Hz, 2H), 2.18 (dd, *J* = 7.3, 5.2 Hz, 2H), 2.02 (qd, *J* = 6.7, 3.4 Hz, 2H), 1.69 – 1.53 (m, 8H), 1.42 – 1.21 (m, 8H) ppm. **^{13}C NMR** (101 MHz, $CDCl_3$) δ 160.0, 139.3, 114.2, 73.4, 33.9, 32.4, 29.4, 29.2, 29.2, 29.0, 27.2, 26.1, 26.0, 25.9, 25.4. ppm **IR** (neat, cm^{-1}): 3077, 2925, 2855, 1640, 1449, 1376, 1054, 991, 933, 910, 888, 839. **HRMS** (ESI+) *calcd.* for ($C_{15}H_{28}NO$) $[M+H]^+$: 238.2165; *found* 238.2169.

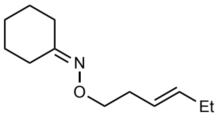

(*E*)-cyclohexanone *O*-hex-3-en-1-yl oxime (1r). General procedure A was applied to cyclohexanone and (*E*)-hex-3-en-1-ol (10.0 mmol) to afford **1r** by column chromatography as a colorless oil (1.76 g, 90%). **^{1}H NMR** (400 MHz, $CDCl_3$) δ 5.53 (dtt, *J* = 15.0, 6.1, 1.2 Hz, 1H), 5.40 (dtt, *J* = 15.1, 6.7, 1.4 Hz, 1H), 3.99 (t, *J* = 7.0 Hz, 2H), 2.49 – 2.39 (m, 2H), 2.32 (dddd, *J* = 8.0, 6.8, 5.7, 1.2 Hz, 2H), 2.23 – 2.14 (m, 2H), 2.06 – 1.94 (m, 2H), 1.70 – 1.62 (m, 2H), 1.62 – 1.55 (m, 4H), 0.96 (t, *J* = 7.5 Hz, 3H) ppm. **^{13}C NMR** (101 MHz, $CDCl_3$) δ 160.3, 134.3, 125.2, 73.0, 32.6, 32.4, 27.2, 26.0, 25.9, 25.8, 25.5, 14.0 ppm. **IR** (neat, cm^{-1}): 2930, 2859, 1449, 1372, 1047, 966, 931. **HRMS** (ESI+) *calcd.* for ($C_{12}H_{22}NO$) $[M+H]^+$: 196.1696; *found* 196.1696.

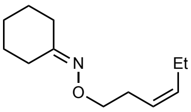

(*Z*)-cyclohexanone *O*-hex-3-en-1-yl oxime (1s). General procedure A was applied to cyclohexanone and (*Z*)-hex-3-en-1-ol (10.0 mmol) to afford **1s** by column chromatography as a colorless oil (1.78 g, 91%). **^{1}H NMR** (400 MHz, $CDCl_3$) δ 5.50 – 5.43 (m, 1H), 5.40 – 5.32 (m, 1H), 3.99 (t, *J* = 7.0 Hz, 2H), 2.48 – 2.42 (m, 2H), 2.42 – 2.36 (m, 2H), 2.22 – 2.17 (m, 2H), 2.11 – 2.02 (m, 2H), 1.70 – 1.63 (m, 2H), 1.62 – 1.56 (m, 4H), 0.96 (t, *J* = 7.5 Hz, 3H) ppm. **^{13}C NMR** (101 MHz, $CDCl_3$) δ 160.2, 133.8, 124.9, 72.8, 32.4, 27.5, 27.2, 26.0, 25.9, 25.4, 20.7, 14.4 ppm. **IR** (neat, cm^{-1}): 2930, 2860, 2860, 1449, 1370, 1044, 930, 916, 840. **HRMS** (ESI+) *calcd.* for ($C_{12}H_{22}NO$) $[M+H]^+$: 196.1696; *found* 196.1692.

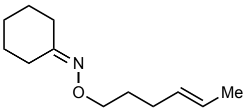

(*E*)-cyclohexanone *O*-hex-4-en-1-yl oxime (1t). General procedure A was applied to cyclohexanone and (*E*)-hex-4-en-1-ol (10.0 mmol) to afford **1t** by column chromatography as a colorless oil (1.68 g, 86%). **^{1}H NMR** (400 MHz, $CDCl_3$) δ 5.50 – 5.38 (m, 2H), 3.99 (t, J = 6.7 Hz, 2H), 2.50 – 2.41 (m, 2H), 2.25 – 2.15 (m, 2H), 2.09 – 2.00 (m, 2H), 1.73 – 1.66 (m, 2H), 1.66 – 1.61 (m, 4H), 1.63 – 1.56 (m, 2H), 1.57 – 1.55 (m, 3H) ppm.

^{13}C NMR (101 MHz, $CDCl_3$) δ 160.2, 130.9, 125.3, 72.8, 32.4, 29.2, 29.1, 27.2, 26.0, 25.9, 25.4, 18.1 ppm. **IR** (neat, cm^{-1}): 2930, 2858, 1448, 1377, 1052, 964, 932, 917, 878. **HRMS** (ESI+) *calcd.* for ($C_{12}H_{22}NO$) $[M+H]^+$: 196.1696; *found* 196.1698.

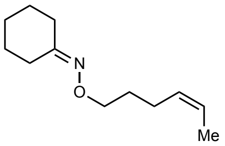

(*Z*)-cyclohexanone *O*-hex-4-en-1-yl oxime (1u). General procedure A was applied to cyclohexanone and (*Z*)-hex-4-en-1-ol (10.0 mmol) to afford **1u** by column chromatography as a colorless oil (1.56 g, 80%). **^{1}H NMR** (400 MHz, $CDCl_3$) δ 5.52 – 5.35 (m, 2H), 4.00 (t, J = 6.6 Hz, 2H), 2.47 (t, J = 6.0 Hz, 2H), 2.22 – 2.17 (m, 2H), 2.16 – 2.08 (m, 2H), 1.75 – 1.62 (m, 4H), 1.62 – 1.56 (m, 7H) ppm. **^{13}C NMR** (101 MHz, $CDCl_3$) δ 160.2, 130.1, 124.5, 72.7, 32.4, 29.1, 27.2, 26.0, 26.0, 25.4, 23.5, 12.8 ppm. **IR** (neat, cm^{-1}): 3012, 2927, 2858, 1448, 1372, 1056, 1035, 931, 877, 837, 700. **HRMS** (ESI+) *calcd.* for ($C_{12}H_{22}NO$) $[M+H]^+$: 196.1696; *found* 196.1694.

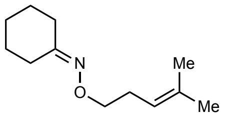

cyclohexanone *O*-(4-methylpent-3-en-1-yl) oxime (1v). General procedure B was applied to cyclohexanone and 5-Bromo-2-methyl-2-pentene (5.0 mmol) to afford **1v** by column chromatography as a colorless oil (0.64 g, 66%). **^{1}H NMR** (400 MHz, $CDCl_3$) δ 5.17 – 5.11 (m, 1H), 3.96 (t, J = 7.1 Hz, 2H), 2.49 – 2.42 (m, 2H), 2.33 (qt, J = 7.2, 1.1 Hz, 2H), 2.23 – 2.14 (m, 2H), 1.70 (q, J = 1.3 Hz, 3H), 1.71 – 1.63 (m, 2H), 1.63 (d, J = 1.3 Hz, 3H), 1.64 – 1.53 (m, 4H) ppm. **^{13}C NMR** (101 MHz, $CDCl_3$) δ 160.2, 133.7, 120.4, 72.9, 32.4, 28.4, 27.2, 26.0, 25.9, 25.8, 25.4, 18.0 ppm. **IR** (neat, cm^{-1}): 2927, 2859, 1640, 1448, 1375, 1045, 1024, 931, 917, 874, 840. **HRMS** (ESI+) *calcd.* for ($C_{12}H_{22}NO$) $[M+H]^+$: 196.1696; *found* 196.1687.

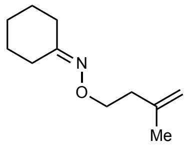

cyclohexanone *O*-(3-methylbut-3-en-1-yl) oxime (1w). General procedure A was applied to cyclohexanone and 3-methylbut-3-en-1-ol (10.0 mmol) to afford **1w** by column chromatography as a colorless oil (1.22 g, 66%). **^{1}H NMR** (400 MHz, $CDCl_3$) δ 4.77 (qd, J = 1.5, 0.8 Hz, 1H), 4.73 (dt, J = 2.3, 1.1 Hz, 1H), 4.11 (t, J = 6.9 Hz, 2H), 2.49 – 2.41 (m, 2H), 2.36 (td, J = 7.0, 1.3 Hz, 2H), 2.25 – 2.15 (m, 2H), 1.76 (t, J = 1.1 Hz, 3H), 1.72 – 1.62 (m, 2H), 1.62 – 1.56 (m, 4H) ppm. **^{13}C NMR** (126 MHz, $CDCl_3$) δ 160.4, 143.1, 111.6, 71.8, 37.4, 32.4, 27.2, 26.0, 25.9, 25.5, 23.0 ppm. **IR** (neat, cm^{-1}): 3075, 2930, 2859, 1649, 1448, 1374, 1106, 1045, 934, 916, 886, 839. **HRMS** (ESI+) *calcd.* for ($C_{11}H_{20}NO$) $[M+H]^+$: 182.1539; *found* 182.1537.

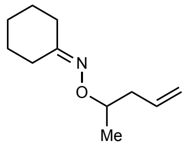

cyclohexanone *O*-pent-4-en-2-yl oxime (1x). General procedure A was applied to cyclohexanone and pent-4-en-2-ol (10.0 mmol) to afford **1x** by column chromatography as a colorless oil (1.02 g, 56%). **^{1}H NMR** (400 MHz, $CDCl_3$) δ 5.81 (ddt, *J* = 17.2, 10.2, 7.1 Hz, 1H), 5.11 – 4.99 (m, 2H), 4.23 – 4.12 (m, 1H), 2.51 – 2.37 (m, 3H), 2.28 – 2.15 (m, 3H), 1.69 – 1.62 (m, 2H), 1.61 – 1.55 (m, 4H), 1.20 (d, *J* = 6.3 Hz, 3H) ppm. **^{13}C NMR** (101 MHz, $CDCl_3$) δ 160.0, 135.1, 116.8, 77.4, 40.3, 32.5, 27.2, 26.0, 25.9, 25.5, 19.3 ppm. **IR** (neat, cm^{-1}): 3076, 2973, 2930, 2858, 1641, 1448, 1334, 1126, 1080, 991, 957, 942, 913, 880, 841. **HRMS** (ESI+) *calcd.* for ($C_{11}H_{20}NO$) $[M+H]^+$: 182.1539; *found* 182.1542.

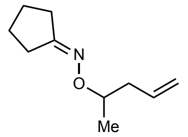

cyclopentanone O-pent-4-en-2-yl oxime (1y). General procedure A was applied to cyclopentanone and pent-4-en-2-ol (10.0 mmol) to afford **1y** by column chromatography as a colorless oil (1.00 g, 60%). **^{1}H NMR** (400 MHz, $CDCl_3$) δ 5.82 (ddt, *J* = 17.2, 10.2, 7.1 Hz, 1H), 5.11 – 5.00 (m, 2H), 4.24 – 4.14 (m, 1H), 2.47 – 2.33 (m, 5H), 2.28 – 2.19 (m, 1H), 1.78 – 1.68 (m, 4H), 1.21 (d, *J* = 6.3 Hz, 3H) ppm. **^{13}C NMR** (101 MHz, $CDCl_3$) δ 166.2, 135.1, 116.9, 77.9, 40.4, 31.1, 27.9, 25.3, 24.8, 19.5 ppm. **IR** (neat, cm^{-1}): 3076, 2964, 1642, 1432, 1373, 1334, 1216, 1125, 1079, 994, 911, 885. **HRMS** (ESI+) *calcd.* for ($C_{10}H_{18}NO$) $[M+H]^+$: 168.1383; *found* 168.1379.

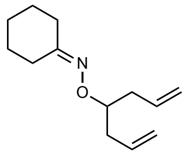

cyclohexanone *O*-hepta-1,6-dien-4-yl oxime (1z). General procedure A was applied to cyclohexanone and hepta-1,6-dien-4-ol (10.0 mmol) to afford **1z** by column chromatography as a colorless oil (1.46 g, 70%). **^{1}H NMR** (500 MHz, $CDCl_3$) δ 5.85 (ddt, *J* = 17.2, 10.2, 7.1 Hz, 2H), 5.12 – 5.04 (m, 4H), 4.14 (p, *J* = 6.0 Hz, 1H), 2.51 – 2.45 (m, 2H), 2.44 – 2.31 (m, 4H), 2.26 – 2.18 (m, 2H), 1.71 – 1.65 (m, 2H), 1.65 – 1.56 (m, *J* = 2.2 Hz, 4H) ppm. **^{13}C NMR** (101 MHz, $CDCl_3$) δ 160.3, 134.9, 116.8, 80.5, 37.5, 32.3, 27.1, 25.9, 25.8, 25.5 ppm. **IR** (neat, cm^{-1}): 3076, 2977, 2931, 2858, 1641, 1448, 1435, 1345, 1022, 891, 940, 910, 841. **HRMS** (ESI+) *calcd.* for ($C_{13}H_{22}NO$) $[M+H]^+$: 208.1696; *found* 208.1697.

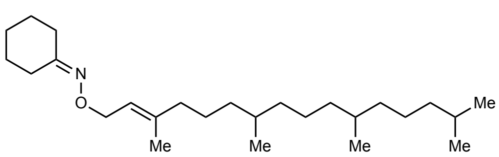

(*E*)-cyclohexanone *O*-(3,7,11,15-tetramethylhexadec-2-en-1-yl) oxime (1aa). General procedure A was applied to cyclohexanone and (*E*)-3,7,11,15-tetramethylhexadec-2-en-1-ol (Phytol, 5.0 mmol) to afford **1aa** by column chromatography as a colorless oil (1.5 g, 77%). **^{1}H NMR** (400 MHz, $CDCl_3$) δ 5.40 (tq, *J* = 6.7, 1.3 Hz,

1H), 4.55 (dq, J = 6.7, 0.8 Hz, 2H), 2.51 – 2.42 (m, 2H), 2.25 – 2.19 (m, 2H), 2.00 (t, J = 7.7 Hz, 2H), 1.71 – 1.64 (m, 5H), 1.63 – 1.48 (m, 5H), 1.45 – 1.33 (m, 4H), 1.33 – 1.19 (m, 8H), 1.17 – 1.11 (m, 2H), 1.13 – 0.99 (m, 4H), 0.87 (d, J = 0.6 Hz, 3H), 0.86 – 0.85 (m, 6H), 0.84 (d, J = 2.1 Hz, 3H) ppm. **^{13}C NMR** (101 MHz, $CDCl_3$) δ 160.9, 141.3, 119.8, 70.3, 40.1, 39.5, 37.6, 37.5, 37.4, 36.8, 32.9, 32.8, 32.4, 28.1, 27.2, 25.9, 25.9, 25.6, 25.3, 24.9, 24.6, 22.9, 22.8, 19.9, 19.9, 16.7 ppm. **IR** (neat, cm^{-1}): 2923, 2853, 1449, 1378, 1054, 937, 875, 839, 722. **LCMS** (ESI+) *calcd.* for ($C_{26}H_{50}NO$) $[M+H]^+$: 392.4; *found* 391.9.

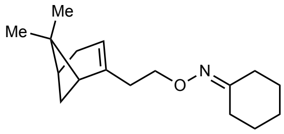

cyclohexanone *O*-(2-((1*R*,5*S*)-6,6-dimethylbicyclo[3.1.1]hept-2-en-2-yl)ethyl) oxime (1ab). General procedure A was applied to cyclohexanone and 2-((1*R*,5*S*)-6,6-dimethylbicyclo[3.1.1]hept-2-en-2-yl)ethan-1-ol [(1*R*)-(−)-Nopol, 5.0 mmol] to afford **1ab** by column chromatography as a colorless oil (1.05 g, 80%). **^{1}H NMR** (400 MHz, $CDCl_3$) δ 5.26 (tt, J = 2.9, 1.4 Hz, 1H), 4.01 (td, J = 7.1, 1.4 Hz, 2H), 2.48 – 2.40 (m, 2H), 2.39 – 2.24 (m, 3H), 2.24 – 2.16 (m, 4H), 2.10 – 2.03 (m, 2H), 1.71 – 1.60 (m, 2H), 1.60 – 1.56 (m, 4H), 1.26 (s, 3H), 1.15 (d, J = 8.5 Hz, 1H), 0.83 (s, 3H) ppm. **^{13}C NMR** (101 MHz, $CDCl_3$) δ 160.3, 145.3, 118.1, 71.8, 46.1, 40.9, 38.1, 36.7, 32.4, 31.8, 31.5, 27.2, 26.5, 26.0, 25.9, 25.5, 21.3 ppm. **IR** (neat, cm^{-1}): 2984, 2923, 2833, 1641, 1448, 1365, 1255, 1219, 1045, 934, 917, 887, 839. **LCMS** (ESI+) *calcd.* for ($C_{17}H_{28}NO$) $[M+H]^+$: 262.2; *found* 261.9.

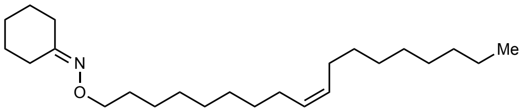

(*Z*)-cyclohexanone *O*-octadec-9-en-1-yl oxime (1ac). In a round bottom flask a solution of Oleic acid (10.0 mmol, 0.2 M) in anhydrous THF was prepared. A solution of $LiAlH_4$ (2.4 M in THF, 10.00 mL, 24.0 mmol, 2.4 equiv.) was added dropwise to the flask at 0 °C, then the reaction mixture was allowed to warm to room temperature and was stirred for 3 hours. Progress of the reaction was monitored via TLC. The reaction mixture was cooled to 0 °C and quenched by adding consecutively diethyl ether (100 mL), water (5 mL), 15% NaOH(aq.) (5 mL) and then again 15 mL of water. The mixture was warmed to RT and stirred for 15 minutes. Na_2SO_4 was added and the suspension was stirred for additional 15 minutes. The solution was filtered through celite pad and the filtrate was evaporated under vacuum, dried under vacuum to afford Oleic alcohol as a colorless oil. The Oleic alcohol was used directly in the next step without further purification. General procedure A was applied to cyclohexanone and Oleic alcohol to afford **1ac** by column chromatography as a colorless oil (2.18 g, 60%, two steps). **^{1}H NMR** (400 MHz, $CDCl_3$) δ 5.40 – 5.28 (m, 2H), 4.00 (t, J = 6.8 Hz, 2H), 2.50 – 2.42 (m, 2H), 2.23 – 2.16 (m, 2H), 2.05 – 1.96 (m, 4H), 1.71 – 1.56 (m, 8H), 1.38 – 1.23 (m, 22H), 0.88 (t, J = 7.0 Hz, 3H) ppm. **^{13}C NMR** (101 MHz, $CDCl_3$) δ 160.3, 130.1, 130.0, 73.5, 32.3, 32.0, 29.9, 29.9, 29.7, 29.6, 29.6, 29.5, 29.4, 29.2, 27.4, 27.2, 26.2, 26.0, 25.9, 25.5, 22.8, 14.2 ppm. **IR** (neat, cm^{-1}): 2925, 2858, 1461, 1377, 1029, 933, 917, 891, 871, 839. **LCMS** (ESI+) *calcd.* for ($C_{24}H_{46}NO$) $[M+H]^+$: 364.4; *found* 363.9.

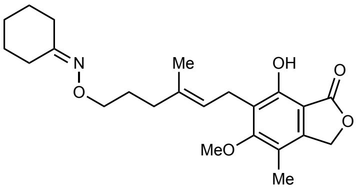

(*E*)-6-(6-((cyclohexylideneamino)oxy)-3-methylhex-2-en-1-yl)-7-hydroxy-5-methoxy-4-methylisobenzofuran-1(3*H*)-one (1ad). According to the reported method[4], the corresponding alcohol was prepared via the following procedures:

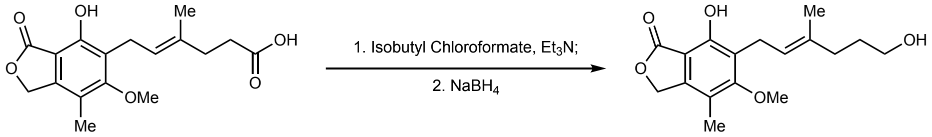

Then general procedure A was applied to cyclohexanone and the corresponding alcohol to afford **1ad** by column chromatography as a colorless oil (40%, 3 steps). **^{1}H NMR** (400 MHz, $CDCl_3$) δ 5.24 – 5.18 (m, 3H), 3.97 (t, *J* = 6.7 Hz, 2H), 3.76 (s, 3H), 3.39 (d, *J* = 6.9 Hz, 2H), 2.46 – 2.42 (m, 2H), 2.23 – 2.18 (m, 2H), 2.14 (s, 3H), 2.06 – 2.00 (m, 2H), 1.79 (d, *J* = 1.3 Hz, 3H), 1.78 – 1.68 (m, 2H), 1.70 – 1.62 (m, 2H), 1.61 – 1.55 (m, 4H) ppm. **^{13}C NMR** (101 MHz, $CDCl_3$) δ 173.1, 163.8, 160.9, 153.8, 144.0, 135.5, 122.6, 122.2, 116.8, 106.5, 73.1, 70.2, 61.1, 36.0, 32.3, 27.4, 27.1, 25.9, 25.9, 25.5, 22.8, 16.3, 11.7 ppm. **IR** (neat, cm^{-1}): 2933, 2859, 1759, 1602, 1475, 1361, 1317, 1200, 1129, 1104, 1078, 1036, 993, 966. **LCMS** (ESI+) *calcd.* for ($C_{23}H_{32}NO_5$) $[M+H]^+$: 402.2; *found* 401.8.

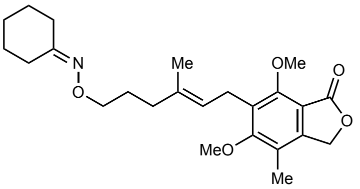

(*E*)-6-(6-((cyclohexylideneamino)oxy)-3-methylhex-2-en-1-yl)-5,7-dimethoxy-4-methylisobenzofuran-1(3*H*)-one (1ae). According to the reported method[5], the corresponding alcohol was prepared via the following procedures:

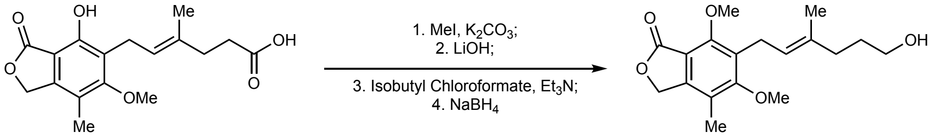

Then general procedure A was applied to cyclohexanone and the corresponding alcohol to afford **1ae** by column chromatography as a colorless oil (10%, 5 steps). **^{1}H NMR** (300 MHz, $CDCl_3$) δ 5.20 – 5.14 (m, 1H), 5.13 (s, 2H), 4.04 (s, 3H), 3.94 (t, *J* = 6.7 Hz, 2H), 3.77 (s, 3H), 3.40 (d, *J* = 6.8 Hz, 2H), 2.48 – 2.38 (m, 2H), 2.21 – 2.18 (m, 5H), 2.07 – 1.98 (m, 2H), 1.79 (d, *J* = 1.3 Hz, 3H), 1.78 – 1.66 (m, 3H), 1.66 – 1.51 (m, 5H) ppm. **^{13}C NMR (101 MHz, $CDCl_3$)** δ 169.1, 162.9, 160.2, 157.0, 146.7, 135.3, 129.5, 123.1, 120.1, 112.7, 72.9, 68.5, 62.8, 61.2, 36.1, 32.4, 27.5, 27.2, 26.0, 25.9, 25.4, 23.6, 16.3, 11.7 ppm. **IR** (neat, cm^{-1}): 2931, 2859, 1759, 1599, 1474, 1449, 1359, 1315, 1198, 1128, 1101, 1075, 1035, 996, 967. **LCMS** (ESI+) *calcd.* for ($C_{24}H_{34}NO_5$) $[M+H]^+$: 416.2; *found* 415.7.

2a-2u and **4a-4p** are either commercially available or prepared by known procedures.[3,6-7]

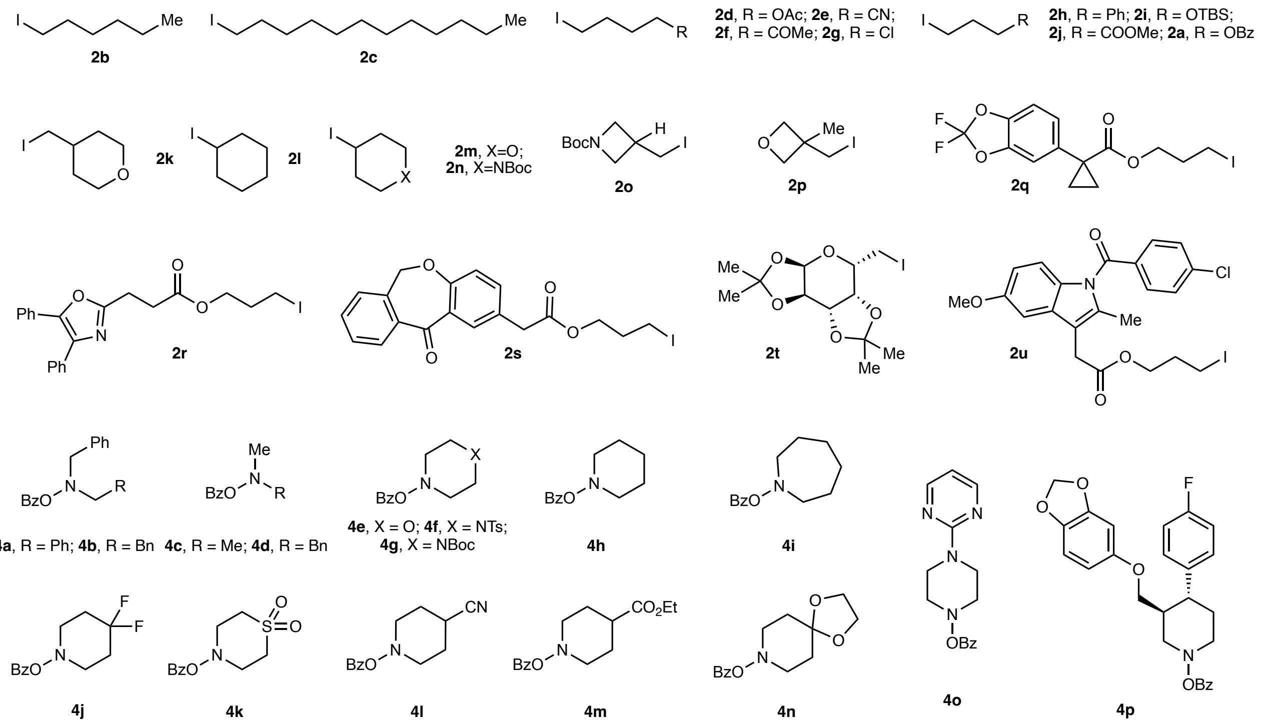

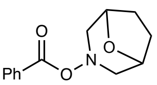

8-oxa-3-azabicyclo[3.2.1]octan-3-yl benzoate (4q). General procedure C was applied to benzoperoxide and 8-oxa-3-azabicyclo[3.2.1]octane (5.0 mmol) to afford **4q** by column chromatography as a white solid (1.08 g, 85%). **^{1}H NMR** (500 MHz, $CDCl_3$) δ 8.01 – 7.94 (m, 2H), 7.59 – 7.52 (m, 1H), 7.47 – 7.39 (m, 2H), 4.51 – 4.44 (m, 2H), 3.45 – 3.37 (m, 2H), 3.07 (dd, J = 9.6, 2.3 Hz, 2H), 2.26 – 2.16 (m, 2H), 2.00 – 1.88 (m, 2H) ppm. **^{13}C NMR** (101 MHz, $CDCl_3$) δ 164.7, 133.2, 129.5, 128.5, 75.2, 61.9, 28.4 ppm. **IR** (neat, cm^{-1}): 2980, 2963, 2952, 2851, 1737, 1445, 1249, 1230, 1087, 1067, 1042, 1024, 984, 877, 864, 770, 715, 674. **HRMS** (ESI+) *calcd*. for ($C_{13}H_{15}NNaO_3$) $[M+Na]^+$: 256.0944; *found* 256.0945. **MP**: 87 – 89 ºC.

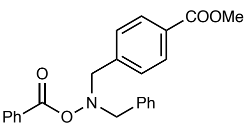

methyl 4-(((benzoyloxy)(benzyl)amino)methyl)benzoate (4r). Methyl 4-((benzylamino)methyl)benzoate was prepared via reported method[8], then general procedure C was applied to benzoperoxide and methyl 4-((benzylamino)methyl)benzoate (5.0 mmol) to afford **4r** by column chromatography as a white solid (1.68 g, 90%). **^{1}H NMR** (500 MHz, $CDCl_3$) δ 7.99 – 7.94 (m, 2H), 7.85 – 7.78 (m, 2H), 7.55 – 7.51 (m, 2H), 7.51 – 7.44 (m, 3H), 7.40 – 7.23 (m, 5H), 4.24 (s, 2H), 4.22 (s, 2H), 3.88 (s, 3H) ppm. **^{13}C NMR** (101 MHz, $CDCl_3$) δ 167.0, 164.9, 141.5, 135.6, 133.1, 129.8, 129.6, 129.6, 129.4, 129.2, 129.2, 128.6, 128.5, 128.0, 62.7, 61.6, 52.1

ppm. **IR** (neat, cm^{-1}): 3031, 2949, 1719, 1612, 1494, 1439, 1276, 1252, 1175, 1087, 1066, 1018, 991, 828, 757, 697. **HRMS** (ESI+) *calcd*. for ($C_{23}H_{22}NO_4$) $[M+Na]^+$: 376.1543; *found* 376.1543. **MP**: 98 – 100 ºC.

4. Ni-catalyzed interrupted chain-walking of unsaturated aliphatic alcohols

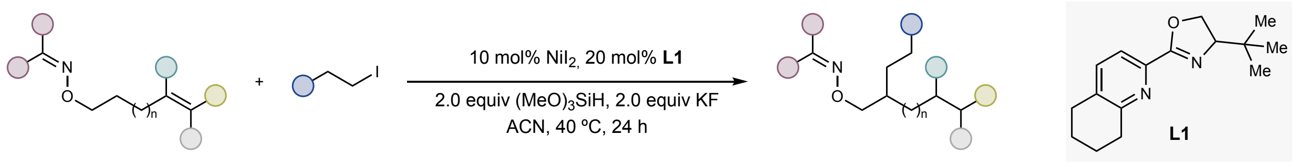

General procedure E for the β-alkylation of unsaturated aliphatic alcohols. To a glass screw 12 mL tube was added **L1** (20 mol%, 0.04 mmol) and brought inside a nitrogen-filled glovebox. Then, NiI_2 (10 mol%, 0.02 mmol) and KF (2.0 equiv., 0.40 mmol) were added, and the tube was sealed with a Teflon-lined screw cap and taken outside of the glovebox. Then, alkene (1.5 equiv., 0.30 mmol), alkyl iodide (1.0 equiv., 0.20 mmol), $(MeO)_3SiH$ (2.0 equiv., 0.40 mmol) and anhydrous MeCN (2.0 mL) were added subsequently via syringe, and the reaction mixture stirred at 40 °C for 24 h. The reaction was cooled to rt and diluted with 10 mL diethyl ether. The resulting organic layer was filtered through celite and evaporated under reduced pressure. The crude was purified by flash column chromatography (*n*-hexane/ethyl acetate) to afford the targeted β-alkylated products.

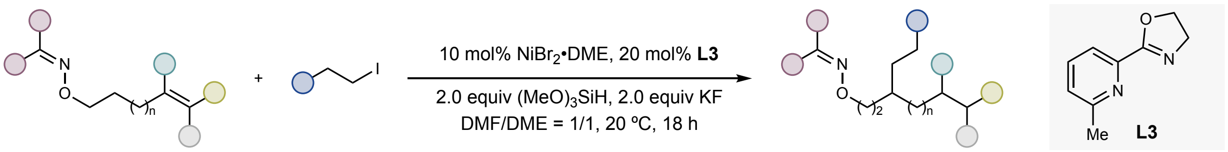

General procedure F for the γ-alkylation of unsaturated aliphatic alcohols. To a glass screw 12 mL tube was added **L3** (20 mol%, 0.04 mmol) and brought inside a nitrogen-filled glovebox. Then, $NiBr_2$•DME (10 mol%, 0.02 mmol) and KF (2.0 equiv., 0.40 mmol) were added, and the tube was sealed with a Teflon-lined screw cap and taken outside of the glovebox. Then, alkene (1.2 equiv., 0.24 mmol), alkyl iodide (1.0 equiv., 0.20 mmol), $(MeO)_3SiH$ (2.0 equiv., 0.40 mmol), anhydrous DMF (1.0 mL) and anhydrous 1,2-Dimethoxyethane (1.0 mL) were added subsequently via syringe, and the reaction mixture stirred at 20 °C for 18 h. The reaction was cooled down to rt and diluted with 10 mL ethyl acetate. The resulting organic layer was washed three times with brine and water, dried over Na_2SO_4, filtered, and concentrated in vacuo. The crude was purified by flash chromatography (*n*-hexane/ethyl acetate) to afford the targeted γ-alkylated products.

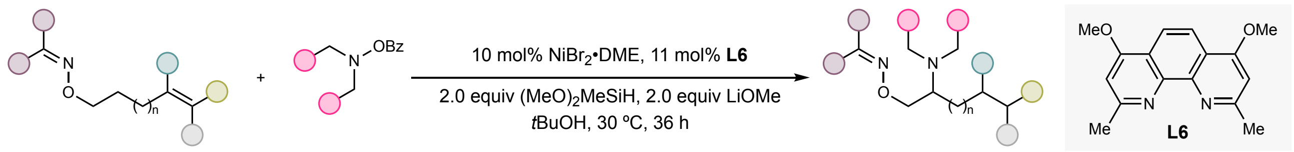

General procedure G for the β-amination of unsaturated aliphatic alcohols. To a glass screw 12 mL tube was added **L6** (11 mol%, 0.022 mmol), nitrogen-containing electrophile (1.5 equiv., 0.30 mmol) and brought inside a nitrogen-filled glovebox. Then, $NiBr_2 \cdot DME$ (10 mol%, 0.02 mmol) and LiOMe (2.0 equiv., 0.40 mmol) were added and the tube was sealed with a Teflon-lined screw cap and taken outside of the glovebox. Then, alkene (1.0 equiv., 0.20 mmol), $(MeO)_2MeSiH$ (2.0 equiv., 0.40 mmol) and anhydrous *t*BuOH (2.0 mL) were added subsequently via syringe, and the reaction mixture was stirred at 30 °C for 36 h. The reaction was cooled down to rt and diluted with 8 mL ethyl acetate. The resulting organic layer was washed three times with brine

and water, dried over Na_2SO_4, filtered, and concentrated in vacuo. The crude was purified by flash chromatography (*n*-hexane/ethyl acetate) to afford the targeted β-aminated products.

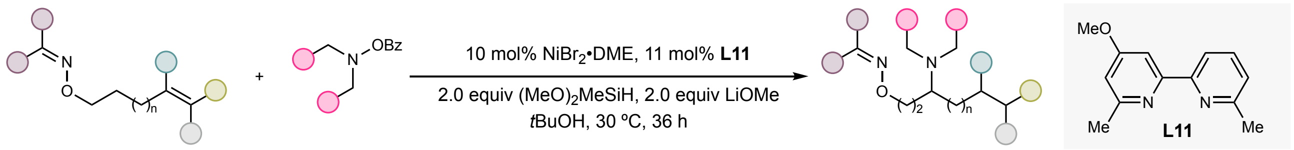

General procedure H for the γ-amination of unsaturated aliphatic alcohols. To a glass screw 12 mL tube was added **L11** (11 mol%, 0.022 mmol), nitrogen-containing electrophile (1.5 equiv., 0.30 mmol) and brought inside a nitrogen-filled glovebox. Then, $NiBr_2$·DME (10 mol%, 0.02 mmol) and LiOMe (2.0 equiv., 0.40 mmol) were added, and the tube was sealed with a Teflon-lined screw cap and taken outside of the glovebox. Then, alkene (1.0 equiv., 0.20 mmol), $(MeO)_2MeSiH$ (2.0 equiv., 0.40 mmol) and anhydrous *t*-BuOH (2.0 mL) were added subsequently via syringe, and the reaction mixture stirred at 30 °C for 36 h. The reaction was cooled down to rt and diluted with 10 mL ethyl acetate. The resulting organic layer was washed three times with brine and water, dried over Na_2SO_4, filtered, and concentrated in vacuo. The crude was purified by flash chromatography (*n*-hexane/ethyl acetate) to afford the targeted γ-aminated products.

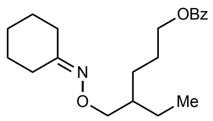

4-(((cyclohexylideneamino)oxy)methyl)hexyl benzoate (3a). Following general procedure E, using cyclohexanone *O*-but-3-en-1-yl oxime (**1a**, 50.2 mg, 0.30 mmol) and 3-iodopropyl benzoate (**2a**, 58.0 mg, 0.20 mmol), purification by flash column chromatographic (*n*-hexane/EtOAc 25/1) afforded **3a** (56.3 mg, 85% yield) as colorless oil. In an independent experiment, 58.3 mg (88% yield) were obtained, giving an average yield of 87%. ^{1}**H NMR** (400 MHz, $CDCl_3$) δ 8.07 – 8.01 (m, 2H), 7.58 – 7.51 (m, 1H), 7.47 – 7.39 (m, 2H), 4.31 (t, *J* = 6.6 Hz, 2H), 3.98 (dd, *J* = 10.1, 5.8 Hz, 1H), 3.91 (dd, *J* = 10.1, 6.5 Hz, 1H), 2.48 – 2.38 (m, 2H), 2.22 – 2.12 (m, 2H), 1.86 – 1.76 (m, 2H), 1.75 – 1.69 (m, 1H), 1.68 – 1.62 (m, 2H), 1.62 – 1.52 (m, 4H), 1.53 – 1.45 (m, 2H), 1.44 – 1.34 (m, 2H), 0.92 (t, *J* = 7.5 Hz, 3H) ppm. ^{13}**C NMR** (101 MHz, $CDCl_3$) δ 166.8, 160.2, 132.9, 130.7, 129.7, 128.4, 76.0, 65.5, 38.9, 32.3, 27.5, 27.2, 26.2, 26.0, 25.9, 25.5, 24.0, 11.3 ppm. **IR** (neat, cm^{-1}): 2929, 2859, 1718, 1450, 1270, 1109, 1069, 1046, 1026, 709. **HRMS** (ESI+): m/z *calcd.* for ($C_{20}H_{29}NNaO_3$) $[M+Na]^+$: 354.2040; *found* 354.2047.

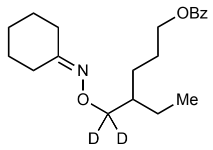

4-(((cyclohexylideneamino)oxy)methyl-*d*$_2$)hexyl benzoate (3a-*d*$_2$). Following general procedure E, using cyclohexanone *O*-but-3-en-1-yl-1,1-*d*$_2$ oxime (**1a-*d*$_2$**, 50.8 mg, 0.30 mmol) and 3-iodopropyl benzoate (**2a**, 58.0 mg, 0.20 mmol), purification by flash column chromatography (*n*-hexane/EtOAc 25/1) afforded **3a-*d*$_2$** (55.1 mg, 83% yield) as colorless oil. In an independent experiment, 58.0 mg (87% yield) were obtained, giving an average

yield of 85%. **^{1}H NMR** (400 MHz, $CDCl_3$) δ 8.05 – 8.00 (m, 2H), 7.56 – 7.50 (m, 1H), 7.45 – 7.38 (m, 2H), 4.30 (t, *J* = 6.6 Hz, 2H), 2.46 – 2.38 (m, 2H), 2.20 – 2.15 (m, 2H), 1.85 – 1.76 (m, 2H), 1.73 – 1.60 (m, 3H), 1.59 – 1.51 (m, 4H), 1.52 – 1.41 (m, 2H), 1.42 – 1.32 (m, 2H), 0.91 (t, *J* = 7.5 Hz, 3H) ppm. **^{13}C NMR** (101 MHz, $CDCl_3$) δ 166.7, 160.0, 132.8, 130.6, 129.6, 128.4, 75.2 (p, *J* = 22.1 Hz), 65.4, 38.6, 32.3, 27.4, 27.1, 26.1, 25.9, 25.8, 25.4, 23.9, 11.2 ppm. **IR** (neat, cm^{-1}): 2931, 2858, 1718, 1450, 1314, 1270, 1110, 1070, 1002, 709. **HRMS** (ESI+): m/z *calcd.* for ($C_{20}H_{27}D_2NNaO_3$) $[M+Na]^+$: 356.2165; *found* 356.2171.

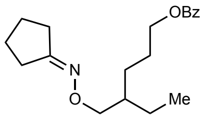

4-(((cyclopentylideneamino)oxy)methyl)hexyl benzoate (3b). Following general procedure E, cyclopentanone *O*-but-3-en-1-yl oxime (**1b**, 46.0 mg, 0.30 mmol) and 3-iodopropyl benzoate (**2a**, 58.0 mg, 0.20 mmol), purification by flash column chromatography (*n*-hexane/EtOAc 25/1) afforded **3b** (44.4 mg, 70%) as colorless oil. In an independent experiment, 48.2 mg (76% yield) were obtained, giving an average yield of 73%. **^{1}H NMR** (400 MHz, $CDCl_3$) δ 8.07 – 8.00 (m, 2H), 7.58 – 7.51 (m, 1H), 7.47 – 7.38 (m, 2H), 4.31 (t, *J* = 6.6 Hz, 2H), 4.00 (dd, *J* = 10.2, 5.8 Hz, 1H), 3.93 (dd, *J* = 10.2, 6.6 Hz, 1H), 2.40 – 2.31 (m, 4H), 1.87 – 1.75 (m, 2H), 1.75 – 1.66 (m, 5H), 1.59 – 1.34 (m, 4H), 0.92 (t, *J* = 7.5 Hz, 3H) ppm. **^{13}C NMR** (101 MHz, $CDCl_3$) δ 166.8, 166.3, 132.9, 130.7, 129.7, 128.4, 76.3, 65.5, 38.9, 31.1, 27.8, 27.4, 26.2, 25.3, 24.8, 24.0, 11.2 ppm. **IR** (neat, cm^{-1}): 2960, 2931, 2871, 1718, 1452, 1271, 1109, 1069, 1047, 1026, 711. **HRMS** (ESI+): m/z *calcd.* for ($C_{19}H_{27}NNaO_3$) $[M+Na]^+$: 340.1883; *found* 340.1882.

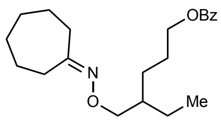

4-(((cycloheptylideneamino)oxy)methyl)hexyl benzoate (3c). Following general procedure E, using cycloheptanone *O*-but-3-en-1-yl oxime (**1c**, 54.4 mg, 0.30 mmol) and 3-iodopropyl benzoate (**2a**, 58.0 mg, 0.20 mmol), purification by flash column chromatography (*n*-hexane/EtOAc 25/1) afforded **3c** (56.7 mg, 82% yield) as colorless oil. In an independent experiment, 58.7 mg (85% yield) were obtained, giving an average yield of 83%. **^{1}H NMR** (400 MHz, $CDCl_3$) δ 8.08 – 8.01 (m, 2H), 7.59 – 7.52 (m, 1H), 7.47 – 7.40 (m, 2H), 4.32 (t, *J* = 6.6 Hz, 2H), 3.99 (dd, *J* = 10.1, 5.7 Hz, 1H), 3.92 (dd, *J* = 10.1, 6.5 Hz, 1H), 2.52 – 2.47 (m, 2H), 2.38 – 2.31 (m, 2H), 1.87 – 1.77 (m, 2H), 1.75 – 1.66 (m, 1H), 1.65 – 1.47 (m, 9H), 1.47 – 1.35 (m, 3H), 0.92 (t, *J* = 7.5 Hz, 3H) ppm. **^{13}C NMR** (101 MHz, $CDCl_3$) δ 166.7, 163.5, 132.9, 130.6, 129.6, 128.4, 76.0, 65.5, 38.9, 33.8, 30.5, 30.4, 29.3, 27.9, 27.5, 26.2, 24.8, 24.0, 11.2 ppm. **IR** (neat, cm^{-1}): 2930, 2859, 1719, 1450, 1314, 1271, 1111, 1069, 1041, 1027, 711. **HRMS** (ESI+): m/z *calcd.* for ($C_{21}H_{31}NNaO_3$) $[M+Na]^+$: 368.2196; *found* 368.2192.

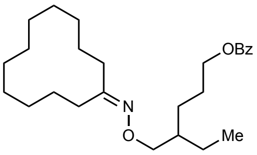

4-(((cyclododecylideneamino)oxy)methyl)hexyl benzoate (3d). Following general procedure E, using cyclododecanone *O*-but-3-en-1-yl oxime (**1d**, 75.4 mg, 0.30 mmol) and 3-iodopropyl benzoate (**2a**, 58.0 mg,

0.20 mmol), purification by flash column chromatography (*n*-hexane/EtOAc 25/1) afforded **3d** (49.9 mg, 60% yield) as colorless oil. In an independent experiment, 53.1 mg (64% yield) were obtained, giving an average yield of 62%. **^{1}H NMR** (400 MHz, $CDCl_3$) δ 8.06 – 8.01 (m, 2H), 7.59 – 7.51 (m, 1H), 7.47 – 7.40 (m, 2H), 4.31 (t, *J* = 6.7 Hz, 2H), 3.98 (dd, *J* = 10.2, 5.7 Hz, 1H), 3.93 (dd, *J* = 10.2, 6.1 Hz, 1H), 2.39 – 2.30 (m, 2H), 2.25 – 2.17 (m, 2H), 1.86 – 1.77 (m, 2H), 1.75 – 1.64 (m, 1H), 1.67 – 1.57 (m, 2H), 1.58 – 1.47 (m, 4H), 1.45 – 1.27 (m, 16H), 0.92 (t, *J* = 7.5 Hz, 3H) ppm. **^{13}C NMR** (101 MHz, $CDCl_3$) δ 166.8, 159.7, 132.9, 130.7, 129.7, 128.5, 75.8, 65.6, 39.2, 31.0, 27.5, 26.9, 26.2, 25.4, 25.2, 24.9, 24.4, 24.1, 23.8, 23.5, 23.5, 23.1, 11.3 ppm. **IR** (neat, cm^{-1}): 2927, 2861, 1719, 1468, 1451, 1270, 1109, 1069, 1026, 710. **HRMS** (ESI+): m/z *calcd.* for ($C_{26}H_{41}NNaO_3$) $[M+Na]^+$: 438.2979; *found* 438.2975.

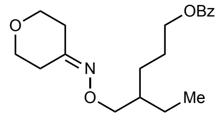

4-((((tetrahydro-4*H*-pyran-4-ylidene)amino)oxy)methyl)hexyl benzoate (3e). Following general procedure E, using tetrahydro-4*H*-pyran-4-one *O*-but-3-en-1-yl oxime (**1f**, 50.8 mg, 0.30 mmol) and 3-iodopropyl benzoate (**2a**, 58.0 mg, 0.20 mmol), purification by flash column chromatography (*n*-hexane/EtOAc 15/1) afforded **3e** (44.9 mg, 67% yield) as colorless oil. In an independent experiment, 49.2 mg (74% yield) were obtained, giving an average yield of 71%. **^{1}H NMR** (400 MHz, $CDCl_3$) δ 8.05 – 8.00 (m, 2H), 7.57 – 7.51 (m, 1H), 7.46 – 7.39 (m, 2H), 4.31 (t, *J* = 6.6 Hz, 2H), 3.99 (dd, *J* = 10.2, 5.8 Hz, 1H), 3.93 (dd, *J* = 10.2, 6.5 Hz, 1H), 3.78 (t, *J* = 5.6 Hz, 2H), 3.68 (t, *J* = 5.8 Hz, 2H), 2.58 (t, *J* = 5.8 Hz, 2H), 2.33 (t, *J* = 5.6 Hz, 2H), 1.86 – 1.74 (m, 2H), 1.75 – 1.67 (m, 1H), 1.56 – 1.43 (m, 2H), 1.42 – 1.31 (m, 2H), 0.91 (t, *J* = 7.5 Hz, 3H) ppm. **^{13}C NMR** (101 MHz, $CDCl_3$) δ 166.7, 154.8, 132.9, 130.6, 129.6, 128.4, 76.2, 68.5, 66.9, 65.4, 38.8, 32.5, 27.4, 26.9, 26.2, 24.0, 11.2 ppm. **IR** (neat, cm^{-1}): 2959, 2929, 2856, 1717, 1452, 1270, 1229, 1098, 1046, 1000, 939, 848, 710. **HRMS** (ESI+): m/z *calcd.* for ($C_{19}H_{27}NNaO_4$) $[M+Na]^+$: 356.1832; *found* 356.1834.

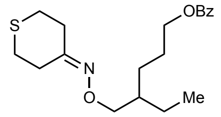

4-((((tetrahydro-4*H*-thiopyran-4-ylidene)amino)oxy)methyl)hexyl benzoate (3f). Following general procedure E, tetrahydro-4*H*-thiopyran-4-one O-but-3-en-1-yl oxime (**1g**, 55.6 mg, 0.30 mmol) and 3-iodopropyl benzoate (**2a**, 58.0 mg, 0.20 mmol), purification by flash column chromatography (*n*-hexane/EtOAc 20/1) afforded **3f** (48.2 mg, 69% yield) as colorless oil. In an independent experiment, 46.1 mg (66% yield) were obtained, giving an average yield of 68%. **^{1}H NMR** (400 MHz, $CDCl_3$) δ 8.07 – 8.00 (m, 2H), 7.58 – 7.53 (m, 1H), 7.48 – 7.38 (m, 2H), 4.31 (t, *J* = 6.6 Hz, 2H), 3.98 (dd, *J* = 10.2, 5.8 Hz, 1H), 3.92 (dd, *J* = 10.2, 6.4 Hz, 1H), 2.83 – 2.73 (m, 4H), 2.72 – 2.60 (m, 2H), 2.56 – 2.48 (m, 2H), 1.86 – 1.74 (m, 2H), 1.73 – 1.66 (m, 1H), 1.55 – 1.34 (m, 4H), 0.91 (t, *J* = 7.5 Hz, 3H) ppm. **^{13}C NMR** (101 MHz, $CDCl_3$) δ 166.7, 156.9, 133.0, 130.6, 129.6, 128.5, 76.3, 65.4, 38.9, 34.0, 29.9, 28.5, 27.6, 27.4, 26.2, 24.0, 11.2 ppm. **IR** (neat, cm^{-1}): 2956, 2918, 2874, 1716, 1451, 1269, 1109, 1069, 1045, 1026, 936, 709. **HRMS** (ESI+): m/z *calcd.* for ($C_{19}H_{27}NNaO_3S$) $[M+Na]^+$: 372.1604; *found* 372.1601.

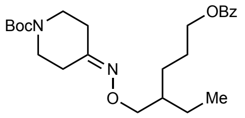

***tert*-butyl 4-(((5-(benzoyloxy)-2-ethylpentyl)oxy)imino)piperidine-1-carboxylate (3g).** Following general procedure E, *tert*-butyl 4-((but-3-en-1-yloxy)imino)piperidine-1-carboxylate (**1h**, 80.5mg, 0.30 mmol) and 3-iodopropyl benzoate (**2a**, 58.0 mg, 0.20 mmol), purification by flash column chromatography (*n*-hexane/EtOAc 10/1) afforded **3g** (61.4 mg, 72% yield) as colorless oil. In an independent experiment, 58.7 mg (68% yield) were obtained, giving an average yield of 70%. **^{1}H NMR** (400 MHz, $CDCl_3$) δ 8.06 – 8.00 (m, 2H), 7.59 – 7.53 (m, 1H), 7.46 – 7.40 (m, 2H), 4.31 (t, *J* = 6.6 Hz, 2H), 3.99 (dd, *J* = 10.2, 5.8 Hz, 1H), 3.93 (dd, *J* = 10.2, 6.4 Hz, 1H), 3.52 (t, *J* = 6.1 Hz, 2H), 3.46 (t, *J* = 6.1 Hz, 2H), 2.54 (t, *J* = 6.1 Hz, 2H), 2.34 – 2.27 (m, 2H), 1.86 – 1.74 (m, 2H), 1.74 – 1.66 (m, 2H), 1.55 – 1.48 (m, 1H), 1.46 (s, 9H), 1.44 – 1.34 (m, 2H), 0.91 (t, *J* = 7.5 Hz, 3H) ppm. **^{13}C NMR** (101 MHz, $CDCl_3$) δ 166.8, 155.9, 154.8, 133.0, 130.6, 129.6, 128.5, 80.1, 76.3, 65.4, 38.9, 31.1, 28.5, 27.4, 26.2, 25.6, 24.0, 11.2 ppm. **IR** (neat, cm^{-1}): 2961, 2926, 2874, 1718, 1694, 1419, 1271, 1237, 1165, 1111, 1047, 990, 711. **HRMS** (ESI+): m/z *calcd.* for ($C_{24}H_{36}N_2NaO_5$) [M+Na]$^+$: 455.2516; *found* 455.2513.

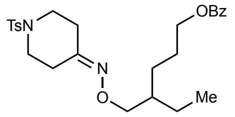

4-((((1-tosylpiperidin-4-ylidene)amino)oxy)methyl)hexyl benzoate (3h). Following general procedure E, using 1-tosylpiperidin-4-one *O*-but-3-en-1-yl oxime (**1i**, 96.7 mg, 0.30 mmol) and 3-iodopropyl benzoate (**2a**, 58.0 mg, 0.20 mmol) with (*S*)-4-(*tert*-butyl)-2-(5,6,7,8-tetrahydronaphthalen-2-yl)-4,5-dihydrooxazole (20 mol%, 0.040 mmol), purification by flash column chromatography (*n*-hexane/EtOAc 10/1) afforded **3h** (66.2 mg, 68% yield) as colorless oil. In an independent experiment, 62.3 mg (64% yield) were obtained, giving an average yield of 66%. **^{1}H NMR** (400 MHz, $CDCl_3$) δ 8.05 – 7.99 (m, 2H), 7.65 – 7.60 (m, 2H), 7.60 – 7.53 (m, 1H), 7.48 – 7.39 (m, 2H), 7.35 – 7.27 (m, 2H), 4.28 (t, *J* = 6.6 Hz, 2H), 3.93 (dd, *J* = 10.1, 5.8 Hz, 1H), 3.87 (dd, *J* = 10.2, 6.4 Hz, 1H), 3.16 (t, *J* = 5.9 Hz, 2H), 3.07 (t, *J* = 6.1 Hz, 2H), 2.63 (t, *J* = 6.1 Hz, 2H), 2.42 (s, 3H), 2.41 – 2.37 (m, 2H), 1.82 – 1.70 (m, 2H), 1.70 – 1.60 (m, 2H), 1.48 – 1.40 (m, 1H), 1.38 – 1.30 (m, 2H), 0.88 (t, *J* = 7.5 Hz, 3H) ppm. **^{13}C NMR** (101 MHz, $CDCl_3$) δ 166.7, 153.8, 143.9, 133.4, 133.0, 130.6, 129.9, 129.6, 128.5, 127.7, 76.4, 65.3, 46.7, 45.3, 38.8, 31.1, 27.3, 26.1, 24.9, 23.9, 21.6, 11.2 ppm. **IR** (neat, cm^{-1}): 2959, 2925, 2873, 1715, 1452, 1361, 1341, 1271, 1163, 1104, 1035, 917, 712, 646, 548. **HRMS** (ESI+): m/z *calcd.* for ($C_{26}H_{34}N_2NaO_5S$) [M+Na]$^+$: 509.2081; *found* 509.2089. The enantiomeric excess of **3h** (30% ee) was determined by Supercritical Fluid Chromatography (SFC) analysis on a Chiralpak® IA-3 (100mm x 3mm x 0.3μm) column (method: 0 min – 0.5 min, 95:5 CO_2:MeOH; 0.5 min – 2.0 min, 95:5 CO_2:MeOH to 80:20 CO_2:MeOH; 2.0 min – 3.0 min, 80:20 CO_2:MeOH; 3.0 min – 5.0 min, 80:20 CO_2:MeOH to 60:40. Column temperature 35 °C, flow rate 1.3 mL/min) with retention time 3.77 min (major) and 4.03 min (minor).

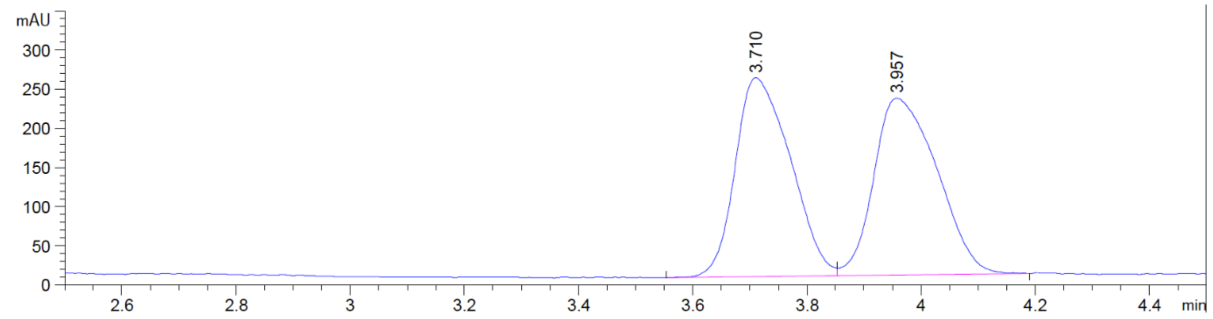

Peak #	RetTime [min]	Type	Width [min]	Area [mAU*s]	Height [mAU]	Area %
1	3.710	BV	0.1099	1709.32104	254.49274	49.5116
2	3.957	VV R	0.1234	1743.04175	226.29042	50.4884

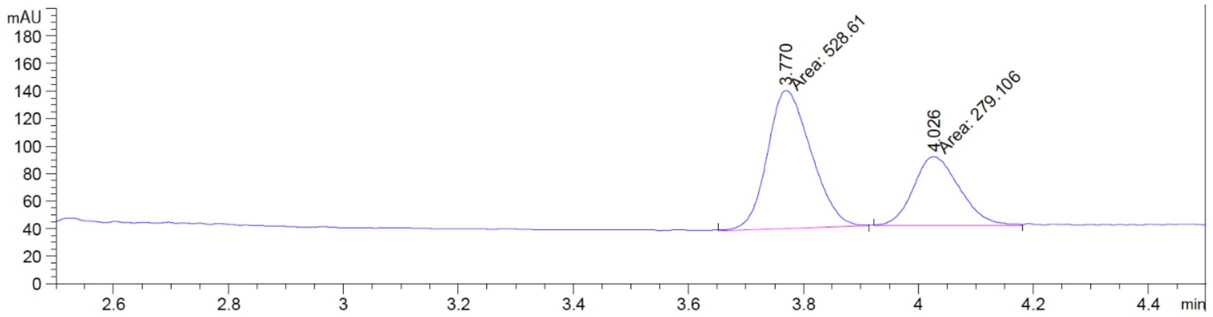

Peak #	RetTime [min]	Type	Width [min]	Area [mAU*s]	Height [mAU]	Area %
1	3.770	MM	0.0879	528.61011	100.21574	65.4450
2	4.026	MM	0.0933	279.10599	49.87029	34.5550

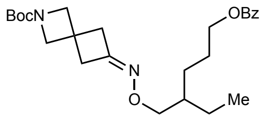

***tert*-butyl 6-(((5-(benzoyloxy)-2-ethylpentyl)oxy)imino)-2-azaspiro[3.3]heptane-2-carboxylate (3i).** Following general procedure E, using *tert*-butyl 6-((but-3-en-1-yloxy)imino)-2-azaspiro[3.3]heptane-2-carboxylate (**1k**, 84.1 mg, 0.30 mmol) and 3-iodopropyl benzoate (**2a**, 58.0 mg, 0.20 mmol), purification by flash column chromatography (*n*-hexane/EtOAc 10/1) afforded **3i** (64.0 mg, 72% yield) as colorless oil. In an independent experiment, 65.8 mg (74% yield) were obtained, giving an average yield of 73%. ^{1}**H NMR** (400 MHz, $CDCl_3$) δ 8.06 – 8.01 (m, 2H), 7.57 – 7.52 (m, 1H), 7.47 – 7.41 (m, 2H), 4.31 (t, *J* = 6.6 Hz, 2H), 4.00 – 3.93 (m, 5H), 3.90 (dd, *J* = 10.4, 6.6 Hz, 1H), 3.09 – 3.03 (m, 2H), 3.05 – 2.99 (m, 2H), 1.85 – 1.73 (m, 2H), 1.75 – 1.63 (m, 2H), 1.52 – 1.45 (m, 1H), 1.43 (s, 9H), 1.42 – 1.33 (m, 2H), 0.91 (t, *J* = 7.5 Hz, 3H) ppm. ^{13}**C NMR** (101 MHz, $CDCl_3$) δ 166.8, 156.2, 152.2, 133.0, 130.6, 129.7, 128.5, 79.8, 76.8, 65.4, 61.1, 43.0, 42.2, 38.8, 32.0, 28.5, 27.3, 26.2, 23.8, 11.2 ppm. **IR** (neat, cm^{-1}): 2958, 2930, 2873, 1700, 1391, 1271, 1157, 1098, 1027, 711. **HRMS** (ESI+): m/z *calcd.* for ($C_{25}H_{36}N_2NaO_5$) $[M+Na]^+$: 467.2516; *found* 467.2528.

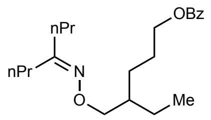

4-(((heptan-4-ylideneamino)oxy)methyl)hexyl benzoate (3j). Following general procedure E, using heptan-4-one *O*-but-3-en-1-yl oxime (**1j**, 55.0 mg, 0.30 mmol) and 3-iodopropyl benzoate (**2a**, 58.0 mg, 0.20 mmol), purification by flash column chromatography (*n*-hexane/EtOAc 30/1) afforded **3j** (43.8 mg, 63% yield) as colorless oil. In an independent experiment, 49.3 mg (71% yield) were obtained, giving an average yield of 67%. **^{1}H NMR** (400 MHz, $CDCl_3$) δ 8.07 – 8.01 (m, 2H), 7.58 – 7.51 (m, 1H), 7.47 – 7.39 (m, 2H), 4.31 (t, *J* = 6.6 Hz, 2H), 3.97 (dd, *J* = 10.1, 5.7 Hz, 1H), 3.92 (dd, *J* = 10.1, 6.3 Hz, 1H), 2.28 – 2.20 (m, 2H), 2.14 – 2.08 (m, 2H), 1.87 – 1.77 (m, 2H), 1.76 – 1.65 (m, 1H), 1.59 – 1.35 (m, 8H), 0.95 – 0.88 (m, 9H) ppm. **^{13}C NMR** (101 MHz, $CDCl_3$) δ 166.8, 161.0, 132.9, 130.7, 129.7, 128.5, 75.9, 65.6, 39.0, 36.3, 30.2, 27.5, 26.2, 24.1, 20.2, 19.5, 14.5, 14.0, 11.3 ppm. **IR** (neat, cm^{-1}): 2960, 2931, 2872, 1720, 1452, 1314, 1271, 1110, 1069, 1050, 1026, 938, 710. **HRMS** (ESI+): m/z *calcd.* for ($C_{21}H_{33}NNaO_3$) $[M+Na]^+$: 370.2353; *found* 370.2354.

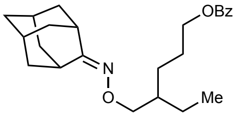

4-(((((1*r*,3*r*,5*R*,7*S*)-adamantan-2-ylidene)amino)oxy)methyl)hexyl benzoate (3k). Following general procedure E, using (1*r*,3*r*,5*R*,7*S*)-adamantan-2-one *O*-but-3-en-1-yl oxime (**1l**, 65.8 mg, 0.30 mmol) and 3-iodopropyl benzoate (**2a**, 58.0 mg, 0.20 mmol), purification by flash column chromatography (*n*-hexane/EtOAc 25/1) afforded **3k** (46.0 mg, 60% yield) as colorless oil. In an independent experiment, 48.3 mg (63% yield) were obtained, giving an average yield of 62%. **^{1}H NMR** (400 MHz, $CDCl_3$) δ 8.07 – 8.01 (m, 2H), 7.57 – 7.50 (m, 1H), 7.47 – 7.38 (m, 2H), 4.30 (t, *J* = 6.6 Hz, 2H), 3.98 (dd, *J* = 10.3, 5.8 Hz, 1H), 3.90 (dd, *J* = 10.3, 6.6 Hz, 1H), 3.48 (t, *J* = 3.7 Hz, 1H), 2.52 (s, 1H), 1.99 – 1.89 (m, 4H), 1.89 – 1.80 (m, 7H), 1.80 – 1.69 (m, 4H), 1.57 – 1.32 (m, 4H), 0.92 (t, *J* = 7.5 Hz, 3H) ppm. **^{13}C NMR** (101 MHz, $CDCl_3$) δ 166.8, 166.6, 132.9, 130.7, 129.7, 128.4, 76.0, 65.6, 39.2, 38.9, 37.8, 36.7, 36.4, 29.8, 28.0, 27.6, 26.2, 24.1, 11.3 ppm. **IR** (neat, cm^{-1}): 2913, 2852, 1718, 1450, 1270, 1109, 1044, 1026, 915, 894, 710. **HRMS** (ESI+): m/z *calcd.* for ($C_{24}H_{33}NNaO_3$) $[M+Na]^+$: 406.2353; *found* 406.2355.

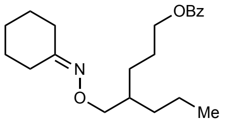

4-(((cyclohexylideneamino)oxy)methyl)heptyl benzoate (3l). Following general procedure E, using cyclohexanone *O*-pent-4-en-1-yl oxime (**1m**, 54.4 mg, 0.30 mmol) and 3-iodopropyl benzoate (**2a**, 58.0 mg, 0.20 mmol), purification by flash column chromatography (*n*-hexane/EtOAc 25/1) afforded **3l** (54.0 mg, 78% yield) as colorless oil. In an independent experiment, 55.9 mg (81% yield) were obtained, giving an average yield of 80%. **^{1}H NMR** (400 MHz, $CDCl_3$) δ 8.07 – 8.01 (m, 2H), 7.58 – 7.52 (m, 1H), 7.47 – 7.40 (m, 2H), 4.31 (t, *J* = 6.6 Hz, 2H), 3.97 (dd, *J* = 10.1, 5.6 Hz, 1H), 3.90 (dd, *J* = 10.1, 6.6 Hz, 1H), 2.48 – 2.40 (m, 2H), 2.23 – 2.14 (m, 2H), 1.86 – 1.74 (m, 3H), 1.70 – 1.60 (m, 2H), 1.61 – 1.42 (m, 6H), 1.39 – 1.27 (m, 4H), 0.90

(t, J = 6.9 Hz, 3H) ppm. **^{13}C NMR** (101 MHz, $CDCl_3$) δ 166.8, 160.1, 132.9, 130.7, 129.7, 128.4, 76.4, 65.5, 37.2, 33.9, 32.4, 28.1, 27.2, 26.2, 26.0, 25.9, 25.5, 20.1, 14.6 ppm. **IR** (neat, cm^{-1}): 2928, 2858, 1719, 1451, 1314, 1272, 1110, 1070, 1047, 1027, 711. **HRMS** (ESI+): m/z *calcd.* for ($C_{21}H_{32}NO_3$) $[M+H]^+$: 346.2377; *found* 346.2381.

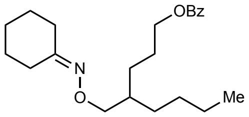

4-(((cyclohexylideneamino)oxy)methyl)octyl benzoate (3m). Following general procedure E, using cyclohexanone *O*-hex-5-en-1-yl oxime (**1n**, 58.6 mg, 0.30 mmol) and 3-iodopropyl benzoate (**2a**, 58.0 mg, 0.20 mmol), purification by flash column chromatography (*n*-hexane/EtOAc 25/1) afforded **3m** (51.7 mg, 72% yield) as colorless oil. In an independent experiment, 53.2 mg (74% yield) were obtained, giving an average yield of 73%. Following general procedure E, using (*E*)-cyclohexanone *O*-hex-3-en-1-yl oxime (**1r**, 58.6 mg, 0.30 mmol) and 3-iodopropyl benzoate (**2a**, 58.0 mg, 0.20 mmol), purification by flash column chromatography (*n*-hexane/EtOAc 25/1) afforded **3m** (49.1 mg, 68% yield) as colorless oil. In an independent experiment, 51.6 mg (72% yield) were obtained, giving an average yield of 70%. Following general procedure E, using (*Z*)-cyclohexanone *O*-hex-3-en-1-yl oxime (**1s,** 58.6 mg, 0.30 mmol) and 3-iodopropyl benzoate (**2a**, 58.0 mg, 0.20 mmol), purification by flash column chromatography (*n*-hexane/EtOAc 25/1) afforded **3m** (49.0 mg, 69% yield) as colorless oil. In an independent experiment, 54.2 mg (75% yield) were obtained, giving an average yield of 72%. Following general procedure E, using (*E*)-cyclohexanone *O*-hex-4-en-1-yl oxime (**1t**, 58.6 mg, 0.30 mmol) and 3-iodopropyl benzoate (**2a**, 58.0 mg, 0.20 mmol), purification by flash column chromatography (*n*-hexane/EtOAc 25/1) afforded **3m** (46.8 mg, 65% yield) as colorless oil. In an independent experiment, 49.3 mg (69% yield) were obtained, giving an average yield of 67%. Following general procedure E, using (*Z*)-cyclohexanone *O*-hex-4-en-1-yl oxime (**1u**, 58.6 mg, 0.30 mmol) and 3-iodopropyl benzoate (**2a**, 58.0 mg, 0.20 mmol), purification by flash column chromatography (*n*-hexane/EtOAc 25/1) afforded **3m** (43.8 mg, 61% yield) as colorless oil. In an independent experiment, 47.4 mg (66% yield) were obtained, giving an average yield of 63%. **^{1}H NMR** (400 MHz, $CDCl_3$) δ 8.07 – 8.00 (m, 2H), 7.57 – 7.51 (m, 1H), 7.47 – 7.38 (m, 2H), 4.31 (t, J = 6.6 Hz, 2H), 3.97 (dd, J = 10.1, 5.7 Hz, 1H), 3.90 (dd, J = 10.1, 6.6 Hz, 1H), 2.49 – 2.39 (m, 2H), 2.21 – 2.10 (m, 2H), 1.87 – 1.71 (m, 3H), 1.70 – 1.60 (m, 2H), 1.61 – 1.52 (m, 4H), 1.55 – 1.39 (m, 2H), 1.39 – 1.23 (m, 6H), 0.88 (t, J = 6.9 Hz, 3H) ppm. **^{13}C NMR** (101 MHz, $CDCl_3$) δ 166.8, 160.1, 132.9, 130.6, 129.6, 128.4, 76.4, 65.5, 37.4, 32.3, 31.2, 29.1, 28.0, 27.2, 26.2, 26.0, 25.9, 25.4, 23.2, 14.2 ppm. **IR** (neat, cm^{-1}): 2925, 2856, 1719, 1450, 1271, 1109, 1069, 1046, 1027, 935, 710. **HRMS** (ESI+): m/z *calcd.* for ($C_{22}H_{34}NO_3$) $[M+H]^+$: 360.2533; *found* 360.2531.

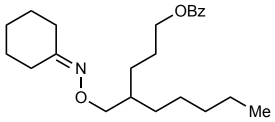

4-(((cyclohexylideneamino)oxy)methyl)nonyl benzoate (3n). Following general procedure E, using cyclohexanone *O*-hept-6-en-1-yl oxime (**1o**, 62.8 mg, 0.30 mmol) and 3-iodopropyl benzoate (**2a**, 58.0 mg, 0.20

mmol), purification by flash column chromatography (*n*-hexane/EtOAc 25/1) afforded **3n** (50.2 mg, 67% yield) as colorless oil. In an independent experiment, 54.2 mg (73% yield) were obtained, giving an average yield of 70%. **^{1}H NMR** (400 MHz, $CDCl_3$) δ 8.07 – 8.01 (m, 2H), 7.59 – 7.51 (m, 1H), 7.47 – 7.39 (m, 2H), 4.31 (t, *J* = 6.6 Hz, 2H), 3.97 (dd, *J* = 10.1, 5.6 Hz, 1H), 3.90 (dd, *J* = 10.1, 6.6 Hz, 1H), 2.48 – 2.39 (m, 2H), 2.23 – 2.13 (m, 2H), 1.87 – 1.73 (m, 3H), 1.69 – 1.61 (m, 2H), 1.60 – 1.54 (m, 4H), 1.54 – 1.40 (m, 2H), 1.37 – 1.23 (m, 8H), 0.88 (t, *J* = 6.9 Hz, 3H) ppm. **^{13}C NMR** (101 MHz, $CDCl_3$) δ 166.8, 160.1, 132.9, 130.7, 129.7, 128.5, 76.4, 65.5, 37.5, 32.4, 31.5, 28.0, 27.2, 26.6, 26.2, 26.0, 25.9, 25.5, 22.8, 14.2 ppm. **IR** (neat, cm^{-1}): 2926, 2856, 1719, 1451, 1314, 1271, 1110, 1069, 1048, 1027, 710. **HRMS** (ESI+): m/z *calcd.* for ($C_{23}H_{36}NO_3$) $[M+H]^+$: 374.2690; *found* 374.2705.

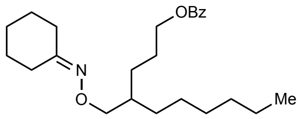

4-(((cyclohexylideneamino)oxy)methyl)decyl benzoate (3o). Following general procedure E, using cyclohexanone *O*-oct-7-en-1-yl oxime (**1p**, 67.0mg, 0.30 mmol) and 3-iodopropyl benzoate (**2a**, 58.0 mg, 0.20 mmol), purification by flash column chromatography (*n*-hexane/EtOAc 30/1) afforded **3o** (55.8 mg, 72% yield) as colorless oil. In an independent experiment, 52.7 mg (68% yield) were obtained, giving an average yield of 70%. **^{1}H NMR** (400 MHz, $CDCl_3$) δ 8.07 – 8.00 (m, 2H), 7.59 – 7.50 (m, 1H), 7.45-7.39 (m, 2H), 4.31 (t, *J* = 6.6 Hz, 2H), 3.96 (dd, *J* = 10.1, 5.6 Hz, 1H), 3.90 (dd, *J* = 10.1, 6.6 Hz, 1H), 2.47 – 2.39 (m, 2H), 2.21 – 2.14 (m, 2H), 1.87 – 1.73 (m, 3H), 1.70 – 1.60 (m, 2H), 1.60 – 1.52 (m, 4H), 1.55 – 1.37 (m, 2H), 1.37 – 1.21 (m, 10H), 0.86 (t, *J* = 6.9 Hz, 3H) ppm. **^{13}C NMR** (101 MHz, $CDCl_3$) δ 166.8, 160.1, 132.9, 130.6, 129.6, 128.4, 76.4, 65.5, 37.4, 32.3, 31.9, 31.5, 29.8, 28.0, 27.2, 26.9, 26.1, 26.0, 25.9, 25.4, 22.8, 14.2 ppm. **IR** (neat, cm^{-1}): 2925, 2856, 1719, 1450, 1314, 1270, 1109, 1069, 1044, 1026, 709. **HRMS** (ESI+): m/z *calcd.* for ($C_{24}H_{38}NO_3$) $[M+H]^+$: 388.2846; *found* 388.2857.

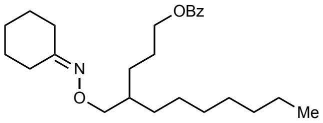

4-(((cyclohexylideneamino)oxy)methyl)undecyl benzoate (3p). Following general procedure E, using cyclohexanone *O*-non-8-en-1-yl oxime (**1q**, 71.2 mg, 0.30 mmol) and 3-iodopropyl benzoate (**2a**, 58.0 mg, 0.20 mmol), purification by flash column chromatography (*n*-hexane/EtOAc 30/1) afforded **3p** (38.6 mg, 48% yield) as colorless oil. In an independent experiment, 41.8 mg (52% yield) were obtained, giving an average yield of 50%. **^{1}H NMR** (400 MHz, $CDCl_3$) δ 8.06 – 8.01 (m, 2H), 7.58 – 7.52 (m, 1H), 7.46 – 7.40 (m, 2H), 4.31 (t, *J* = 6.6 Hz, 2H), 3.96 (dd, *J* = 10.1, 5.6 Hz, 1H), 3.90 (dd, *J* = 10.1, 6.6 Hz, 1H), 2.48 – 2.40 (m, 2H), 2.23 – 2.11 (m, 2H), 1.88 – 1.71 (m, 3H), 1.69 – 1.61 (m, 4H), 1.62 – 1.52 (m, 4H), 1.53 – 1.39 (m, 2H), 1.35 – 1.29 (m, 4H), 1.30 – 1.23 (m, 6H), 0.87 (t, *J* = 6.9 Hz, 3H) ppm. **^{13}C NMR** (101 MHz, $CDCl_3$) δ 166.8, 160.2, 132.9, 130.7, 129.7, 128.4, 76.4, 65.5, 37.4, 32.4, 32.0, 31.5, 30.1, 29.4, 28.0, 27.2, 26.9, 26.2, 26.0, 25.9, 25.5, 22.8, 14.2 ppm. **IR** (neat, cm^{-1}): 2924, 2854, 1719, 1451, 1314, 1270, 1110, 1069, 1045, 1026, 936, 709. **HRMS** (ESI+): m/z *calcd.* for ($C_{25}H_{40}NO_3$) $[M+H]^+$: 402.3003; *found* 402.3018.

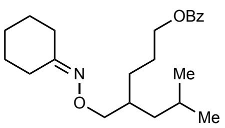

4-(((cyclohexylideneamino)oxy)methyl)-6-methylheptyl benzoate (3q). Following general procedure E, using cyclohexanone *O*-(4-methylpent-3-en-1-yl) oxime (**1v**, 58.6 mg, 0.30 mmol) and 3-iodopropyl benzoate (**2a**, 58.0 mg, 0.20 mmol). ^{1}H NMR and GC-FID analysis of the crude mixture showed the rr was 9:1 (rr refers to the ratio of the major regioisomer to all other regioisomers). Purification by flash column chromatography (*n*-hexane/EtOAc 25/1) afforded **3q** (29.1 mg, 41% yield) as a light-yellow oil. In an independent experiment, 33.1 mg (46% yield) were obtained, giving an average yield of 43%. **^{1}H NMR** (400 MHz, $CDCl_3$) δ 8.07 – 8.00 (m, 2H), 7.58 – 7.51 (m, 1H), 7.48 – 7.39 (m, 2H), 4.31 (t, *J* = 6.6 Hz, 2H), 3.95 (dd, *J* = 10.1, 5.4 Hz, 1H), 3.89 (dd, *J* = 10.1, 6.6 Hz, 1H), 2.50 – 2.36 (m, 2H), 2.21 – 2.15 (m, 2H), 1.89 – 1.77 (m, 3H), 1.73 – 1.61 (m, 4H), 1.61 – 1.53 (m, 3H), 1.54 – 1.37 (m, 2H), 1.27 – 1.09 (m, 2H), 0.88 (t, *J* = 6.5 Hz, 6H) ppm. **^{13}C NMR** (101 MHz, $CDCl_3$) δ 166.8, 160.1, 132.9, 130.7, 129.7, 128.4, 76.6, 65.5, 41.4, 35.2, 32.4, 28.5, 27.2, 26.1, 26.0, 25.9, 25.6, 25.5, 23.2, 23.0 ppm. **IR** (neat, cm^{-1}): 2928, 2660, 1720, 1450, 1272, 1109, 1069, 1047, 1026, 933, 711. **HRMS** (ESI+): m/z *calcd.* for ($C_{22}H_{33}NNaO_3$) $[M+Na]^+$: 382.2353; *found* 382.2352.

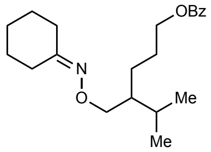

4-(((cyclohexylideneamino)oxy)methyl)-5-methylhexyl benzoate (3r). Following general procedure E, using cyclohexanone *O*-(3-methylbut-3-en-1-yl) oxime (**1w**, 54.4 mg, 0.30 mmol) and 3-iodopropyl benzoate (**2a**, 58.0 mg, 0.20 mmol), purification by flash column chromatography (*n*-hexane/EtOAc 25/1) afforded **3r** (34.2 mg, 50% yield) as colorless oil. In an independent experiment, 37.8 mg (55% yield) were obtained, giving an average yield of 52%. **^{1}H NMR** (400 MHz, $CDCl_3$) δ 8.10 – 7.97 (m, 2H), 7.59 – 7.52 (m, 1H), 7.45 – 7.41 (m, 2H), 4.31 (t, *J* = 6.6 Hz, 2H), 4.05 (dd, *J* = 10.1, 5.6 Hz, 1H), 3.94 (dd, *J* = 10.1, 6.6 Hz, 1H), 2.48 – 2.39 (m, 2H), 2.30 – 2.12 (m, 2H), 1.92 – 1.73 (m, 3H), 1.72 – 1.42 (m, 9H), 0.91 (t, *J* = 6.5 Hz, 6H) ppm. **^{13}C NMR** (101 MHz, $CDCl_3$) δ 166.8, 160.1, 132.9, 130.7, 129.7, 128.4, 74.7, 65.5, 43.1, 32.3, 28.9, 27.2, 27.0, 26.0, 25.9, 25.5, 25.3, 19.9, 19.5 ppm. **IR** (neat, cm^{-1}): 2930, 2863, 1718, 1450, 1270, 1109, 1026, 709. **HRMS** (ESI+): m/z *calcd.* for ($C_{21}H_{31}NNaO_3$) $[M+Na]^+$: 368.2196; *found* 368.2198.

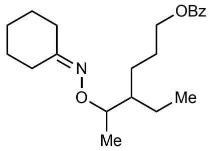

5-((cyclohexylideneamino)oxy)-4-ethylhexyl benzoate (3s). Following general procedure E, using cyclohexanone *O*-pent-4-en-2-yl oxime (**1x**, 54.4 mg, 0.30 mmol) and 3-iodopropyl benzoate (**2a**, 58.0 mg, 0.20 mmol), purification by flash column chromatography (*n*-hexane/EtOAc 25/1) afforded **3s** (48.6 mg, 70% yield, dr 1:1) as colorless oil. In an independent experiment, 51.9 mg (75% yield) were obtained, giving an average yield of 73%. **^{1}H NMR** (400 MHz, $CDCl_3$) δ 8.06 – 8.01 (m, 2H), 7.59 – 7.51 (m, 1H), 7.46 – 7.41 (m, 2H),

4.35 – 4.26 (m, 2H), 4.25 – 4.18 (m, 0.5H), 4.18 – 4.11 (m, 0.5H), 2.49 – 2.35 (m, 2H), 2.22 – 2.12 (m, 2H), 1.95 – 1.73 (m, 2H), 1.70 – 1.61 (m, 3H), 1.59 – 1.54 (m, 2H), 1.54 – 1.41 (m, 3H), 1.40 – 1.20 (m, 3H), 1.17 (d, J = 6.4 Hz, 1.5H), 1.15 (d, J = 6.5 Hz, 1.5H), 0.98 – 0.86 (m, 3H) ppm. **^{13}C NMR** (101 MHz, $CDCl_3$) δ 166.8, 159.6, 132.9, 130.7 (130,7), 129,7 (129.7), 128.5, 80.1 (79.9), 65.6 (65.6), 43.5 (43.2), 32.5 (32.4), 27.3 (27.3), 27.0 (26.8), 26.4 (26.1), 26.0 (26.0), 25.9 (25.6), 25.6 (25.5), 23.6 (22.3), 16.6 (15.5), 12.1 (11.9) ppm. **IR** (neat, cm^{-1}): 2930, 2859, 1719, 1450, 1314, 1272, 1110, 948, 711. **HRMS** (ESI+): m/z *calcd.* for ($C_{21}H_{31}NNaO_3$) $[M+Na]^+$: 368.2196; *found* 368.2190.

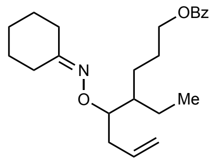

5-((cyclohexylideneamino)oxy)-4-ethyloct-7-en-1-yl benzoate (3t). Following general procedure E, using cyclohexanone *O*-hepta-1,6-dien-4-yl oxime (**1z**, 62.2 mg, 0.30 mmol) and 3-iodopropyl benzoate (**2a**, 58.0 mg, 0.20 mmol), purification by flash column chromatography (*n*-hexane/EtOAc 30/1) afforded **3t** (29.9 mg, 40% yield, dr 1:1) as colorless oil. In an independent experiment, 32.2 mg (44% yield) were obtained, giving an average yield of 42%. **^{1}H NMR** (400 MHz, $CDCl_3$) δ 8.06 – 8.01 (m, 2H), 7.58 – 7.52 (m, 1H), 7.46 – 7.40 (m, 2H), 5.92 – 5.75 (m, 1H), 5.09 – 5.02 (m, 1H), 5.02 – 4.98 (m, 1H), 4.30 (t, J = 6.6 Hz, 1.5H), 4.29 (t, J = 6.7 Hz, 1.5H), 4.17 – 4.05 (m, 1H), 2.47 – 2.25 (m, 4H), 2.17 (t, J = 6.2 Hz, 2H), 1.91 – 1.72 (m, 2H), 1.69 – 1.62 (m, 2H), 1.59 – 1.49 (m, 5H), 1.49 – 1.28 (m, 4H), 0.93 (t, J = 7.5 Hz, 1.5H), 0.91 (t, J = 7.4 Hz, 1.5H) ppm. **^{13}C NMR** (101 MHz, $CDCl_3$) δ 166.8, 159.8 (159.7), 135.9 (135.7), 132.9, 130.7, 129.7, 128.5, 116.5 (116.4), 83.4 (83.2), 65.6 (65.5), 41.5 (41.4), 35.7 (35.2), 32.4, 27.3 (27.3), 26.8 (26.8), 26.0, 26.0 (25.9), 25.9 (25.7), 25.6, 22.7 (22.6), 12.0 (11.7) ppm. **IR** (neat, cm^{-1}): 3072, 2930, 2859, 1719, 1450, 1314, 1270, 1110, 1026, 939, 710. **HRMS** (ESI+): m/z *calcd.* for ($C_{23}H_{34}NO_3$) $[M+H]^+$: 372.2533; *found* 372.2543.

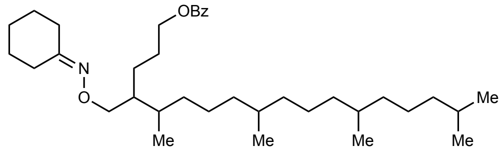

4-(((cyclohexylideneamino)oxy)methyl)-5,9,13,17-tetramethyloctadecyl benzoate (3u). Following general procedure E, using (*E*)-cyclohexanone *O*-(3,7,11,15-tetramethylhexadec-2-en-1-yl) oxime (**1aa**, 117.5 mg, 0.30 mmol) and 1-iodododecane (**2a**, 58.0 mg, 0.20 mmol), purification by flash column chromatography (*n*-hexane/EtOAc 50/1) afforded **3u** (54.4 mg, 49% yield) as colorless oil. In an independent experiment, 51.1 mg (46% yield) were obtained, giving an average yield of 47%. **^{1}H NMR** (400 MHz, $CDCl_3$) δ 8.06 – 8.02 (m, 2H), 7.55 (ddt, J = 8.0, 6.8, 1.4 Hz, 1H), 7.47 – 7.40 (m, 2H), 4.31 (t, J = 6.6 Hz, 2H), 4.07 (dd, J = 10.0, 5.4 Hz, 1H), 3.91 (dd, J = 10.0, 7.0 Hz, 1H), 2.47 – 2.39 (m, 2H), 2.24 – 2.13 (m, 2H), 1.88 – 1.76 (m, 2H), 1.70 – 1.61 (m, 2H), 1.62 – 1.53 (m, 4H), 1.54 – 1.44 (m, 3H), 1.40 – 1.30 (m, 4H), 1.31 – 1.20 (m, 10H), 1.19 – 1.10 (m, 4H), 1.11 – 0.98 (m, 4H), 0.88 – 0.82 (m, 15H) ppm. **^{13}C NMR** (101 MHz, $CDCl_3$) δ 166.8, 160.3, 132.9, 130.7, 129.7, 128.5, 74.6, 65.5, 41.8, 41.6, 39.5, 37.7, 37.6, 37.6, 37.5, 37.4, 34.9, 34.9, 34.3, 34.2, 33.0, 32.9, 32.3, 28.1, 27.2, 27.2, 26.3, 26.3, 26.0, 25.9, 25.6, 25.3, 24.9, 24.6, 22.9, 22.8, 19.9, 19.9, 19.8, 16.0, 16.0 ppm. **IR**

(neat, cm^{-1}): 2925, 2858, 1722, 1452, 1379, 1314, 1273, 1112, 1027, 711. **LCMS** (ESI+): m/z *calcd.* for ($C_{36}H_{62}NO_3$) $[M+H]^+$: 556.5; *found* 555.8.

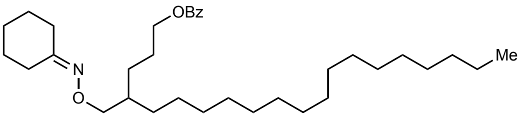

4-(((cyclohexylideneamino)oxy)methyl)icosyl benzoate (S-4). Following general procedure E, using (*Z*)-cyclohexanone *O*-octadec-9-en-1-yl oxime (**1ab**, 109.1 mg, 0.30 mmol) and 1-iodohexane (**2a**, 58.0 mg, 0.20 mmol), purification by flash column chromatography (*n*-hexane/EtOAc 50/1) afforded **S-1** (15.8 mg, 15% yield) as colorless oil. In an independent experiment, 14.7 mg (14% yield) were obtained, giving an average yield of 15%. **^{1}H NMR** (400 MHz, $CDCl_3$) δ 8.06 – 8.02 (m, 2H), 7.59 – 7.51 (m, 1H), 7.46 – 7.40 (m, 2H), 4.31 (t, J = 6.6 Hz, 2H), 3.99 (dd, J = 10.1, 5.7 Hz, 1H), 3.93 (dd, J = 10.1, 6.6 Hz, 1H), 2.47 – 2.42 (m, 2H), 2.24 – 2.18 (m, 2H), 1.85 – 1.75 (m, 4H), 1.61 – 1.54 (m, 2H), 1.54 – 1.43 (m, 2H), 1.34 – 1.29 (m, 5H), 1.28 – 1.22 (m, 28H), 0.88 (t, J = 7.0, 3H) ppm. **^{13}C NMR** (101 MHz, $CDCl_3$) δ 166.8, 161.2, 132.9, 130.7, 129.7, 128.5, 76.6, 65.5, 37.4, 32.2, 32.1, 31.5, 30.2, 29.8, 29.8, 29.8, 29.5, 28.0, 27.1, 26.9, 26.2, 25.9, 25.9, 25.6, 22.8, 14.3 ppm. **IR** (neat, cm^{-1}): 2924, 2853, 1722, 1451, 1273, 1111, 711. **LCMS** (ESI+): m/z *calcd.* for ($C_{34}H_{58}NO_3$) $[M+H]^+$: 528.4; *found* 527.9.

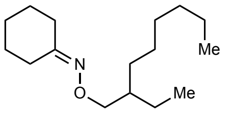

cyclohexanone *O*-(2-ethyloctyl) oxime (3v). Following general procedure E, using cyclohexanone *O*-but-3-en-1-yl oxime (**1a**, 50.2 mg, 0.30 mmol) and 1-iodohexane (**2b**, 42.4 mg, 0.20 mmol), purification by flash column chromatography (*n*-hexane/EtOAc 50/1) afforded **3u** (34.8 mg, 68% yield) as colorless oil. In an independent experiment, 36.8 mg (73% yield) were obtained, giving an average yield of 70%. **^{1}H NMR** (400 MHz, $CDCl_3$) δ 3.90 (d, *J* = 6.2 Hz, 2H), 2.49 – 2.43 (m, 2H), 2.22 – 2.16 (m, 2H), 1.71 – 1.64 (m, 2H), 1.65 – 1.54 (m, 5H), 1.42 – 1.23 (m, 12H), 0.93 – 0.84 (m, 6H) ppm. **^{13}C NMR** (101 MHz, $CDCl_3$) δ 160.0, 76.4, 39.2, 32.4, 32.0, 31.1, 29.9, 27.2, 26.9, 26.0, 25.9, 25.5, 24.1, 22.8, 14.3, 11.2 ppm. **IR** (neat, cm^{-1}): 2956, 2925, 2856, 1449, 1378, 1044, 937, 889, 839. **HRMS** (ESI+): m/z *calcd.* for ($C_{16}H_{32}NO$) $[M+H]^+$: 254.2478; *found* 254.2484.

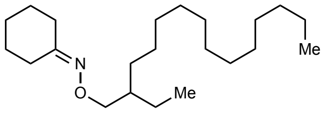

cyclohexanone *O*-(2-ethyltetradecyl) oxime (3w). Following general procedure E, using cyclohexanone *O*-but-3-en-1-yl oxime (**1a**, 50.2 mg, 0.30 mmol) and 1-iodododecane (**2c**, 59.3 mg, 0.20 mmol), purification by flash column chromatography (*n*-hexane/EtOAc 50/1) afforded **3v** (45.8 mg, 68% yield) as colorless oil. In an independent experiment, 49.5 mg (73% yield) were obtained, giving an average yield of 71%. **^{1}H NMR** (400 MHz, $CDCl_3$) δ 3.90 (d, *J* = 6.2 Hz, 2H), 2.46 (t, *J* = 6.0 Hz, 2H), 2.23 – 2.14 (m, 2H), 1.72 – 1.55 (m, 7H), 1.42 – 1.20 (m, 24H), 0.93 – 0.82 (m, 6H) ppm. **^{13}C NMR** (101 MHz, $CDCl_3$) δ 159.9, 76.4, 39.2, 32.4, 32.1, 31.1, 30.2, 30.0, 29.8, 29.8, 29.8, 29.5, 27.2, 26.9, 26.0, 26.0, 25.5, 24.1, 22.8, 14.3, 11.2 ppm. **IR** (neat, cm^{-1}):

2922, 2853, 1462, 1045, 936, 889, 836. **HRMS** (ESI+): m/z *calcd.* for ($C_{22}H_{44}NO$) $[M+H]^+$: 338.3417; *found* 358. 338.3428.

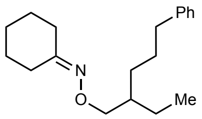

cyclohexanone *O*-(2-ethyl-5-phenylpentyl) oxime (3x). Following general procedure E, using cyclohexanone *O*-but-3-en-1-yl oxime (**1a**, 50.2 mg, 0.30 mmol) and (3-iodopropyl)benzene (**2h**, 49.2 mg, 0.20 mmol), purification byflash column chromatography (*n*-hexane/EtOAc 50/1) afforded **3w** (43.9 mg, 76% yield) as colorless oil. In an independent experiment, 47.0 mg (82% yield) were obtained, giving an average yield of 79%. **^{1}H NMR** (400 MHz, $CDCl_3$) δ 7.31 – 7.22 (m, 2H), 7.21 – 7.12 (m, 3H), 3.93 (dd, *J* = 9.1, 5.2 Hz, 1H), 3.90 (dd, *J* = 9.1, 5.3 Hz, 1H), 2.65 – 2.56 (m, 2H), 2.49 – 2.40 (m, 2H), 2.23 – 2.15 (m, 2H), 1.72 – 1.60 (m, 5H), 1.64 – 1.53 (m, 4H), 1.47 – 1.28 (m, 4H), 0.89 (t, *J* = 7.5 Hz, 3H) ppm. **^{13}C NMR** (101 MHz, $CDCl_3$) δ 160.0, 142.9, 128.5, 128.3, 125.7, 76.2, 39.1, 36.5, 32.4, 30.8, 28.8, 27.2, 26.0, 25.9, 25.5, 24.1, 11.2 ppm. **IR** (neat, cm^{-1}): 3026, 2927, 2857, 1496, 1450, 1379, 1041, 935, 889, 873, 839, 746, 697. **HRMS** (ESI+): m/z *calcd.* for ($C_{19}H_{30}NO$) $[M+H]^+$: 288.2322; *found* 288.2325.

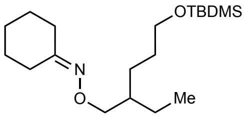

cyclohexanone *O*-(5-((*tert*-butyldimethylsilyl)oxy)-2-ethylpentyl) oxime (3y). Following general procedure E, cyclohexanone *O*-but-3-en-1-yl oxime (**1a**, 50.2 mg, 0.30 mmol) and *tert*-butyl(3-iodopropoxy)dimethylsilane (**2i**, 60.0 mg, 0.20 mmol), purification by flash column chromatography (*n*-hexane/EtOAc 50/1) afforded **3x** (49.8 mg, 73% yield) as colorless oil. In an independent experiment, 53.2 mg (78% yield) were obtained, giving an average yield of 75%. **^{1}H NMR** (400 MHz, $CDCl_3$) δ 3.95 – 3.86 (m, 2H), 3.59 (t, *J* = 6.6 Hz, 2H), 2.48 – 2.41 (m, 2H), 2.22 – 2.13 (m, 2H), 1.71 – 1.61 (m, 3H), 1.61 – 1.49 (m, 6H), 1.44 – 1.30 (m, 4H), 0.89 (t, *J* = 7.4 Hz, 3H), 0.87 (s, 9H), 0.04 (s, 6H) ppm. **^{13}C NMR** (101 MHz, $CDCl_3$) δ 159.9, 76.3, 63.8, 39.0, 32.4, 30.2, 27.2, 27.1, 26.1, 26.0, 25.9, 25.5, 24.1, 18.5, 11.2, -5.1 ppm. **IR** (neat, cm^{-1}): 2928, 2857, 1462, 1253, 1096, 1045, 937, 834, 773. **HRMS** (ESI+): m/z *calcd.* for ($C_{19}H_{40}NO_2Si$) $[M+H]^+$: 342.2823; *found* 342.2834.

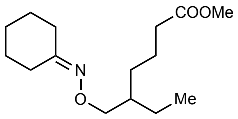

methyl 5-(((cyclohexylideneamino)oxy)methyl)heptanoate (3z). Following general procedure E, cyclohexanone *O*-but-3-en-1-yl oxime (**1a**, 50.2 mg, 0.30 mmol) and methyl 4-iodobutanoate (**2j**, 45.2 mg, 0.20 mmol), purirication by flash column chromatography (*n*-hexane/EtOAc 25/1) afforded **3y** (44.3mg, 82% yield) as colorless oil. In an independent experiment, 41.7 mg (77% yield) were obtained, giving an average yield of 80%. **^{1}H NMR** (400 MHz, $CDCl_3$) δ 3.92 (dd, *J* = 10.1, 5.9 Hz, 1H), 3.87 (dd, *J* = 10.1, 6.4 Hz, 1H), 3.65 (s, 3H), 2.47 – 2.41 (m, 2H), 2.29 (t, *J* = 7.5 Hz, 2H), 2.21 – 2.14 (m, 2H), 1.71 – 1.61 (m, 5H), 1.61 – 1.56 (m,

4H), 1.44 – 1.25 (m, 4H), 0.89 (t, J = 7.5 Hz, 3H) ppm. **^{13}C NMR** (101 MHz, $CDCl_3$) δ 174.3, 160.1, 76.0, 51.6, 38.9, 34.6, 32.3, 30.7, 27.2, 26.0, 25.9, 25.4, 23.9, 22.5, 11.2 ppm. **IR** (neat, cm^{-1}): 2929, 2859, 1739, 1440, 1436, 1376, 1243, 1199, 1167, 1105, 1046, 936, 873, 839. **HRMS** (ESI+): m/z *calcd.* for ($C_{15}H_{28}NO_3$) $[M+H]^+$: 270.2064; *found* 270.2070.

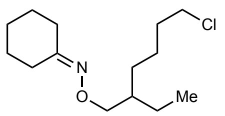

cyclohexanone *O*-(6-chloro-2-ethylhexyl) oxime (3aa). Following general procedure E, cyclohexanone *O*-but-3-en-1-yl oxime (**1a**, 50.2 mg, 0.30 mmol) and 1-chloro-4-iodobutane (**2g**, 43.7 mg, 0.20 mmol), purification by flash column chromatography (*n*-hexane/EtOAc 50/1) afforded **3z** (36.4 mg, 70% yield) as colorless oil. In an independent experiment, 40.8 mg (79% yield) were obtained, giving an average yield of 74%. **^{1}H NMR** (400 MHz, $CDCl_3$) δ 3.93 (dd, J = 10.1, 6.0 Hz, 1H), 3.88 (dd, J = 10.1, 6.4 Hz, 1H), 3.53 (t, J = 6.8 Hz, 2H), 2.48 – 2.42 (m, 2H), 2.23 – 2.15 (m, 2H), 1.82 – 1.71 (m, 2H), 1.70 – 1.64 (m, 2H), 1.63 – 1.56 (m, 5H), 1.53 – 1.39 (m, 2H), 1.41 – 1.29 (m, 4H), 0.90 (t, J = 7.5 Hz, 3H) ppm. **^{13}C NMR** (101 MHz, $CDCl_3$) δ 160.1, 76.1, 45.2, 39.0, 33.1, 32.4, 30.4, 27.2, 26.0, 26.0, 25.5, 24.3, 24.1, 11.3 ppm. **IR** (neat, cm^{-1}): 2928, 2857, 1720, 1451, 1314, 1273, 1111, 711. **HRMS** (ESI+): m/z *calcd.* for ($C_{14}H_{27}ClNO$) $[M+H]^+$: 260.1776; *found* 260.1783.

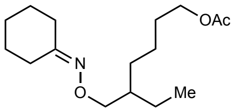

5-(((cyclohexylideneamino)oxy)methyl)heptyl acetate (3ab). Following general procedure E, using cyclohexanone *O*-but-3-en-1-yl oxime (**1a**, 50.2 mg, 0.30 mmol) and 4-iodobutyl acetate (**2d**, 48.4 mg, 0.20 mmol), purification by flash column chromatography (*n*-hexane/EtOAc 30/1) afforded **3aa** (41.1 mg, 73% yield) as colorless oil. In an independent experiment, 45.1 mg (80% yield) were obtained, giving an average yield of 76%. **^{1}H NMR** (400 MHz, $CDCl_3$) δ 4.05 (t, J = 6.7 Hz, 2H), 3.94 – 3.86 (m, 2H), 2.49 – 2.41 (m, 2H), 2.22 – 2.16 (m, 2H), 2.04 (s, 3H), 1.72 – 1.55 (m, 9H), 1.44 – 1.28 (m, 6H), 0.89 (t, J = 7.5 Hz, 3H) ppm. **^{13}C NMR** (101 MHz, $CDCl_3$) δ 171.4, 160.1, 76.2, 64.7, 39.1, 32.4, 30.8, 29.1, 27.2, 26.0, 25.9, 25.5, 24.1, 23.3, 21.2, 11.2 ppm. **IR** (neat, cm^{-1}): 2929, 2859, 1739, 1449, 1365, 1234, 1037, 936, 889, 839. **HRMS** (ESI+): m/z *calcd.* for ($C_{16}H_{30}NO_3$) $[M+H]^+$: 284.2220; *found* 284.2224.

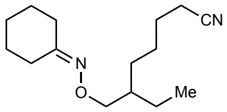

6-(((cyclohexylideneamino)oxy)methyl)octanenitrile (3ac). Following general procedure E, using cyclohexanone *O*-but-3-en-1-yl oxime (**1a**, 50.2 mg, 0.30 mmol) and 5-iodopentanenitrile (**2e**, 41.8 mg, 0.20 mmol), purification by flash column chromatography (*n*-hexane/EtOAc 10/1) afforded **3ab** (35.0 mg, 70% yield) as colorless oil. In an independent experiment, 36.9 mg (74% yield) were obtained, giving an average yield of 72%. **^{1}H NMR** (400 MHz, $CDCl_3$) δ 3.93 (dd, J = 10.1, 5.8 Hz, 1H), 3.86 (dd, J = 10.1, 6.5 Hz, 1H), 2.47 – 2.42 (m, 2H), 2.33 (t, J = 7.1 Hz, 2H), 2.22 – 2.15 (m, 2H), 1.70 – 1.62 (m, 5H), 1.64 – 1.54 (m, 4H), 1.54 –

1.41 (m, 2H), 1.41 – 1.27 (m, 4H), 0.89 (t, J = 7.5 Hz, 3H) ppm. **^{13}C NMR** (101 MHz, $CDCl_3$) δ 160.1, 119.9, 76.0, 38.9, 32.3, 30.3, 27.2, 26.1, 26.0, 25.9, 25.9, 25.4, 24.0, 17.2, 11.2 ppm. **IR** (neat, cm^{-1}): 2930, 2859, 2246, 1639, 1449, 1043, 935, 918, 887, 839. **HRMS** (ESI+): m/z *calcd.* for ($C_{15}H_{26}N_2NaO$) [M+Na]$^+$: 273.1937; *found* 273.1939.

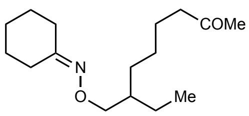

7-(((cyclohexylideneamino)oxy)methyl)nonan-2-one (3ad). Following general procedure E, using cyclohexanone *O*-but-3-en-1-yl oxime (**1a**, 50.2 mg, 0.30 mmol) and 6-iodohexan-2-one (**2f**, 45.2 mg, 0.20 mmol), purification by flash column chromatography (*n*-hexane/EtOAc 15/1) afforded **3ac** (24.8 mg, 46% yield) as colorless oil. In an independent experiment, 27.7 mg (52% yield) were obtained, giving an average yield of 49%. **^{1}H NMR** (400 MHz, $CDCl_3$) δ 3.93 – 3.85 (m, 2H), 2.48 – 2.39 (m, 4H), 2.21 – 2.16 (m, 2H), 2.13 (s, 3H), 1.71 – 1.63 (m, 2H), 1.63 – 1.54 (m, 7H), 1.40 – 1.26 (m, 6H), 0.88 (t, J = 7.5 Hz, 3H) ppm. **^{13}C NMR** (101 MHz, $CDCl_3$) δ 209.4, 160.0, 76.1, 43.9, 39.0, 32.4, 30.9, 30.0, 27.2, 26.5, 26.0, 25.9, 25.4, 24.4, 24.0, 11.2 ppm. **IR** (neat, cm^{-1}): 2927, 2857, 1716, 1449, 1358, 1162, 1045, 936, 889, 839. **HRMS** (ESI+): m/z *calcd.* for ($C_{16}H_{30}NO_2$) [M+H]$^+$: 268.2271; *found* 268.2279.

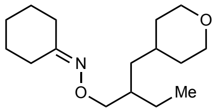

cyclohexanone *O*-(2-((tetrahydro-2*H*-pyran-4-yl)methyl)butyl) oxime (3ae). Following general procedure E, using cyclohexanone *O*-but-3-en-1-yl oxime (**1a**, 50.2 mg, 0.30 mmol) and 4-(iodomethyl)tetrahydro-2*H*-pyran (**2k**, 45.2 mg, 0.20 mmol), purification by flash column chromatographic purification (*n*-hexane/EtOAc 15/1) afforded **3ad** (33.2 mg, 62% yield) as colorless oil. In an independent experiment, 36.3 mg (68% yield) were obtained, giving an average yield of 65%. **^{1}H NMR** (400 MHz, $CDCl_3$) δ 3.96 – 3.90 (m, 3H), 3.87 (dd, J = 10.0, 6.1 Hz, 1H), 3.41 – 3.31 (m, 2H), 2.48 – 2.42 (m, 2H), 2.22 – 2.15 (m, 2H), 1.74 (p, J = 6.4 Hz, 1H), 1.70 – 1.62 (m, 3H), 1.62 – 1.54 (m, 6H), 1.42 – 1.30 (m, 2H), 1.29 – 1.17 (m, 4H), 0.89 (t, J = 7.5 Hz, 3H) ppm. **^{13}C NMR** (101 MHz, $CDCl_3$) δ 160.1, 76.5, 68.3, 39.1, 35.7, 33.8, 33.6, 32.6, 32.4, 27.2, 26.0, 26.0, 25.5, 24.7, 11.2 ppm. **IR** (neat, cm^{-1}): 2928, 2854, 1448, 1101, 1042, 936, 837. **HRMS** (ESI+): m/z *calcd.* for ($C_{16}H_{30}NO_2$) [M+H]$^+$: 268.2271; *found* 268.2276.

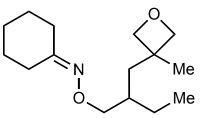

cyclohexanone *O*-(2-((3-methyloxetan-3-yl)methyl)butyl) oxime (3af). Following general procedure E, using cyclohexanone *O*-but-3-en-1-yl oxime (**1a**, 50.2 mg, 0.30 mmol) and 3-(iodomethyl)-3-methyloxetane (**2p**, 42.4 mg, 0.20 mmol), purification by flash column chromatography (*n*-hexane/EtOAc 15/1) afforded **3ae** (33.4mg, 66% yield) as colorless oil. In an independent experiment, 35.4 mg (70% yield) were obtained, giving an average yield of 68%. **^{1}H NMR** (400 MHz, $CDCl_3$) δ 4.47 (dd, J = 9.3, 5.6 Hz, 2H), 4.30 (dd, J = 5.6, 3.3 Hz, 2H), 3.83 (d, J = 5.5 Hz, 2H), 2.49 – 2.39 (m, 2H), 2.21 – 2.14 (m, 2H), 1.79 – 1.65 (m, 3H), 1.62 – 1.55 (m, 5H), 1.35 (s,

3H), 1.41 – 1.22 (m, 3H), 0.91 (t, *J* = 7.4 Hz, 3H) ppm. **^{13}C NMR** (101 MHz, $CDCl_3$) δ 160.1, 84.2, 84.0, 75.9, 41.0, 39.1, 36.3, 32.4, 27.2, 26.0, 25.9, 25.5, 25.1, 23.6, 11.3 ppm. **IR** (neat, cm^{-1}): 2926, 2858, 1640, 1449, 1379, 1046, 981, 935, 919, 887, 836. **HRMS** (ESI+): m/z *calcd.* for ($C_{15}H_{27}NNaO_2$) $[M+Na]^+$: 276.1934; *found* 276.1938.

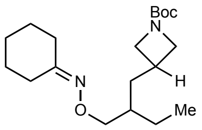

***tert*-butyl 3-(2-(((cyclohexylideneamino)oxy)methyl)butyl)azetidine-1-carboxylate (3ag).** Following general procedure A, using cyclohexanone *O*-but-3-en-1-yl oxime (**1a**, 50.2 mg, 0.30 mmol) and *tert*-butyl 3-(iodomethyl)azetidine-1-carboxylate (**2o**, 59.4 mg, 0.20 mmol), purification by flash column chromatography (*n*-hexane/EtOAc 10/1) afforded **3af** (54.7 mg, 78% yield) as colorless oil. In an independent experiment, 56.0 mg (83% yield) were obtained, giving an average yield of 81%. **^{1}H NMR** (400 MHz, $CDCl_3$) δ 3.98 (t, *J* = 8.3 Hz, 2H), 3.90 (dd, *J* = 10.1, 4.9 Hz, 1H), 3.82 (dd, *J* = 10.1, 5.9 Hz, 1H), 3.56 – 3.46 (m, 2H), 2.67 – 2.54 (m, 1H), 2.48 – 2.36 (m, 2H), 2.21 – 2.14 (m, 2H), 1.72 – 1.53 (m, 9H), 1.42 (s, 9H), 1.38 – 1.26 (m, 2H), 0.89 (t, *J* = 7.5 Hz, 3H) ppm. **^{13}C NMR** (101 MHz, $CDCl_3$) δ 160.2, 156.5, 79.2, 75.9, 37.8, 36.6, 32.3, 28.6, 27.2, 27.1, 26.0, 25.9, 25.5, 24.4, 11.3 ppm. **IR** (neat, cm^{-1}): 2961, 2928, 2875, 1701, 1449, 1397, 1364, 1131, 1047, 935, 772. **HRMS** (ESI+): m/z *calcd.* for ($C_{19}H_{34}N_2NaO_3$) $[M+Na]^+$: 361.2462; *found* 361.2467.

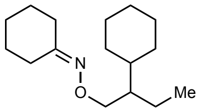

cyclohexanone *O*-(2-cyclohexylbutyl) oxime (3ah). Following general procedure E, using cyclohexanone *O*-but-3-en-1-yl oxime (**1a**, 50.2 mg, 0.30 mmol) and iodocyclohexane (**2l**, 42.0mg, 0.20 mmol), purification by flash column chromatography (*n*-hexane/EtOAc 50/1) afforded **3ag** (27.6 mg, 55% yield) as colorless oil. In an independent experiment, 29.0 mg (58% yield) were obtained, giving an average yield of 56%. **^{1}H NMR** (400 MHz, $CDCl_3$) δ 4.01 (dd, *J* = 9.9, 5.6 Hz, 1H), 3.92 (dd, *J* = 9.9, 5.9 Hz, 1H), 2.49 – 2.42 (m, 2H), 2.24 – 2.15 (m, 2H), 1.77 – 1.55 (m, 11H), 1.49 – 1.30 (m, 4H), 1.29 – 1.01 (m, 5H), 0.90 (t, *J* = 7.3 Hz, 3H) ppm. **^{13}C NMR** (101 MHz, $CDCl_3$) δ 159.9, 74.6, 44.8, 39.2, 32.4, 30.6, 30.0, 27.2, 27.1, 27.1, 26.9, 26.0, 26.0, 25.6, 21.8, 12.4 ppm. **IR** (neat, cm^{-1}): 2922, 2851, 1640, 1448, 1042, 936, 889, 839. **HRMS** (ESI+): m/z *calcd.* for ($C_{16}H_{30}NO$) $[M+H]^+$: 252.2322; *found* 252.2328.

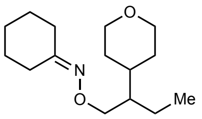

cyclohexanone *O*-(2-(tetrahydro-2*H*-pyran-4-yl)butyl) oxime (3ai). Following general procedure E, cyclohexanone *O*-but-3-en-1-yl oxime (**1a**, 50.2 mg, 0.30 mmol) and 4-iodotetrahydro-2*H*-pyran (**2m**, 42.4, 0.20 mmol), purification by flash column chromatography (*n*-hexane/EtOAc 15/1) afforded **3ah** (33.9 mg, 67% yield) as colorless oil. In an independent experiment, 37.0 mg (73% yield) were obtained, giving an average yield of 70%. **^{1}H NMR** (400 MHz, $CDCl_3$) δ 4.05 – 3.94 (m, 4H), 3.36 (tt, *J* = 11.6, 2.4 Hz, 2H), 2.49 – 2.39

(m, 2H), 2.25 – 2.14 (m, 2H), 1.71 – 1.58 (m, 7H), 1.57 – 1.52 (m, 2H), 1.51 – 1.39 (m, 4H), 1.39 – 1.29 (m, 1H), 0.92 (t, J = 7.3 Hz, 3H) ppm. **^{13}C NMR** (101 MHz, $CDCl_3$) δ 160.2, 73.7, 68.7, 68.7, 44.3, 36.6, 32.4, 30.6, 30.4, 27.2, 26.0, 26.0, 25.6, 21.2, 12.0 ppm. **IR** (neat, cm^{-1}): 2927, 2854, 1448, 1380, 1157, 1101, 1041, 1017, 984, 936, 872, 838. **HRMS** (ESI+): m/z *calcd.* for ($C_{15}H_{28}NO_2$) [M+H]$^+$: 254.2115; *found* 254.2122.

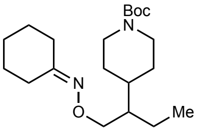

***tert*-butyl 4-(1-((cyclohexylideneamino)oxy)butan-2-yl)piperidine-1-carboxylate (3aj).** Following general procedure E, using cyclohexanone *O*-but-3-en-1-yl oxime (**1a**, 50.2 mg, 0.30 mmol) and *tert*-butyl 4-iodopiperidine-1-carboxylate (**2n**, 62.2 mg, 0.20 mmol), purification by flash column chromatography (*n*-hexane/EtOAc 10/1) afforded **3ai** (49.4 mg, 70% yield) as colorless oil. In an independent experiment, 52.6 mg (75% yield) were obtained, giving an average yield of 72%. **^{1}H NMR** (400 MHz, $CDCl_3$) δ 4.11 (s, 2H), 4.04 – 3.91 (m, 2H), 2.62 (t, J = 12.7 Hz, 2H), 2.48 – 2.40 (m, 2H), 2.27 – 2.11 (m, 2H), 1.71 – 1.48 (m, 10H), 1.45 (s, 9H), 1.42 – 1.26 (m, 4H), 0.92 (t, J = 7.4 Hz, 3H) ppm. **^{13}C NMR** (101 MHz, $CDCl_3$) δ 160.2, 155.0, 79.3, 73.9, 44.1, 37.7, 32.4, 29.5, 29.3, 28.6, 27.2, 26.0, 26.0, 25.6, 21.5, 12.2 ppm. **IR** (neat, cm^{-1}): 2927, 2856, 1692, 1448, 1421, 1364, 1278, 1232, 1171, 1041, 935, 870, 768. **HRMS** (ESI+): m/z *calcd.* for ($C_{20}H_{36}N_2NaO_3$) [M+Na]$^+$: 375.2618; *found* 375.2626.

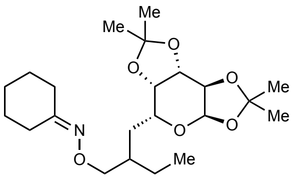

cyclohexanone *O*-(2-(((3a*R*,5*R*,5a*S*,8a*S*,8b*R*)-2,2,7,7-tetramethyltetrahydro-5*H*-bis([1,3]dioxolo) [4,5-*b*:4',5'-*d*]pyran-5-yl)methyl)butyl) oxime (3ak). Following general procedure E, using cyclohexanone *O*-but-3-en-1-yl oxime (**1a**, 50.2 mg, 0.30 mmol) and (**2t**, 3a*R*,5*S*,5a*R*,8a*S*,8b*R*)-5-(iodomethyl)-2,2,7,7-tetramethyltetrahydro-5*H*-bis([1,3]dioxolo)[4,5-*b*:4',5'-d]pyran (74.1 mg, 0.20 mmol), purification by flash column chromatography (*n*-hexane/EtOAc 15/1) afforded **3aj** (41.2 mg, 50% yield, dr 1:1) as colorless oil. In an independent experiment, 42.8 mg (52% yield) were obtained, giving an average yield of 51%. **^{1}H NMR** (400 MHz, $CDCl_3$) δ 5.49 (t, J = 4.8 Hz, 1H), 4.55 (t, J = 2.7 Hz, 0.5H), 4.53 (t, J = 2.6 Hz, 0.5H), 4.28 – 4.23 (m, 0.5H), 4.25 – 4.24 (m, 0.5H), 4.12 (dd, J = 7.9, 1.8 Hz, 0.5H), 4.07 (dd, J = 7.9, 1.9 Hz, 0.5H), 4.02 (dd, J = 10.2, 5.0 Hz, 0.5H), 3.98 – 3.93 (m, 1H), 3.92 (s, 0.5H), 3.88 – 3.86 (m, 0.5H), 3.88 – 3.82 (m, 0.5H), 2.47 – 2.34 (m, 2H), 2.18 – 2.08 (m, 2H), 1.93 – 1.83 (m, 0.5H), 1.81 – 1.73 (m, 0.5H), 1.72 – 1.60 (m, 3H), 1.60 – 1.50 (m, 5H), 1.51 (s, 1.5H), 1.48 (s, 1.5H), 1.43 (s, 1.5H), 1.42 (s, 1.5H), 1.41 – 1.33 (m, 2H), 1.31 (s, 3H), 1.29 (s, 3H), 0.90 (t, J = 7.5 Hz, 1.5H), 0.88 (t, J = 7.5 Hz, 1.5H) ppm. **^{13}C NMR** (101 MHz, $CDCl_3$) δ 159.7 (159.6), 109.0, 108.3 (108.3), 96.8 (96.7), 76.2 (74.3), 73.5 (73.1), 71.2, 70.6 (70.6), 65.5 (65.2), 36.2 (35.2), 32.3 (32.2), 31.9 (31.4), 27.1 (27.1), 26.1, 26.0, 26.0 (26.0), 25.9 (25.8), 25.4 (25.3), 25.0 (25.0), 24.6 (24.5), 23.6, 11.4 (10.7) ppm. **IR** (neat, cm^{-1}): 2927, 2860, 1450, 1380, 1254, 1209, 1168, 1100, 1066, 1033, 994, 917, 891. **HRMS** (ESI+): m/z *calcd.* for ($C_{22}H_{38}NO_6$) [M+H]$^+$: 412.2694; *found* 412.2706.

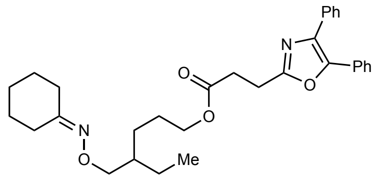

4-(((cyclohexylideneamino)oxy)methyl)hexyl 3-(4,5-diphenyloxazol-2-yl)propanoate (3al). Following general procedure E, using cyclohexanone *O*-but-3-en-1-yl oxime (**1a**, 50.2 mg, 0.30 mmol) and 3-iodopropyl 3-(4,5-diphenyloxazol-2-yl)propanoate (**2r**, 92.3 mg, 0.20 mmol), purification by flash column chromatography (*n*-hexane/EtOAc 20/1) afforded **3an** (80.4 mg, 80% yield) as colorless oil. In an independent experiment, 82.4 mg (82% yield) were obtained, giving an average yield of 81%. **^{1}H NMR** (400 MHz, $CDCl_3$) δ 7.65 – 7.60 (m, 2H), 7.59 – 7.55 (m, 2H), 7.39 – 7.29 (m, 6H), 4.16 – 4.07 (m, 2H), 3.93 (dd, *J* = 10.1, 5.8 Hz, 1H), 3.87 (dd, *J* = 10.2, 6.4 Hz, 1H), 3.23 – 3.14 (m, 2H), 2.95 – 2.86 (m, 2H), 2.49 – 2.40 (m, 2H), 2.21 – 2.13 (m, 2H), 1.73 – 1.62 (m, 5H), 1.61 – 1.54 (m, 4H), 1.45 – 1.27 (m, 4H), 0.88 (t, *J* = 7.5 Hz, 3H) ppm. **^{13}C NMR** (101 MHz, $CDCl_3$) δ 172.2, 161.9, 160.1, 145.5, 135.3, 132.6, 129.1, 128.8, 128.7, 128.6, 128.2, 128.0, 126.6, 76.0, 65.4, 38.8, 32.4, 31.3, 27.4, 27.2, 26.1, 26.0, 25.9, 25.5, 24.0, 23.7, 11.2 ppm. **IR** (neat, cm^{-1}): 2929, 2858, 1734, 1447, 1219, 1170, 1055, 1025, 962, 935, 916, 762, 693. **HRMS** (ESI+): m/z *calcd.* for ($C_{31}H_{39}N_2O_4$) [M+H]$^+$: 503.2904; *found* 503.2913.

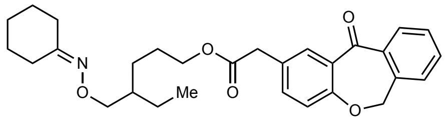

4-(((cyclohexylideneamino)oxy)methyl)hexyl 2-(11-oxo-6,11-dihydrodibenzo[*b*,*e*]oxepin-2-yl)acetate (3am). Following general procedure E, using cyclohexanone *O*-but-3-en-1-yl oxime (**1a**, 50.2 mg, 0.30 mmol) and 3-iodopropyl 2-(11-oxo-6,11-dihydrodibenzo[*b*,*e*]oxepin-2-yl)acetate (**2s**, 87.3 mg, 0.20 mmol), purification by flash column chromatography (*n*-hexane/EtOAc 15/1) afforded **3ak** (60.2 mg, 63% yield) as colorless oil. In an independent experiment, 63.0 mg (66% yield) were obtained, giving an average yield of 64%. **^{1}H NMR** (400 MHz, $CDCl_3$) δ 8.11 (d, *J* = 2.4 Hz, 1H), 7.89 (dd, *J* = 7.7, 1.4 Hz, 1H), 7.55 (td, *J* = 7.4, 1.4 Hz, 1H), 7.47 (td, *J* = 7.6, 1.3 Hz, 1H), 7.43 (dd, *J* = 8.4, 2.4 Hz, 1H), 7.36 (dd, *J* = 7.4, 1.3 Hz, 1H), 7.02 (d, *J* = 8.4 Hz, 1H), 5.18 (s, 2H), 4.14 – 4.04 (m, 2H), 3.92 (dd, *J* = 10.1, 5.9 Hz, 1H), 3.86 (dd, *J* = 10.1, 6.4 Hz, 1H), 3.63 (s, 2H), 2.49 – 2.38 (m, 2H), 2.23 – 2.12 (m, 2H), 1.70 – 1.61 (m, 5H), 1.63 – 1.53 (m, 4H), 1.41 – 1.27 (m, 4H), 0.87 (t, *J* = 7.5 Hz, 3H) ppm. **^{13}C NMR** (101 MHz, $CDCl_3$) δ 190.9, 171.6, 160.6, 160.1, 140.6, 136.5, 135.7, 132.9, 132.6, 129.6, 129.4, 128.1, 127.9, 125.3, 121.2, 76.0, 73.8, 65.6, 40.4, 38.8, 32.3, 27.4, 27.2, 26.1, 26.0, 26.0, 25.5, 24.0, 11.2 ppm. **IR** (neat, cm^{-1}): 3076, 2928, 2858, 1732, 1648, 1611, 1599, 1489, 1451, 1413, 1379, 1298, 1284, 1254, 1220, 1203, 1160, 1138, 1120, 1045, 1015, 935, 831, 759, 641. **HRMS** (ESI+): m/z *calcd.* for ($C_{29}H_{36}NO_5$) [M+H]$^+$: 478.2588; *found* 478.2592.

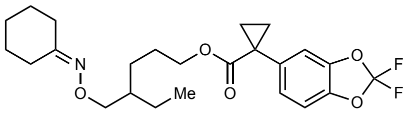

4-(((cyclohexylideneamino)oxy)methyl)hexyl 1-(2,2-difluorobenzo[*d*][1,3]dioxol-5-yl)cyclopropane-1-carboxylate (3an). Following general procedure E, using cyclohexanone *O*-but-3-en-1-yl oxime (**1a**, 50.2 mg, 0.30 mmol) and 3-iodopropyl 1-(2,2-difluorobenzo[*d*][1,3]dioxol-5-yl)cyclopropane-1-carboxylate (**2q**, 82.0 mg, 0.20 mmol), purification by flash column chromatography (*n*-hexane/EtOAc 25/1) afforded **3al** (52.2 mg, 58% yield) as colorless oil. In an independent experiment, 56.9 mg (63% yield) were obtained, giving an average yield of 61%. **^{1}H NMR** (400 MHz, $CDCl_3$) δ 7.07 – 7.01 (m, 2H), 7.00 – 6.92 (m, 1H), 4.06 – 3.95 (m, 2H), 3.88 (dd, *J* = 10.1, 6.0 Hz, 1H), 3.83 (dd, *J* = 10.1, 6.4 Hz, 1H), 2.46 – 2.38 (m, 2H), 2.21 – 2.13 (m, 2H), 1.70 – 1.62 (m, 2H), 1.62 – 1.52 (m, 9H), 1.40 – 1.29 (m, 1H), 1.29 – 1.20 (m, 3H), 1.15 (q, *J* = 4.0 Hz, 2H), 0.83 (t, *J* = 7.5 Hz, 3H) ppm. **^{13}C NMR** (101 MHz, $CDCl_3$) δ 174.1, 160.0, 143.5, 142.9, 136.0, 131.8 (t, *J* = 255.0 Hz), 125.8, 112.1, 108.9, 76.0, 65.7, 38.7, 32.3, 29.2, 27.2, 26.0, 25.9, 25.9, 25.4, 23.9, 16.8, 11.0 ppm. **^{19}F NMR** (376 MHz, $CDCl_3$) δ -50.0 ppm. **IR** (neat, cm^{-1}): 2930, 2859, 1720, 1503, 1441, 1293, 1231, 1152, 1074, 1033, 934, 885, 704. **HRMS** (ESI+): m/z *calcd.* for ($C_{24}H_{32}F_2NO_5$) $[M+H]^+$: 452.2243; *found* 452.2239.

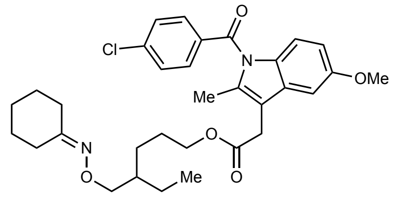

4-(((cyclohexylideneamino)oxy)methyl)hexyl 2-(1-(4-chlorobenzoyl)-5-methoxy-2-methyl-1*H*-indol-3-yl)acetate (3ao). Following general procedure E, using cyclohexanone *O*-but-3-en-1-yl oxime (**1a**, 50.2 mg, 0.30 mmol) and 3-iodopropyl 2-(1-(4-chlorobenzoyl)-5-methoxy-2-methyl-1*H*-indol-3-yl)acetate (**2u**, 105.2 mg, 0.20 mmol), purification by flash column chromatography (*n*-hexane/EtOAc 10/1) afforded **3am** (76.3 mg, 67% yield) as colorless oil. In an independent experiment, 86.2 mg (76% yield) were obtained, giving an average yield of 72%. **^{1}H NMR** (400 MHz, $CDCl_3$) δ 7.70 – 7.63 (m, 2H), 7.50 – 7.43 (m, 2H), 6.96 (d, *J* = 2.5 Hz, 1H), 6.86 (d, *J* = 9.0 Hz, 1H), 6.66 (dd, *J* = 9.0, 2.6 Hz, 1H), 4.09 (td, *J* = 6.7, 1.1 Hz, 2H), 3.90 (dd, *J* = 10.2, 5.9 Hz, 1H), 3.87 – 3.84 (m, 4H), 3.65 (s, 2H), 2.46 – 2.40 (m, 2H), 2.38 (s, 3H), 2.21 – 2.14 (m, 2H), 1.70 – 1.62 (m, 5H), 1.63 – 1.54 (m, 4H), 1.38 – 1.25 (m, 4H), 0.85 (t, *J* = 7.5 Hz, 3H) ppm. **^{13}C NMR** (101 MHz, $CDCl_3$) δ 171.1, 168.4, 160.2, 156.2, 139.4, 136.0, 134.1, 131.3, 131.0, 130.8, 129.3, 115.1, 112.9, 111.8, 101.5, 76.0, 65.6, 55.8, 38.8, 32.3, 30.6, 27.4, 27.2, 26.1, 26.0, 25.9, 25.5, 24.0, 13.5, 11.2 ppm. **IR** (neat, cm^{-1}): 2930, 2859, 1734, 1685, 1592, 1478, 1457, 1357, 1317, 1260, 1223, 1167, 1143, 1089, 1068, 1039, 1015, 926, 836, 745. **HRMS** (ESI+): m/z *calcd.* for ($C_{32}H_{40}ClN_2O_5$) $[M+H]^+$: 567.2620; *found* 567.2628.

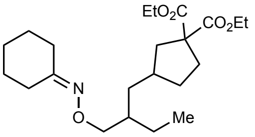

diethyl 3-(2-(((cyclohexylideneamino)oxy)methyl)butyl)cyclopentane-1,1-dicarboxylate (3ap). Following general procedure E, using cyclohexanone *O*-but-3-en-1-yl oxime (**1a**, 50.2 mg, 0.30 mmol) and diethyl 2-allyl-

2-(2-iodoethyl)malonate (70.8 mg, 0.20 mmol), purification by flash column chromatography (*n*-hexane/EtOAc 10/1) afforded **3ao** (39.6 mg, 50% yield, dr 1:1) as colorless oil. In an independent experiment, 42.0 mg (53% yield) were obtained, giving an average yield of 51%. **^{1}H NMR** (400 MHz, $CDCl_3$) δ 4.22 – 4.12 (m, 4H), 3.95 – 3.84 (m, 2H), 2.49 – 2.40 (m, 3H), 2.34 – 2.24 (m, 1H), 2.21 – 2.16 (m, 2H), 2.16 – 2.02 (m, 1H), 1.93 – 1.81 (m, 1H), 1.74 – 1.62 (m, 5H), 1.63 – 1.53 (m, 4H), 1.41 – 1.29 (m, 4H), 1.27 – 1.18 (m, 7H), 0.88 (t, *J* = 7.5 Hz, 3H) ppm. **^{13}C NMR** (101 MHz, $CDCl_3$) δ 173.0 (173.0), 172.9, 160.1, 76.4 (76.2), 61.4, 60.2, 60.1, 41.3 (41.2), 38.2 (38.1), 37.6 (37.5), 37.2 (37.1), 33.9 (33.9), 32.6, 32.3, 27.2, 26.0 (25.9), 25.4, 24.8, 24.4 (24.4), 14.2, 11.2, 11.1 ppm. **IR** (neat, cm^{-1}): 2958, 2928, 2857, 1731, 1462, 1274, 1210, 1187, 1146, 1041, 968. **HRMS** (ESI+): m/z *calcd.* for ($C_{22}H_{38}NO_5$) $[M+H]^+$: 396.2744; *found* 396.2749.

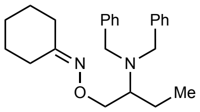

cyclohexanone *O*-(2-(dibenzylamino)butyl) oxime (5a). Following general procedure G, using cyclohexanone *O*-but-3-en-1-yl oxime (**1a**, 33.5 mg, 0.20 mmol) and *O*-benzoyl-*N*,*N*-dibenzylhydroxylamine (**4a**, 95.2 mg, 0.30 mmol), purification by flash column chromatography (*n*-hexane/EtOAc 50/1) afforded **5a** (59.0 mg, 81% yield) as a white solid. In an independent experiment, 58.0 mg (79% yield) were obtained, giving an average yield of 80%. **^{1}H NMR** (400 MHz, $CDCl_3$) δ 7.43 – 7.37 (m, 4H), 7.33 – 7.26 (m, 4H), 7.25 – 7.18 (m, 2H), 4.27 (dd, *J* = 10.6, 6.8 Hz, 1H), 4.10 (dd, *J* = 10.6, 4.7 Hz, 1H), 3.81 (d, *J* = 13.8 Hz, 2H), 3.67 (d, *J* = 13.8 Hz, 2H), 2.89 – 2.78 (m, 1H), 2.60 – 2.47 (m, 2H), 2.28 – 2.20 (m, 2H), 1.77 – 1.53 (m, 7H), 1.51 – 1.37 (m, 1H), 0.94 (t, *J* = 7.4 Hz, 3H) ppm. **^{13}C NMR** (101 MHz, $CDCl_3$) δ 159.9, 141.0, 129.0, 128.1, 126.7, 73.8, 58.2, 54.3, 32.3, 27.2, 26.0, 25.9, 25.8, 22.1, 11.9 ppm. **IR** (neat, cm^{-1}): 3025, 2933, 2856, 1493, 1446, 1434, 1358, 1259, 1143, 1063, 1043, 994, 904, 867, 743, 694. **HRMS** (ESI+): m/z *calcd.* for ($C_{24}H_{33}N_2O$) $[M+H]^+$: 365.2587; *found* 365.2589. **MP:** 56 – 58 ºC.

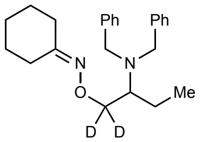

cyclohexanone *O*-(2-(dibenzylamino)butyl-1,1-*d*$_2$) oxime (5a-*d*$_2$). Following general procedure G, using cyclohexanone *O*-but-3-en-1-yl-1,1-*d*$_2$ oxime (**1a-*d*$_2$**, 33.8 mg, 0.20 mmol) and *O*-benzoyl-*N*,*N*-dibenzylhydroxylamine (**4a**, 95.2 mg, 0.30 mmol), purification by flash column chromatography (*n*-hexane/EtOAc 50/1) afforded **5a-*d*$_2$** (59.9 mg, 82% yield) as a white solid. In an independent experiment, 57.1 mg (78% yield) were obtained, giving an average yield of 80%. **^{1}H NMR** (400 MHz, $CDCl_3$) δ 7.45 – 7.39 (m, 4H), 7.35 – 7.28 (m, 4H), 7.26 – 7.20 (m, 2H), 3.82 (d, *J* = 13.8 Hz, 2H), 3.69 (dd, *J* = 13.7, 1.9 Hz, 2H), 2.89 – 2.82 (m, 1H), 2.62 – 2.48 (m, 2H), 2.29 – 2.22 (m, 2H), 1.77 – 1.56 (m, 7H), 1.52 – 1.40 (m, 1H), 0.99 – 0.92 (m, 3H) ppm. **^{13}C NMR** (101 MHz, $CDCl_3$) δ 159.9, 140.9, 128.9, 128.1, 126.7, 58.0, 54.3, 32.3, 27.2, 26.0, 25.9, 25.7, 22.1, 11.9 ppm. **IR** (neat, cm^{-1}): 3026, 2933, 2856, 1493, 1447, 1360, 1142, 1101, 1074, 1043, 977, 964, 820, 745, 695. **HRMS** (ESI+): m/z *calcd.* for ($C_{24}H_{31}D_2N_2O$) $[M+H]^+$: 367.2713; *found* 367.2719. **MP**: 56 – 58 ºC.

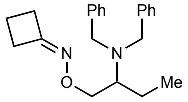

cyclobutanone *O*-(2-(dibenzylamino)butyl) oxime (5b). Following general procedure G, using cyclobutanone *O*-but-3-en-1-yl oxime (**1e**, 27.8 mg, 0.20 mmol) and *O*-benzoyl-*N*,*N*-dibenzylhydroxylamine (**4a**, 95.2 mg, 0.30 mmol). ^{1}H NMR and GC-FID analysis of the crude mixture showed a rr of 8:1 (rr refers to the ratio of the major regioisomer to all other regioisomers). Purification by flash column chromatography (*n*-hexane/EtOAc 50/1) afforded **5b** (38.0 mg, 56% yield) as a white solid. In an independent experiment, 41.8 mg (62% yield) were obtained, giving an average yield of 59%. **^{1}H NMR** (400 MHz, $CDCl_3$) δ 7.43 – 7.37 (m, 4H), 7.33 – 7.26 (m, 4H), 7.25 – 7.18 (m, 2H), 4.27 (dd, *J* = 10.7, 7.0 Hz, 1H), 4.08 (dd, *J* = 10.7, 4.7 Hz, 1H), 3.79 (d, *J* = 13.8 Hz, 2H), 3.67 (d, *J* = 13.8 Hz, 2H), 3.01 – 2.90 (m, 4H), 2.88 – 2.79 (m, 1H), 2.09 – 1.98 (m, 2H), 1.66 – 1.53 (m, 1H), 1.49 – 1.36 (m, 1H), 0.94 (t, *J* = 7.4 Hz, 3H) ppm. **^{13}C NMR** (101 MHz, $CDCl_3$) δ 158.4, 140.9, 129.0, 128.1, 126.7, 74.4, 58.1, 54.2, 31.7, 31.4, 22.0, 14.7, 11.9 ppm. **IR** (neat, cm^{-1}): 3025, 2927, 2854, 1494, 1452, 1361, 1244, 1164, 1021, 947, 913, 876, 747, 698. **HRMS** (ESI+): m/z *calcd.* for ($C_{22}H_{29}N_2O$) $[M+H]^+$: 337.2274; *found* 337.2287. **MP**: 46 – 48 ºC.

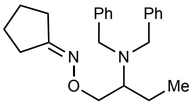

cyclopentanone *O*-(2-(dibenzylamino)butyl) oxime (5c). Following general procedure G, using cyclopentanone O-but-3-en-1-yl oxime **1b**, 30.6 mg, 0.20 mmol) and *O*-benzoyl-*N*,*N*-dibenzylhydroxylamine (**4a**, 95.2 mg, 0.30 mmol), purification by flash column chromatography (*n*-hexane/EtOAc 50/1) afforded **5c** (58.2 mg, 83% yield) as a white solid. In an independent experiment, 53.3 mg (76% yield) were obtained, giving an average yield of 79%. **^{1}H NMR** (400 MHz, $CDCl_3$) δ 7.45 – 7.37 (m, 4H), 7.35 – 7.26 (m, 4H), 7.25 – 7.18 (m, 2H), 4.29 (dd, *J* = 10.6, 7.0 Hz, 1H), 4.13 (dd, *J* = 10.6, 4.6 Hz, 1H), 3.81 (d, *J* = 13.8 Hz, 2H), 3.69 (d, *J* = 13.8 Hz, 2H), 2.90 – 2.80 (m, 1H), 2.53 – 2.44 (m, 2H), 2.44 – 2.36 (m, 2H), 1.86 – 1.72 (m, 4H), 1.68 – 1.55 (m, 1H), 1.50 – 1.37 (m, 1H), 0.95 (t, *J* = 7.4 Hz, 3H) ppm. **^{13}C NMR** (101 MHz, $CDCl_3$) δ 166.2, 141.0, 129.0, 128.1, 126.7, 74.5, 58.1, 54.3, 31.1, 28.2, 25.4, 24.9, 22.1, 11.9 ppm. **IR** (neat, cm^{-1}): 3026, 2924, 2853, 1493, 1453, 1362, 1098, 1041, 964, 926, 743, 696. **HRMS** (ESI+): m/z *calcd.* for ($C_{23}H_{31}N_2O$) $[M+H]^+$: 351.2431; *found* 351.2439. **MP:** 45 – 47 ºC.

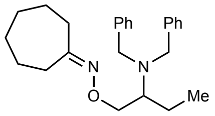

cycloheptanone *O*-(2-(dibenzylamino)butyl) oxime (5d). Following general procedure G, using cycloheptanone *O*-but-3-en-1-yl oxime (**1c**, 39.1 mg, 0.20 mmol) and *O*-benzoyl-*N*,*N*-dibenzylhydroxylamine (**4a**, 95.2 mg, 0.30 mmol), purification by flash column chromatography (*n*-hexane/EtOAc 50/1) afforded **5d** (39.4 mg, 52% yield) as a light-yellow oil. In an independent experiment, 44.7 mg (59% yield) were obtained, giving an average yield of 56%. **^{1}H NMR** (400 MHz, $CDCl_3$) δ 7.44 – 7.38 (m, 4H), 7.34 – 7.27 (m, 4H), 7.25

– 7.19 (m, 2H), 4.28 (dd, J = 10.6, 6.7 Hz, 1H), 4.13 (dd, J = 10.6, 4.7 Hz, 1H), 3.82 (d, J = 13.8 Hz, 2H), 3.69 (d, J = 13.8 Hz, 2H), 2.90 – 2.80 (m, 1H), 2.68 – 2.52 (m, 2H), 2.45 – 2.38 (m, 2H), 1.75 – 1.54 (m, 9H), 1.52 – 1.39 (m, 1H), 0.95 (t, J = 7.4 Hz, 3H) ppm. **^{13}C NMR** (101 MHz, $CDCl_3$) δ 163.5, 141.0, 129.0, 128.1, 126.7, 73.8, 58.2, 54.3, 33.9, 30.5, 29.6, 27.9, 24.9, 22.1, 11.9 ppm. **IR** (neat, cm^{-1}): 3025, 2972, 2853, 1493, 1453, 1364, 1143, 1061, 994, 870, 841, 745, 698. **HRMS** (ESI+): m/z *calcd.* for ($C_{25}H_{35}N_2O$) [M+H]$^+$: 379.2744; *found* 379.2752.

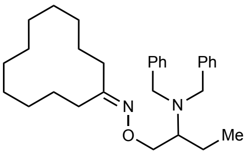

cyclododecanone *O*-(2-(dibenzylamino)butyl) oxime (5e). Following general procedure G, using cyclododecanone *O*-but-3-en-1-yl oxime (**1d**, 50.3 mg, 0.20 mmol) and *O*-benzoyl-*N*,*N*-dibenzylhydroxylamine (**4a**, 95.2 mg, 0.30 mmol), purification by flash column chromatography (*n*-hexane/EtOAc 60/1) afforded **5e** (66.7 mg, 74% yield) as a light-yellow oil. In an independent experiment, 68.1 mg (78% yield) were obtained, giving an average yield of 76%. **^{1}H NMR** (400 MHz, $CDCl_3$) δ 7.44 – 7.38 (m, 4H), 7.34 – 7.27 (m, 4H), 7.25 – 7.19 (m, 2H), 4.29 (ddd, J = 10.6, 6.1, 2.0 Hz, 1H), 4.12 (ddd, J = 10.5, 5.1, 1.9 Hz, 1H), 3.84 (d, J = 13.6 Hz, 2H), 3.68 (dd, J = 13.8, 1.9 Hz, 2H), 2.88 – 2.79 (m, 1H), 2.55 – 2.45 (m, 1H), 2.41 – 2.32 (m, 1H), 2.32 – 2.20 (m, 2H), 1.78 – 1.46 (m, 6H), 1.46 – 1.28 (m, 14H), 0.95 (td, J = 7.4, 1.9 Hz, 3H) ppm. **^{13}C NMR** (101 MHz, $CDCl_3$) δ 159.8, 141.0, 128.9, 128.1, 126.7, 73.5, 58.3, 54.3, 30.8, 27.0, 25.4, 25.3, 24.9, 24.4, 23.7, 23.5, 23.4, 23.4, 22.9, 22.2, 11.9 ppm. **IR** (neat, cm^{-1}): 3026, 2927, 2862, 1708, 1468, 1453, 1362, 1250, 1149, 1043, 971, 910, 821, 742, 696. **HRMS** (ESI+): m/z *calcd.* for ($C_{30}H_{45}N_2O$) [M+H]$^+$: 449.3526; *found* 449.3531.

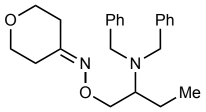

tetrahydro-4*H*-pyran-4-one *O*-(2-(dibenzylamino)butyl) oxime (5f). Following general procedure G, using tetrahydro-4*H*-pyran-4-one *O*-but-3-en-1-yl oxime (**1f**, 33.8 mg, 0.20 mmol) and *O*-benzoyl-*N*,*N*-dibenzylhydroxylamine (**4a**, 95.2 mg, 0.30 mmol), purification by flash column chromatography (*n*-hexane/EtOAc 50/1) afforded **5f** (48.5 mg, 66% yield) as a light-yellow solid. In an independent experiment, 43.3 mg (59% yield) were obtained, giving an average yield of 63%. **^{1}H NMR** (400 MHz, $CDCl_3$) δ 7.42 – 7.37 (m, 4H), 7.34 – 7.27 (m, 4H), 7.25 – 7.19 (m, 2H), 4.30 (dd, J = 10.7, 6.6 Hz, 1H), 4.12 (dd, J = 10.7, 4.8 Hz, 1H), 3.84 (t, J = 5.8 Hz, 2H), 3.80 (d, J = 13.7 Hz, 2H), 3.77 (t, J = 5.9 Hz, 2H), 3.66 (d, J = 13.8 Hz, 2H), 2.89 – 2.80 (m, 1H), 2.74 – 2.61 (m, 2H), 2.39 (t, J = 5.6 Hz, 2H), 1.68 – 1.57 (m, 1H), 1.51 – 1.38 (m, 1H), 0.95 (t, J = 7.4 Hz, 3H) ppm. **^{13}C NMR** (101 MHz, $CDCl_3$) δ 154.7, 140.8, 128.9, 128.2, 126.8, 74.0, 68.5, 66.9, 58.2, 54.3, 32.5, 27.2, 21.9, 11.9 ppm. **IR** (neat, cm^{-1}): 2960, 2852, 1496, 1453, 1362, 1287, 1229, 1098, 1042, 998, 938, 849, 743, 696. **HRMS** (ESI+): m/z *calcd.* for ($C_{23}H_{31}N_2O_2$) [M+H]$^+$: 367.2387; *found* 367.2380. **MP**: 37 – 39 ºC.

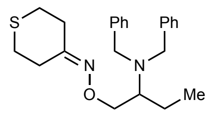

tetrahydro-4*H*-thiopyran-4-one *O*-(2-(dibenzylamino)butyl) oxime (5g). Following general procedure G, using tetrahydro-4*H*-thiopyran-4-one *O*-but-3-en-1-yl oxime (**1g**, 37.1 mg, 0.20 mmol) and *O*-benzoyl-*N*,*N*-dibenzylhydroxylamine (**4a**, 95.2 mg, 0.30 mmol), purification by flash column chromatography (*n*-hexane/EtOAc 50/1) afforded **5g** (55.6 mg, 73% yield) as a light-yellow solid. In an independent experiment, 58.2 mg (76% yield) were obtained, giving an average yield of 74%. **^{1}H NMR** (400 MHz, $CDCl_3$) δ 7.42 – 7.36 (m, 4H), 7.33 – 7.27 (m, 4H), 7.25 – 7.19 (m, 2H), 4.28 (dd, *J* = 10.7, 6.6 Hz, 1H), 4.10 (dd, *J* = 10.7, 4.8 Hz, 1H), 3.79 (d, *J* = 13.8 Hz, 2H), 3.65 (d, *J* = 13.8 Hz, 2H), 2.95 – 2.79 (m, 5H), 2.75 (t, *J* = 5.9 Hz, 2H), 2.61 – 2.54 (m, 2H), 1.70 – 1.56 (m, 1H), 1.50 – 1.38 (m, 1H), 0.95 (t, *J* = 7.4 Hz, 3H) ppm. **^{13}C NMR** (101 MHz, $CDCl_3$) δ 156.8, 140.8, 128.9, 128.2, 126.8, 74.0, 58.2, 54.2, 34.0, 29.9, 28.5, 27.9, 21.9, 11.9 ppm. **IR** (neat, cm^{-1}): 3027, 2960, 2926, 2855, 1493, 1455, 1423, 1359, 1271, 1147, 1062, 993, 936, 873, 743, 694. **HRMS** (ESI+): m/z *calcd.* for ($C_{23}H_{31}N_2OS$) $[M+H]^+$: 383.2152; *found* 383.2165. **MP**: 57 – 59 ºC.

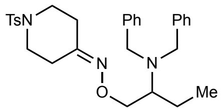

1-tosylpiperidin-4-one *O*-(2-(dibenzylamino)butyl) oxime (5h). Following general procedure G, using 1-tosylpiperidin-4-one *O*-but-3-en-1-yl oxime (**1h**, 64.5 mg, 0.20 mmol) and *O*-benzoyl-*N*,*N*-dibenzylhydroxylamine (**4a**, 95.2 mg, 0.30 mmol), purification by flash column chromatography (*n*-hexane/EtOAc 2/1) afforded **5h** (62.4 mg, 60% yield) as a light-yellow solid. In an independent experiment, 57.3 mg (55% yield) were obtained, giving an average yield of 57%. **^{1}H NMR** (400 MHz, $CDCl_3$) δ 7.68 – 7.63 (m, 2H), 7.35 – 7.30 (m, 6H), 7.26 – 7.21 (m, 4H), 7.21 – 7.15 (m, 2H), 4.21 (dd, *J* = 10.7, 6.7 Hz, 1H), 4.01 (dd, *J* = 10.7, 4.8 Hz, 1H), 3.72 (d, *J* = 13.8 Hz, 2H), 3.58 (d, *J* = 13.8 Hz, 2H), 3.19 (t, *J* = 6.0 Hz, 2H), 3.13 (t, *J* = 6.1 Hz, 2H), 2.80 – 2.73 (m, 1H), 2.73 – 2.64 (m, 2H), 2.44 – 2.38 (m, 5H), 1.65 – 1.52 (m, 1H), 1.42 – 1.30 (m, 1H), 0.89 (t, *J* = 7.4 Hz, 3H) ppm. **^{13}C NMR** (101 MHz, $CDCl_3$) δ 153.7, 144.0, 140.7, 133.4, 129.9, 128.9, 128.2, 127.8, 126.8, 74.1, 58.2, 54.2, 46.8, 45.4, 31.1, 25.1, 21.8, 21.7, 11.9 ppm. **IR** (neat, cm^{-1}): 3085, 3025, 3966, 2925, 2901, 2860, 2840, 2799, 1597, 1493, 1462, 1360, 1340, 1302, 1236, 1163, 1099, 1039, 997, 924, 817, 730, 697, 682, 586, 548. **HRMS** (ESI+): m/z *calcd.* for ($C_{30}H_{38}N_3O_3S$) $[M+H]^+$: 520.2628; *found* 520.2616. **MP**: 138 – 140 ºC.

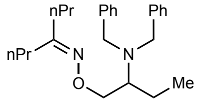

heptan-4-one *O*-(2-(dibenzylamino)butyl) oxime (5i). Following general procedure G, using heptan-4-one *O*-but-3-en-1-yl oxime (**1j**, 36.7 mg, 0.20 mmol) and *O*-benzoyl-*N*,*N*-dibenzylhydroxylamine (**4a**, 95.2 mg, 0.30 mmol), purification by flash column chromatography (*n*-hexane/EtOAc 100/1) afforded **5i** (55.3 mg, 73% yield) as a light-yellow oil. In an independent experiment, 57.7 mg (76% yield) were obtained, giving an average yield

of 74%. **^{1}H NMR** (400 MHz, $CDCl_3$) δ 7.44 – 7.38 (m, 4H), 7.34 – 7.27 (m, 4H), 7.25 – 7.19 (m, 2H), 4.28 (dd, *J* = 10.5, 6.5 Hz, 1H), 4.13 (dd, *J* = 10.5, 4.8 Hz, 1H), 3.83 (d, *J* = 13.8 Hz, 2H), 3.69 (d, *J* = 13.8 Hz, 2H), 2.90 – 2.81 (m, 1H), 2.41 – 2.28 (m, 2H), 2.22 – 2.15 (m, 2H), 1.72 – 1.53 (m, 5H), 1.53 – 1.42 (m, 1H), 1.04 – 0.92 (m, 9H) ppm. **^{13}C NMR** (101 MHz, $CDCl_3$) δ 161.0, 141.0, 128.9, 128.1, 126.7, 73.7, 58.2, 54.3, 36.3, 30.5, 22.2, 20.1, 19.5, 14.6, 14.0, 11.9 ppm. **IR** (neat, cm^{-1}): 3027, 2960, 2871, 1494, 1454, 1362, 1248, 1150, 1028, 937, 905, 742, 696. **HRMS** (ESI+): m/z *calcd.* for ($C_{25}H_{37}N_2O$) $[M+H]^+$: 381.2900; *found* 381.2908.

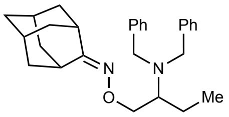

(1*r*,3*r*,5*R*,7*S*)-adamantan-2-one *O*-(2-(dibenzylamino)butyl) oxime (5j). Following general procedure G, using adamantan-2-one *O*-but-3-en-1-yl oxime (**1l**, 43.9 mg, 0.20 mmol) and *O*-benzoyl-*N*,*N*-dibenzylhydroxylamine (**4a**, 95.2 mg, 0.30 mmol). ^{1}H NMR and GC-FID analysis of the crude showed a rr of 8:1 (rr refers to the ratio of the major regioisomer to all other regioisomers). Purification by flash column chromatography (*n*-hexane/EtOAc 60/1) afforded **5j** (56.8 mg, 68% yield) as a light-yellow solid. In an independent experiment, 49.3 mg (59% yield) were obtained, giving an average yield of 64%. **^{1}H NMR** (400 MHz, $CDCl_3$) δ 7.45 – 7.40 (m, 4H), 7.34 – 7.28 (m, 4H), 7.25 – 7.20 (m, 2H), 4.30 (dd, *J* = 10.7, 6.7 Hz, 1H), 4.14 (dd, *J* = 10.7, 4.7 Hz, 2H), 3.83 (d, *J* = 13.8 Hz, 2H), 3.70 (d, *J* = 13.8 Hz, 1H), 3.63 – 3.57 (m, 1H), 2.91 – 2.82 (m, 1H), 2.62 (s, 1H), 2.07 – 1.83 (m, 12H), 1.69 – 1.56 (m, 1H), 1.52 – 1.40 (m, 1H), 0.95 (t, *J* = 7.4 Hz, 3H) ppm. **^{13}C NMR** (101 MHz, $CDCl_3$) δ 166.3, 141.0, 128.9, 128.1, 126.7, 73.7, 58.2, 54.3, 39.2, 39.1, 37.8, 37.7, 36.7, 36.4, 30.0, 28.0, 28.0, 22.1, 11.9 ppm. **IR** (neat, cm^{-1}): 3025, 2915, 2851, 1493, 1449, 1358, 1144, 1093, 1060, 993, 889, 821, 740, 695. **HRMS** (ESI+): m/z *calcd.* for ($C_{28}H_{37}N_2O$) $[M+H]^+$: 417.2900; *found* 417.2905. **MP**: 75 – 77 ºC.

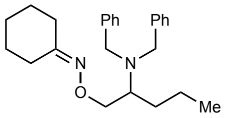

cyclohexanone *O*-(2-(dibenzylamino)pentyl) oxime (5k). Following general procedure G, using cyclohexanone *O*-pent-4-en-1-yl oxime (**1m**, 39.1 mg, 0.20 mmol) and *O*-benzoyl-*N*,*N*-dibenzylhydroxylamine (**4a**, 95.2 mg, 0.30 mmol), purification by flash column chromatography (*n*-hexane/EtOAc 50/1) afforded **5k** (53.0 mg, 70%) as a light-yellow oil. In an independent experiment, 54.6 mg (72% yield) were obtained, giving an average yield of 71%. **^{1}H NMR** (400 MHz, $CDCl_3$) δ 7.43 – 7.37 (m, 4H), 7.33 – 7.27 (m, 4H), 7.25 – 7.19 (m, 2H), 4.28 (dd, *J* = 10.8, 6.8 Hz, 1H), 4.09 (dd, *J* = 10.6, 4.8 Hz, 1H), 3.80 (d, *J* = 13.8 Hz, 2H), 3.67 (d, *J* = 13.8 Hz, 2H), 3.00 – 2.89 (m, 1H), 2.61 – 2.47 (m, 2H), 2.25 (t, *J* = 6.3 Hz, 2H), 1.78 – 1.43 (m, 8H), 1.39 – 1.22 (m, 2H), 0.80 (t, *J* = 7.1 Hz, 3H) ppm. **^{13}C NMR** (101 MHz, $CDCl_3$) δ 159.9, 141.0, 129.0, 128.1, 126.7, 74.0, 56.0, 54.3, 32.3, 31.5, 27.2, 26.0, 25.9, 25.8, 20.1, 14.3 ppm. **IR** (neat, cm^{-1}): 3027, 2929, 2858, 1494, 1451, 1362, 1150, 1027, 917, 879, 838, 744, 696. **HRMS** (ESI+): m/z *calcd.* for ($C_{25}H_{35}N_2O$) $[M+H]^+$: 379.2744; *found* 379.2742.

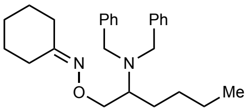

cyclohexanone *O*-(2-(dibenzylamino)hexyl) oxime (5l). Following general procedure G, using cyclohexanone *O*-hex-5-en-1-yl oxime (**1n**, 41.9 mg, 0.20 mmol) and *O*-benzoyl-*N*,*N*-dibenzylhydroxylamine (**4a**, 95.2 mg, 0.30 mmol), purification by flash column chromatography (*n*-hexane/EtOAc 50/1) afforded **5l** (40.8 mg, 52% yield) as a light-yellow oil. In an independent experiment, 37.6 mg (48% yield) were obtained, giving an average yield of 50%. Following general procedure G, using (*E*)-cyclohexanone *O*-hex-4-en-1-yl oxime (**1t**, 41.9 mg, 0.20 mmol) and *O*-benzoyl-*N*,*N*-dibenzylhydroxylamine (**4a**, 95.2 mg, 0.30 mmol), purification by flash column chromatography (*n*-hexane/EtOAc 50/1) afforded **5l** (47.5 mg, 60% yield) as a light-yellow oil. In an independent experiment, 49.8 mg (63% yield) were obtained, giving an average yield of 62%. Following general procedure G, using (*E*)-cyclohexanone *O*-hex-3-en-1-yl oxime (**1r**, 41.9 mg, 0.20 mmol) and *O*-benzoyl-*N*,*N*-dibenzylhydroxylamine (**4a**, 95.2 mg, 0.30 mmol), purification by flash column chromatography (*n*-hexane/EtOAc 50/1) afforded **5l** (48.1 mg, 61% yield) as a light-yellow oil. In an independent experiment, 51.6 mg (66% yield) were obtained, giving an average yield of 63%. **^{1}H NMR** (400 MHz, $CDCl_3$) δ 7.43 – 7.37 (m, 4H), 7.33 – 7.27 (m, 4H), 7.25 – 7.19 (m, 2H), 4.28 (dd, *J* = 10.6, 6.8 Hz, 1H), 4.09 (dd, *J* = 10.6, 4.8 Hz, 1H), 3.80 (d, *J* = 13.8 Hz, 2H), 3.67 (d, *J* = 13.7 Hz, 2H), 2.96 – 2.88 (m, 1H), 2.61 – 2.47 (m, 2H), 2.28 – 2.21 (m, 2H), 1.76 – 1.54 (m, 7H), 1.50 – 1.30 (m, 2H), 1.28 – 1.11 (m, 3H), 0.86 (t, *J* = 7.2 Hz, 3H) ppm. **^{13}C NMR** (101 MHz, $CDCl_3$) δ 159.9, 141.0, 129.0, 128.1, 126.7, 74.0, 56.2, 54.3, 32.3, 29.1, 28.8, 27.2, 26.0, 25.9, 25.8, 22.8, 14.2 ppm. **IR** (neat, cm^{-1}): 3027, 2928, 2857, 1494, 1451, 1362, 1254, 1148, 1106, 1027, 931, 885, 839, 744, 696. **HRMS** (ESI+): m/z *calcd.* for ($C_{26}H_{37}N_2O$) $[M+H]^+$: 393.2900; *found* 393.2900.

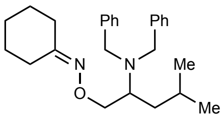

cyclohexanone *O*-(2-(dibenzylamino)-4-methylpentyl) oxime (5m). Following general procedure G, using cyclohexanone *O*-(4-methylpent-3-en-1-yl) oxime (**1v**, 41.9 mg, 0.20 mmol) and *O*-benzoyl-*N*,*N*-dibenzylhydroxylamine (**4a**, 95.2 mg, 0.30 mmol), purification by flash column chromatography (*n*-hexane/EtOAc 50/1) afforded **5m** (26.8 mg, 34% yield) as a light-yellow oil. In an independent experiment, 25.0 mg (32% yield) were obtained, giving an average yield of 33%. **^{1}H NMR** (400 MHz, $CDCl_3$) δ 7.41 – 7.35 (m, 4H), 7.32 – 7.26 (m, 4H), 7.24 – 7.17 (m, 2H), 4.27 (dd, *J* = 10.7, 6.9 Hz, 1H), 4.05 (dd, *J* = 10.7, 4.9 Hz, 1H), 3.76 (d, *J* = 13.6 Hz, 2H), 3.64 (d, *J* = 13.6 Hz, 2H), 3.04 – 2.94 (m, 1H), 2.61 – 2.46 (m, 2H), 2.24 (t, *J* = 6.3 Hz, 2H), 1.87 – 1.58 (m, 7H), 1.54 – 1.44 (m, 1H), 1.16 – 1.07 (m, 1H), 0.83 (d, *J* = 6.7 Hz, 3H), 0.58 (d, *J* = 6.5 Hz, 3H) ppm. **^{13}C NMR** (101 MHz, $CDCl_3$) δ 160.0, 141.0, 129.1, 128.1, 126.8, 74.3, 54.3, 54.1, 38.7, 32.4, 27.2, 26.0, 25.9, 25.8, 24.8, 23.5, 22.3 ppm. **IR** (neat, cm^{-1}): 3027, 2959, 2928, 2872, 1493, 1453, 1362, 1250, 1148, 1097, 1028, 972, 885, 743, 696. **HRMS** (ESI+): m/z *calcd.* for ($C_{26}H_{37}N_2O$) $[M+H]^+$: 393.2900; *found* 393.2914.

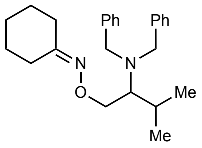

cyclohexanone *O*-(2-(dibenzylamino)-4-methylpentyl) oxime (5n). Following general procedure G, using cyclohexanone *O*-(3-methylbut-3-en-1-yl) oxime (**1w**, 39.1 mg, 0.20 mmol) and *O*-benzoyl-*N*,*N*-dibenzylhydroxylamine (**4a**, 95.2 mg, 0.30 mmol), purification by flash column chromatography (*n*-hexane/EtOAc 80/1) afforded **5n** (30.1 mg, 40% yield) as a light-yellow oil. In an independent experiment, 33.7 mg (45% yield) were obtained, giving an average yield of 42%. ^{1}**H NMR** (400 MHz, $CDCl_3$) δ 7.42 – 7.36 (m, 4H), 7.33 – 7.27 (m, 4H), 7.25 – 7.19 (m, 2H), 4.37 – 4.26 (m, 2H), 3.91 (d, *J* = 13.7 Hz, 2H), 3.59 (d, *J* = 13.8 Hz, 2H), 2.58 – 2.42 (m, 3H), 2.32 – 2.22 (m, 2H), 2.00 – 1.87 (m, 1H), 1.76 –1.56 (m, 6H), 1.02 (d, *J* = 6.7 Hz, 3H), 0.88 (d, *J* = 6.6 Hz, 3H) ppm. ^{13}**C NMR** (101 MHz, $CDCl_3$) δ 160.2, 140.9, 129.2, 128.1, 126.7, 71.4, 62.5, 55.0, 32.4, 28.4, 27.3, 26.0, 25.9, 21.4, 20.5 ppm. **IR** (neat, cm^{-1}): 2922, 2853, 1454, 1377, 1064, 745, 698. **HRMS** (ESI+): m/z *calcd.* for ($C_{25}H_{35}N_2O$) $[M+H]^+$: 379.2744; *found* 379.2754.

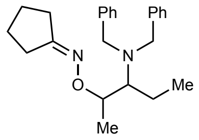

cyclopentanone *O*-(3-(dibenzylamino)pentan-2-yl) oxime (5o). Following general procedure G, using cyclopentanone *O*-pent-4-en-2-yl oxime (**1y**, 33.5 mg, 0.20 mmol) and *O*-benzoyl-*N*,*N*-dibenzylhydroxylamine (**4a**, 95.2 mg, 0.30 mmol), purification by flash column chromatography (*n*-hexane/Acetone 120/1) afforded **5o** (46.8 mg, 64% yield, dr 8:1) as a light-yellow oil. In an independent experiment, 41.1 mg (56% yield) were obtained, giving an average yield of 60%. ^{1}**H NMR** (400 MHz, $CDCl_3$) δ 7.42 – 7.37 (m, 4H), 7.34 – 7.29 (m, 4H), 7.26 – 7.21 (m, 2H), 4.63 – 4.56 (m, 1H), 3.86 (d, *J* = 13.9 Hz, 2H), 3.60 (d, *J* = 13.7 Hz, 2H), 2.53 – 2.30 (m, 5H), 1.82 – 1.70 (m, 5H), 1.65 – 1.54 (m, 1H), 1.24 – 1.20 (m, 3H), 1.05 – 0.98 (m, 3H) ppm. ^{13}**C NMR** (101 MHz, $CDCl_3$) δ 165.9, 140.8, 129.0, 128.2, 126.7, 77.6, 63.0, 54.4, 31.1, 28.1, 25.3, 24.8, 19.6, 18.7, 12.8 ppm. **IR** (neat, cm^{-1}): 3025, 2973, 2822, 1493, 1452, 1380, 1087, 1045, 880. **HRMS** (ESI+): m/z *calcd.* for ($C_{24}H_{33}N_2O$) $[M+H]^+$: 365.2587; *found* 365.2597.

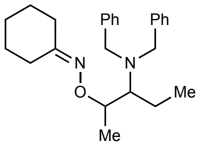

cyclohexanone *O*-(3-(dibenzylamino)pentan-2-yl) oxime (5p). Following general procedure G, using cyclohexanone *O*-pent-4-en-2-yl oxime (**1x**, 39.1 mg, 0.20 mmol) and *O*-benzoyl-*N*,*N*-dibenzylhydroxylamine (**4a**, 95.2 mg, 0.30 mmol), purification by flash column chromatography (*n*-hexane/Acetone 150/1) afforded **5p** (54.2 mg, 72% yield, dr 12:1) as a light-yellow oil. In an independent experiment, 57.3 mg (76% yield) were obtained, giving an average yield of 74%. ^{1}**H NMR** (400 MHz, $CDCl_3$) δ 7.40 – 7.34 (m, 4H), 7.32 – 7.26 (m, 4H), 7.24 – 7.18 (m, 2H), 4.60 – 4.52 (m, 1H), 3.84 (d, *J* = 13.9 Hz, 2H), 3.55 (d, *J* = 14.0 Hz, 2H), 2.55 – 2.32 (m, 3H), 2.30 – 2.16 (m, 2H), 1.80 – 1.63 (m, 3H), 1.63 – 1.46 (m, 5H), 1.18 (d, *J* = 6.4 Hz, 3H), 0.98 (t, *J* =

7.4 Hz, 3H) ppm. **^{13}C NMR** (101 MHz, $CDCl_3$) δ 159.9, 140.9, 129.0, 128.2, 126.7, 63.1, 54.4, 32.5, 27.4, 26.1, 26.1, 25.8, 19.4, 18.7, 12.8 ppm. **IR** (neat, cm^{-1}): 3027, 2930, 2856, 1494, 1450, 1366, 1254, 1104, 1059, 935, 919, 841, 743, 697. **HRMS** (ESI+): m/z *calcd.* for ($C_{25}H_{35}N_2O$) $[M+H]^+$: 379.2744; *found* 379.2752.

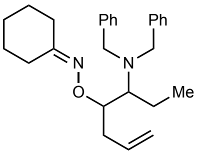

cyclohexanone *O*-(5-(dibenzylamino)hept-1-en-4-yl) oxime (5q). Following general procedure G, using cyclohexanone *O*-hepta-1,6-dien-4-yl oxime (**1z**, 41.4 mg, 0.20 mmol) and *O*-benzoyl-*N*,*N*-dibenzylhydroxylamine (**4a**, 95.2 mg, 0.30 mmol), purification by flash column chromatography (*n*-hexane/Acetone 150/1) afforded **5q** (26.6 mg, 33% yield) as a light-yellow oil. In an independent experiment, 22.4 mg (28% yield) were obtained, giving an average yield of 30%. **^{1}H NMR** (400 MHz, $CDCl_3$) δ 7.37 – 7.31 (m, 4H), 7.31 – 7.24 (m, 4H), 7.23 – 7.16 (m, 2H), 5.58 – 5.56 (m, 1H), 4.95 – 4.85 (m, 2H), 4.50 – 4.44 (m, 1H), 3.83 (d, *J* = 13.9 Hz, 2H), 3.50 (d, *J* = 13.9 Hz, 2H), 2.55 – 2.32 (m, 3H), 2.41 – 2.32 (m, 1H), 2.39 – 2.15 (m, 3H), 1.76 – 1.64 (m, 3H), 1.62 – 1.46 (m, 5H), 0.96 (t, *J* = 7.4 Hz, 3H) ppm. **^{13}C NMR** (101 MHz, $CDCl_3$) δ 160.0, 140.9, 135.1, 129.2, 128.1, 126.7, 117.0, 80.2, 60.5, 54.4, 37.8, 32.5, 27.5, 26.1, 26.1, 25.8, 18.6, 12.6 ppm. **IR** (neat, cm^{-1}): 3027, 2928, 2856, 1661, 1640, 1494, 1451, 1363, 1253, 1027, 992, 936, 917, 841, 744, 698. **HRMS** (ESI+): m/z *calcd.* for ($C_{27}H_{37}N_2O$) $[M+H]^+$: 405.2900; *found* 405.2907.

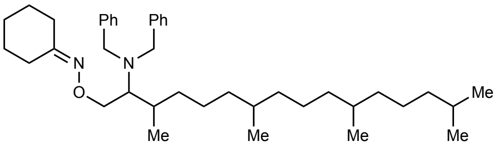

cyclohexanone *O*-(5-(dibenzylamino)hept-1-en-4-yl) oxime (5r). Following general procedure G, using (*E*)-cyclohexanone *O*-(3,7,11,15-tetramethylhexadec-2-en-1-yl) oxime (**1aa**, 78.3 mg, 0.20 mmol) and *O*-benzoyl-*N*,*N*-dibenzylhydroxylamine (**4a**, 95.2 mg, 0.30 mmol), purification by flash column chromatography (*n*-hexane/EtOAc 100/1) afforded **5q** (44.6 mg, 38% yield) as a light-yellow oil. In an independent experiment, 49.0 mg (42% yield) were obtained, giving an average yield of 40%. **^{1}H NMR** (400 MHz, $CDCl_3$) δ 7.38 – 7.33 (m, 4H), 7.31 – 7.26 (m, 4H), 7.23 – 7.18 (m, 2H), 4.36 – 4.24 (m, 2H), 3.87 (d, *J* = 13.6 Hz, 2H), 3.58 (d, *J* = 13.7 Hz, 2H), 2.62 – 2.54 (m, 1H), 2.57 – 2.49 (m, 2H), 2.29 – 2.23 (m, 2H), 1.82 – 1.67 (m, 4H), 1.69 – 1.57 (m, 4H), 1.61 – 1.46 (m, 1H), 1.39 – 0.99 (m, 19H), 0.88 (s, 3H), 0.88 – 0.81 (m, 12H) ppm. **^{13}C NMR** (101 MHz, $CDCl_3$) δ 140.9, 129.2, 128.1 126.7, 71.5, 60.7, 55.1, 39.5, 37.8, 37.7, 37.65, 37.59, 37.5, 34.3, 33.3, 33.2, 33.0, 32.9, 32.4, 28.1, 27.3, 26.0, 25.9, 25.0, 24.7, 24.7, 24.2, 24.1, 22.9, 22.8, 19.9, 19.8, 16.8, 16.8 ppm. **IR** (neat, cm^{-1}): 3063, 2925, 2856, 1494, 1454, 1377, 1028, 935, 746, 698. **LCMS** (ESI+): m/z *calcd.* for ($C_{40}H_{65}N_2O$) $[M+H]^+$: 589.5; *found* 588.9.

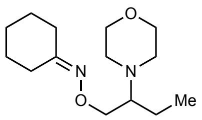

cyclohexanone *O*-(2-morpholinobutyl) oxime (5s). Following general procedure G, using cyclohexanone *O*-but-3-en-1-yl oxime (**1a**, 33.5 mg, 0.20 mmol) and morpholino benzoate (**4e**, 62.2 mg, 0.30 mmol), purification by flash column chromatography (*n*-hexane/EtOAc 6/1) afforded **5r** (35.2 mg, 69% yield) as a yellow oil. In an independent experiment, 36.3 mg (71% yield) were obtained, giving an average yield of 70%. **^{1}H NMR** (400 MHz, $CDCl_3$) δ 4.14 (dd, *J* = 10.8, 6.6 Hz, 1H), 3.99 (dd, *J* = 10.8, 4.4 Hz, 1H), 3.72 – 3.60 (m, 4H), 2.75 – 2.67 (m, 2H), 2.63 – 2.51 (m, 3H), 2.51 – 2.38 (m, 2H), 2.22 – 2.15 (m, 2H), 1.70 – 1.55 (m, 6H), 1.51 – 1.39 (m, 2H), 0.95 (t, *J* = 7.4 Hz, 3H) ppm. **^{13}C NMR** (101 MHz, $CDCl_3$) δ 160.3, 73.2, 67.9, 64.6, 50.0, 32.3, 27.2, 26.0, 25.9, 25.7, 21.2, 11.4 ppm. **IR** (neat, cm^{-1}): 3046, 1679, 1519, 1483, 1423, 1383, 1325, 1260, 1165, 994, 921, 825, 789, 682. **HRMS** (ESI+): m/z *calcd.* for ($C_{14}H_{27}N_2O_2$) $[M+H]^+$: 255.2067; *found* 255.2069.

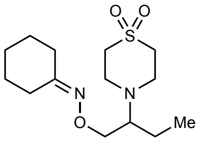

4-(1-((cyclohexylideneamino)oxy)butan-2-yl)thiomorpholine 1,1-dioxide (5t). Following general procedure G, using cyclohexanone *O*-but-3-en-1-yl oxime (**1a**, 33.5 mg, 0.20 mmol) and 1,1-dioxidothiomorpholino benzoate (**4k**, 76.6 mg, 0.30 mmol), purification by flash column chromatography (*n*-hexane/EtOAc 1/1) afforded **5s** (50.3 mg, 83% yield) as a light-yellow oil. In an independent experiment, 47.6 mg (79% yield) were obtained, giving an average yield of 81%. **^{1}H NMR** (500 MHz, $CDCl_3$) δ 4.03 – 3.93 (m, 2H), 3.24 – 3.16 (m, 2H), 3.05 – 2.90 (m, 6H), 2.86 – 2.77 (m, 1H), 2.44 – 2.38 (m, 2H), 2.16 (t, *J* = 6.3 Hz, 2H), 1.69 – 1.52 (m, 6H), 1.43 – 1.32 (m, 2H), 0.92 (t, *J* = 7.4 Hz, 3H) ppm. **^{13}C NMR** (101 MHz, $CDCl_3$) δ 160.6, 73.6, 65.1, 53.0, 47.4, 32.2, 27.1, 25.8, 25.8, 25.6, 22.3, 11.5 ppm. **IR** (neat, cm^{-1}): 2929, 2857, 1449, 1331, 1302, 1270, 1188, 1123, 1039, 989, 933, 858, 719. **HRMS** (ESI+): m/z *calcd.* for ($C_{14}H_{27}N_2O_3S$) $[M+H]^+$: 303.1737; *found* 303.1747.

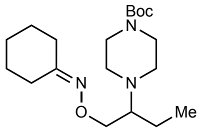

***tert*-butyl 4-(1-((cyclohexylideneamino)oxy)butan-2-yl)piperazine-1-carboxylate (5u).** Following general procedure G, using cyclohexanone *O*-but-3-en-1-yl oxime (**1a**, 33.5 mg, 0.20 mmol) and tert-butyl 4-(benzoyloxy)piperazine-1-carboxylate (**4g**, 91.6 mg, 0.30 mmol), purification by flash column chromatography (*n*-hexane/EtOAc 5/1) afforded **5t** (56.7 mg, 80% yield) as a yellow oil. In an independent experiment, 59.3 mg (84% yield) were obtained, giving an average yield of 82%. **^{1}H NMR** (400 MHz, $CDCl_3$) δ 4.08 (ddd, *J* = 10.9, 6.8, 1.3 Hz, 1H), 3.95 (ddd, *J* = 10.8, 4.4, 1.3 Hz, 1H), 3.41 – 3.27 (m, 4H), 2.70 – 2.57 (m, 3H), 2.52 – 2.36 (m, 4H), 2.20 – 2.12 (m, 2H), 1.68 – 1.52 (m, 6H), 1.46 – 1.37 (m, 11H), 0.92 (td, *J* = 7.4, 1.3 Hz, 3H) ppm. **^{13}C NMR** (101 MHz, $CDCl_3$) δ 160.2, 155.0, 79.4, 73.2, 64.5, 49.2, 32.3, 28.5, 27.2, 25.9, 25.9, 25.6, 22.1, 11.5

ppm. **IR** (neat, cm^{-1}): 2930, 2858, 1451, 1419, 1364, 1244, 1167, 1115, 1048, 1002, 934, 869. **HRMS** (ESI+): m/z *calcd.* for ($C_{19}H_{36}N_3O_3$) $[M+H]^+$: 354.2751; *found* 354.2760.

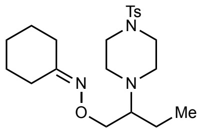

cyclohexanone *O*-(2-(4-tosylpiperazin-1-yl)butyl) oxime (5v). Following general procedure G, using cyclohexanone *O*-but-3-en-1-yl oxime (**1a**, 33.5 mg, 0.20 mmol) and 4-tosylpiperazin-1-yl benzoate (**4f**, 108.1 mg, 0.30 mmol), purification by flash column chromatography (*n*-hexane/EtOAc 5/1) afforded **5u** (54.8 mg, 67% yield) as a light-yellow oil. In an independent experiment, 58.9 mg (72% yield) were obtained, giving an average yield of 70%. **^{1}H NMR** (400 MHz, $CDCl_3$) δ 7.64 – 7.59 (m, 2H), 7.33 – 7.28 (m, 2H), 4.05 (dd, *J* = 11.0, 7.0 Hz, 1H), 3.95 (dd, *J* = 11.0, 4.1 Hz, 1H), 3.07 – 2.86 (m, 4H), 2.83 – 2.73 (m, 2H), 2.68 – 2.54 (m, 3H), 2.42 (s, 3H), 2.43 – 2.37 (m, 2H), 2.16 (t, *J* = 6.2 Hz, 2H), 1.75 – 1.54 (m, 6H), 1.43 – 1.32 (m, 2H), 0.85 (t, *J* = 7.4 Hz, 3H) ppm. **^{13}C NMR** (101 MHz, $CDCl_3$) δ 160.4, 143.6, 132.8, 129.7, 128.0, 73.1, 64.4, 48.6, 47.0, 32.3, 27.2, 25.9, 25.7, 22.0, 21.6, 11.5 ppm. **IR** (neat, cm^{-1}): 2929, 2855, 1597, 1451, 1350, 1327, 1166, 1112, 1046, 945, 816, 731, 652. **HRMS** (ESI+): m/z *calcd.* for ($C_{21}H_{34}N_3O_3S$) $[M+H]^+$: 408.2315; *found* 408.2321.

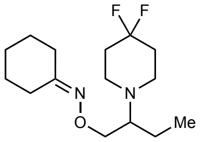

cyclohexanone *O*-(2-(4,4-difluoropiperidin-1-yl)butyl) oxime (5w). Following general procedure G, using cyclohexanone *O*-but-3-en-1-yl oxime (**1a**, 33.5 mg, 0.20 mmol) and 4,4-difluoropiperidin-1-yl benzoate (**4j**, 72.4 mg, 0.30 mmol), purification by flash column chromatography (*n*-hexane/EtOAc 50/1) afforded **5v** (44.5 mg, 77% yield) as a colorless oil. In an independent experiment, 43.4 mg (75% yield) were obtained, giving an average yield of 76%. **^{1}H NMR** (400 MHz, $CDCl_3$) δ 4.08 (dd, *J* = 10.9, 7.1 Hz, 1H), 3.97 (dd, *J* = 10.9, 4.5 Hz, 1H), 2.85 – 2.76 (m, 2H), 2.76 – 2.69 (m, 1H), 2.66 – 2.57 (m, 2H), 2.46 – 2.39 (m, 2H), 2.21 – 2.15 (m, 2H), 2.01 – 1.81 (m, 4H), 1.70 – 1.54 (m, 6H), 1.46 – 1.37 (m, 2H), 0.93 (t, *J* = 7.4 Hz, 3H) ppm. **^{13}C NMR** (101 MHz, $CDCl_3$) δ 160.3, 122.7 (t, *J* = 241.3 Hz), 73.5, 64.1, 46.0 (t, *J* = 5.4 Hz), 35.1 (t, *J* = 22.1 Hz), 32.3, 27.2, 26.0, 25.9, 25.7, 22.4, 11.6 ppm. **^{19}F NMR** (376 MHz, $CDCl_3$) δ -97.3 ppm. **IR** (neat, cm^{-1}): 2932, 2859, 1449, 1358, 1316, 1223, 1121, 1090, 1064, 1045, 993, 949, 871, 839. **HRMS** (ESI+): m/z *calcd.* for ($C_{15}H_{27}F_2N_2O$) $[M+H]^+$: 289.2086; *found* 289.2095.

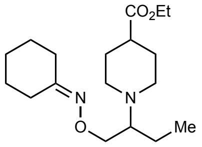

ethyl 1-(1-((cyclohexylideneamino)oxy)butan-2-yl)piperidine-4-carboxylate (5x). Following general procedure G, using cyclohexanone *O*-but-3-en-1-yl oxime (**1a**, 33.5 mg, 0.20 mmol) and ethyl 1-(benzoyloxy)piperidine-4-carboxylate (**4m**, 83.2 mg, 0.30 mmol), purification by flash column chromatography

(*n*-hexane/EtOAc 5/1) afforded **5w** (48.2 mg, 74% yield) as a colorless oil. In an independent experiment, 49.4 mg (77% yield) were obtained, giving an average yield of 75%. **^{1}H NMR** (400 MHz, $CDCl_3$) δ 4.18 – 4.02 (m, 3H), 3.94 (dd, *J* = 10.7, 4.6 Hz, 1H), 2.90 – 2.71 (m, 2H), 2.67 – 2.52 (m, 2H), 2.48 – 2.35 (m, 2H), 2.31 – 2.13 (m, 4H), 1.88 – 1.77 (m, 2H), 1.74 – 1.52 (m, 8H), 1.47 – 1.35 (m, 2H), 1.22 (t, *J* = 7.1 Hz, 3H), 0.91 (t, *J* = 7.4 Hz, 3H) ppm. **^{13}C NMR** (101 MHz, $CDCl_3$) δ 175.6, 160.1, 73.2, 64.6, 60.2, 51.5, 46.6, 42.0, 32.3, 29.4, 29.2, 27.2, 25.95, 25.91, 25.6, 22.4, 14.3, 11.6 ppm. **IR** (neat, cm^{-1}): 2929, 2858, 1732, 1448, 1285, 1260, 1175, 1155, 1044, 989, 934, 871. **HRMS** (ESI+): m/z *calcd.* for ($C_{18}H_{33}N_2O_3$) $[M+H]^+$: 325.2486; *found* 325.2500.

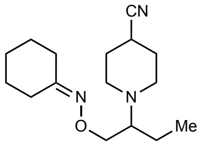

1-(1-((cyclohexylideneamino)oxy)butan-2-yl)piperidine-4-carbonitrile (5y). Following general procedure G, using cyclohexanone *O*-but-3-en-1-yl oxime (**1a**, 33.5 mg, 0.20 mmol) and 4-cyanopiperidin-1-yl benzoate (**4l**, 69.1 mg, 0.30 mmol), purification by flash column chromatography (*n*-hexane/EtOAc 2/1) afforded **5x** (40.7 mg, 73% yield) as a light-yellow oil. In an independent experiment, 37.0 mg (67% yield) were obtained, giving an average yield of 70%. **^{1}H NMR** (400 MHz, $CDCl_3$) δ 4.07 (dd, *J* = 10.8, 7.1 Hz, 1H), 3.94 (dd, *J* = 10.8, 4.4 Hz, 1H), 2.92 – 2.83 (m, 1H), 2.77 – 2.68 (m, 1H), 2.68 – 2.53 (m, 3H), 2.48 – 2.36 (m, 3H), 2.16 (t, *J* = 6.2 Hz, 2H), 1.93 – 1.69 (m, 4H), 1.69 – 1.52 (m, 6H), 1.43 – 1.34 (m, 2H), 0.90 (t, *J* = 7.4 Hz, 3H) ppm. **^{13}C NMR** (101 MHz, $CDCl_3$) δ 160.3, 122.1, 73.3, 64.7, 47.0, 32.2, 29.8, 29.7, 27.1, 26.8, 25.9, 25.6, 22.1, 11.6 ppm. **IR** (neat, cm^{-1}): 2928, 2858, 2811, 2239, 1447, 1385, 1318, 1255, 1157, 1081, 1045, 989, 934, 870, 839. **HRMS** (ESI+): m/z *calcd.* for ($C_{16}H_{28}N_3O$) $[M+H]^+$: 278.2227; *found* 278.2234.

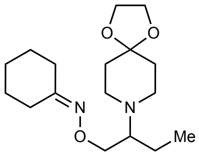

cyclohexanone *O*-(2-(1,4-dioxa-8-azaspiro[4.5]decan-8-yl)butyl) oxime (5z). Following general procedure G, using cyclohexanone *O*-but-3-en-1-yl oxime (**1a**, 33.5 mg, 0.20 mmol) and 1,4-dioxa-8-azaspiro[4.5]decan-8-yl benzoate (**4n**, 79.0 mg, 0.30 mmol), purification by flash column chromatography (*n*-hexane/EtOAc 3/1) afforded **5y** (48.4 mg, 78% yield) as a colorless oil. In an independent experiment, 45.1 mg (73% yield) were obtained, giving an average yield of 75%. **^{1}H NMR** (400 MHz, $CDCl_3$) δ 4.11 (dd, *J* = 10.7, 6.4 Hz, 1H), 3.95 (dd, *J* = 10.7, 4.7 Hz, 1H), 3.91 (s, 4H), 2.79 – 2.70 (m, 2H), 2.69 – 2.53 (m, 3H), 2.48 – 2.36 (m, 2H), 2.20 – 2.11 (m, 2H), 1.74 – 1.52 (m, 10H), 1.50 – 1.36 (m, 2H), 0.91 (t, *J* = 7.4 Hz, 3H) ppm. **^{13}C NMR** (101 MHz, $CDCl_3$) δ 160.1, 107.9, 73.3, 64.3, 64.2, 47.1, 35.8, 32.3, 27.2, 26.0, 25.9, 25.6, 22.3, 11.7 ppm. **IR** (neat, cm^{-1}): 2927, 2870, 1639, 1467, 1448, 1361, 1314, 1218, 1141, 1087, 1065, 1039, 944, 912, 872, 782. **HRMS** (ESI+): m/z *calcd.* for ($C_{17}H_{31}N_2O_3$) $[M+H]^+$: 311.2329; *found* 311.2341.

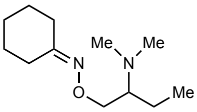

cyclohexanone *O*-(2-(dimethylamino)butyl) oxime (5aa). Following general procedure G, using cyclohexanone *O*-but-3-en-1-yl oxime (**1a**, 33.5 mg, 0.20 mmol) and *O*-benzoyl-*N,N*-dimethylhydroxylamine (**4c**, 49.6 mg, 0.30 mmol), purification by flash column chromatography (EtOAc/Et_3N 100/1) afforded **5z** (22.6 mg, 53% yield) as a light-yellow oil. In an independent experiment, 25.1 mg (59% yield) were obtained, giving an average yield of 56%. **^{1}H NMR** (400 MHz, $CDCl_3$) δ 4.15 (dd, *J* = 10.7, 6.3 Hz, 1H), 3.99 (dd, *J* = 10.7, 4.6 Hz, 1H), 2.64 – 2.56 (m, 1H), 2.47 – 2.41 (m, 2H), 2.34 (s, 6H), 2.21 – 2.16 (m, 2H), 1.70 – 1.62 (m, 2H), 1.62 – 1.54 (m, 4H), 1.53 – 1.40 (m, 2H), 0.94 (t, *J* = 7.4 Hz, 3H) ppm. **^{13}C NMR** (101 MHz, $CDCl_3$) δ 160.3, 72.6, 64.4, 41.7, 32.3, 27.2, 26.0, 25.9, 25.7, 22.1, 11.5 ppm. **IR** (neat, cm^{-1}): 2957, 2922, 2852, 1643, 1560, 1447, 1419, 1376, 1048. **HRMS** (ESI+): m/z *calcd.* for ($C_{12}H_{25}N_2O$) $[M+H]^+$: 213.1961; *found* 213.1958.

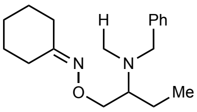

cyclohexanone *O*-(2-(benzyl(methyl)amino)butyl) oxime (5ab). Following general procedure G, using cyclohexanone *O*-but-3-en-1-yl oxime (**1a**, 33.5 mg, 0.20 mmol) and *O*-benzoyl-*N*-benzyl-*N*-methylhydroxylamine (**4d**, 72.4 mg, 0.30 mmol), purification by flash column chromatography (*n*-hexane/EtOAc 5/1) afforded **5aa** (42.0 mg, 73% yield) as a light-yellow oil. In an independent experiment, 40.2 mg (70% yield) were obtained, giving an average yield of 71%. **^{1}H NMR** (500 MHz, $CDCl_3$) δ 7.37 – 7.32 (m, 2H), 7.32 – 7.27 (m, 2H), 7.25 – 7.19 (m, 1H), 4.22 (d, *J* = 7.0 Hz, 1H), 4.05 (d, *J* = 4.7 Hz, 1H), 3.76 (d, *J* = 13.6 Hz, 1H), 3.66 (d, *J* = 13.7 Hz, 1H), 2.87 – 2.80 (m, 1H), 2.55 – 2.44 (m, 2H), 2.24 (s, 3H), 2.22 (t, *J* = 6.3 Hz, 2H), 1.74 – 1.57 (m, 6H), 1.57 – 1.42 (m, 2H), 1.01 (t, *J* = 7.4 Hz, 3H) ppm. **^{13}C NMR** (126 MHz, $CDCl_3$) δ 160.1, 128.7, 128.2, 126.7, 73.4, 63.4, 59.0, 37.1, 32.4, 27.2, 27.0, 26.0, 25.7, 22.4, 11.8 ppm. **IR** (neat, cm^{-1}): 2929, 2858, 2790, 1494, 1449, 1230, 1047, 991, 933, 917, 871, 840, 731, 697. **HRMS** (ESI+): m/z *calcd.* for ($C_{18}H_{29}N_2O$) $[M+H]^+$: 289.2274; *found* 289.2284.

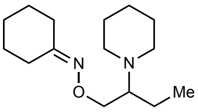

cyclohexanone *O*-(2-(piperidin-1-yl)butyl) oxime (5ac). Following general procedure G, using cyclohexanone *O*-but-3-en-1-yl oxime (**1a**, 33.5 mg, 0.20 mmol) and piperidin-1-yl benzoate (**4h**, 61.6 mg, 0.30 mmol), purification by flash column chromatography (EtOAc/Et_3N 100/1) afforded **5ab** (40.0 mg, 79% yield) as a light-yellow oil. In an independent experiment, 37.7 mg (75% yield) were obtained, giving an average yield of 77%. **^{1}H NMR** (400 MHz, $CDCl_3$) δ 4.15 (dd, *J* = 10.6, 6.2 Hz, 1H), 3.96 (dd, *J* = 10.6, 4.8 Hz, 1H), 2.69 – 2.54 (m, 3H), 2.50 – 2.38 (m, 4H), 2.21 – 2.14 (m, 2H), 1.70 – 1.36 (m, 14H), 0.93 (t, *J* = 7.4 Hz, 3H) ppm. **^{13}C NMR** (101 MHz, $CDCl_3$) δ 160.1, 73.1, 65.1, 50.6, 32.3, 27.2, 26.9, 26.0, 25.9, 25.7, 25.2, 22.5, 11.8 ppm. **IR** (neat, cm^{-1}): 3027, 2928, 2854, 1640, 1449, 1382, 1345, 1271, 1170, 1103, 1046, 990, 934, 872, 783. **HRMS** (ESI+): m/z *calcd.* for ($C_{15}H_{29}N_2O$) $[M+H]^+$: 253.2274; *found* 253.2285.

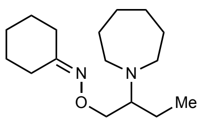

cyclohexanone *O*-(2-(azepan-1-yl)butyl) oxime (5ad). Following general procedure G, using cyclohexanone *O*-but-3-en-1-yl oxime (**1a**, 33.5 mg, 0.20 mmol) and azepan-1-yl benzoate (**4i**, 65.8 mg, 0.30 mmol), purification by flash column chromatography (*n*-hexane/EtOAc 2/1) afforded **5ac** (30.2 mg, 57% yield) as a light-yellow oil. In an independent experiment, 31.9 mg (60% yield) were obtained, giving an average yield of 58%. **^{1}H NMR** (400 MHz, $CDCl_3$) δ 4.10 (dd, *J* = 10.5, 6.8 Hz, 1H), 3.91 (dd, *J* = 10.5, 5.5 Hz, 1H), 2.88 – 2.57 (m, 5H), 2.52 – 2.37 (m, 2H), 2.24 – 2.14 (m, 2H), 1.74 – 1.50 (m, 14H), 1.44 – 1.33 (m, 2H), 0.95 (t, *J* = 7.4 Hz, 3H) ppm. **^{13}C NMR** (101 MHz, $CDCl_3$) δ 160.0, 74.1, 65.8, 51.9, 32.4, 30.4, 27.2, 26.0, 25.9, 25.6, 23.2, 11.8 ppm. **IR** (neat, cm^{-1}): 2923, 2855, 1640, 1448, 1355, 1237, 1146, 1105, 1064, 1041, 991, 934, 872, 839. **HRMS** (ESI+): m/z *calcd.* for ($C_{16}H_{31}N_2O$) $[M+H]^+$: 267.2431; *found* 267.2434.

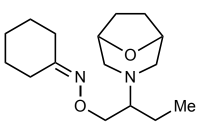

cyclohexanone *O*-(2-(8-oxa-3-azabicyclo[3.2.1]octan-3-yl)butyl) oxime (5ae). Following general procedure G, using cyclohexanone *O*-but-3-en-1-yl oxime (**1a**, 33.5 mg, 0.20 mmol) and 8-oxa-3-azabicyclo[3.2.1]octan-3-yl benzoate (**4q**, 70.0 mg, 0.30 mmol), purification by flash column chromatography (*n*-hexane/EtOAc 10/1) afforded **5ad** (29.6 mg, 53% yield) as a light-yellow oil. In an independent experiment, 26.4 mg (47% yield) were obtained, giving an average yield of 50%. **^{1}H NMR** (400 MHz, $CDCl_3$) δ 4.24 (s, 2H), 4.08 (dd, *J* = 10.6, 7.0 Hz, 1H), 3.93 (d, *J* = 5.0 Hz, 1H), 2.93 (dd, *J* = 10.9, 2.1 Hz, 1H), 2.66 (dd, *J* = 10.8, 2.1 Hz, 1H), 2.57 – 2.36 (m, 5H), 2.18 (t, *J* = 6.3 Hz, 2H), 1.96 – 1.75 (m, 4H), 1.73 – 1.53 (m, 6H), 1.46 – 1.32 (m, 2H), 0.94 (t, *J* = 7.4 Hz, 3H) ppm. **^{13}C NMR** (101 MHz, $CDCl_3$) δ 160.1, 75.5, 75.2, 73.4, 63.8, 57.0, 52.2, 32.3, 28.7, 28.5, 27.2, 26.0, 25.9, 25.6, 21.7, 11.4 ppm. **IR** (neat, cm^{-1}): 2931, 2859, 1643, 1449, 1225, 1200, 1163, 1063, 991, 920, 872, 839. **HRMS** (ESI+): m/z *calcd.* for ($C_{16}H_{29}N_2O_2$) $[M+H]^+$: 281.2224; *found* 281.2231.

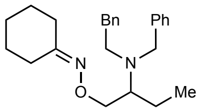

cyclohexanone *O*-(2-(benzyl(phenethyl)amino)butyl) oxime (5af). Following general procedure G, using cyclohexanone *O*-but-3-en-1-yl oxime (**1a**, 33.5 mg, 0.20 mmol) and *O*-benzoyl-*N*-benzyl-*N*-phenethylhydroxylamine (**4b**, 99.4 mg, 0.30 mmol), purification by flash column chromatography (*n*-hexane/acetone 80/1) afforded **5ae** (54.8 mg, 72% yield) as a light-yellow oil. In an independent experiment, 51.0 mg (67% yield) were obtained, giving an average yield of 70%. **^{1}H NMR** (400 MHz, $CDCl_3$) δ 7.39 – 7.33 (m, 2H), 7.33 – 7.26 (m, 2H), 7.26 – 7.19 (m, 3H), 7.19 – 7.15 (m, 1H), 7.13 – 7.07 (m, 2H), 4.21 (ddd, *J* = 10.7, 6.9, 1.3 Hz, 1H), 4.03 (ddd, *J* = 10.7, 5.1, 1.3 Hz, 1H), 3.90 (d, *J* = 14.2 Hz, 1H), 3.72 (d, *J* = 14.2 Hz, 1H), 2.96 – 2.88 (m, 1H), 2.88 – 2.78 (m, 2H), 2.78 – 2.61 (m, 2H), 2.54 – 2.42 (m, 2H), 2.22 (t, *J* = 6.3 Hz, 2H), 1.75 – 1.56 (m, 6H), 1.53 – 1.39 (m, 2H), 0.93 (td, *J* = 7.4, 1.2 Hz, 3H) ppm. **^{13}C NMR** (101 MHz, $CDCl_3$) δ 160.0, 141.5, 141.0, 128.9, 128.7, 128.3, 128.1, 126.6, 125.8, 74.2, 60.3, 55.2, 52.9, 36.2, 32.3, 27.2, 26.0,

25.9, 25.7, 22.7, 11.9 ppm. **IR** (neat, cm^{-1}): 3026, 2929, 2858, 1494, 1450, 1367, 1145, 1101, 1044, 990, 933, 871, 839, 731, 696. **HRMS** (ESI+): m/z *calcd.* for ($C_{25}H_{35}N_2O$) $[M+H]^+$: 379.2744; *found* 379.2753.

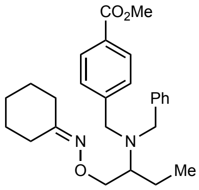

methyl 4-((benzyl(1-((cyclohexylideneamino)oxy)butan-2-yl)amino)methyl)benzoate (5ag). Following general procedure G, using cyclohexanone *O*-but-3-en-1-yl oxime (**1a**, 33.5 mg, 0.20 mmol) and methyl 4-(((benzoyloxy)(benzyl)amino)methyl)benzoate (**4r**, 112.6 mg, 0.30 mmol), purification by flash column chromatography (*n*-hexane/ EtOAc 10/1) afforded **5af** (61.1 mg, 72% yield) as a light-yellow oil. In an independent experiment, 65.7 mg (78% yield) were obtained, giving an average yield of 75%. **^{1}H NMR** (400 MHz, $CDCl_3$) δ 7.95 (d, *J* = 8.0 Hz, 2H), 7.45 (d, *J* = 8.0 Hz, 2H), 7.37 (d, *J* = 7.1 Hz, 2H), 7.28 (t, *J* = 7.8 Hz, 2H), 7.21 (t, *J* = 7.3 Hz, 1H), 4.24 (dd, *J* = 10.8, 6.9 Hz, 1H), 4.1 (dd, *J* = 10.8, 4.6 Hz, 1H), 3.9 (s, 3H), 3.8 – 3.6 (m, 4H), 2.8 (p, *J* = 6.8 Hz, 1H), 2.6 – 2.4 (m, 2H), 2.2 (t, *J* = 6.2 Hz, 2H), 1.7 – 1.6 (m, 2H), 1.7 – 1.5 (m, 5H), 1.5 – 1.4 (m, 1H), 0.9 (t, *J* = 7.4 Hz, 3H) ppm. **^{13}C NMR** (101 MHz, $CDCl_3$) δ 167.3, 160.0, 146.7, 140.6, 129.5, 128.9, 128.8, 128.7, 128.2, 126.9, 73.7, 58.5, 54.4, 54.2, 52.1, 32.3, 27.2, 26.0, 25.9, 25.8, 22.1, 11.9 ppm. **IR** (neat, cm^{-1}): 2930, 2858, 1720, 1610, 1494, 1434, 1274, 1172, 1106, 1019, 933, 869, 840, 756, 698. **HRMS** (ESI+): m/z *calcd.* for ($C_{26}H_{35}N_2O_3$) $[M+H]^+$: 423.2642; *found* 423.2648.

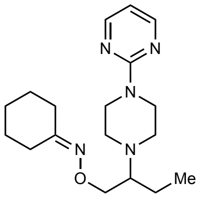

cyclohexanone *O*-(2-(4-(pyrimidin-2-yl)piperazin-1-yl)butyl) oxime (5ah). Following general procedure G, using cyclohexanone *O*-but-3-en-1-yl oxime (**1a**, 33.5 mg, 0.20 mmol) and 4-(pyrimidin-2-yl)piperazin-1-yl benzoate (**4o**, 85.3 mg, 0.30 mmol), purification by flash column chromatography (*n*-hexane/EtOAc 2/1) afforded **5ag** (53.8 mg, 81% yield) as a light-yellow oil. In an independent experiment, 52.4 mg (79% yield) were obtained, giving an average yield of 80%. **^{1}H NMR** (400 MHz, $CDCl_3$) δ 8.29 – 8.24 (m, 2H), 6.42 (t, *J* = 4.7 Hz, 1H), 4.13 (dd, *J* = 10.8, 6.7 Hz, 1H), 3.99 (dd, *J* = 10.8, 4.4 Hz, 1H), 3.82 – 3.68 (m, 4H), 2.81 – 2.64 (m, 3H), 2.64 – 2.56 (m, 2H), 2.47 – 2.33 (m, 2H), 2.19 – 2.12 (m, 2H), 1.68 – 1.41 (m, 8H), 0.96 (t, *J* = 7.4 Hz, 3H) ppm. **^{13}C NMR** (101 MHz, $CDCl_3$) δ 161.9, 160.2, 157.8, 109.6, 73.2, 64.6, 49.2, 44.7, 32.3, 27.2, 25.9, 25.9, 25.6, 22.1, 11.6 ppm. **IR** (neat, cm^{-1}): 2928, 2854, 1584, 1545, 1498, 1445, 1357, 1306, 1260, 1045, 981, 934, 912, 871, 796, 779. **HRMS** (ESI+): m/z *calcd.* for ($C_{18}H_{30}N_5O$) $[M+H]^+$: 332.2445; *found* 332.2452.

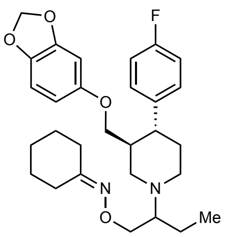

cyclohexanone *O*-(2-((3*S*,4*R*)-3-((benzo[*d*][1,3]dioxol-5-yloxy)methyl)-4-(4-fluorophenyl)piperidin-1-yl)butyl) oxime (5ai). Following general procedure G, using cyclohexanone *O*-but-3-en-1-yl oxime (**1a**, 33.5 mg, 0.20 mmol) and (3*S*,4*R*)-3-((benzo[d][1,3]dioxol-5-yloxy)methyl)-4-(4-fluorophenyl)piperi-din-1-yl benzoate (**4p**, 134.9 mg, 0.30 mmol), purification by flash column chromatography (*n*-hexane/acetone 5/1) afforded **5ah** (48.1 mg, 48% yield, dr 1:1) as a yellow oil. In an independent experiment, 51.1 mg (51% yield) were obtained, giving an average yield of 50%. **^{1}H NMR** (400 MHz, $CDCl_3$) δ 7.20 – 7.12 (m, 2H), 7.00 – 6.92 (m, 2H), 6.61 (dd, *J* = 8.5, 0.9 Hz, 1H), 6.33 (t, *J* = 2.5 Hz, 1H), 6.12 (dt, *J* = 8.5, 2.8 Hz, 1H), 5.86 (s, 2H), 4.20 (ddd, *J* = 10.8, 6.5, 2.4 Hz, 1H), 4.04 (dd, *J* = 10.7, 4.5 Hz, 1H), 3.58 – 3.51 (m, 1H), 3.47 – 3.38 (m, 1H), 3.26 – 3.14 (m, 1H), 2.99 – 2.87 (m, 1H), 2.81 – 2.62 (m, 2H), 2.52 – 2.28 (m, 4H), 2.23 – 2.04 (m, 3H), 1.88 – 1.44 (m, 10H), 0.99 (t, *J* = 7.4 Hz, 3H) ppm. **^{13}C NMR** (101 MHz, $CDCl_3$) δ 161.6 (d, *J* = 244.1 Hz), 160.25 (160.23), 154.7 (154.6), 148.2, 141.6 (141.5), 140.3 (d, *J* = 3.2 Hz), 128.9 (d, *J* = 7.7 Hz), 115.4 (d, *J* = 20.9 Hz), 107.9, 105.7 (105.6), 101.1, 98.1 (98.0), 73.2 (73.1), 70.1 (70.0), 64.9 (64.7), 56.8, 52.7, 51.4, 47.1, 44.9, 43.0 (42.8), 35.5 (35.3), 32.3, 27.2, 26.0 (25.9), 25.9, (25.9), 25.7, 22.5 (22.4), 11.8 (11.8) ppm. ^{19}F NMR (376 MHz, $CDCl_3$) δ -116.9 (d, *J* = 4.1 Hz) ppm. **IR** (neat, cm^{-1}): 2928, 2861, 2805, 1632, 1605, 1508, 1487, 1466, 1385, 1222, 1181, 1135, 1090, 1037, 990, 934, 831, 783. **HRMS** (ESI+): m/z *calcd.* for ($C_{29}H_{38}FN_2O_4$) $[M+H]^+$: 497.2810; *found* 497.2822.

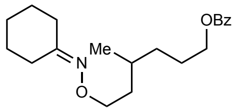

6-((cyclohexylideneamino)oxy)-4-methylhexyl benzoate (6a). Following general procedure F, using cyclohexanone *O*-but-3-en-1-yl oxime (**1a**, 40.1 mg, 0.24 mmol) and 3-iodopropyl benzoate (**2a**, 58.0 mg, 0.20 mmol). ^{1}H NMR and GC-FID analysis of the crude mixtures showed a rr of 8.6:1 (rr refers to the ratio of the major regioisomer to all other regioisomers). Purification by flash column chromatography (*n*-hexane/EtOAc 25/1) afforded **6a** (58.3 mg, 88% yield) as colorless oil. In an independent experiment, 59.7 mg (90% yield) were obtained, giving an average yield of 89%. **^{1}H NMR** (400 MHz, $CDCl_3$) δ 8.08 – 7.99 (m, 2H), 7.58 – 7.52 (m, 1H), 7.47 – 7.40 (m, 2H), 4.30 (t, *J* = 6.7 Hz, 2H), 4.12 – 3.97 (m, 2H), 2.48 – 2.40 (m, 2H), 2.25 – 2.16 (m, 2H), 1.86 – 1.69 (m, 3H), 1.70 – 1.61 (m, 3H), 1.63 – 1.57 (m, 4H), 1.56 – 1.42 (m, 2H), 1.42 – 1.26 (m, 1H), 0.95 (d, *J* = 6.5 Hz, 3H) ppm. **^{13}C NMR** (101 MHz, $CDCl_3$) δ 166.8, 160.2, 132.9, 130.7, 129.7, 128.9, 71.6, 65.5, 35.9, 33.4, 32.4, 30.0, 27.2, 26.4, 26.0, 25.9, 25.5, 19.8 ppm. **IR** (neat, cm^{-1}): 2930, 2858, 1718, 1450, 1380, 1314, 1271, 1109, 1069, 1026, 936, 711. **HRMS** (ESI+): m/z *calcd.* for ($C_{20}H_{30}NO_3$) $[M+H]^+$: 332.2220; *found* 332.2221.

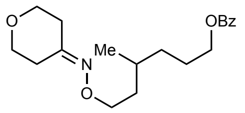

4-methyl-6-(((tetrahydro-4*H*-pyran-4-ylidene)amino)oxy)hexyl benzoate (6b). Following general procedure F, using tetrahydro-4*H*-pyran-4-one O-but-3-en-1-yl oxime (**1f**, 40.6 mg, 0.24 mmol) and 3-iodopropyl benzoate (**2a**, 58.0 mg, 0.20 mmol). ^{1}H NMR and GC-FID analysis of the crude mixtures showed a rr of 7:1 (rr refers to the ratio of the major regioisomer to all other regioisomers). Purification by flash column chromatography (*n*-hexane/EtOAc 15/1) afforded **6b** (56.0 mg, 84% yield) as colorless oil. In an independent experiment, 59.6 mg (89% yield) were obtained, giving an average yield of 86%. **^{1}H NMR** (400 MHz, $CDCl_3$) δ 8.06 – 8.02 (m, 2H), 7.57 – 7.52 (m, 1H), 7.48 – 7.39 (m, 2H), 4.31 (t, *J* = 6.7 Hz, 2H), 4.12 – 4.03 (m, 2H), 3.79 (t, *J* = 5.7 Hz, 2H), 3.71 (t, *J* = 5.8 Hz, 2H), 2.60 (t, *J* = 5.9 Hz, 2H), 2.42 – 2.28 (m, 2H), 1.87 – 1.69 (m, 3H), 1.68 – 1.60 (m, 1H), 1.54 – 1.44 (m, 2H), 1.38 – 1.24 (m, 1H), 0.96 (d, *J* = 6.5 Hz, 3H) ppm. **^{13}C NMR** (101 MHz, $CDCl_3$) δ 166.8, 154.9, 133.0, 130.6, 129.7, 128.5, 71.9, 68.5, 66.9, 65.4, 35.9, 33.4, 32.6, 29.9, 26.9, 26.4, 19.8 ppm. **IR** (neat, cm^{-1}): 2959, 2927, 2854, 1719, 1452, 1381, 1315, 1274, 1100, 850, 713. **HRMS** (ESI+): m/z *calcd.* for ($C_{19}H_{28}NO_4$) $[M+H]^+$: 334.2013; *found* 334.2022.

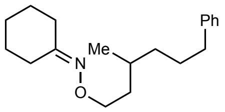

cyclohexanone *O*-(3-methyl-6-phenylhexyl) oxime (6c). Following general procedure F, using cyclohexanone *O*-but-3-en-1-yl oxime (**1a**, 40.1 mg, 0.24 mmol) and (3-iodopropyl)benzene (**2h**, 43.7 mg, 0.20 mmol). ^{1}H NMR and GC-FID analysis of the crude mixtures showed a rr of 7.6:1 (rr refers to the ratio of the major regioisomer to all other regioisomers). Purification by flash column chromatography (*n*-hexane/EtOAc 50/1) afforded **6c** (46.0 mg, 80% yield) as colorless oil. In an independent experiment, 49.4 mg (86% yield) were obtained, giving an average yield of 83%. **^{1}H NMR** (400 MHz, $CDCl_3$) δ 7.30 – 7.25 (m, 2H), 7.20 – 7.15 (m, 3H), 4.10 – 3.96 (m, 2H), 2.59 (ddd, *J* = 8.8, 7.2, 2.0 Hz, 2H), 2.49 – 2.40 (m, 2H), 2.23 – 2.15 (m, 2H), 1.73 – 1.62 (m, 5H), 1.63 – 1.53 (m, 5H), 1.50 – 1.40 (m, 1H), 1.40 – 1.33 (m, 1H), 1.27 – 1.15 (m, 1H), 0.91 (d, *J* = 6.5 Hz, 3H) ppm. **^{13}C NMR** (101 MHz, $CDCl_3$) δ 160.1, 143.0, 128.5, 128.4, 125.7, 71.8, 36.9, 36.3, 36.0, 32.4, 30.1, 29.1, 27.2, 26.0, 25.9, 25.5, 19.9 ppm. **IR** (neat, cm^{-1}): 2928, 2857, 1496, 1450, 1377, 1059, 937, 840, 747, 698. **HRMS** (ESI+): m/z *calcd.* for ($C_{19}H_{30}NO$) $[M+H]^+$: 288.2322; *found* 288.2327.

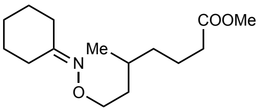

methyl 7-((cyclohexylideneamino)oxy)-5-methylheptanoate (6d). Following general procedure F, using cyclohexanone *O*-but-3-en-1-yl oxime (**1a**, 40.1 mg, 0.24 mmol) and methyl 4-iodobutanoate (**2j**, 45.6 mg, 0.20 mmol). ^{1}H NMR and GC-FID analysis of the crude mixtures showed a rr of 7.8:1 (rr refers to the ratio of the major regioisomer to all other regioisomers). Purification by flash column chromatography (*n*-hexane/EtOAc 25/1) afforded **6d** (41.6 mg, 77% yield) as colorless oil. In an independent experiment, 44.3 mg (83% yield) were obtained, giving an average yield of 80%. **^{1}H NMR** (400 MHz, $CDCl_3$) δ 4.08 – 3.96 (m, 2H), 3.66 (s, 3H),

2.48 – 2.40 (m, 2H), 2.28 (t, *J* = 7.3 Hz, 2H), 2.22 – 2.15 (m, 2H), 1.72 – 1.63 (m, 4H), 1.63 – 1.54 (m, 6H), 1.49 – 1.39 (m, 1H), 1.38 – 1.28 (m, 1H), 1.22 – 1.13 (m, 1H), 0.91 (d, *J* = 6.6 Hz, 3H) ppm. **^{13}C NMR** (101 MHz, $CDCl_3$) δ 174.4, 160.1, 71.6, 51.6, 36.7, 35.9, 34.5, 32.4, 30.0, 27.2, 26.0, 25.9, 25.4, 22.6, 19.8 ppm. **IR** (neat, cm^{-1}): 2930, 2859, 1740, 1436, 1377, 1169, 1057, 938. **HRMS** (ESI+): m/z *calcd.* for ($C_{15}H_{28}NO_3$) $[M+H]^+$: 270.2064; *found* 270.2062.

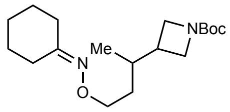

***tert*-butyl 3-(4-((cyclohexylideneamino)oxy)butan-2-yl)azetidine-1-carboxylate (6e).** Following general procedure F, using cyclohexanone *O*-but-3-en-1-yl oxime (**1a**, 40.1 mg, 0.24 mmol) and *tert*-butyl 3-iodoazetidine-1-carboxylate (**2o**, 56.6 mg, 0.30 mmol). ^{1}H NMR and GC-FID analysis of the crude mixtures showed a rr of 7:1 (rr refers to the ratio of the major regioisomer to all other regioisomers). Purification by flash column chromatography (*n*-hexane/EtOAc 10/1) afforded **6e** (53.2 mg, 82% yield) as colorless oil. In an independent experiment, 55.8 mg (86% yield) were obtained, giving an average yield of 84%. **^{1}H NMR** (400 MHz, $CDCl_3$) δ 4.08 – 3.96 (m, 2H), 3.93 (td, *J* = 8.4, 3.2 Hz, 2H), 3.63 – 3.58 (m, 2H), 2.51 – 2.37 (m, 2H), 2.34 – 2.23 (m, 1H), 2.22 – 2.11 (m, 2H), 1.81 – 1.68 (m, 1H), 1.70 – 1.63 (m, 5H), 1.62 – 1.57 (m, 2H), 1.43 (s, 9H), 1.40 – 1.30 (m, 1H), 0.88 (d, *J* = 6.6 Hz, 3H) ppm. **^{13}C NMR** (101 MHz, $CDCl_3$) δ 160.4, 156.5, 79.3, 71.0, 35.2, 34.4, 33.3, 32.4, 28.6, 27.2, 26.0, 25.9, 25.5, 16.5 ppm. **IR** (neat, cm^{-1}): 2931, 2874, 1703, 1401, 1365, 1255, 1134, 1062, 935. **HRMS** (ESI+): m/z *calcd.* for ($C_{18}H_{32}N_2NaO_3$) $[M+Na]^+$: 347.2305; *found* 347.2309.

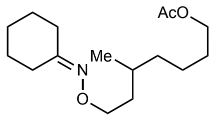

7-((cyclohexylideneamino)oxy)-5-methylheptyl acetate (6f). Following general procedure F, using cyclohexanone *O*-but-3-en-1-yl oxime (**1a**, 40.1 mg, 0.24 mmol) and 4-iodobutyl acetate (**2d**, 48.4 mg, 0.20 mmol). ^{1}H NMR and GC-FID analysis of the crude mixtures showed a rr was 9:1 (rr refers to the ratio of the major regioisomer to all other regioisomers). Purification by flash column chromatography (*n*-hexane/EtOAc 30/1) afforded **6f** (45.3 mg, 80% yield) as colorless oil. In an independent experiment, 48.5 mg (85% yield) were obtained, giving an average yield of 83%. **^{1}H NMR** (400 MHz, $CDCl_3$) δ 4.12 – 3.96 (m, 4H), 2.45 (t, *J* = 5.9 Hz, 2H), 2.24 – 2.16 (m, 2H), 2.04 (s, 3H), 1.74 – 1.62 (m, 3H), 1.64 – 1.53 (m, 7H), 1.50 – 1.37 (m, 1H), 1.39 – 1.30 (m, 3H), 1.21 – 1.13 (m, 1H), 0.90 (d, *J* = 6.6 Hz, 3H) ppm. **^{13}C NMR** (101 MHz, $CDCl_3$) δ 171.4, 160.1, 71.7, 64.8, 36.8, 35.9, 32.4, 30.1, 29.0, 27.2, 26.0, 25.9, 25.5, 23.5, 21.2, 19.8 ppm. **IR** (neat, cm^{-1}): 2929, 2859, 1739, 1449, 1365, 1236, 1039, 938, 840. **HRMS** (ESI+): m/z *calcd.* for ($C_{16}H_{30}NO_3$) $[M+H]^+$: 284.2220; *found* 284.2223.

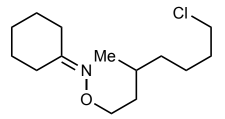

cyclohexanone *O*-(7-chloro-3-methylheptyl) oxime (6g). Following general procedure F, using cyclohexanone *O*-but-3-en-1-yl oxime (**1a**, 40.1 mg, 0.24 mmol) and 1-chloro-4-iodobutane (**2g**, 43.7 mg, 0.20 mmol). ^{1}H NMR and GC-FID analysis of the crude mixtures showed a rr of 7.7:1 (rr refers to the ratio of the major regioisomer to all other regioisomers). Purification by flash column chromatography (*n*-hexane/EtOAc 50/1) afforded **6g** (42.8 mg, 82% yield) as colorless oil. In an independent experiment, 40.0 mg (77% yield) were obtained, giving an average yield of 80%. **^{1}H NMR** (400 MHz, $CDCl_3$) δ 4.10 – 3.96 (m, 2H), 3.53 (t, *J* = 6.7 Hz, 2H), 2.55 – 2.39 (m, 2H), 2.30 – 2.12 (m, 2H), 1.80 – 1.70 (m, 2H), 1.72 – 1.61 (m, 3H), 1.65 – 1.52 (m, 5H), 1.51 – 1.29 (m, 4H), 1.25 – 1.11 (m, 1H), 0.91 (d, *J* = 6.5 Hz, 3H) ppm. **^{13}C NMR** (101 MHz, $CDCl_3$) δ 160.1, 71.7, 45.3, 36.4, 35.9, 33.0, 32.4, 30.0, 27.2, 26.0, 25.9, 25.5, 24.4, 19.8 ppm. **IR** (neat, cm^{-1}): 2930, 2860, 1448, 1378, 1058, 938. **HRMS** (ESI+): m/z *calcd.* for ($C_{14}H_{27}ClNO$) $[M+H]^+$: 260.1776; *found* 260.1781.

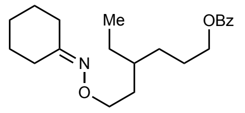

6-((cyclohexylideneamino)oxy)-4-ethylhexyl benzoate (6h). Following general procedure F, using cyclohexanone O-pent-4-en-1-yl oxime (**1m**, 43.5 mg, 0.24 mmol) and 3-iodopropyl benzoate (**2a**, 58.0 mg, 0.20 mmol). ^{1}H NMR and GC-FID analysis of the crude mixtures showed a rr of 5.8:1 (rr refers to the ratio of the major regioisomer to all other regioisomers). Purification by flash column chromatography (*n*-hexane/EtOAc 25/1) afforded **6h** (51.9 mg, 75% yield) as colorless oil. In an independent experiment, 55.3 mg (80% yield) were obtained, giving an average yield of 78%. **^{1}H NMR** (400 MHz, $CDCl_3$) δ 8.08 – 8.01 (m, 2H), 7.58 – 7.52 (m, 1H), 7.47 – 7.39 (m, 2H), 4.30 (t, *J* = 6.7 Hz, 2H), 4.05 (t, *J* = 6.8 Hz,2H), 2.44 (t, *J* = 6.0 Hz, 2H), 2.23 – 2.14 (m, 2H), 1.81 – 1.73 (m, 2H), 1.69 – 1.56 (m, 8H), 1.50 – 1.41 (m, 3H), 1.41 – 1.33 (m, 2H), 0.88 (t, *J* = 7.4 Hz, 3H) ppm. **^{13}C NMR** (101 MHz, $CDCl_3$) δ 166.8, 160.1, 132.9, 130.7, 129.7, 128.5, 71.7, 65.5, 36.1, 32.4, 32.4, 29.7, 27.2, 26.1, 26.1, 26.0, 25.9, 25.5, 10.9 ppm. **IR** (neat, cm^{-1}): 2932, 2860, 1720, 1451, 1314, 1273, 1111, 1070, 712. **HRMS** (ESI+): m/z *calcd.* for ($C_{21}H_{32}NO_3$) $[M+H]^+$: 346.2377; *found* 346.2387.

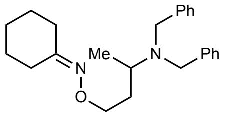

cyclohexanone *O*-(3-(dibenzylamino)butyl) oxime (7a). Following general procedure H, using cyclohexanone *O*-but-3-en-1-yl oxime (**1a**, 33.5 mg, 0.20 mmol) and *O*-benzoyl-*N*,*N*-dibenzylhydroxylamine (**4a**, 95.2 mg, 0.30 mmol). ^{1}H NMR and GC-FID analysis of the crude mixtures showed a rr of 11:1 (rr refers to the ratio of the major regioisomer to all other regioisomers). Purification by flash column chromatography (*n*-hexane/EtOAc 50/1) afforded **7a** (64.9 mg, 89% yield) as a light-yellow oil. In an independent experiment, 66.2 mg (91% yield) were obtained, giving an average yield of 90%. **^{1}H NMR** (400 MHz, $CDCl_3$) δ 7.41 – 7.35 (m, 4H), 7.32 – 7.24 (m, 4H), 7.23 – 7.16 (m, 2H), 4.18 – 4.02 (m, 2H), 3.72 (d, *J* = 13.8 Hz, 2H), 3.43 (d, *J* = 13.9

Hz, 2H), 2.99 – 2.82 (m, 1H), 2.37 – 2.10 (m, 4H), 2.06 – 1.90 (m, 1H), 1.73 – 1.45 (m, 7H), 1.05 (d, *J* = 6.6 Hz, 3H) ppm. **^{13}C NMR** (101 MHz, $CDCl_3$) δ 160.0, 140.7, 128.8, 128.2, 126.7, 71.1, 53.4, 49.8, 33.2, 32.3, 27.1, 26.0, 25.8, 25.3, 13.6 ppm. **IR** (neat, cm^{-1}): 3026, 2929, 2858, 1494, 1450, 1373, 1152, 1103, 1028, 992, 936, 872, 839, 743, 696. **HRMS** (ESI+): m/z *calcd.* for ($C_{24}H_{33}N_2O$) $[M+H]^+$: 365.2587; *found* 365.2590.

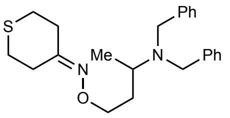

tetrahydro-4*H*-thiopyran-4-one *O*-(3-(dibenzylamino)butyl) oxime (7b). Following general procedure H, using tetrahydro-4*H*-thiopyran-4-one *O*-but-3-en-1-yl oxime (**1g**, 37.1 mg, 0.20 mmol) and *O*-benzoyl-*N*,*N*-dibenzylhydroxylamine (**4a**, 95.2 mg, 0.30 mmol). ^{1}H NMR and GC-FID analysis of the crude mixtures showed a rr of 13:1 (rr refers to the ratio of the major regioisomer to all other regioisomers). Purification by flash column chromatography (*n*-hexane/EtOAc 50/1) afforded **7b** (69.7 mg, 91% yield) as a light-brown oil. In an independent experiment, 70.9 mg (93% yield) were obtained, giving an average yield of 92%. **^{1}H NMR** (400 MHz, $CDCl_3$) δ 7.44 – 7.37 (m, 4H), 7.34 – 7.27 (m, 4H), 7.26 – 7.19 (m, 2H), 4.19 – 4.09 (m, 2H), 3.75 (d, *J* = 13.8 Hz, 2H), 3.44 (d, *J* = 13.8 Hz, 2H), 2.97 – 2.86 (m, 1H), 2.81 – 2.72 (m, 2H), 2.68 – 2.49 (m, 6H), 2.04 – 1.91 (m, 1H), 1.69 – 1.58 (m, 1H), 1.08 (d, *J* = 6.6 Hz, 3H) ppm. **^{13}C NMR** (101 MHz, $CDCl_3$) δ 156.7, 140.6, 128.7, 128.2, 126.7, 71.2, 53.3, 49.5, 34.0, 33.2, 29.9, 28.4, 27.4, 13.4 ppm. **IR** (neat, cm^{-1}): 3026, 2958, 2929, 2873, 1493, 1453, 1425, 1373, 1268, 1152, 1073, 1039, 978, 940, 912, 881, 743, 696. **HRMS** (ESI+): m/z *calcd.* for ($C_{23}H_{31}N_2OS$) $[M+H]^+$: 383.2152; *found* 383.2166.

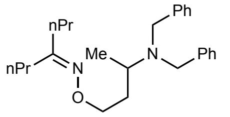

heptan-4-one *O*-(3-(dibenzylamino)butyl) oxime (7c). Following general procedure G, using heptan-4-one *O*-but-3-en-1-yl oxime (**1j**, 36.7 mg, 0.20 mmol) and *O*-benzoyl-*N*,*N*-dibenzylhydroxylamine (**4a**, 95.2 mg, 0.30 mmol). ^{1}H NMR and GC-FID analysis of the crude mixtures showed a rr of 9:1 (rr refers to the ratio of the major regioisomer to all other regioisomers). Purification by flash column chromatography (*n*-hexane/EtOAc 100/1) afforded **7c** (68.8 mg, 90% yield) as a light-yellow oil. In an independent experiment, 66.4 mg (87% yield) were obtained, giving an average yield of 89%. **^{1}H NMR** (400 MHz, $CDCl_3$) δ 7.42 – 7.39 (m, 4H), 7.32 – 7.26 (m, 4H), 7.23 – 7.17 (m, 2H), 4.16 – 4.02 (m, 2H), 3.73 (d, *J* = 13.8 Hz, 2H), 3.45 (d, *J* = 13.8 Hz, 2H), 2.91 (q, *J* = 6.8 Hz, 1H), 2.14 – 1.93 (m, 5H), 1.67 – 1.57 (m, 1H), 1.56 – 1.45 (m, 2H), 1.45 – 1.34 (m, 2H), 1.06 (d, *J* = 6.6 Hz, 3H), 0.92 (t, *J* = 7.4 Hz, 3H), 0.85 (t, *J* = 7.4 Hz, 3H) ppm. **^{13}C NMR** (101 MHz, $CDCl_3$) δ 161.0, 140.7, 128.8, 128.3, 126.8, 71.2, 53.4, 50.0, 36.3, 33.2, 30.0, 20.2, 19.3, 14.4, 14.0, 13.7 ppm. **IR** (neat, cm^{-1}): 3027, 2960, 2931, 2871, 1494, 1454, 1374, 1152, 1073, 1055, 1027, 942, 905, 743, 727, 697. **HRMS** (ESI+): m/z *calcd.* for ($C_{25}H_{37}N_2O$) $[M+H]^+$: 381.2900; *found* 381.2907.

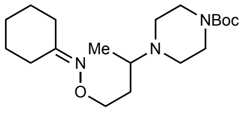

***tert*-butyl 4-(4-((cyclohexylideneamino)oxy)butan-2-yl)piperazine-1-carboxylate (7d).** Following general procedure G, using cyclohexanone *O*-but-3-en-1-yl oxime (**1a**, 33.5 mg, 0.20 mmol) and tert-butyl 4-(benzoyloxy)piperazine-1-carboxylate (**4g**, 91.6 mg, 0.30 mmol). ^{1}H NMR and GC-FID analysis of the crude mixtures showed a rr of 5:1 (rr refers to the ratio of the major regioisomer to all other regioisomers). Purification by flash column chromatography (*n*-hexane/EtOAc 4/1) afforded **7d** (46.7 mg, 66% yield) as a light-yellow oil. In an independent experiment, 50.8 mg (72% yield) were obtained, giving an average yield of 69%. **^{1}H NMR** (400 MHz, $CDCl_3$) δ 4.13 – 3.98 (m, 2H), 3.46 – 3.33 (m, 4H), 2.80 – 2.67 (m, 1H), 2.54 – 2.33 (m, 6H), 2.23 – 2.14 (m, 2H), 1.93 – 1.82 (m, 1H), 1.76 – 1.63 (m, 3H), 1.63 – 1.53 (m, 4H), 1.45 (s, 9H), 0.99 (d, *J* = 6.6 Hz, 3H) ppm. **^{13}C NMR** (101 MHz, $CDCl_3$) δ 160.2, 154.9, 79.6, 71.2, 56.7, 48.2, 32.7, 32.4, 28.6, 27.2, 26.0, 25.9, 25.4, 14.6 ppm. **IR** (neat, cm^{-1}): 2929, 2858, 2810, 1694, 1451, 1420, 1365, 1245, 1167, 1128, 1045, 1003, 935, 868. **HRMS** (ESI+): m/z *calcd.* for ($C_{19}H_{36}N_3O_3$) $[M+H]^+$: 354.2751; *found* 354.2747.

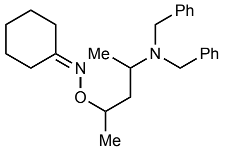

cyclohexanone *O*-(4-(dibenzylamino)pentan-2-yl) oxime (7e). Following general procedure H, using cyclohexanone *O*-pent-4-en-2-yl oxime (**1x**, 39.1 mg, 0.20 mmol) and *O*-benzoyl-*N*,*N*-dibenzylhydroxylamine (**4a**, 95.2 mg, 0.30 mmol). ^{1}H NMR and GC-FID analysis of the crude mixtures showed a rr > 20:1 (rr refers to the ratio of the major regioisomer to all other regioisomers). Purification by flash column chromatography (*n*-hexane/EtOAc 80/1) afforded **7e** (69.6 mg, 92% yield, dr 1.4:1) as a light-yellow oil. In an independent experiment, 66.4 mg (88% yield) were obtained, giving an average yield of 90%. **^{1}H NMR** (400 MHz, $CDCl_3$) δ 7.43 – 7.37 (m, 4H), 7.32 – 7.25 (m, 4H), 7.24 – 7.16 (m, 2H), 4.42 – 4.23 (m, 1H) 3.70 (d, *J* = 13.8 Hz, 2H), 3.47 and 3.44 (d, *J* = 13.9 Hz and *J* = 13.5 Hz, 2H), 3.00 – 2.86 (m, 1H), 2.40 – 2.20 (m, 2H), 2.20 – 2.02 (m, 2H), 1.76 – 1.43 (m, 7H), 1.39 – 1.30 (m, 1H), 1.17 and 1.08 (d, *J* = 6.2 Hz and *J* = 6.5 Hz, 3H), 1.06 and 1.04 (d, *J* = 4.0 Hz and *J* = 3.6 Hz, 3H) ppm. **^{13}C NMR** (101 MHz, $CDCl_3$) δ 159.4 (159.1), 140.9 (140.8), 129.0 (128.8), 128.2 (128.2), 126.7 (126.7), 75.8 (75.6), 53.6 (53.4), 49.9 (49.3), 40.1 (40.0), 32.4 (32.4), 27.1 (27.1), 26.0, 25.8 (25.7), 25.3 (25.3), 20.7 (20.2), 14.2 (14.0). ppm. **IR** (neat, cm^{-1}): 3026, 2926, 2798, 1729, 1494, 1451, 1372, 1245, 1154, 1028, 990, 950, 8842, 745, 697. **HRMS** (ESI+): m/z *calcd.* for ($C_{25}H_{35}N_2O$) $[M+H]^+$: 379.2744; *found* 379.2755.

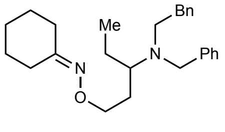

cyclohexanone *O*-(3-(benzyl(phenethyl)amino)pentyl) oxime (7f). Following general procedure H, using cyclohexanone *O*-pent-4-en-1-yl oxime (**1m**, 39.1 mg, 0.20 mmol) and *O*-benzoyl-*N*-benzyl-*N*-

phenethylhydroxylamine (**4b**, 99.4 mg, 0.30 mmol). ^{1}H NMR and GC-FID analysis of the crude mixtures showed a rr of 6.7:1 (rr refers to the ratio of the major regioisomer to all other regioisomers). Purification by flash column chromatography (*n*-hexane/EtOAc 50/1) afforded **7f** (51.3 mg, 65% yield) as a light-yellow oil. In an independent experiment, 47.4 mg (60% yield) were obtained, giving an average yield of 63%. **^{1}H NMR** (400 MHz, $CDCl_3$) δ 7.35 – 7.26 (m, 4H), 7.26 – 7.13 (m, 4H), 7.12 – 7.06 (m, 2H), 4.12 – 4.01 (m, 2H), 3.73 – 3.61 (m, 2H), 2.75 – 2.58 (m, 5H), 2.48 – 2.31 (m, 2H), 2.23 – 2.15 (m, 2H), 1.86 – 1.75 (m, 1H), 1.70 – 1.62 (m, 2H), 1.62 – 1.52 (m, 4H), 1.39 – 1.25 (m, 3H), 0.88 (t, J = 7.4 Hz, 3H) ppm. **^{13}C NMR** (101 MHz, $CDCl_3$) δ 160.0, 141.2, 141.0, 128.9, 128.8, 128.3, 128.2, 126.6, 125.9, 71.4, 58.4, 54.5, 52.0, 36.1, 32.4, 29.7, 27.2, 26.0, 25.9, 25.4, 23.2, 12.1 ppm. **IR** (neat, cm^{-1}): 3026, 2927, 2857, 1733, 1603, 1494, 1451, 1373, 1249, 1141, 1105, 1048, 1028, 917, 839, 730, 696. **HRMS** (ESI+): m/z *calcd.* for ($C_{26}H_{37}N_2O$) [M+H]$^+$: 393.2900; *found* 393.2892.

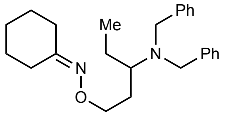

cyclohexanone *O*-(3-(dibenzylamino)pentyl) oxime (7g). Following general procedure H, using cyclohexanone *O*-pent-4-en-1-yl oxime (**1m**, 39.1 mg, 0.20 mmol) and *O*-benzoyl-*N*,*N*-dibenzylhydroxylamine (**4a**, 95.2 mg, 0.30 mmol). ^{1}H NMR and GC-FID analysis of the crude mixtures showed a rr of 7.4:1. Purification by flash column chromatography (*n*-hexane/EtOAc 60/1) afforded **7g** (55.3 mg, 73% yield) as a light-yellow oil. In an independent experiment, 52.1 mg (69% yield) were obtained, giving an average yield of 71%. **^{1}H NMR** (400 MHz, $CDCl_3$) δ 7.40 – 7.34 (m, 4H), 7.31 – 7.26 (m, 4H), 7.23 – 7.17 (m, 2H), δ 4.16 – 4.01 (m, 2H), 3.66 – 3.52 (m, 4H), 2.58 – 2.51 (m, 1H), 2.40 – 2.22 (m, 2H), 2.22 – 2.14 (m, 2H), 1.99 – 1.88 (m, 1H), 1.75 – 1.47 (m, 8H), 1.38 – 1.27 (m, 1H), 0.90 (t, J = 7.4 Hz, 3H) ppm. **^{13}C NMR** (101 MHz, $CDCl_3$) δ 160.0, 140.7, 129.0, 128.2, 126.7, 71.4, 56.4, 53.5, 32.4, 29.1, 27.2, 26.0, 25.8, 25.3, 22.4, 12.1 ppm. **IR** (neat, cm^{-1}): 3027, 2928, 2858, 1494, 1451, 1373, 1144, 1047, 1027, 931, 916, 886, 838, 743, 696. **HRMS** (ESI+): m/z *calcd.* for ($C_{25}H_{35}N_2O$) [M+H]$^+$: 379.2744; *found* 379.2754.

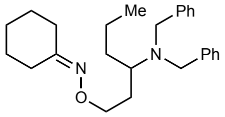

cyclohexanone *O*-(3-(dibenzylamino)hexyl) oxime (7h). Following general procedure H, using cyclohexanone *O*-hex-5-en-1-yl oxime (**1n**, 41.9 mg, 0.20 mmol) and *O*-benzoyl-*N*,*N*-dibenzylhydroxylamine (**4a**, 95.2 mg, 0.30 mmol). ^{1}H NMR and GC-FID analysis of the crude mixtures showed a rr of 4.4:1. Purification by flash column chromatography (*n*-hexane/EtOAc 60/1) afforded **7h** (53.2 mg, 68% yield) as a colorless oil. In an independent experiment, 57.0 mg (73% yield) were obtained, giving an average yield of 70%. ^{1}H NMR and GC-FID analysis of the reaction (*E*)-cyclohexanone *O*-hex-4-en-1-yl oxime (**1t**, 41.9 mg, 0.20 mmol) and *O*-benzoyl-*N*,*N*-dibenzylhydroxylamine (**4a**, 95.2 mg, 0.30 mmol) resulted in a rr of 5.7:1. Purification by flash column chromatography (*n*-hexane/EtOAc 60/1) afforded **7h** (60.5 mg, 77% yield) as a colorless oil. In an independent experiment, 57.4 mg (73% yield) were obtained, giving an average yield of 75%. **^{1}H NMR** (400

MHz, $CDCl_3$) δ 7.42 – 7.35 (m, 4H), 7.34 – 7.27 (m, 4H), 7.25 – 7.18 (m, 2H), 4.19 – 4.04 (m, 2H), 3.60 (q, *J* = 13.7 Hz, 4H), 2.74 – 2.63 (m, 1H), 2.42 – 2.14 (m, 4H), 2.01 – 1.91 (m, 1H), 1.75 – 1.48 (m, 7H), 1.46 – 1.21 (m, 4H), 0.84 (td, *J* = 7.2, 1.0 Hz, 3H) ppm. **^{13}C NMR** (101 MHz, $CDCl_3$) δ 159.9, 140.7, 129.0, 128.2, 126.7, 71.3, 54.1, 53.5, 32.3, 31.9, 29.4, 27.1, 26.0, 25.8, 25.3, 20.3, 14.3 ppm. **IR** (neat, cm^{-1}): 3027, 2928, 2859, 1494, 1451, 1364, 1249, 1144, 1103, 1044, 1028, 943, 916, 887, 838, 744, 697. **HRMS** (ESI+): m/z *calcd.* for ($C_{26}H_{37}N_2O$) $[M+H]^+$: 393.2900; *found* 393.2915.

5. Unsuccessful or low yielding substrates

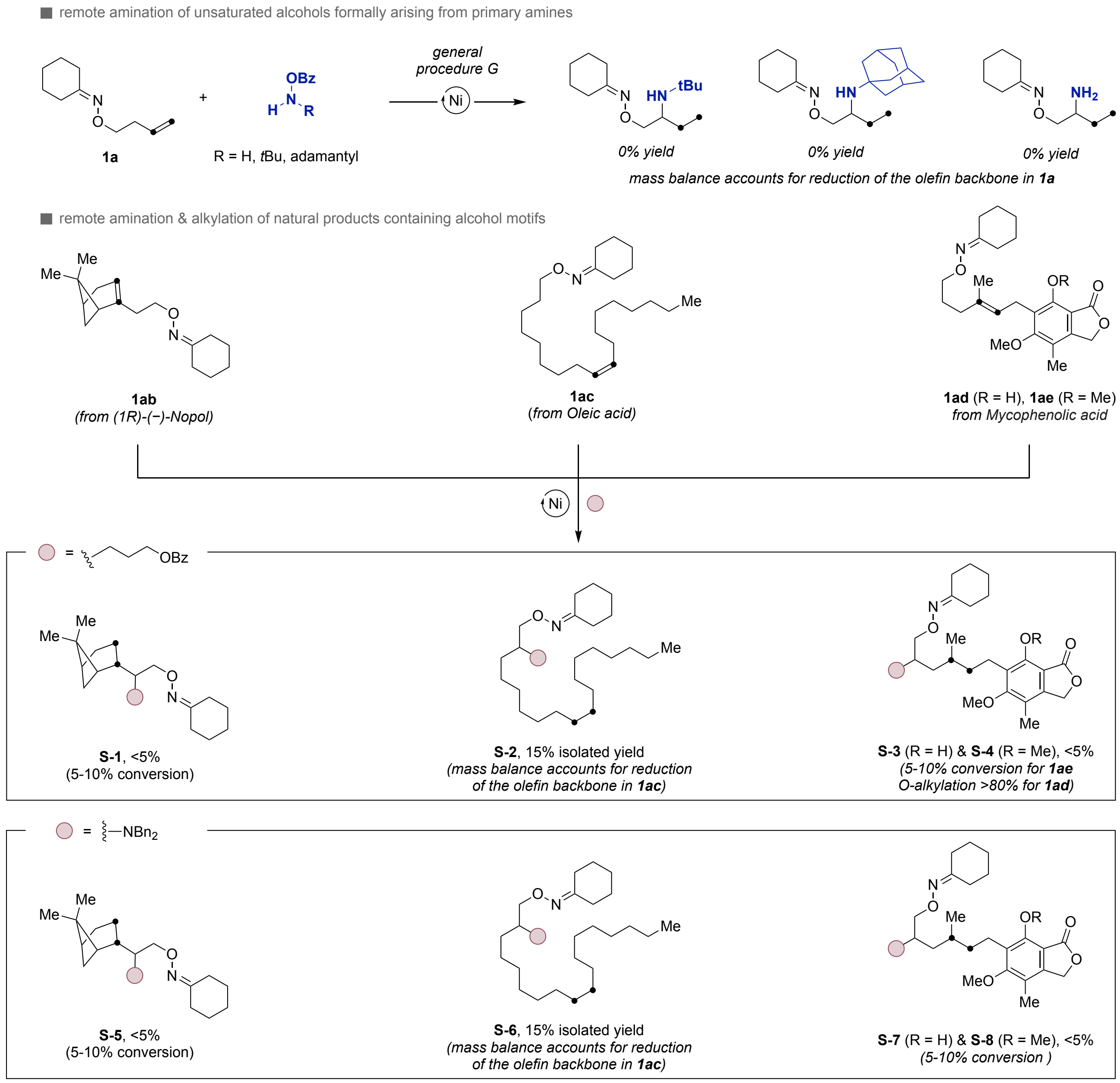

6. Deprotection of the traceless directing group

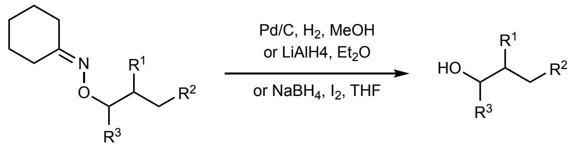

General procedure I:[9] To a glass screw 12 mL tube was added the alkylation or amination products (1.0 equiv., 0.20 mmol) and brought inside a nitrogen-filled glovebox. Pd/C (palladium, 5% on activated carbon, 80 mg) was added and the reaction tube was sealed with a Teflon-lined screw cap. Subsequently, the flash was taken outside of the glovebox, back-filled with H_2 balloon 3 times. After adding 2.0 mL of anhydrous MeOH, the reaction mixture was left stirred at room temperature for 24 h. Then, the H_2 balloon was removed, and the resulting layer was filtered through celite and finally evaporated in vacuo. The residue was purified by silica gel column chromatography (*n*-hexane/ethyl acetate) to obtain the targeted aliphatic alcohol.

General procedure J:[10] In a glass screw 12 mL tube, the protected aliphatic alcohol (1.0 equiv., 0.20 mmol) was dissolved in dry diethyl ether. Then, $LiAlH_4$ (10.0 equiv.) was added and the reaction mixture was stirred for 48 h at 40 °C. The reaction was then quenched with water, extracted with diethyl ether, dried over Na_2SO_4, and purified by column chromatography (*n*-hexane/ethyl acetate) to yield the corresponding aliphatic alcohol.

General procedure K:[11] To a stirred solution of the protected aliphatic alcohol (1.0 equiv., 0.20 mmol) in THF (1 mL) was added $NaBH_4$ (5.0 equiv., 1.0 mmol) at room temperature. Then, a solution of I_2 (2.0 equiv., 0.40 mmol) in THF (1 mL) was added dropwise and slowly at room temperature. Subsequently, the mixture was heated at reflux and stirred overnight. The reaction mixture was quenched by dropwise addition of MeOH (10 mL) at 0 °C, diluted with water (20 mL) and brine (20 mL), and extracted with $CHCl_3$ (4 x 30 mL). The combined organic layers were then dried over Na_2SO_4, filtered, and the product was purified by column chromatography (n-hexane/ethyl acetate) to yield the corresponding aliphatic alcohol.

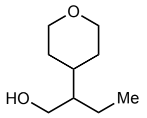

2-(tetrahydro-2*H*-pyran-4-yl)butan-1-ol (8a). Following general procedure I, using cyclohexanone *O*-(2-(tetrahydro-2*H*-pyran-4-yl)butyl) oxime (**3ah**, 50.7 mg, 0.20 mmol) and Pd/C (80 mg, palladium, 5% on activated carbon, dry), purification by column chromatography (n-hexane/EtOAc 7/1) afforded **8a** (29.0 mg, 92% yield) as colorless oil. **^{1}H NMR** (400 MHz, $CDCl_3$) δ 4.02 – 3.92 (m, 2H), 3.69 – 3.61 (m, 2H), 3.37 (td, *J* = 11.76, 2.23 Hz, 2H), 1.76 – 1.62 (m, 1H), 1.62 – 1.50 (m, 2H), 1.49 – 1.27 (m, 4H), 1.24 – 1.16 (m, 2H), 0.93 (t, *J* = 7.33 Hz, 3H) ppm. **^{13}C NMR** (101 MHz, $CDCl_3$) δ 68.6, 68.6, 62.4, 47.2, 35.7, 30.6, 30.3, 20.4, 12.1 ppm. **IR** (neat, cm^{-1}): 3418, 2955, 2930, 2874, 2847, 1465, 1097, 1040, 983. **HRMS** (ESI+): m/z *calcd.* for ($C_9H_{19}O_2$) $[M+H]^+$: 159.1380; *found* 159.1377.

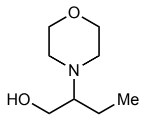

2-morpholinobutan-1-ol (8b). Following general procedure I, using cyclohexanone *O*-(2-morpholinobutyl) oxime (**5r**, 50.8 mg, 0.20 mmol) and Pd/C (80 mg, palladium, 5% on activated carbon, dry), purification by column chromatography (n-hexane/EtOAc 2/1) afforded **8b** (24 mg, 75% yield) as a light-yellow solid. **^{1}H NMR** (400 MHz, $CDCl_3$) δ 3.76 – 3.62 (m, 4H), 3.56 (dd, *J* = 10.9, 5.1 Hz, 1H), 3.26 (t, *J* = 10.4 Hz, 1H), 3.19 (br, 1H), 2.76 – 2.66 (m, 2H), 2.54 – 2.41 (m, 3H), 1.71 – 1.59 (m, 1H), 1.22 – 1.09 (m, 1H), 0.90 (t, *J* = 7.5 Hz, 3H) ppm. **^{13}C NMR** (101 MHz, $CDCl_3$) δ 67.6, 67.2, 59.9, 48.6, 18.3, 11.7 ppm. **IR** (neat, cm^{-1}): 3339, 2928, 2854, 1650, 1462, 1386, 1313, 1260, 1115, 1054, 993, 919, 850. **HRMS** (ESI+): m/z *calcd.* for ($C_8H_{18}NO_2$) $[M+H]^+$: 160.1332; *found* 160.1337.

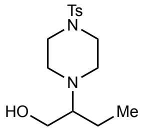

2-(4-tosylpiperazin-1-yl)butan-1-ol (8c). Following general procedure I, using cyclohexanone *O*-(2-(4-tosylpiperazin-1-yl)butyl) oxime (**5u**, 81.5 mg, 0.20 mmol) and Pd/C (80 mg, palladium, 5% on activated carbon, dry), purification by column chromatography (DCM/EtOAc 1/2) afforded **8c** (60 mg, 96% yield) as a light-yellow solid. **^{1}H NMR** (400 MHz, $CDCl_3$) δ 7.66 – 7.60 (d, *J* = 8.3 Hz, 2H), 7.36 – 7.31 (m, 2H), 3.51 (dd, *J* = 10.7, 4.8 Hz, 1H), 3.20 (t, *J* = 10.5 Hz, 1H), 3.10 – 2.87 (m, 4H), 2.84 – 2.74 (m, 2H), 2.58 – 2.48 (m, 3H), 2.44 (s, 3H), 1.65 – 1.52 (m, 1H), 1.20 – 1.06 (m, 1H), 0.88 (t, *J* = 7.5 Hz, 3H) ppm. **^{13}C NMR** (101 MHz, $CDCl_3$) δ 143.9, 132.5, 129.9, 127.9, 67.0, 60.0, 47.5, 46.8, 21.7, 18.6, 11.7 ppm. **IR** (neat, cm^{-1}): 3389, 2960, 2851, 1598, 1452, 1404, 1344, 1328, 1306, 1260, 1163, 1092, 946, 808, 731, 652. **HRMS** (ESI+): m/z *calcd.* for ($C_{15}H_{25}N_2O_3S$) $[M+H]^+$: 313.1580; *found* 313.1576. **MP**: 110 – 112 ºC.

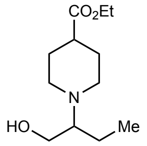

ethyl 1-(1-hydroxybutan-2-yl)piperidine-4-carboxylate (8d). Following general procedure I, using ethyl 1-(1-((cyclohexylideneamino)oxy)butan-2-yl)piperidine-4-carboxylate (**5w**, 64.9 mg, 0.20 mmol) and Pd/C (80 mg, palladium, 5% on activated carbon, dry), purification by column chromatography (n-hexane/EtOAc 2/1) afforded **8d** (41.2 mg, 90% yield) as a brown oil. **^{1}H NMR** (400 MHz, $CDCl_3$) δ 4.13 (q, *J* = 7.1 Hz, 2H), 3.54 (dd, *J* = 10.5, 5.0 Hz, 1H), 3.22 (t, *J* = 10.5 Hz, 1H), 2.86 – 2.76 (m, 1H), 2.76 – 2.49 (m, 4H), 2.34 – 2.23 (m, 1H), 2.17 (td, *J* = 11.3, 2.6 Hz, 1H), 1.96 – 1.87 (m, 2H), 1.84 – 1.53 (m, 3H), 1.25 (t, *J* = 7.1 Hz, 3H), 1.19 – 1.04 (m, 1H), 0.88 (t, *J* = 7.5 Hz, 3H).ppm. **^{13}C NMR** (101 MHz, $CDCl_3$) δ 175.1, 67.2, 60.5, 60.1, 51.5, 44.5, 41.6, 29.2, 28.9, 18.4, 14.4, 11.8 ppm. **IR** (neat, cm^{-1}): 3392, 2926, 2856, 2810, 1729, 1448, 1376, 1285, 1261, 1174, 1096, 1045. **HRMS** (ESI+): m/z *calcd.* for ($C_{12}H_{24}NO_3$) $[M+H]^+$: 230.1751; *found* 230.1755.

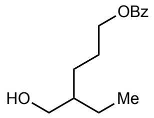

4-(hydroxymethyl)hexyl benzoate (8e). Following general procedure I, using 4-(((cyclohexylideneamino)oxy)methyl)hexyl benzoate (**3a**, 66.3 mg, 0.20 mmol) and Pd/C (80 mg, palladium, 5% on activated carbon, dry), purification by column chromatography (n-hexane/EtOAc 10/1) afforded **8e** (44.5 mg, 95% yield) as colorless oil. **^{1}H NMR** (400 MHz, $CDCl_3$) δ 8.07 – 8.00 (m, 2H), 7.58 – 7.51 (m, 1H), 7.48 – 7.39 (m, 2H), 4.32 (t, *J* = 6.68 Hz, 2H), 3.62 – 3.55 (m, 2H), 1.85 – 1.74 (m, 2H), 1.56 – 1.32 (m, 6H), 0.91 (t, *J* = 7.39 Hz, 3H) ppm. **^{13}C NMR** (101 MHz, $CDCl_3$) δ 166.8, 133.0, 130.6, 129.6, 128.5, 65.4, 65.1, 41.8, 26.9, 26.3, 23.4, 11.2 ppm. **IR** (neat, cm^{-1}): 2930, 2859, 1739, 1436, 1198, 1168, 1045, 936. **HRMS** (ESI+): m/z *calcd.* for ($C_{14}H_{20}NaO_3$) $[M+Na]^+$: 259.1305; *found* 259.1298.

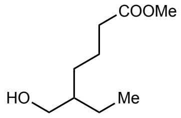

methyl 5-(hydroxymethyl)heptanoate (8f). Following general procedure I, using methyl 5-(((cyclohexylideneamino)oxy)me-thyl)heptanoate (**3y**, 53.9 mg, 0.20 mmol) and Pd/C (80 mg, palladium, 5% on activated carbon, dry), purification by column chromatography (n-hexane/EtOAc 10/1) afforded **8f** (30.3 mg, 87% yield) as colorless oil. **^{1}H NMR** (400 MHz, $CDCl_3$) δ 3.67 (s, 3H), 3.60 – 3.52 (m, 2H), 2.32 (t, *J* = 7.37 Hz, 2H), 1.70 – 1.58 (m, 2H), 1.48 – 1.27 (m, 6H), 0.89 (t, *J* = 7.32 Hz, 3H) ppm. **^{13}C NMR** (101 MHz, $CDCl_3$) δ 174.4, 65.1, 51.7, 41.9, 34.4, 30.0, 23.4, 22.2, 11.3 ppm. **IR** (neat, cm^{-1}): 3407, 2955, 2931, 2874, 1737, 1460, 1437, 1363, 1200, 1166, 1038. **HRMS** (ESI+): m/z *calcd.* for ($C_9H_{18}NaO_3$) $[M+Na]^+$: 197.1148; *found* 197.1144.

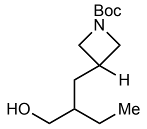

***tert*-butyl 3-(2-(hydroxymethyl)butyl)azetidine-1-carboxylate (8g).** Following general procedure I, using *tert*-butyl 3-(2-(((cyclohexylideneamino)oxy)methyl)butyl) azetidine-1-carboxylate (**3af**, 67.7 mg, 0.20 mmol) and Pd/C (80 mg, palladium, 5% on activated carbon, dry), purification by column chromatography (n-hexane/EtOAc 10/1) afforded **8g** (36.5 mg, 75% yield) as colorless oil. **^{1}H NMR** (400 MHz, $CDCl_3$) δ 3.97 (td, *J* = 8.30, 1.80 Hz, 2H), 3.54 – 3.45 (m, 4H), 2.65 – 2.51 (m, 1H), 2.02 (s, 1H), 1.67 – 1.51 (m, 2H), 1.40 (s, 9H), 1.36 – 1.24 (m, 3H), 0.87 (t, *J* = 7.17 Hz, 3H) ppm. **^{13}C NMR** (101 MHz, $CDCl_3$) δ 156.5, 79.2, 64.6, 40.4, 35.8, 28.4, 27.0, 23.5, 11.2 ppm. **IR** (neat, cm^{-1}): 3440, 2962, 2930, 2876, 1701, 1677, 1404, 1365, 1138, 1046, 771. **HRMS** (ESI+): m/z *calcd.* for ($C_{13}H_{25}NNaO_3$) $[M+Na]^+$: 266.1727; *found* 266.1732.

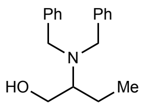

2-(dibenzylamino)butan-1-ol (8h). Following general procedure J, using cyclohexanone *O*-(2-(dibenzylamino)butyl) oxime (**5a**, 72.9 mg, 0.20 mmol) and $LiAlH_4$ (76 mg, 2.0 mmol), purification by column chromatography (n-hexane/EtOAc 5/1) afforded **8h** (48.6 mg, 90% yield) as colorless oil. **^{1}H NMR** (400 MHz,

$CDCl_3$) δ 7.36 – 7.20 (m, 10 H), 3.83 (d, *J* = 13.3 Hz, 2H), 3.53 (dd, *J* = 10.6, 4.9 Hz, 1H), 3.48 – 3.37 (m, 3H), 2.77 – 2.68 (m, 1H), 1.87 – 1.74 (m, 1H), 1.28 – 1.18 (m, 1H), 0.92 (t, *J* = 7.5 Hz, 3H) ppm. **^{13}C NMR** (101 MHz, $CDCl_3$) δ 139.5, 129.2, 128.6, 127.3, 60.9, 60.6, 53.4, 18.0, 11.9 ppm. **IR** (neat, cm^{-1}): 3434, 3027, 2930, 2873, 1602, 1494, 1453, 1362, 1250, 1134, 1051, 1028, 967, 908, 745, 696. **HRMS** (ESI+): m/z *calcd.* for ($C_{18}H_{24}NO$) $[M+Na]^+$: 270.1852; *found* 270.1854.

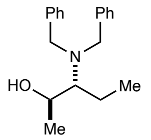

3-(dibenzylamino)pentan-2-ol (8i). Following general procedure K, using cyclohexanone *O*-(3-(dibenzylamino)pentan-2-yl) oxime (**5p**, 75.7 mg, 0.20 mmol), $NaBH_4$ (38 mg, 1.0 mmol) and I_2 (101.5 mg, 0.40 mmol), purification by column chromatography (n-hexane/EtOAc 5/1) afforded **8i** (35.6 mg, 63% yield) as colorless oil. **^{1}H NMR** (400 MHz, $CDCl_3$) δ 7.36 – 7.29 (m, 8H), 7.26 – 7.21 (m, 2H), 3.95 – 3.87 (m, 1H), 3.74 (d, *J* = 13.7 Hz, 2H), 3.64 (d, *J* = 13.7 Hz, 2H), 2.61 – 2.55 (m, 1H), 2.33 (br, 1H), 1.82 – 1.72 (m, 1H), 1.55 – 1.45 (m, 1H), 1.18 (d, *J* = 6.6 Hz, 3H), 1.00 (t, *J* = 7.4 Hz, 3H) ppm. **^{13}C NMR** (101 MHz, $CDCl_3$) δ 140.1, 129.1, 128.5, 127.2, 66.9, 63.7, 55.6, 20.7, 18.5, 12.6 ppm. **IR** (neat, cm^{-1}): 3424, 3027, 2962, 2926, 1723, 1601, 1494, 1453, 1360, 1176, 1096, 1010, 917, 815, 745, 698. **HRMS** (ESI+): m/z *calcd.* for ($C_{19}H_{26}NO$) $[M+H]^+$: 284.2009; *found* 284.2013.

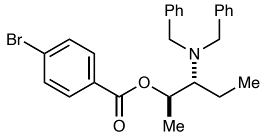

3-(dibenzylamino)pentan-2-yl 4-bromobenzoate (8i'). 4-bromobenzoyl chloride (24.2 mg, 0.11 mmol) was added to a stirred solution of 3-(dibenzylamino)pentan-2-ol (**8i**, 28.3 mg, 0.10 mmol), DMAP (1.2 mg, 0.10 mmol) and DCC (24.8 mg, 0.12 mmol) in CH_2Cl_2 (2 ml) in one portion under argon at 0 °C. The resulting solution was stirred at room temperature for 72 hours. After that, the solid was removed by filtration. Then, wash the solids with CH_2Cl_2 (2 x 5 ml) and the combined filtrate was evaporated. Purification by column chromatography (n-hexane/EtOAc 10/1) afforded **8i'** (38.2 mg, 80 % yield) as white solid. **^{1}H NMR** (400 MHz, $CDCl_3$) δ 7.91 – 7.85 (m, 2H), 7.61 – 7.54 (m, 2H), 7.38 – 7.26 (m, 8H), 7.25 – 7.18 (m, 2H), 5.60 – 5.51 (m, 1H), 3.81 (d, *J* = 13.7 Hz, 2H), 3.52 (d, *J* = 13.7 Hz, 2H), 2.65 – 2.58 (m, 1H), 1.94 – 1.80 (m, 1H), 1.71 – 1.59 (m, 1H), 1.28 (d, *J* = 6.5 Hz, 2H), 1.04 (t, *J* = 7.4 Hz, 1H) ppm. **^{13}C NMR** (101 MHz, $CDCl_3$) δ 165.4, 140.1, 131.9, 131.2, 129.8, 129.0, 128.4, 128.2, 127.0, 70.6, 62.7, 54.1, 19.4, 19.1, 12.8 ppm. **IR** (neat, cm^{-1}): 3025, 2965, 2927, 2805, 1703, 1588, 1494, 1453, 1376, 1271, 1121, 1047, 1010, 966, 856, 741, 696. **HRMS** (ESI+): m/z *calcd.* for ($C_{26}H_{29}BrNO_2$) $[M+H]^+$: 466.1376; *found* 466.1371. **MP**: 97 – 99 ºC.

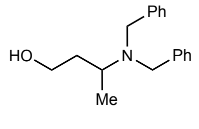

3-(dibenzylamino)butan-1-ol (8j). Following general procedure J, using cyclohexanone *O*-(3-(dibenzylamino)butyl) oxime (**7a**, 72.9 mg, 0.20 mmol) and $LiAlH_4$ (76 mg, 2.0 mmol), purification by column

chromatography (n-hexane/EtOAc 5/1) afforded **8j** (50.2 mg, 93% yield) as a light-yellow oil. **^{1}H NMR** (400 MHz, $CDCl_3$) δ 7.40 – 7.29 (m, 8H), 7.29 – 7.22 (m, 2H), 4.67 (br, 1H), 3.91 (d, *J* = 13.2 Hz, 2H), 3.74 (dt, *J* = 10.7, 4.4 Hz, 1H), 3.59 – 3.50 (m, 1H), 3.29 (d, *J* = 13.2 Hz, 2H), 3.11 – 2.99 (m, 1H), 2.06 – 1.93 (m, 1H), 1.39 – 1.30 (m, 1H), 1.07 (d, *J* = 6.6 Hz, 3H) ppm. **^{13}C NMR** (101 MHz, $CDCl_3$) δ 139.1, 129.3, 128.6, 127.3, 63.2, 53.6, 53.4, 35.0, 12.4 ppm. **IR** (neat, cm^{-1}): 3047, 2930, 2838, 1602, 1494, 1452, 1364, 1243, 1134, 1073, 1028, 967, 744, 725, 696. **HRMS** (ESI+): m/z *calcd.* for ($C_{18}H_{24}NO$) $[M+H]^+$: 270.1852; *found* 270.1856.

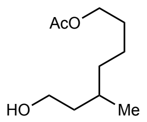

7-hydroxy-5-methylheptyl acetate (8k). Following general procedure I, using 7-((cyclohexylideneamino)oxy)-5-methylheptyl acetate (**6f**, 56.6mg, 0.20 mmol) and Pd/C (80 mg, palladium, 5% on activated carbon, dry), purification by column chromatography (n-hexane/EtOAc 10/1) afforded **8k** (33.1 mg, 89% yield) as colorless oil. **^{1}H NMR** (400 MHz, $CDCl_3$) δ 4.06 (t, *J* = 6.7 Hz, 2H), 3.74 – 3.62 (m, 2H), 2.05 (s, 3H), 1.66 – 1.53 (m, 4H), 1.43 – 1.29 (m, 5H), 1.22 – 1.13 (m, 1H), 0.90 (d, J = 6.5 Hz, 3H) ppm. **^{13}C NMR** (101 MHz, $CDCl_3$) δ 171.4, 64.7, 61.3, 40.0, 36.8, 29.5, 29.0, 23.4, 21.2, 19.7 ppm. **IR** (neat, cm^{-1}): 3440, 2962, 2930, 2876, 1701, 1677, 1404, 1365, 1138, 1046, 771. **HRMS** (ESI+): m/z *calcd.* for ($C_{10}H_{20}NaO_3$) $[M+Na]^+$: 211.1305; *found* 211.1299.

References:

1. Fang, D.; Zhang, Y.; Chen, Y. Radical C(*sp*^{3})–H Heck-Type Reaction of N -Alkoxybenzimidoyl Chlorides with Styrenes to Construct Alkenols. *Org. Lett.* **2022**, *24*, 2050–2054.
2. Wu, Z.; Fatuzzo, N.; Dong, G. Distal Alkenyl C–H Functionalization via the Palladium/Norbornene Cooperative Catalysis. *J. Am. Chem. Soc.* **2020**, *142*, 2715–2720.
3. Talavera, L.; Freund, R. R. A.; Zhang, H.; Wakeling, M.; Jensen, M.; Martin, R. Nickel-Catalyzed 1,1-Aminoborylation of Unactivated Terminal Alkenes. *ACS Catal.* **2023**, *13*, 5538–5543.
4. Watkins, W. J.; Chen, J. M.; Cho, A.; Chong, L.; Collins, N.; Fardis, M.; Huang, W.; Hung, M.; Kirschberg, T.; Lee, W. A.; Liu, X.; Thomas, W.; Xu, J.; Zeynalzadegan, A.; Zhang, J. Phosphonic Acid-Containing Analogues of Mycophenolic Acid as Inhibitors of IMPDH. *Bioorganic & Medicinal Chemistry Letters* **2006**, *16*, 3479–3483.
5. Li, Y.; Yang, Y.; Xin, J.; Tang, P. Nucleophilic Trifluoromethoxylation of Alkyl Halides without Silver. Nat Commun 2020, 11 (1), 755.
6. Svejstrup, T. D.; Ruffoni, A.; Juliá, F.; Aubert, V. M.; Leonori, D. Synthesis of Arylamines via Aminium Radicals. *Angew. Chem. Int. Ed.* **2017**, *56*, 14948–14952.
7. Bera, S.; Mao, R.; Hu, X. Enantioselective C(*sp*^{3})–C(*sp*^{3}) Cross-Coupling of Non-Activated Alkyl Electrophiles via Nickel Hydride Catalysis. *Nat. Chem.* **2021**, *13*, 270–277.
8. Chianelli, D.; Rucker, P. V.; Roland, J.; Tully, D. C.; Nelson, J.; Liu, X.; Bursulaya, B.; Hernandez, E. D.; Wu, J.; Prashad, M.; Schlama, T.; Liu, Y.; Chu, A.; Schmeits, J.; Huang, D. J.; Hill, R.; Bao, D.; Zoll, J.; Kim, Y.; Groessl, T.; McNamara, P.; Liu, B.; Richmond, W.; Sancho-Martinez, I.; Phimister, A.; Seidel, H. M.; Badman, M. K.; Joseph, S. B.; Laffitte, B.; Molteni, V. Nidufexor (LMB763), a Novel FXR Modulator for the Treatment of Nonalcoholic Steatohepatitis. *J. Med. Chem.* **2020**, *63*, 3868–3880.
9. Xue, Y.; Zhou, R.-B.; Luo, J.; Hu, B.-C.; Liu, Z.-Q.; Jiang, C. Palladium-Catalyzed C(Sp3)–H Nitrooxylation of Masked Alcohols. *Org. Biomol. Chem.* **2023**, *21*, 75–79.
10. Tan, E.; Zanini, M.; Echavarren, A. M. Iridium-Catalyzed β-Alkynylation of Aliphatic Oximes as Masked Carbonyl Compounds and Alcohols. *Angew. Chem. Int. Ed.* **2020**, *59*, 10470–10473.
11. Antien, K.; Geraci, A.; Parmentier, M.; Baudoin, O. A New Dioxazolone for the Synthesis of 1,2-Aminoalcohols via Iridium(III)-Catalyzed C(*sp*^{3})−H Amidation. *Angew. Chem. Int. Ed.* **2021**, *60*, 22948–22955.

7. Crystallographic Data

Data collection: The measured crystals were prepared under inert conditions immersed in perfluoropolyether as protecting oil for manipulation.

Crystal structure determination for compound **8i'** was carried out using a Rigaku diffractometer equipped with a Pilatus 200K area detector, a Rigaku MicroMax-007HF microfocus rotating anode with MoK_{α} radiation, Confocal Max Flux optics and an Oxford Cryosystems low temperature device Cryostream 700 plus (T = -173 °C). Full-sphere data collection was used with ω and φ scans. *Programs used:* Data collection data reduction with CrysAlisPro[1] and absorption correction with Scale3 Abspack scaling algorithm[2].

Crystal structure determination for **5a, 5c** and **8j** was carried out using an Apex DUO Kappa 4-axis goniometer equipped with an APPEX 2 4K CCD area detector, a Microfocus Source E025 IuS using MoK_{α} radiation, Quazar MX multilayer Optics as monochromator and an Oxford Cryosystems low temperature device Cryostream 700 plus (T = -173 °C). Full-sphere data collection was used with ω and φ scans. *Programs used:* Data collection APEX-2[3], data reduction Bruker Saint[4] V/.60A and absorption correction SADABS[5].

Structure Solution and Refinement: Crystal structure solution was achieved using the computer program SHELXT[6]. Visualization and processing was performed with the program OLEX2[7]. Missing atoms were subsequently located from difference Fourier synthesis and added to the atom list. Least-squares refinement on F^2 using all measured intensities was carried out using the program SHELXL 2015[8]. All non-hydrogen atoms were refined including anisotropic displacement parameters.

Comments to the structures: Compound 8i': The compound crystallizes in the centrosymmetric space group $P2_1/c$ where one molecule of the desired compound is present in the asymmetric unit. The

[1] Data reduction with CrysAlisPro 1.171.44.110 (Rigaku OD, 2018).
[2] Empirical absorption correction using spherical harmonics implemented in Scale3 Abspack scaling algorithm, CrysAlisPro 1.171.44.110 (Rigaku OD, 2018).
[3] Data collection with APEX II version v2013.4-1. Bruker (2007). Bruker AXS Inc., Madison, Wisconsin, USA.
[4] Data reduction with Bruker SAINT version V8.30c. Bruker (2007). Bruker AXS Inc., Madison, Wisconsin, USA.
[5] SADABS: V2012/1 Bruker (2001). Bruker AXS Inc., Madison, Wisconsin, USA. Blessing, Acta Cryst. **1995**, A51, 33-38.
[6] SHELXT; V2018/2. Sheldrick, G.M. *Acta Cryst.* **2015** A71, 3-8.
[7] OLEX2 Version 1.5-ac7-014. O. V. Dolomanov, L. J. Bourhis, R. J. Gildea, J. A. K. Howard, H. Puschmann, OLEX2: A complete structure solution, refinement and analysis program. *J. Appl. Cryst.* **2009**, *42*, 339–341.
[8] SHELXL; SHELXL-2018/3. Sheldrick, G.M. *Acta Cryst.* **2015** C71, 3-8.

structure presents a disorder in which part of the molecule shows two inverted orientations that include the chiral atoms C8 and C10 with a ratio of 89:11. This disorder corresponds to two orientations of the same diastereomer *RS* and *SR*. In the structure the diastereomer *SS* or *RR* is not present. One of the tolyl groups is disordered over two positions (60:40). In addition to that, more than one crystal has been measured, and they lead to the same results. **Compound 5a**: The asymmetric unit contains one molecule of the organic compound. One of the phenyl rings in the molecule is disordered in two orientations with a ratio of 53:47.

Figure S1. ORTEP drawing (50 %) showing the structure of compound **5a**.

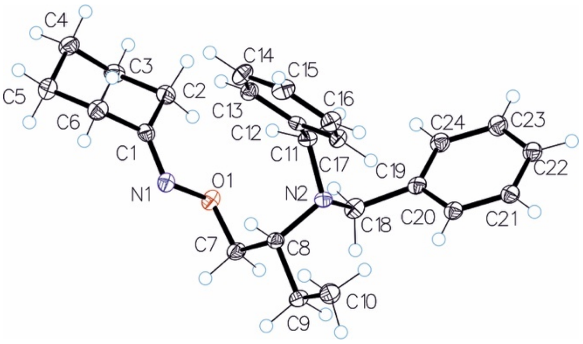

Crystal data and structure refinement for **5a** (CCDC-2449321)

Empirical formula	$C_{24}H_{32}N_2O$
Formula weight	364.51
Temperature/K	99.75
Crystal system	triclinic
Space group	P-1
a/Å	9.2106(11)
b/Å	10.2052(14)
c/Å	12.1418(16)
α/°	83.845(4)
β/°	70.901(4)
γ/°	74.706(3)
Volume/Å^3	1040.0(2)
Z	2
ρ_{calc}g/cm^3	1.164
μ/mm^{-1}	0.071
F(000)	396.0
Crystal size/mm^3	0.2 × 0.2 × 0.1
Radiation	MoKα (λ = 0.71073)
2Θ range for data collection/°	3.55 to 54.43
Index ranges	$-11 \le h \le 11$, $-12 \le k \le 13$, $-12 \le l \le 15$

Reflections collected	10159
Independent reflections	4555 [R_{int} = 0.0577, R_{sigma} = 0.0642]
Data/restraints/parameters	4555/144/300
Goodness-of-fit on F^2	1.075
Final R indexes [I>=2σ (I)]	R_1 = 0.0594, wR_2 = 0.1586
Final R indexes [all data]	R_1 = 0.0640, wR_2 = 0.1643
Largest diff. peak/hole / e $Å^{-3}$	0.35/-0.40

Figure S2. ORTEP drawing (50 %) showing the structure of compound **5c.**

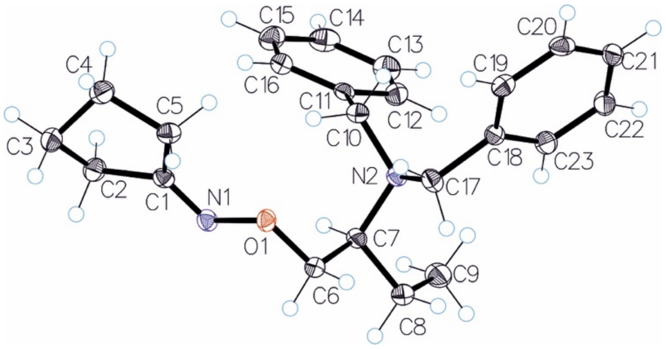

Crystal data and structure refinement for **5c** (CCDC-2449322)

Empirical formula	$C_{23}H_{30}N_2O$
Formula weight	350.49
Temperature/K	100.05
Crystal system	monoclinic
Space group	$P2_1/c$
a/Å	12.538(4)
b/Å	14.794(5)
c/Å	11.817(4)
α/°	90
β/°	115.521(10)
γ/°	90
Volume/Å^3	1978.0(12)
Z	4
ϱ_{calc}g/cm^3	1.177
μ/mm^{-1}	0.072
F(000)	760.0
Crystal size/mm^3	0.2 × 0.2 × 0.05
Radiation	MoKα (λ = 0.71073)
2Θ range for data collection/°	3.6 to 59.328
Index ranges	$-17 \le h \le 15$, $-17 \le k \le 20$, $-13 \le l \le 15$
Reflections collected	20090
Independent reflections	5364 [R_{int} = 0.0564, R_{sigma} = 0.0618]
Data/restraints/parameters	5364/0/236
Goodness-of-fit on F^2	1.025
Final R indexes [I>=2σ (I)]	R_1 = 0.0581, wR_2 = 0.1259
Final R indexes [all data]	R_1 = 0.0953, wR_2 = 0.1410
Largest diff. peak/hole / e Å^{-3}	0.29/-0.26

Figure S3. ORTEP drawing (50 %) showing the structure of compound **8i'**.

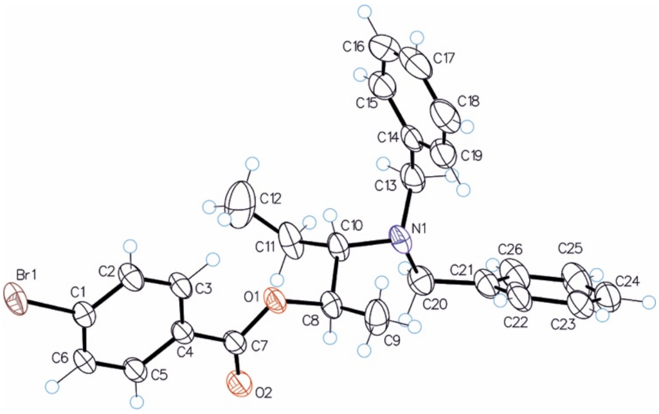

Crystal data and structure refinement for **8i'** (CCDC-2451736)

Empirical formula	$C_{26}H_{28}BrNO_2$
Formula weight	466.40
Temperature/K	100(2)
Crystal system	monoclinic
Space group	$P2_1/c$
a/Å	9.4863(4)
b/Å	10.0258(3)
c/Å	24.4849(11)
α/°	90
β/°	95.341(4)
γ/°	90
Volume/Å^3	2318.59(16)
Z	4
ϱ_{calc}g/cm^3	1.336
μ/mm^{-1}	1.794
F(000)	968.0
Crystal size/mm^3	0.2 × 0.200 × 0.1
Radiation	Mo Kα (λ = 0.71073)
2Θ range for data collection/°	4.312 to 55.754
Index ranges	-12 ≤ h ≤ 12, -13 ≤ k ≤ 12, -32 ≤ l ≤ 30
Reflections collected	16225
Independent reflections	5529 [R_{int} = 0.0303, R_{sigma} = 0.0425]
Data/restraints/parameters	5529/588/437
Goodness-of-fit on F^2	1.032
Final R indexes [I>=2σ (I)]	R_1 = 0.0394, wR_2 = 0.0854
Final R indexes [all data]	R_1 = 0.0632, wR_2 = 0.0931
Largest diff. peak/hole / e Å^{-3}	0.51/-0.43

Figure S4. ORTEP drawing (50 %) showing the structure of compound **8j**.

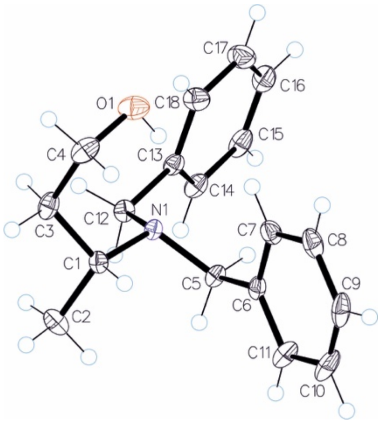

Crystal data and structure refinement for **8j** (CCDC-2449323)

Empirical formula	$C_{18}H_{23}NO$
Formula weight	269.37
Temperature/K	99.89
Crystal system	triclinic
Space group	P-1
a/Å	9.0868(6)
b/Å	9.4056(6)
c/Å	9.4194(6)
α/°	109.709(2)
β/°	94.069(2)
γ/°	92.511(2)
Volume/Å^3	754.00(8)
Z	2
ρ_{calc}g/cm^3	1.186
μ/mm^{-1}	0.073
F(000)	292.0
Crystal size/mm^3	0.2 × 0.1 × 0.1
Radiation	MoKα (λ = 0.71073)
2Θ range for data collection/°	4.506 to 63.192
Index ranges	$-13 \le h \le 13$, $-11 \le k \le 13$, $-13 \le l \le 13$
Reflections collected	12578
Independent reflections	4991 [R_{int} = 0.0398, R_{sigma} = 0.0356]
Data/restraints/parameters	4991/0/184
Goodness-of-fit on F^2	1.065
Final R indexes [I>=2σ (I)]	R_1 = 0.0504, wR_2 = 0.1382
Final R indexes [all data]	R_1 = 0.0567, wR_2 = 0.1436
Largest diff. peak/hole / e Å^{-3}	0.51/-0.23

8. NMR Spectra

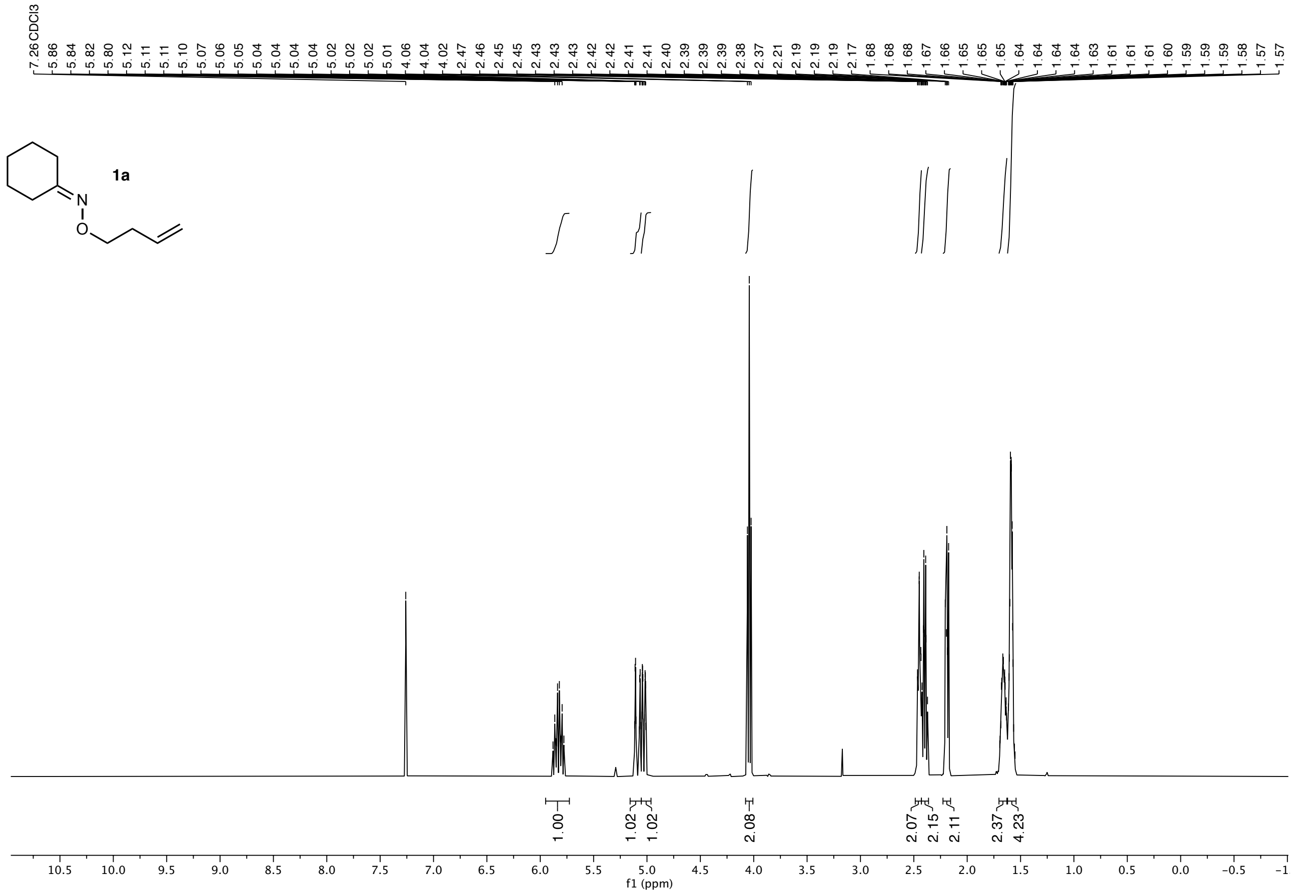

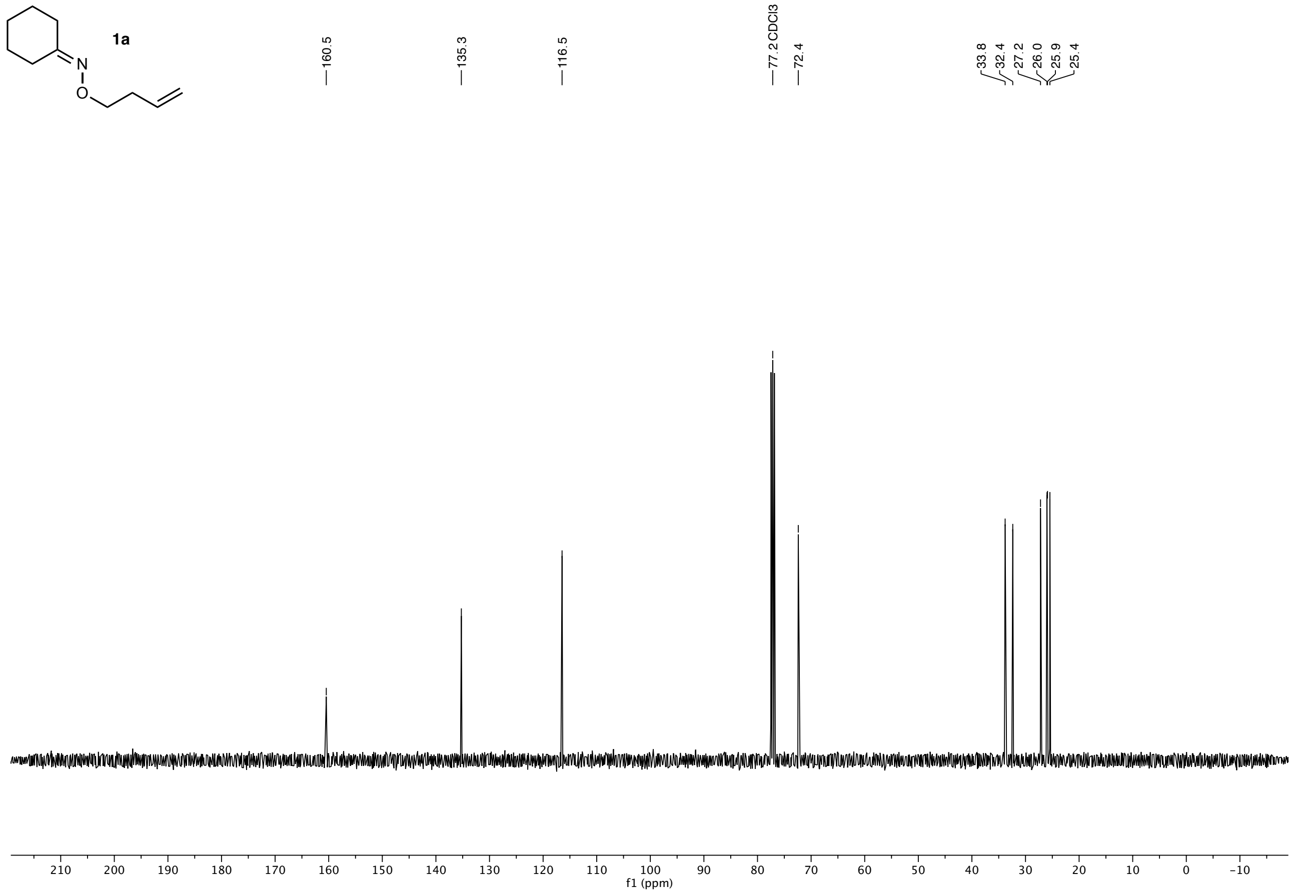

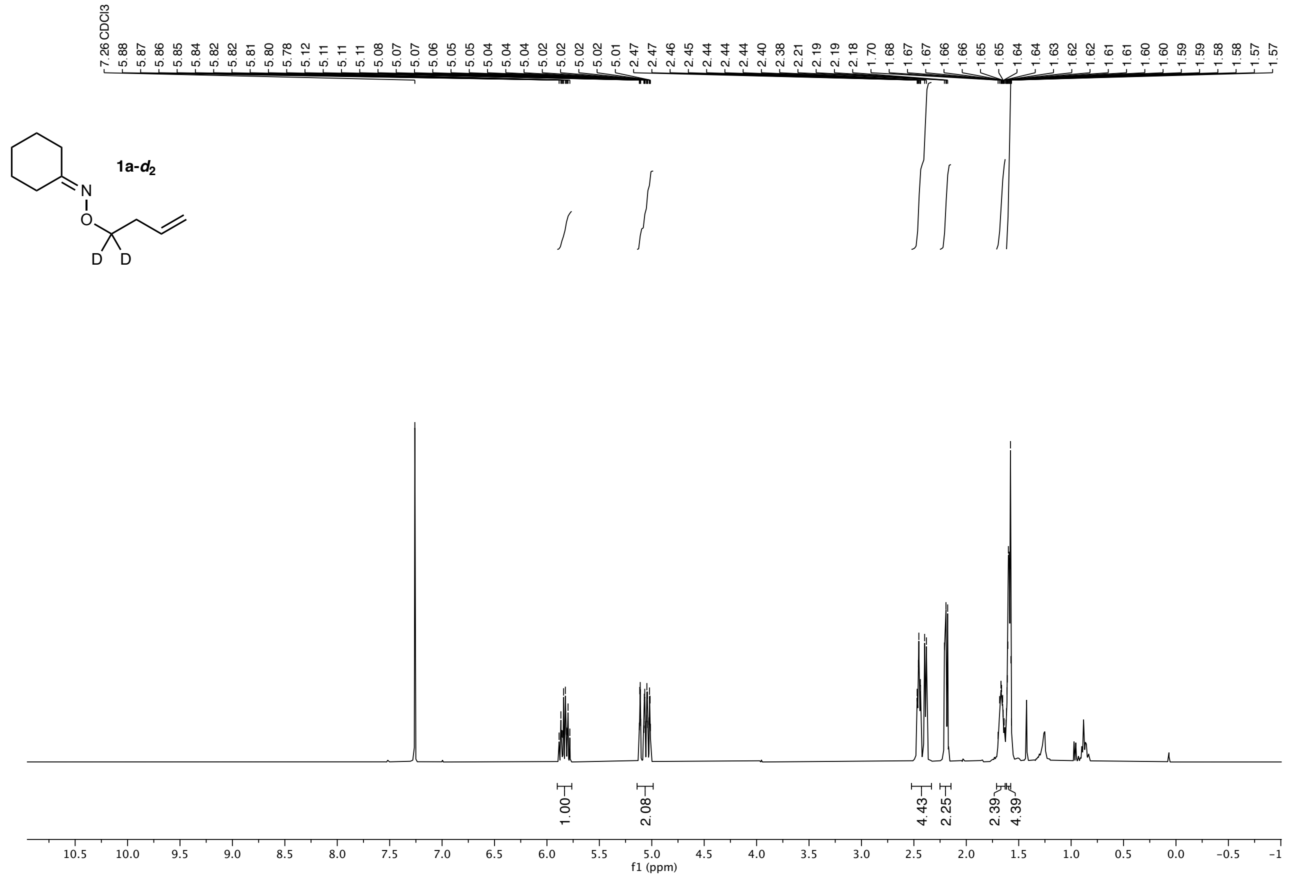
1a-d_2
N
O
D D
7.26 CDCl3
f1 (ppm)

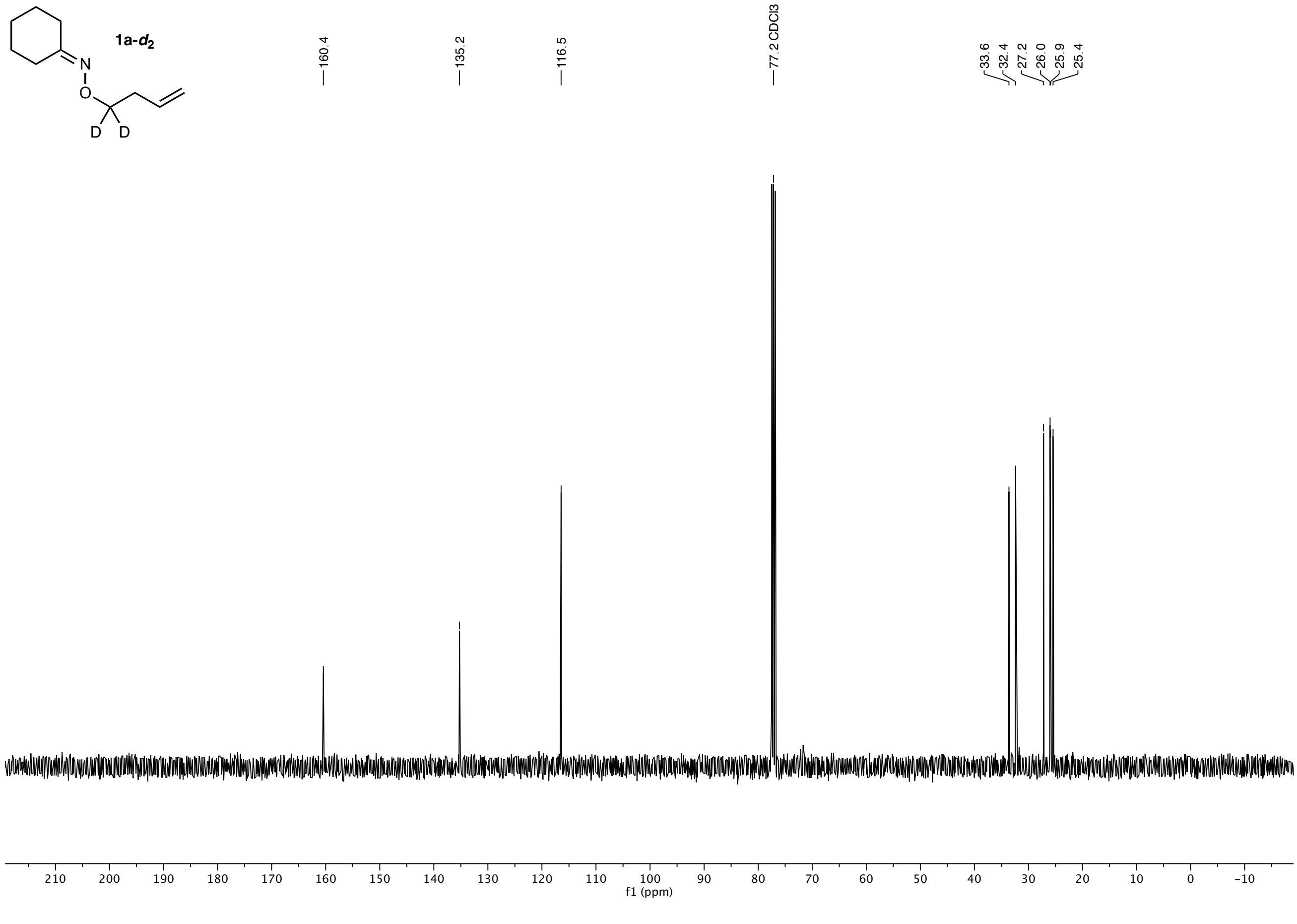
1a-d_2
N
O
D D
160.4
135.2
116.5
77.2 CDCl3
33.6
32.4
27.2
26.0
25.9
25.4
f1 (ppm)

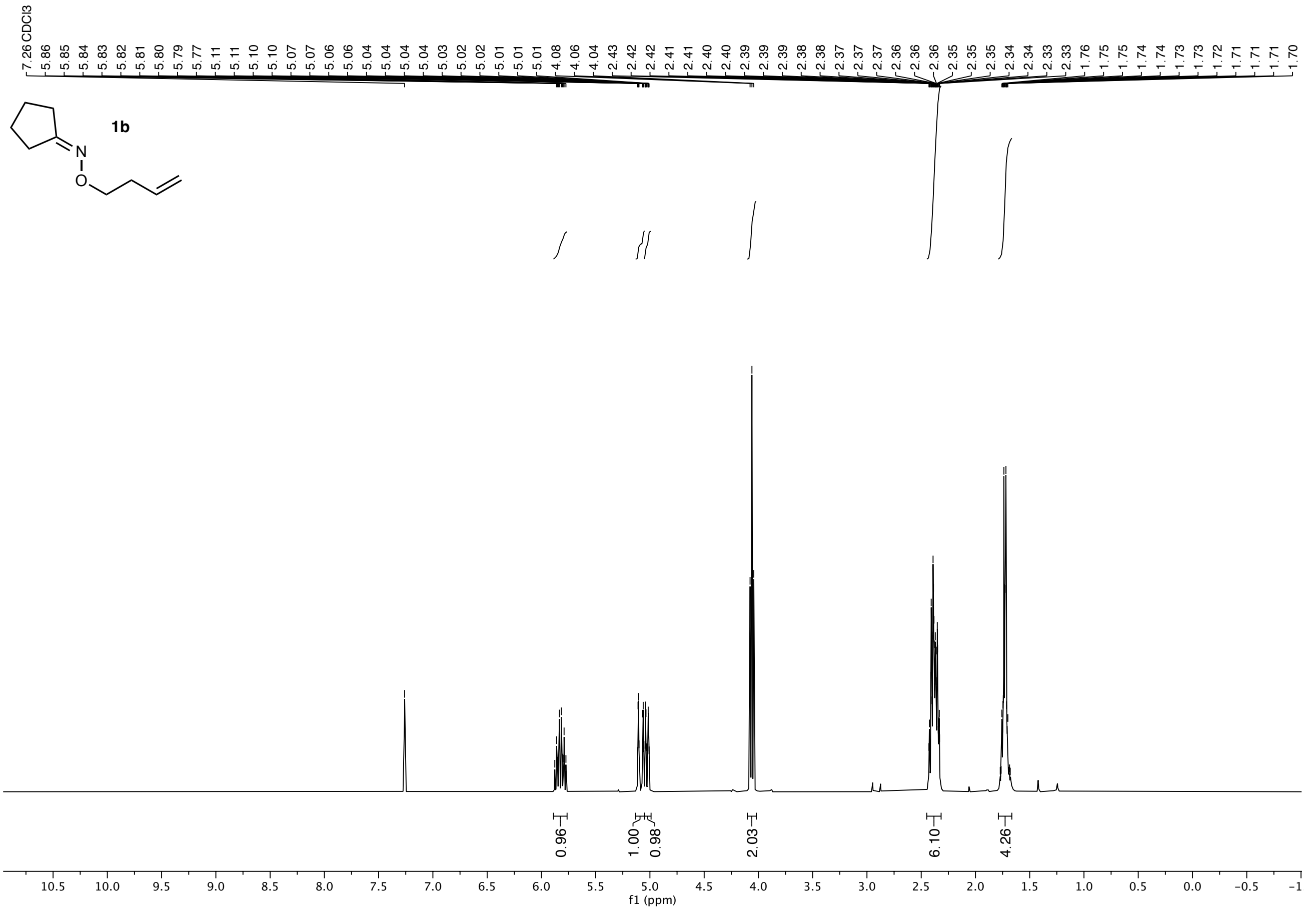
1b
7.26 CDCl3
5.86
5.85
5.84
5.83
5.82
5.81
5.80
5.79
5.77
5.11
5.11
5.10
5.10
5.07
5.07
5.06
5.06
5.04
5.04
5.04
5.04
5.03
5.02
5.02
5.01
5.01
5.01
4.08
4.06
4.04
2.43
2.42
2.42
2.41
2.41
2.40
2.40
2.39
2.39
2.39
2.38
2.38
2.37
2.37
2.36
2.36
2.36
2.35
2.35
2.35
2.34
2.34
2.33
2.33
1.76
1.75
1.75
1.74
1.74
1.73
1.73
1.72
1.71
1.71
1.71
1.70
0.96
1.00
0.98
2.03
6.10
4.26
f1 (ppm)

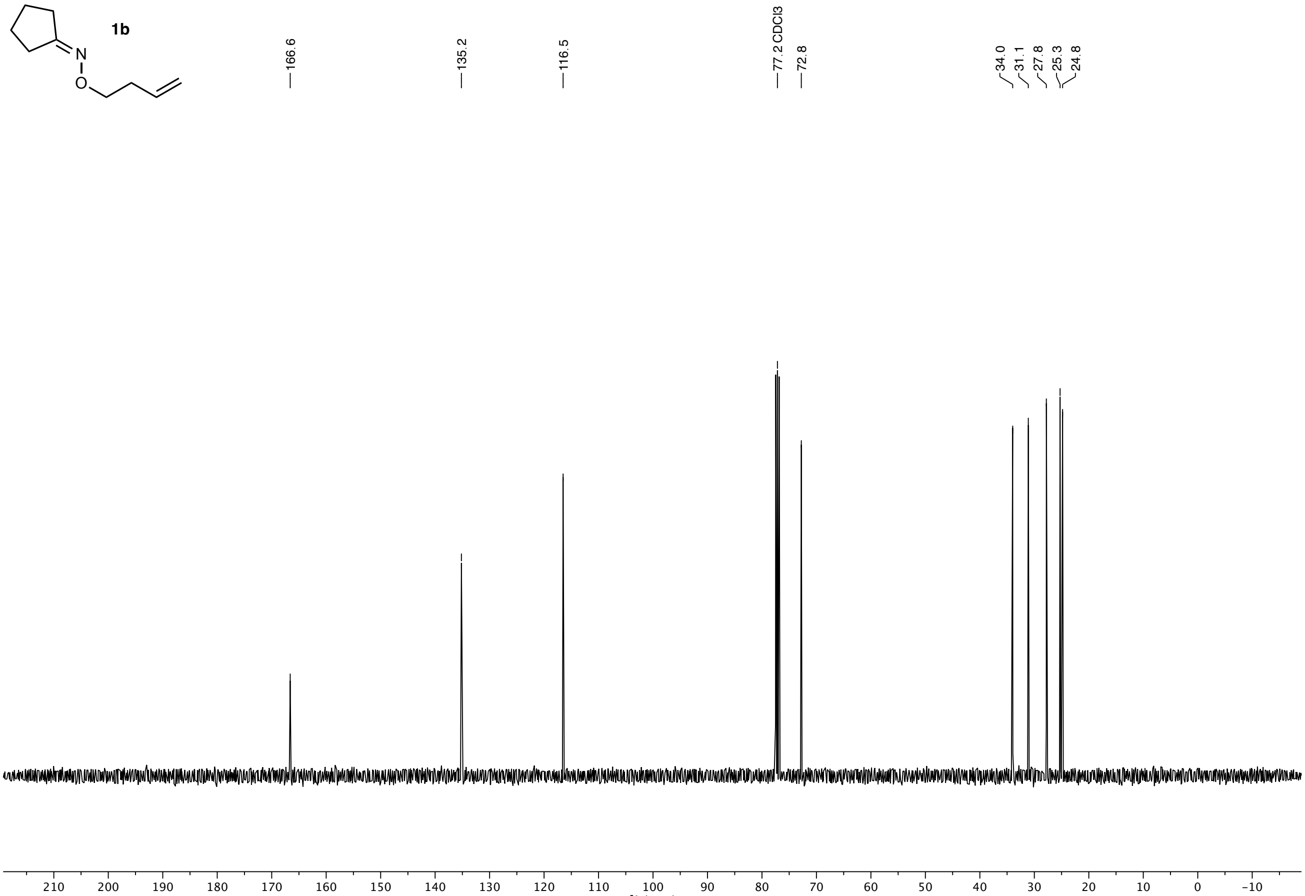
1b
166.6
135.2
116.5
77.2 CDCl3
72.8
34.0
31.1
27.8
25.3
24.8
f1 (ppm)

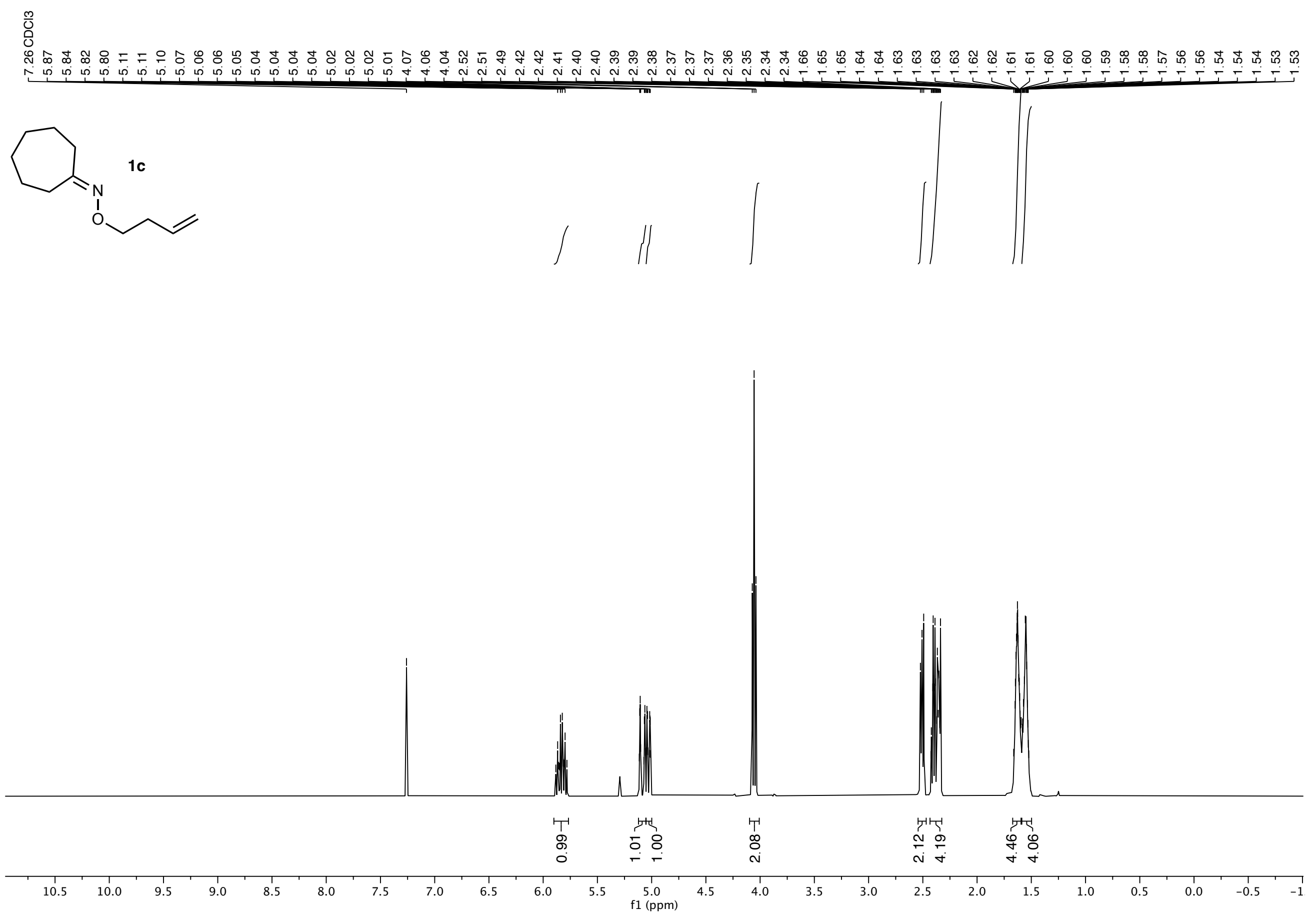
1c
N
O
7.26 CDCl3
5.87 5.84 5.82 5.80 5.11 5.11 5.10 5.07 5.06 5.06 5.05 5.04 5.04 5.04 5.04 5.02 5.02 5.02 5.01 4.07 4.06 4.04 2.52 2.51 2.49 2.42 2.42 2.41 2.40 2.40 2.39 2.39 2.38 2.37 2.37 2.37 2.36 2.35 2.34 2.34 1.66 1.65 1.65 1.64 1.64 1.63 1.63 1.63 1.63 1.62 1.62 1.61 1.61 1.61 1.60 1.60 1.60 1.59 1.58 1.58 1.57 1.56 1.56 1.54 1.54 1.54 1.53 1.53
0.99
1.01
1.00
2.08
2.12
4.19
4.46
4.06
10.5 10.0 9.5 9.0 8.5 8.0 7.5 7.0 6.5 6.0 5.5 5.0 4.5 4.0 3.5 3.0 2.5 2.0 1.5 1.0 0.5 0.0 −0.5 −1
f1 (ppm)

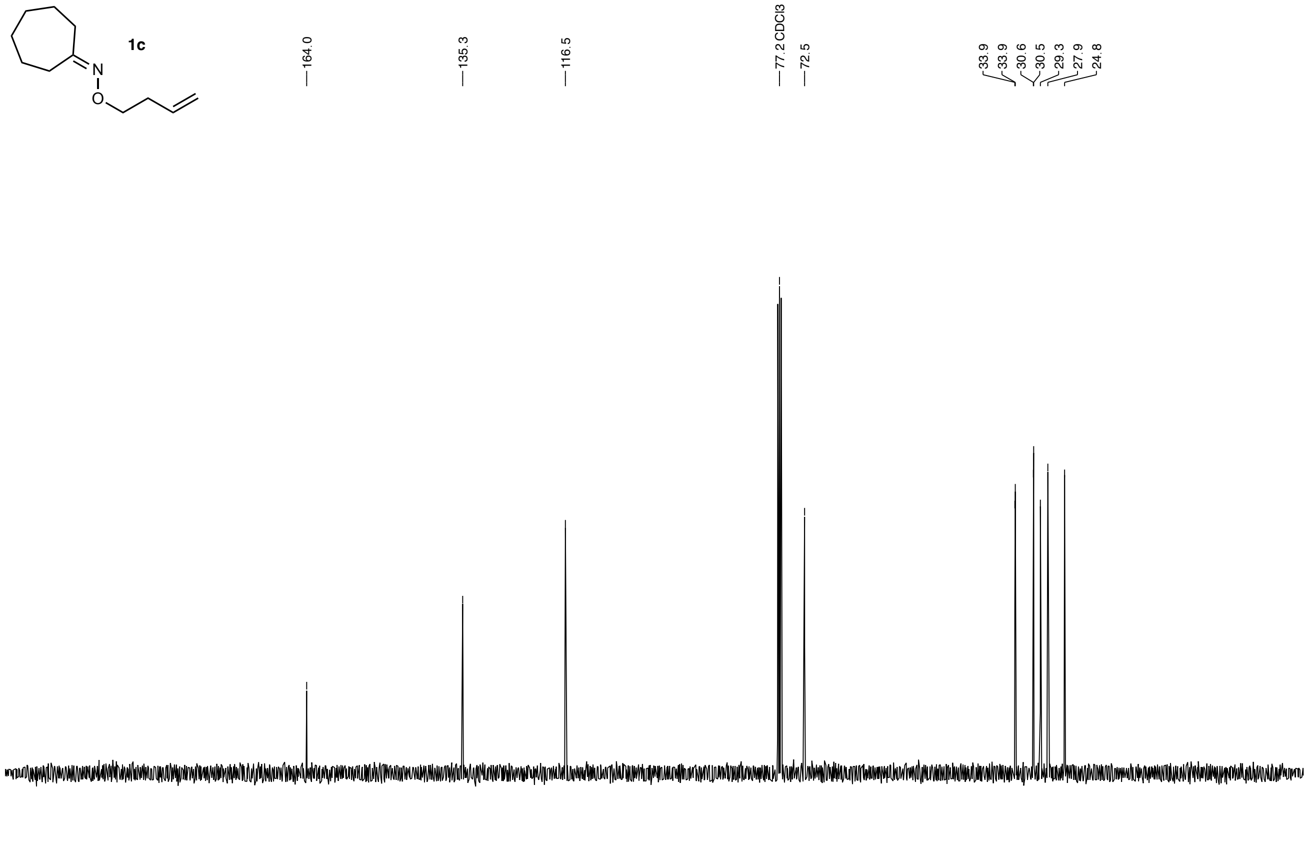
1c
N
O
164.0
135.3
116.5
77.2 CDCl3
72.5
33.9 33.9 30.6 30.5 29.3 27.9 24.8
210 200 190 180 170 160 150 140 130 120 110 100 90 80 70 60 50 40 30 20 10 0 −10
f1 (ppm)

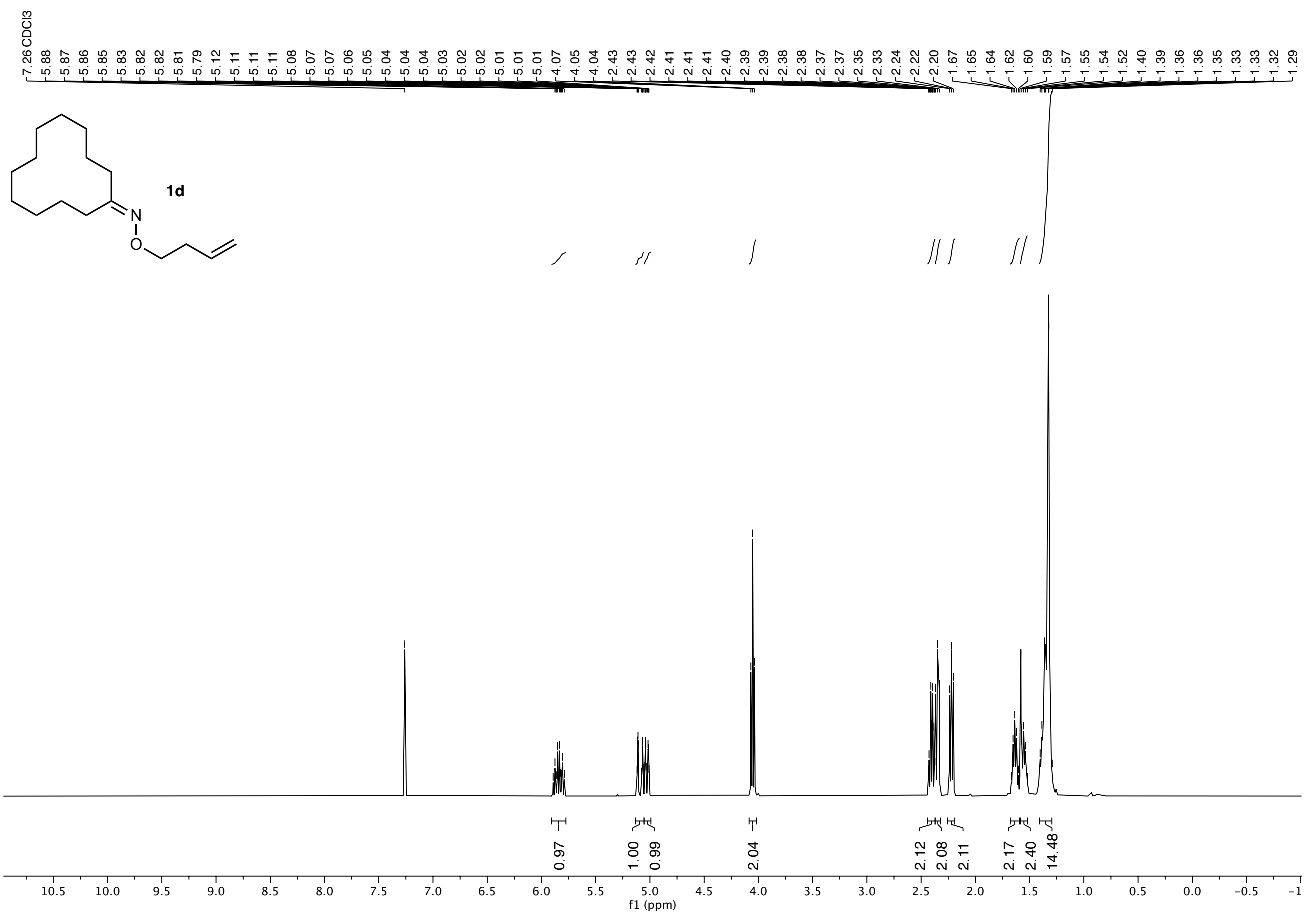
7.26 CDCl3
5.88 5.87 5.86 5.85 5.83 5.82 5.82 5.81 5.79 5.12 5.11 5.11 5.11 5.08 5.07 5.07 5.06 5.05 5.04 5.04 5.04 5.03 5.02 5.02 5.01 5.01 5.01 4.07 4.05 4.04 2.43 2.42 2.41 2.41 2.41 2.40 2.39 2.39 2.38 2.38 2.37 2.37 2.35 2.33 2.24 2.22 2.20 1.67 1.65 1.64 1.62 1.60 1.59 1.57 1.55 1.54 1.52 1.40 1.39 1.36 1.36 1.35 1.33 1.33 1.32 1.29
N
O
1d
0.97
1.00
0.99
2.04
2.12
2.08
2.11
2.17
2.40
14.48
10.5 10.0 9.5 9.0 8.5 8.0 7.5 7.0 6.5 6.0 5.5 5.0 4.5 4.0 3.5 3.0 2.5 2.0 1.5 1.0 0.5 0.0 −0.5 −1
f1 (ppm)

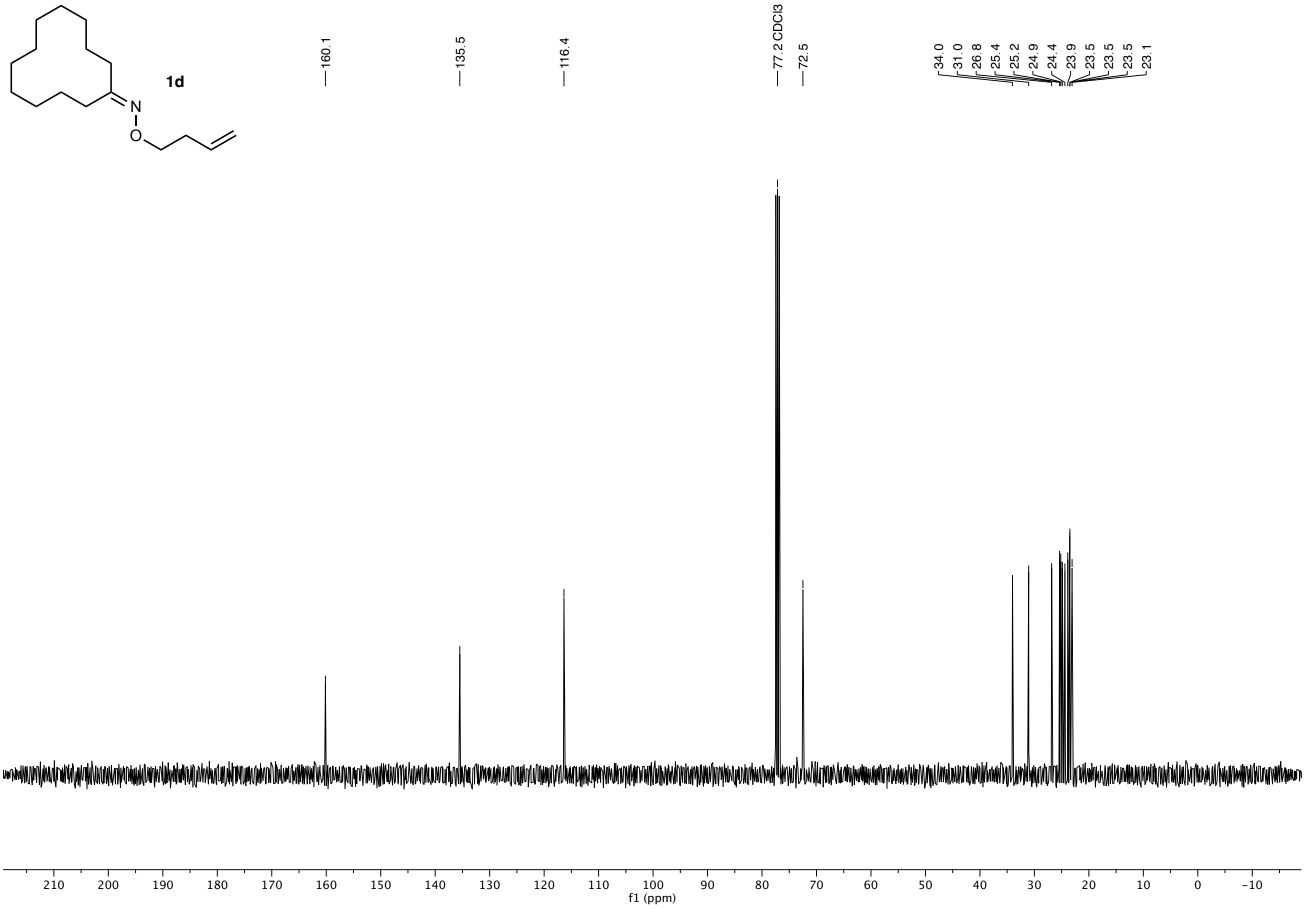
N
O
1d
160.1
135.5
116.4
77.2 CDCl3
72.5
34.0 31.0 26.8 25.4 25.2 24.9 24.4 23.9 23.5 23.5 23.1
210 200 190 180 170 160 150 140 130 120 110 100 90 80 70 60 50 40 30 20 10 0 −10
f1 (ppm)

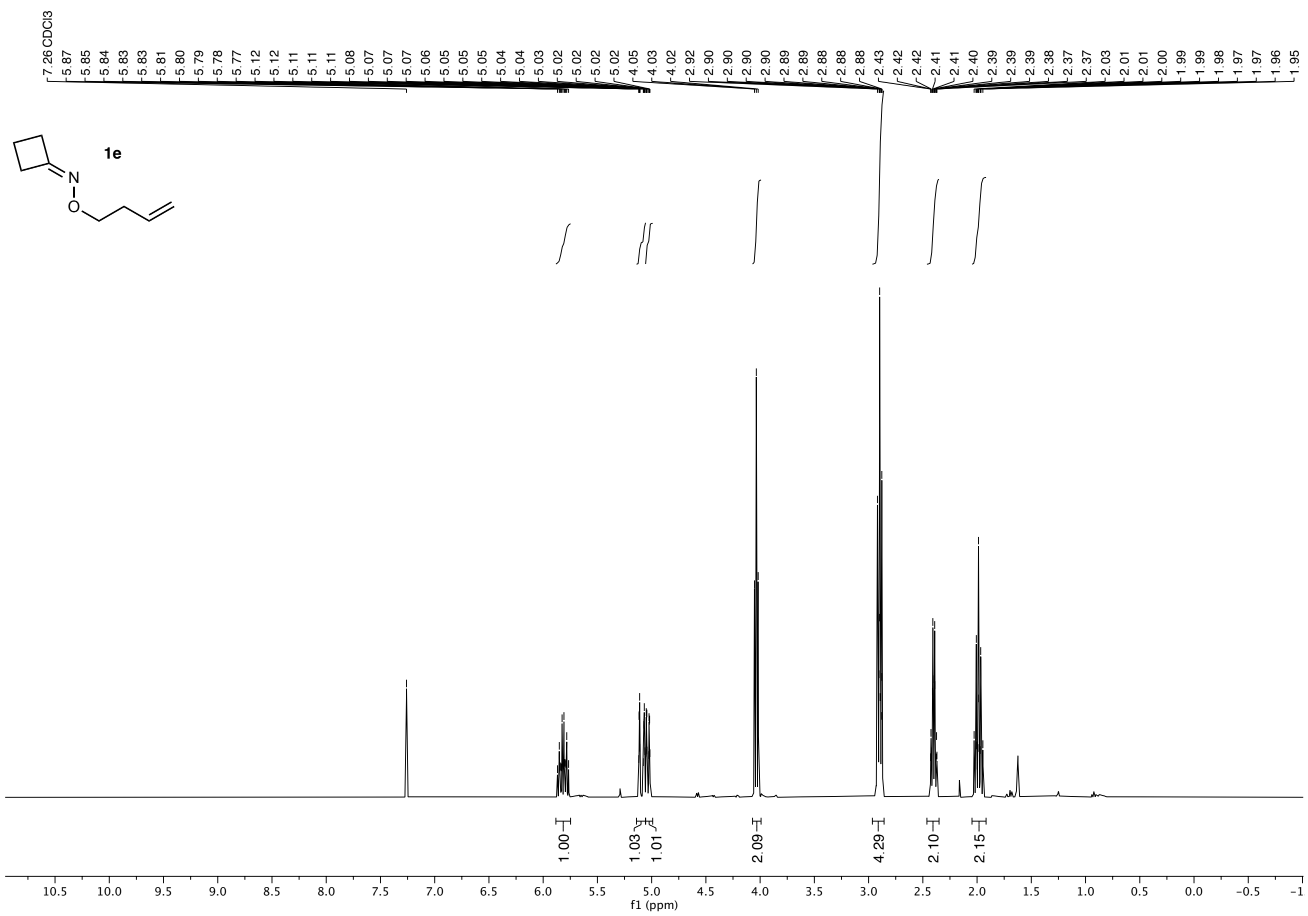
1e
N
O
7.26 CDCl3
5.87 5.85 5.84 5.83 5.83 5.81 5.80 5.79 5.78 5.77 5.12 5.12 5.11 5.11 5.11 5.08 5.07 5.07 5.07 5.06 5.05 5.05 5.05 5.04 5.04 5.03 5.02 5.02 5.02 5.02 4.05 4.03 4.02 2.92 2.90 2.90 2.90 2.90 2.89 2.89 2.88 2.88 2.88 2.43 2.42 2.42 2.41 2.41 2.40 2.39 2.39 2.39 2.38 2.37 2.37 2.03 2.01 2.01 2.00 1.99 1.99 1.98 1.97 1.97 1.96 1.95
1.00
1.03
1.01
2.09
4.29
2.10
2.15
10.5 10.0 9.5 9.0 8.5 8.0 7.5 7.0 6.5 6.0 5.5 5.0 4.5 4.0 3.5 3.0 2.5 2.0 1.5 1.0 0.5 0.0 −0.5 −1
f1 (ppm)

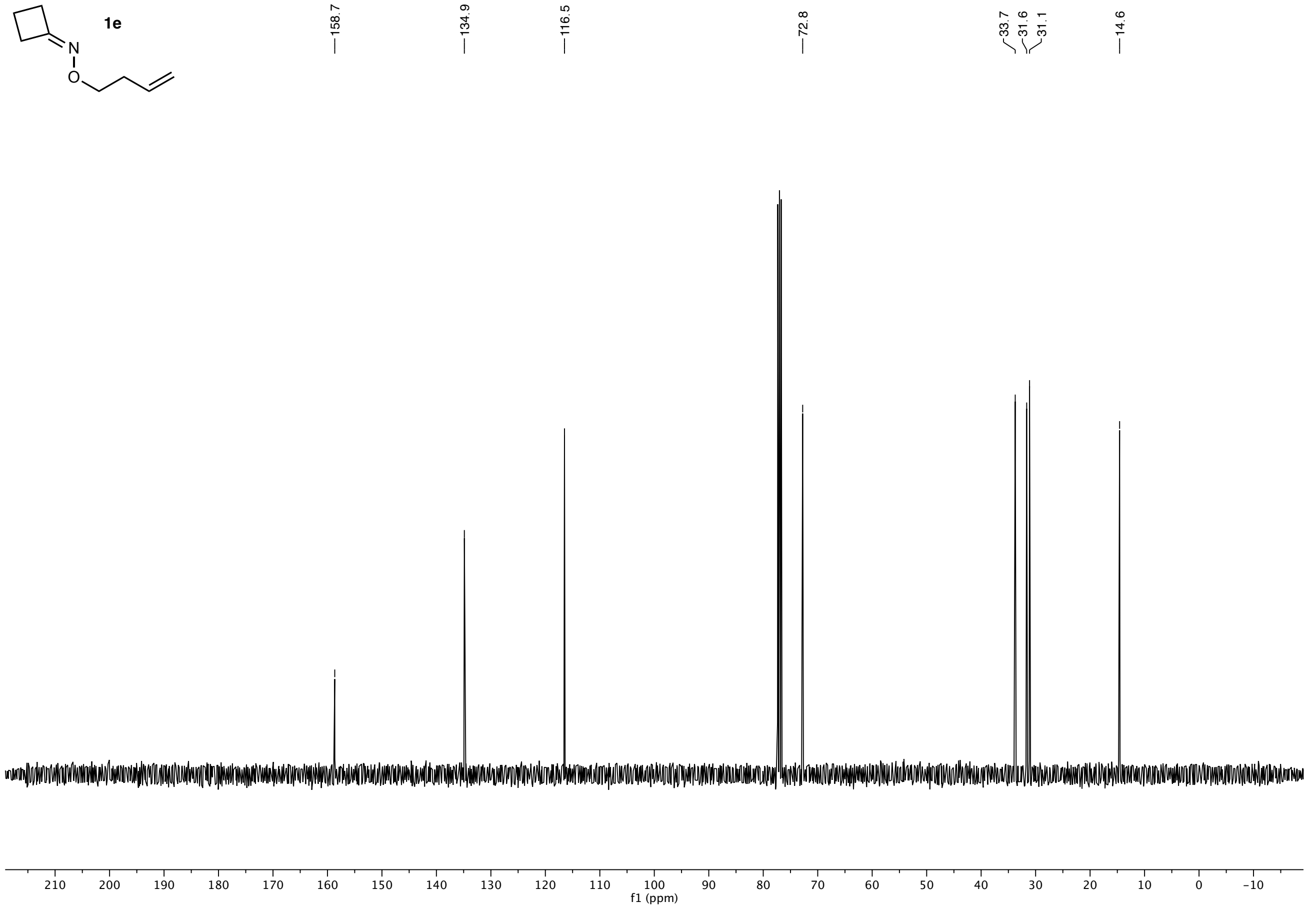
1e
N
O
158.7
134.9
116.5
72.8
33.7
31.6
31.1
14.6
210 200 190 180 170 160 150 140 130 120 110 100 90 80 70 60 50 40 30 20 10 0 −10
f1 (ppm)

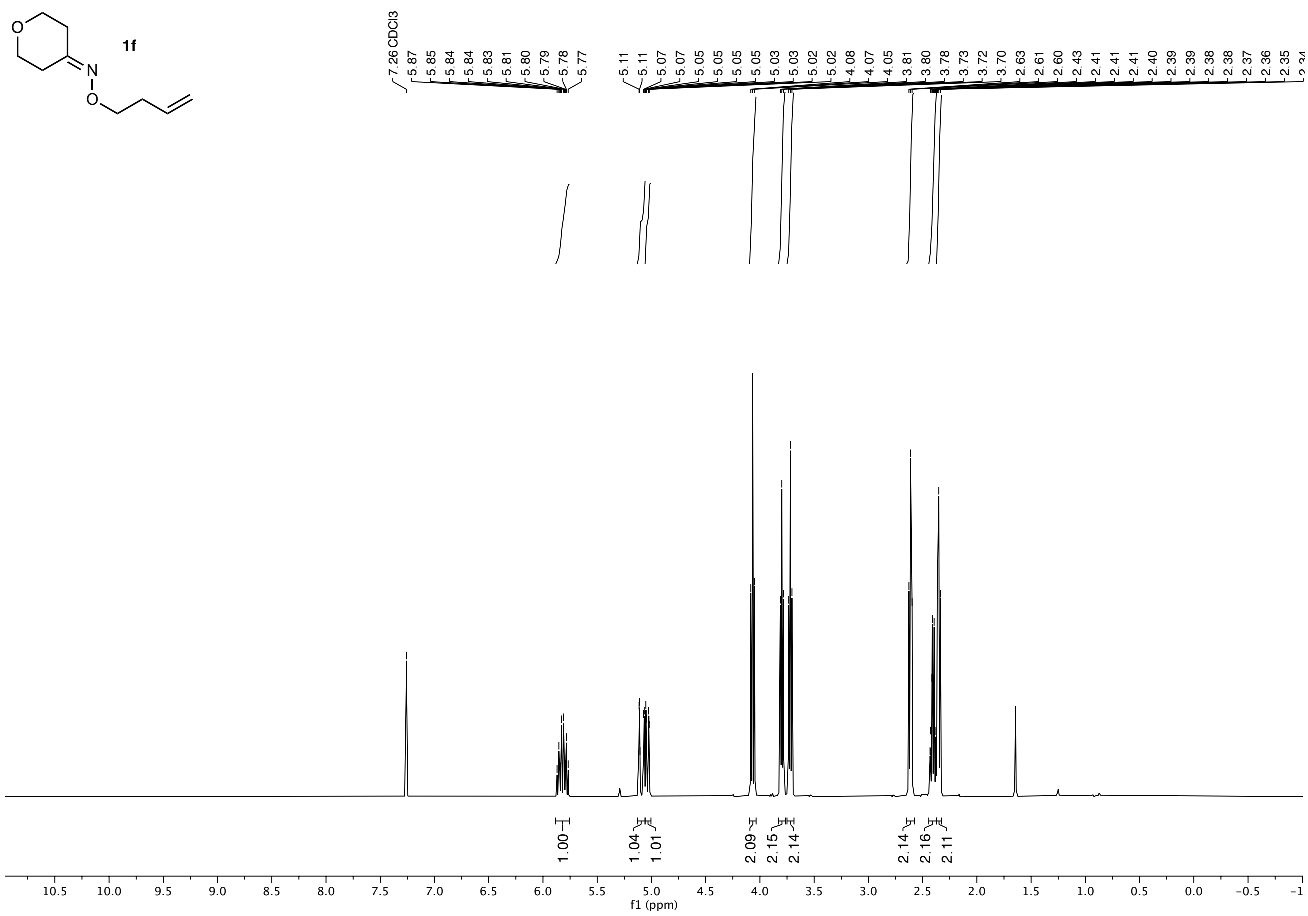
1f
O
N
7.26 CDCl3
5.87
5.85
5.84
5.84
5.83
5.81
5.80
5.79
5.78
5.77
5.11
5.11
5.07
5.07
5.05
5.05
5.05
5.05
5.03
5.03
5.02
5.02
4.08
4.07
4.05
3.81
3.80
3.78
3.73
3.72
3.70
2.63
2.61
2.60
2.43
2.41
2.41
2.41
2.40
2.39
2.39
2.38
2.38
2.37
2.36
2.35
1.00
1.04
1.01
2.09
2.15
2.14
2.14
2.16
2.11
f1 (ppm)

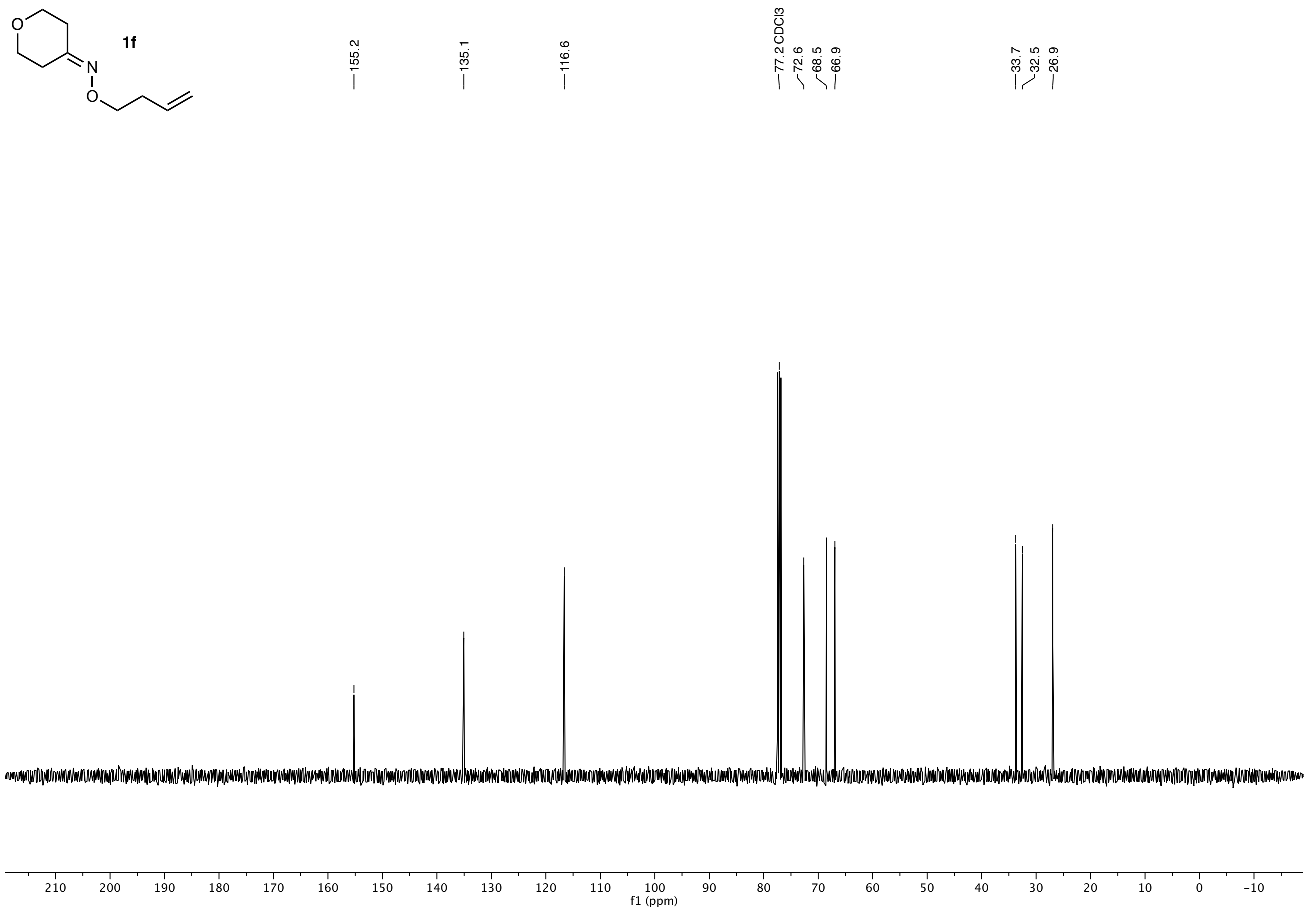
1f
O
N
155.2
135.1
116.6
77.2 CDCl3
72.6
68.5
66.9
33.7
32.5
26.9
f1 (ppm)

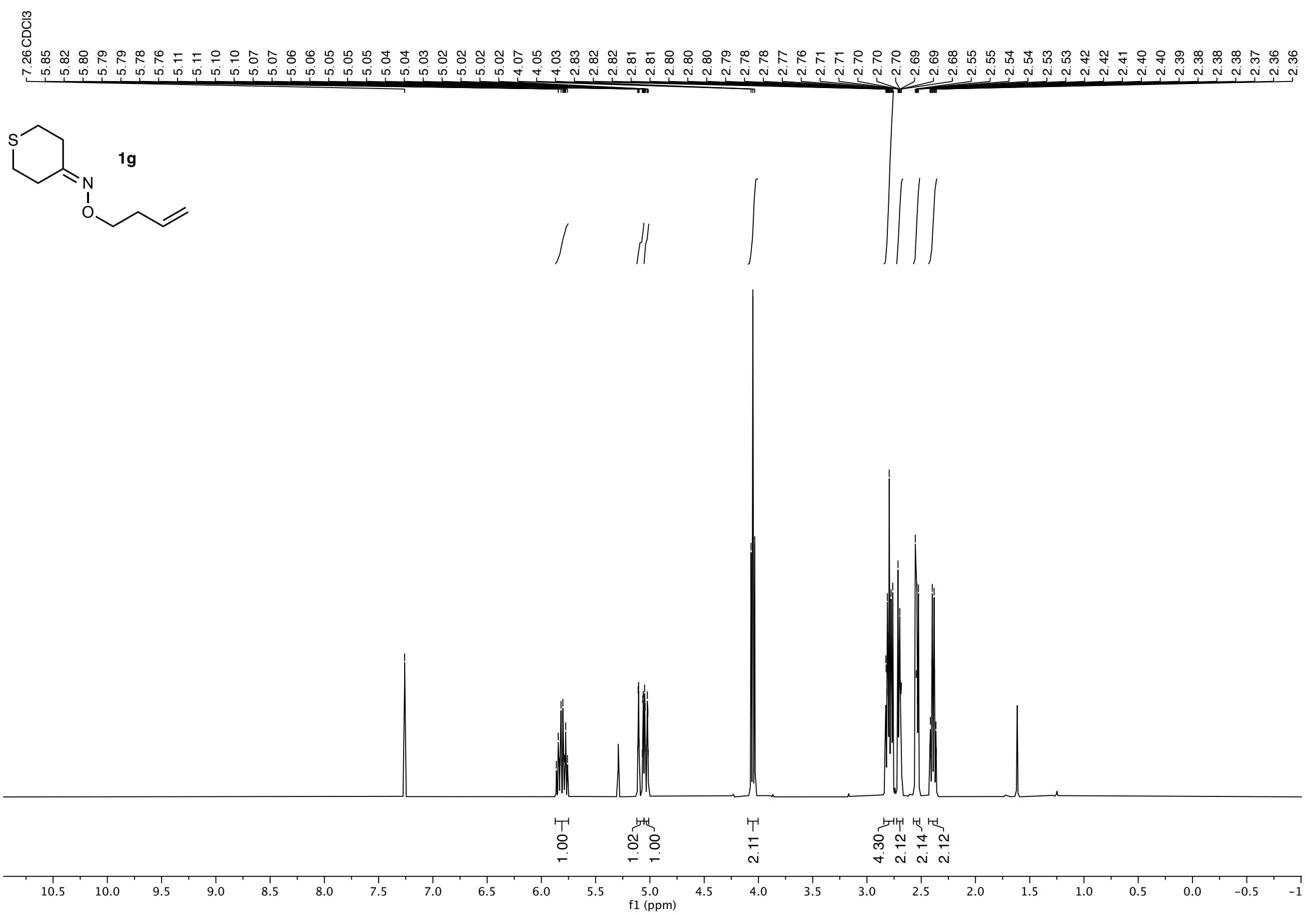
7.26 CDCl3
5.85
5.82
5.80
5.79
5.78
5.76
5.11
5.10
5.10
5.07
5.07
5.06
5.06
5.05
5.05
5.05
5.04
5.04
5.03
5.02
5.02
5.02
5.02
4.07
4.05
4.03
2.83
2.82
2.82
2.81
2.81
2.80
2.80
2.80
2.79
2.78
2.78
2.77
2.76
2.71
2.71
2.70
2.70
2.70
2.69
2.69
2.68
2.55
2.54
2.54
2.53
2.53
2.42
2.42
2.41
2.40
2.40
2.39
2.38
2.38
2.38
2.37
2.36
2.36
S
N
O
1g
1.00
1.02
1.00
2.11
4.30
2.12
2.14
2.12
10.5
10.0
9.5
9.0
8.5
8.0
7.5
7.0
6.5
6.0
5.5
5.0
4.5
4.0
3.5
3.0
2.5
2.0
1.5
1.0
0.5
0.0
-0.5
-1
f1 (ppm)

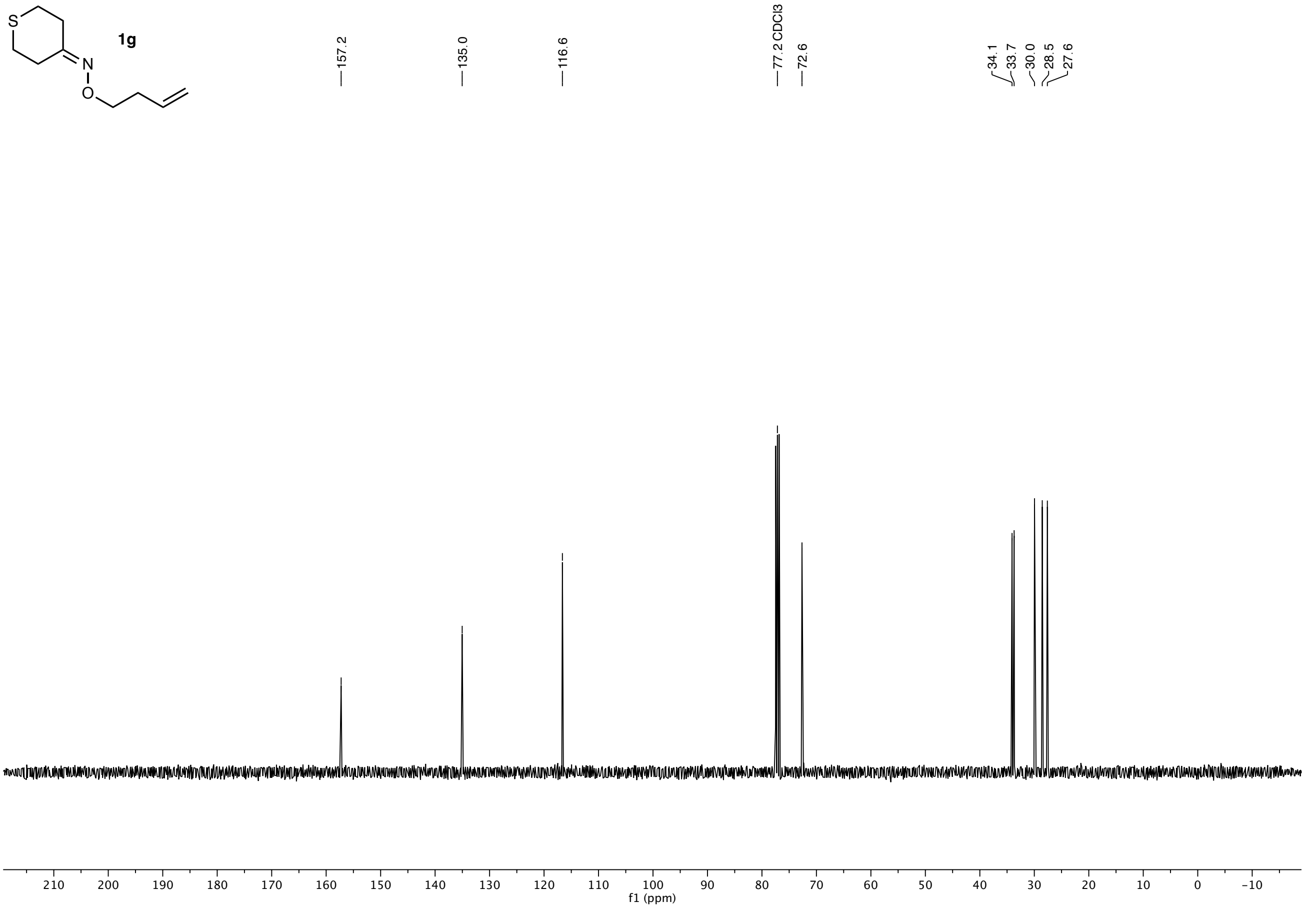
S
N
O
1g
157.2
135.0
116.6
77.2 CDCl3
72.6
34.1
33.7
30.0
28.5
27.6
210
200
190
180
170
160
150
140
130
120
110
100
90
80
70
60
50
40
30
20
10
0
-10
f1 (ppm)

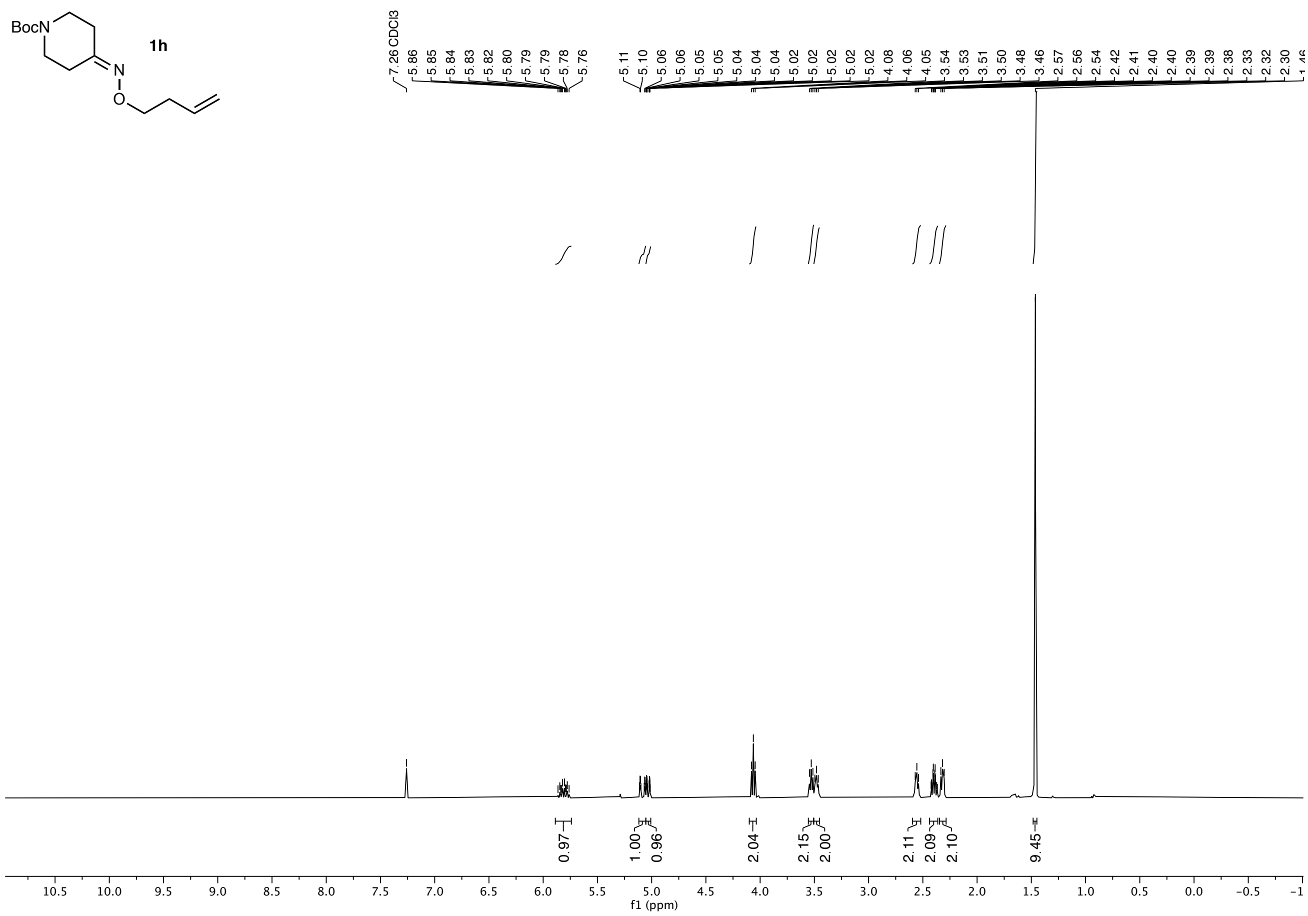

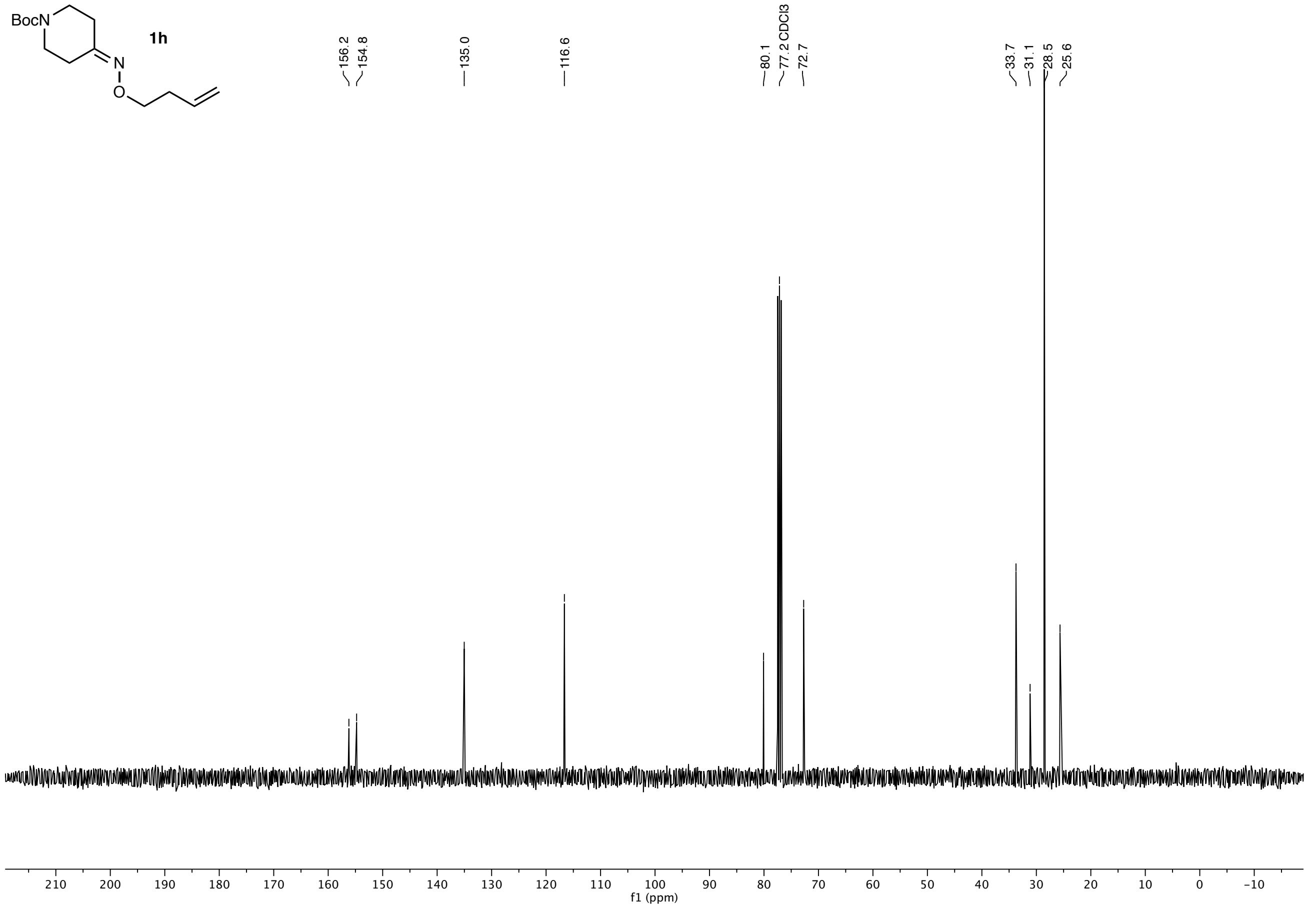

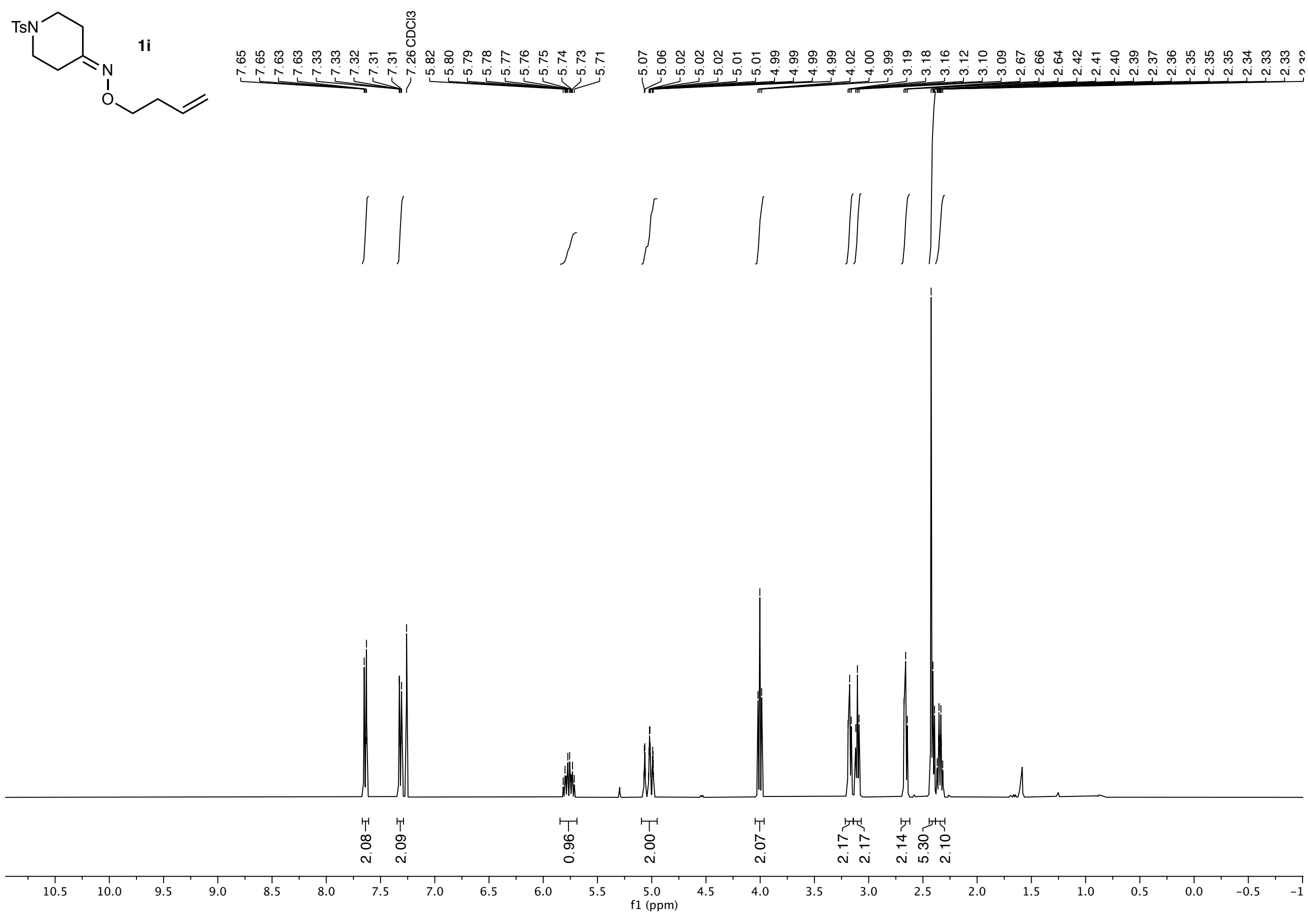
TsN
N
O
1i
7.65 7.65 7.63 7.63 7.33 7.32 7.31 7.31 7.26 CDCl3 5.82 5.80 5.79 5.78 5.77 5.76 5.75 5.74 5.73 5.71 5.07 5.06 5.02 5.02 5.01 5.01 4.99 4.99 4.99 4.99 4.02 4.00 3.99 3.19 3.18 3.16 3.12 3.10 3.09 2.67 2.66 2.64 2.42 2.41 2.40 2.39 2.37 2.36 2.35 2.35 2.35 2.34 2.33 2.33
2.08 2.09 0.96 2.00 2.07 2.17 2.17 2.14 5.30 2.10
10.5 10.0 9.5 9.0 8.5 8.0 7.5 7.0 6.5 6.0 5.5 5.0 4.5 4.0 3.5 3.0 2.5 2.0 1.5 1.0 0.5 0.0 −0.5
f1 (ppm)

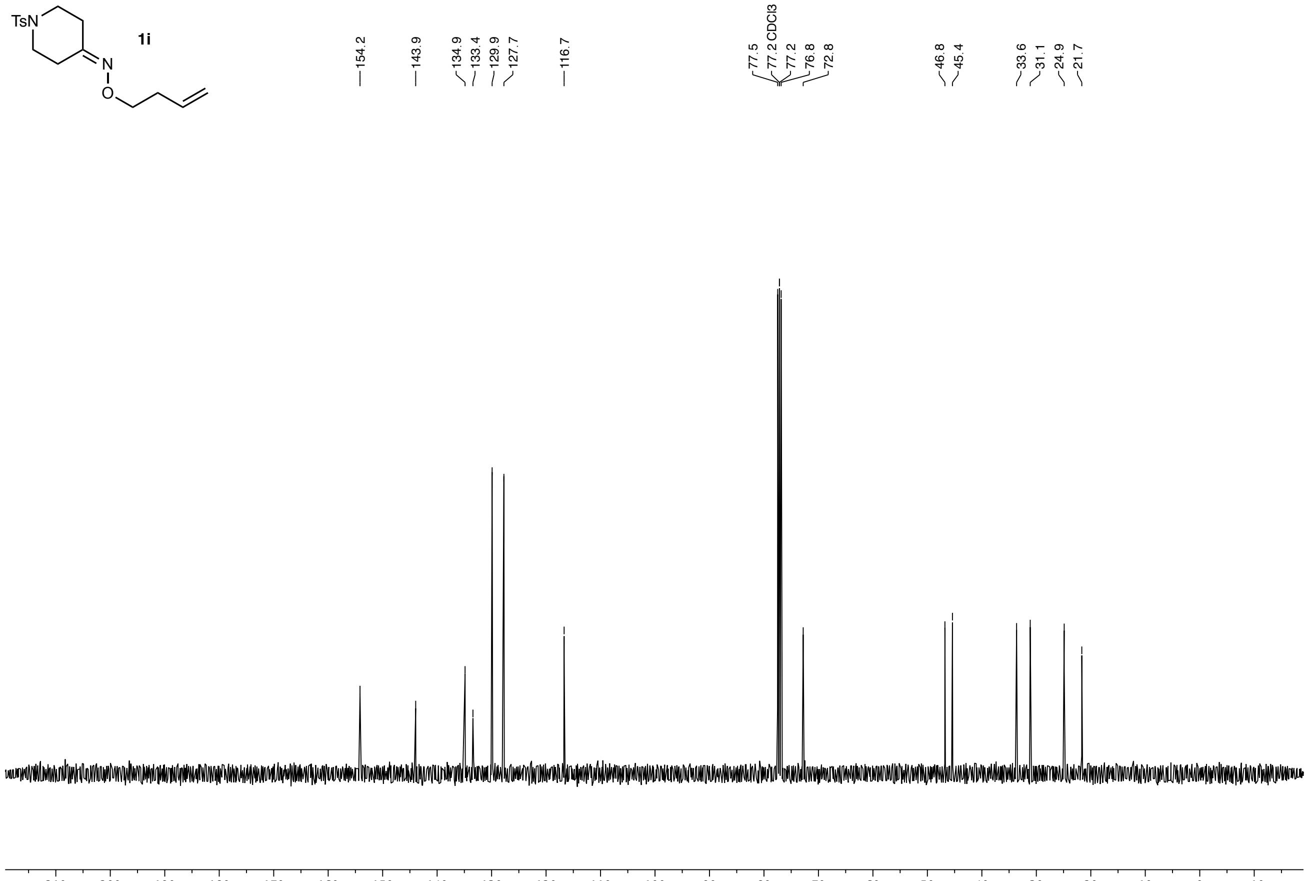
TsN
N
O
1i
154.2 143.9 134.9 133.4 129.9 127.7 116.7 77.5 77.2 CDCl3 77.2 76.8 72.8 46.8 45.4 33.6 31.1 24.9 21.7
210 200 190 180 170 160 150 140 130 120 110 100 90 80 70 60 50 40 30 20 10 0 −10
f1 (ppm)

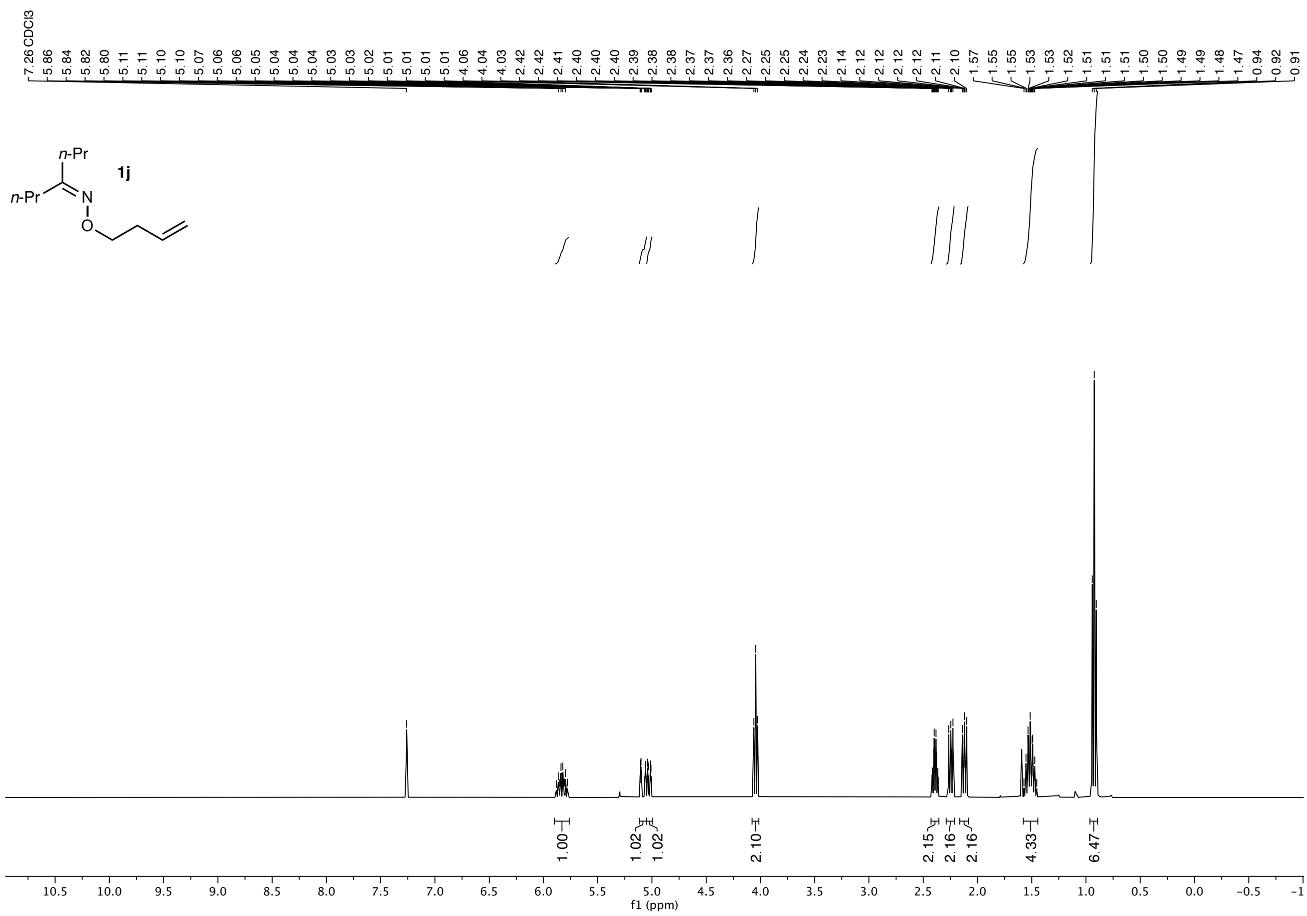
7.26 CDCl3
5.86
5.84
5.82
5.80
5.11
5.11
5.10
5.10
5.07
5.06
5.06
5.05
5.04
5.04
5.04
5.03
5.03
5.02
5.01
5.01
5.01
5.01
4.06
4.04
4.03
2.42
2.42
2.41
2.40
2.40
2.40
2.39
2.38
2.38
2.37
2.37
2.36
2.27
2.25
2.24
2.23
2.14
2.12
2.12
2.12
2.12
2.11
2.10
1.57
1.55
1.55
1.53
1.53
1.52
1.51
1.51
1.51
1.50
1.49
1.49
1.48
1.47
0.94
0.92
0.91
n-Pr
1j
n-Pr
N
O
1.00
1.02
1.02
2.10
2.15
2.16
2.16
4.33
6.47
10.5
10.0
9.5
9.0
8.5
8.0
7.5
7.0
6.5
6.0
5.5
5.0
4.5
4.0
3.5
3.0
2.5
2.0
1.5
1.0
0.5
0.0
-0.5
-1
f1 (ppm)

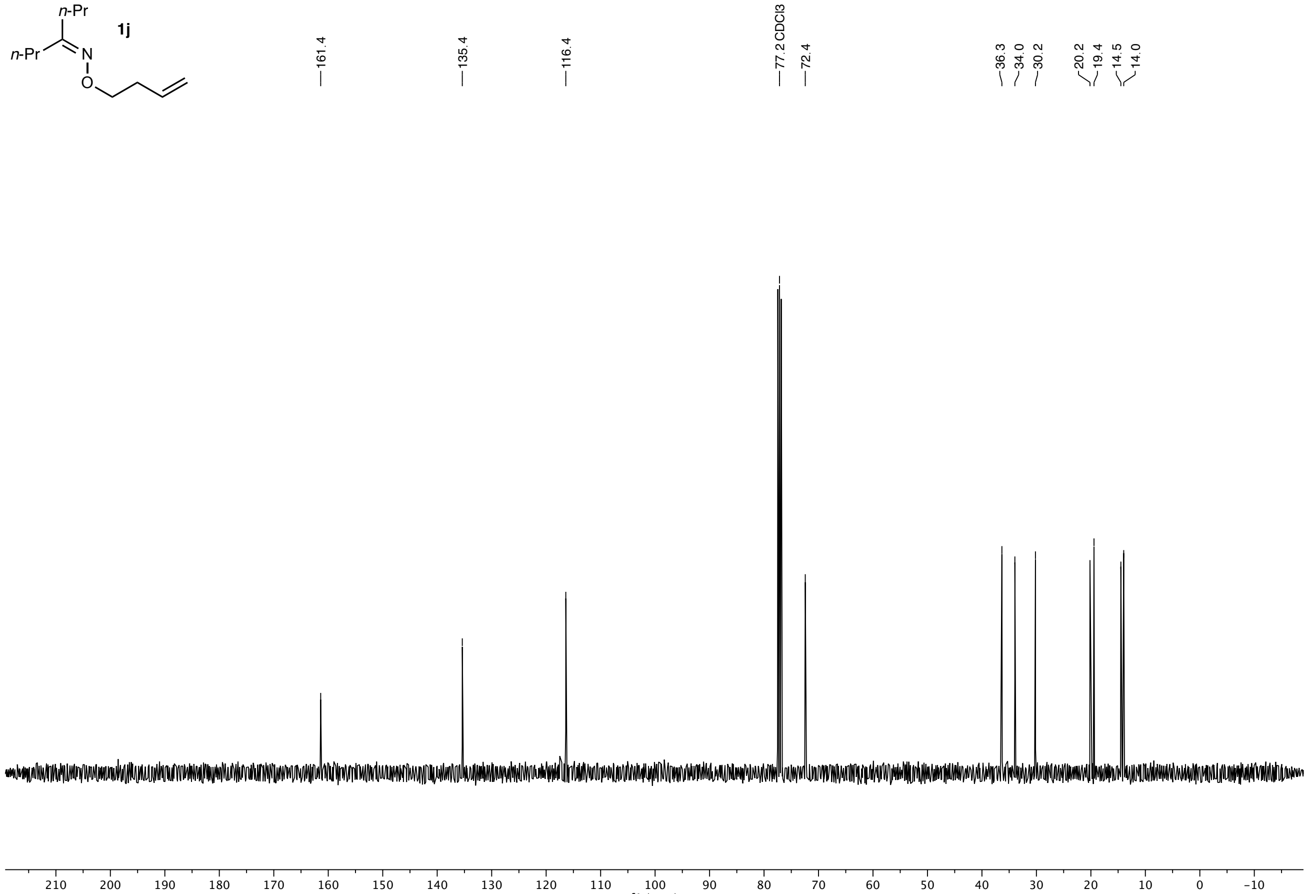
n-Pr
1j
n-Pr
N
O
161.4
135.4
116.4
77.2 CDCl3
72.4
36.3
34.0
30.2
20.2
19.4
14.5
14.0
210
200
190
180
170
160
150
140
130
120
110
100
90
80
70
60
50
40
30
20
10
0
-10
f1 (ppm)

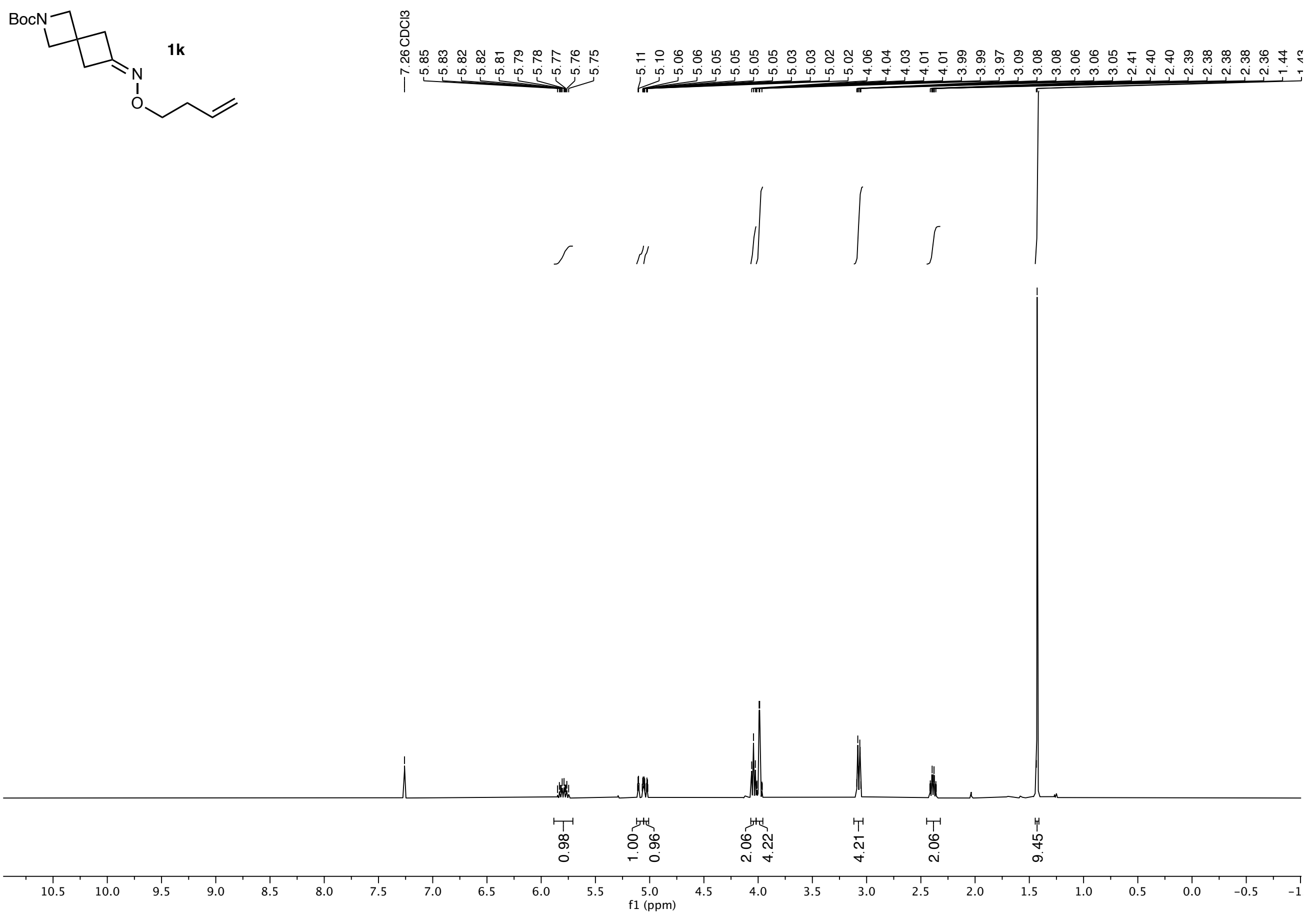
BocN
N
O
1k
7.26 CDCl3
5.85 5.83 5.82 5.82 5.81 5.79 5.78 5.77 5.76 5.75
5.11 5.10 5.06 5.06 5.05 5.05 5.05 5.05 5.03 5.03 5.02 5.02 4.06 4.04 4.03 4.01 4.01 3.99 3.99 3.97 3.09 3.08 3.08 3.06 3.06 3.05 2.41 2.40 2.40 2.39 2.38 2.38 2.38 2.36 1.44
0.98
1.00
0.96
2.06
4.22
4.21
2.06
9.45
10.5 10.0 9.5 9.0 8.5 8.0 7.5 7.0 6.5 6.0 5.5 5.0 4.5 4.0 3.5 3.0 2.5 2.0 1.5 1.0 0.5 0.0 −0.5
f1 (ppm)

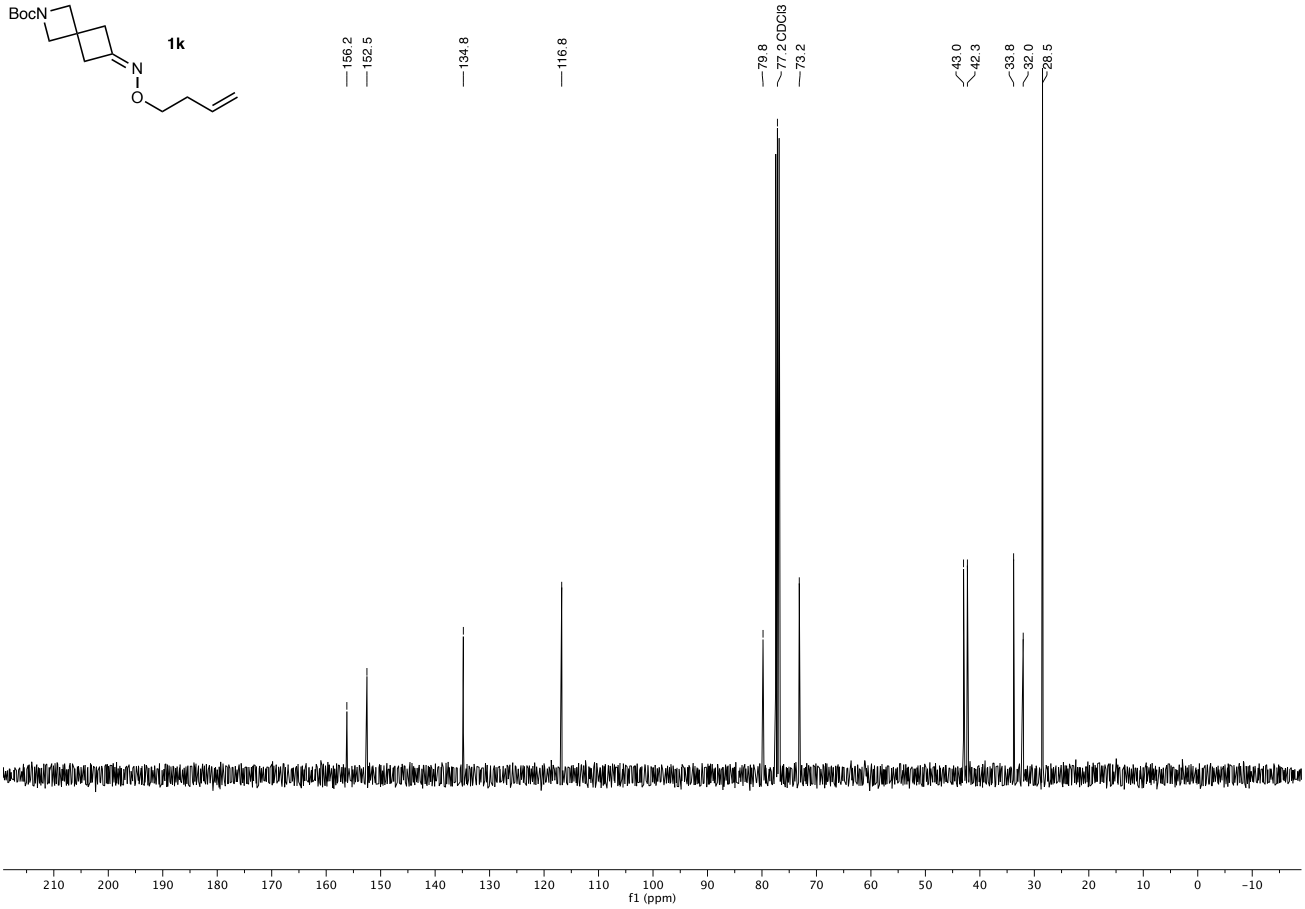
BocN
N
O
1k
156.2
152.5
134.8
116.8
79.8
77.2 CDCl3
73.2
43.0
42.3
33.8
32.0
28.5
210 200 190 180 170 160 150 140 130 120 110 100 90 80 70 60 50 40 30 20 10 0 −10
f1 (ppm)

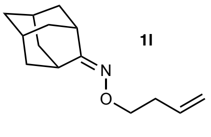

1l

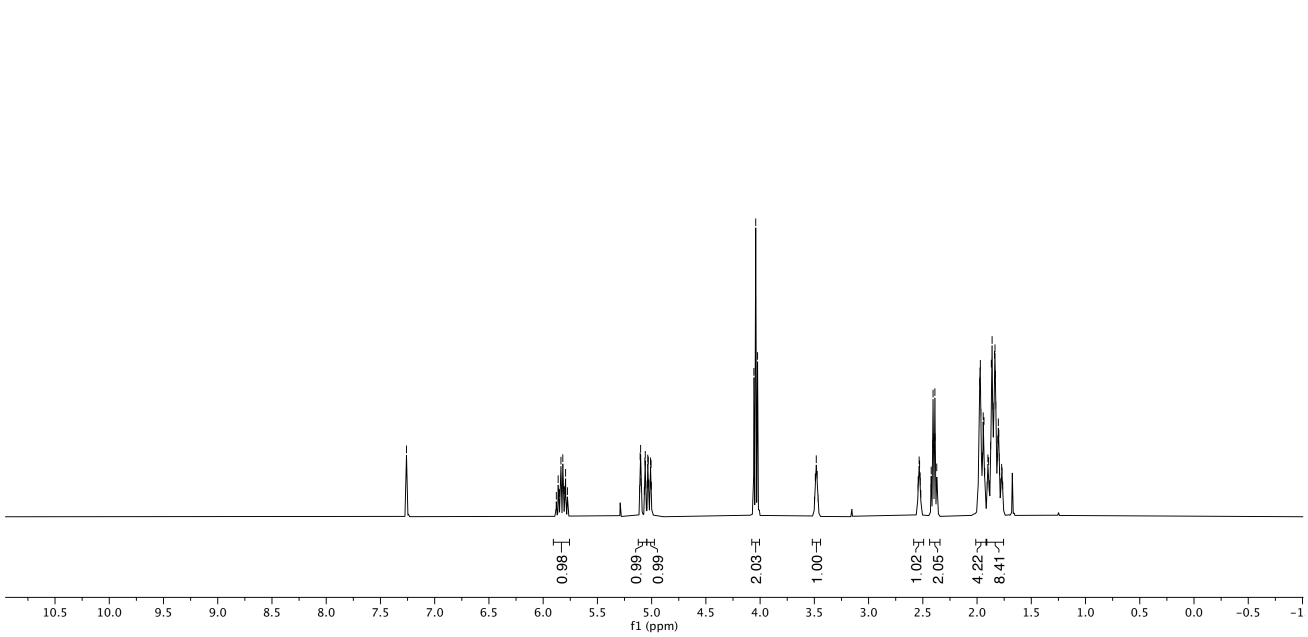
7.26 CDCl3
5.86
5.84
5.82
5.79
5.11
5.10
5.10
5.06
5.06
5.05
5.04
5.04
5.03
5.03
5.03
5.01
5.01
5.01
5.01
4.06
4.04
4.02
3.49
3.48
3.47
2.54
2.53
2.53
2.52
2.52
2.42
2.42
2.41
2.41
2.40
2.39
2.39
2.39
2.38
2.37
2.37
1.98
1.97
1.96
1.95
1.94
1.94
1.93
1.90
1.89
1.89
1.88
1.87
1.86
1.85
1.84
1.83
1.83
1.83
1.81
1.80
1.80
1.79
1.78
1.77
1.77
0.98
0.99
0.99
2.03
1.00
1.02
2.05
4.22
8.41
f1 (ppm)

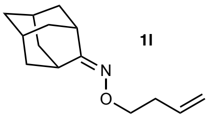

1l

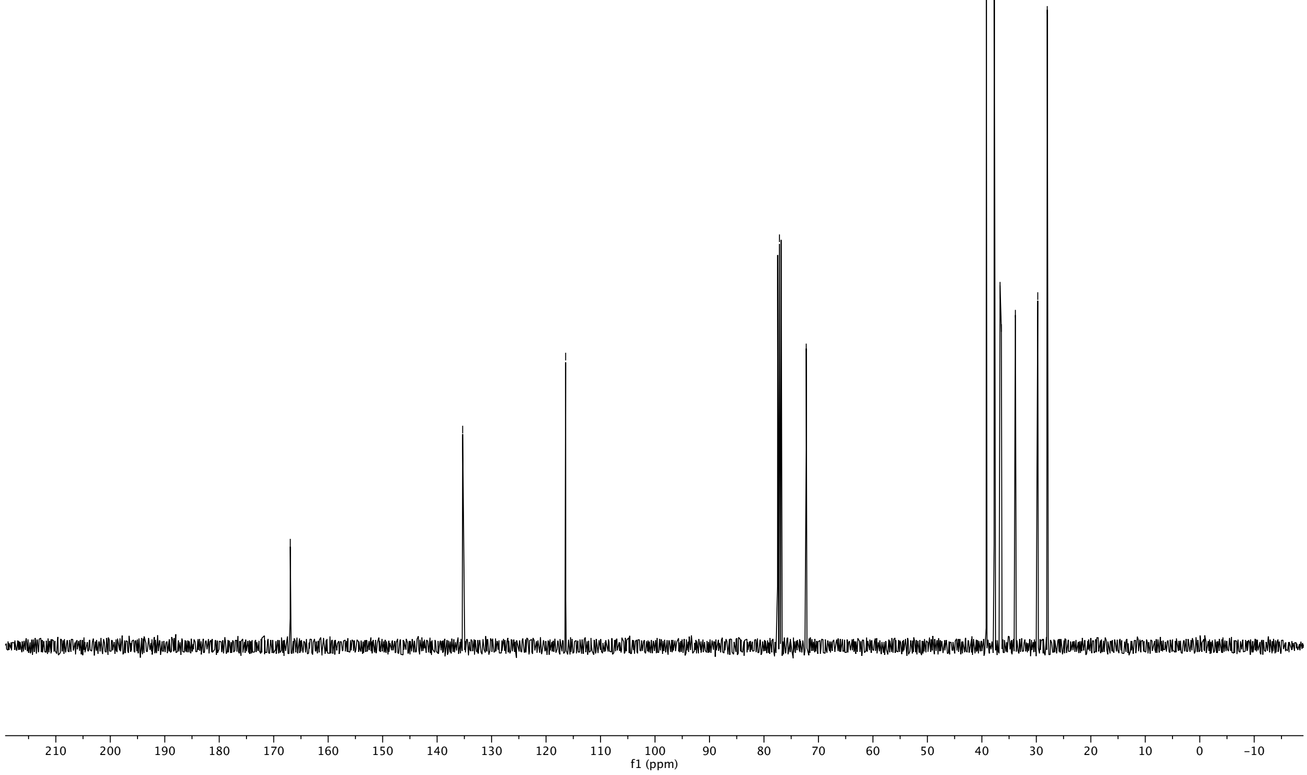
167.0
135.3
116.4
77.2 CDCl3
72.2
39.2
37.7
36.7
36.4
33.8
29.7
28.0
f1 (ppm)

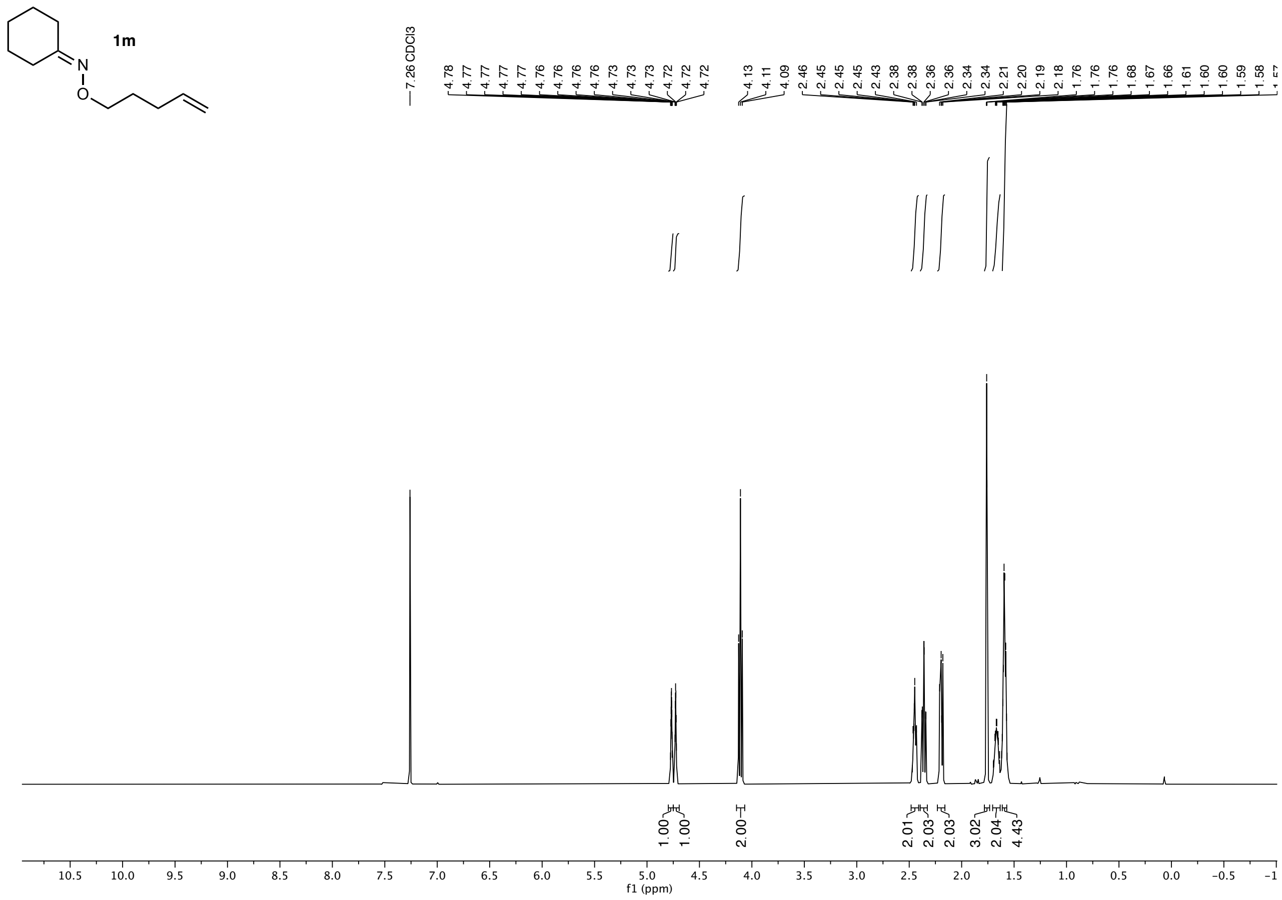
1m
7.26 CDCl3
4.78
4.77
4.77
4.77
4.77
4.76
4.76
4.76
4.76
4.73
4.73
4.73
4.72
4.72
4.72
4.72
4.13
4.11
4.09
2.46
2.45
2.45
2.45
2.43
2.38
2.38
2.36
2.36
2.34
2.34
2.21
2.20
2.19
2.18
1.76
1.76
1.76
1.68
1.67
1.66
1.61
1.60
1.60
1.59
1.58
1.00
1.00
2.00
2.01
2.03
2.03
3.02
2.04
4.43
f1 (ppm)

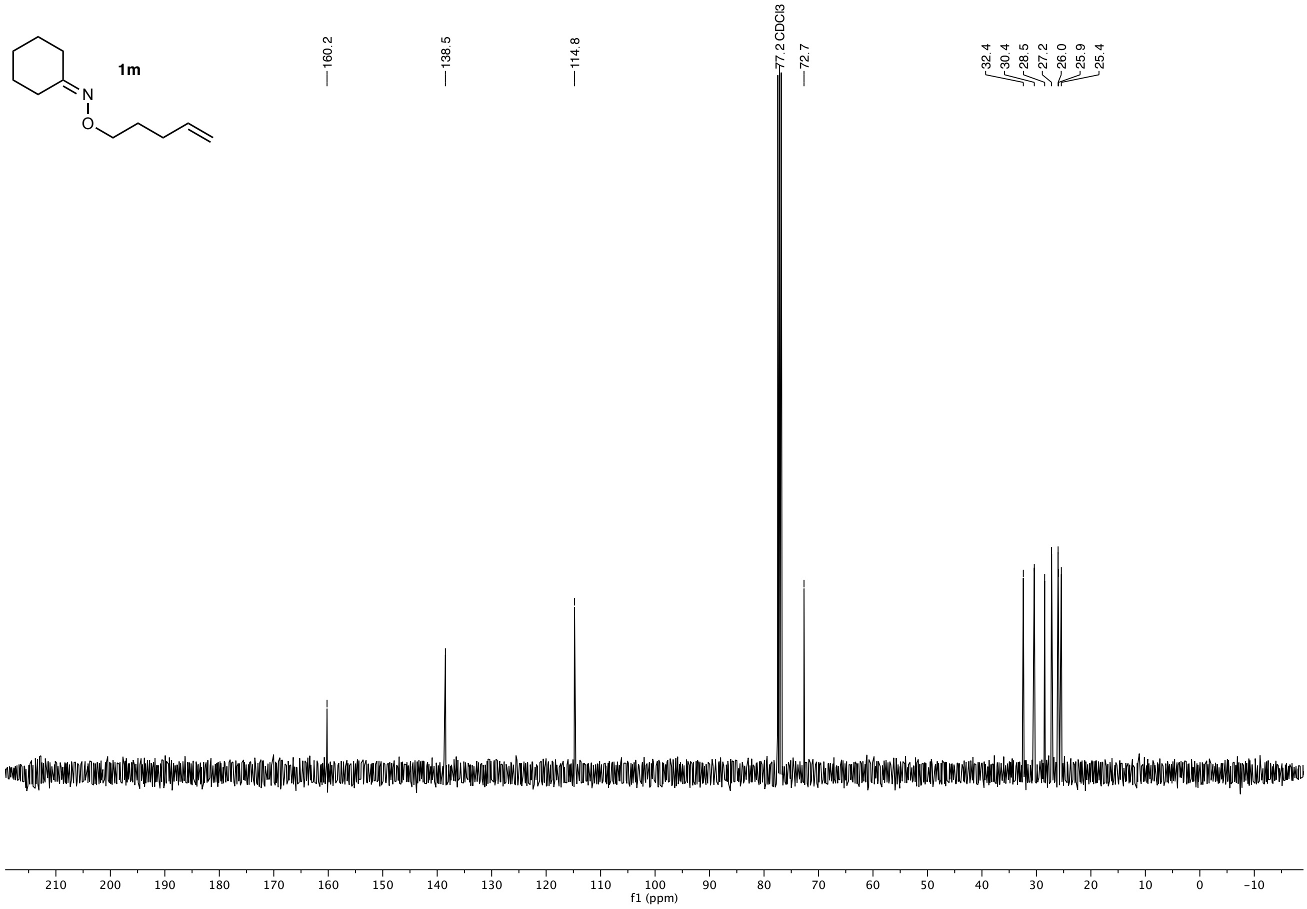
1m
160.2
138.5
114.8
77.2 CDCl3
72.7
32.4
30.4
28.5
27.2
26.0
25.9
25.4
f1 (ppm)

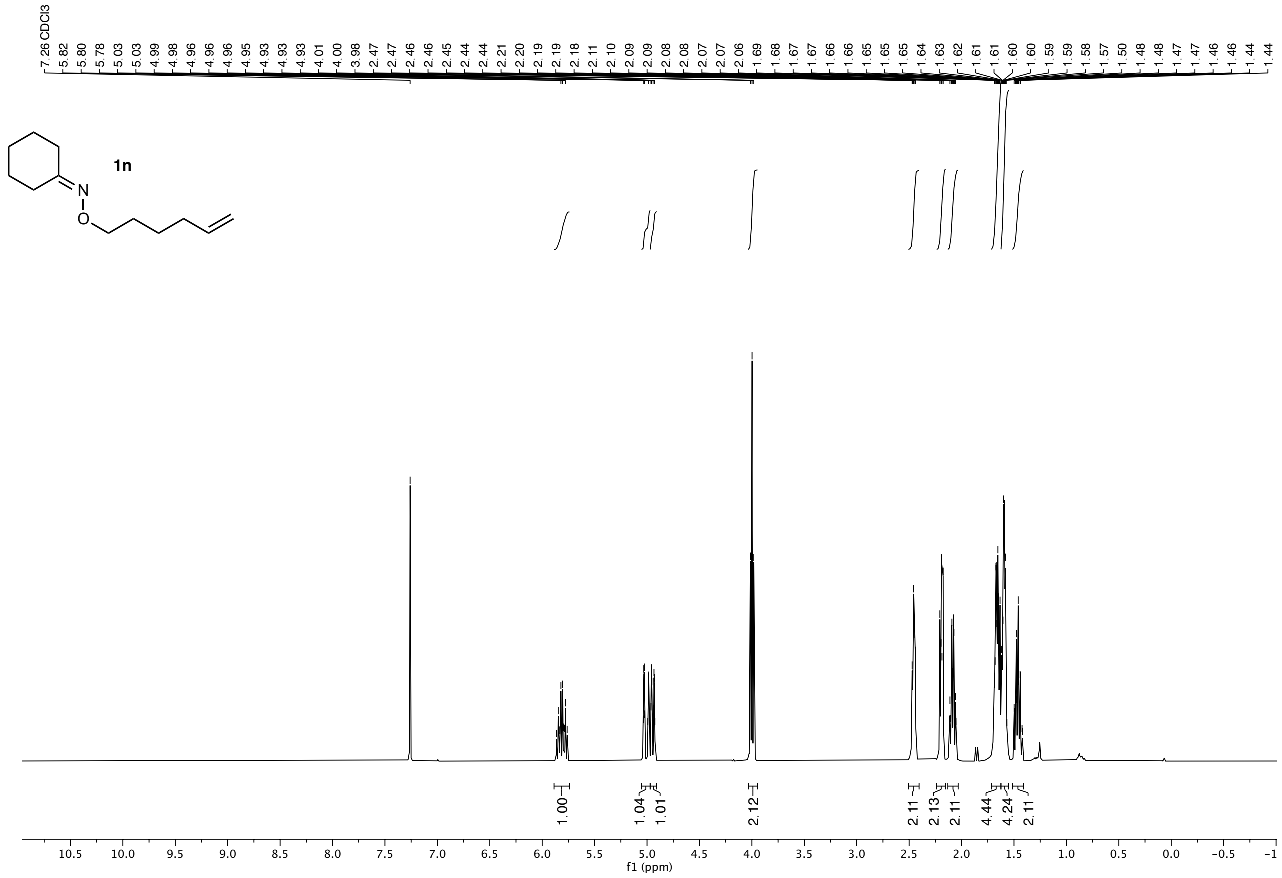
7.26 CDCl3
5.82
5.80
5.78
5.03
5.03
4.99
4.98
4.96
4.96
4.95
4.93
4.93
4.93
4.01
4.00
3.98
2.47
2.46
2.46
2.45
2.44
2.44
2.21
2.20
2.19
2.19
2.18
2.11
2.10
2.09
2.09
2.08
2.08
2.07
2.07
2.06
1.69
1.68
1.67
1.67
1.66
1.66
1.65
1.65
1.64
1.63
1.62
1.61
1.61
1.60
1.60
1.59
1.59
1.58
1.57
1.50
1.48
1.47
1.47
1.46
1.46
1.44
1.44
1n
N
O
1.00
1.04
1.01
2.12
2.11
2.13
2.11
4.44
4.24
2.11
f1 (ppm)

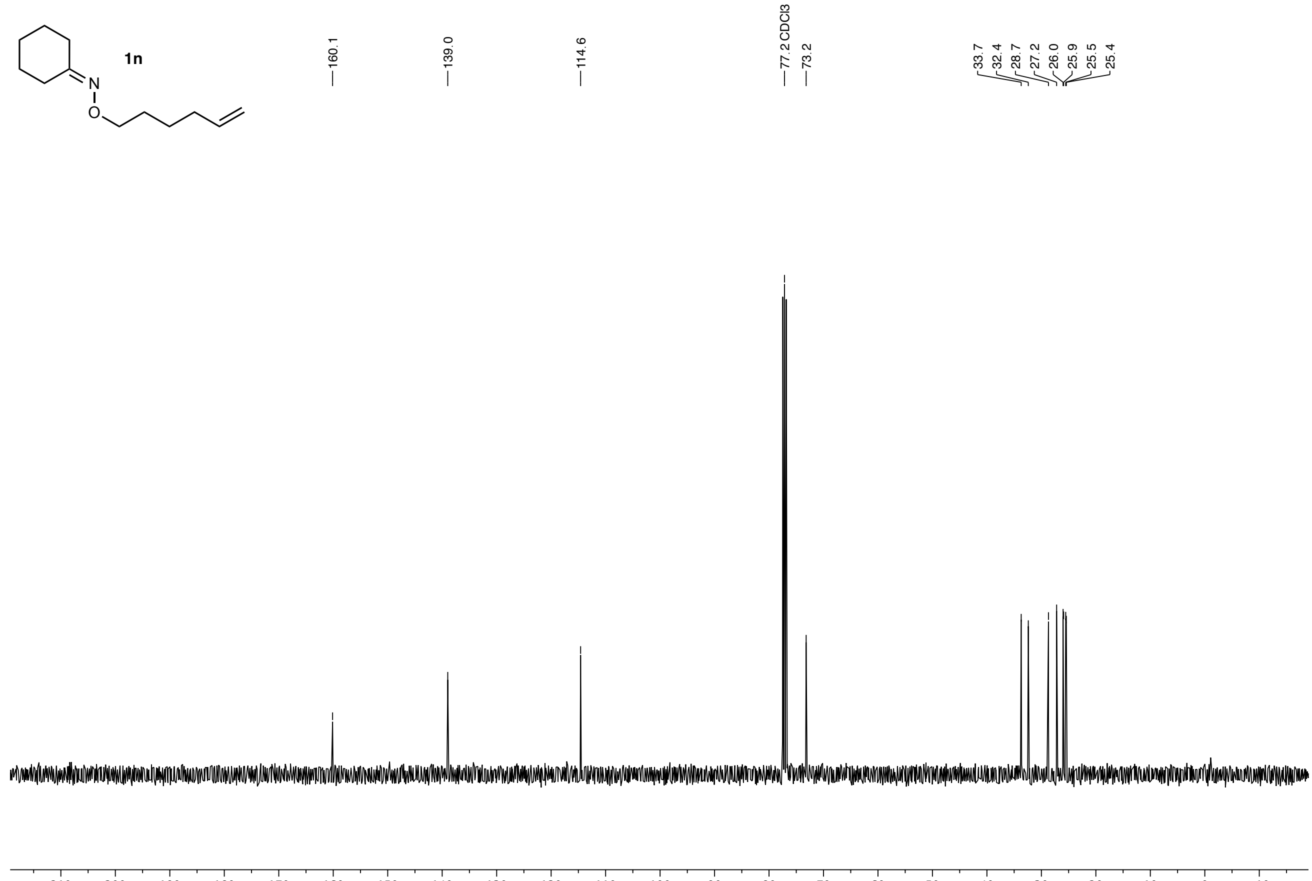
1n
N
O
160.1
139.0
114.6
77.2 CDCl3
73.2
33.7
32.4
28.7
27.2
26.0
25.9
25.5
25.4
f1 (ppm)

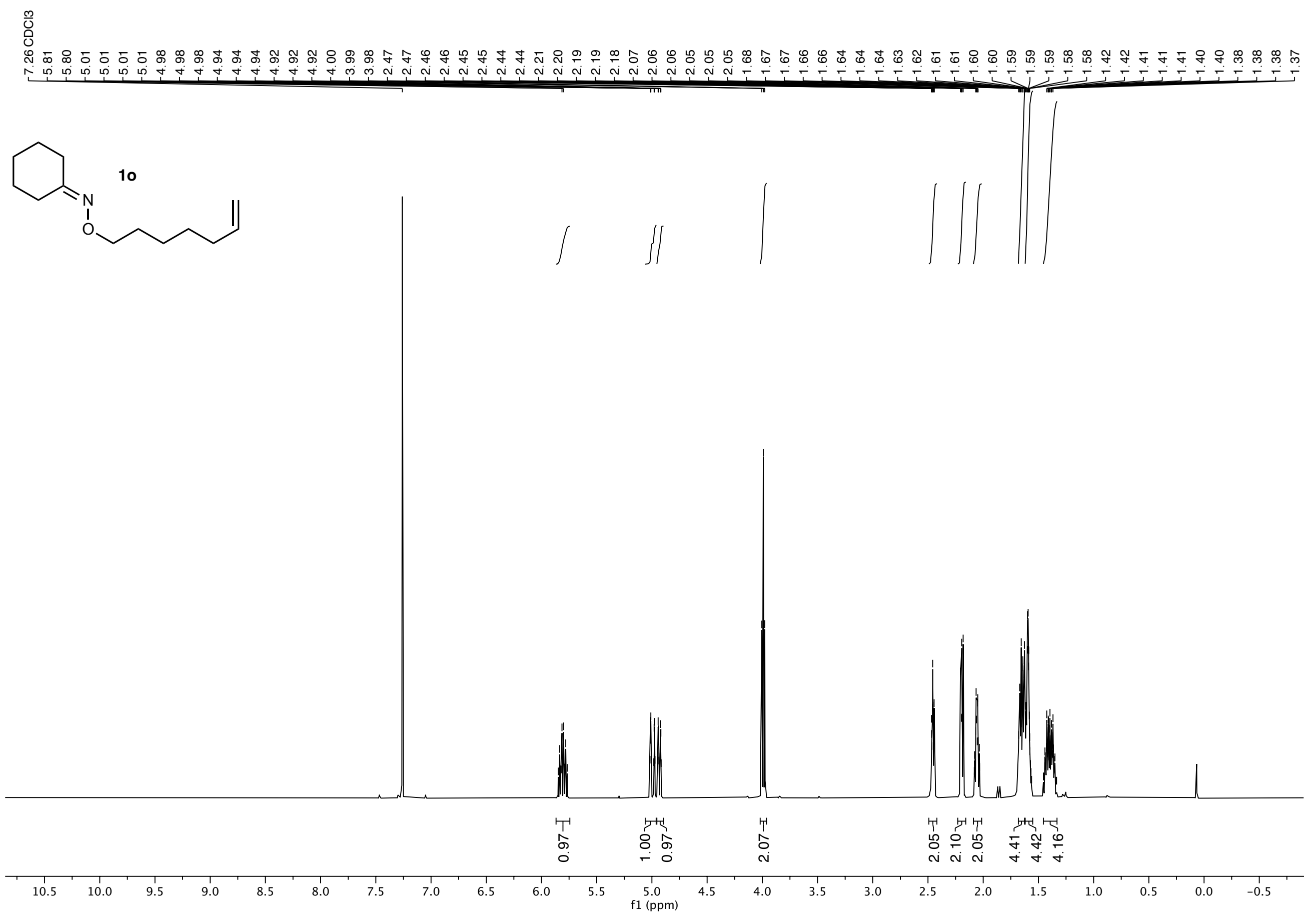
7.26 CDCl3
5.81
5.80
5.01
5.01
5.01
5.01
4.98
4.98
4.98
4.94
4.94
4.94
4.92
4.92
4.92
4.00
3.99
3.98
2.47
2.47
2.46
2.46
2.45
2.45
2.44
2.44
2.21
2.20
2.19
2.19
2.18
2.07
2.06
2.06
2.05
2.05
2.05
1.68
1.67
1.67
1.66
1.66
1.64
1.64
1.63
1.62
1.61
1.61
1.60
1.60
1.59
1.59
1.59
1.59
1.58
1.42
1.42
1.41
1.41
1.41
1.40
1.38
1.38
1.38
1.37
1o
N
O
0.97
1.00
0.97
2.07
2.05
2.10
2.05
4.41
4.42
4.16
10.5
10.0
9.5
9.0
8.5
8.0
7.5
7.0
6.5
6.0
5.5
5.0
4.5
4.0
3.5
3.0
2.5
2.0
1.5
1.0
0.5
0.0
-0.5
f1 (ppm)

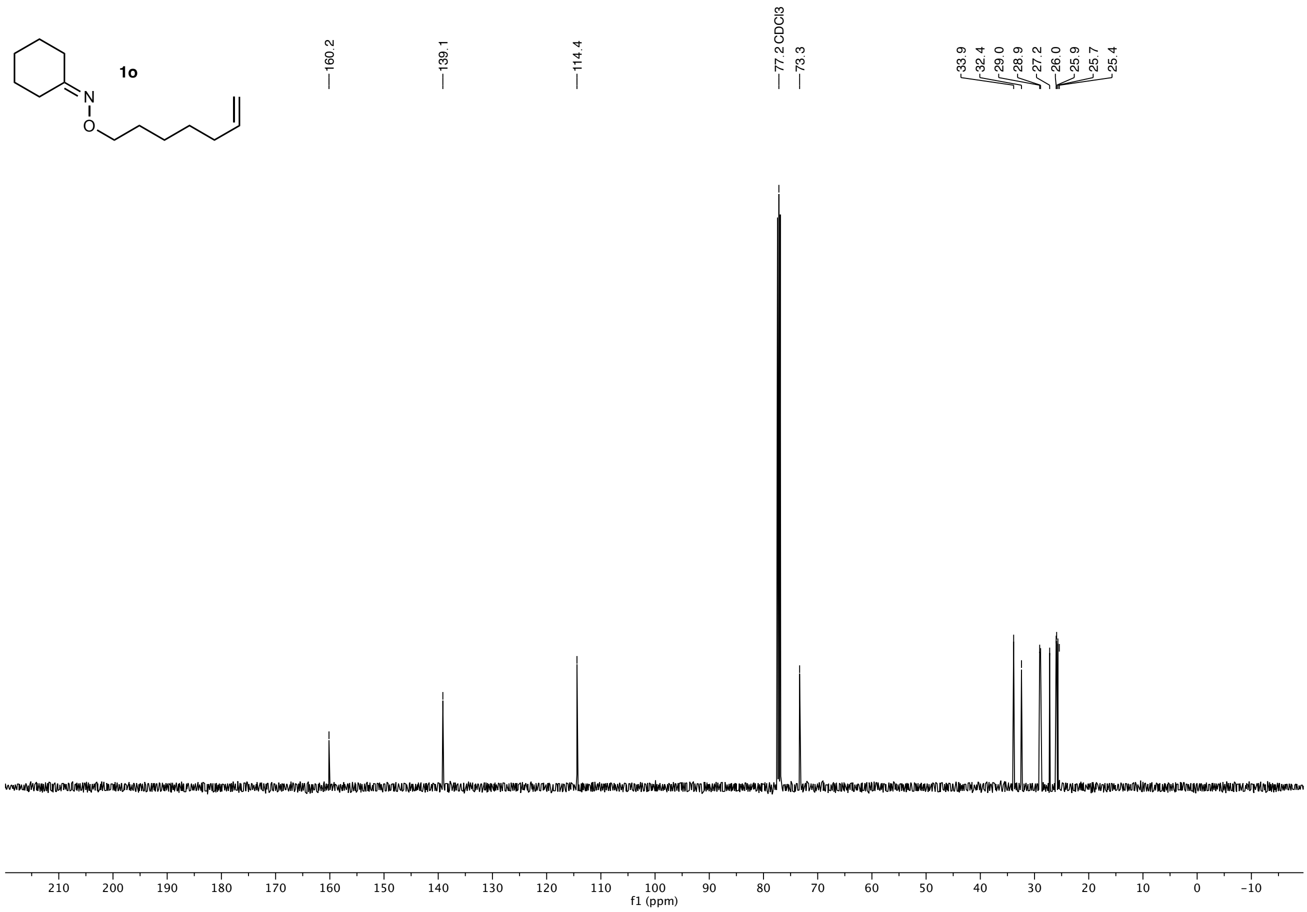
1o
N
O
160.2
139.1
114.4
77.2 CDCl3
73.3
33.9
32.4
29.0
28.9
27.2
26.0
25.9
25.7
25.4
210
200
190
180
170
160
150
140
130
120
110
100
90
80
70
60
50
40
30
20
10
0
-10
f1 (ppm)

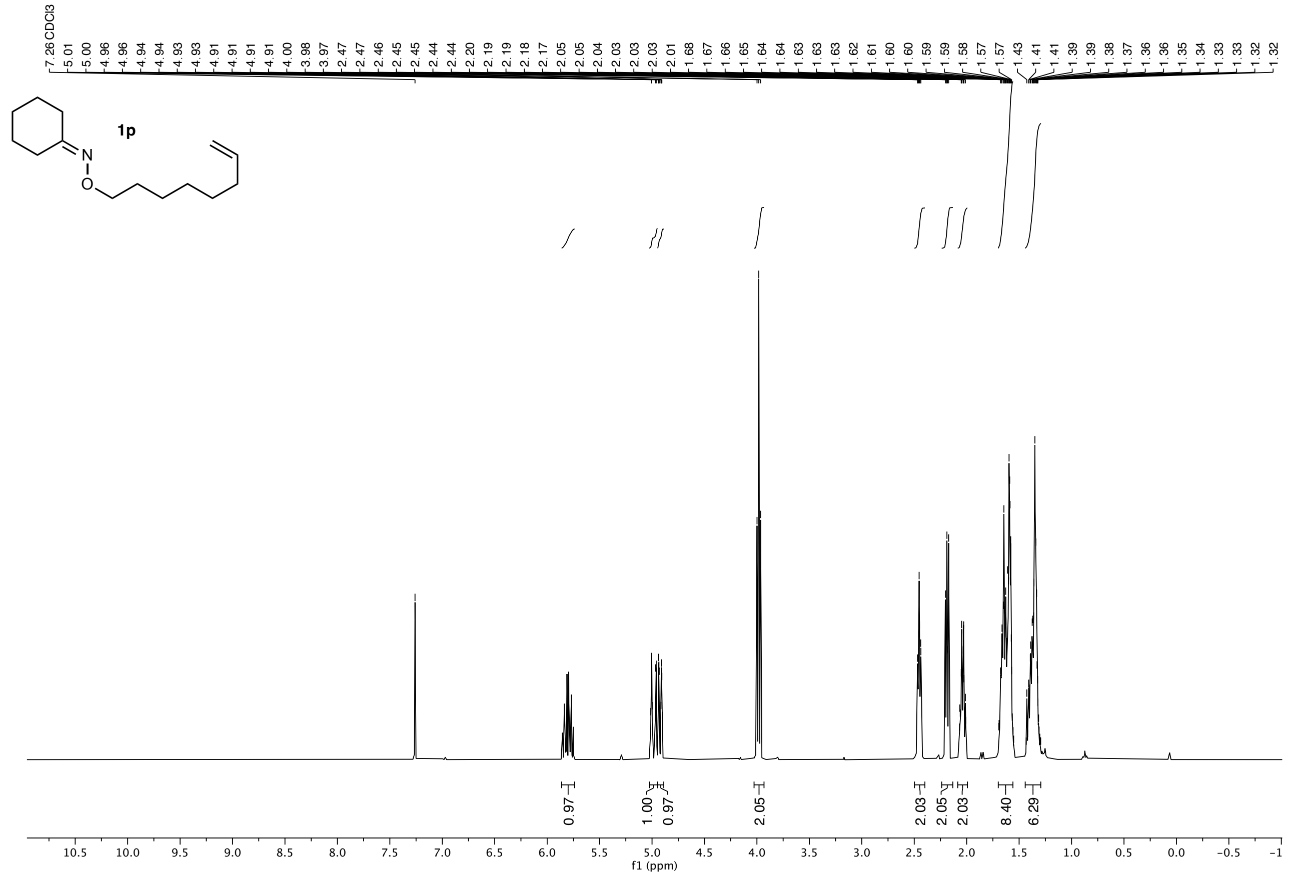
7.26 CDCl3
5.01 5.00 4.96 4.94 4.94 4.93 4.93 4.91 4.91 4.91 4.91 4.00 3.98 3.97 2.47 2.47 2.46 2.45 2.45 2.44 2.44 2.20 2.19 2.19 2.18 2.17 2.05 2.05 2.04 2.03 2.03 2.03 2.01 1.68 1.67 1.66 1.65 1.64 1.64 1.63 1.63 1.63 1.62 1.61 1.60 1.60 1.59 1.59 1.58 1.57 1.57 1.43 1.41 1.41 1.39 1.39 1.38 1.37 1.36 1.36 1.35 1.34 1.33 1.33 1.32 1.32
1p
N
O
0.97
1.00
0.97
2.05
2.03
2.05
2.03
8.40
6.29
10.5 10.0 9.5 9.0 8.5 8.0 7.5 7.0 6.5 6.0 5.5 5.0 4.5 4.0 3.5 3.0 2.5 2.0 1.5 1.0 0.5 0.0 −0.5 −1
f1 (ppm)

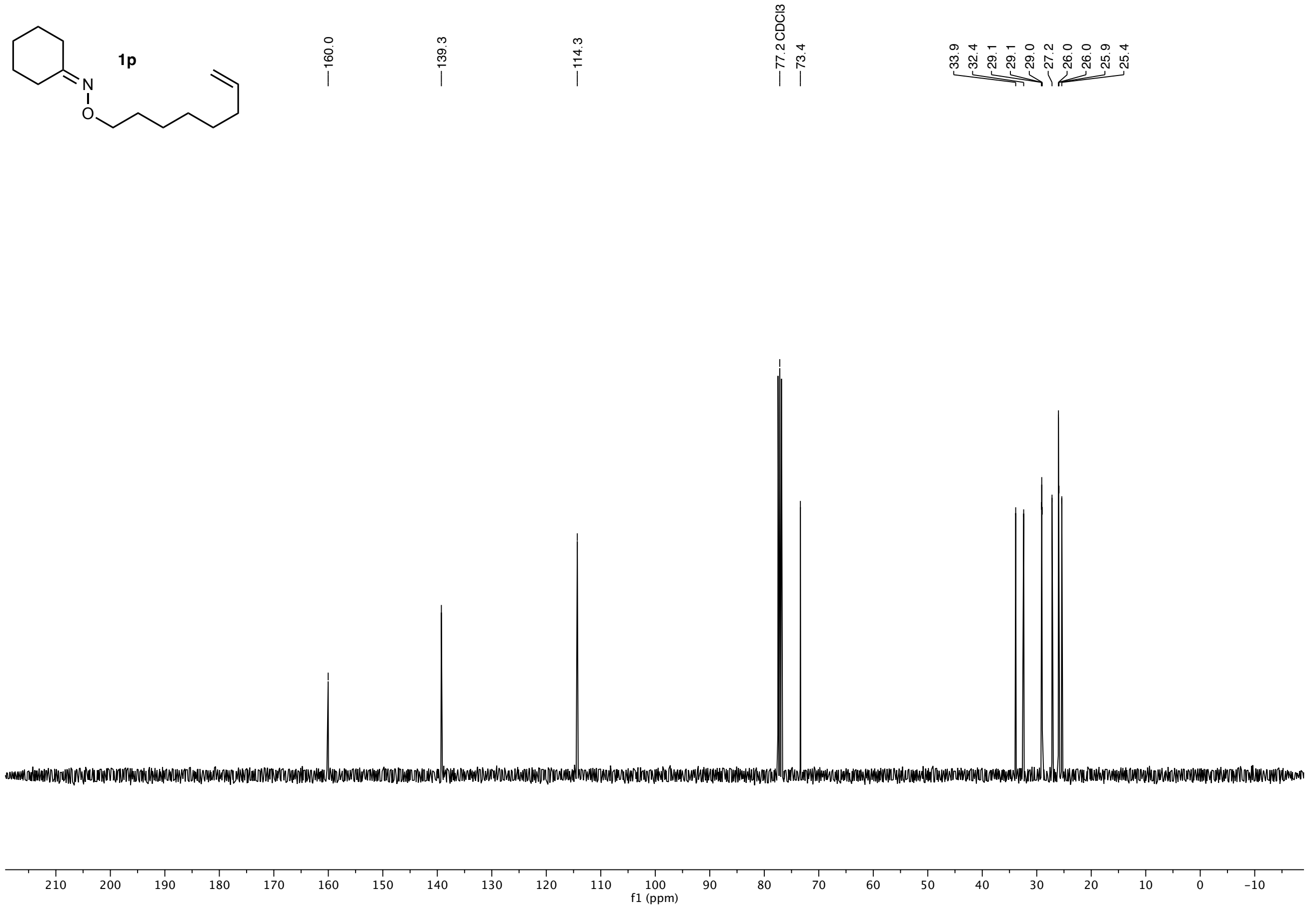
1p
N
O
160.0
139.3
114.3
77.2 CDCl3
73.4
33.9 32.4 29.1 29.1 29.0 27.2 26.0 26.0 25.9 25.4
210 200 190 180 170 160 150 140 130 120 110 100 90 80 70 60 50 40 30 20 10 0 −10
f1 (ppm)

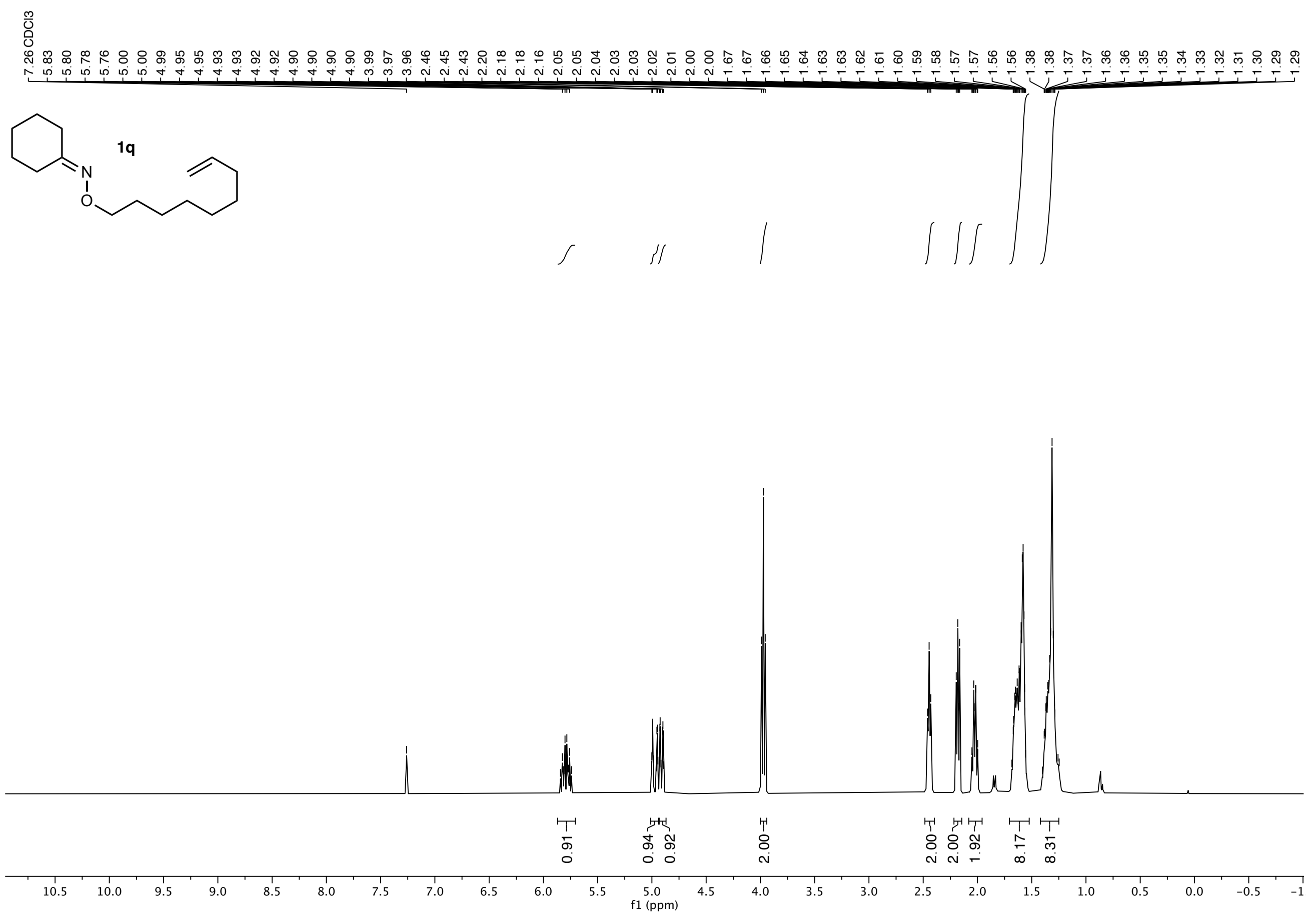
1q
f1 (ppm)

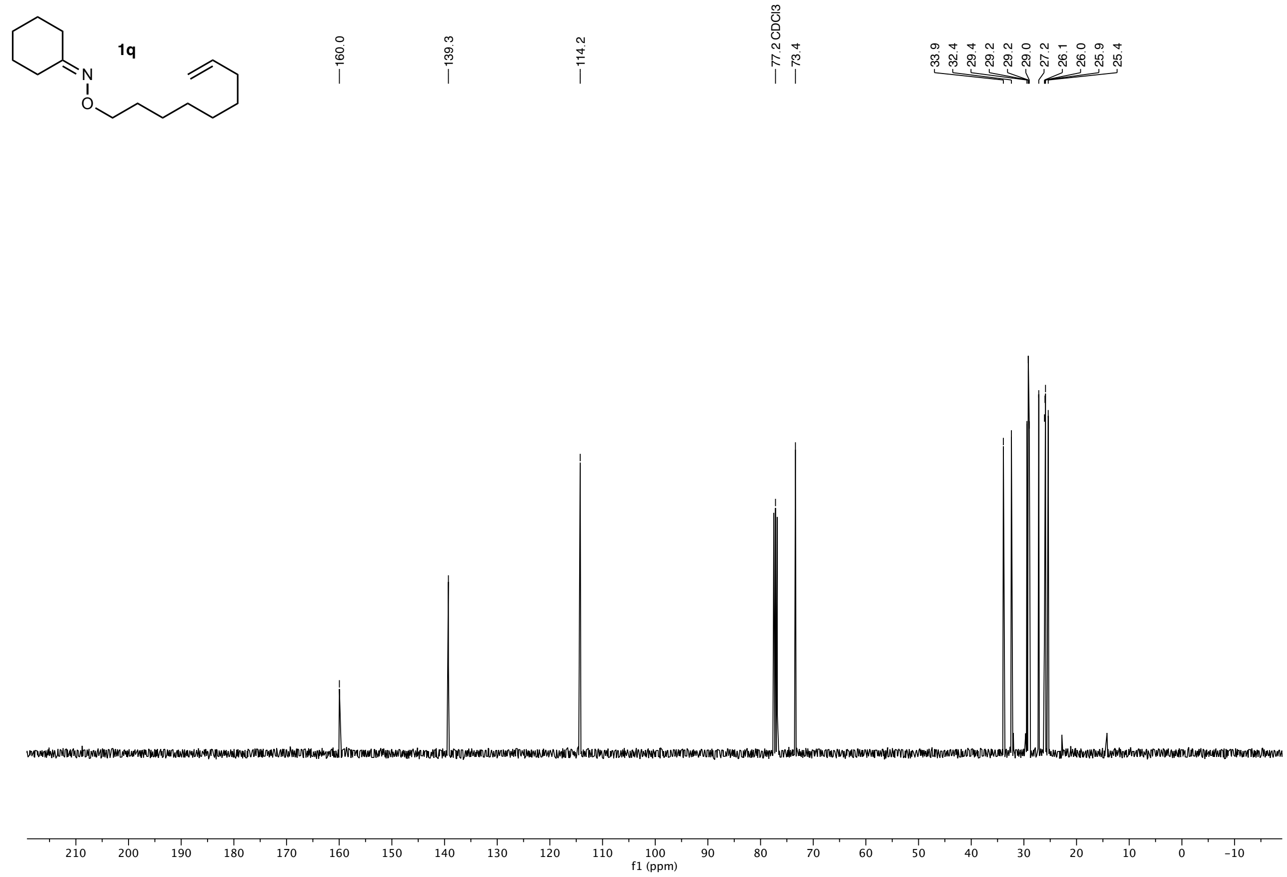
1q
f1 (ppm)

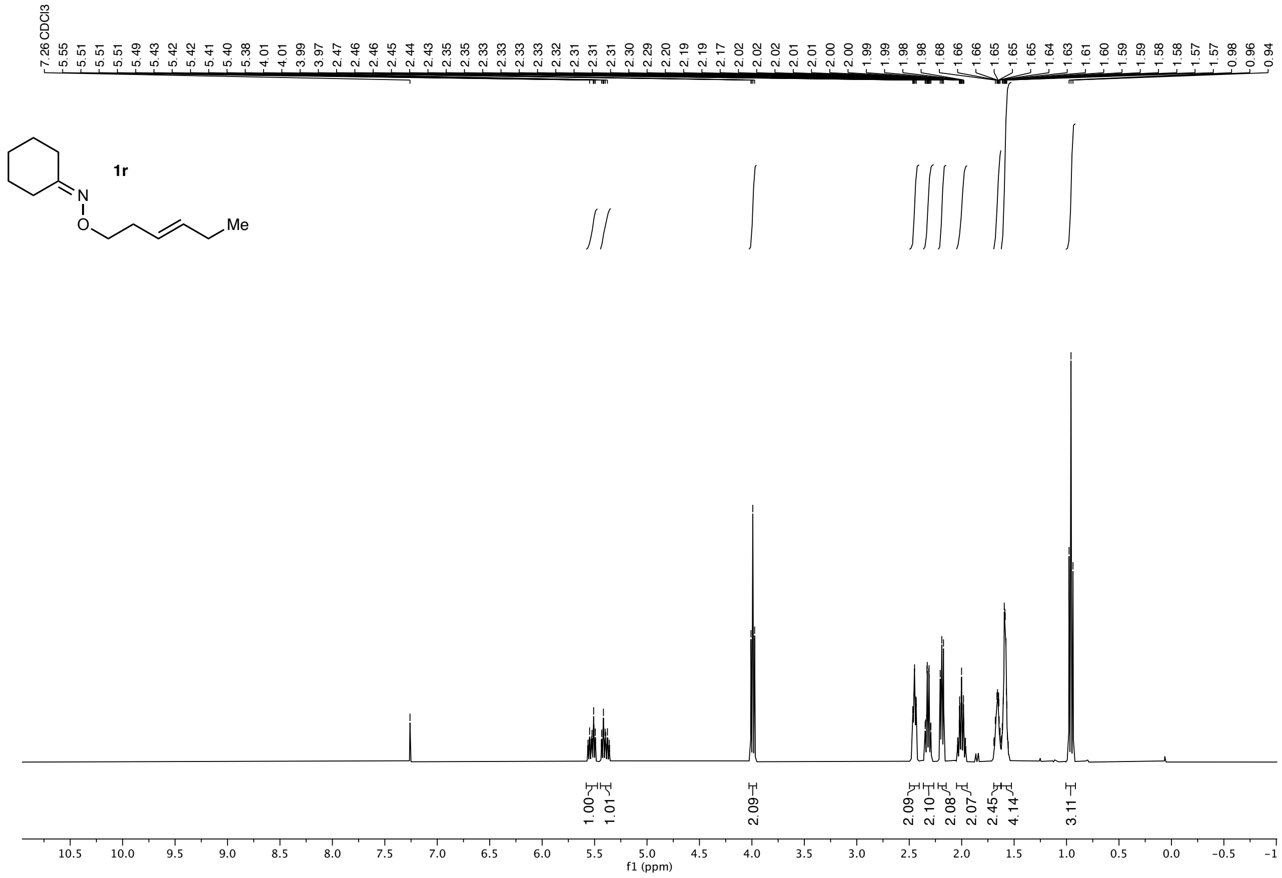
1r
N
O
Me
7.26 CDCl3
5.55
5.51
5.51
5.51
5.49
5.43
5.42
5.42
5.41
5.40
5.38
4.01
4.01
3.99
3.97
2.47
2.46
2.46
2.45
2.44
2.43
2.35
2.35
2.33
2.33
2.33
2.32
2.31
2.31
2.31
2.30
2.29
2.20
2.19
2.19
2.17
2.02
2.02
2.02
2.01
2.01
2.00
2.00
1.99
1.99
1.98
1.98
1.68
1.66
1.66
1.65
1.65
1.65
1.64
1.63
1.61
1.60
1.59
1.59
1.58
1.58
1.57
0.98
0.96
0.94
1.00
1.01
2.09
2.09
2.10
2.08
2.07
2.45
4.14
3.11
10.5
10.0
9.5
9.0
8.5
8.0
7.5
7.0
6.5
6.0
5.5
5.0
4.5
4.0
3.5
3.0
2.5
2.0
1.5
1.0
0.5
0.0
-0.5
-1
f1 (ppm)

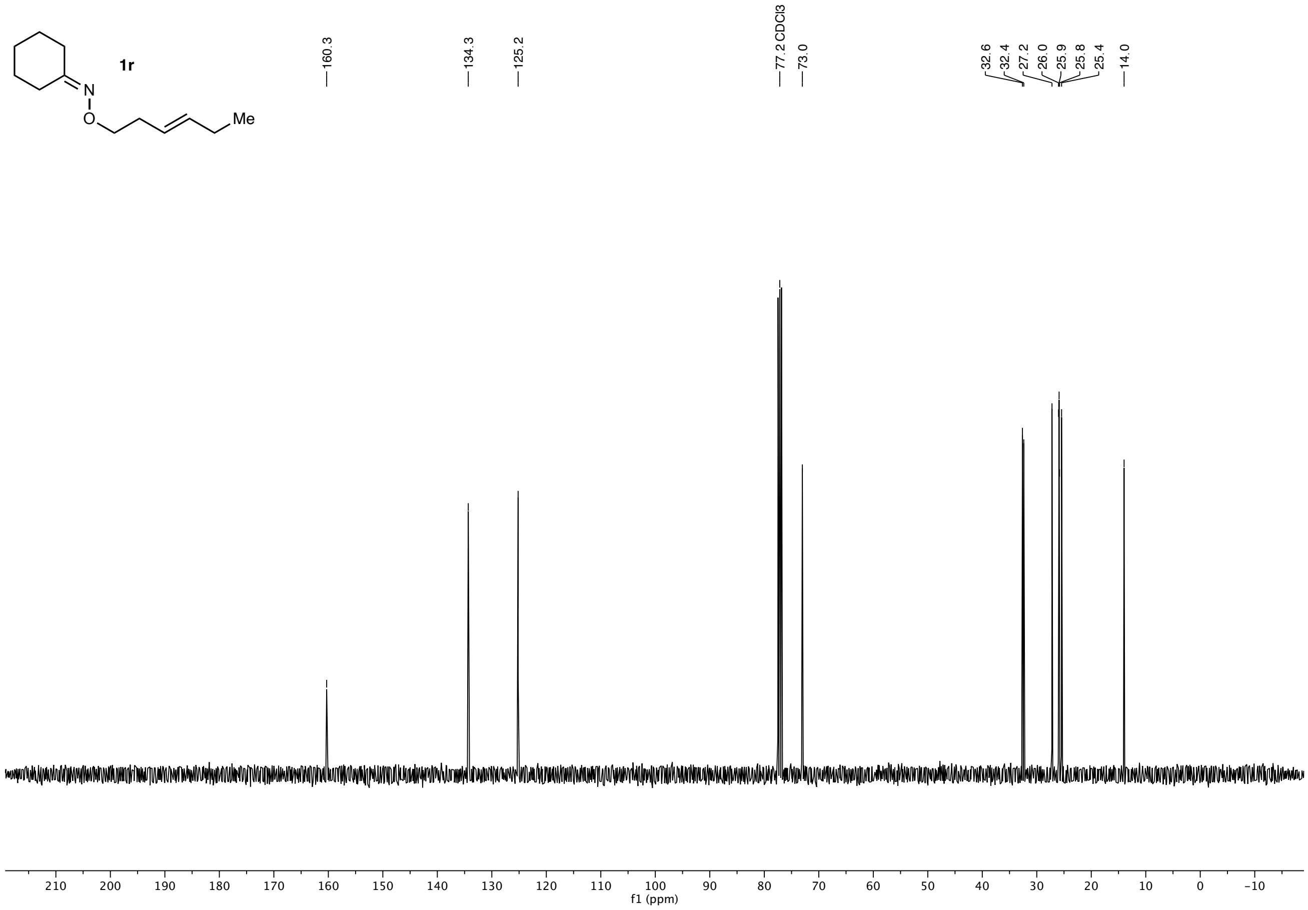
1r
N
O
Me
160.3
134.3
125.2
77.2 CDCl3
73.0
32.6
32.4
27.2
26.0
25.9
25.8
25.4
14.0
210
200
190
180
170
160
150
140
130
120
110
100
90
80
70
60
50
40
30
20
10
0
-10
f1 (ppm)

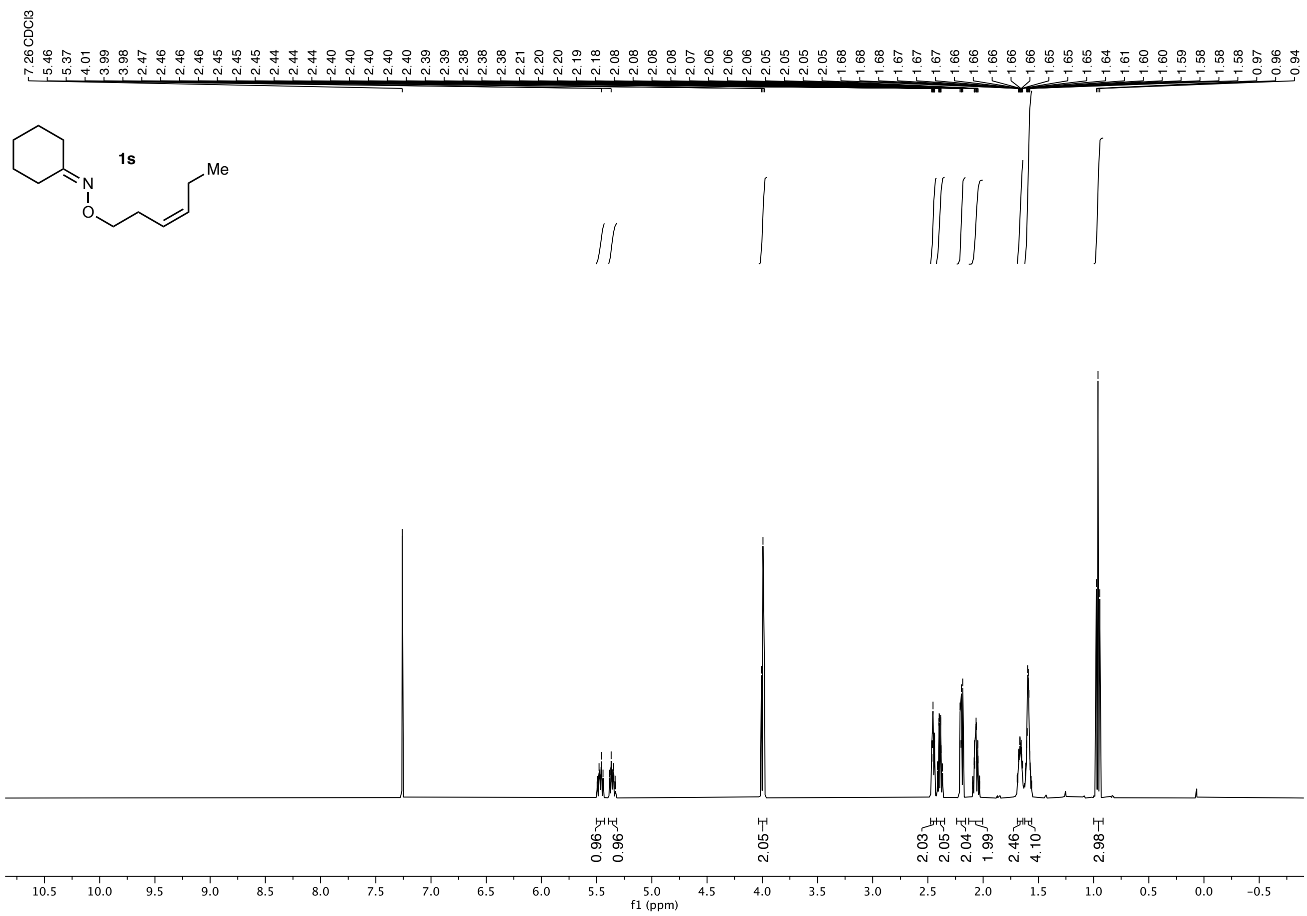
7.26 CDCl3
5.46
5.37
4.01
3.99
3.98
2.47
2.46
2.46
2.45
2.45
2.45
2.44
2.44
2.44
2.40
2.40
2.40
2.40
2.40
2.39
2.39
2.38
2.38
2.38
2.21
2.20
2.20
2.19
2.18
2.08
2.08
2.08
2.08
2.07
2.06
2.06
2.06
2.05
2.05
2.05
2.05
1.68
1.68
1.68
1.67
1.67
1.67
1.66
1.66
1.66
1.66
1.66
1.65
1.65
1.65
1.64
1.61
1.60
1.60
1.59
1.58
1.58
1.58
0.97
0.96
0.94
1s
N
O
Me
0.96
0.96
2.05
2.03
2.05
2.04
1.99
2.46
4.10
2.98
f1 (ppm)

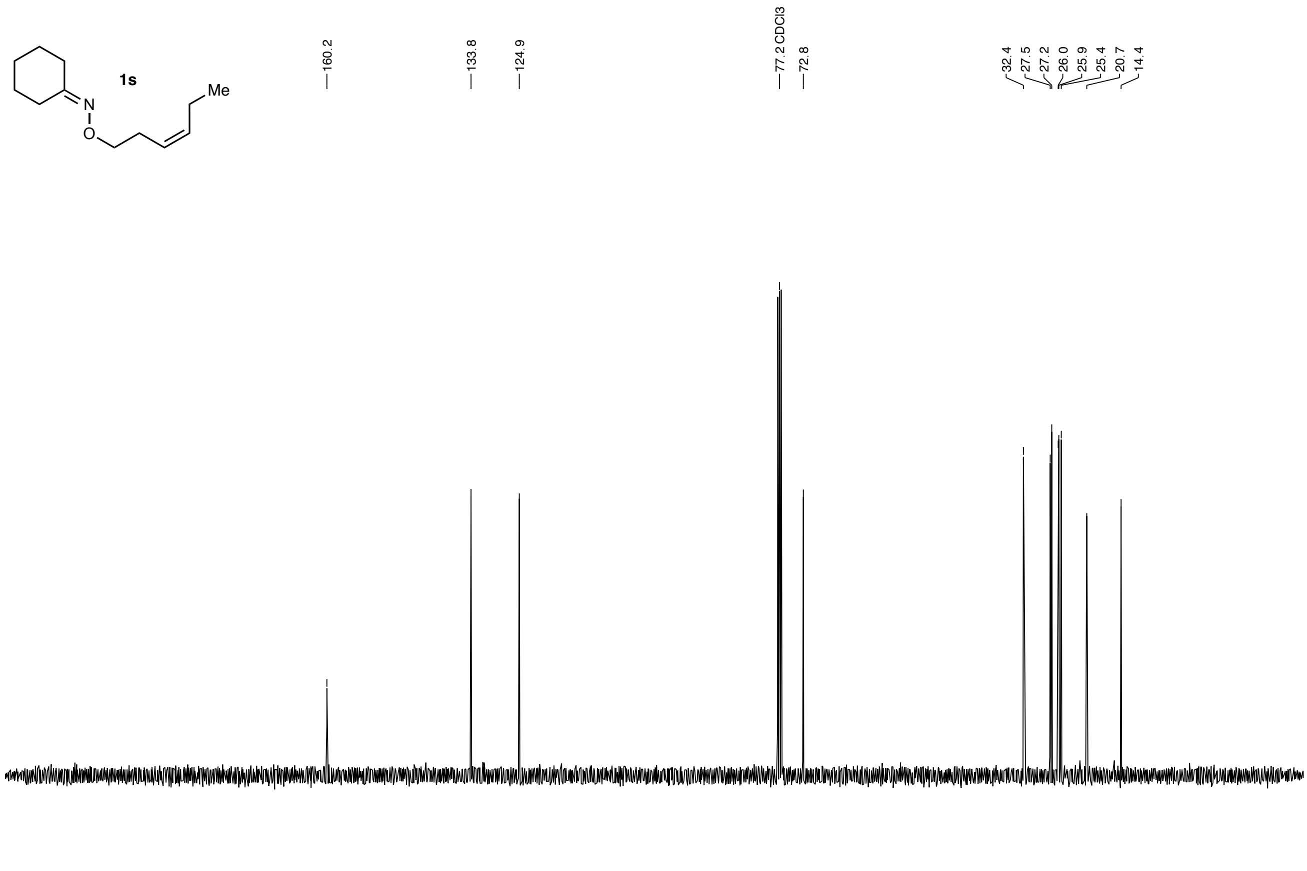
160.2
133.8
124.9
77.2 CDCl3
72.8
32.4
27.5
27.2
26.0
25.9
25.4
20.7
14.4
1s
N
O
Me
f1 (ppm)

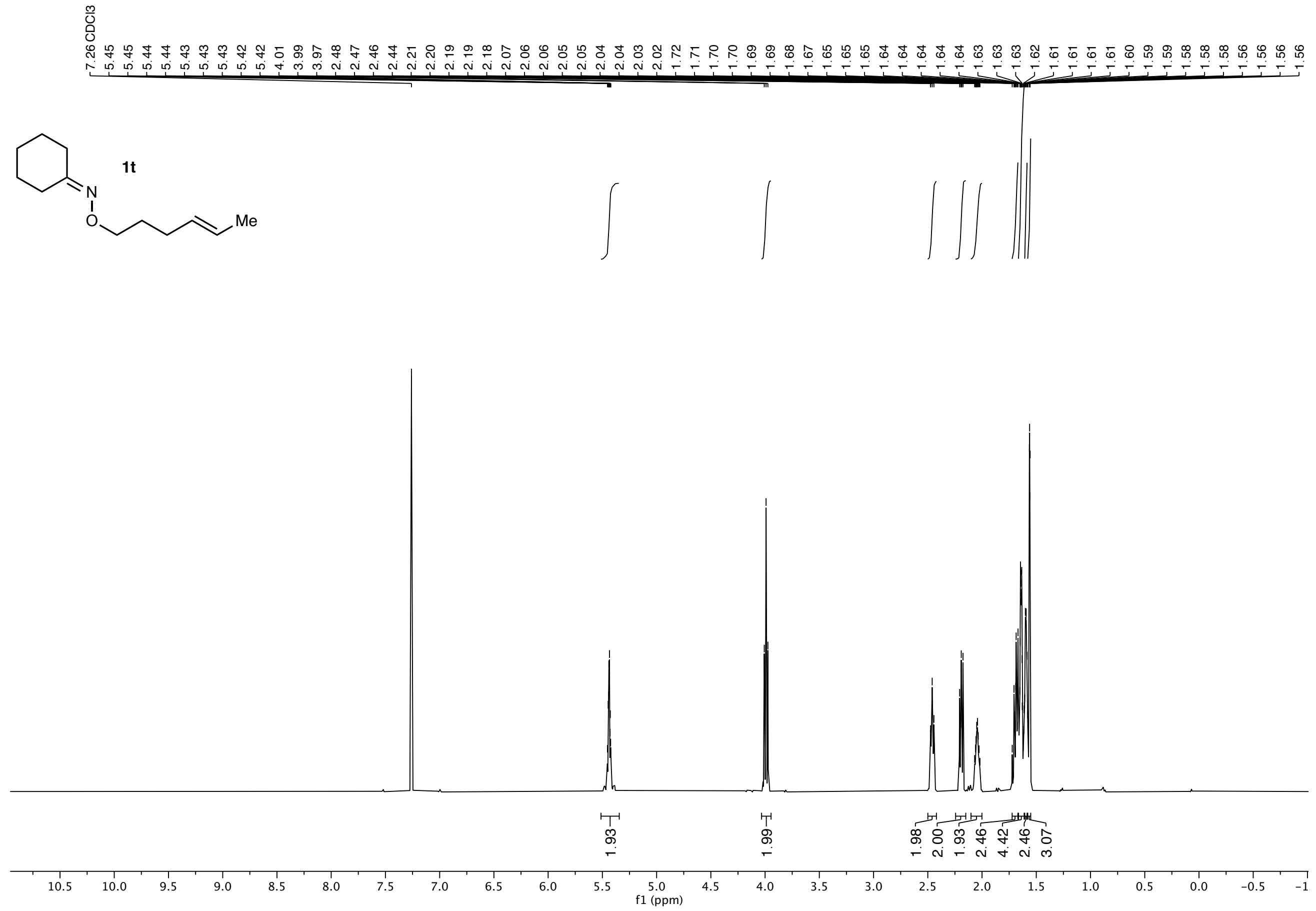
7.26 CDCl3
5.45 5.45 5.44 5.44 5.43 5.43 5.42 5.42 4.01 3.99 3.97 2.48 2.47 2.46 2.44 2.21 2.20 2.19 2.19 2.18 2.07 2.06 2.06 2.05 2.05 2.04 2.04 2.03 2.02 1.72 1.71 1.70 1.69 1.69 1.68 1.67 1.65 1.65 1.64 1.64 1.64 1.64 1.63 1.63 1.63 1.62 1.61 1.61 1.61 1.61 1.60 1.59 1.59 1.58 1.58 1.56 1.56 1.56 1.56
1t
N
O
Me
1.93
1.99
1.98
2.00
1.93
2.46
4.42
2.46
3.07
10.5 10.0 9.5 9.0 8.5 8.0 7.5 7.0 6.5 6.0 5.5 5.0 4.5 4.0 3.5 3.0 2.5 2.0 1.5 1.0 0.5 0.0 −0.5 −1
f1 (ppm)

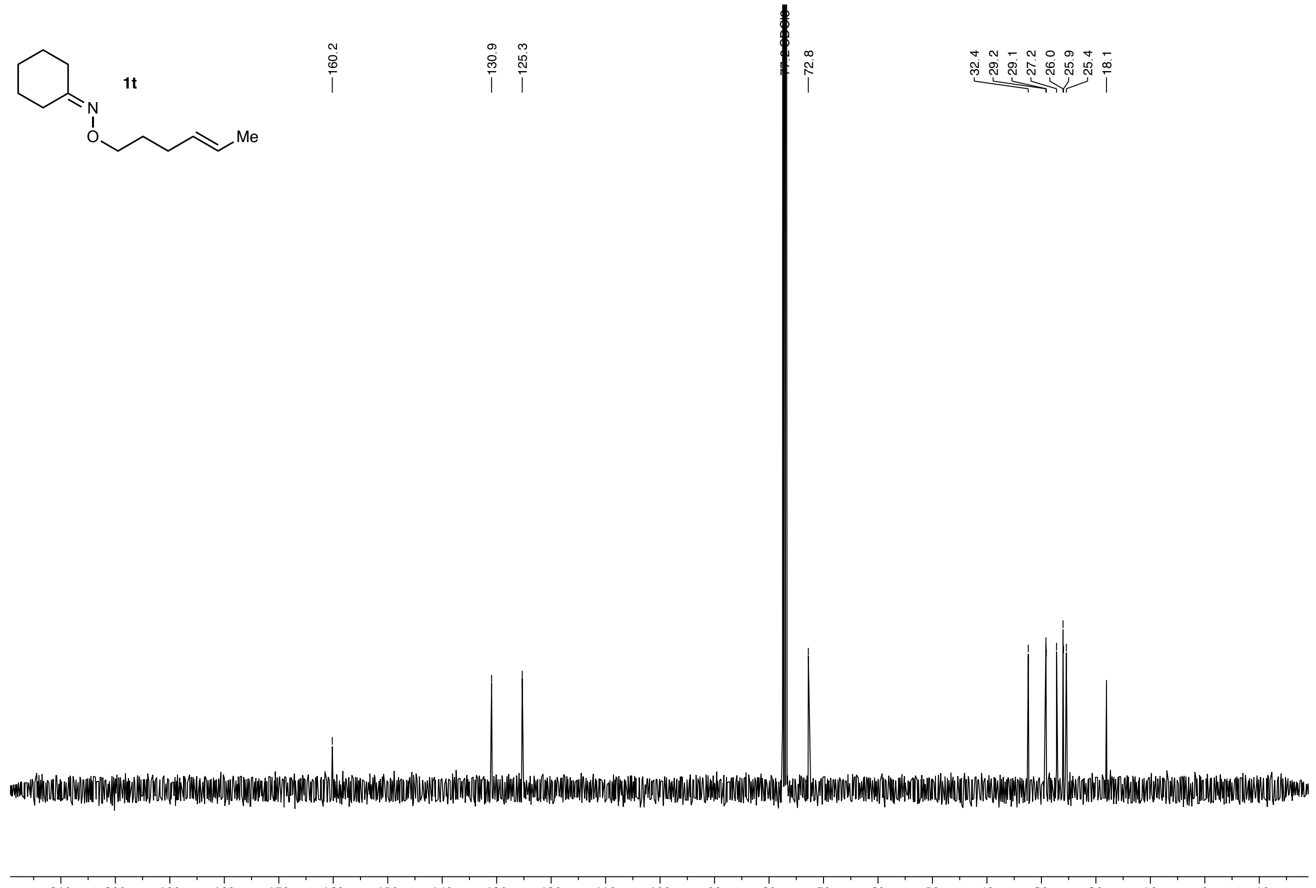
1t
N
O
Me
160.2
130.9
125.3
72.8
32.4
29.2
29.1
27.2
26.0
25.9
25.4
18.1
210 200 190 180 170 160 150 140 130 120 110 100 90 80 70 60 50 40 30 20 10 0 −10
f1 (ppm)

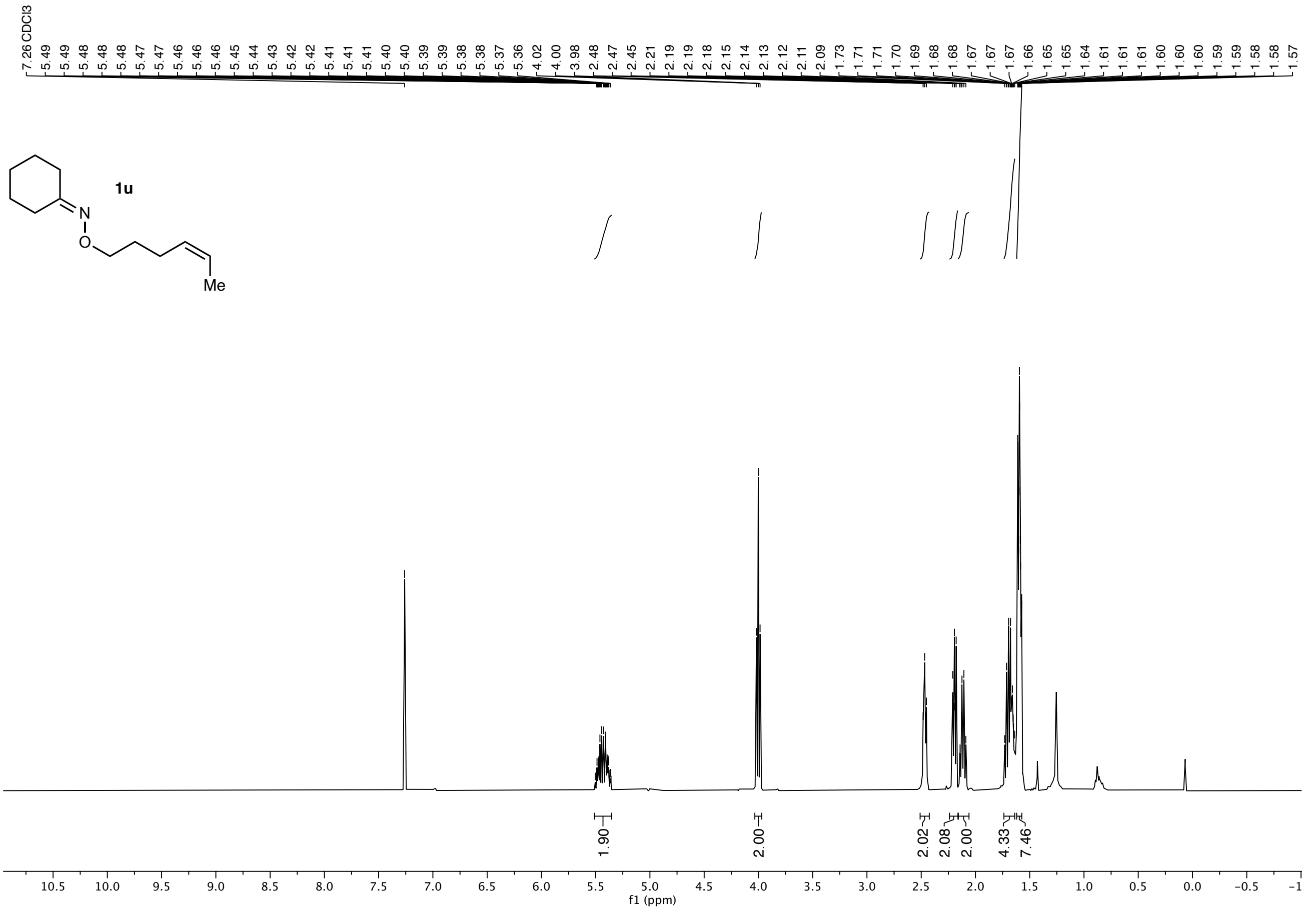
7.26 CDCl3
5.49 5.49 5.48 5.48 5.47 5.47 5.46 5.46 5.45 5.44 5.43 5.42 5.41 5.41 5.41 5.40 5.40 5.39 5.39 5.38 5.38 5.37 5.36 4.02 4.00 3.98 2.48 2.47 2.45 2.21 2.19 2.19 2.18 2.15 2.14 2.13 2.12 2.11 2.09 1.73 1.71 1.71 1.70 1.69 1.68 1.68 1.67 1.67 1.67 1.66 1.65 1.64 1.61 1.61 1.61 1.60 1.60 1.60 1.59 1.59 1.58 1.58 1.57
1u
N
O
Me
1.90
2.00
2.02
2.08
2.00
4.33
7.46
f1 (ppm)

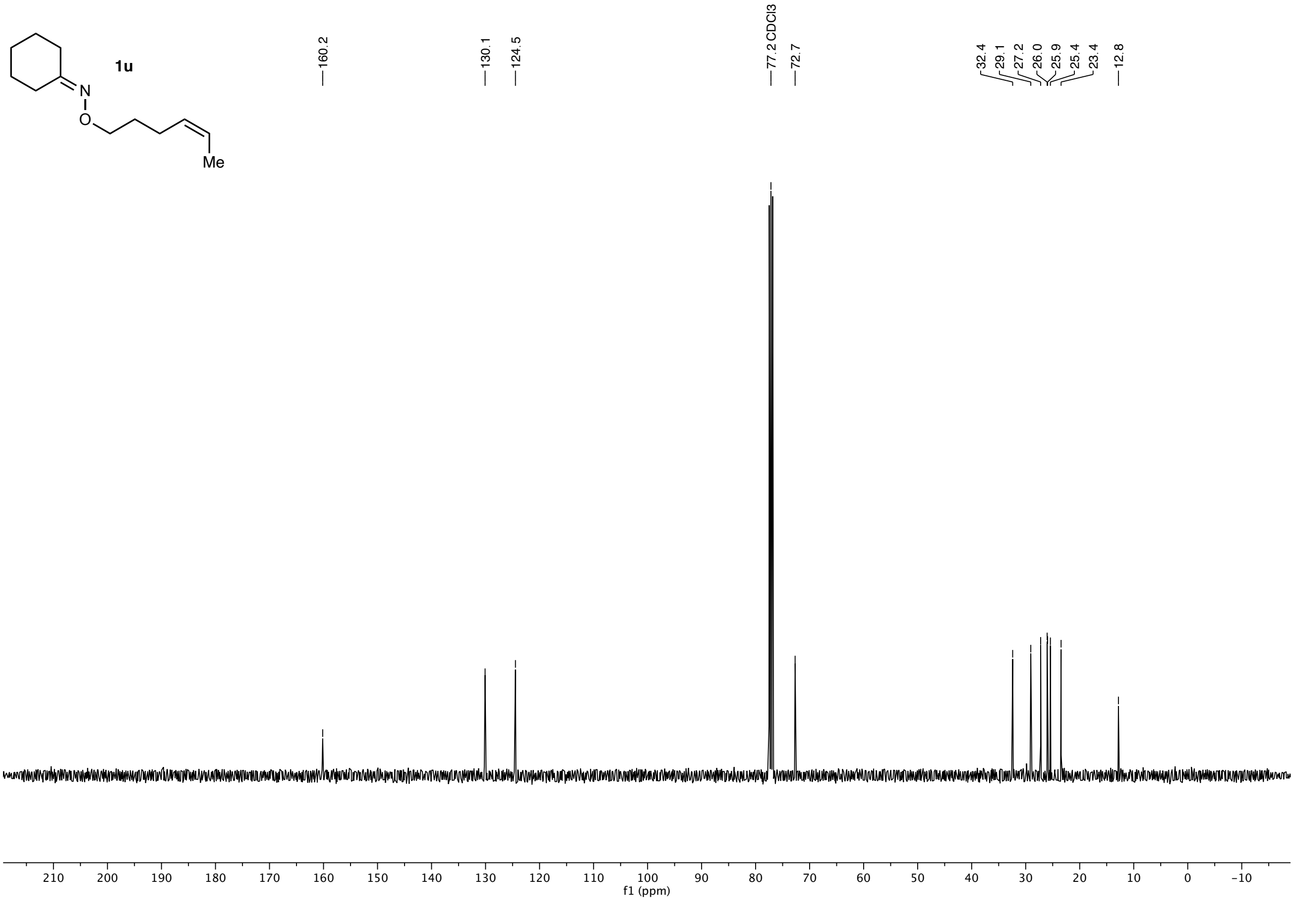
1u
N
O
Me
160.2
130.1
124.5
77.2 CDCl3
72.7
32.4
29.1
27.2
26.0
25.9
25.4
23.4
12.8
f1 (ppm)

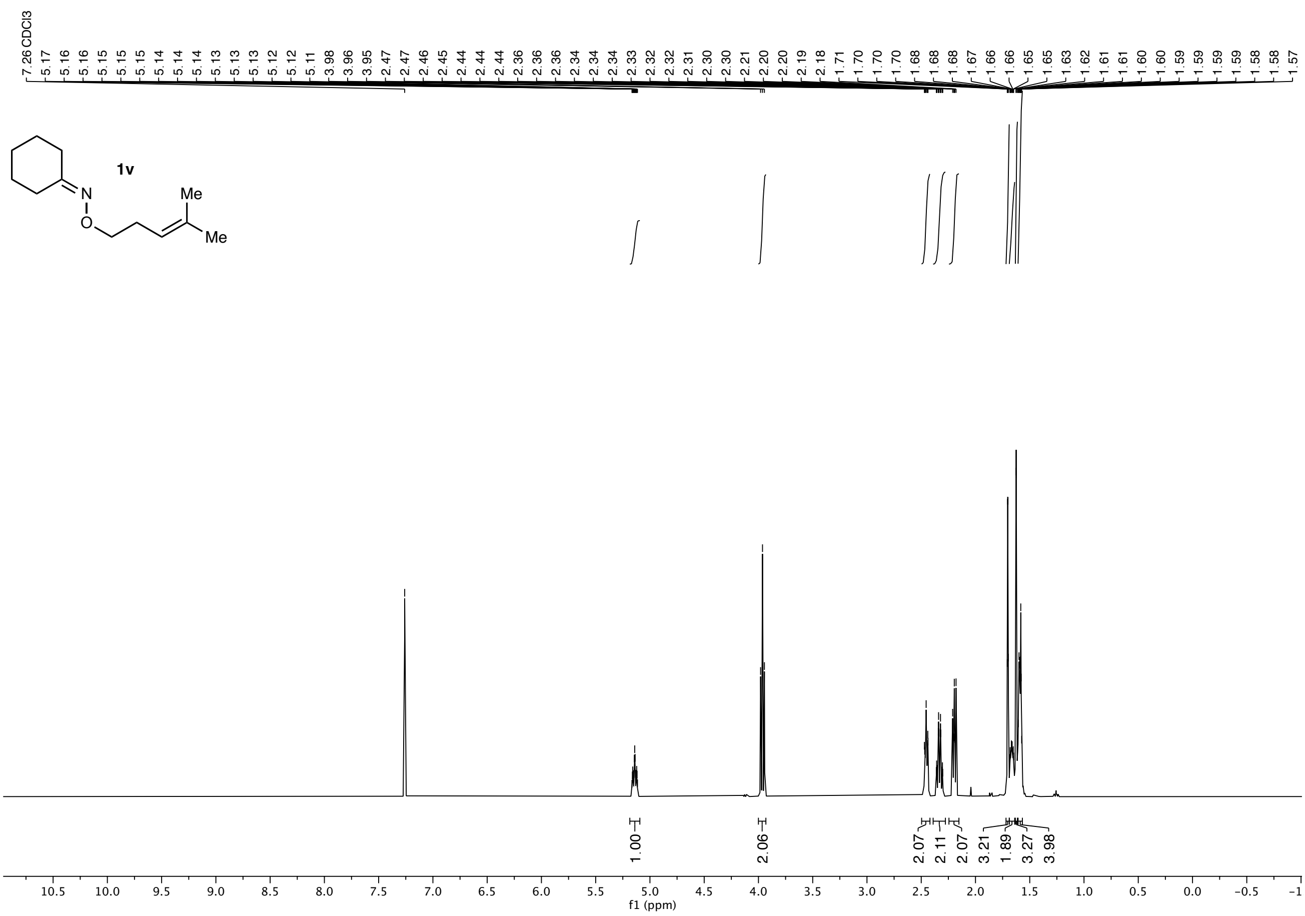
7.26 CDCl3
5.17 5.16 5.16 5.15 5.15 5.15 5.14 5.14 5.13 5.13 5.13 5.12 5.11 3.98 3.96 3.95 2.47 2.47 2.46 2.45 2.44 2.44 2.44 2.36 2.36 2.36 2.34 2.34 2.34 2.33 2.32 2.32 2.31 2.30 2.30 2.21 2.20 2.20 2.19 2.18 1.71 1.70 1.70 1.68 1.68 1.68 1.67 1.66 1.66 1.65 1.65 1.63 1.62 1.61 1.61 1.60 1.60 1.59 1.59 1.59 1.59 1.58 1.58 1.57
1v
N
O
Me
Me
1.00
2.06
2.07
2.11
2.07
3.21
1.89
3.27
3.98
f1 (ppm)

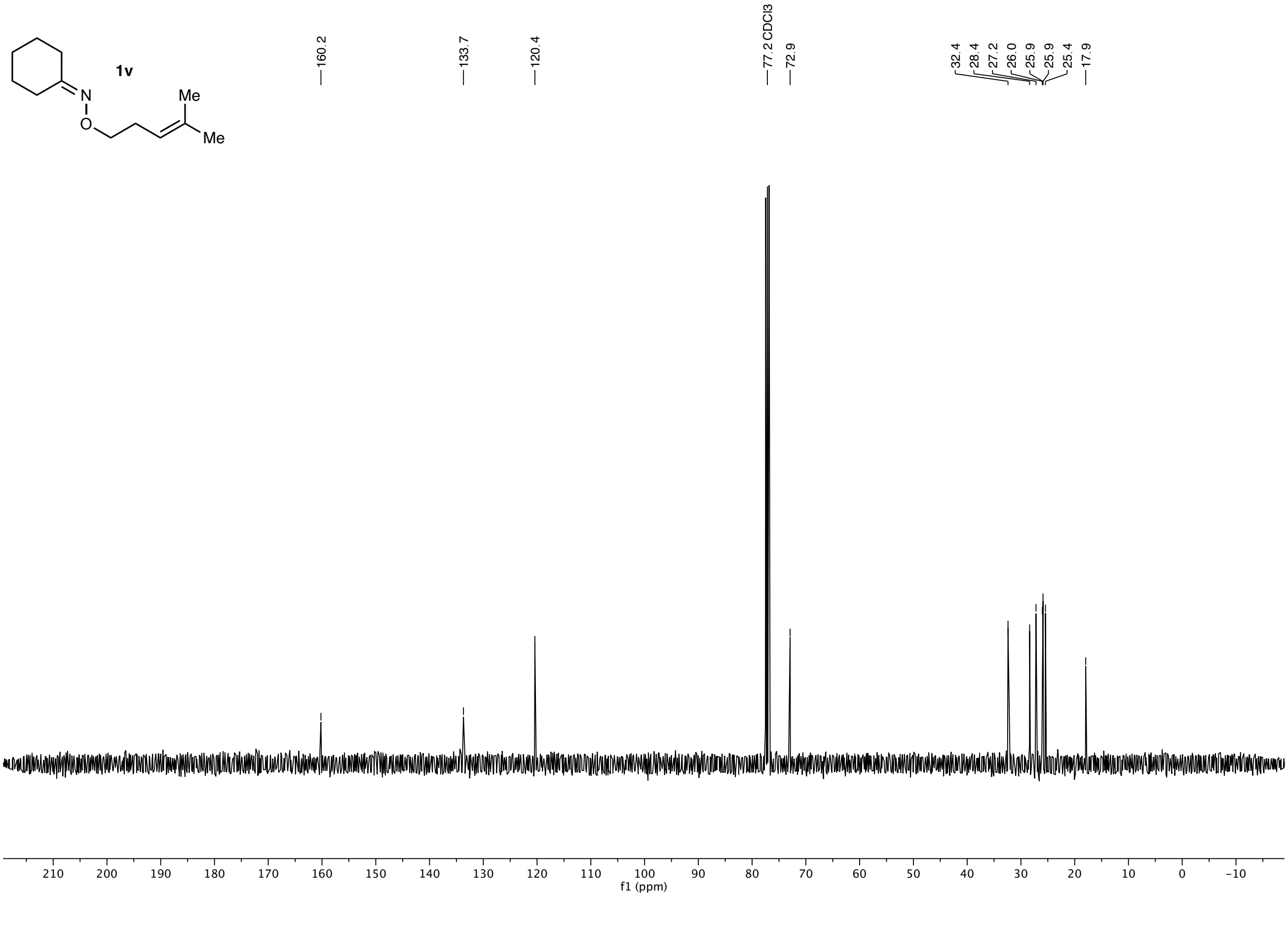
160.2
133.7
120.4
77.2 CDCl3
72.9
32.4 28.4 27.2 26.0 25.9 25.9 25.4
17.9
1v
N
O
Me
Me
f1 (ppm)

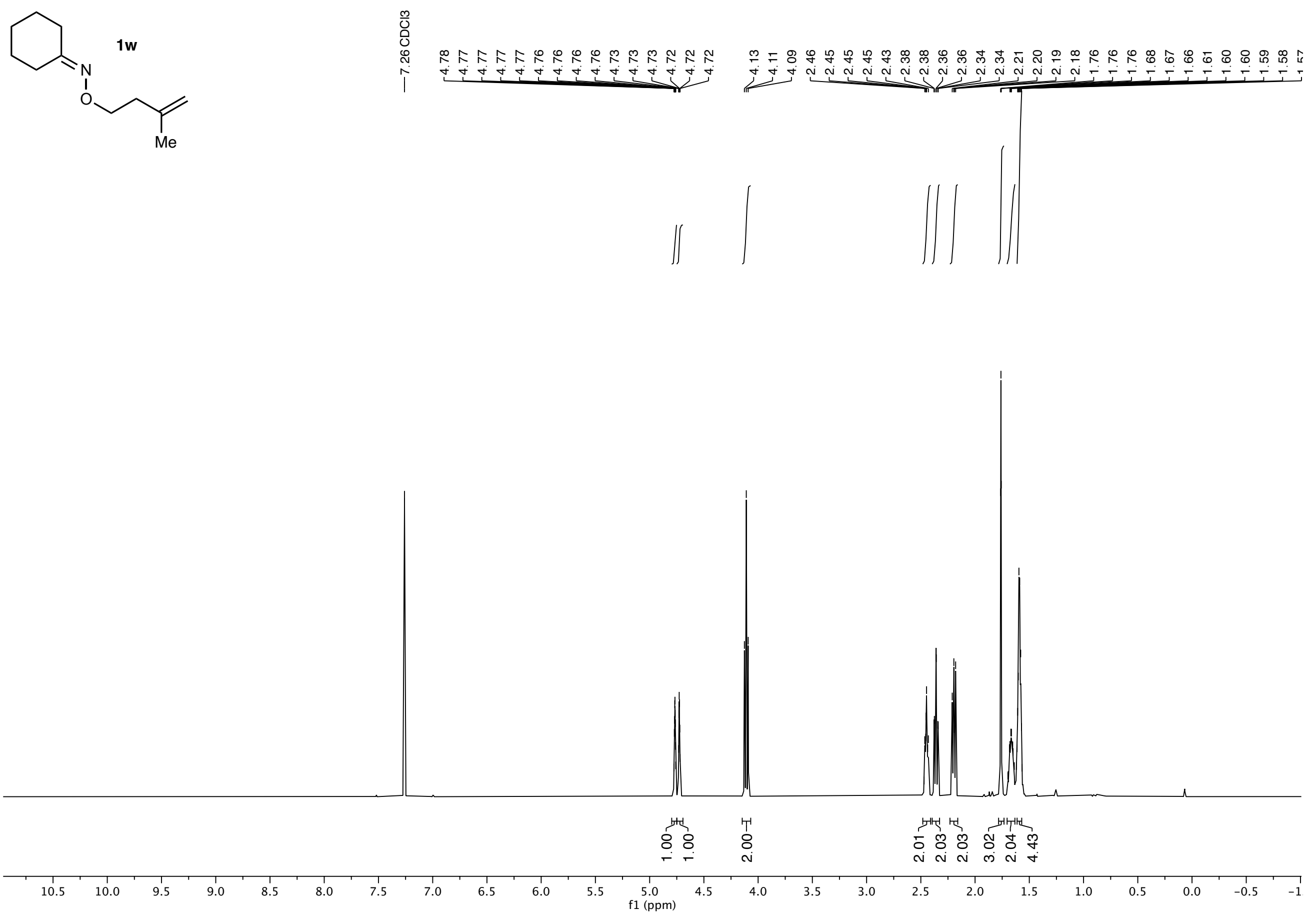
1w
N
O
Me
7.26 CDCl3
4.78
4.77
4.77
4.77
4.77
4.76
4.76
4.76
4.73
4.73
4.72
4.72
4.72
4.72
4.13
4.11
4.09
2.46
2.45
2.45
2.45
2.43
2.38
2.38
2.36
2.36
2.34
2.34
2.21
2.20
2.19
2.18
1.76
1.76
1.76
1.68
1.67
1.66
1.61
1.60
1.60
1.59
1.58
1.00
1.00
2.00
2.01
2.03
2.03
3.02
2.04
4.43
f1 (ppm)

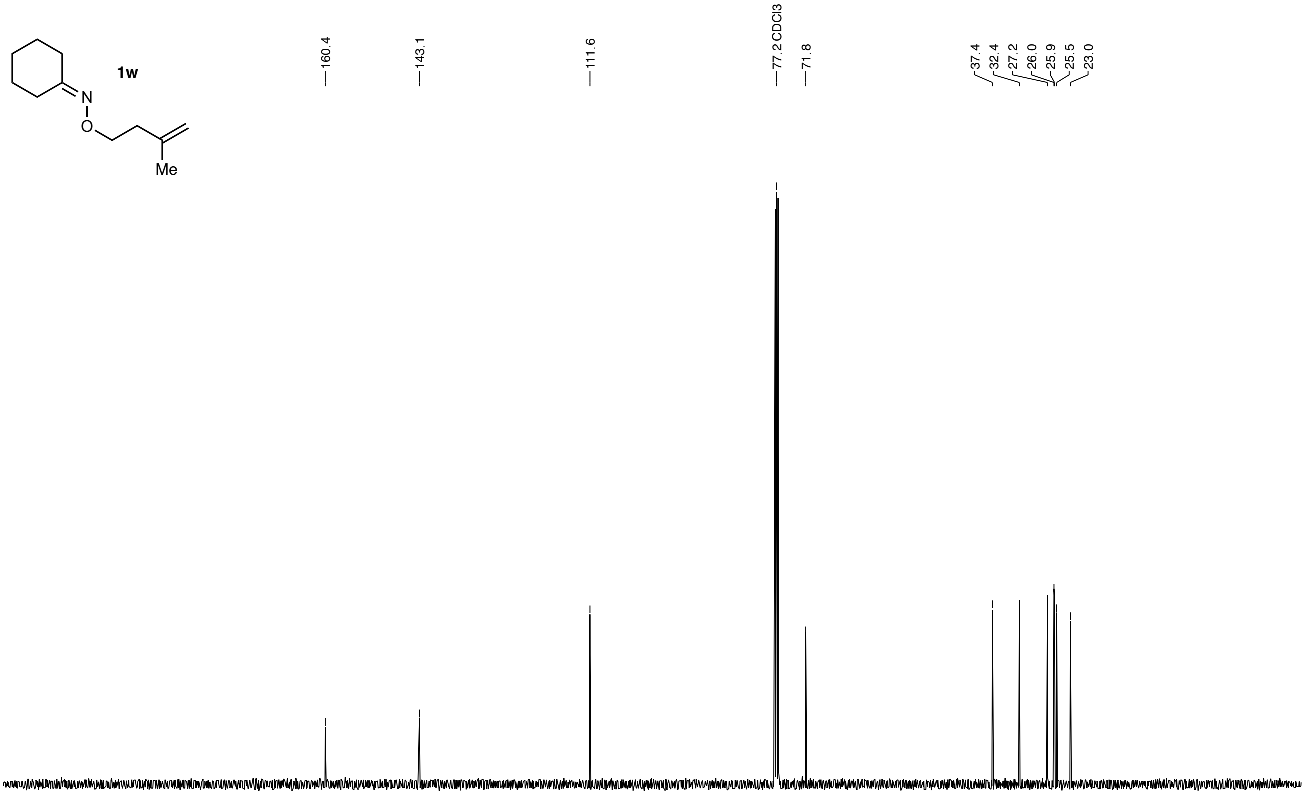
1w
N
O
Me
160.4
143.1
111.6
77.2 CDCl3
71.8
37.4
32.4
27.2
26.0
25.9
25.5
23.0
f1 (ppm)

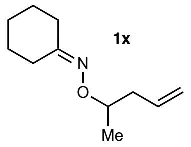

1x

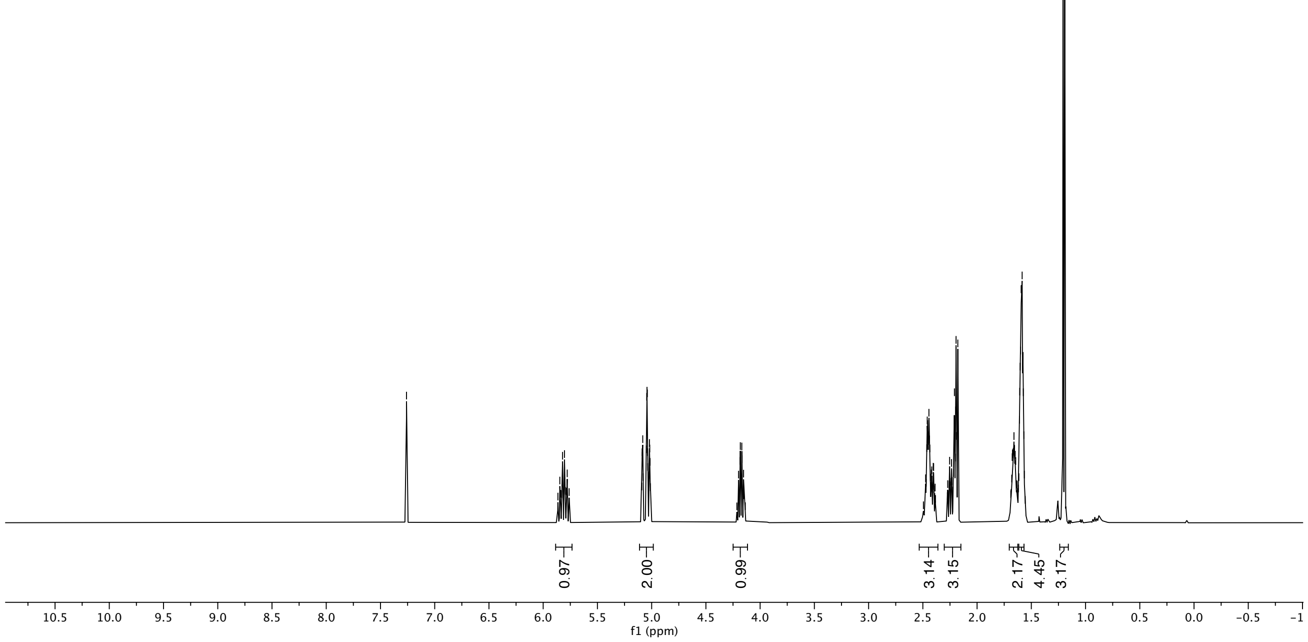
7.26 CDCl3
5.82
5.80
5.78
5.09
5.09
5.08
5.08
5.08
5.05
5.05
5.04
5.04
5.04
5.04
5.02
5.02
5.02
5.01
4.20
4.19
4.18
4.17
4.17
4.15
2.46
2.46
2.46
2.45
2.45
2.44
2.44
2.44
2.42
2.42
2.40
2.40
2.25
2.24
2.22
2.21
2.21
2.20
2.20
2.19
2.19
2.18
1.68
1.67
1.67
1.67
1.66
1.65
1.65
1.65
1.64
1.61
1.61
1.60
1.59
1.59
1.58
1.58
1.57
1.57
1.56
1.21
1.19
0.97
2.00
0.99
3.14
3.15
2.17
4.45
3.17
10.5 10.0 9.5 9.0 8.5 8.0 7.5 7.0 6.5 6.0 5.5 5.0 4.5 4.0 3.5 3.0 2.5 2.0 1.5 1.0 0.5 0.0 −0.5 −1
f1 (ppm)

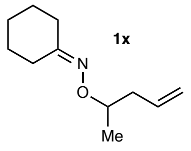

1x

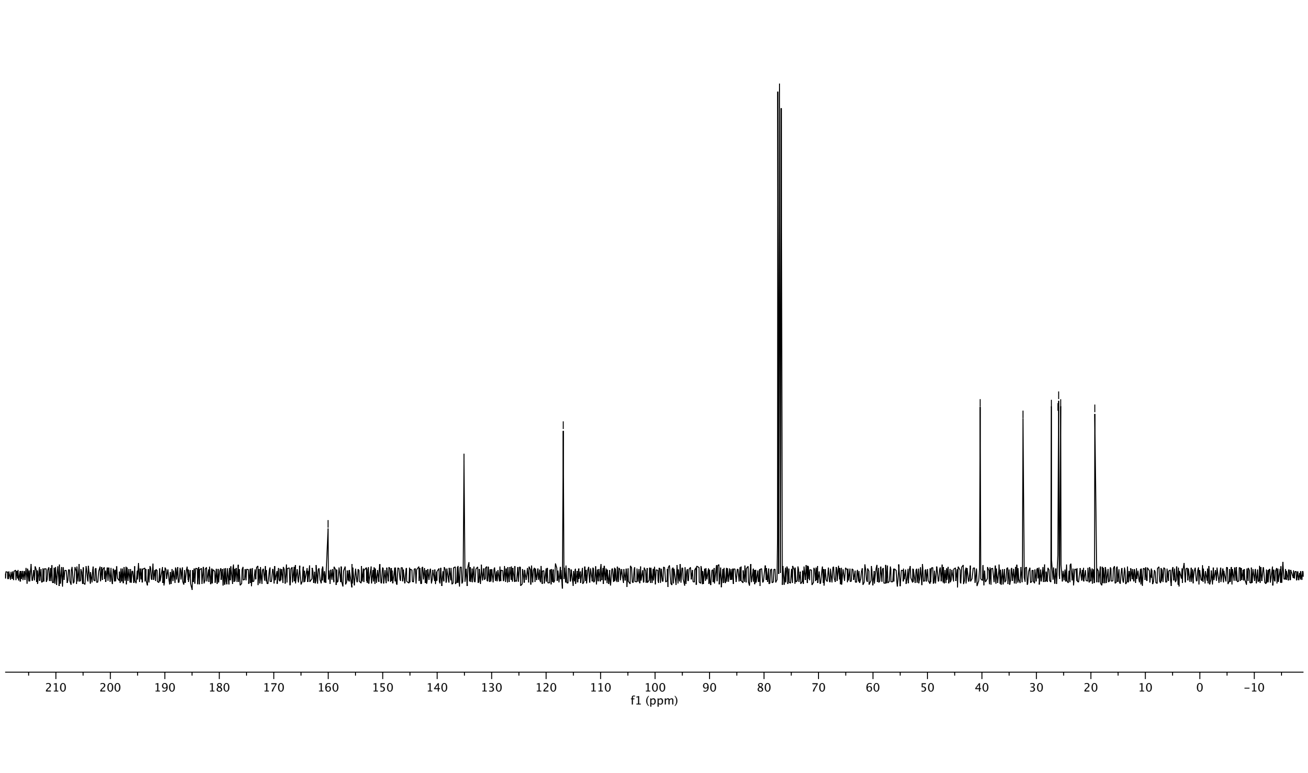
160.0
135.1
116.9
77.4
77.2 CDCl3
40.3
32.5
27.3
26.0
25.9
25.5
19.3
210 200 190 180 170 160 150 140 130 120 110 100 90 80 70 60 50 40 30 20 10 0 −10
f1 (ppm)

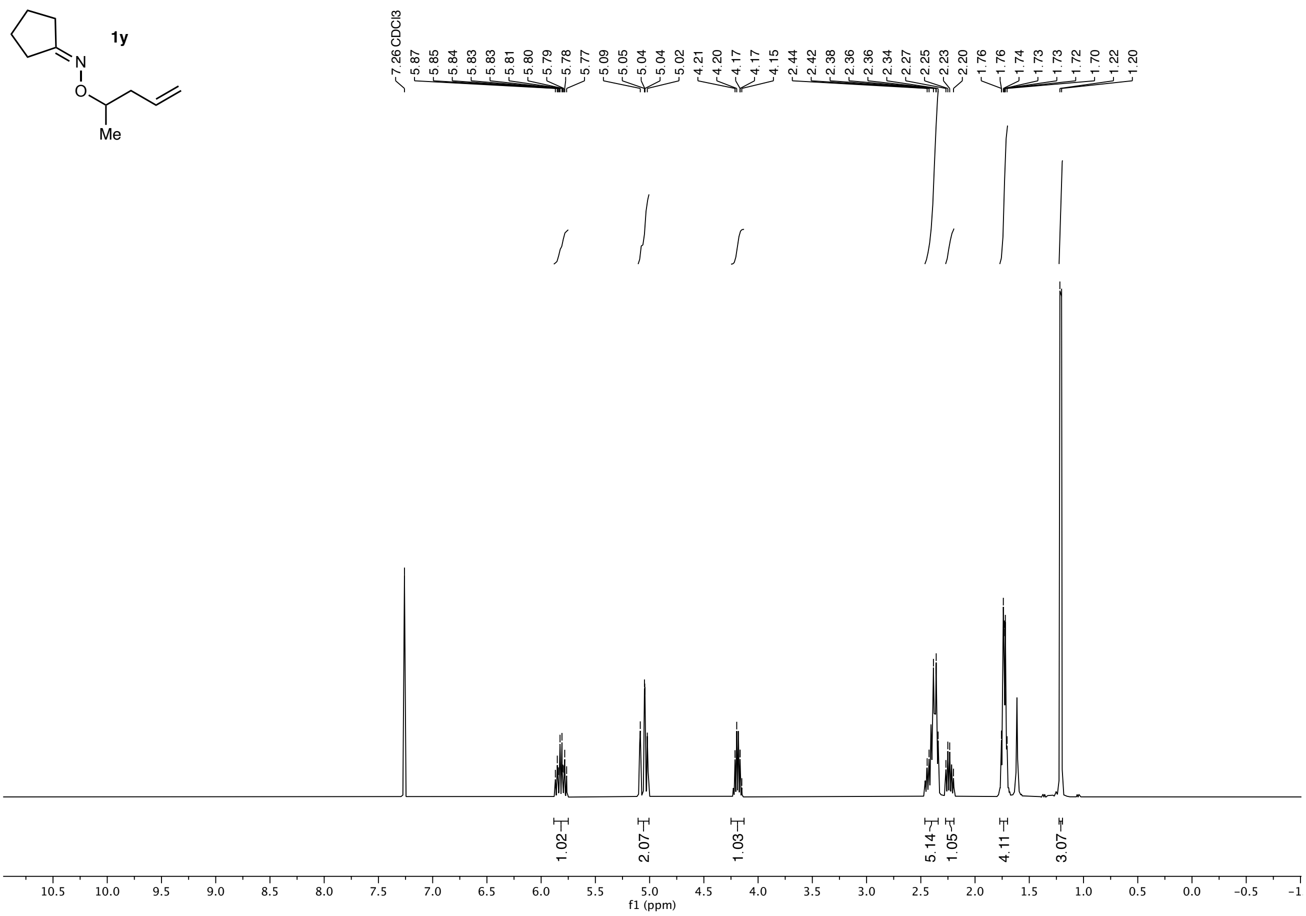
1y
N
O
Me
7.26 CDCl3
5.87
5.85
5.84
5.83
5.83
5.81
5.80
5.79
5.78
5.77
5.09
5.05
5.04
5.04
5.02
4.21
4.20
4.17
4.17
4.15
2.44
2.42
2.38
2.36
2.36
2.34
2.27
2.25
2.23
2.20
1.76
1.76
1.74
1.73
1.73
1.72
1.70
1.22
1.20
1.02
2.07
1.03
5.14
1.05
4.11
3.07
f1 (ppm)

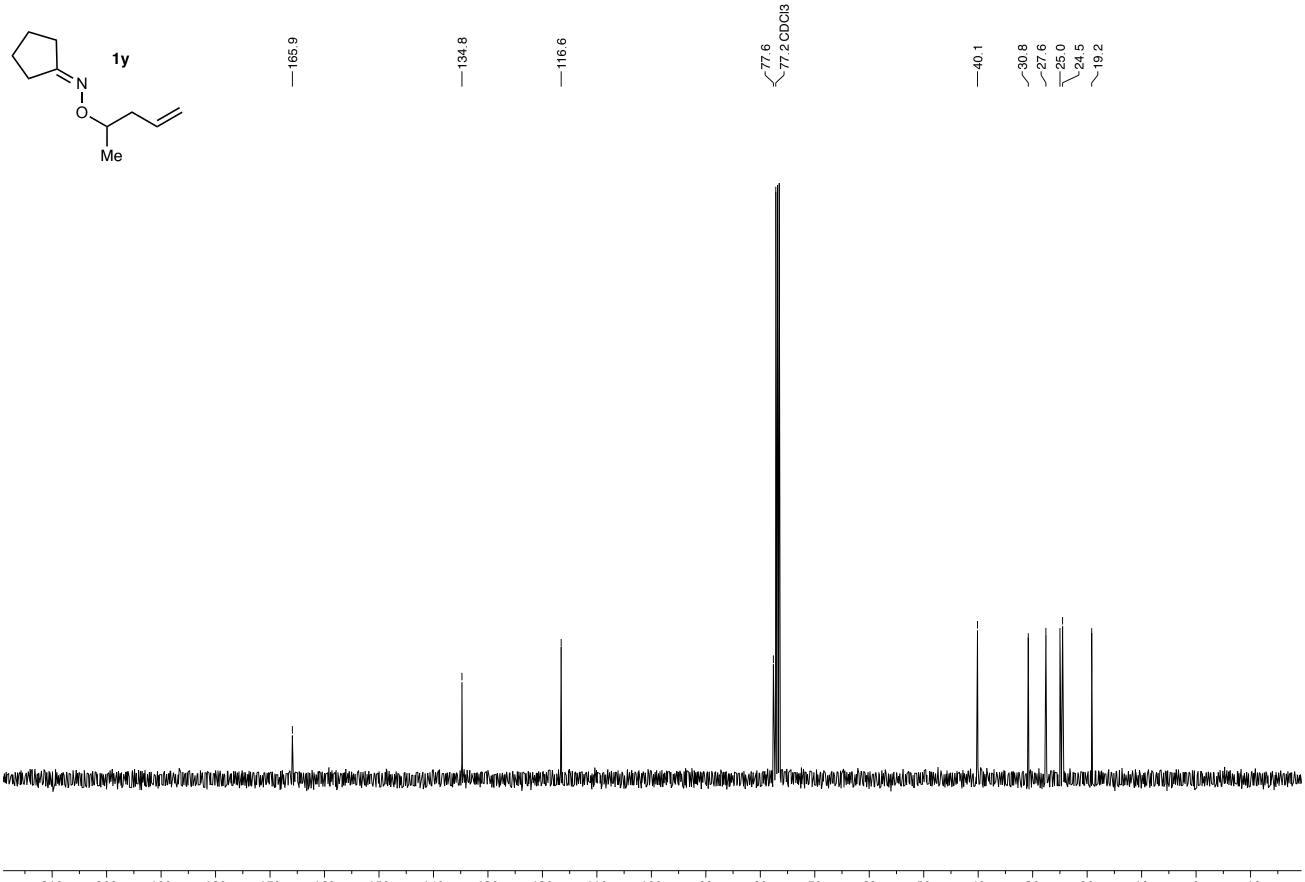
1y
N
O
Me
165.9
134.8
116.6
77.6
77.2 CDCl3
40.1
30.8
27.6
25.0
24.5
19.2
f1 (ppm)

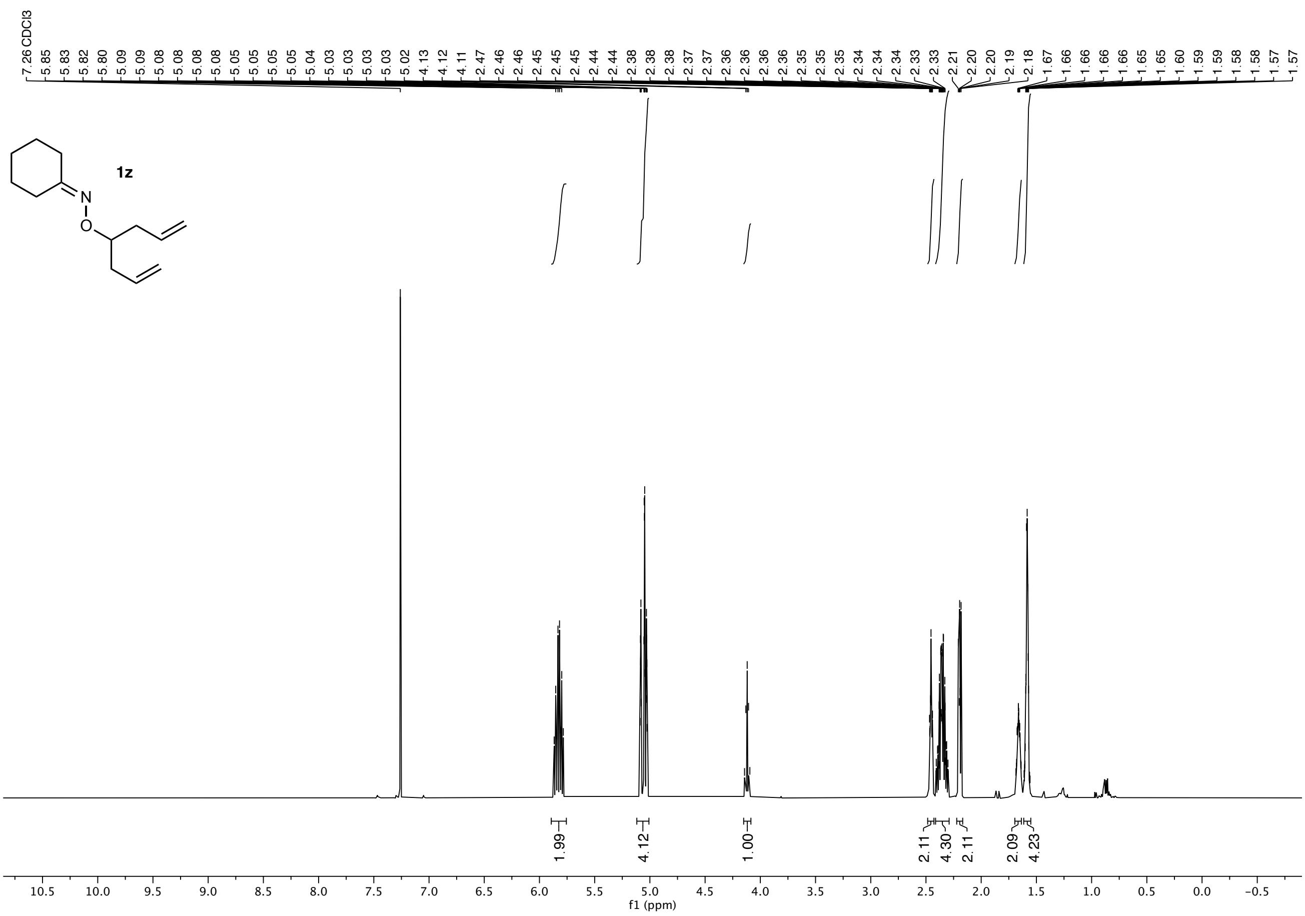
7.26 CDCl3
5.85
5.83
5.82
5.80
5.09
5.09
5.08
5.08
5.08
5.08
5.05
5.05
5.05
5.04
5.03
5.03
5.03
5.03
5.02
4.13
4.12
4.11
2.47
2.46
2.46
2.45
2.45
2.45
2.44
2.38
2.38
2.38
2.37
2.37
2.36
2.36
2.36
2.35
2.35
2.35
2.34
2.34
2.34
2.33
2.33
2.21
2.20
2.20
2.19
2.18
1.67
1.66
1.66
1.66
1.66
1.65
1.65
1.60
1.59
1.59
1.58
1.58
1.57
1.57
1z
N
O
1.99
4.12
1.00
2.11
4.30
2.11
2.09
4.23
10.5 10.0 9.5 9.0 8.5 8.0 7.5 7.0 6.5 6.0 5.5 5.0 4.5 4.0 3.5 3.0 2.5 2.0 1.5 1.0 0.5 0.0 -0.5
f1 (ppm)

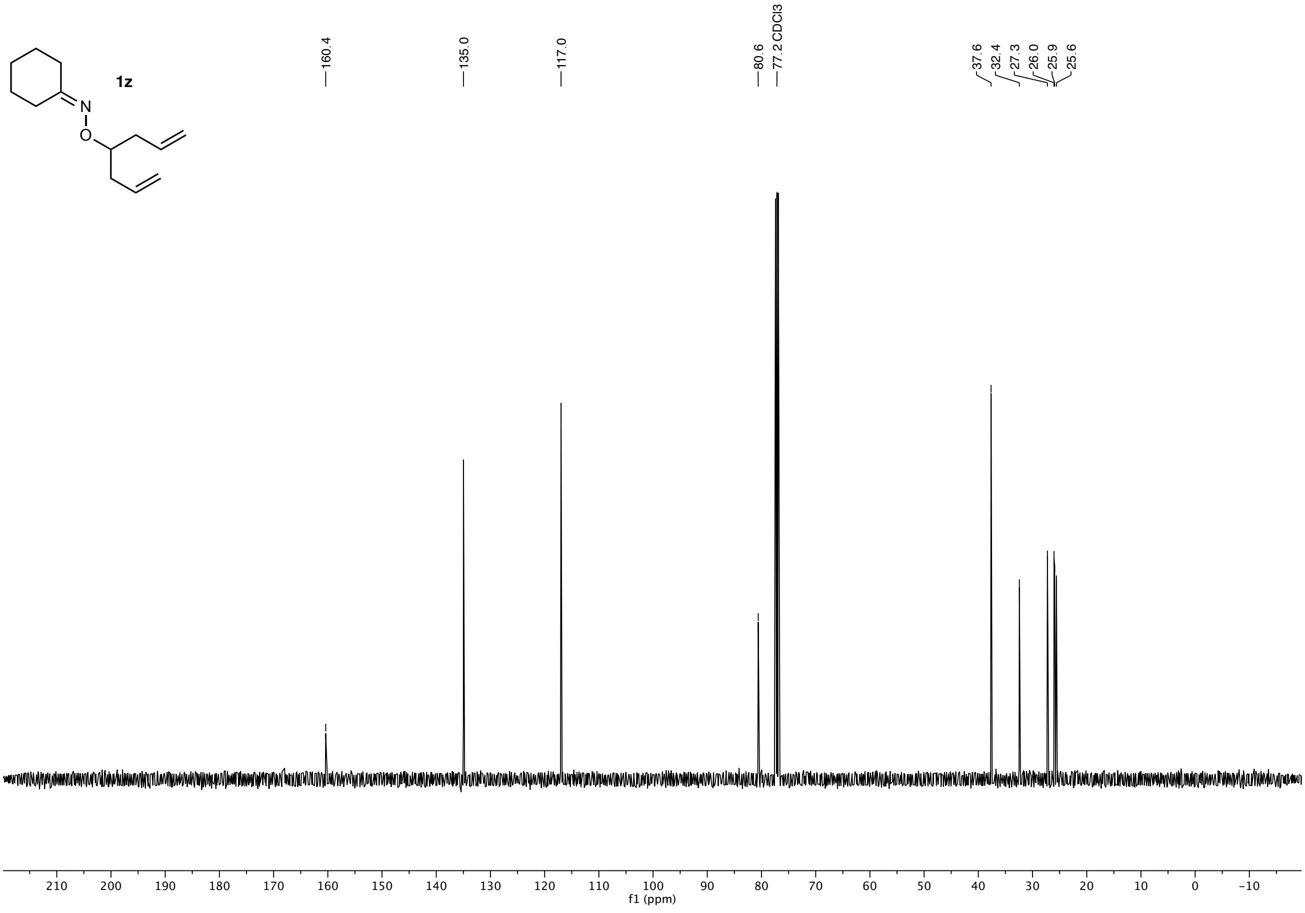
1z
N
O
160.4
135.0
117.0
80.6
77.2 CDCl3
37.6
32.4
27.3
26.0
25.9
25.6
210 200 190 180 170 160 150 140 130 120 110 100 90 80 70 60 50 40 30 20 10 0 -10
f1 (ppm)

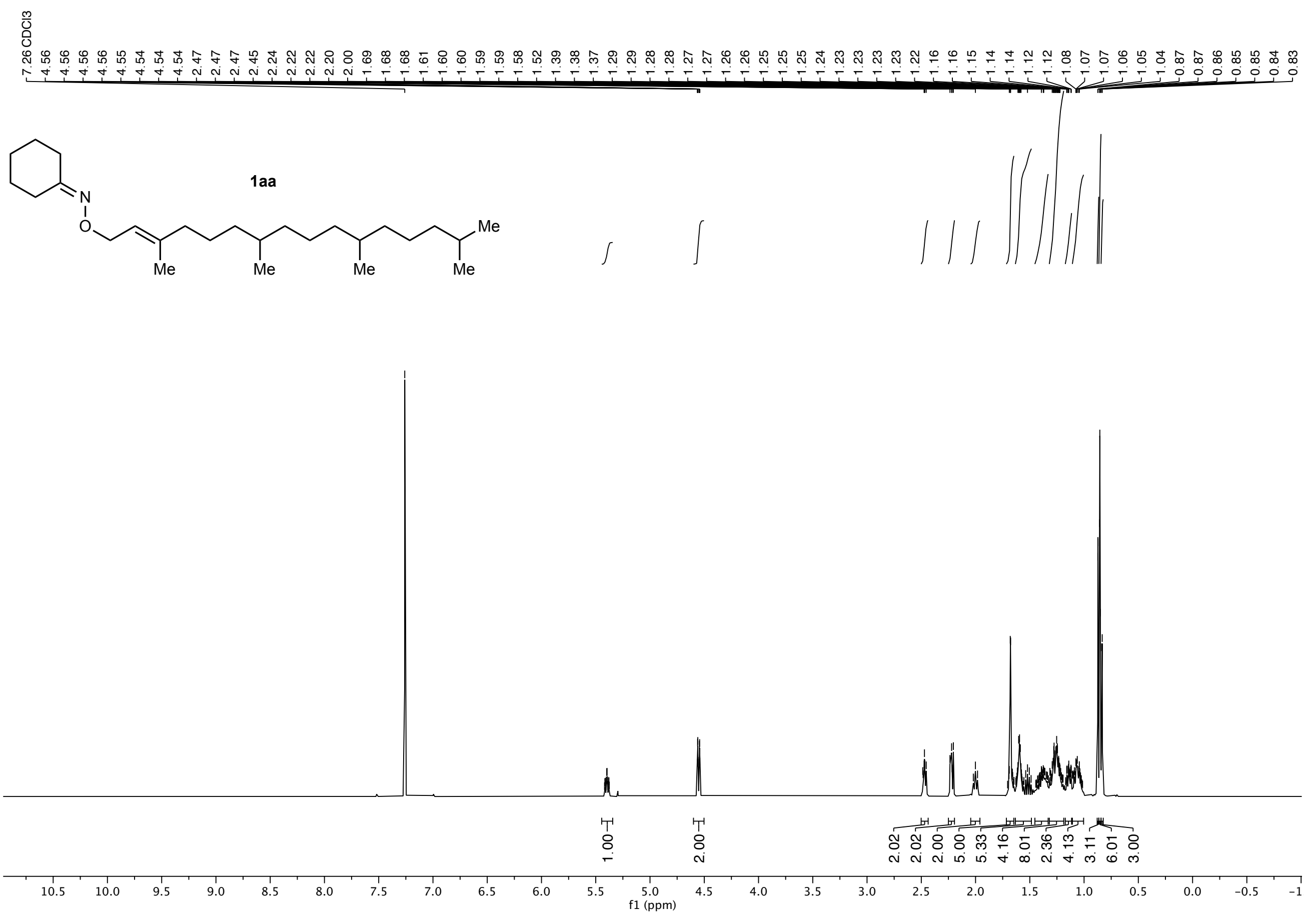
7.26 CDCl3
4.56 4.56 4.56 4.56 4.55 4.54 4.54 4.54 2.47 2.47 2.47 2.45 2.24 2.22 2.22 2.20 2.00 1.69 1.68 1.68 1.61 1.60 1.60 1.59 1.59 1.58 1.52 1.39 1.38 1.37 1.29 1.29 1.28 1.28 1.27 1.27 1.26 1.26 1.25 1.25 1.25 1.24 1.23 1.23 1.23 1.23 1.22 1.16 1.16 1.15 1.14 1.14 1.12 1.12 1.08 1.07 1.07 1.06 1.05 1.04 0.87 0.87 0.86 0.85 0.85 0.84 0.83
1aa
N
O
Me
Me
Me
Me
Me
1.00
2.00
2.02 2.02 2.00 5.00 5.33 4.16 8.01 2.36 4.13 3.11 6.01 3.00
f1 (ppm)

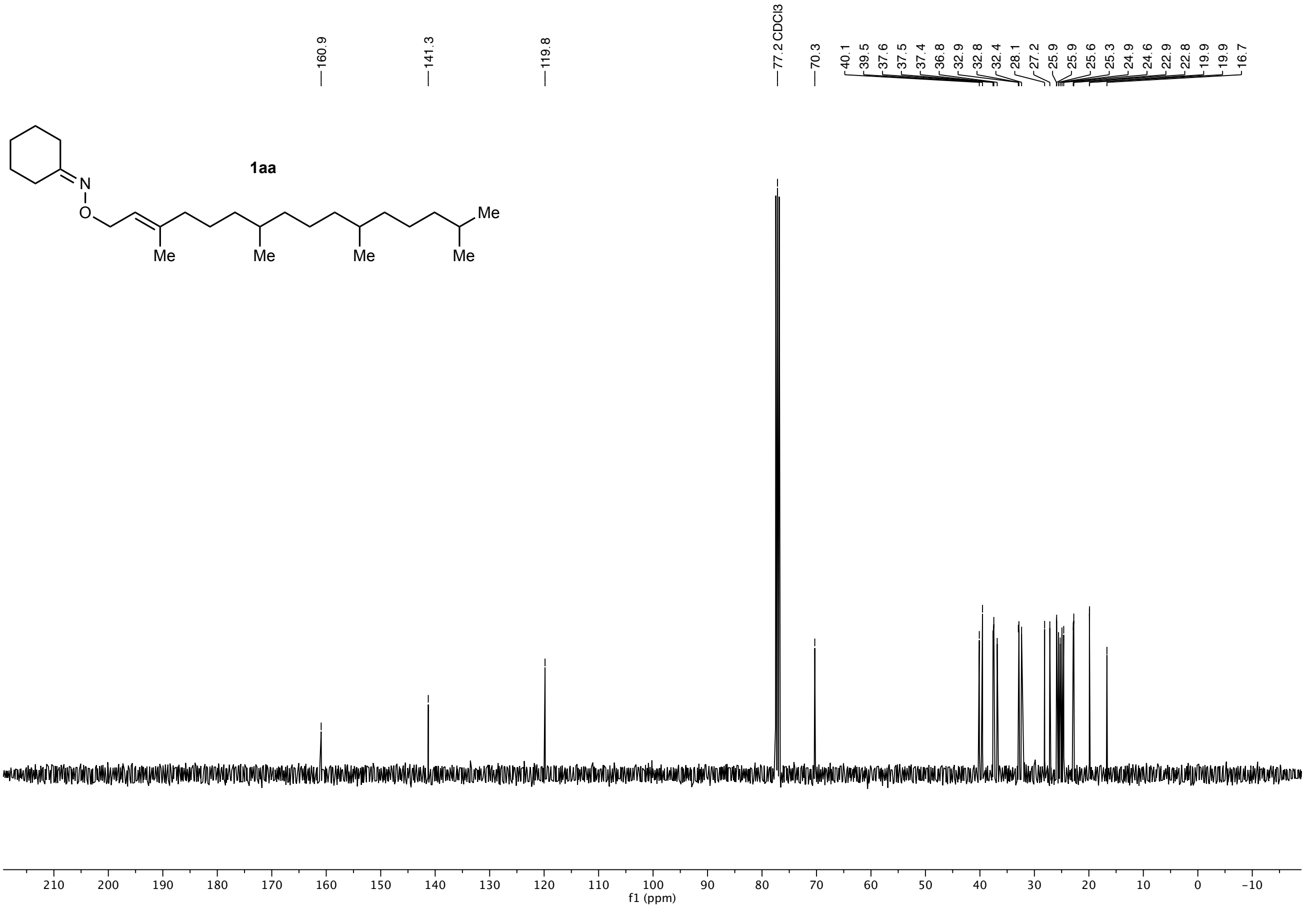
160.9
141.3
119.8
77.2 CDCl3
70.3
40.1 39.5 37.6 37.5 37.4 36.8 32.9 32.8 32.4 28.1 27.2 25.9 25.9 25.6 25.3 24.9 24.6 22.9 22.8 19.9 19.9 16.7
1aa
N
O
Me
Me
Me
Me
Me
f1 (ppm)

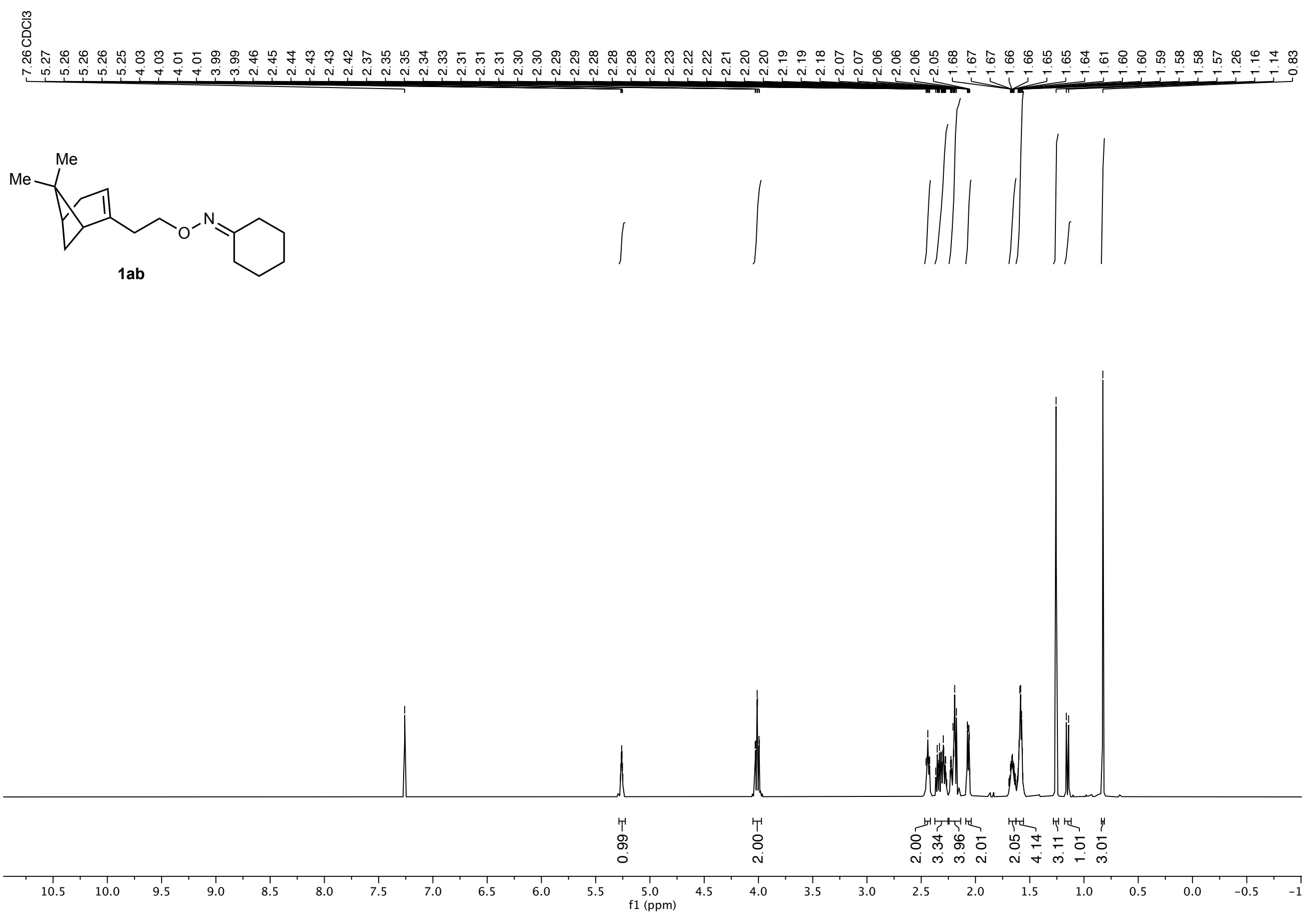
7.26 CDCl3
5.27
5.26
5.26
5.26
5.25
4.03
4.03
4.01
4.01
3.99
3.99
2.46
2.45
2.44
2.43
2.43
2.42
2.37
2.35
2.35
2.34
2.33
2.31
2.31
2.31
2.30
2.30
2.29
2.29
2.28
2.28
2.28
2.28
2.23
2.23
2.22
2.22
2.21
2.20
2.20
2.19
2.19
2.18
2.07
2.07
2.06
2.06
2.06
2.05
1.68
1.67
1.67
1.66
1.66
1.65
1.65
1.64
1.61
1.60
1.60
1.59
1.58
1.58
1.57
1.26
1.16
1.14
0.83
Me
Me
O
N
1ab
0.99
2.00
2.00
3.34
3.96
2.01
2.05
4.14
3.11
1.01
3.01
10.5
10.0
9.5
9.0
8.5
8.0
7.5
7.0
6.5
6.0
5.5
5.0
4.5
4.0
3.5
3.0
2.5
2.0
1.5
1.0
0.5
0.0
−0.5
−1
f1 (ppm)

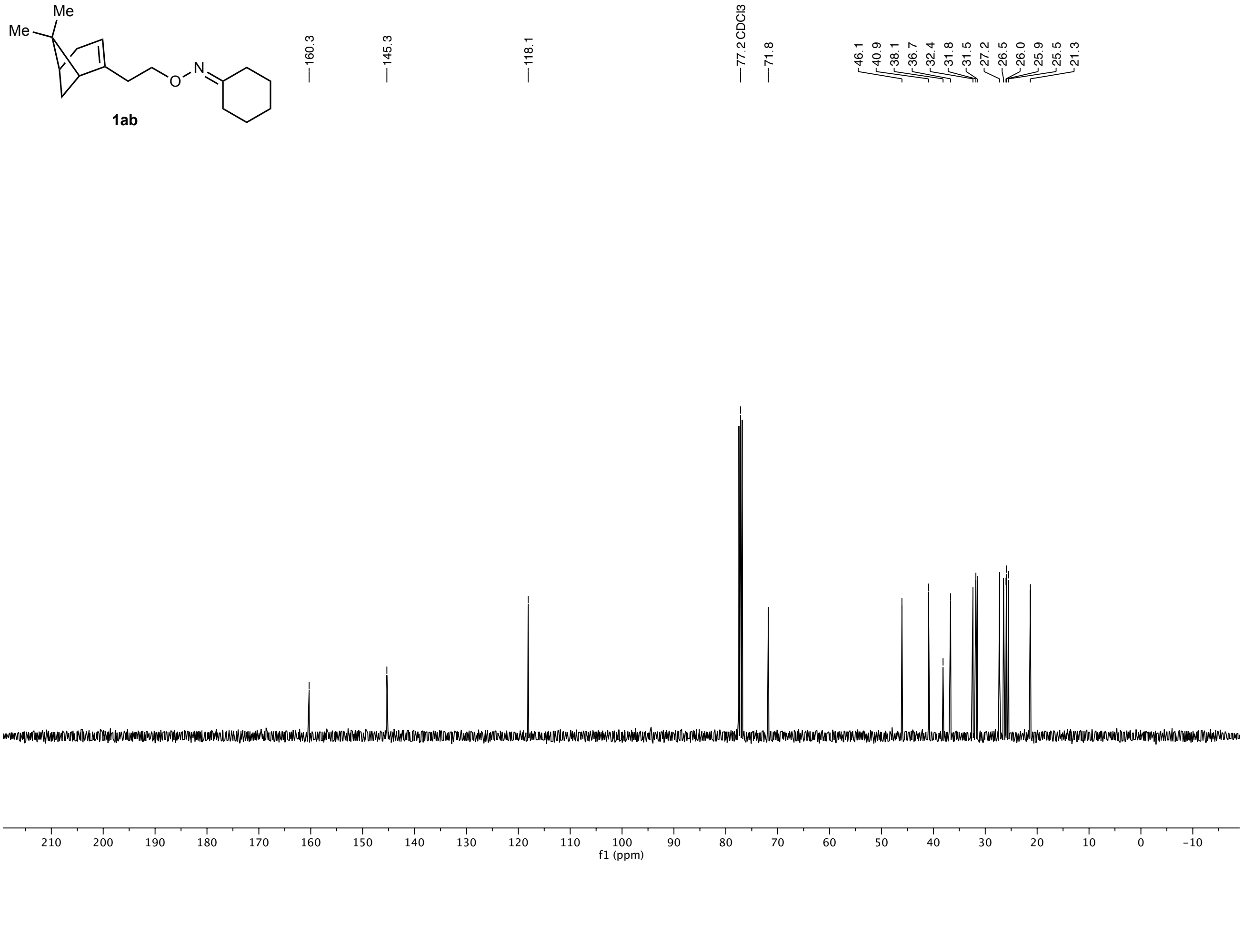
Me
Me
O
N
1ab
160.3
145.3
118.1
77.2 CDCl3
71.8
46.1
40.9
38.1
36.7
32.4
31.8
31.5
27.2
26.5
26.0
25.9
25.5
21.3
210
200
190
180
170
160
150
140
130
120
110
100
90
80
70
60
50
40
30
20
10
0
−10
f1 (ppm)

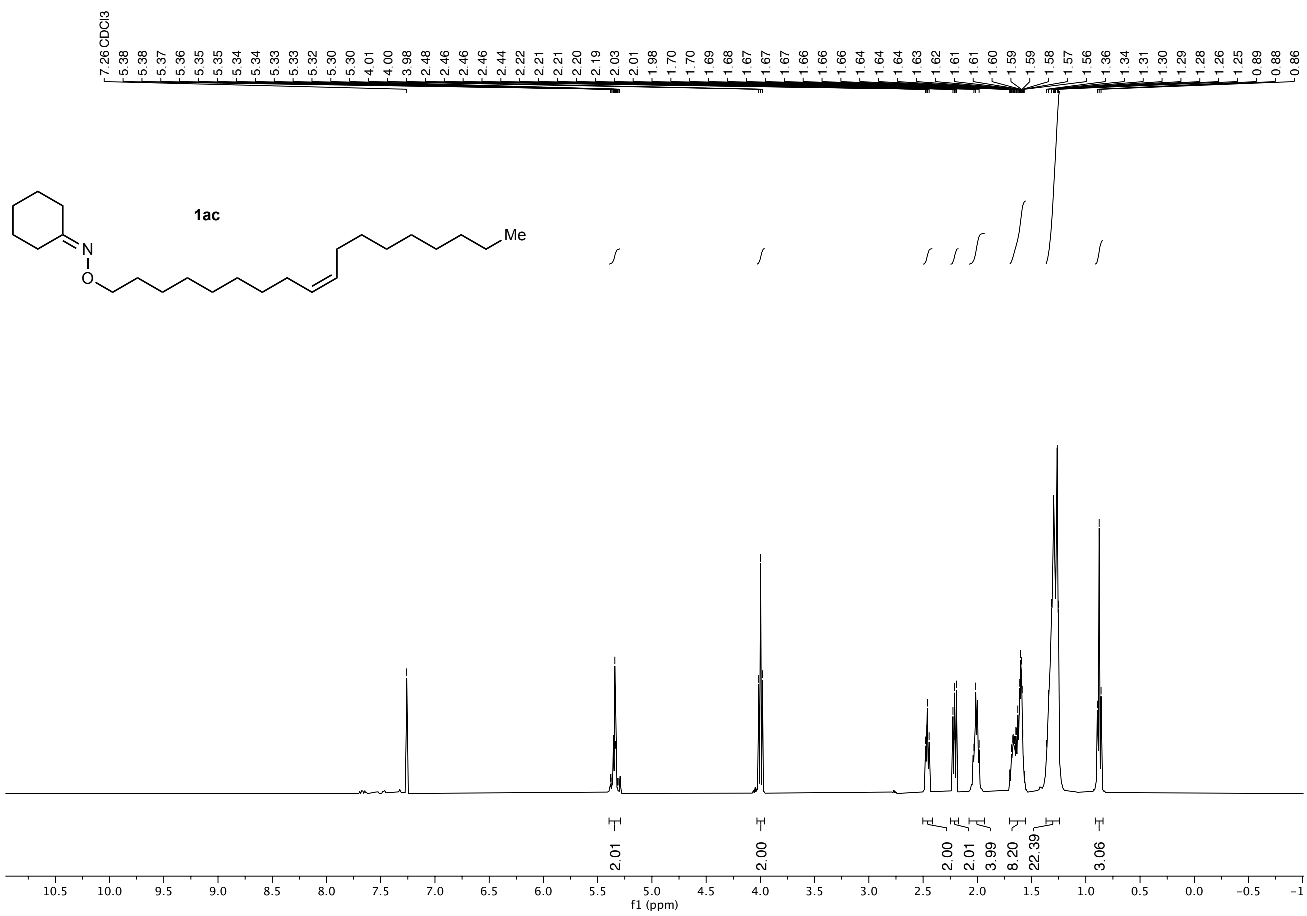
1ac
N
O
Me
7.26 CDCl3
5.38
5.38
5.37
5.36
5.35
5.35
5.34
5.34
5.33
5.33
5.32
5.30
5.30
4.01
4.00
3.98
2.48
2.46
2.46
2.46
2.44
2.22
2.21
2.21
2.20
2.19
2.03
2.01
1.98
1.70
1.70
1.69
1.68
1.67
1.67
1.66
1.66
1.66
1.64
1.64
1.64
1.63
1.62
1.61
1.61
1.60
1.59
1.59
1.58
1.57
1.56
1.36
1.34
1.31
1.30
1.29
1.28
1.26
1.25
0.89
0.88
0.86
2.01
2.00
2.00
2.01
3.99
8.20
22.39
3.06
f1 (ppm)

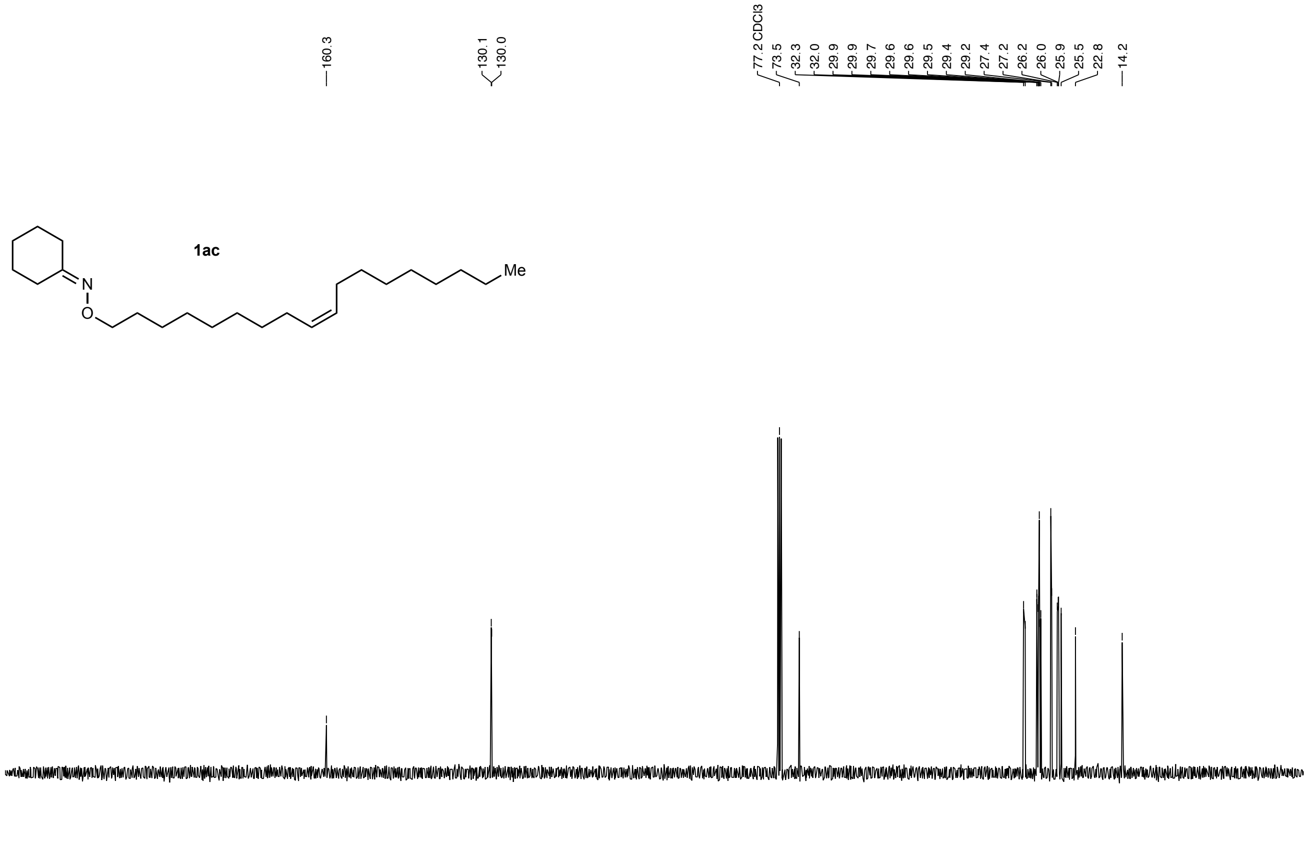
1ac
N
O
Me
160.3
130.1
130.0
77.2 CDCl3
73.5
32.3
32.0
29.9
29.9
29.7
29.6
29.6
29.5
29.4
29.2
27.4
27.2
26.2
26.0
25.9
25.5
22.8
14.2
f1 (ppm)

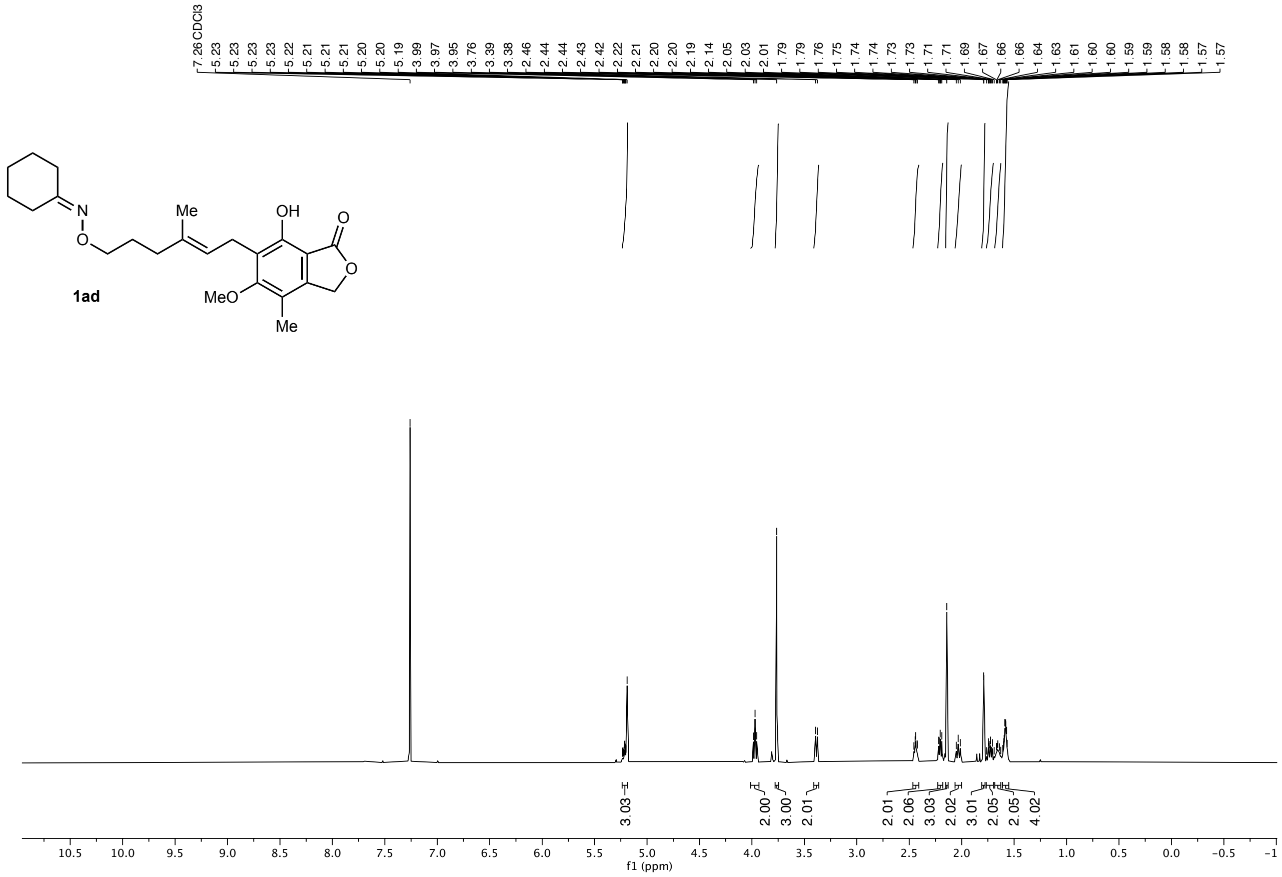
7.26 CDCl3
5.23
5.23
5.23
5.23
5.22
5.21
5.21
5.21
5.20
5.20
5.19
3.99
3.97
3.95
3.76
3.39
3.38
2.46
2.44
2.44
2.43
2.42
2.22
2.21
2.20
2.20
2.19
2.14
2.05
2.03
2.01
1.79
1.79
1.76
1.75
1.74
1.74
1.73
1.73
1.71
1.71
1.69
1.67
1.66
1.66
1.64
1.63
1.61
1.60
1.59
1.59
1.58
1.58
1.57
1.57
N
O
Me
OH
O
O
MeO
Me
1ad
3.03
2.00
3.00
2.01
2.01
2.06
3.03
2.02
3.01
2.05
2.05
4.02
10.5
10.0
9.5
9.0
8.5
8.0
7.5
7.0
6.5
6.0
5.5
5.0
4.5
4.0
3.5
3.0
2.5
2.0
1.5
1.0
0.5
0.0
-0.5
-1
f1 (ppm)

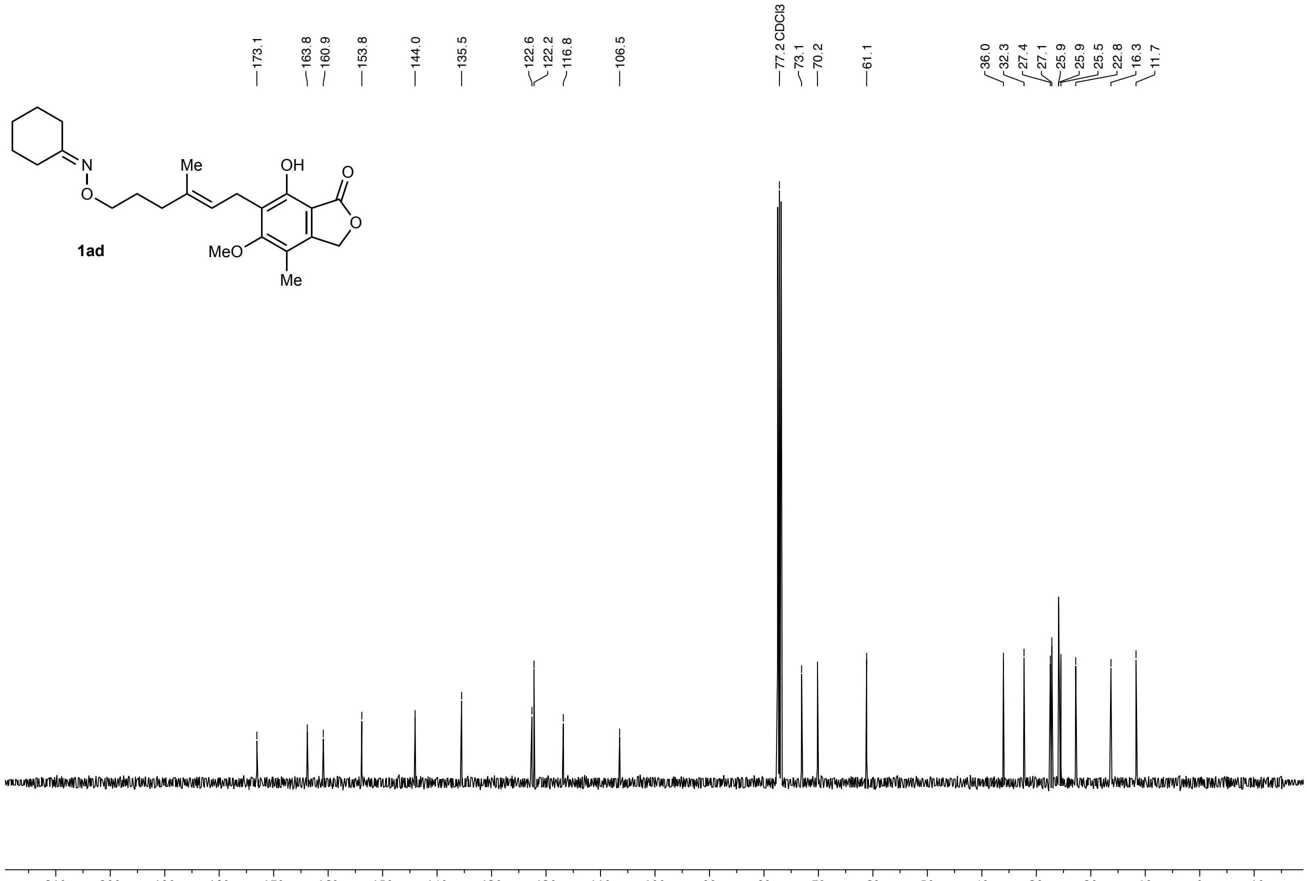
173.1
163.8
160.9
153.8
144.0
135.5
122.6
122.2
116.8
106.5
77.2 CDCl3
73.1
70.2
61.1
36.0
32.3
27.4
27.1
25.9
25.9
25.5
22.8
16.3
11.7
N
O
Me
OH
O
O
MeO
Me
1ad
210
200
190
180
170
160
150
140
130
120
110
100
90
80
70
60
50
40
30
20
10
0
-10
f1 (ppm)

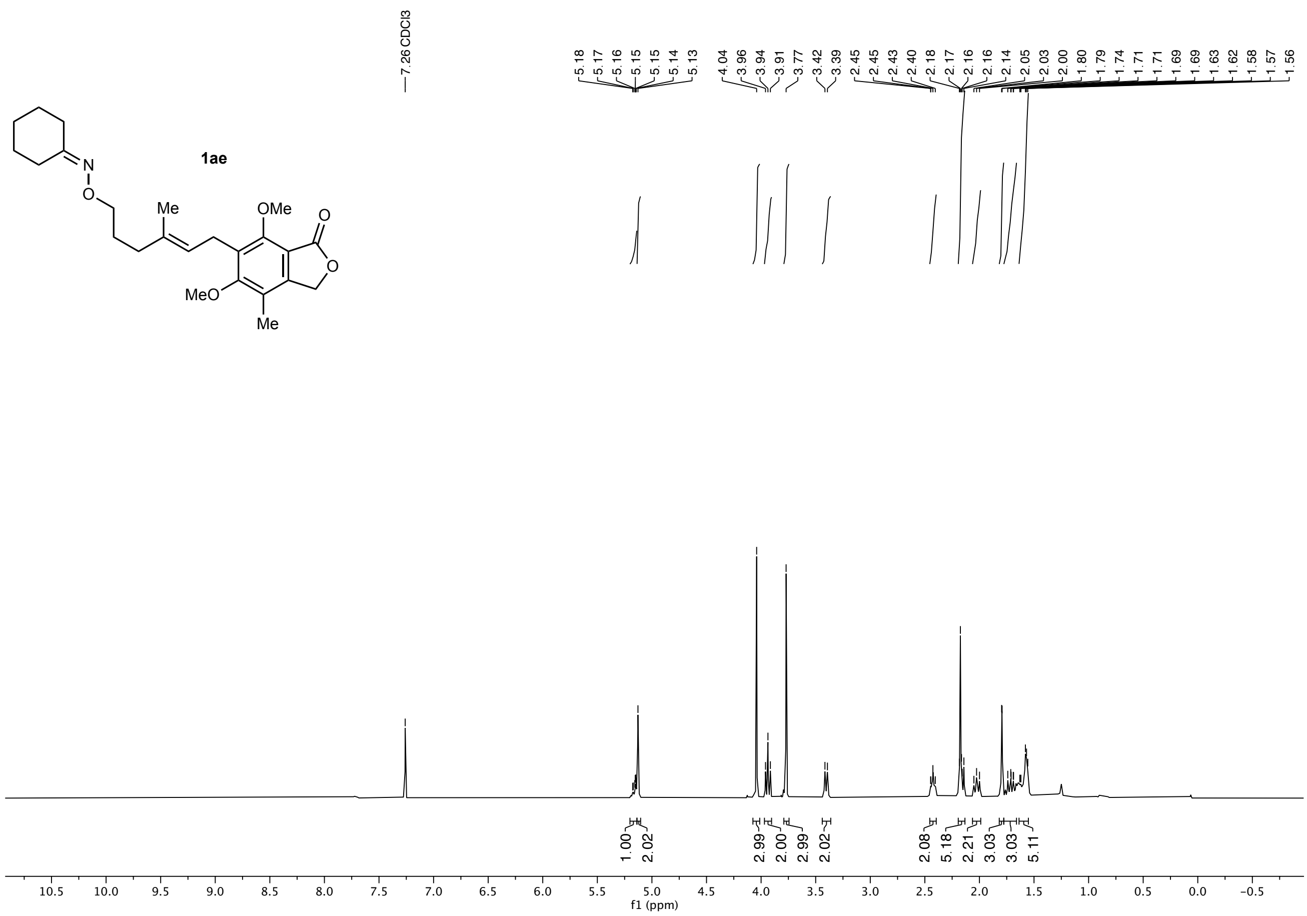
1ae
OMe
MeO
Me
O
N
7.26 CDCl3
5.18 5.17 5.16 5.15 5.15 5.14 5.13
4.04 3.96 3.94 3.91 3.77 3.42 3.39
2.45 2.45 2.43 2.40 2.18 2.17 2.16 2.16 2.14 2.05 2.03 2.00 1.80 1.79 1.74 1.71 1.71 1.69 1.69 1.63 1.62 1.58 1.57 1.56
1.00 2.02 2.99 2.00 2.99 2.02 2.08 5.18 2.21 3.03 3.03 5.11
f1 (ppm)

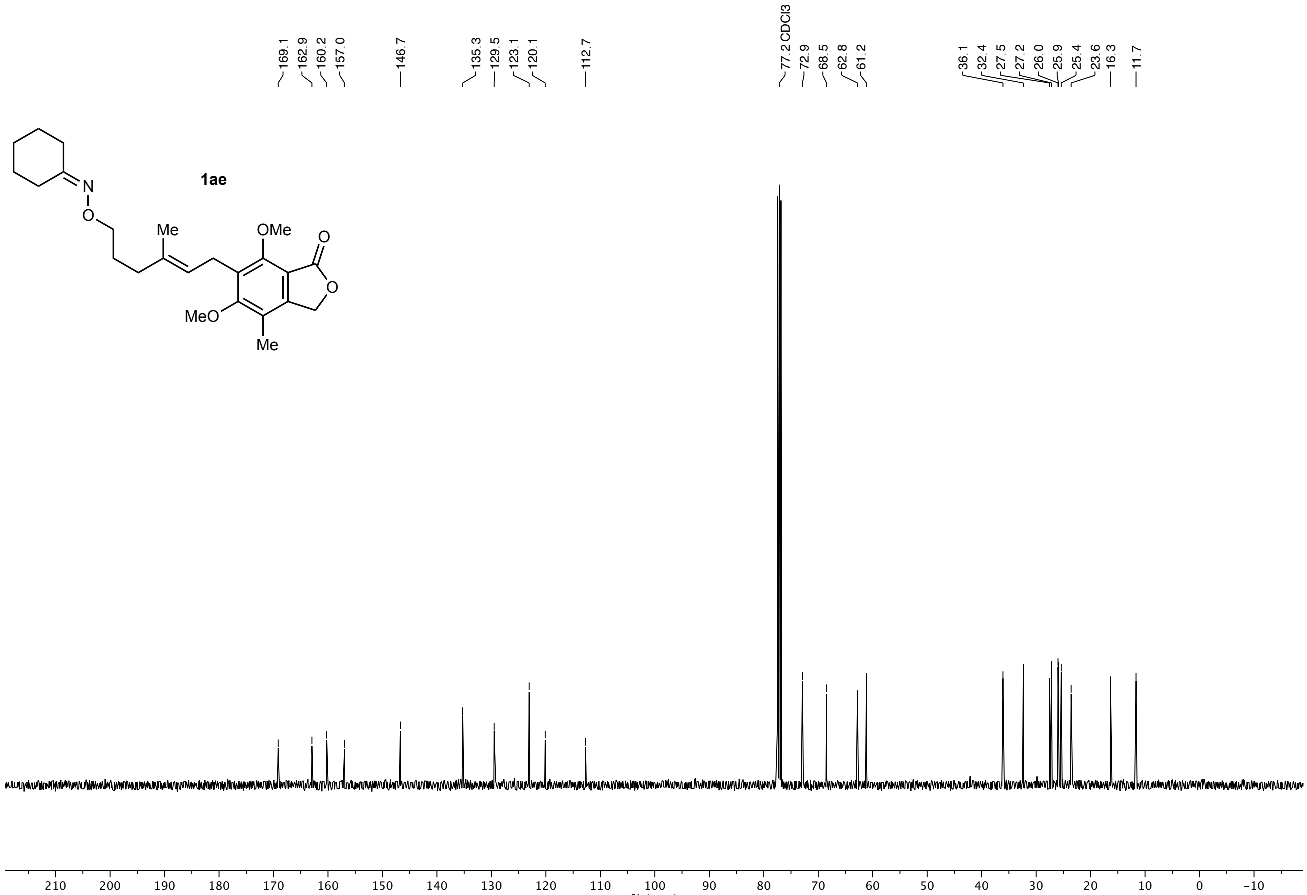
1ae
OMe
MeO
Me
O
N
169.1 162.9 160.2 157.0 146.7 135.3 129.5 123.1 120.1 112.7
77.2 CDCl3 72.9 68.5 62.8 61.2
36.1 32.4 27.5 27.2 26.0 25.9 25.4 23.6 16.3 11.7
f1 (ppm)

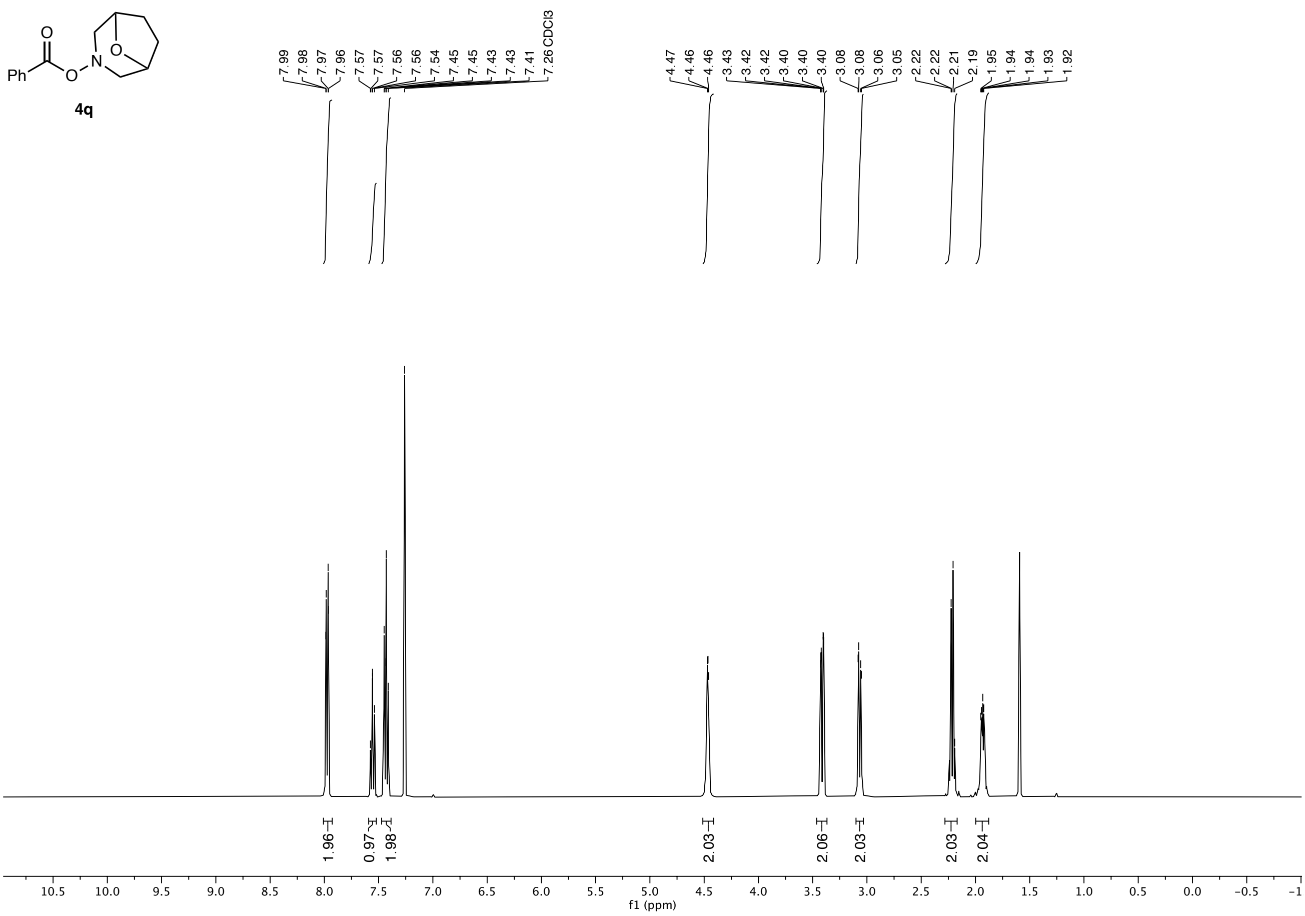

7.99 7.98 7.97 7.96 7.57 7.57 7.56 7.56 7.54 7.45 7.45 7.43 7.43 7.41 7.26 CDCl3
4.47 4.46 4.46 3.43 3.42 3.42 3.40 3.40 3.40 3.08 3.08 3.06 3.05 2.22 2.22 2.21 2.19 1.95 1.94 1.93 1.92
1.96 0.97 1.98 2.03 2.06 2.03 2.03 2.04
10.5 10.0 9.5 9.0 8.5 8.0 7.5 7.0 6.5 6.0 5.5 5.0 4.5 4.0 3.5 3.0 2.5 2.0 1.5 1.0 0.5 0.0 −0.5 −1
f1 (ppm)

4q

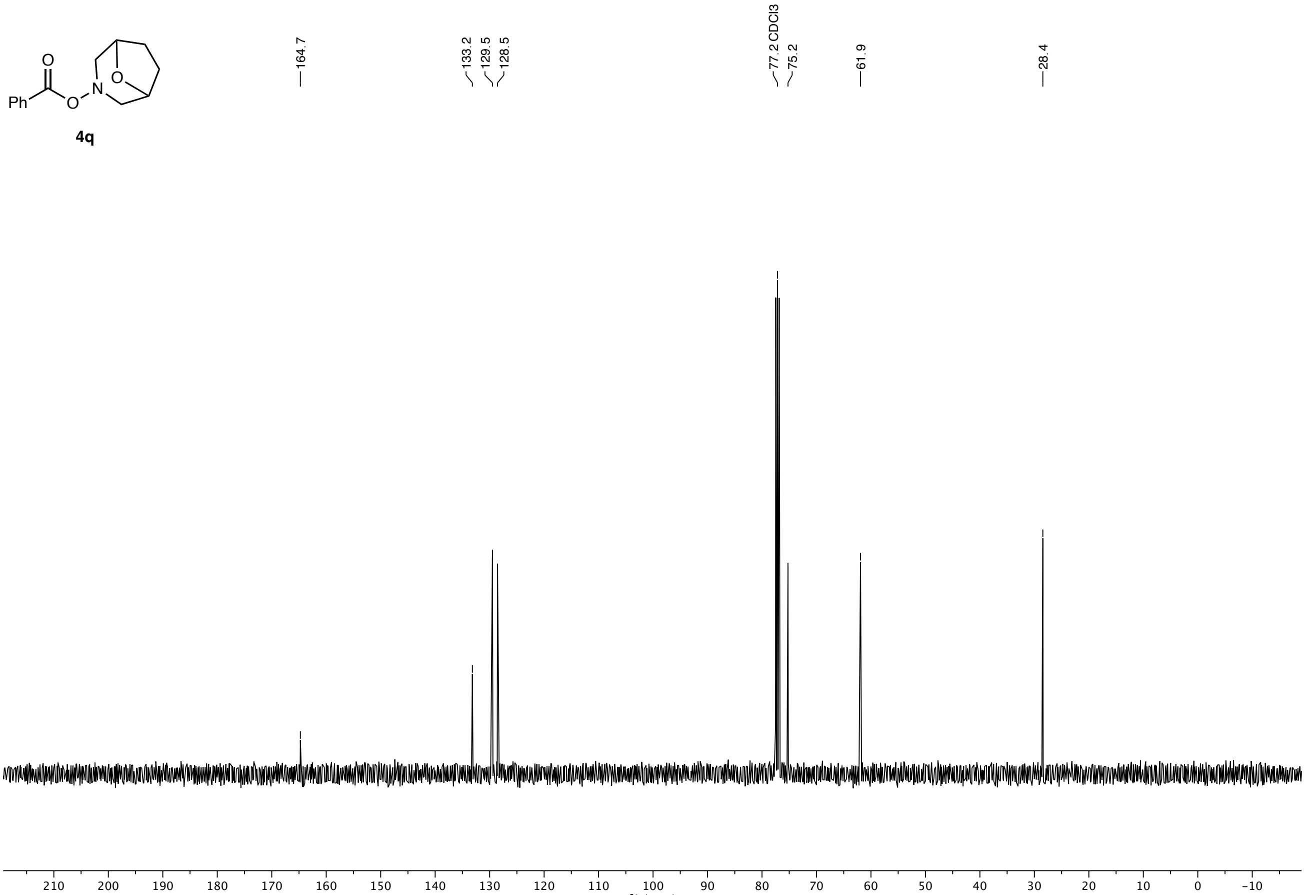

164.7
133.2 129.5 128.5
77.2 CDCl3 75.2
61.9
28.4
210 200 190 180 170 160 150 140 130 120 110 100 90 80 70 60 50 40 30 20 10 0 −10
f1 (ppm)

4q

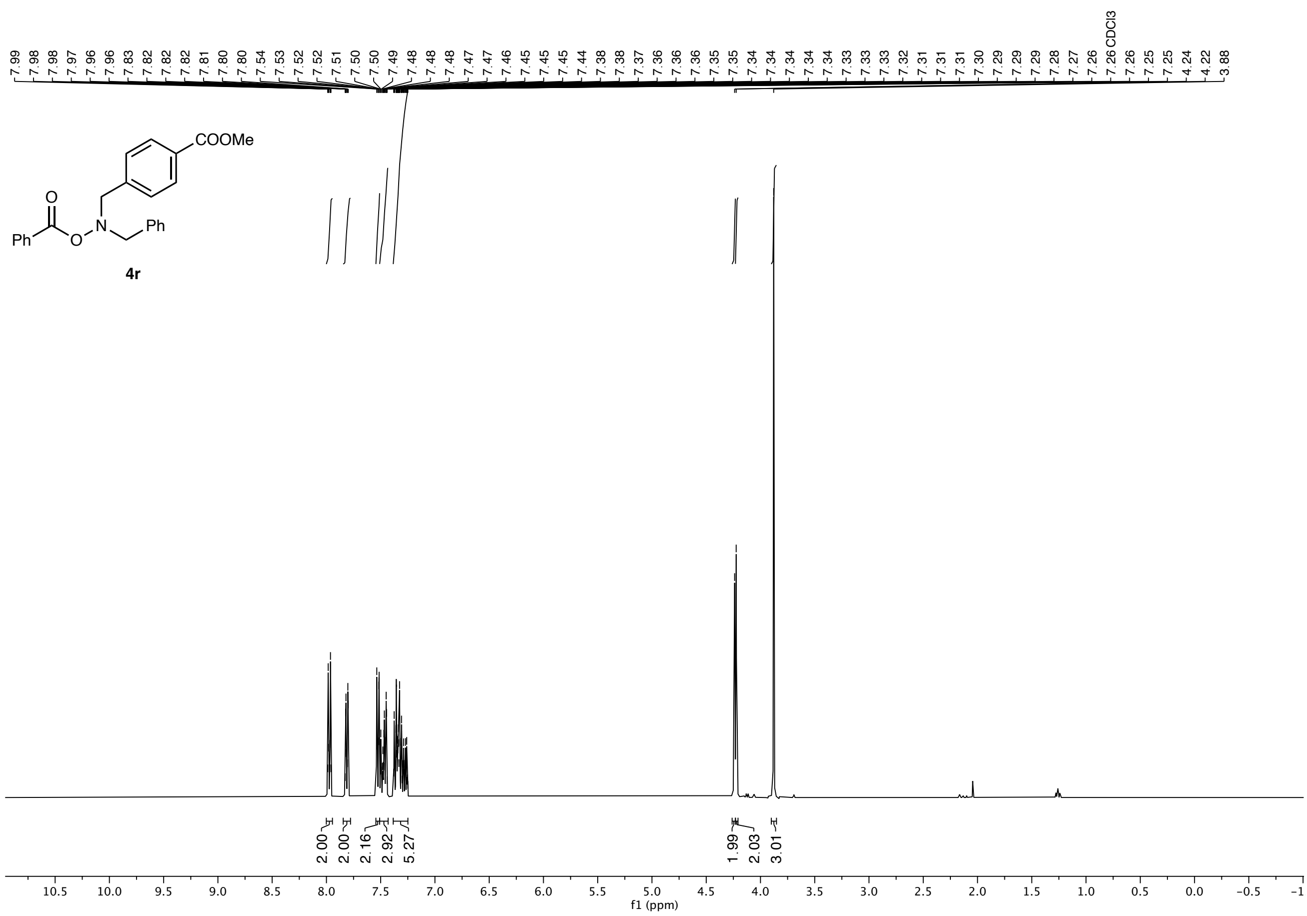
COOMe
O
Ph
O
N
Ph
4r
7.26 CDCl3
4.24
4.22
3.88
2.00
2.00
2.16
2.92
5.27
1.99
2.03
3.01
f1 (ppm)

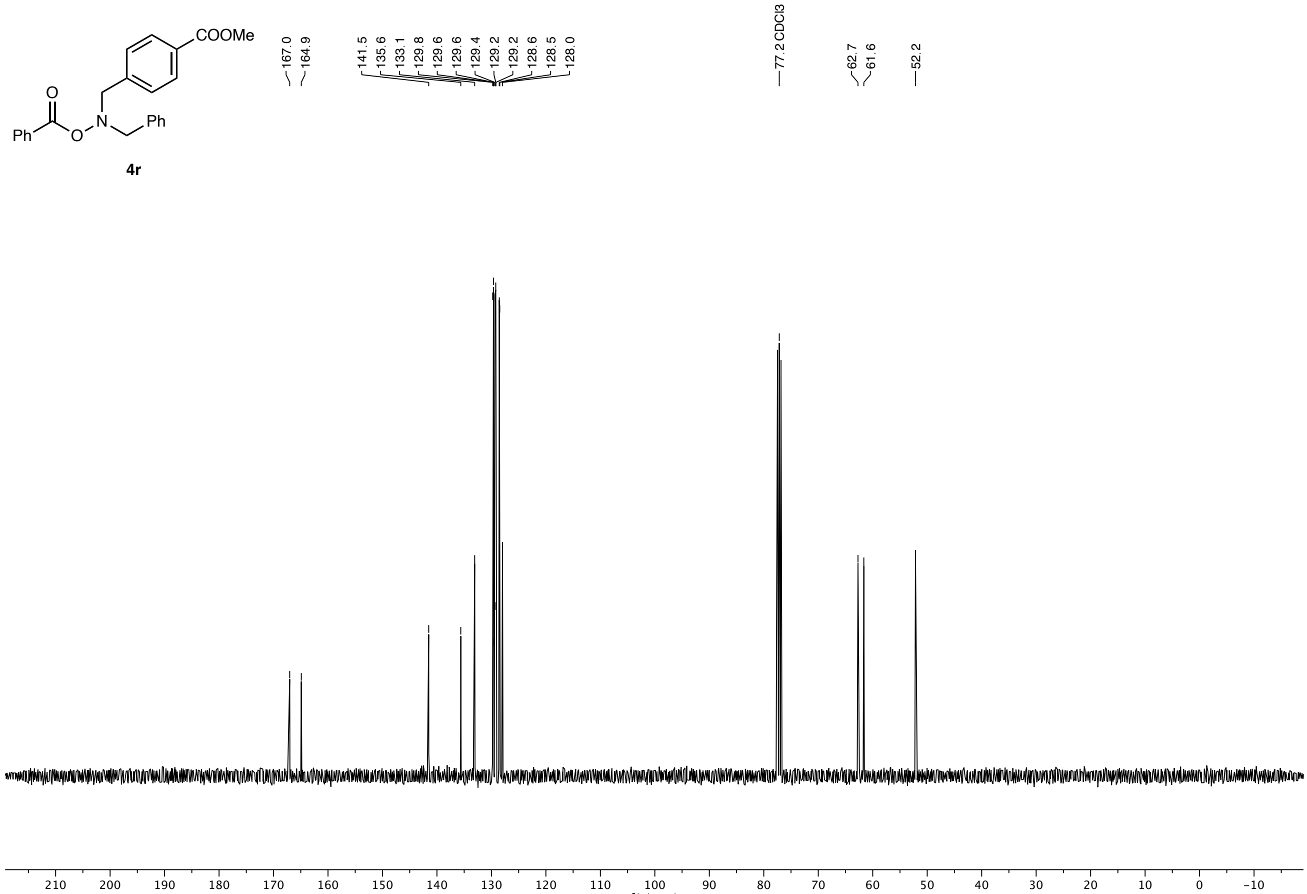
COOMe
O
Ph
O
N
Ph
4r
167.0
164.9
141.5
135.6
133.1
129.8
129.6
129.6
129.4
129.2
129.2
128.6
128.5
128.0
77.2 CDCl3
62.7
61.6
52.2
f1 (ppm)

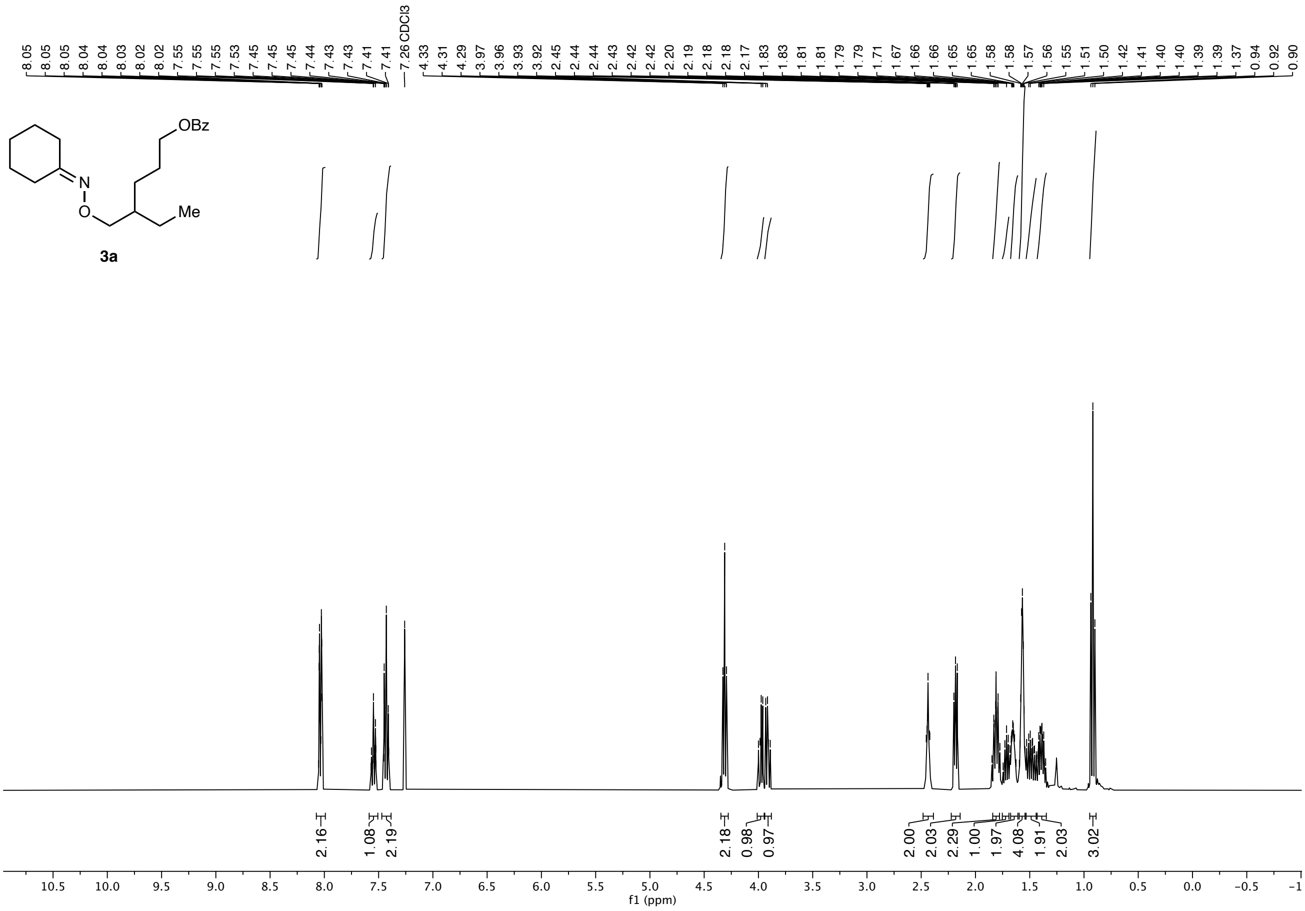
OBz
N
O
Me
3a
8.05
8.05
8.05
8.04
8.04
8.03
8.02
8.02
7.55
7.55
7.55
7.53
7.45
7.45
7.45
7.44
7.43
7.43
7.41
7.41
7.26 CDCl3
4.33
4.31
4.29
3.97
3.96
3.93
3.92
2.45
2.44
2.44
2.43
2.42
2.42
2.20
2.19
2.18
2.18
2.17
1.83
1.83
1.81
1.81
1.79
1.79
1.71
1.67
1.66
1.66
1.65
1.65
1.58
1.58
1.57
1.56
1.55
1.51
1.50
1.42
1.41
1.40
1.40
1.39
1.39
1.37
0.94
0.92
0.90
2.16
1.08
2.19
2.18
0.98
0.97
2.00
2.03
2.29
1.00
1.97
4.08
1.91
2.03
3.02
10.5
10.0
9.5
9.0
8.5
8.0
7.5
7.0
6.5
6.0
5.5
5.0
4.5
4.0
3.5
3.0
2.5
2.0
1.5
1.0
0.5
0.0
−0.5
−1
f1 (ppm)

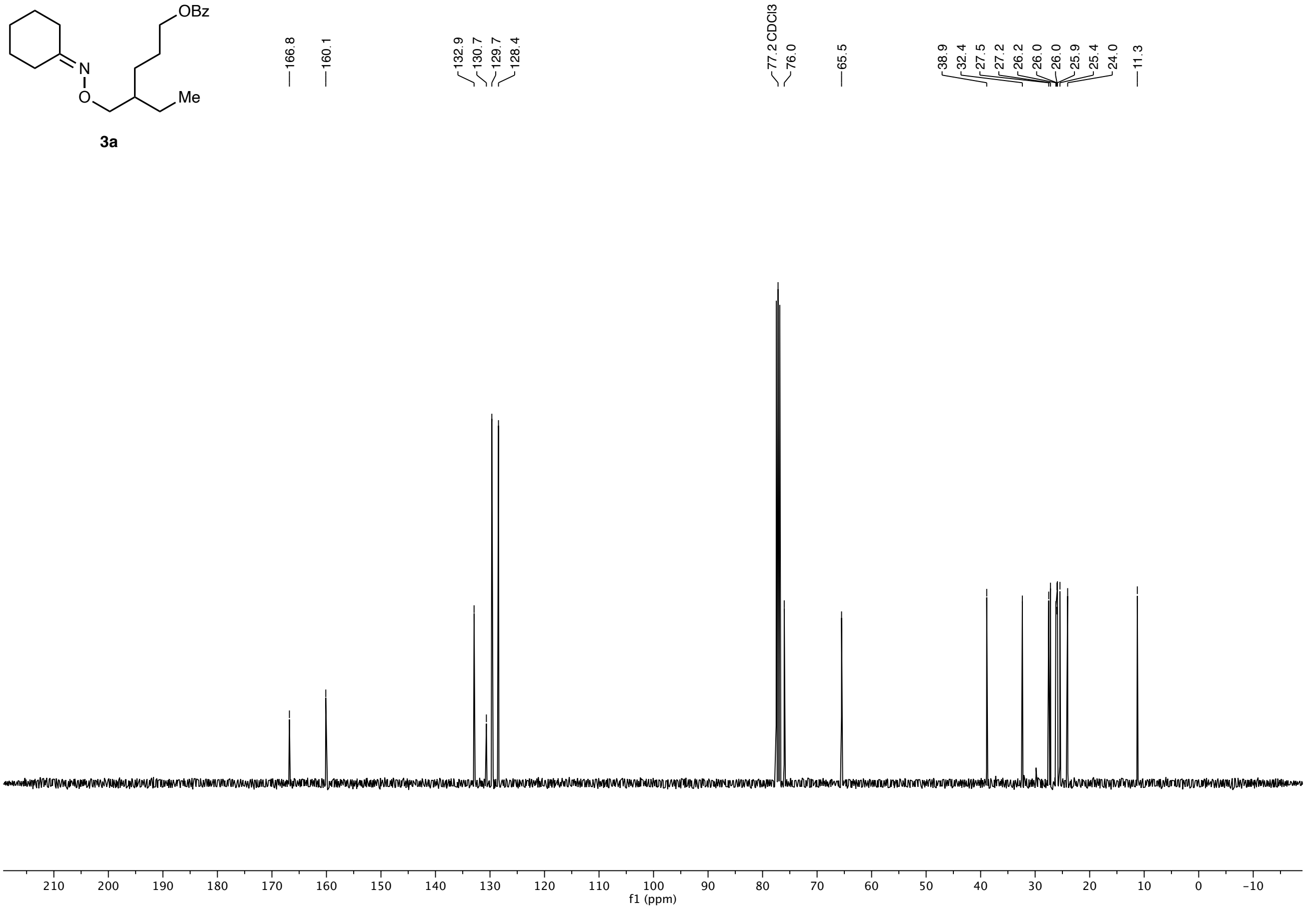
OBz
N
O
Me
3a
166.8
160.1
132.9
130.7
129.7
128.4
77.2 CDCl3
76.0
65.5
38.9
32.4
27.5
27.2
26.2
26.0
26.0
25.9
25.4
24.0
11.3
210
200
190
180
170
160
150
140
130
120
110
100
90
80
70
60
50
40
30
20
10
0
−10
f1 (ppm)

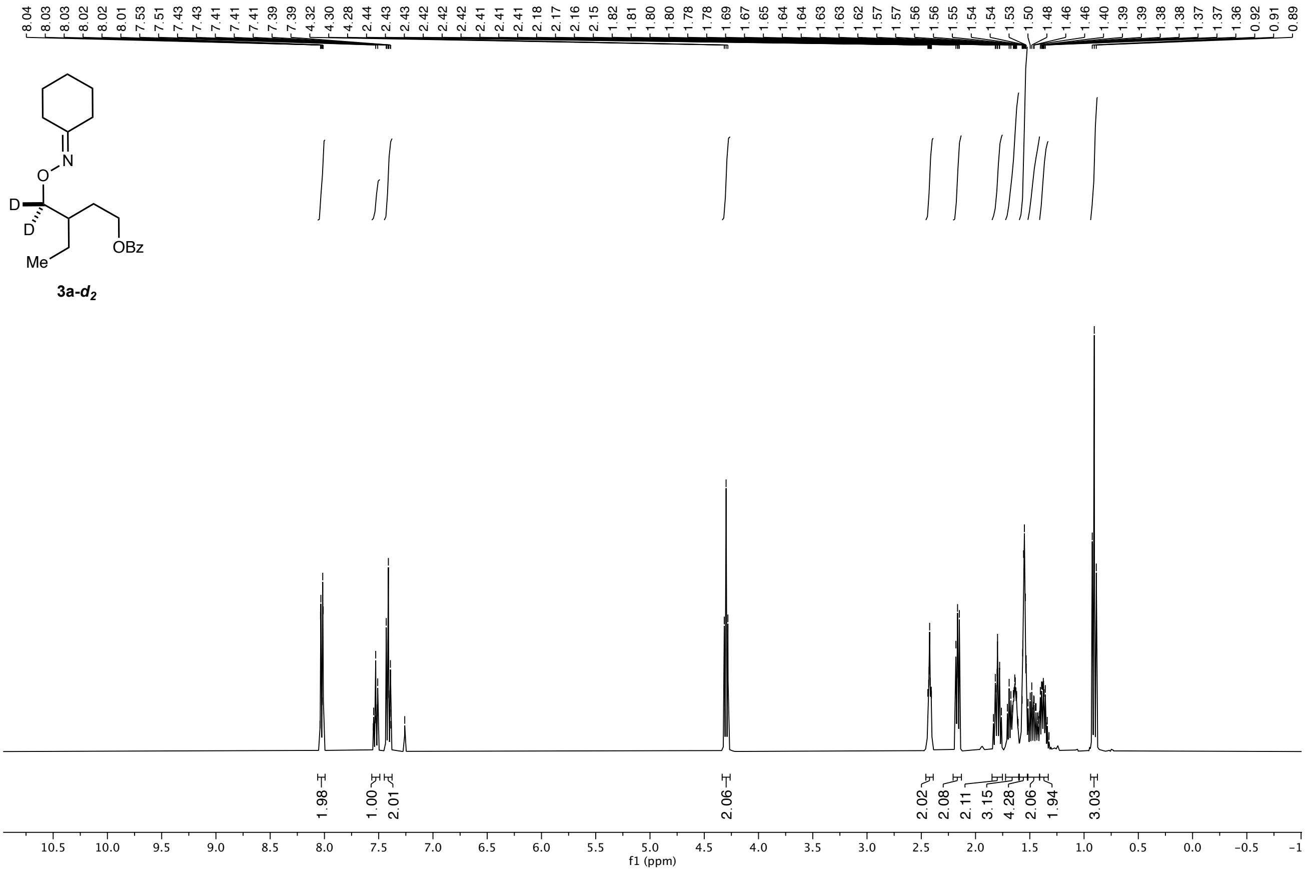
8.04 8.03 8.03 8.02 8.02 8.01 7.53 7.51 7.43 7.43 7.41 7.41 7.41 7.39 7.39 4.32 4.30 4.28 2.44 2.43 2.43 2.42 2.42 2.42 2.41 2.41 2.41 2.18 2.17 2.16 2.15 1.82 1.81 1.80 1.80 1.78 1.69 1.67 1.65 1.64 1.64 1.63 1.63 1.62 1.57 1.57 1.56 1.56 1.55 1.54 1.54 1.54 1.53 1.50 1.48 1.46 1.46 1.40 1.39 1.39 1.38 1.38 1.37 1.36 0.92 0.91 0.89
N
O
D
D
OBz
Me
3a-d2
1.98
1.00
2.01
2.06
2.02
2.08
2.11
3.15
4.28
2.06
1.94
3.03
f1 (ppm)

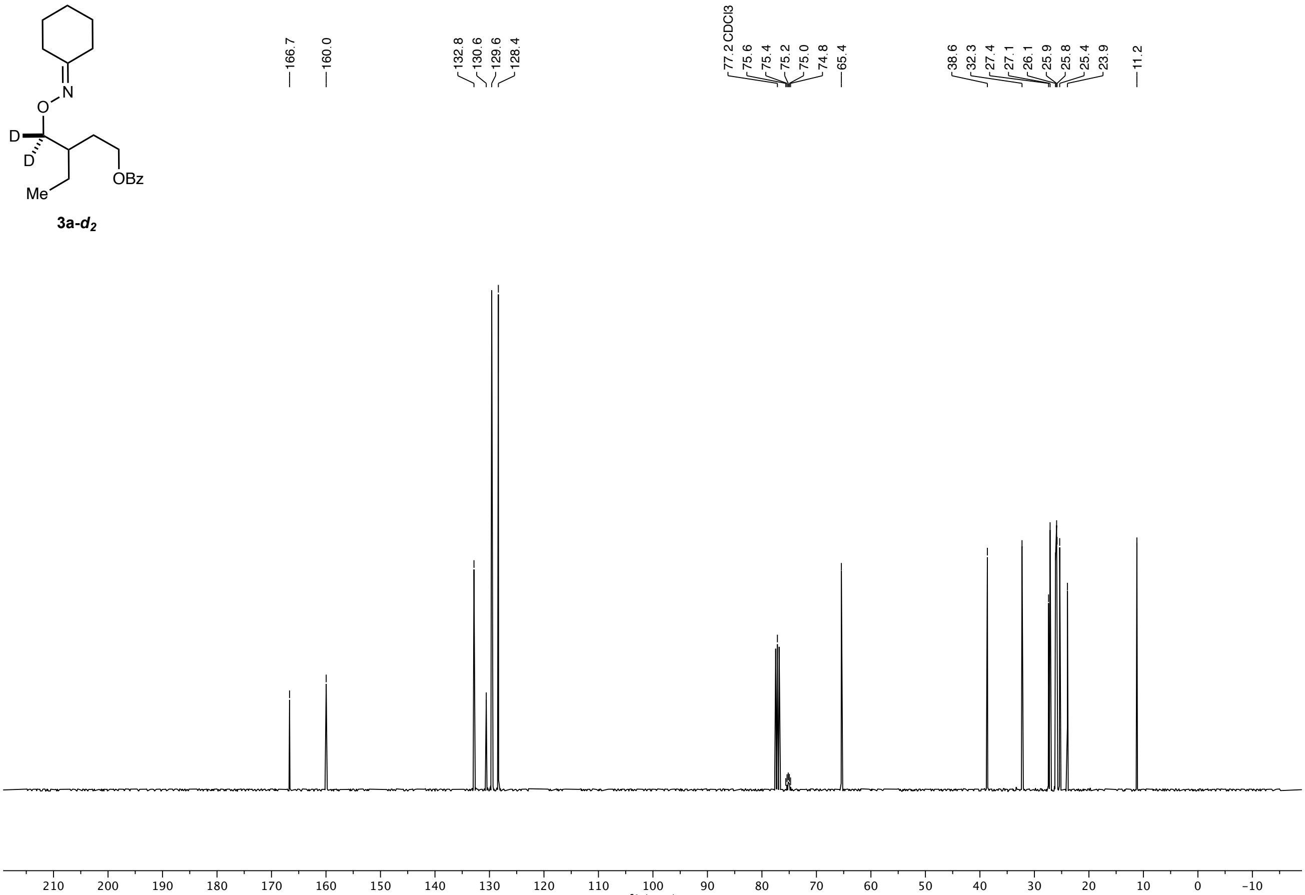
166.7
160.0
132.8 130.6 129.6 128.4
77.2 CDCl3 75.6 75.4 75.2 75.0 74.8
65.4
38.6 32.3 27.4 27.1 26.1 25.9 25.8 25.4 23.9
11.2
N
O
D
D
OBz
Me
3a-d2
f1 (ppm)

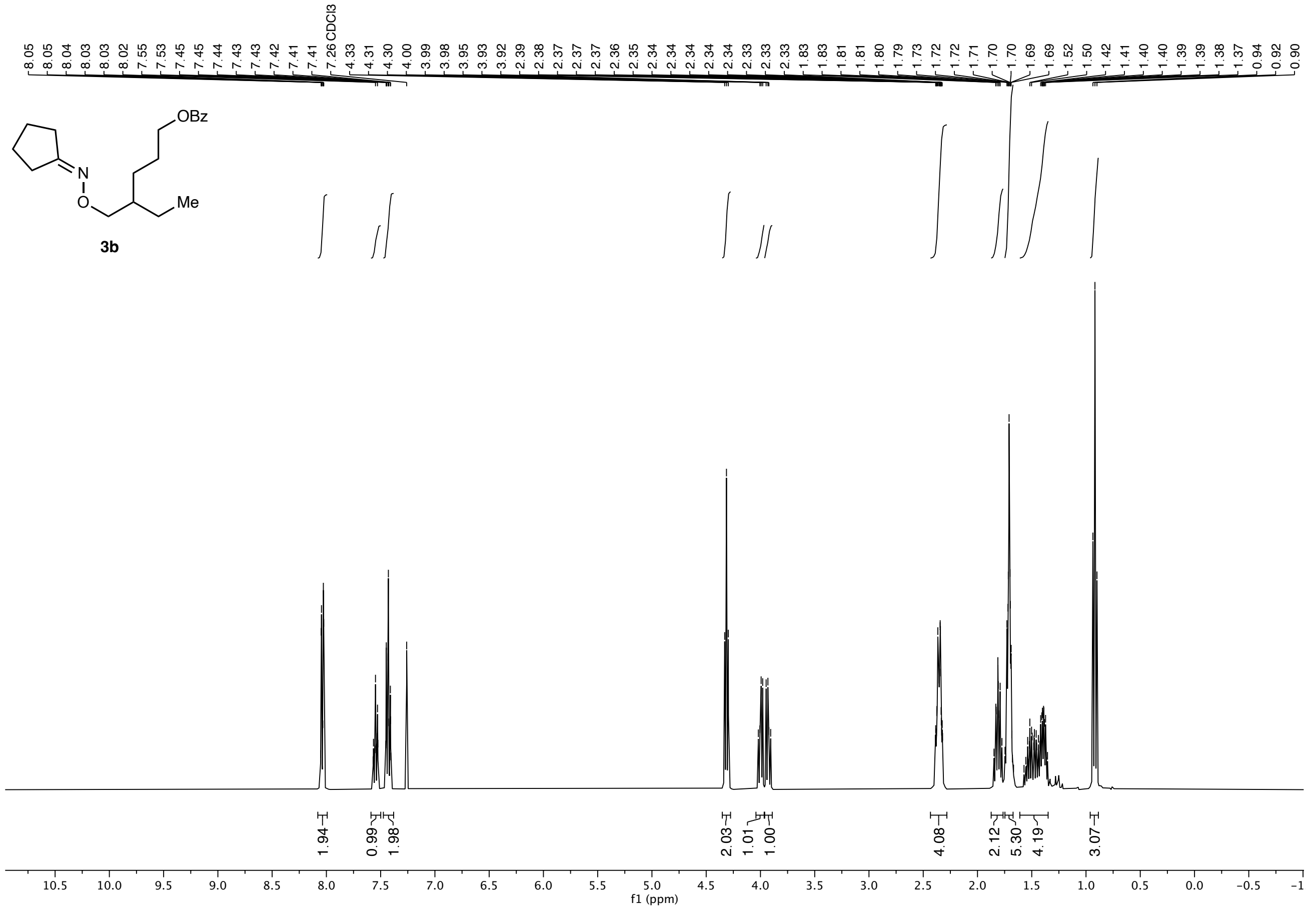
8.05 8.05 8.04 8.03 8.03 8.02 7.55 7.53 7.45 7.45 7.44 7.43 7.43 7.42 7.41 7.41 7.26 CDCl3 4.33 4.31 4.30 4.00 3.99 3.98 3.95 3.93 3.92 2.39 2.38 2.37 2.37 2.37 2.36 2.35 2.34 2.34 2.34 2.34 2.34 2.33 2.33 2.33 1.83 1.83 1.81 1.81 1.80 1.79 1.73 1.72 1.72 1.71 1.70 1.70 1.69 1.69 1.52 1.50 1.42 1.41 1.40 1.40 1.39 1.39 1.38 1.37 0.94 0.92 0.90
OBz
N
O
Me
3b
1.94
0.99
1.98
2.03
1.01
1.00
4.08
2.12
5.30
4.19
3.07
10.5 10.0 9.5 9.0 8.5 8.0 7.5 7.0 6.5 6.0 5.5 5.0 4.5 4.0 3.5 3.0 2.5 2.0 1.5 1.0 0.5 0.0 −0.5 −1
f1 (ppm)

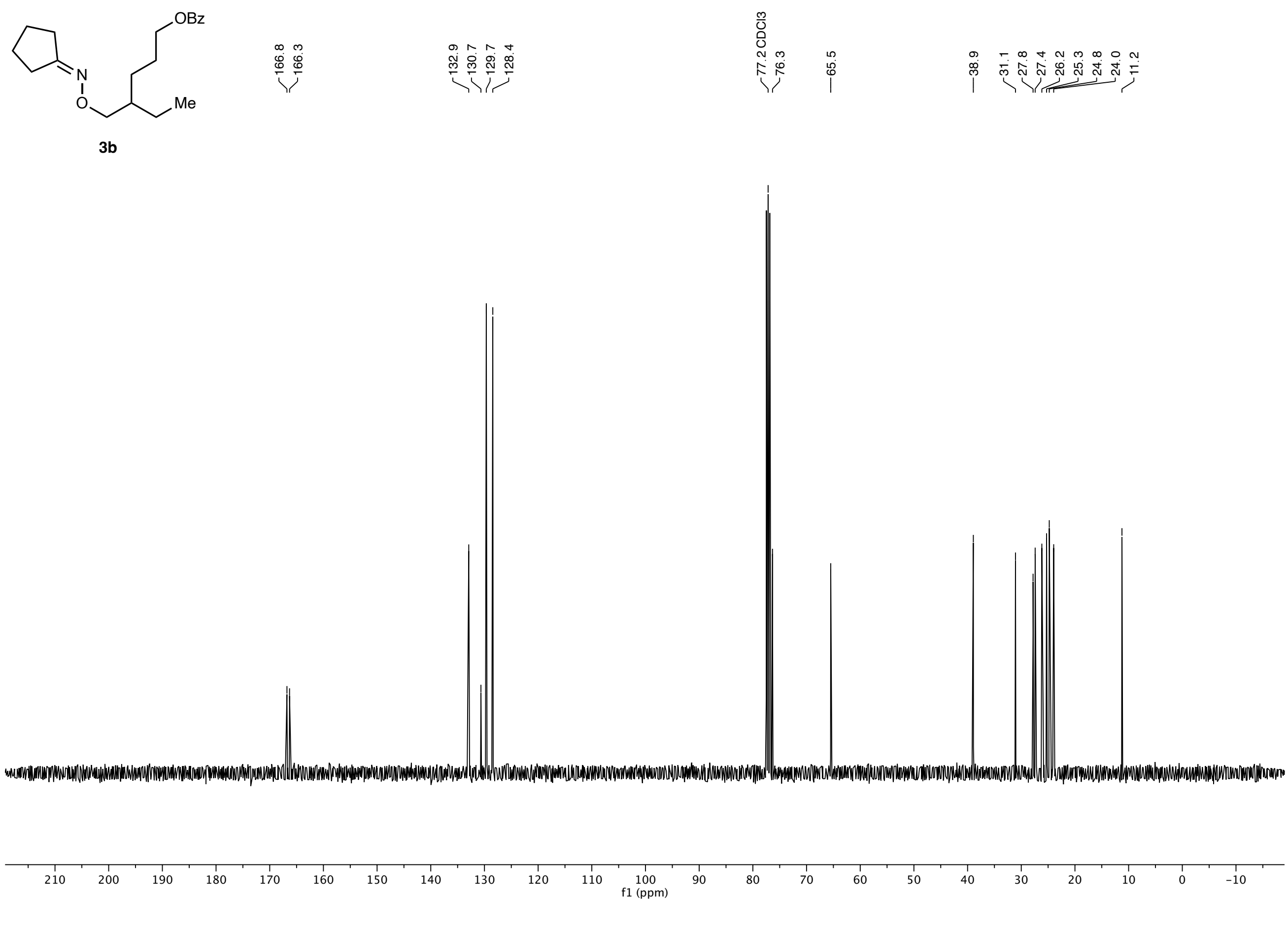
OBz
N
O
Me
3b
166.8 166.3
132.9 130.7 129.7 128.4
77.2 CDCl3 76.3
65.5
38.9
31.1 27.8 27.4 26.2 25.3 24.8 24.0 11.2
210 200 190 180 170 160 150 140 130 120 110 100 90 80 70 60 50 40 30 20 10 0 −10
f1 (ppm)

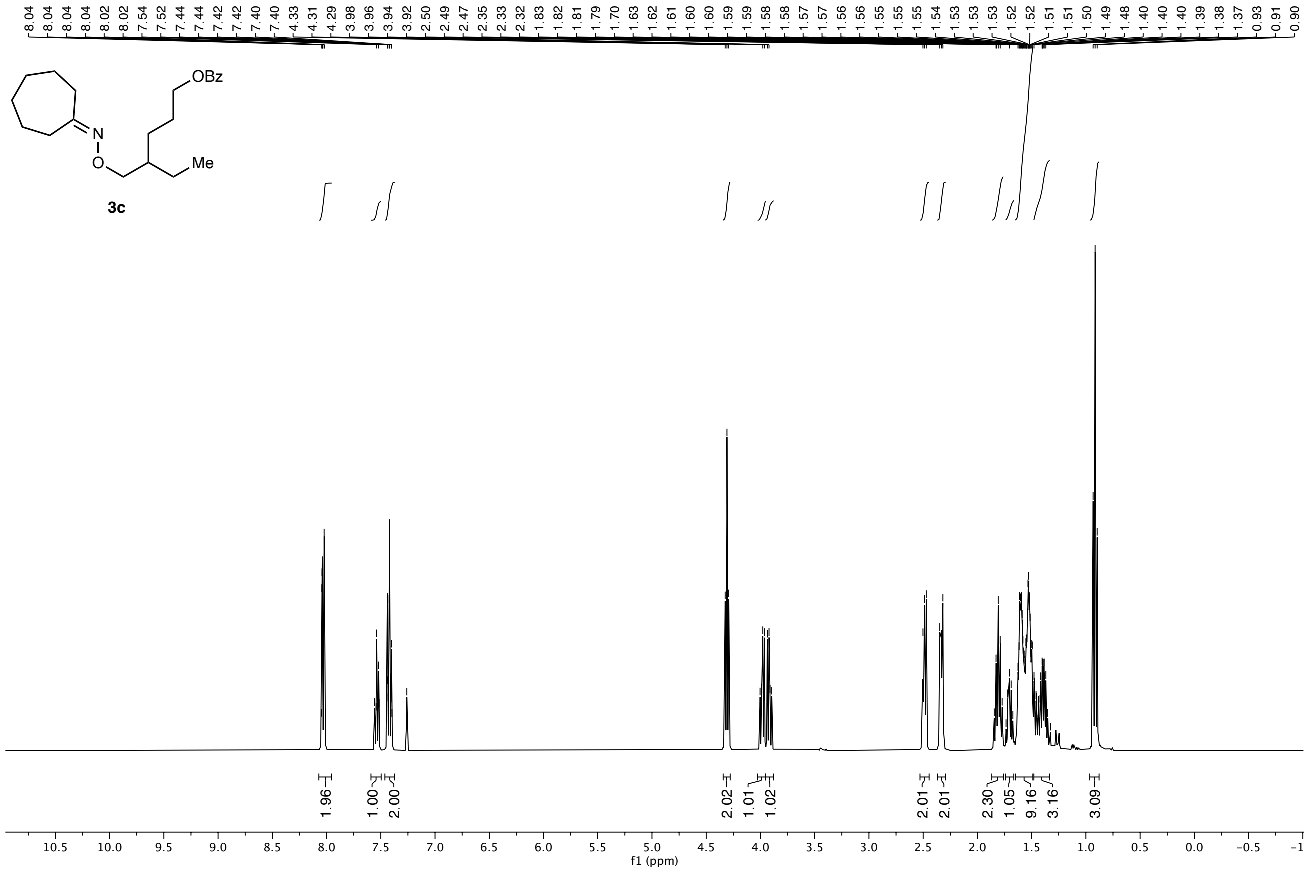
8.04 8.04 8.04 8.04 8.02 7.54 7.52 7.44 7.44 7.42 7.42 7.40 7.40 4.33 4.31 4.29 3.98 3.96 3.94 3.92 2.50 2.49 2.47 2.35 2.33 2.32 1.83 1.82 1.81 1.79 1.70 1.63 1.62 1.61 1.60 1.60 1.59 1.59 1.58 1.58 1.57 1.57 1.56 1.56 1.55 1.55 1.55 1.54 1.53 1.53 1.53 1.53 1.52 1.52 1.51 1.51 1.50 1.49 1.48 1.40 1.40 1.40 1.39 1.38 1.37 0.93 0.91 0.90
OBz
N
O
Me
3c
1.96 1.00 2.00 2.02 1.01 1.02 2.01 2.01 2.30 1.05 9.16 3.16 3.09
10.5 10.0 9.5 9.0 8.5 8.0 7.5 7.0 6.5 6.0 5.5 5.0 4.5 4.0 3.5 3.0 2.5 2.0 1.5 1.0 0.5 0.0 −0.5 −1
f1 (ppm)

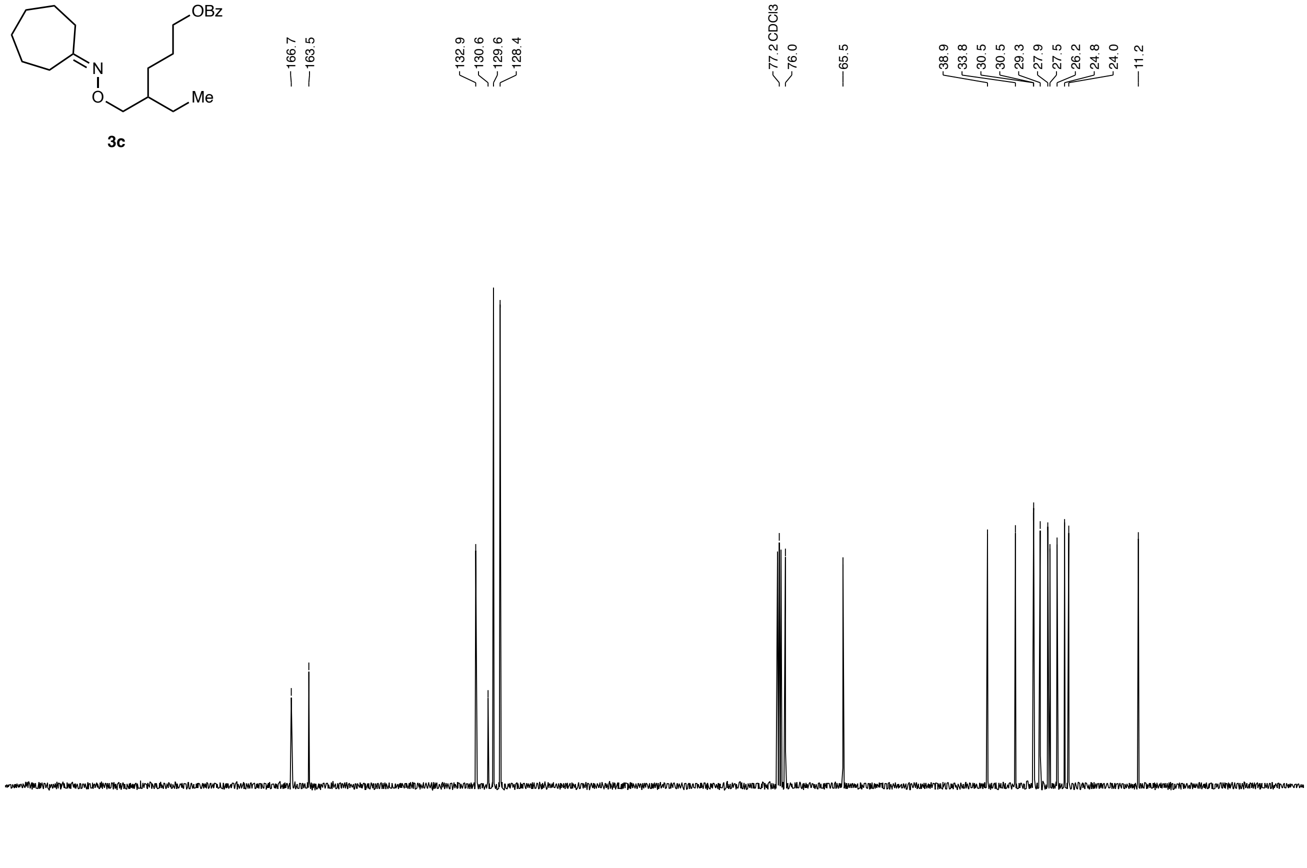
OBz
N
O
Me
3c
166.7 163.5 132.9 130.6 129.6 128.4 77.2 CDCl3 76.0 65.5 38.9 33.8 30.5 30.5 29.3 27.9 27.5 26.2 24.8 24.0 11.2
210 200 190 180 170 160 150 140 130 120 110 100 90 80 70 60 50 40 30 20 10 0 −10
f1 (ppm)

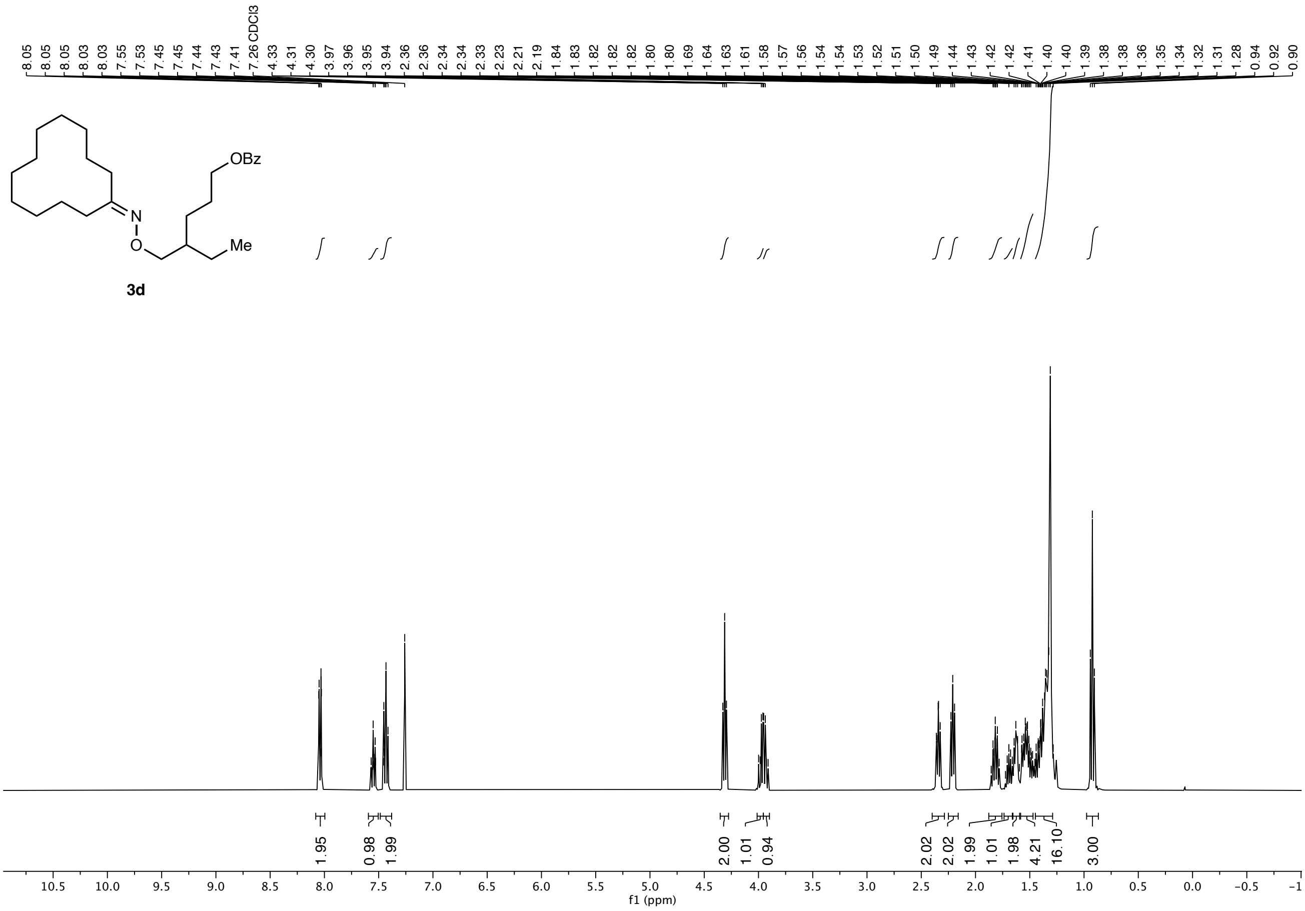
OBz
N
O
Me
8.05 8.05 8.05 8.03 8.03 7.55 7.53 7.45 7.45 7.44 7.43 7.41 7.26 CDCl3 4.33 4.31 4.30 3.97 3.96 3.95 3.94 2.36 2.36 2.34 2.34 2.33 2.23 2.21 2.19 1.84 1.83 1.82 1.82 1.82 1.80 1.80 1.69 1.64 1.63 1.61 1.58 1.57 1.56 1.54 1.54 1.53 1.52 1.51 1.50 1.49 1.44 1.43 1.42 1.42 1.41 1.40 1.40 1.39 1.38 1.38 1.36 1.35 1.34 1.32 1.31 1.28 0.94 0.92 0.90
1.95 0.98 1.99 2.00 1.01 0.94 2.02 2.02 1.99 1.01 1.98 4.21 16.10 3.00
f1 (ppm)

3d

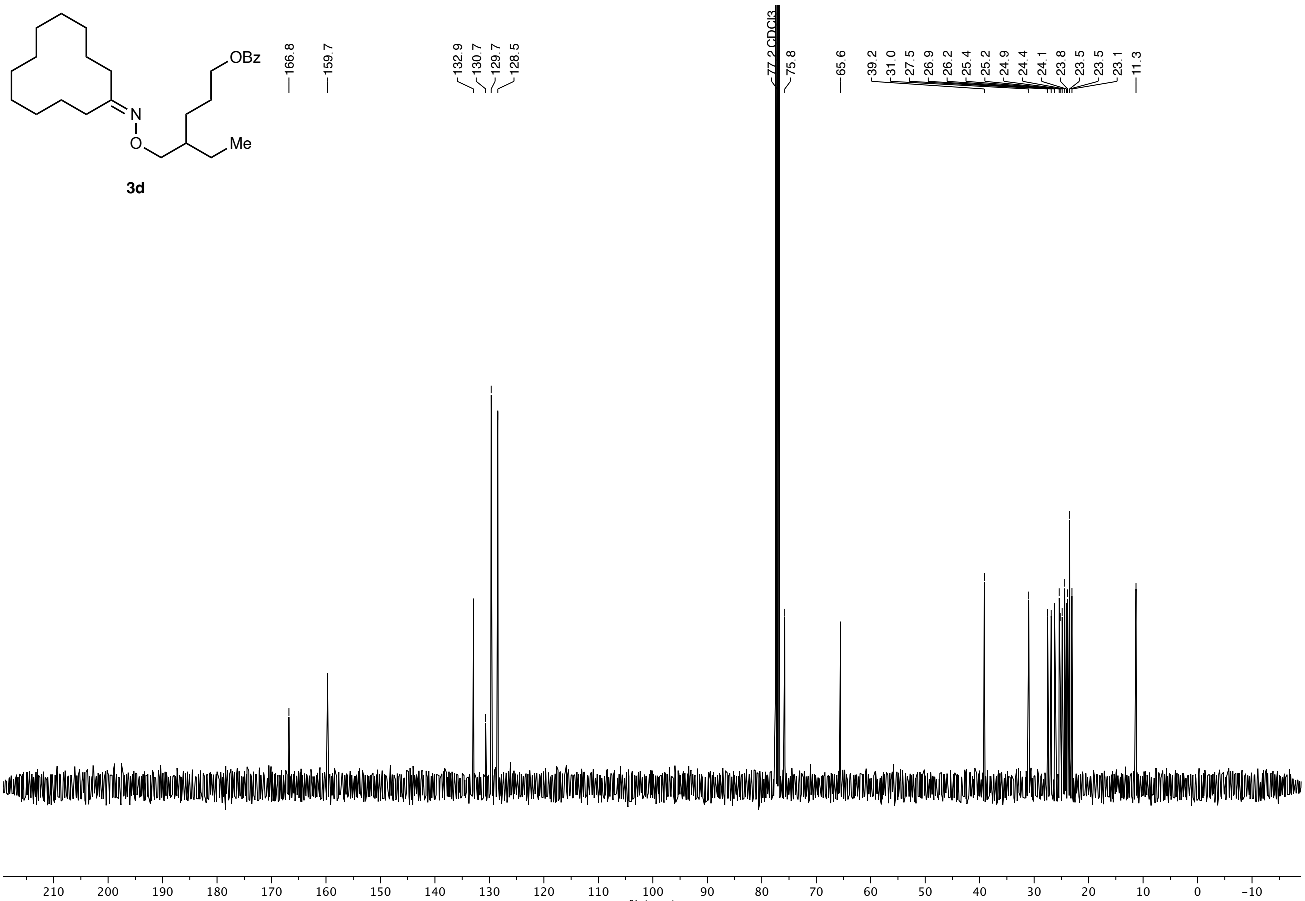
OBz
N
O
Me
166.8 159.7 132.9 130.7 129.7 128.5 77.2 CDCl3 75.8 65.6 39.2 31.0 27.5 26.9 26.2 25.4 25.2 24.9 24.4 24.1 23.8 23.5 23.5 23.1 11.3
f1 (ppm)

3d

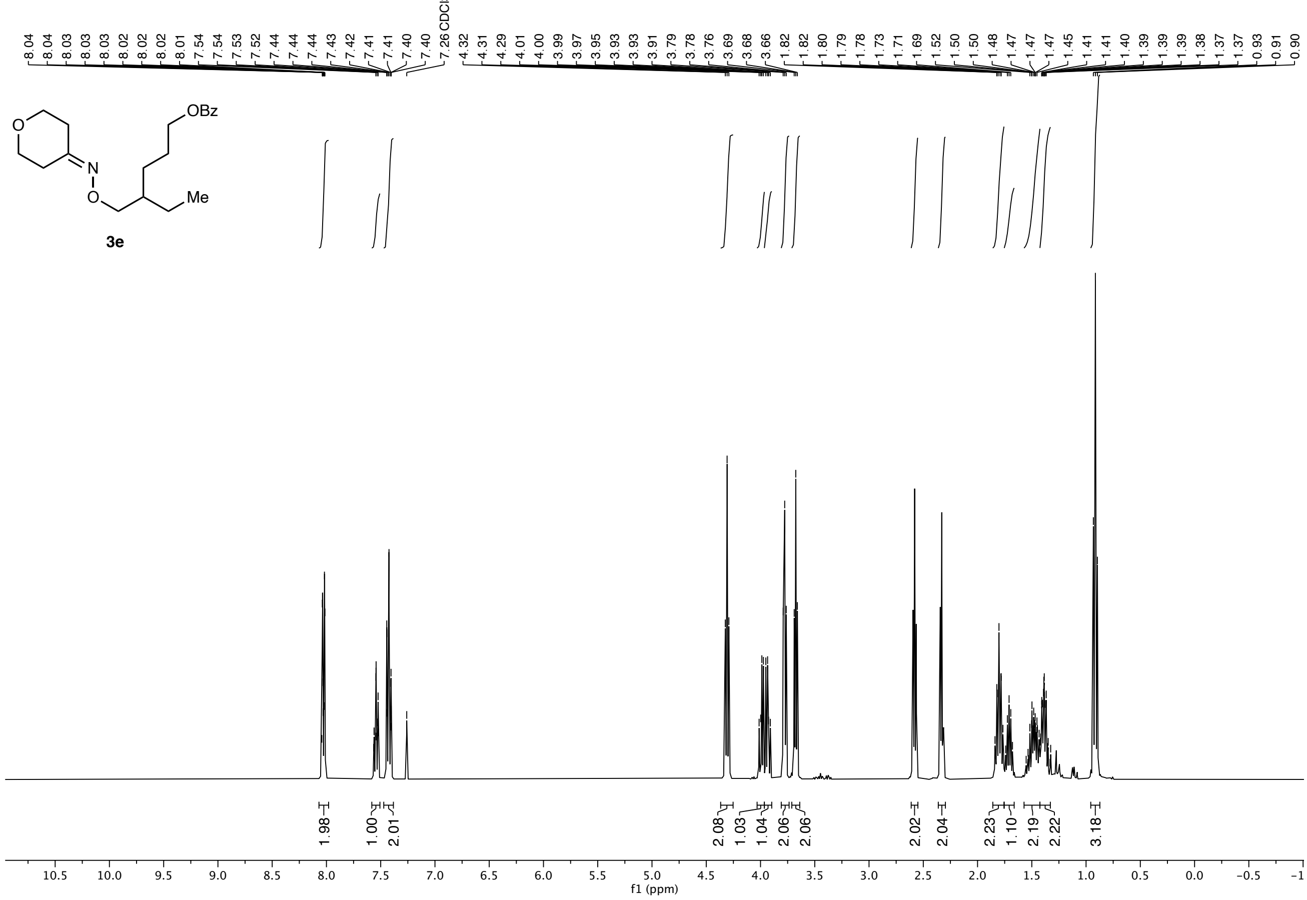
OBz
Me
3e
f1 (ppm)

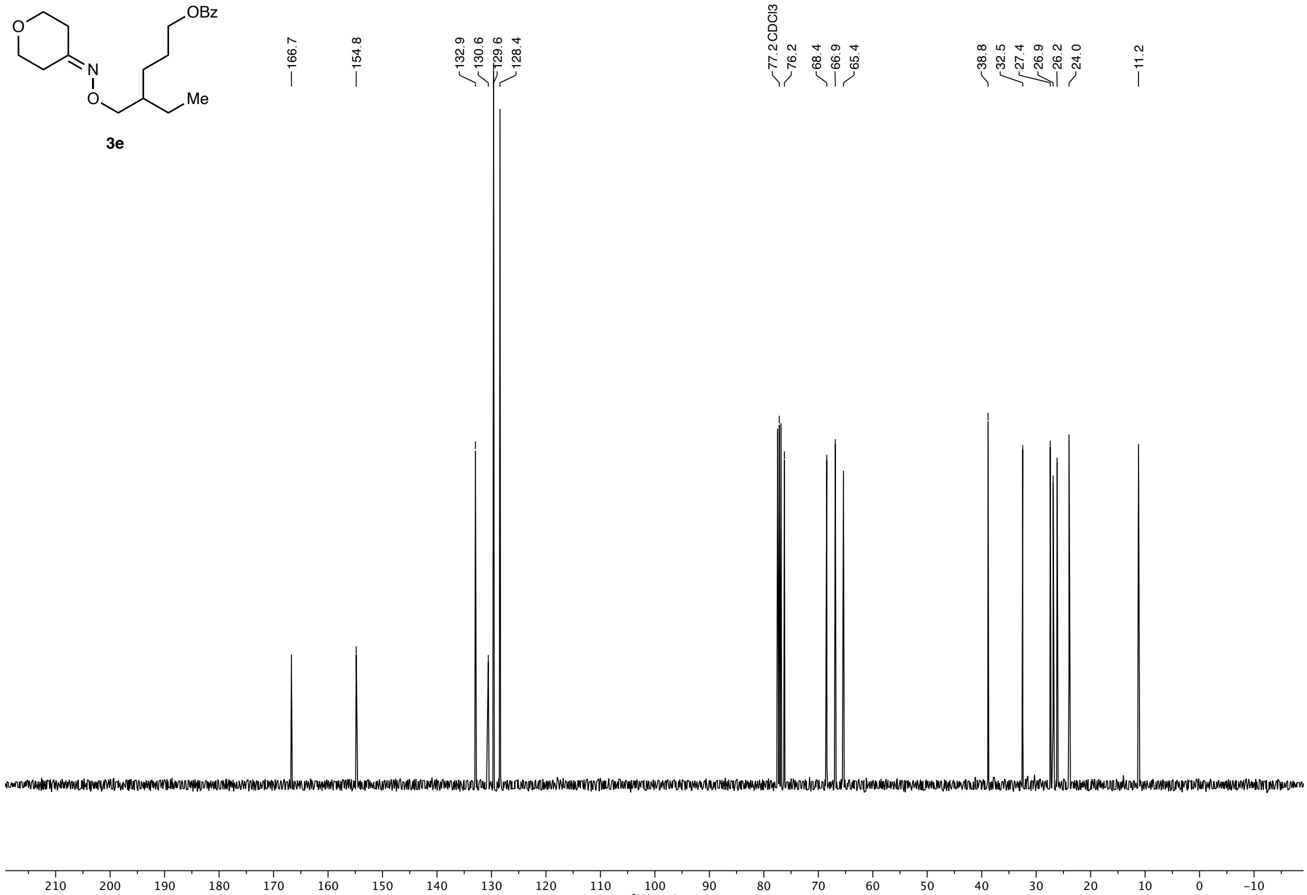
OBz
Me
3e
166.7
154.8
132.9
130.6
129.6
128.4
77.2 CDCl3
76.2
68.4
66.9
65.4
38.8
32.5
27.4
26.9
26.2
24.0
11.2
f1 (ppm)

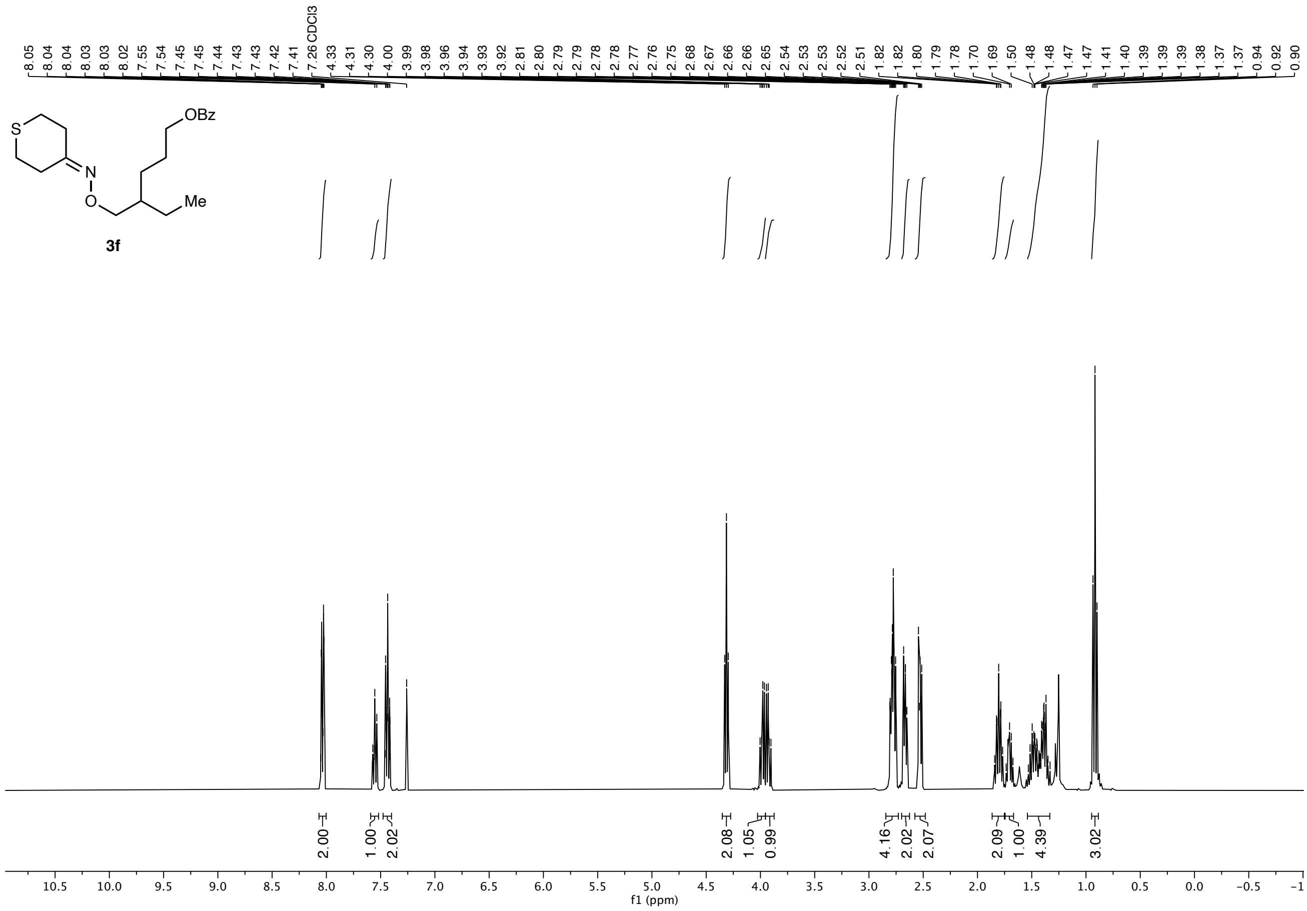
8.05 8.04 8.04 8.03 8.03 8.02 7.55 7.54 7.45 7.45 7.44 7.43 7.43 7.42 7.41 7.26 CDCl3 4.33 4.31 4.30 4.00 3.99 3.98 3.96 3.94 3.93 3.92 2.81 2.80 2.79 2.79 2.78 2.78 2.77 2.76 2.75 2.68 2.67 2.66 2.66 2.65 2.54 2.53 2.53 2.52 2.51 1.82 1.82 1.80 1.79 1.78 1.70 1.69 1.50 1.48 1.48 1.47 1.47 1.41 1.40 1.39 1.39 1.38 1.37 1.37 0.94 0.92 0.90
S
N
O
OBz
Me
3f
2.00 1.00 2.02 2.08 1.05 0.99 4.16 2.02 2.07 2.09 1.00 4.39 3.02
10.5 10.0 9.5 9.0 8.5 8.0 7.5 7.0 6.5 6.0 5.5 5.0 4.5 4.0 3.5 3.0 2.5 2.0 1.5 1.0 0.5 0.0 −0.5 −1
f1 (ppm)

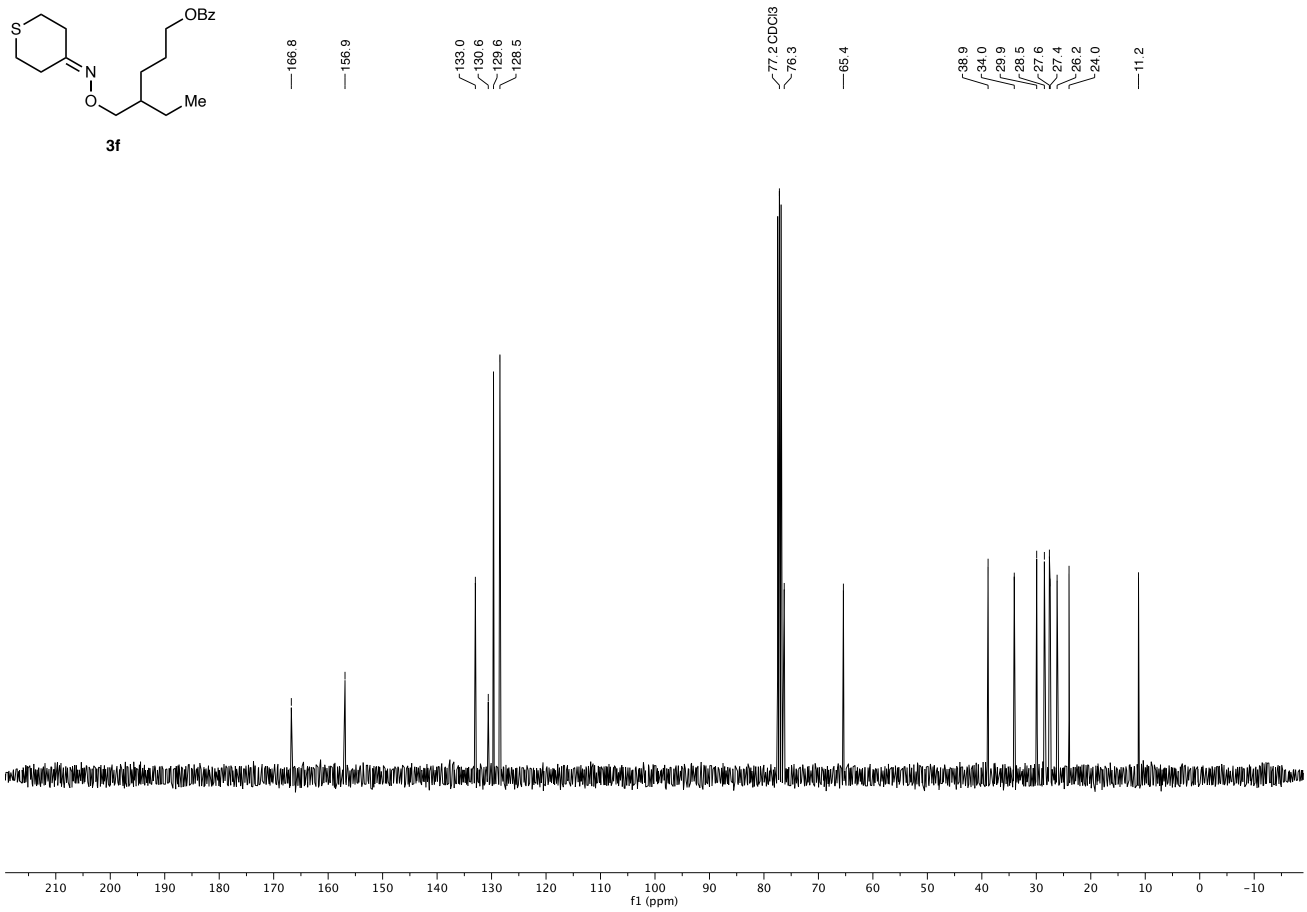
S
N
O
OBz
Me
3f
166.8 156.9 133.0 130.6 129.6 128.5 77.2 CDCl3 76.3 65.4 38.9 34.0 29.9 28.5 27.6 27.4 26.2 24.0 11.2
210 200 190 180 170 160 150 140 130 120 110 100 90 80 70 60 50 40 30 20 10 0 −10
f1 (ppm)

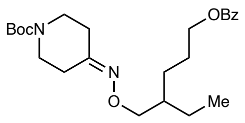
BocN
N
O
OBz
Me

3g

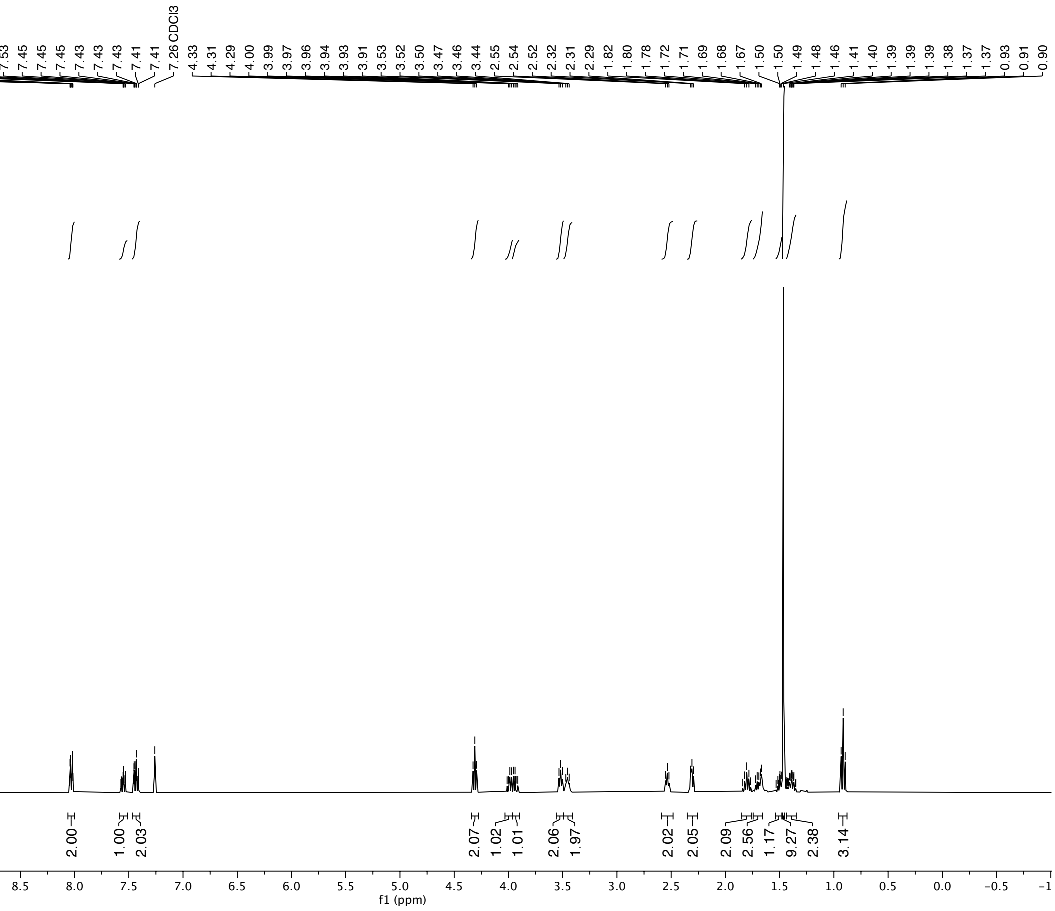
8.04 8.04 8.04 8.03 8.03 8.02 8.02 8.01 7.55 7.55 7.55 7.54 7.53 7.45 7.45 7.45 7.43 7.43 7.43 7.41 7.26 CDCl3 4.33 4.31 4.29 4.00 3.99 3.97 3.96 3.94 3.93 3.91 3.53 3.52 3.50 3.47 3.46 3.44 2.55 2.54 2.52 2.32 2.31 2.29 1.82 1.80 1.78 1.72 1.71 1.69 1.68 1.67 1.50 1.50 1.49 1.48 1.46 1.41 1.40 1.39 1.39 1.39 1.38 1.37 0.93 0.91 0.90
2.00 1.00 2.03 2.07 1.02 1.01 2.06 1.97 2.02 2.05 2.09 2.56 1.17 9.27 2.38 3.14
10.5 10.0 9.5 9.0 8.5 8.0 7.5 7.0 6.5 6.0 5.5 5.0 4.5 4.0 3.5 3.0 2.5 2.0 1.5 1.0 0.5 0.0 −0.5 −1
f1 (ppm)

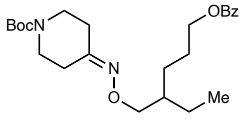
BocN
N
O
OBz
Me

3g

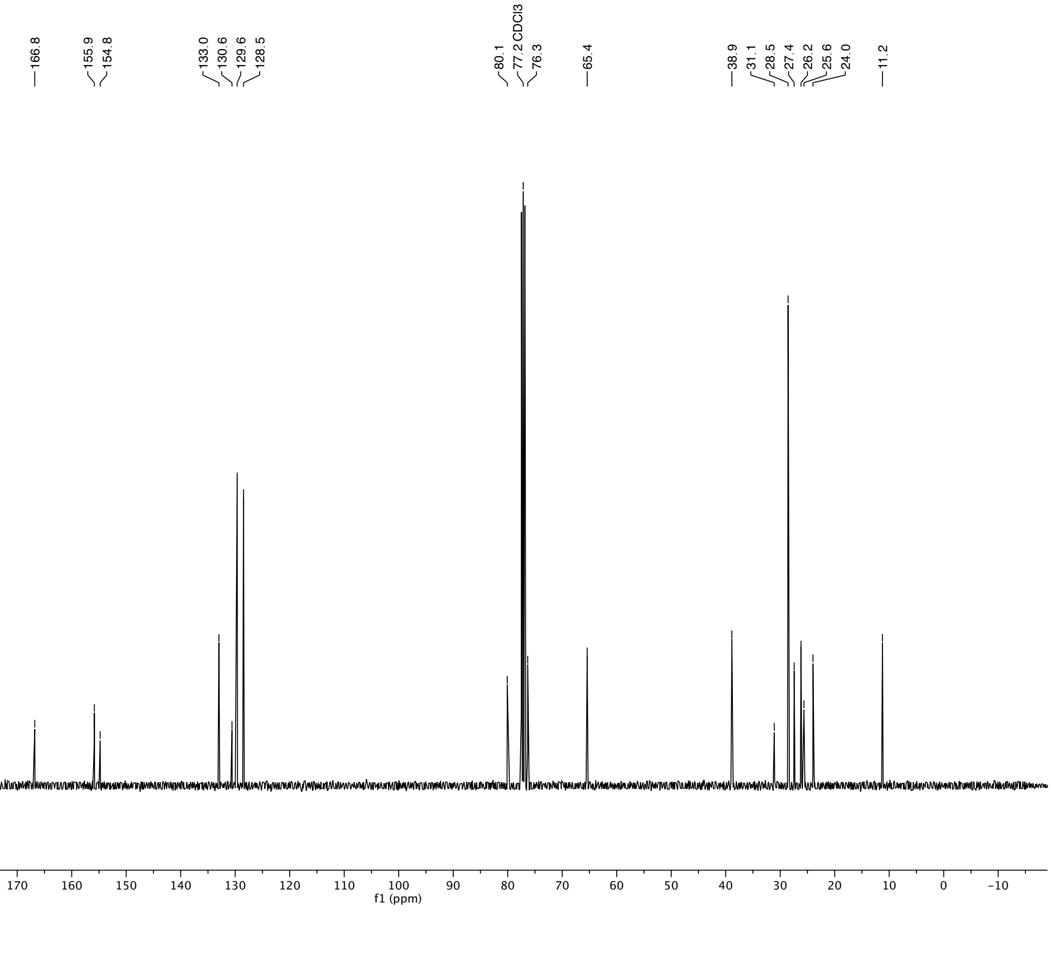
166.8 155.9 154.8 133.0 130.6 129.6 128.5 80.1 77.2 CDCl3 76.3 65.4 38.9 31.1 28.5 27.4 26.2 25.6 24.0 11.2
210 200 190 180 170 160 150 140 130 120 110 100 90 80 70 60 50 40 30 20 10 0 −10
f1 (ppm)

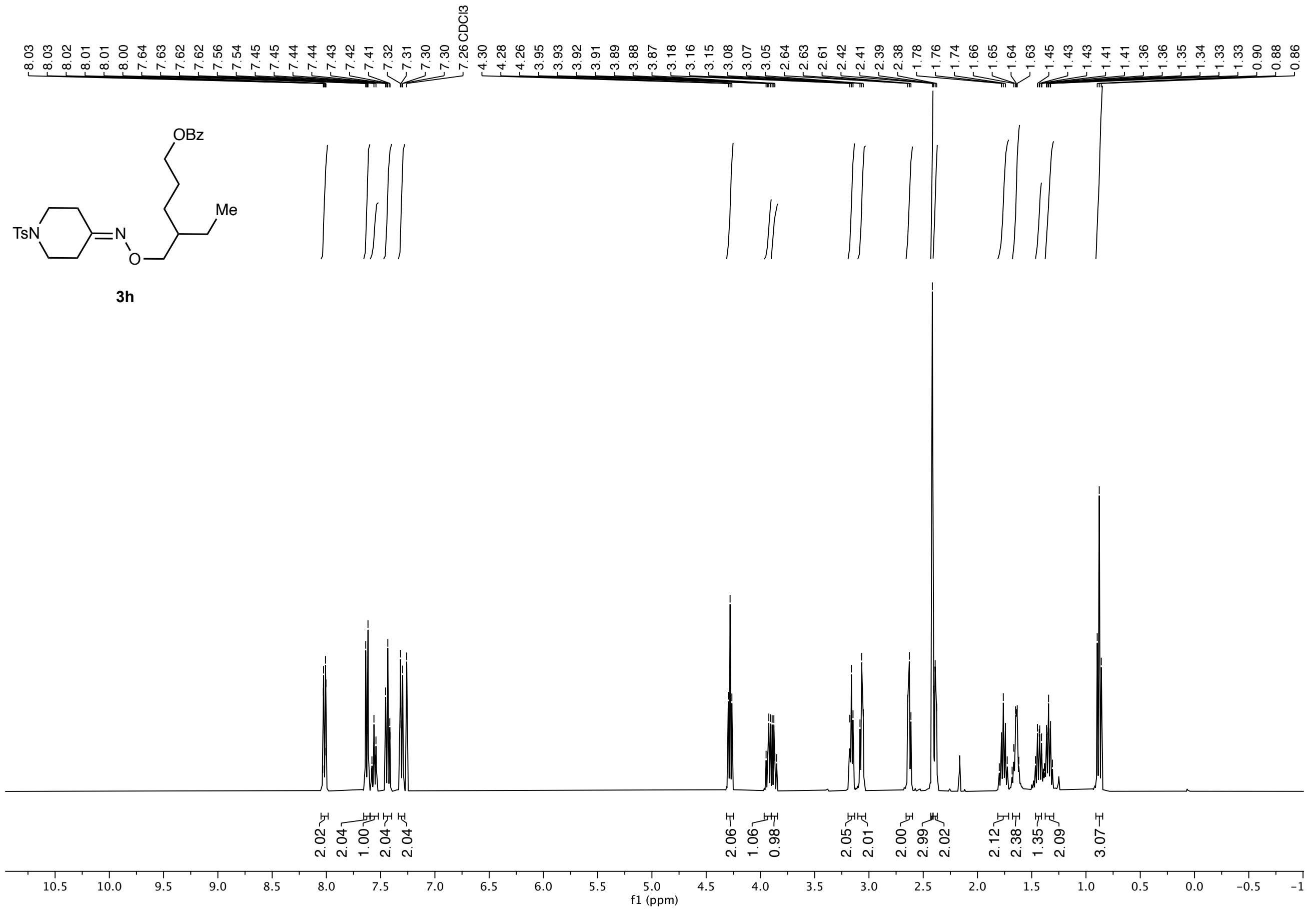
8.03 8.03 8.02 8.01 8.01 8.00 7.64 7.63 7.62 7.62 7.56 7.54 7.45 7.45 7.44 7.44 7.43 7.42 7.41 7.32 7.31 7.30 7.30 7.26 CDCl3 4.30 4.28 4.26 3.95 3.93 3.92 3.91 3.89 3.88 3.87 3.18 3.16 3.15 3.08 3.07 3.05 2.64 2.63 2.61 2.42 2.41 2.39 2.38 1.78 1.76 1.74 1.66 1.65 1.64 1.63 1.45 1.43 1.43 1.41 1.41 1.36 1.36 1.35 1.34 1.33 0.90 0.88 0.86
OBz
Me
TsN
N
O
3h
2.02 2.04 1.00 2.04 2.04 2.06 1.06 0.98 2.05 2.01 2.00 2.99 2.02 2.12 2.38 1.35 2.09 3.07
10.5 10.0 9.5 9.0 8.5 8.0 7.5 7.0 6.5 6.0 5.5 5.0 4.5 4.0 3.5 3.0 2.5 2.0 1.5 1.0 0.5 0.0 −0.5 −1
f1 (ppm)

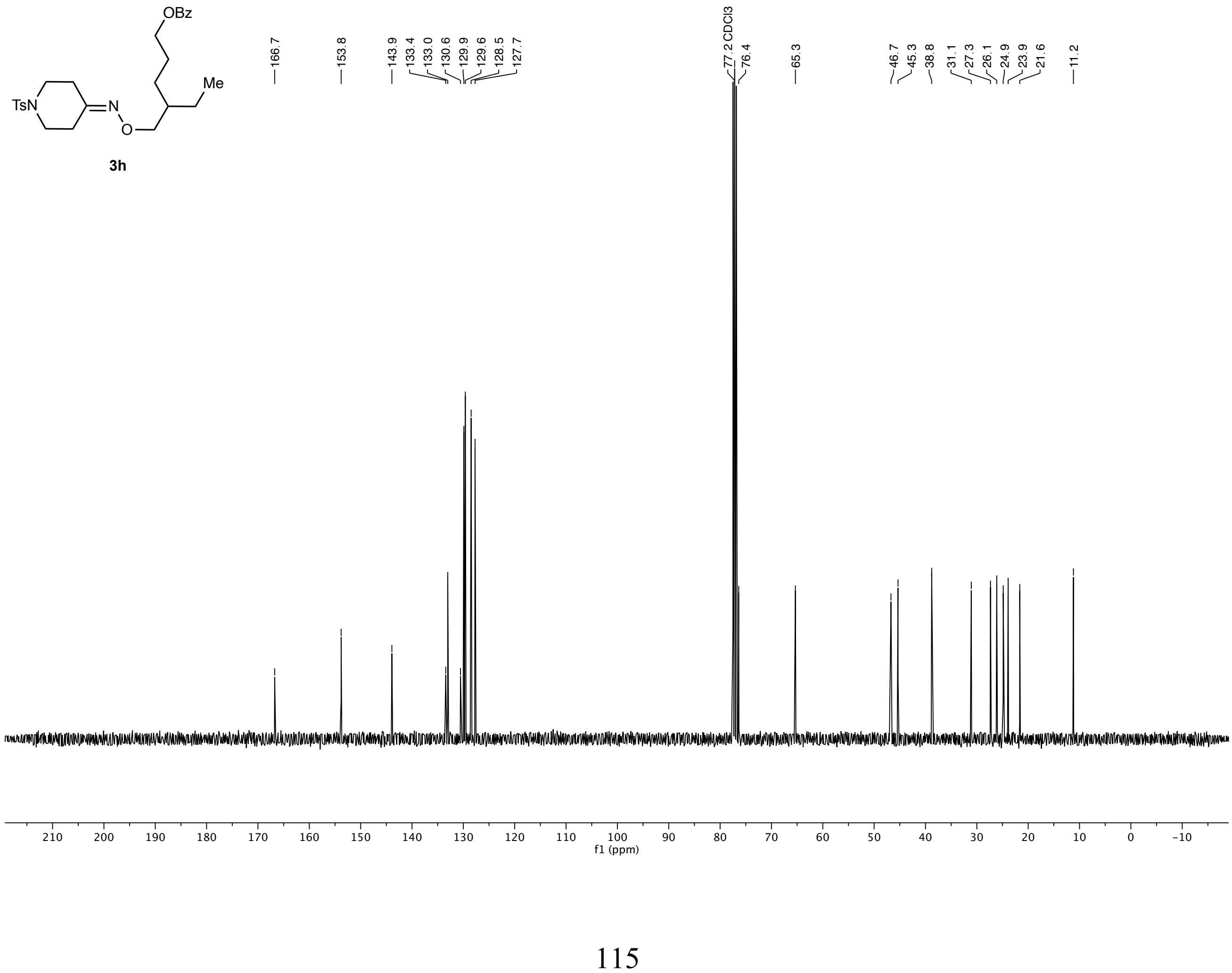
OBz
Me
TsN
N
O
3h
166.7 153.8 143.9 133.4 133.0 130.6 129.9 129.6 128.5 127.7 77.2 CDCl3 76.4 65.3 46.7 45.3 38.8 31.1 27.3 26.1 24.9 23.9 21.6 11.2
210 200 190 180 170 160 150 140 130 120 110 100 90 80 70 60 50 40 30 20 10 0 −10
f1 (ppm)

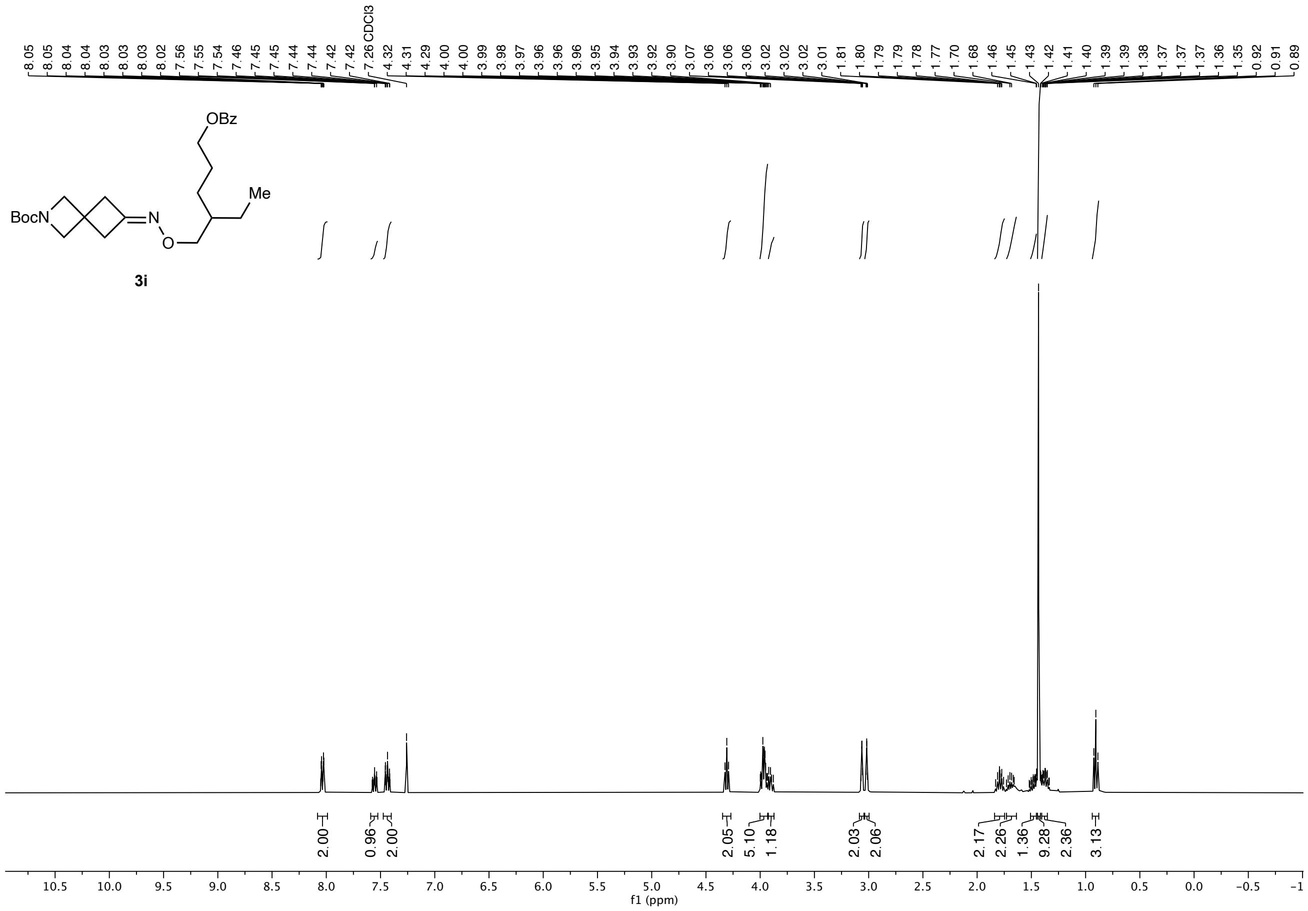
8.05 8.05 8.04 8.04 8.03 8.03 8.03 8.02 7.56 7.55 7.54 7.46 7.45 7.45 7.44 7.44 7.42 7.42 7.26 CDCl3 4.32 4.31 4.29 4.00 4.00 3.99 3.98 3.97 3.96 3.96 3.95 3.94 3.93 3.92 3.90 3.07 3.06 3.06 3.06 3.02 3.02 3.02 3.01 1.81 1.80 1.79 1.79 1.78 1.77 1.70 1.68 1.46 1.45 1.43 1.42 1.41 1.40 1.39 1.39 1.38 1.37 1.37 1.37 1.36 1.35 0.92 0.91 0.89
OBz
Me
BocN
N
O
3i
2.00 0.96 2.00 2.05 5.10 1.18 2.03 2.06 2.17 2.26 1.36 9.28 2.36 3.13
f1 (ppm)

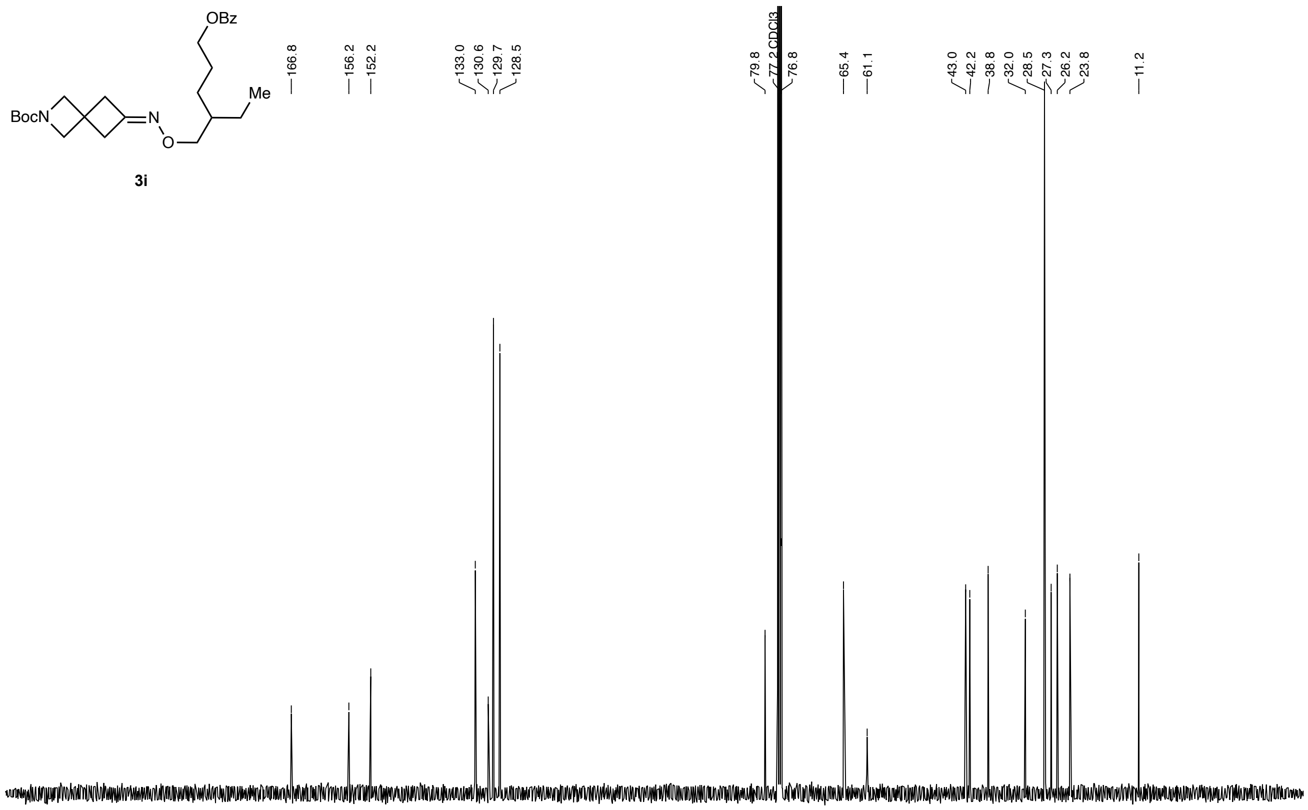
166.8 156.2 152.2 133.0 130.6 129.7 128.5 79.8 77.2 CDCl3 76.8 65.4 61.1 43.0 42.2 38.8 32.0 28.5 27.3 26.2 23.8 11.2
OBz
Me
BocN
N
O
3i
f1 (ppm)

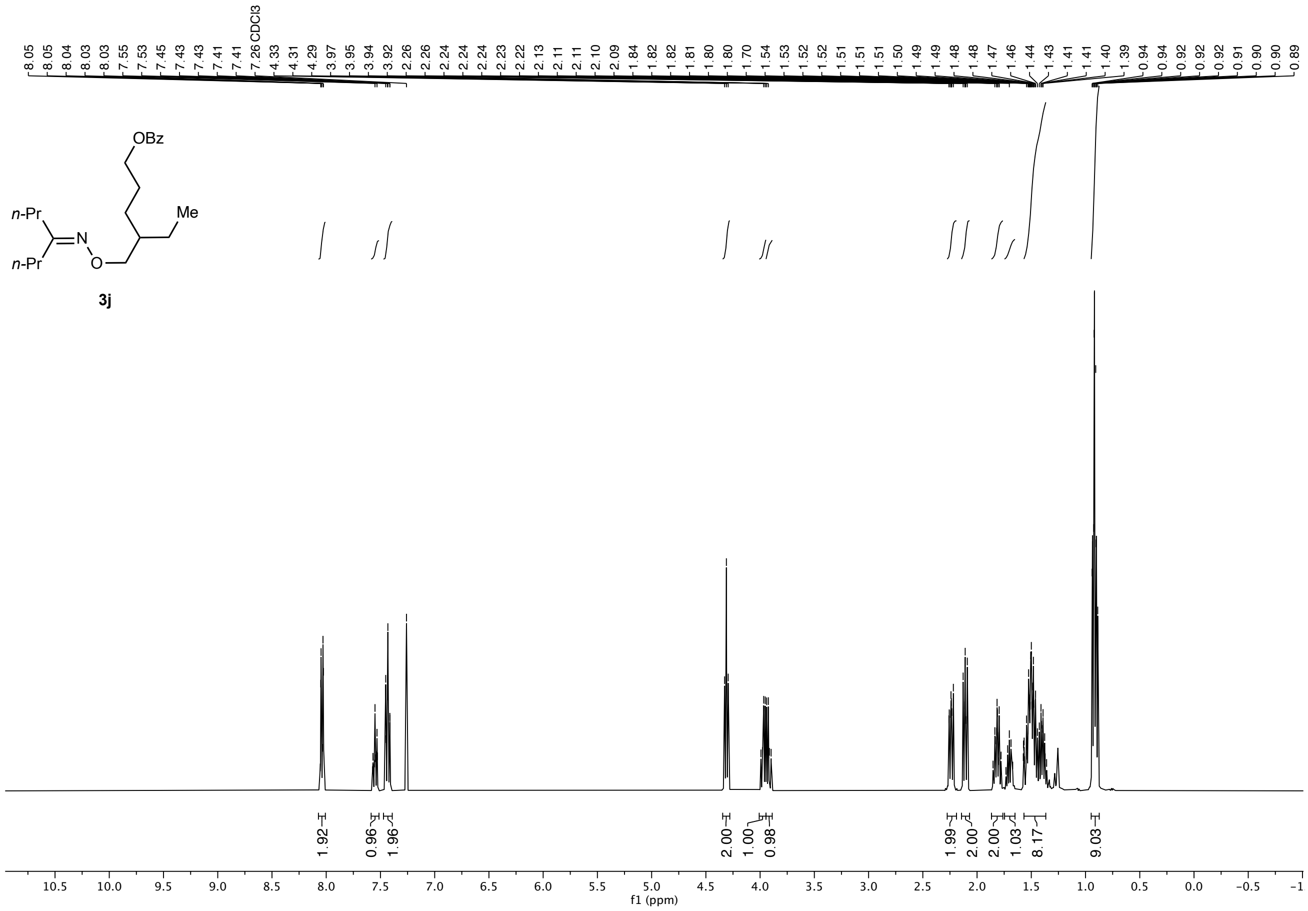
8.05 8.05 8.04 8.03 8.03 7.55 7.53 7.45 7.43 7.43 7.41 7.41 7.26 CDCl3 4.33 4.31 4.29 3.97 3.95 3.94 3.92 2.26 2.26 2.24 2.24 2.24 2.23 2.22 2.13 2.11 2.11 2.10 2.09 1.84 1.82 1.82 1.81 1.80 1.80 1.70 1.54 1.53 1.52 1.52 1.51 1.51 1.51 1.50 1.49 1.49 1.48 1.48 1.47 1.46 1.44 1.43 1.41 1.41 1.40 1.39 0.94 0.94 0.92 0.92 0.92 0.91 0.90 0.90 0.89
OBz
n-Pr
N
Me
n-Pr
O
3j
1.92
0.96
1.96
2.00
1.00
0.98
1.99
2.00
2.00
1.03
8.17
9.03
f1 (ppm)

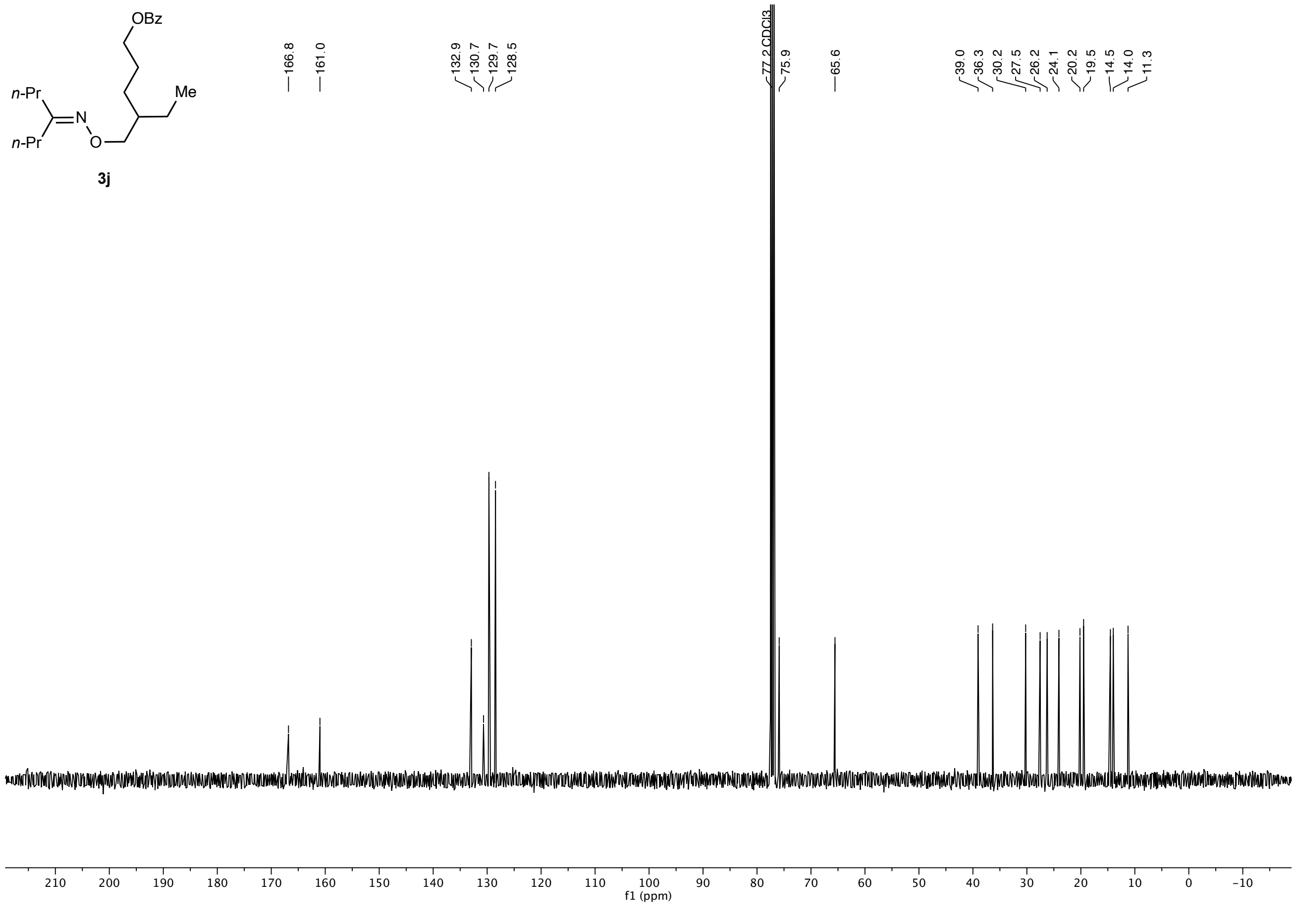
OBz
n-Pr
N
Me
n-Pr
O
3j
166.8
161.0
132.9 130.7 129.7 128.5
77.2 CDCl3
75.9
65.6
39.0 36.3 30.2 27.5 26.2 24.1 20.2 19.5 14.5 14.0 11.3
f1 (ppm)

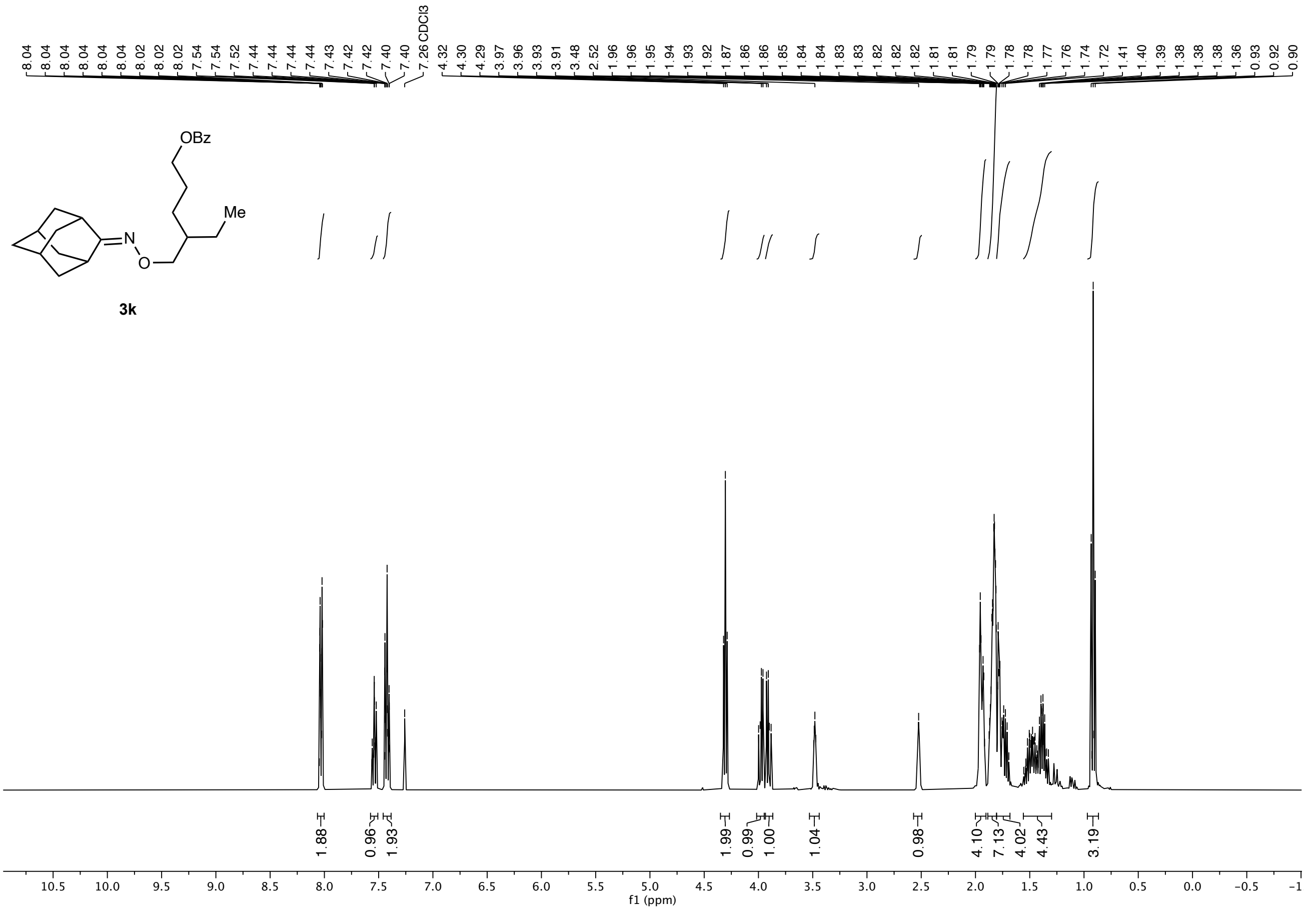
8.04 8.04 8.04 8.04 8.04 8.02 8.02 8.02 7.54 7.54 7.52 7.44 7.44 7.44 7.43 7.42 7.42 7.40
7.26 CDCl3
4.32 4.30 4.29 3.97 3.96 3.93 3.91 3.48 2.52 1.96 1.96 1.95 1.94 1.93 1.92 1.87 1.86 1.86 1.85 1.84 1.84 1.83 1.83 1.82 1.82 1.82 1.81 1.81 1.79 1.79 1.78 1.78 1.77 1.76 1.74 1.72 1.41 1.40 1.39 1.38 1.38 1.38 1.36 0.93 0.92 0.90
OBz
Me
N
O
1.88
0.96
1.93
1.99
0.99
1.00
1.04
0.98
4.10
7.13
4.02
4.43
3.19
f1 (ppm)
3k

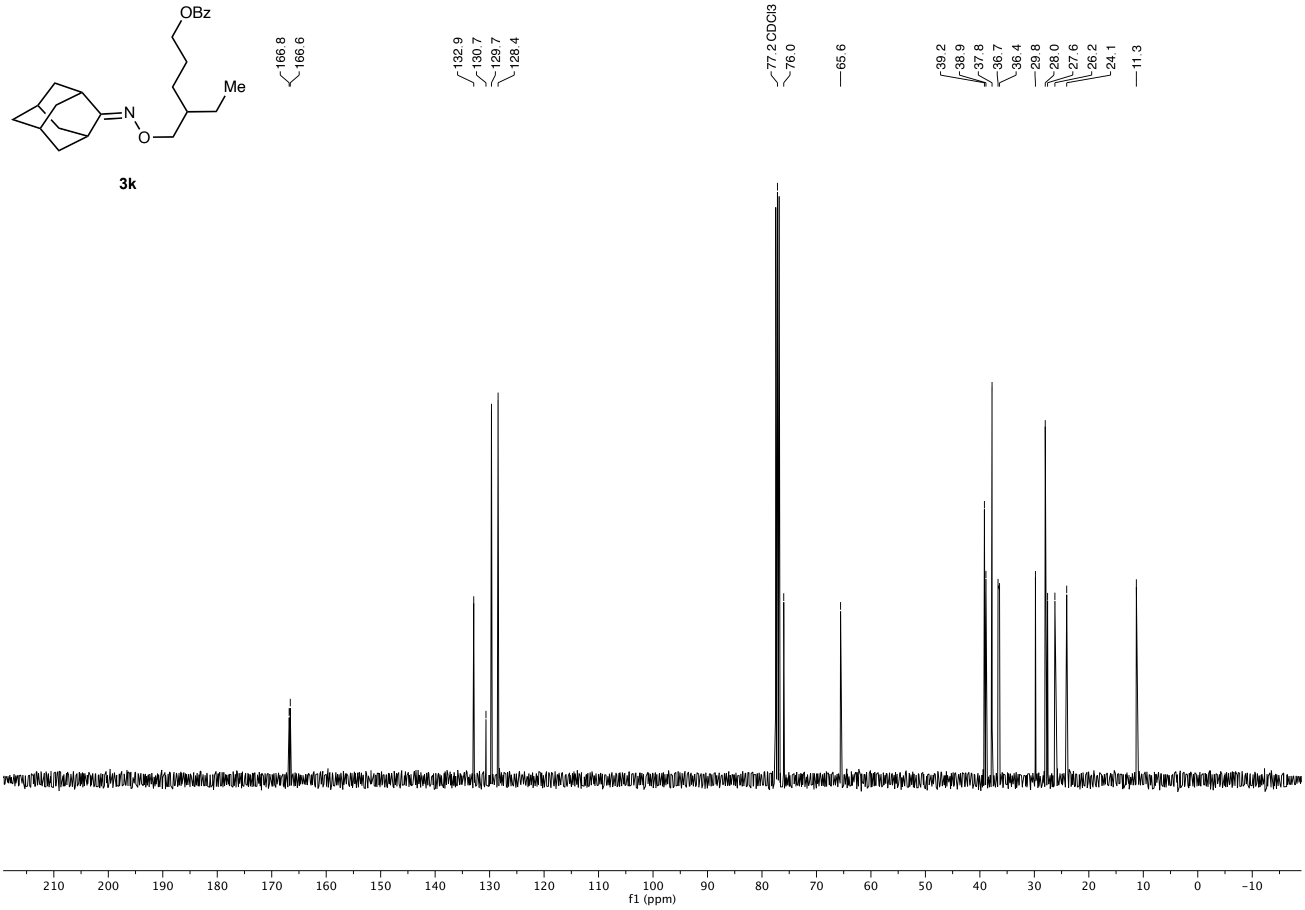
166.8
166.6
132.9
130.7
129.7
128.4
77.2 CDCl3
76.0
65.6
39.2 38.9 37.8 36.7 36.4 29.8 28.0 27.6 26.2 24.1
11.3
OBz
Me
N
O
f1 (ppm)
3k

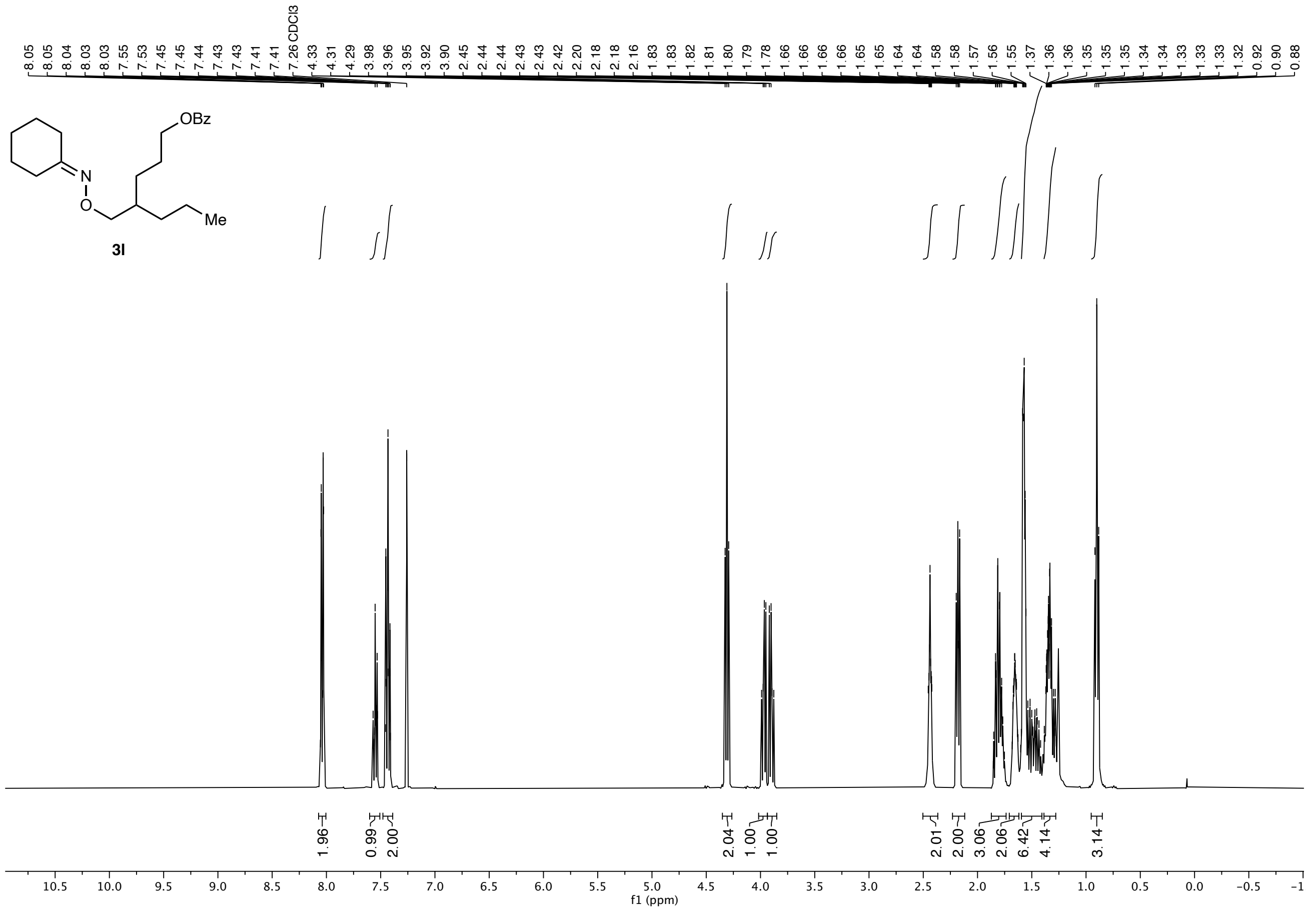
OBz
N
O
Me
3l
f1 (ppm)

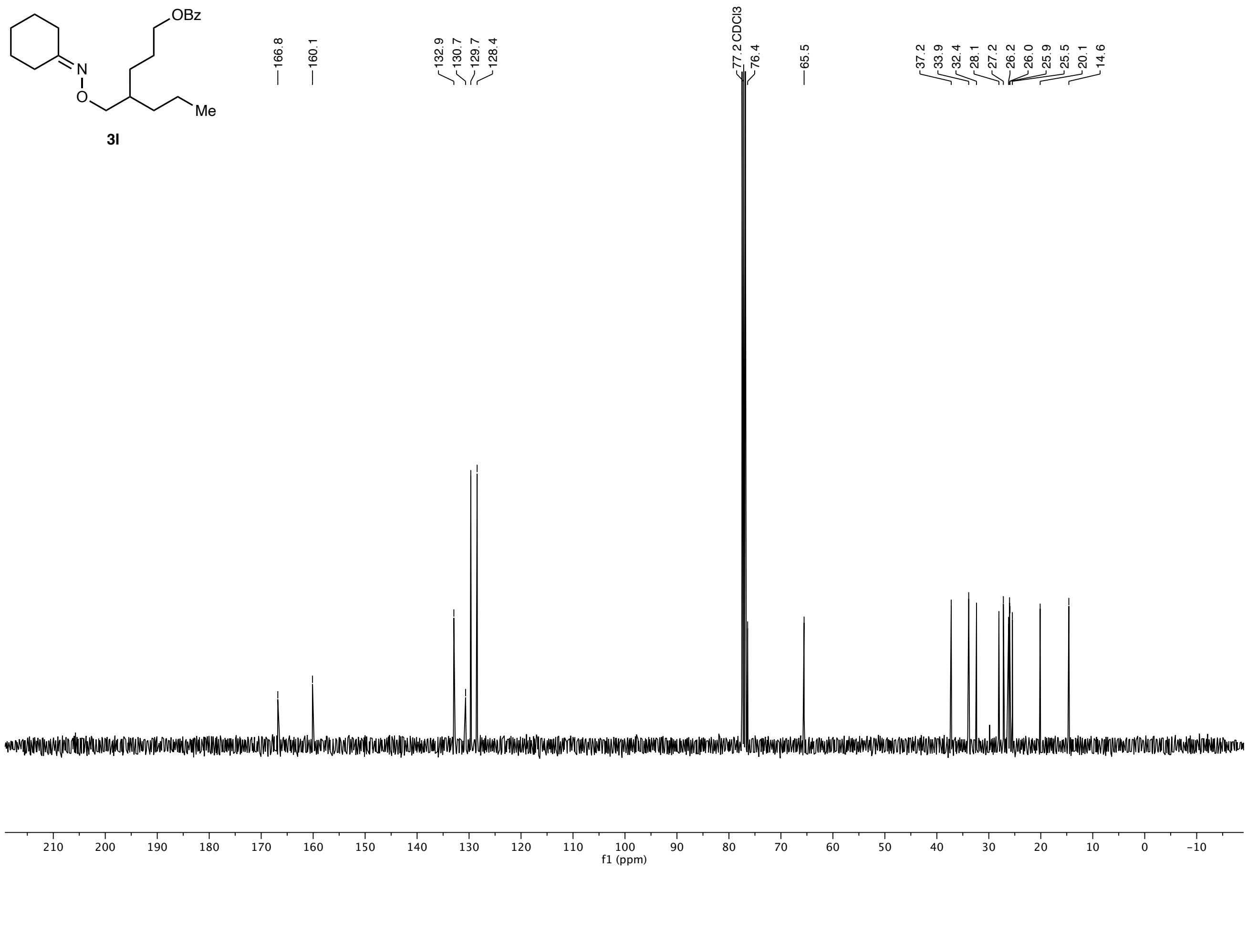
OBz
N
O
Me
3l
f1 (ppm)

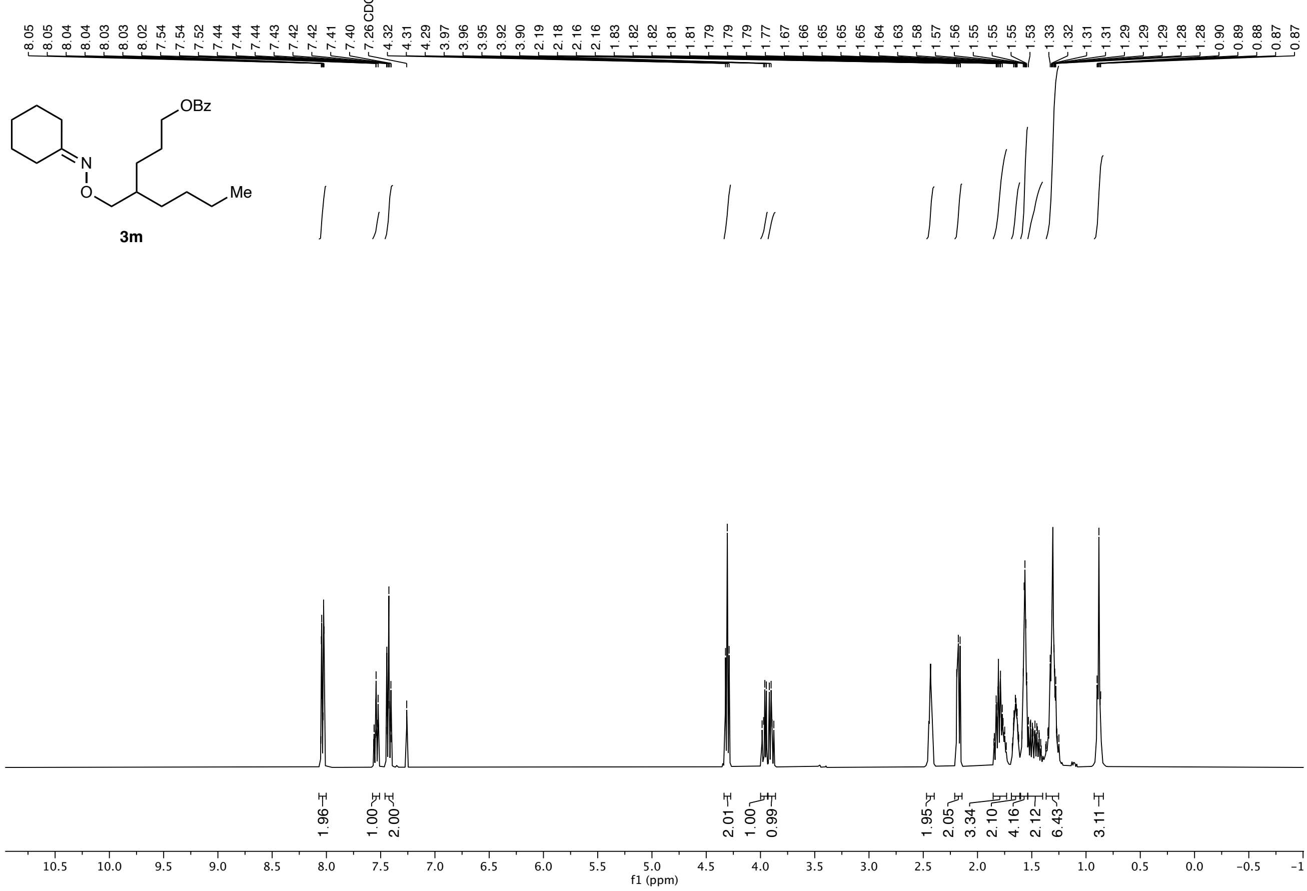
8.05 8.05 8.04 8.04 8.03 8.03 8.02 7.54 7.54 7.52 7.44 7.44 7.44 7.43 7.42 7.42 7.41 7.40 7.26 CDCl3 4.32 4.31 4.29 3.97 3.96 3.95 3.92 3.90 2.19 2.18 2.16 2.16 1.83 1.82 1.82 1.81 1.81 1.79 1.79 1.77 1.67 1.66 1.65 1.65 1.65 1.64 1.63 1.58 1.57 1.56 1.55 1.55 1.55 1.53 1.33 1.32 1.31 1.29 1.29 1.29 1.28 1.28 0.90 0.89 0.88 0.87 0.87
OBz
N
O
Me
3m
1.96 1.00 2.00 2.01 1.00 0.99 1.95 2.05 3.34 2.10 4.16 2.12 6.43 3.11
f1 (ppm)

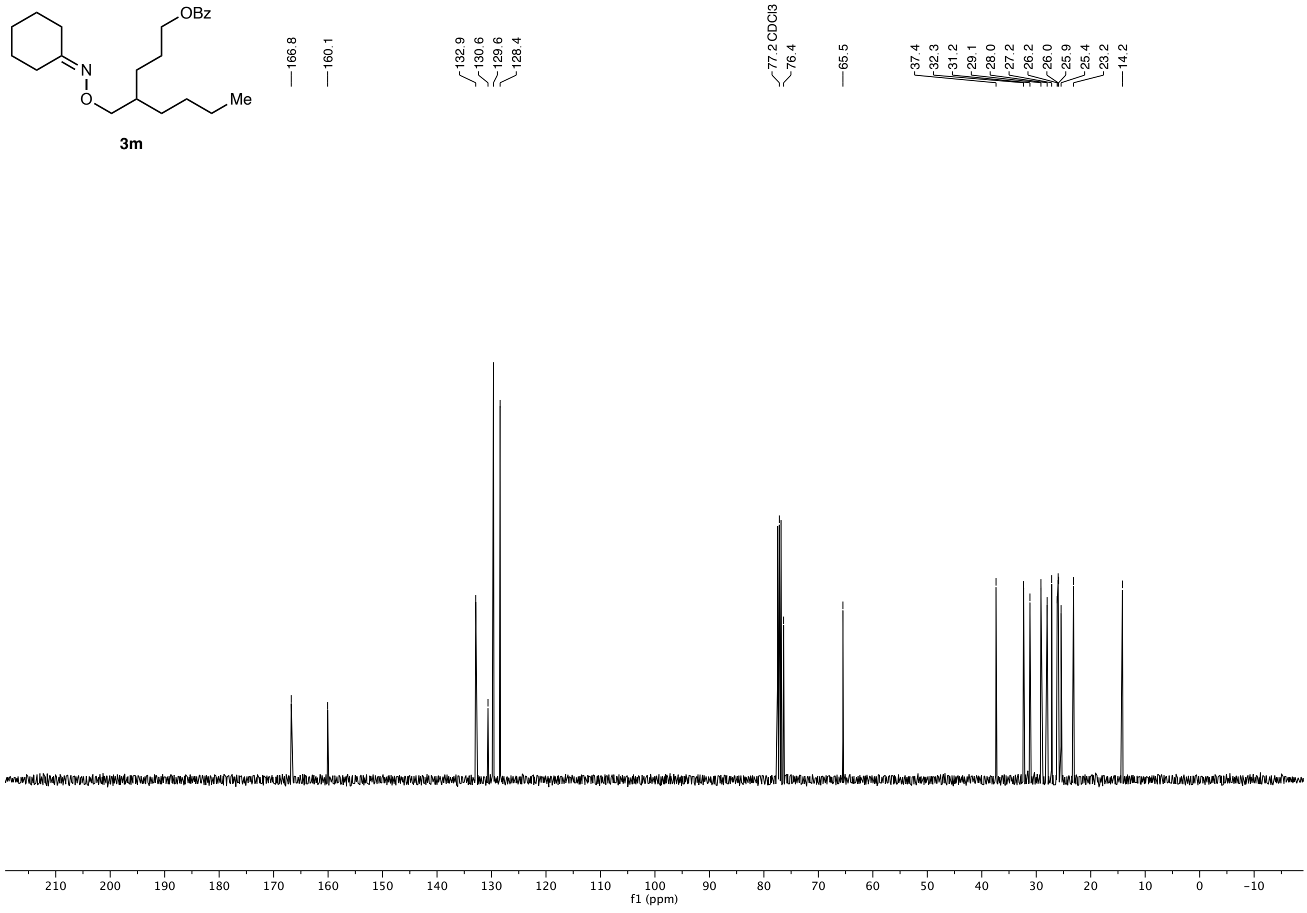
OBz
N
O
Me
3m
166.8 160.1 132.9 130.6 129.6 128.4 77.2 CDCl3 76.4 65.5 37.4 32.3 31.2 29.1 28.0 27.2 26.2 26.0 25.9 25.4 23.2 14.2
f1 (ppm)

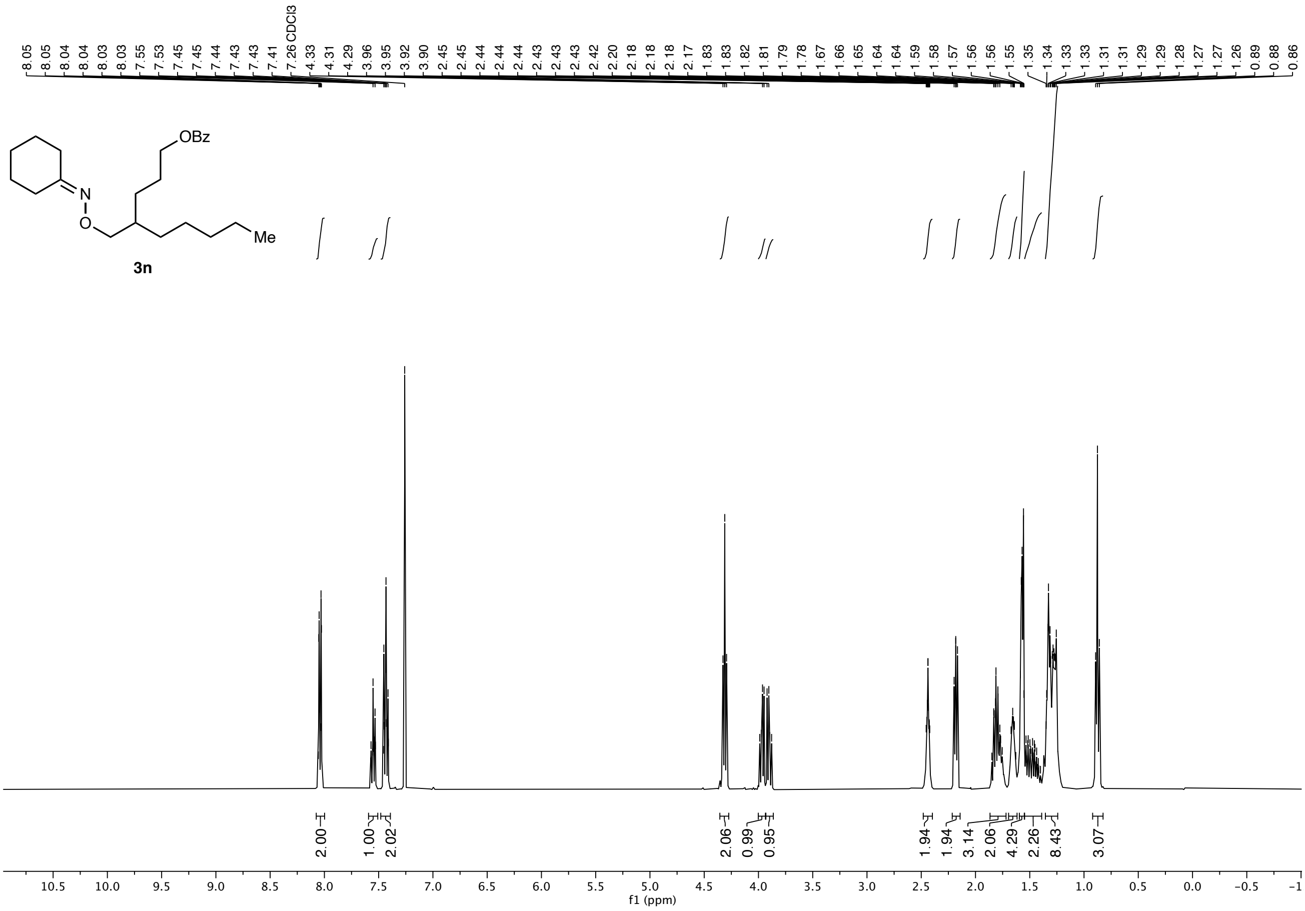
8.05 8.05 8.04 8.04 8.03 8.03 7.55 7.53 7.45 7.45 7.44 7.43 7.43 7.41 7.26 CDCl3 4.33 4.31 4.29 3.96 3.95 3.92 3.90 2.45 2.45 2.44 2.44 2.44 2.43 2.43 2.43 2.42 2.20 2.18 2.18 2.18 2.17 1.83 1.83 1.82 1.81 1.79 1.78 1.67 1.66 1.65 1.64 1.59 1.58 1.57 1.56 1.56 1.55 1.35 1.34 1.33 1.33 1.31 1.31 1.29 1.29 1.28 1.27 1.26 0.89 0.88 0.86
OBz
N
O
Me
3n
2.00 1.00 2.02 2.06 0.99 0.95 1.94 1.94 3.14 2.06 4.29 2.26 8.43 3.07
f1 (ppm)

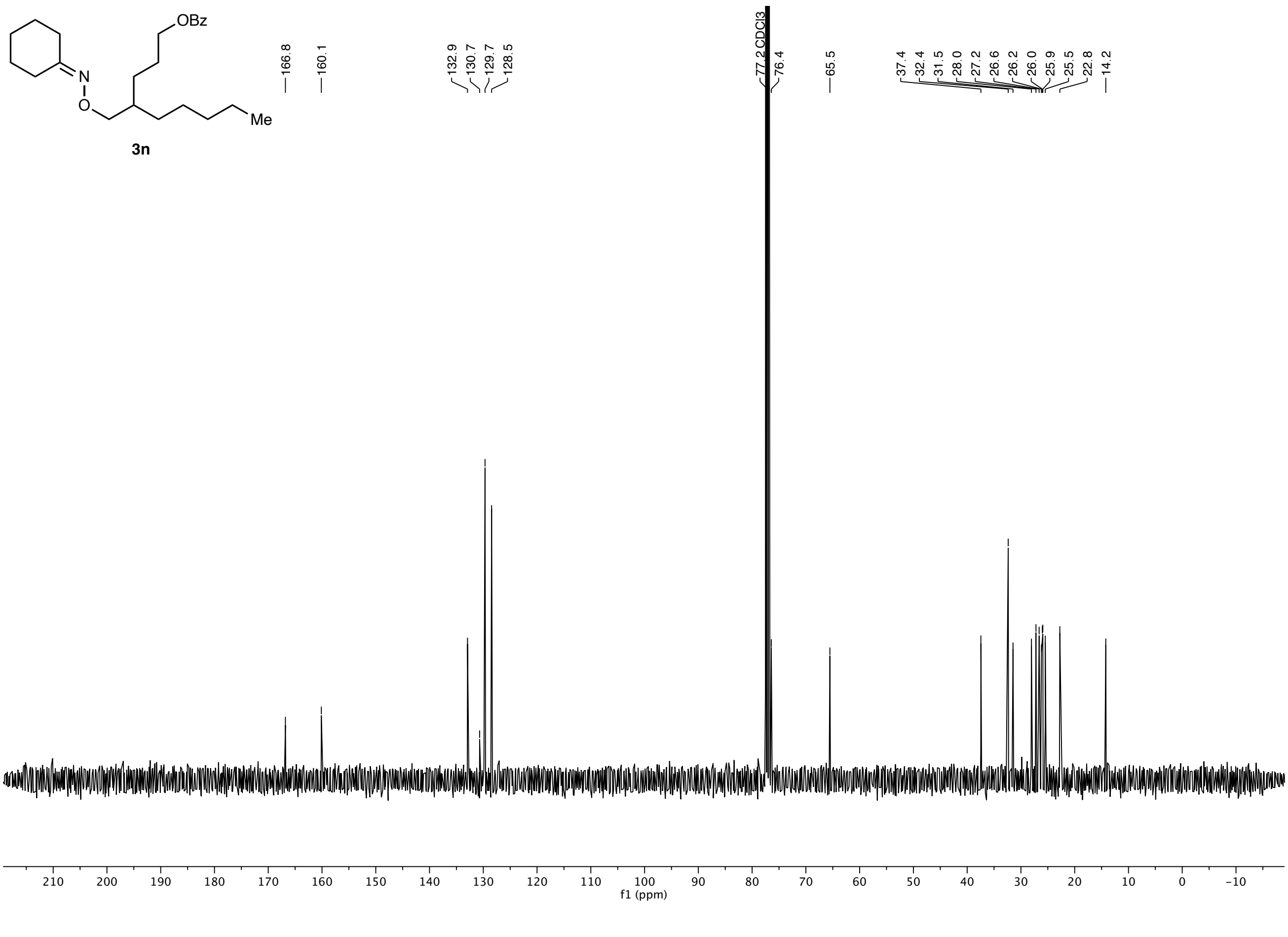
OBz
N
O
Me
3n
166.8 160.1 132.9 130.7 129.7 128.5 77.2 CDCl3 76.4 65.5 37.4 32.4 31.5 28.0 27.2 26.6 26.2 26.0 25.9 25.5 22.8 14.2
f1 (ppm)

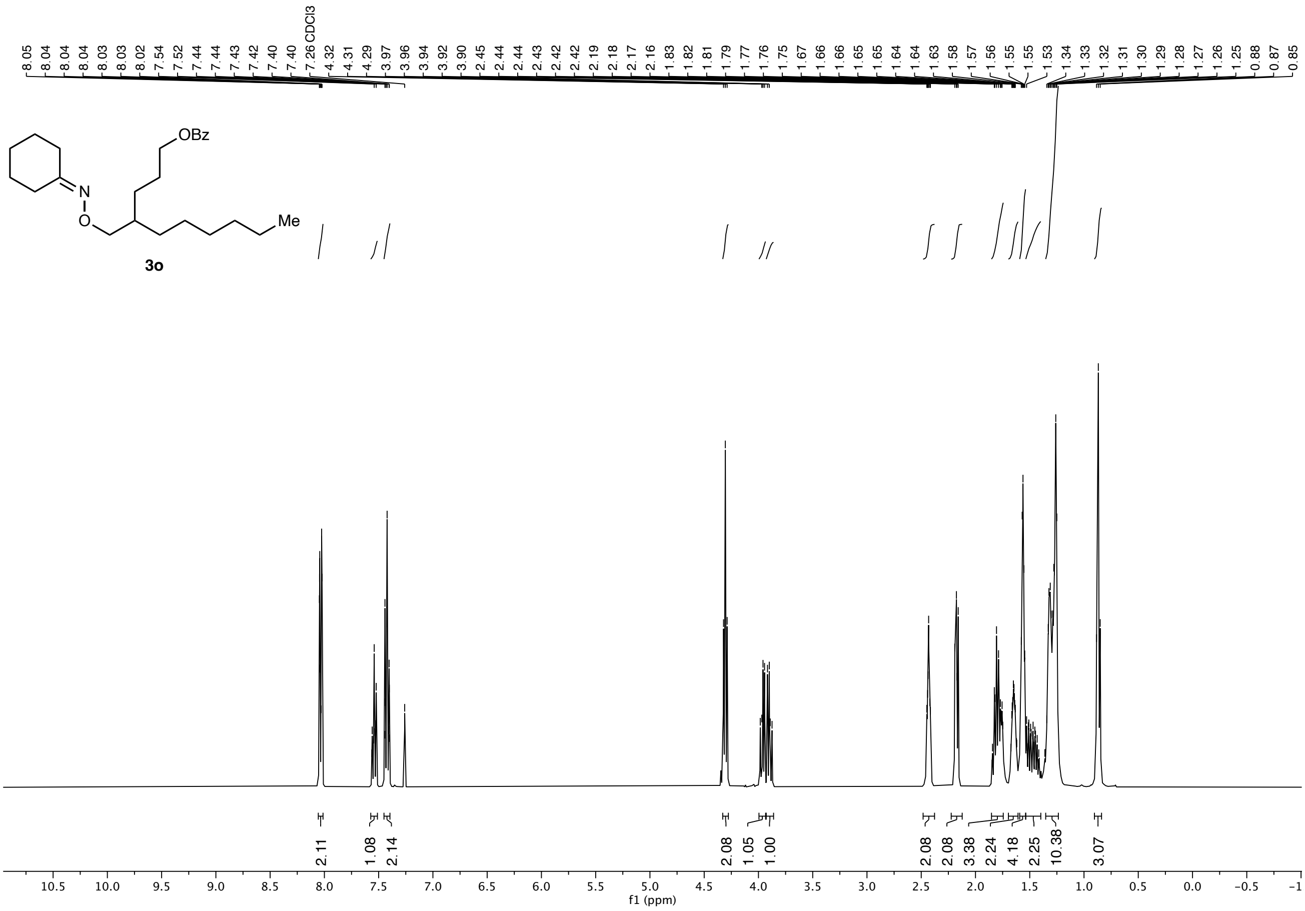
8.05 8.04 8.04 8.04 8.03 8.03 8.02 7.54 7.52 7.44 7.44 7.43 7.42 7.42 7.40 7.40 7.26 CDCl3 4.32 4.31 4.29 3.97 3.96 3.94 3.92 3.90 2.45 2.44 2.44 2.43 2.42 2.42 2.19 2.18 2.17 2.16 1.83 1.82 1.81 1.79 1.77 1.76 1.75 1.67 1.66 1.66 1.65 1.65 1.64 1.64 1.63 1.58 1.57 1.56 1.55 1.55 1.53 1.34 1.33 1.32 1.31 1.30 1.29 1.28 1.27 1.26 1.25 0.88 0.87 0.85
OBz
N
O
Me
3o
2.11
1.08
2.14
2.08
1.05
1.00
2.08
2.08
3.38
2.24
4.18
2.25
10.38
3.07
f1 (ppm)

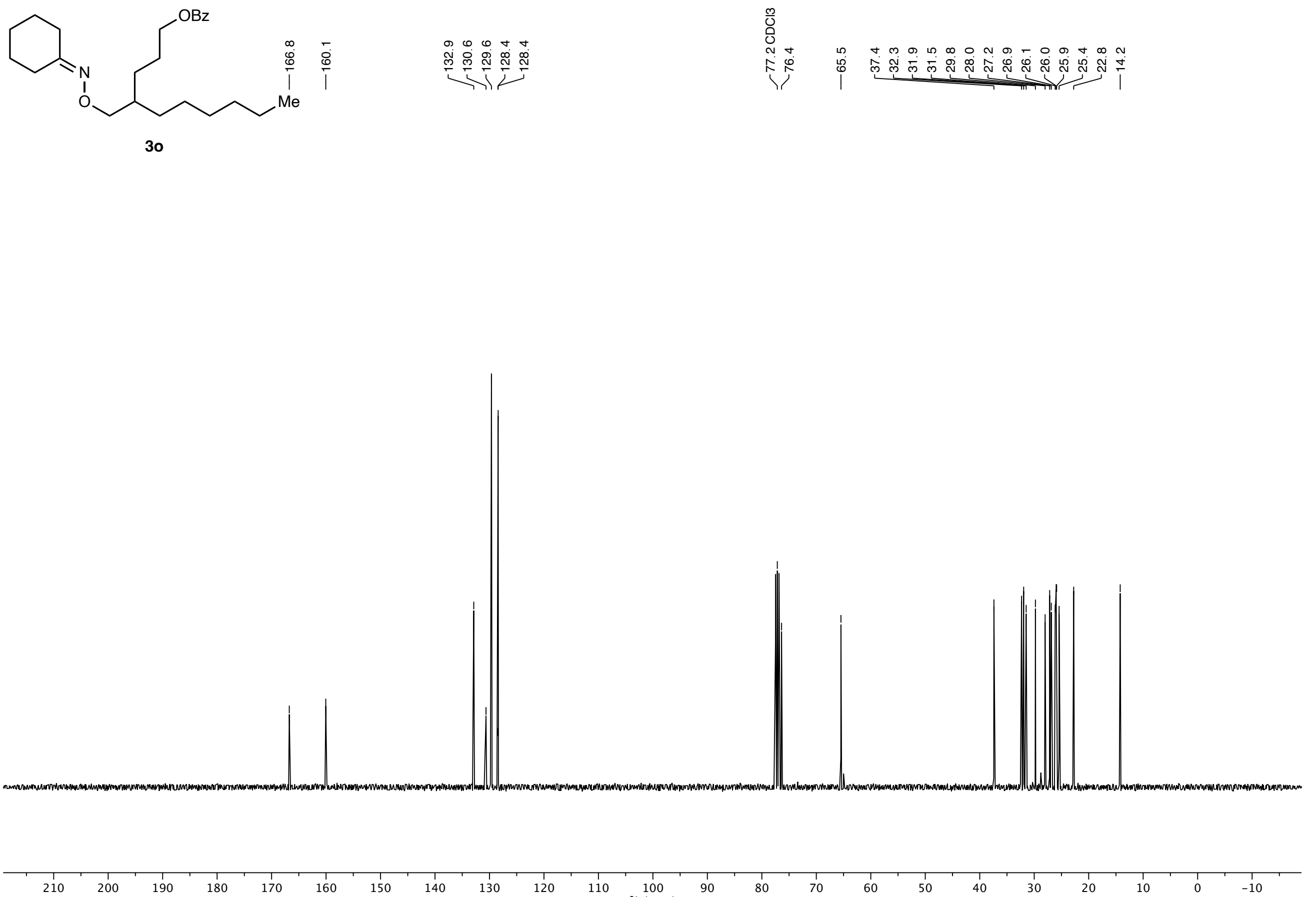
OBz
N
O
Me
3o
166.8
160.1
132.9 130.6 129.6 128.4 128.4
77.2 CDCl3
76.4
65.5
37.4 32.3 31.9 31.5 29.8 28.0 27.2 26.9 26.1 26.0 25.9 25.4 22.8
14.2
f1 (ppm)

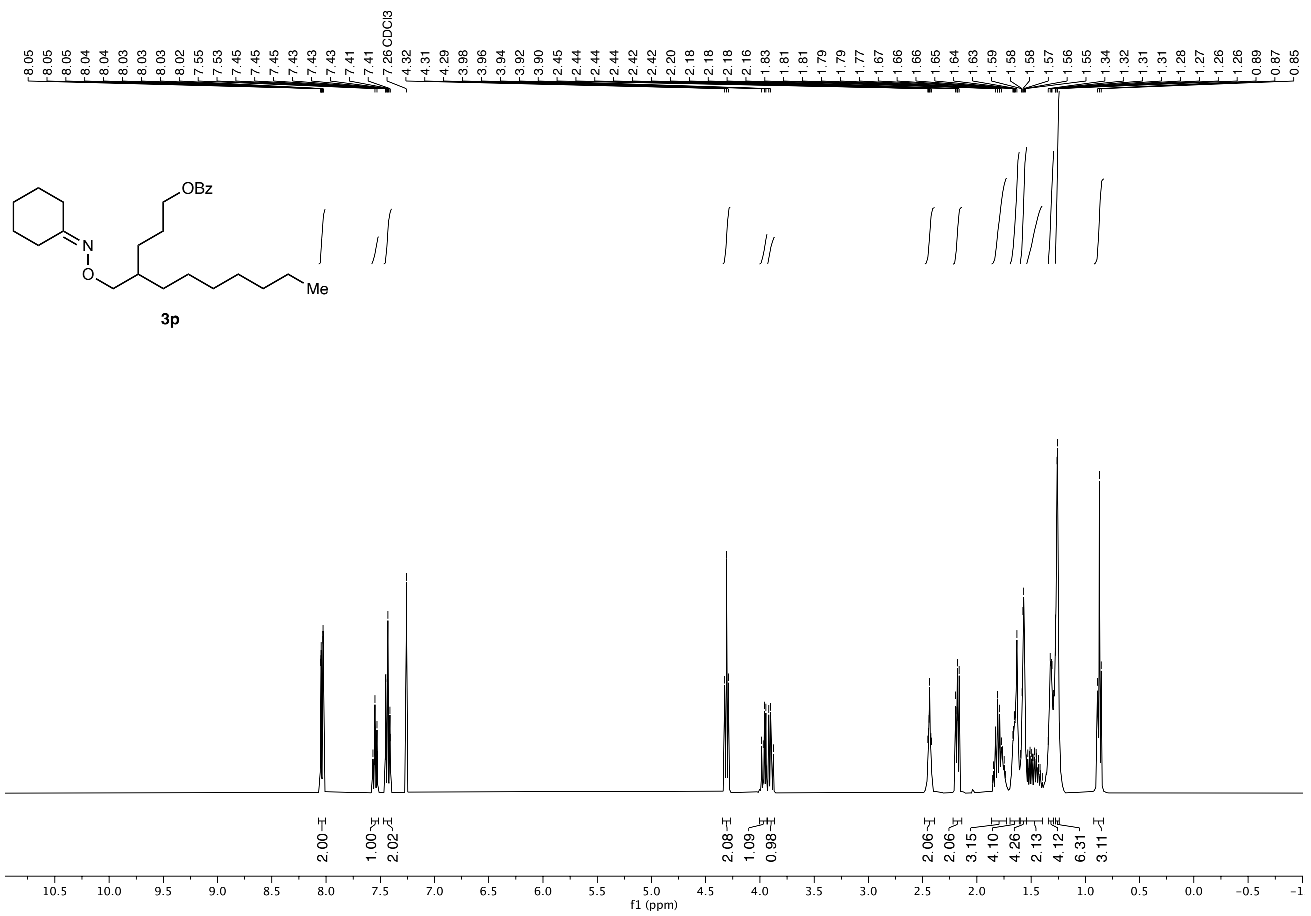
OBz
N
O
Me
3p
f1 (ppm)

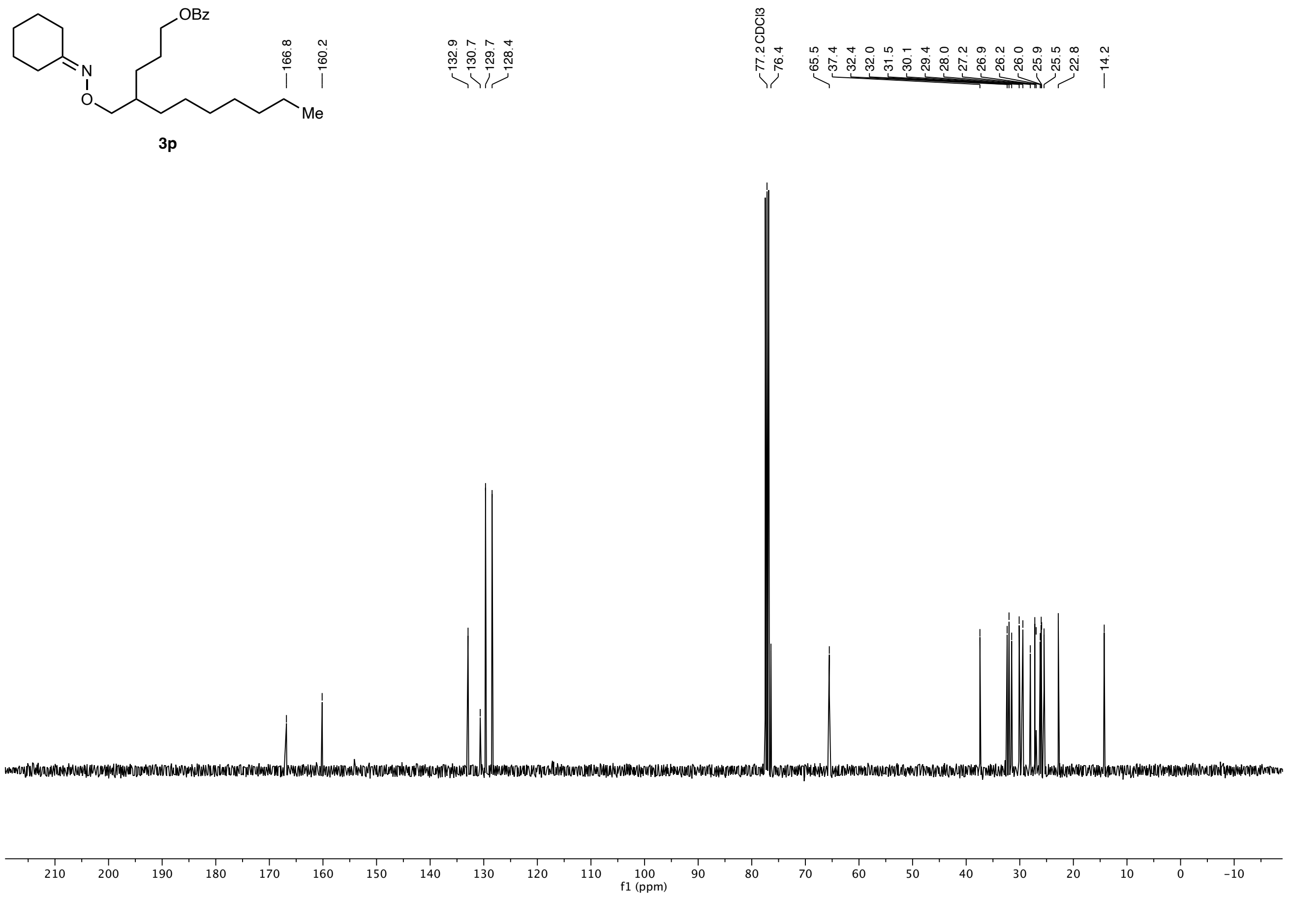
OBz
N
O
Me
3p
f1 (ppm)

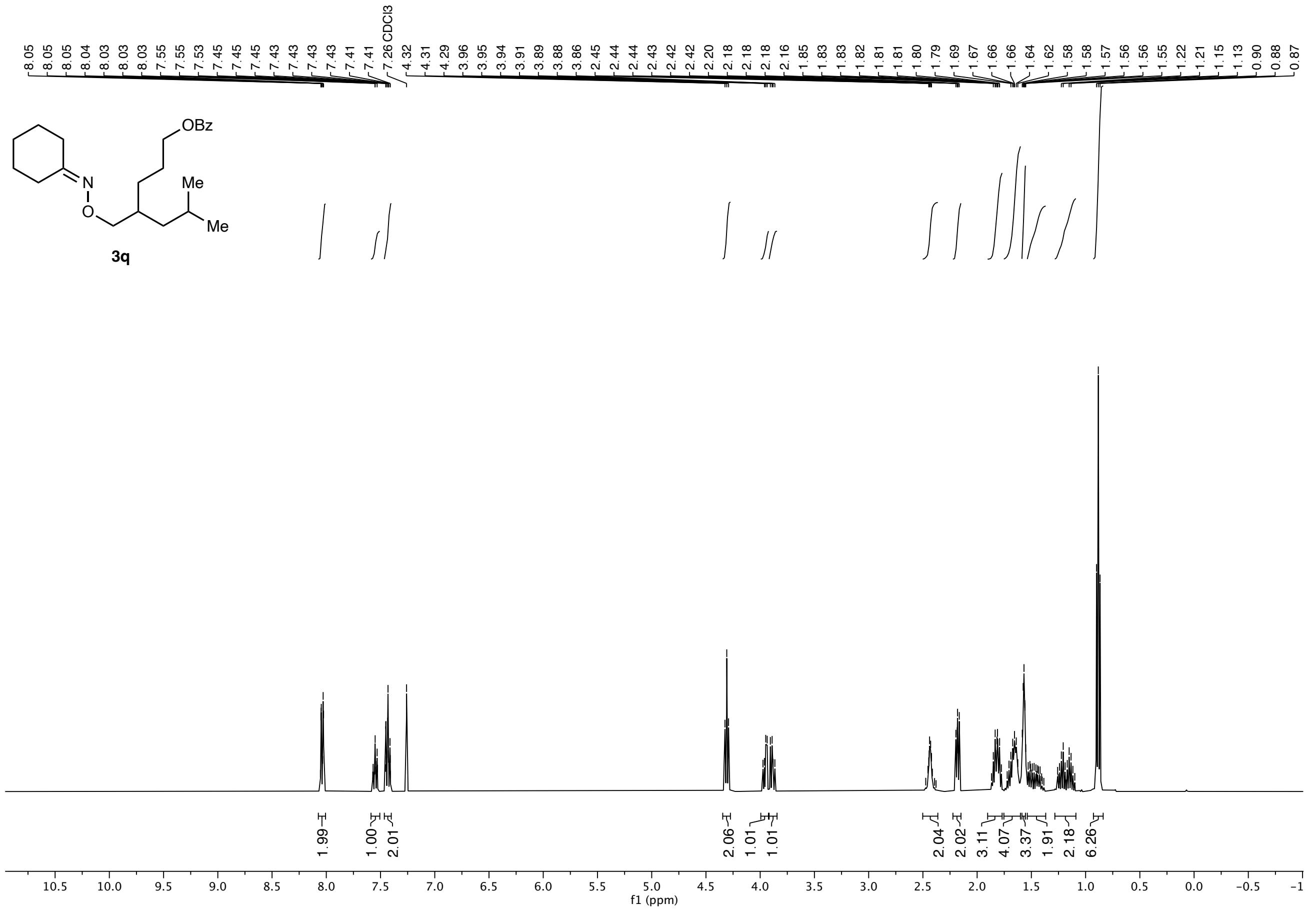
8.05 8.05 8.05 8.04 8.03 8.03 8.03 7.55 7.55 7.53 7.45 7.45 7.45 7.43 7.43 7.43 7.41 7.41 7.26 CDCl3 4.32 4.31 4.29 3.96 3.95 3.94 3.91 3.89 3.88 3.86 2.45 2.44 2.44 2.43 2.42 2.42 2.20 2.18 2.18 2.18 2.16 1.85 1.83 1.83 1.82 1.81 1.81 1.80 1.79 1.69 1.67 1.66 1.66 1.64 1.62 1.58 1.58 1.57 1.56 1.56 1.55 1.22 1.21 1.15 1.13 0.90 0.88 0.87
OBz
N
O
Me
Me
3q
1.99 1.00 2.01 2.06 1.01 1.01 2.04 2.02 3.11 4.07 3.37 1.91 2.18 6.26
f1 (ppm)

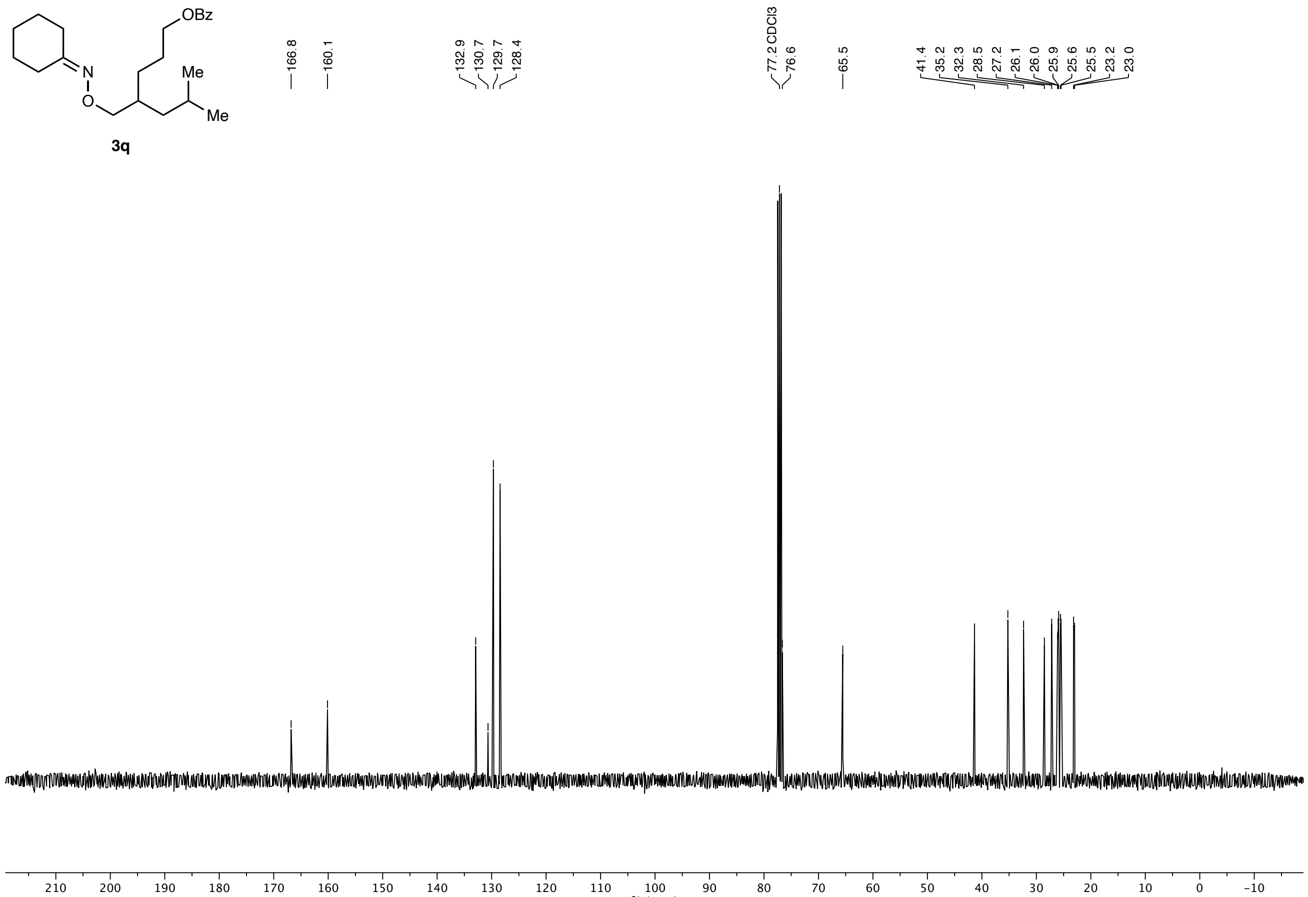
OBz
N
O
Me
Me
3q
166.8 160.1 132.9 130.7 129.7 128.4 77.2 CDCl3 76.6 65.5 41.4 35.2 32.3 28.5 27.2 26.1 26.0 25.9 25.6 25.5 23.2 23.0
f1 (ppm)

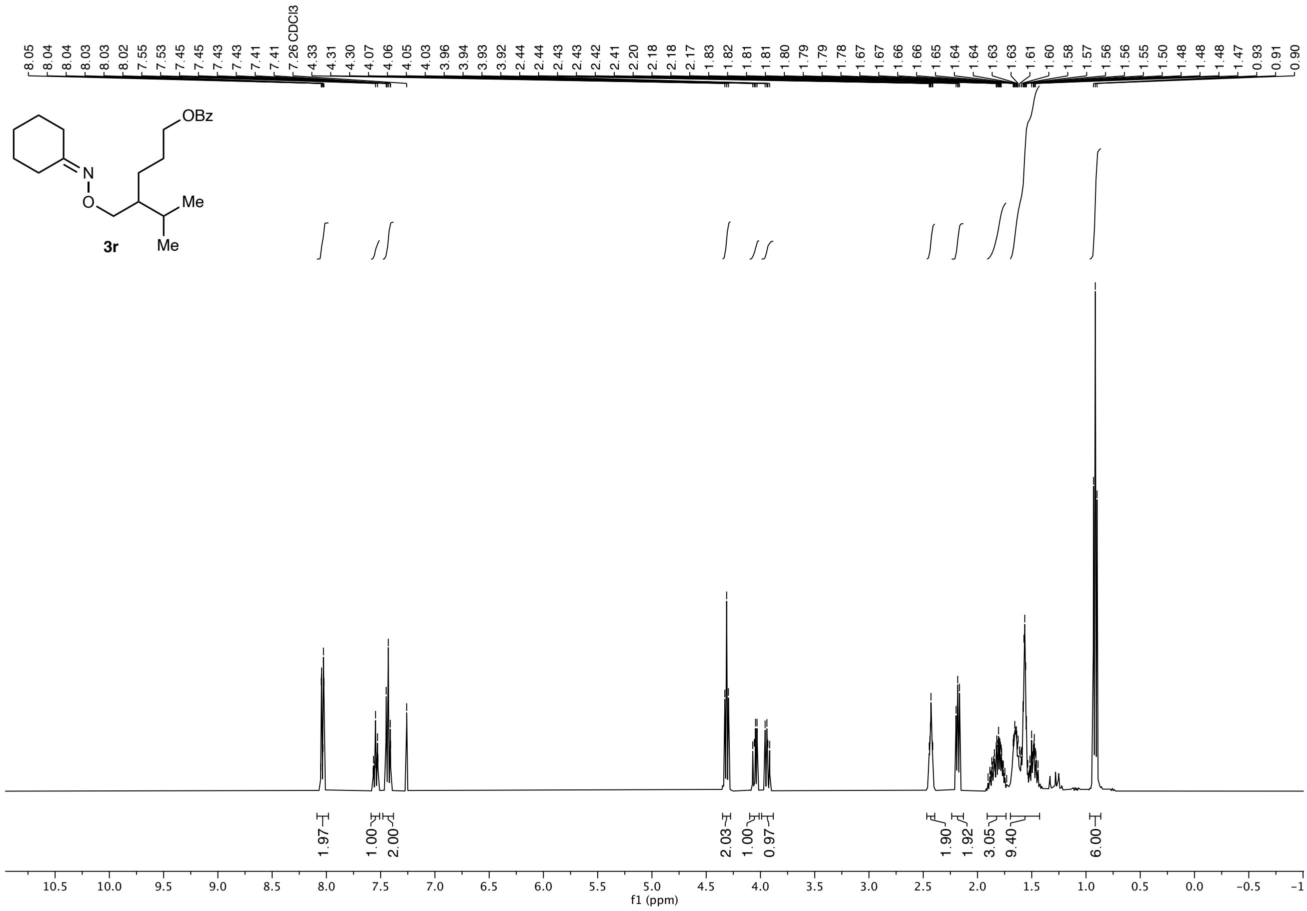
OBz
N
O
Me
Me
3r
8.05 8.04 8.04 8.03 8.03 8.02 7.55 7.53 7.45 7.45 7.43 7.43 7.41 7.41 7.26 CDCl3 4.33 4.31 4.30 4.07 4.06 4.05 4.03 3.96 3.94 3.93 3.92 2.44 2.43 2.43 2.42 2.41 2.20 2.18 2.18 2.17 1.83 1.82 1.81 1.81 1.80 1.79 1.79 1.78 1.67 1.67 1.66 1.66 1.65 1.64 1.64 1.63 1.63 1.61 1.60 1.58 1.57 1.56 1.56 1.55 1.50 1.48 1.48 1.48 1.47 0.93 0.91 0.90
1.97 1.00 2.00 2.03 1.00 0.97 1.90 1.92 3.05 9.40 6.00
f1 (ppm)

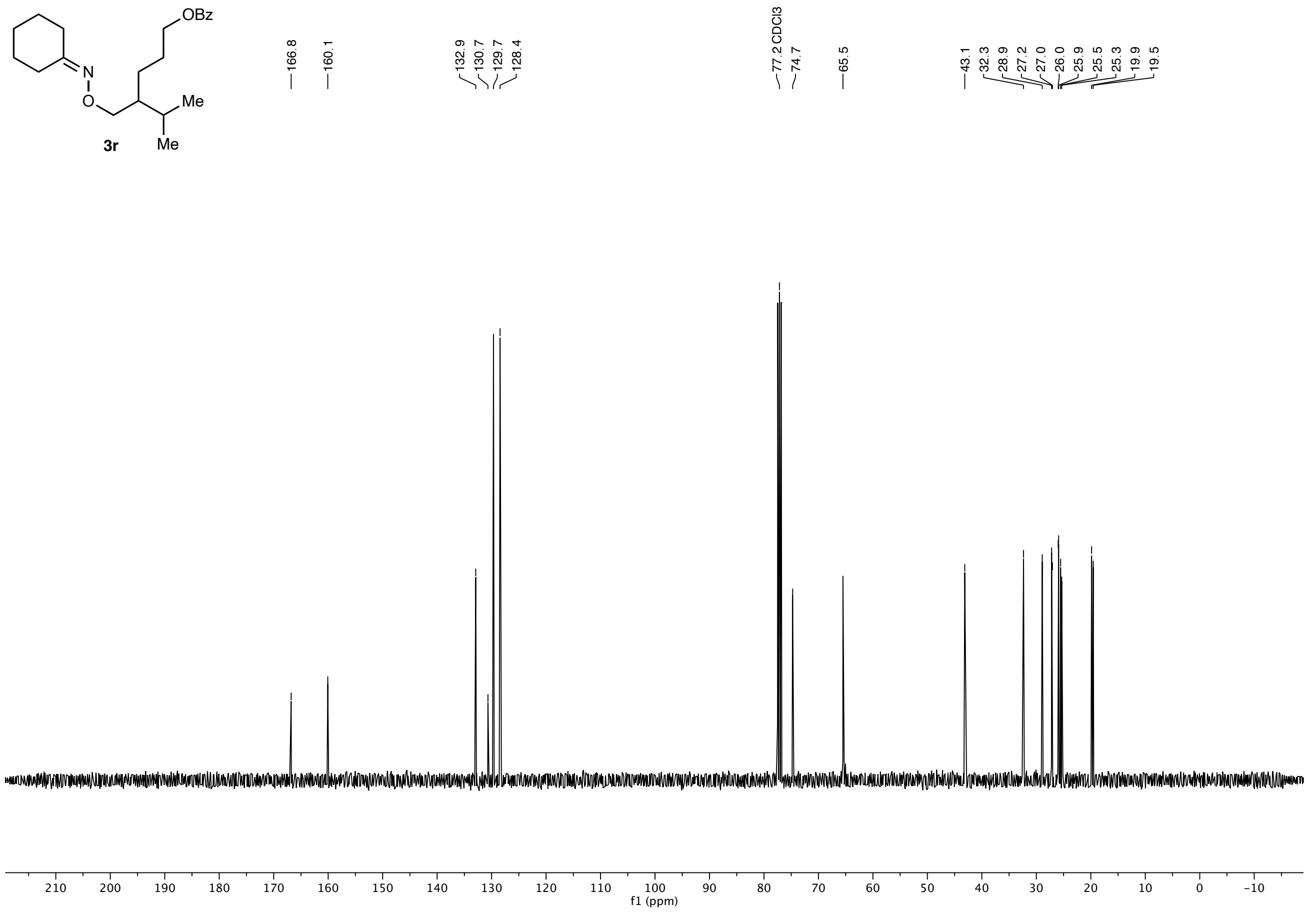
OBz
N
O
Me
Me
3r
166.8 160.1 132.9 130.7 129.7 128.4 77.2 CDCl3 74.7 65.5 43.1 32.3 28.9 27.2 27.0 26.0 25.9 25.5 25.3 19.9 19.5
f1 (ppm)

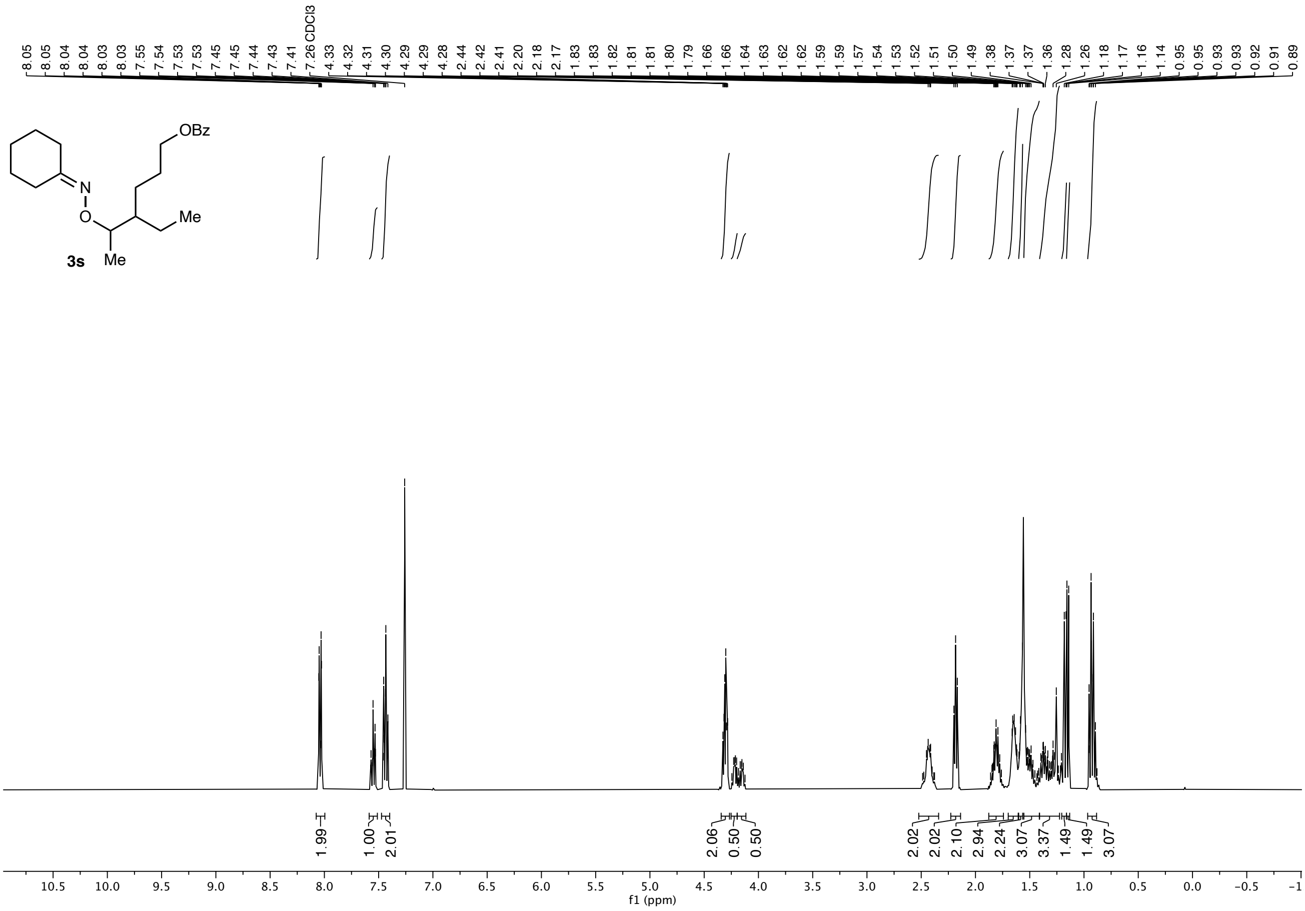
OBz
N
O
Me
Me
3s
f1 (ppm)

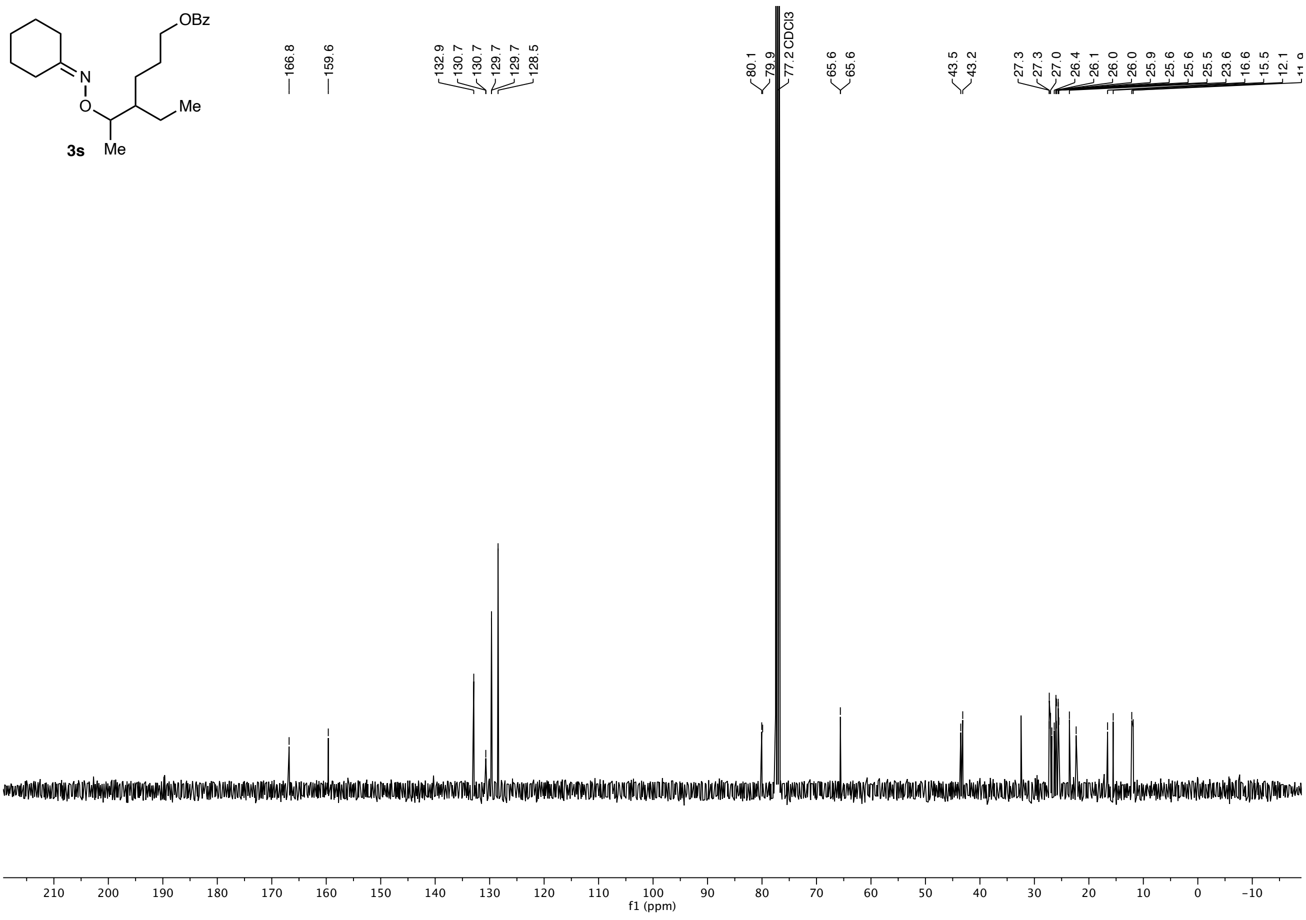
OBz
N
O
Me
Me
3s
f1 (ppm)

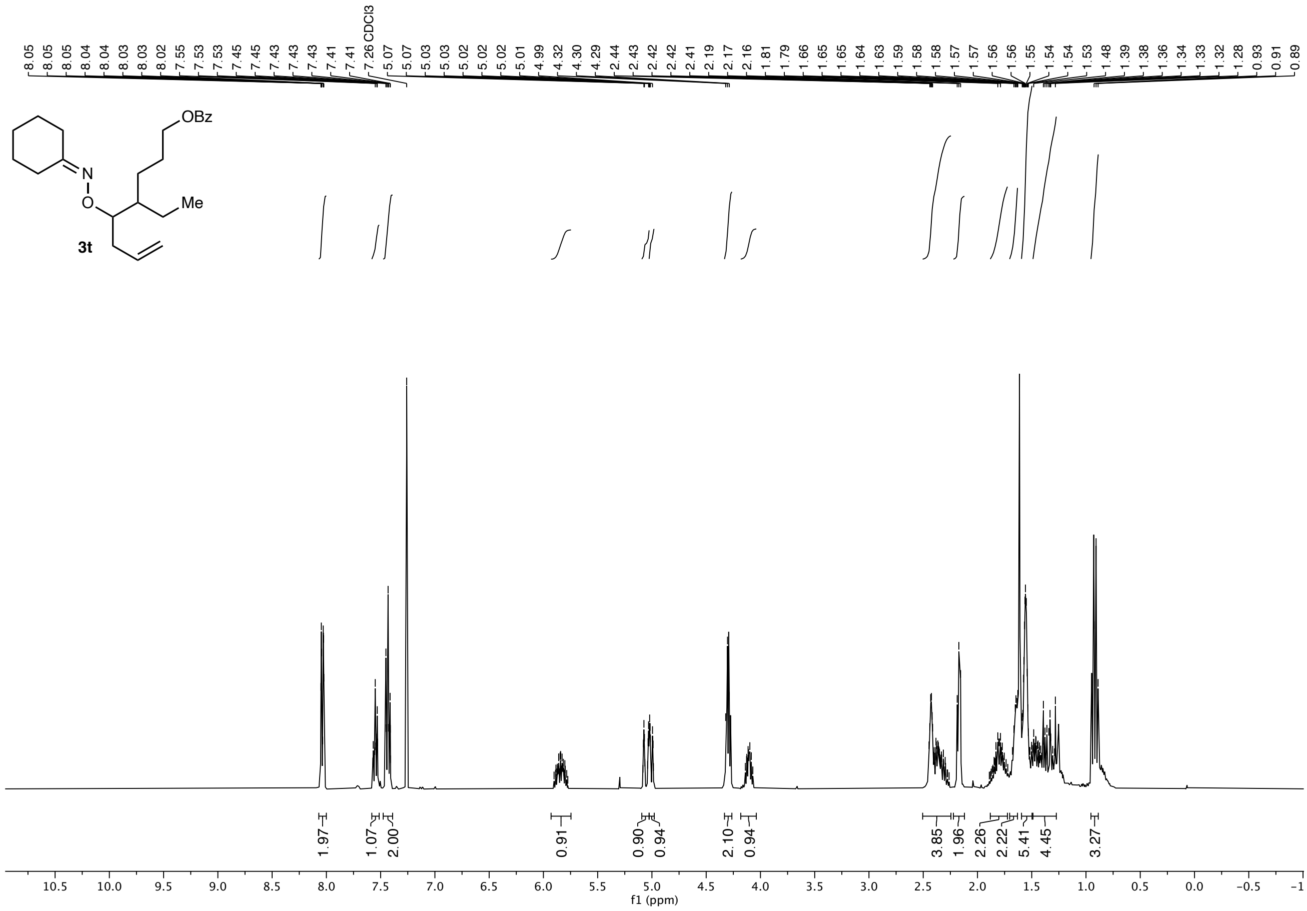
8.05 8.05 8.05 8.04 8.04 8.03 8.03 8.02 7.55 7.53 7.53 7.45 7.45 7.43 7.43 7.43 7.41 7.41 7.26 CDCl3 5.07 5.07 5.03 5.03 5.02 5.02 5.02 5.01 4.99 4.32 4.30 4.29 2.44 2.43 2.42 2.42 2.41 2.19 2.17 2.16 1.81 1.79 1.66 1.65 1.65 1.64 1.63 1.59 1.58 1.58 1.57 1.57 1.56 1.56 1.55 1.54 1.54 1.53 1.48 1.39 1.38 1.36 1.34 1.33 1.32 1.28 0.93 0.91 0.89
OBz
N
O
Me
3t
1.97
1.07
2.00
0.91
0.90
0.94
2.10
0.94
3.85
1.96
2.26
2.22
5.41
4.45
3.27
f1 (ppm)

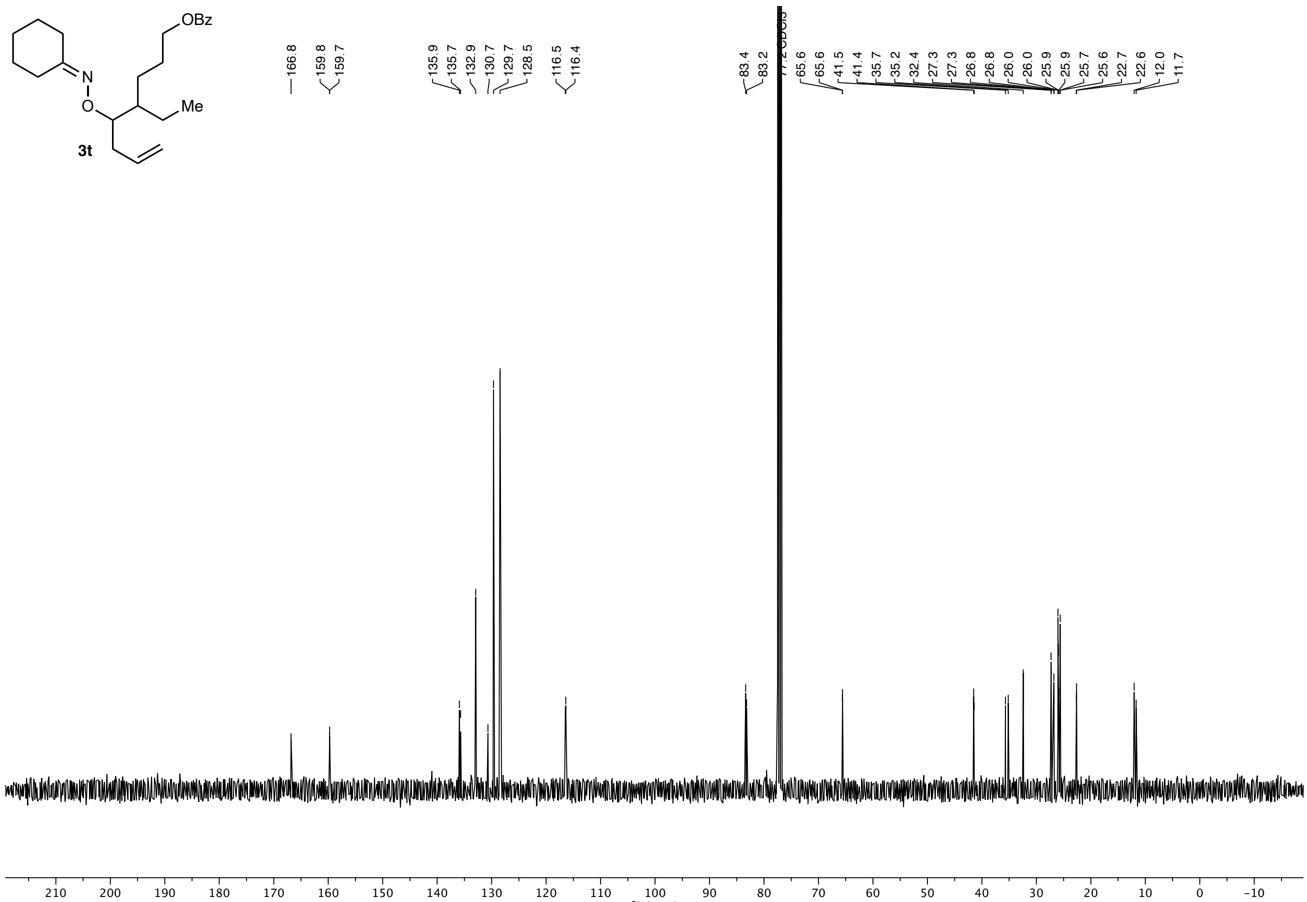
166.8 159.8 159.7 135.9 135.7 132.9 130.7 129.7 128.5 116.5 116.4 83.4 83.2 65.6 65.6 41.5 41.4 35.7 35.2 32.4 27.3 27.3 26.8 26.8 26.0 26.0 25.9 25.9 25.7 25.6 22.7 22.6 12.0 11.7
OBz
N
O
Me
3t
f1 (ppm)

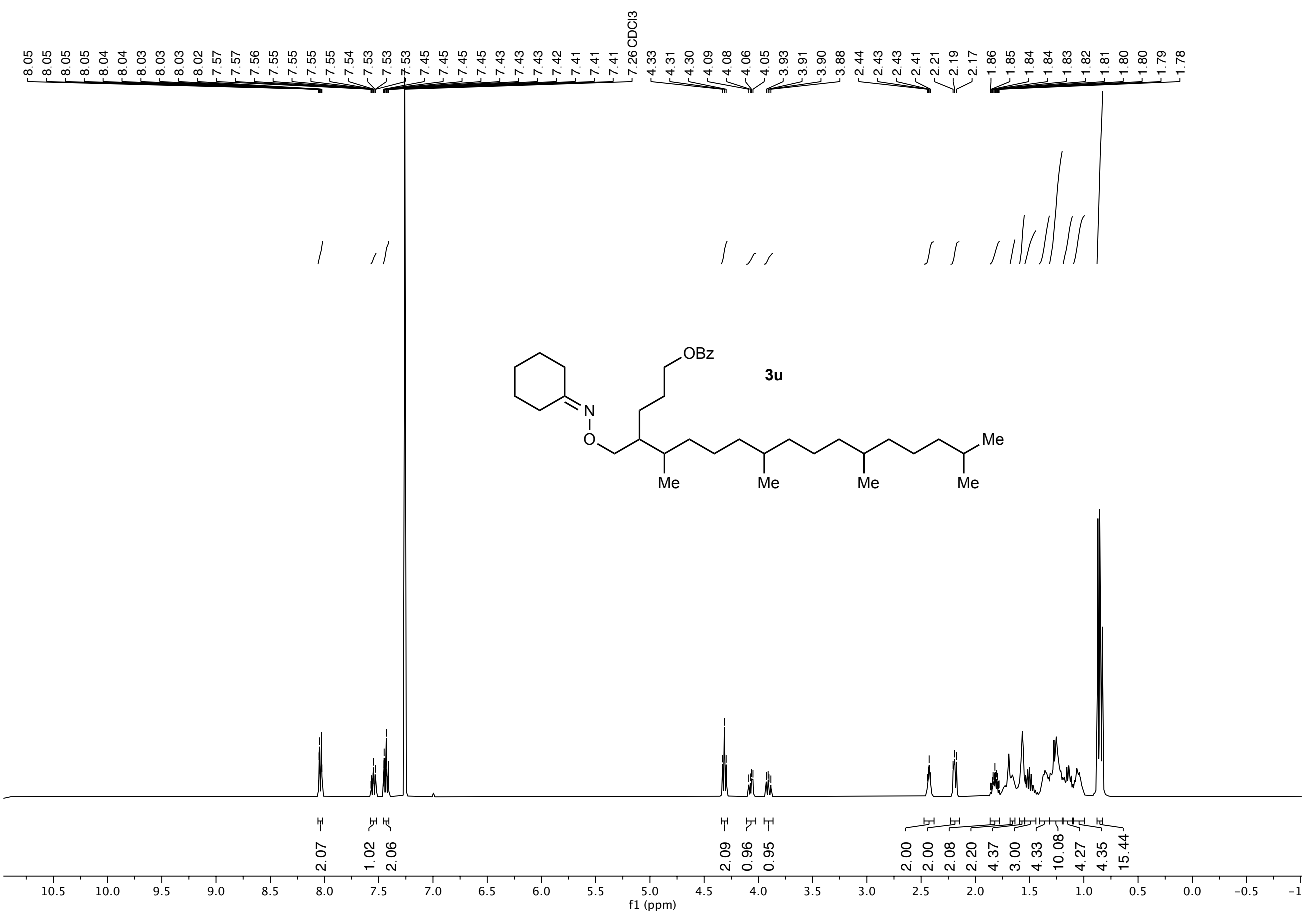
8.05 8.05 8.05 8.05 8.04 8.04 8.03 8.03 8.03 8.02 7.57 7.57 7.56 7.55 7.55 7.55 7.55 7.54 7.53 7.53 7.53 7.45 7.45 7.45 7.45 7.43 7.43 7.43 7.42 7.41 7.41 7.26 CDCl3 4.33 4.31 4.30 4.09 4.08 4.06 4.05 3.93 3.91 3.90 3.88 2.44 2.43 2.43 2.41 2.21 2.19 2.17 1.86 1.85 1.84 1.84 1.83 1.82 1.81 1.80 1.80 1.79 1.78
OBz
3u
N
O
Me
2.07 1.02 2.06 2.09 0.96 0.95 2.00 2.00 2.08 2.20 4.37 3.00 4.33 10.08 4.27 4.35 15.44
f1 (ppm)

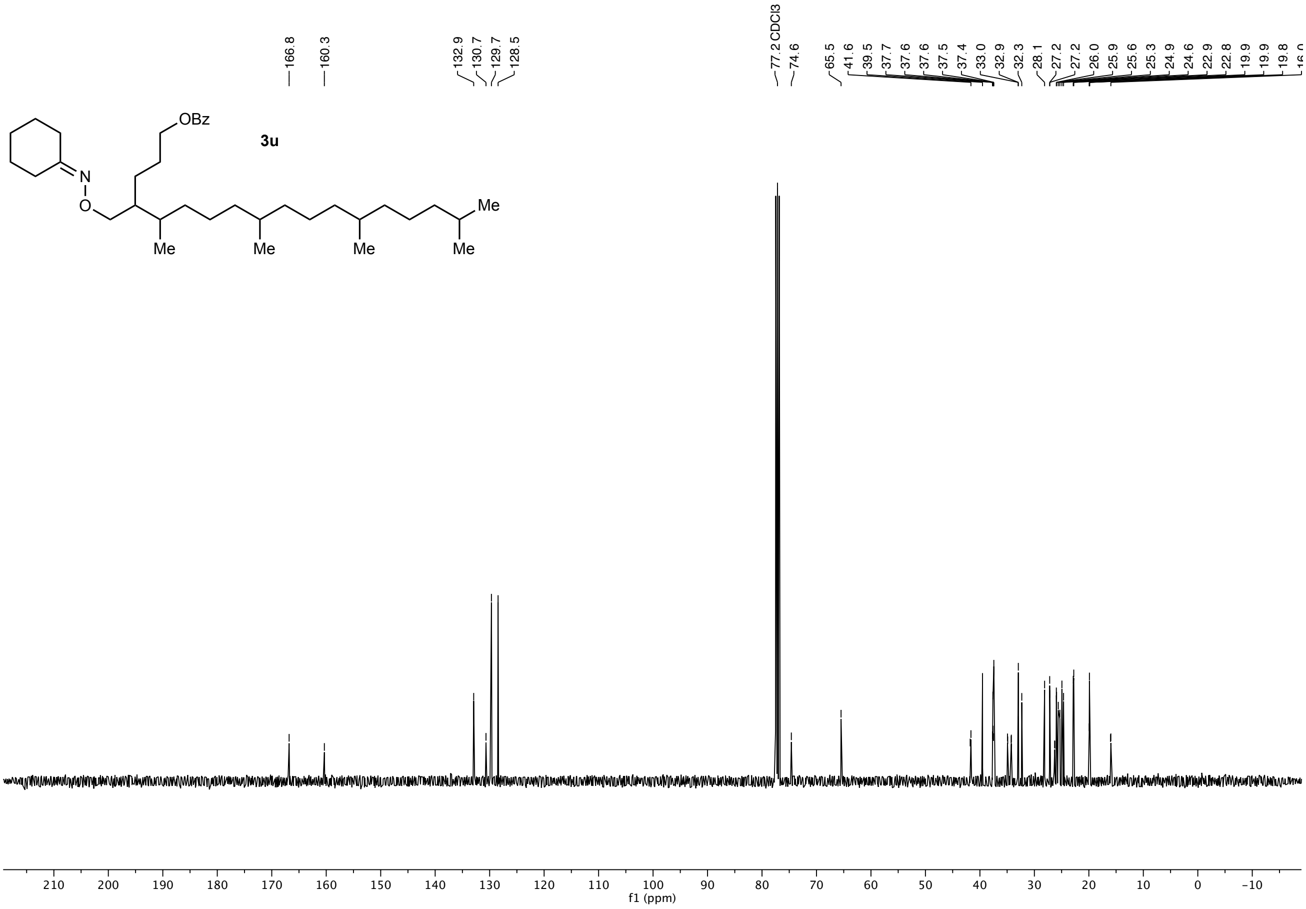
166.8 160.3 132.9 130.7 129.7 128.5 77.2 CDCl3 74.6 65.5 41.6 39.5 37.7 37.6 37.6 37.5 37.4 33.0 32.9 32.3 28.1 27.2 27.2 26.0 25.9 25.6 25.3 24.9 24.6 22.9 22.8 19.9 19.9 19.8
OBz
3u
N
O
Me
f1 (ppm)

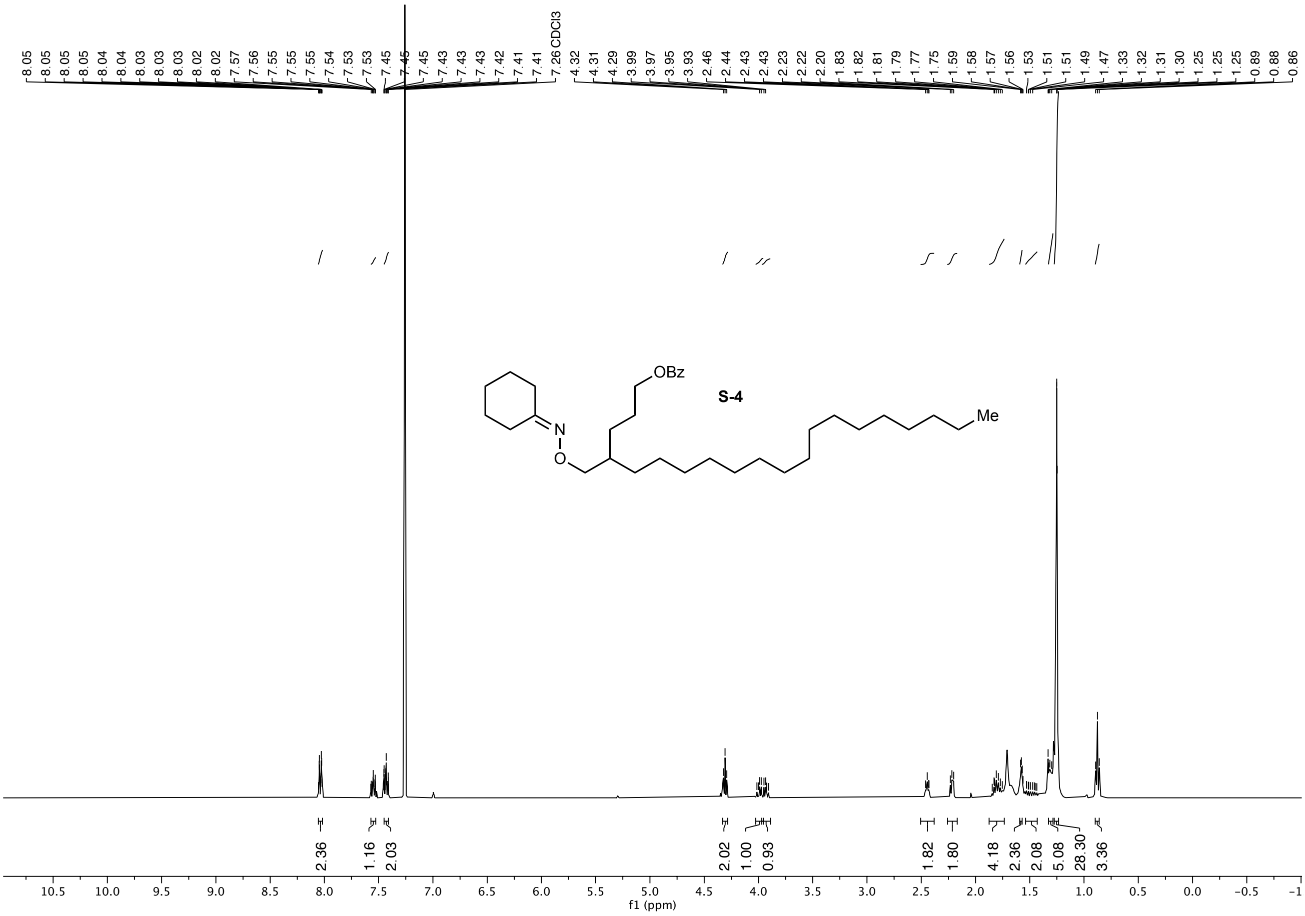
8.05 8.05 8.05 8.05 8.04 8.03 8.03 8.03 8.02 8.02 7.57 7.56 7.55 7.55 7.54 7.53 7.53 7.45 7.45 7.45 7.43 7.43 7.43 7.42 7.41 7.26 CDCl3 4.32 4.31 4.29 3.99 3.97 3.95 3.93 2.46 2.44 2.43 2.43 2.23 2.22 2.20 1.83 1.82 1.81 1.79 1.77 1.75 1.59 1.58 1.57 1.56 1.53 1.51 1.51 1.49 1.47 1.33 1.32 1.31 1.30 1.25 1.25 1.25 0.89 0.88 0.86
OBz
S-4
N
O
Me
2.36 1.16 2.03 2.02 1.00 0.93 1.82 1.80 4.18 2.36 2.08 5.08 28.30 3.36
10.5 10.0 9.5 9.0 8.5 8.0 7.5 7.0 6.5 6.0 5.5 5.0 4.5 4.0 3.5 3.0 2.5 2.0 1.5 1.0 0.5 0.0 −0.5 −1
f1 (ppm)

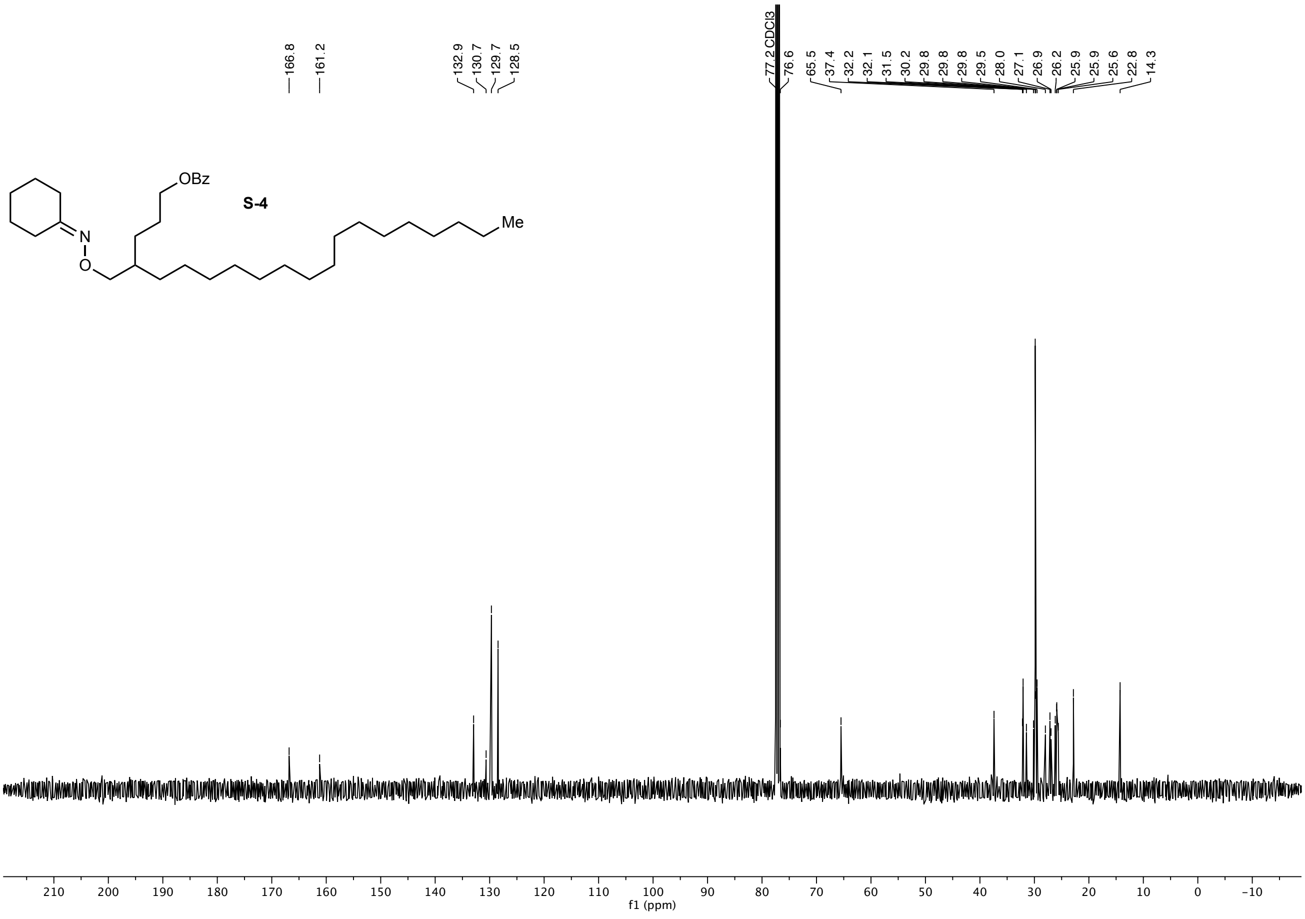
166.8 161.2 132.9 130.7 129.7 128.5 77.2 CDCl3 76.6 65.5 37.4 32.2 32.1 31.5 30.2 29.8 29.8 29.8 29.5 28.0 27.1 26.9 26.2 25.9 25.9 25.6 22.8 14.3
OBz
S-4
N
O
Me
210 200 190 180 170 160 150 140 130 120 110 100 90 80 70 60 50 40 30 20 10 0 −10
f1 (ppm)

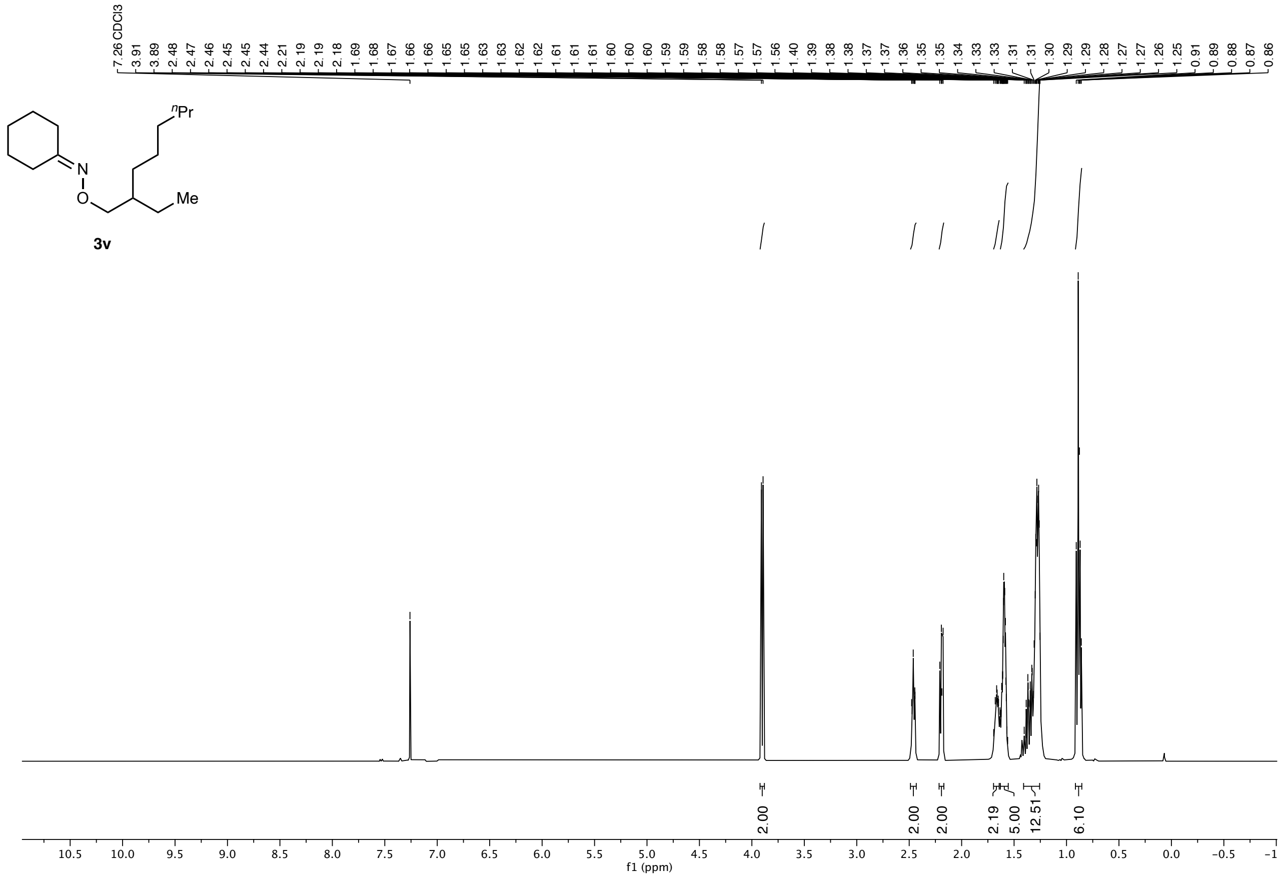
7.26 CDCl3
3.91 3.89 2.48 2.47 2.46 2.45 2.45 2.44 2.21 2.19 2.19 2.18 1.69 1.68 1.67 1.66 1.66 1.65 1.65 1.63 1.63 1.62 1.62 1.61 1.61 1.61 1.60 1.60 1.60 1.59 1.59 1.58 1.58 1.57 1.57 1.56 1.40 1.39 1.38 1.38 1.37 1.37 1.36 1.35 1.35 1.34 1.33 1.33 1.31 1.31 1.30 1.29 1.29 1.28 1.27 1.27 1.26 1.25 0.91 0.89 0.88 0.87 0.86
N
O
^{n}Pr
Me
2.00
2.00
2.00
2.19
5.00
12.51
6.10
f1 (ppm)

3v

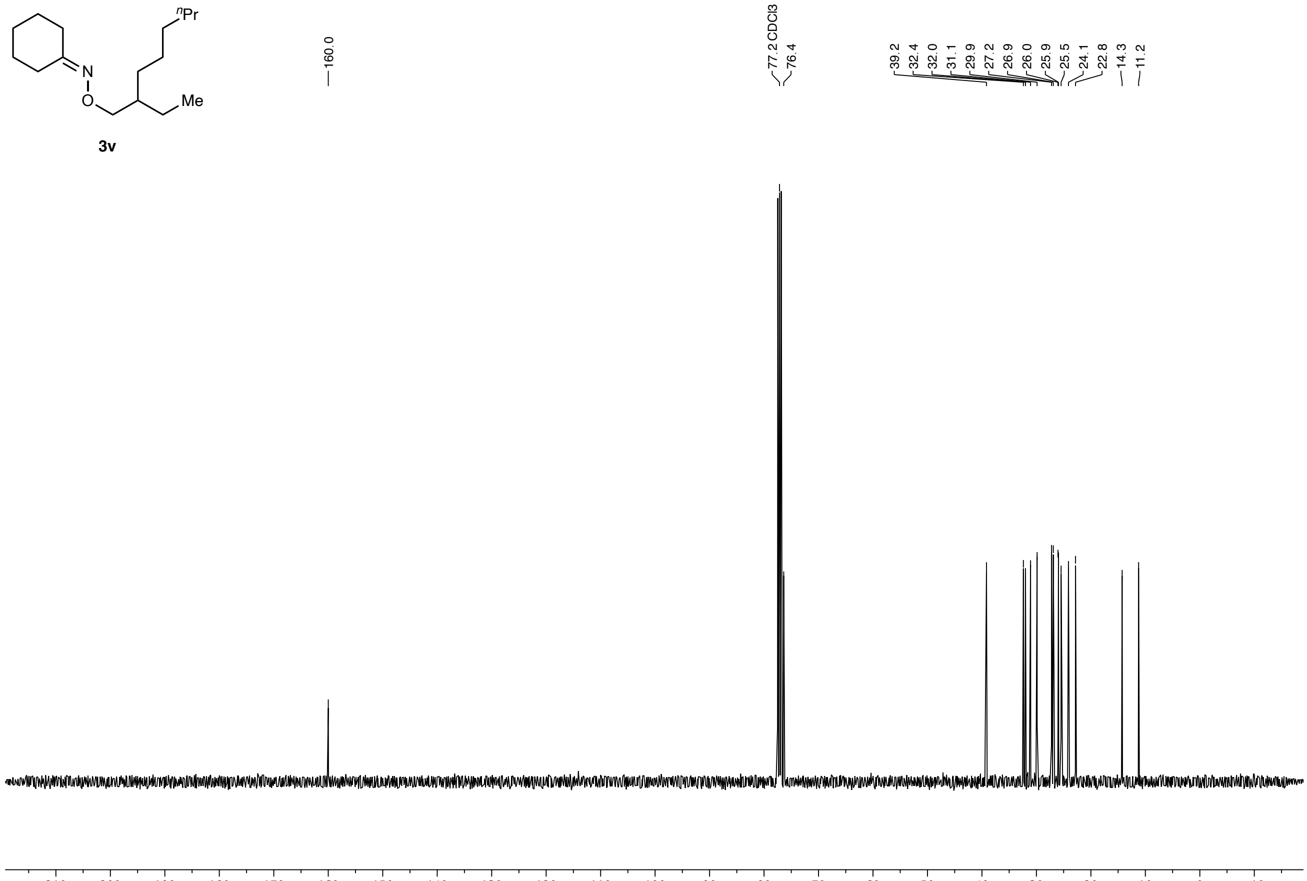
160.0
77.2 CDCl3
76.4
39.2 32.4 32.0 31.1 29.9 27.2 26.9 26.0 25.9 25.5 24.1 22.8
14.3
11.2
N
O
^{n}Pr
Me
f1 (ppm)

3v

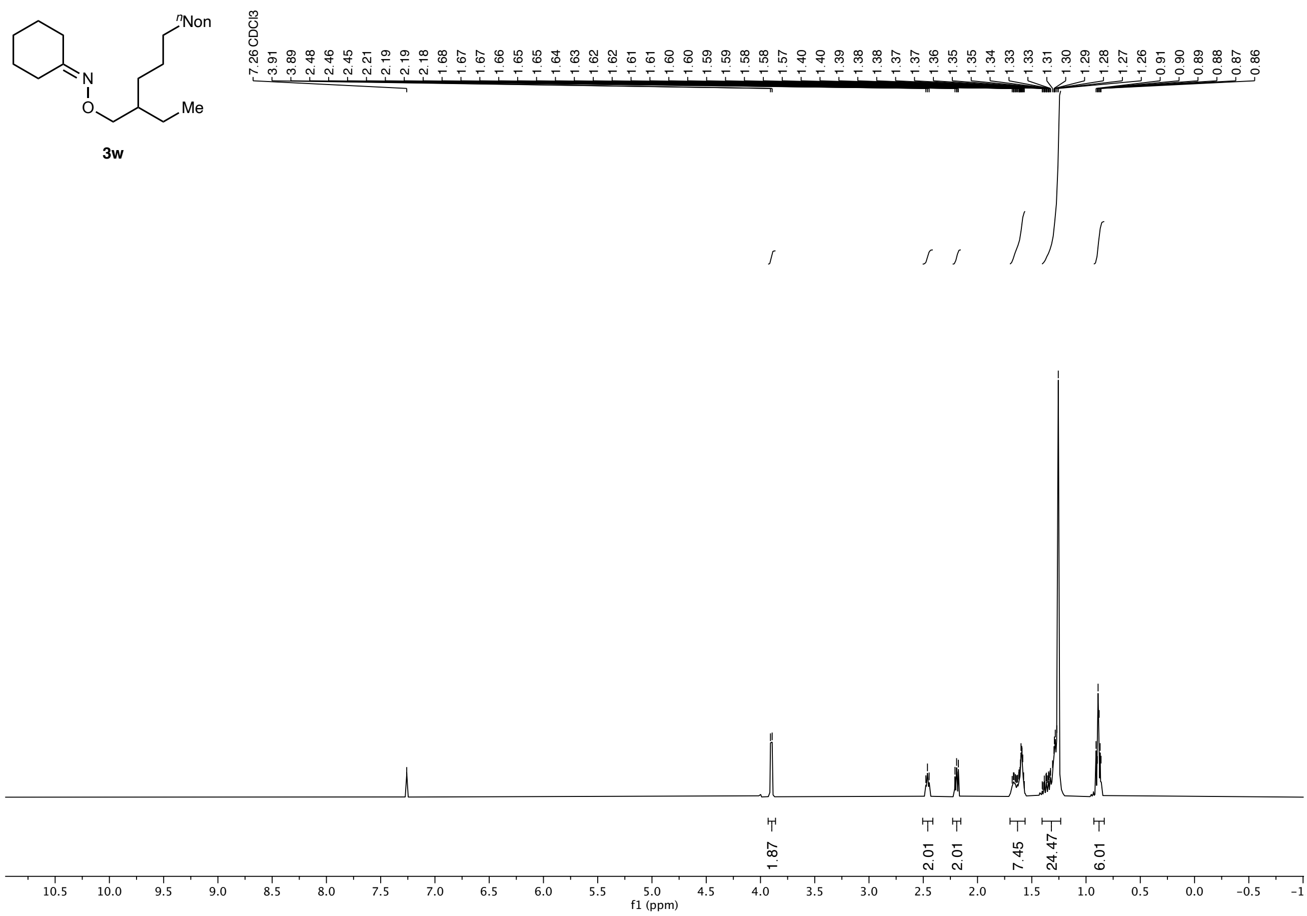

ⁿNon
N
O
Me
3w
7.26 CDCl3, 3.91, 3.89, 2.48, 2.46, 2.45, 2.21, 2.19, 2.19, 2.18, 1.68, 1.67, 1.67, 1.66, 1.65, 1.65, 1.64, 1.63, 1.62, 1.62, 1.61, 1.61, 1.60, 1.60, 1.59, 1.59, 1.58, 1.58, 1.57, 1.40, 1.40, 1.39, 1.38, 1.38, 1.37, 1.37, 1.36, 1.35, 1.35, 1.34, 1.33, 1.33, 1.31, 1.30, 1.29, 1.28, 1.27, 1.26, 0.91, 0.90, 0.89, 0.88, 0.87, 0.86
1.87
2.01
2.01
7.45
24.47
6.01
f1 (ppm)

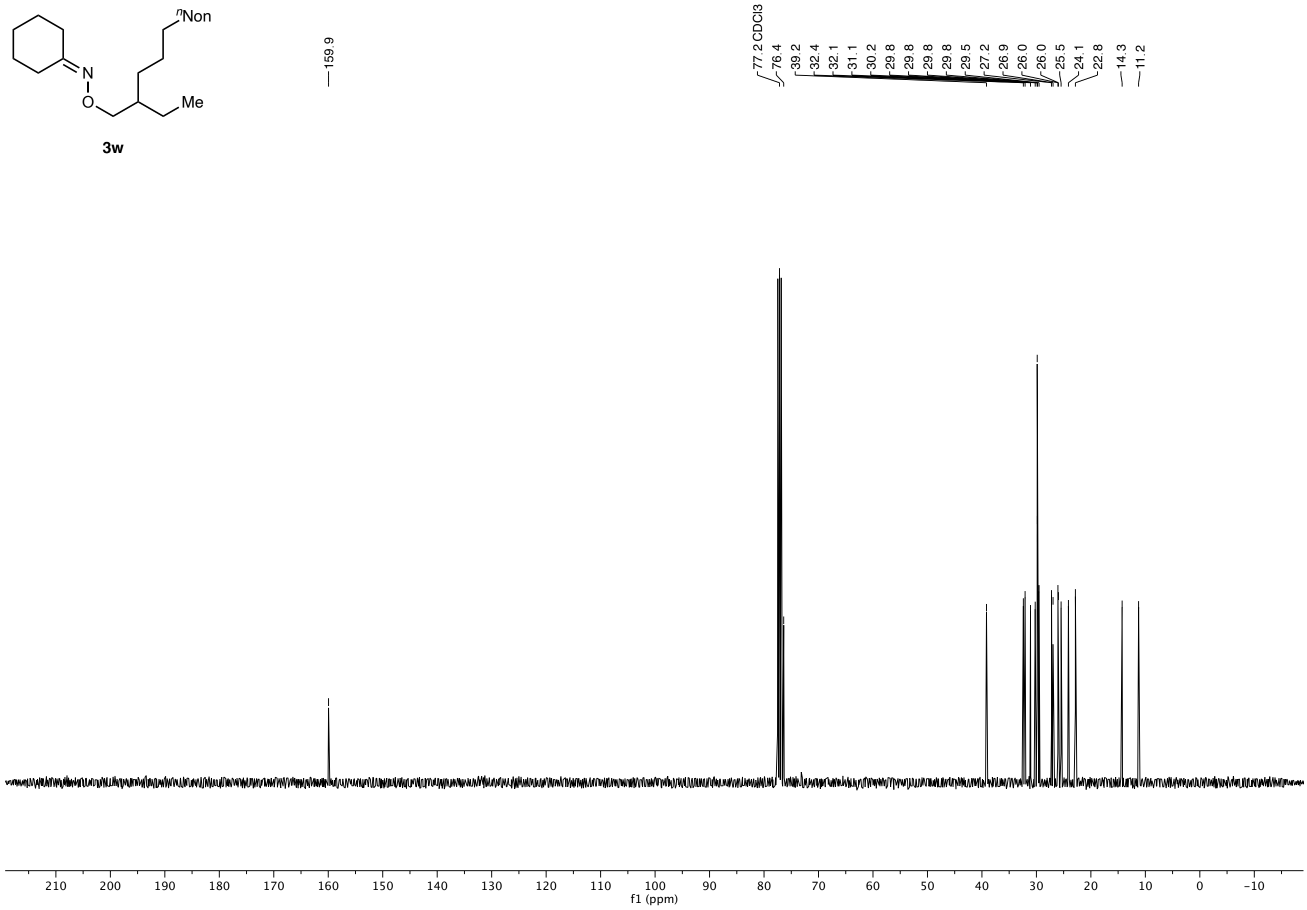

ⁿNon
N
O
Me
3w
159.9
77.2 CDCl3, 76.4, 39.2, 32.4, 32.1, 31.1, 30.2, 29.8, 29.8, 29.8, 29.8, 29.5, 27.2, 26.9, 26.0, 26.0, 25.5, 24.1, 22.8, 14.3, 11.2
f1 (ppm)

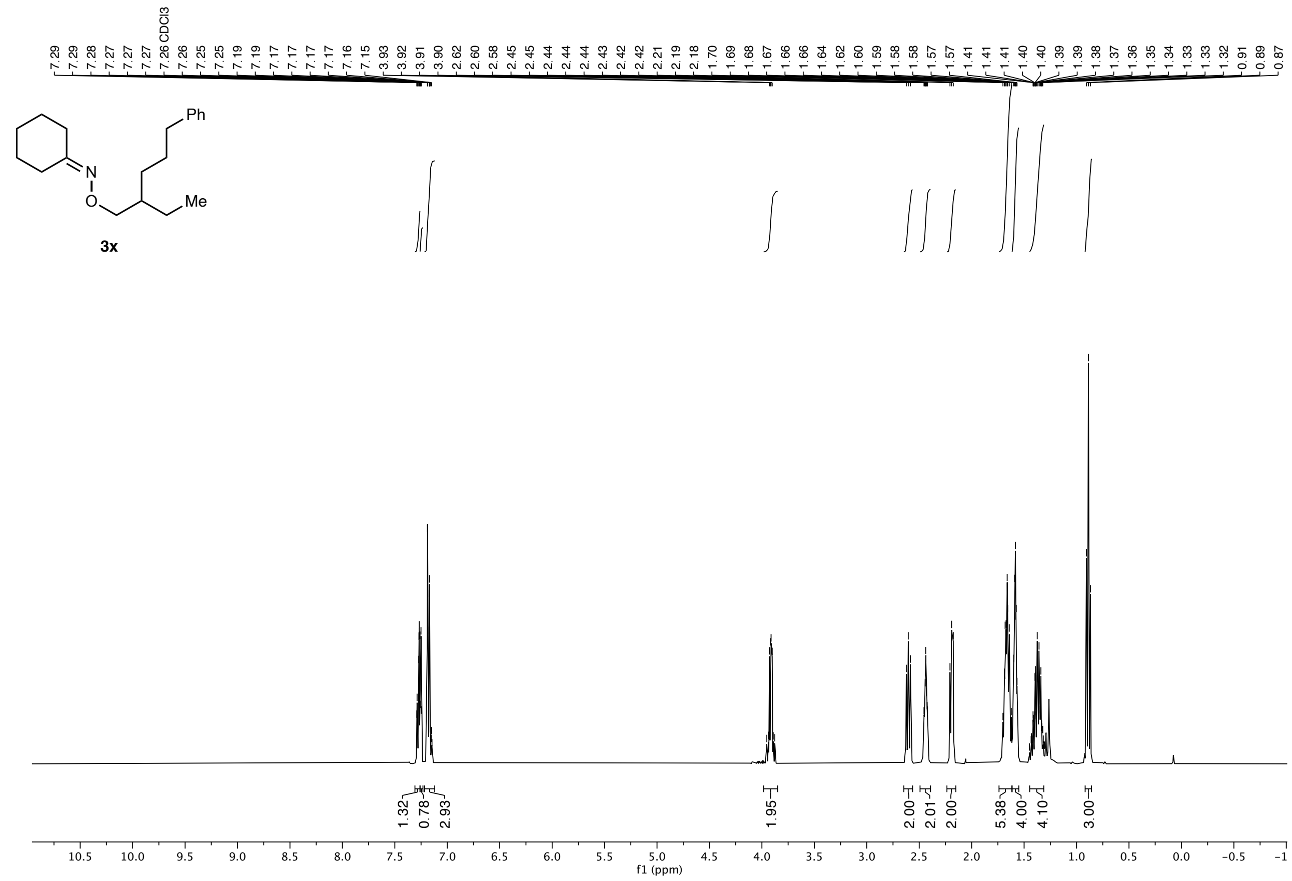
7.29 7.29 7.28 7.27 7.27 7.27 7.26 CDCl3 7.26 7.25 7.25 7.19 7.19 7.17 7.17 7.17 7.17 7.16 7.15 3.93 3.92 3.91 3.90 2.62 2.60 2.58 2.45 2.45 2.44 2.44 2.44 2.43 2.42 2.42 2.21 2.19 2.18 1.70 1.69 1.68 1.67 1.66 1.66 1.64 1.62 1.60 1.59 1.58 1.58 1.57 1.57 1.41 1.41 1.41 1.40 1.40 1.39 1.39 1.38 1.37 1.36 1.35 1.34 1.33 1.33 1.32 0.91 0.89 0.87
Ph
N
O
Me
3x
1.32 0.78 2.93 1.95 2.00 2.01 2.00 5.38 4.00 4.10 3.00
10.5 10.0 9.5 9.0 8.5 8.0 7.5 7.0 6.5 6.0 5.5 5.0 4.5 4.0 3.5 3.0 2.5 2.0 1.5 1.0 0.5 0.0 −0.5 −1
f1 (ppm)

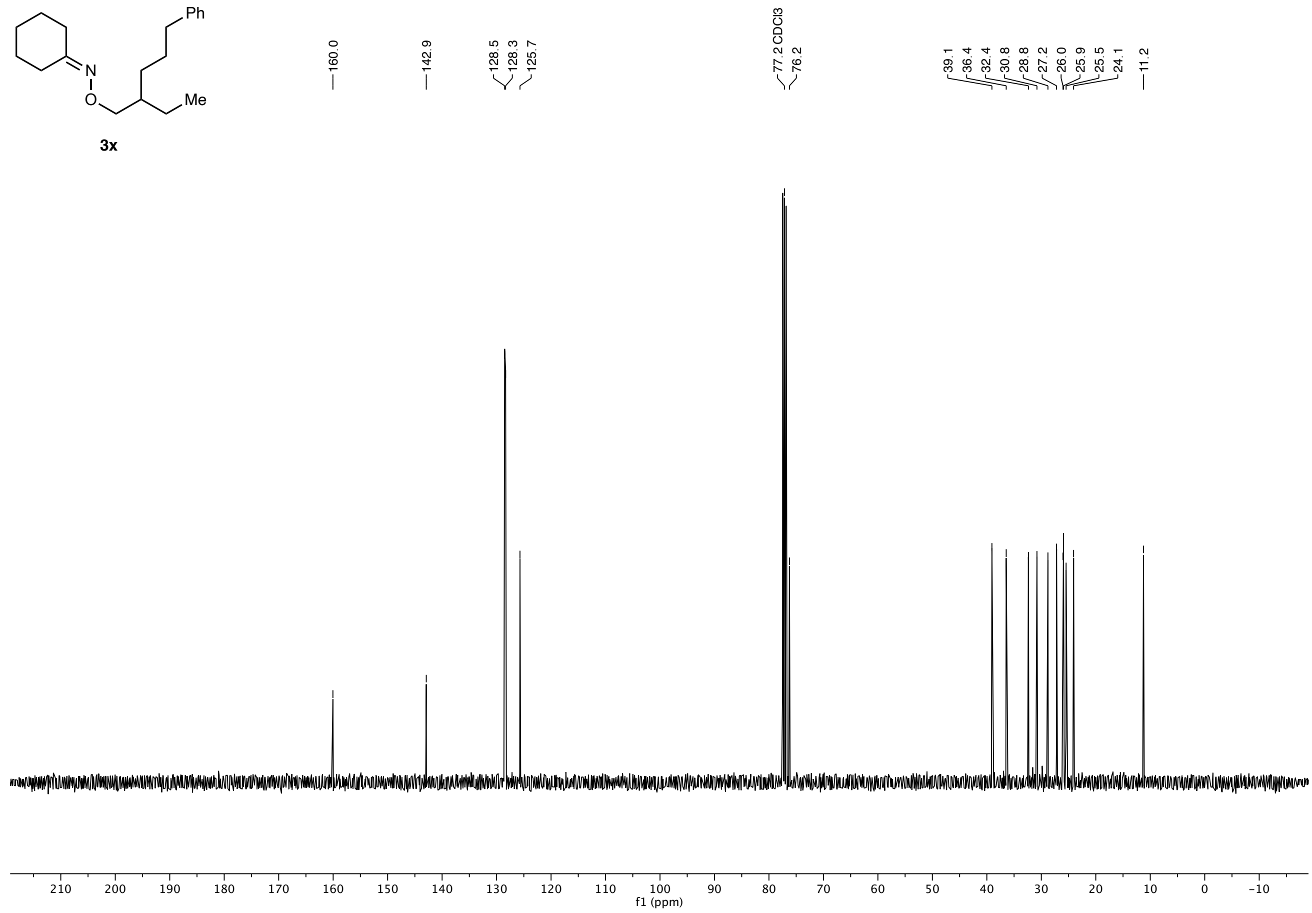
Ph
N
O
Me
3x
160.0 142.9 128.5 128.3 125.7 77.2 CDCl3 76.2 39.1 36.4 32.4 30.8 28.8 27.2 26.0 25.9 25.5 24.1 11.2
210 200 190 180 170 160 150 140 130 120 110 100 90 80 70 60 50 40 30 20 10 0 −10
f1 (ppm)

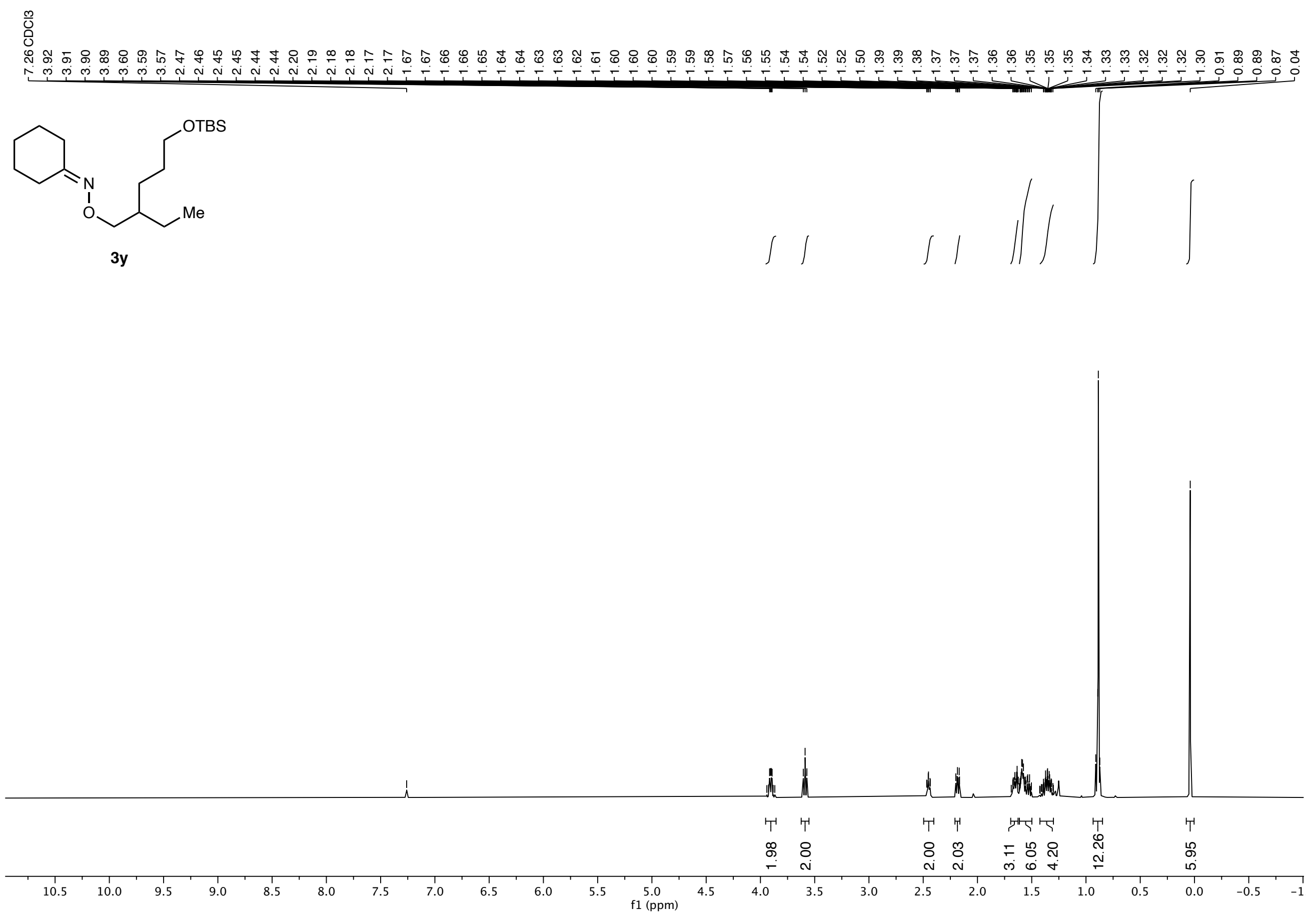
7.26 CDCl3
3.92
3.91
3.90
3.89
3.60
3.59
3.57
2.47
2.46
2.45
2.45
2.44
2.44
2.20
2.19
2.18
2.18
2.17
2.17
1.67
1.67
1.66
1.66
1.65
1.64
1.64
1.63
1.63
1.62
1.61
1.60
1.60
1.60
1.59
1.59
1.58
1.57
1.56
1.55
1.54
1.54
1.52
1.52
1.50
1.39
1.39
1.38
1.37
1.37
1.37
1.36
1.36
1.35
1.35
1.35
1.34
1.33
1.33
1.32
1.32
1.32
1.30
0.91
0.89
0.87
0.04
OTBS
N
O
Me
3y
1.98
2.00
2.00
2.03
3.11
6.05
4.20
12.26
5.95
f1 (ppm)

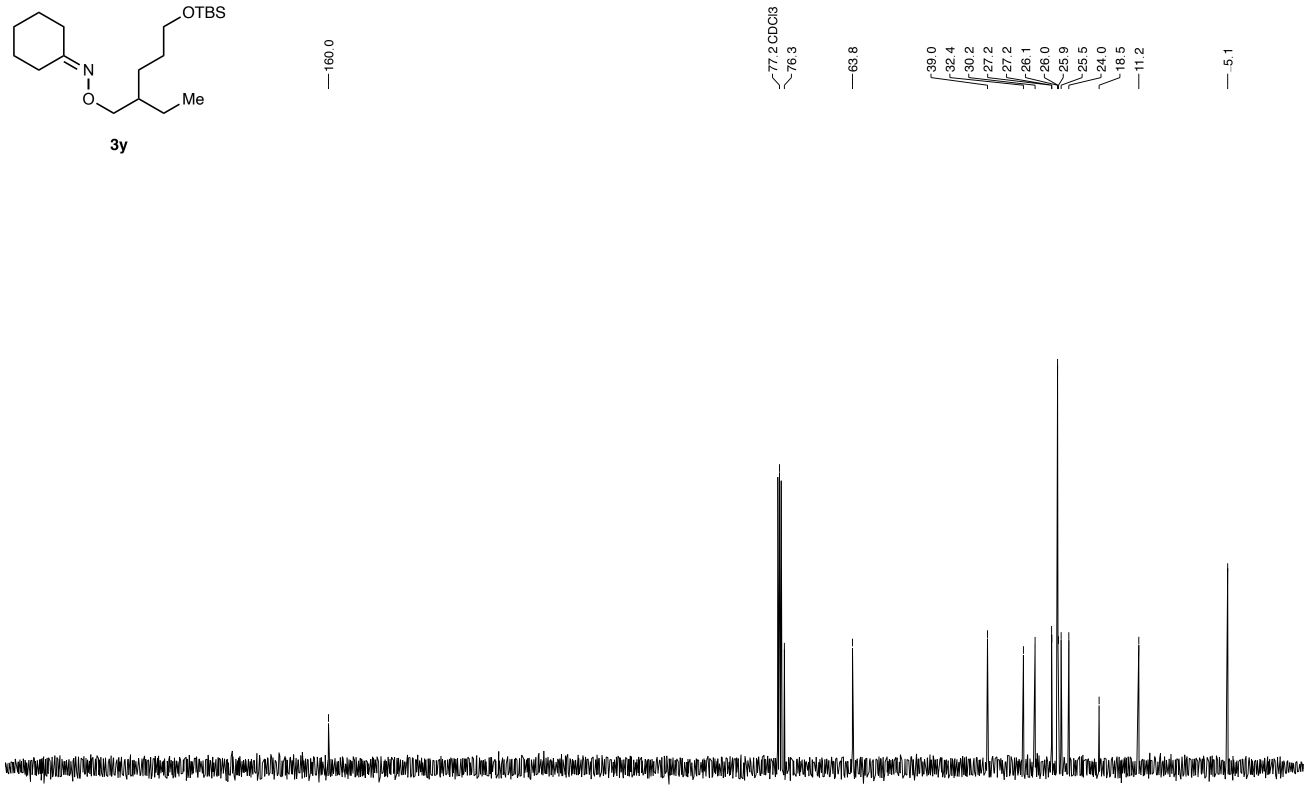
OTBS
N
O
Me
3y
160.0
77.2 CDCl3
76.3
63.8
39.0
32.4
30.2
27.2
27.2
26.1
26.0
25.9
25.5
24.0
18.5
11.2
-5.1
f1 (ppm)

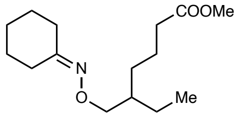
COOMe
N
O
Me

3z

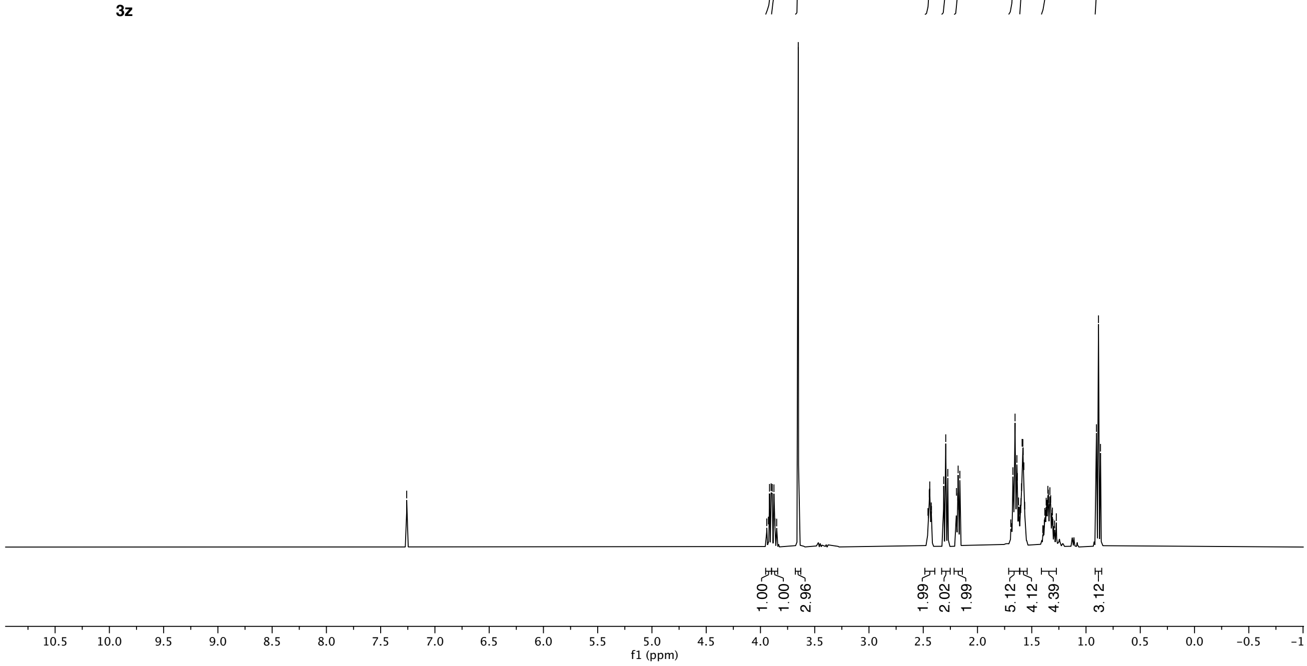
7.26 CDCl3
1.00
1.00
2.96
1.99
2.02
1.99
5.12
4.12
4.39
3.12
f1 (ppm)

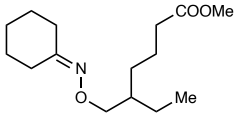
COOMe
N
O
Me

3z

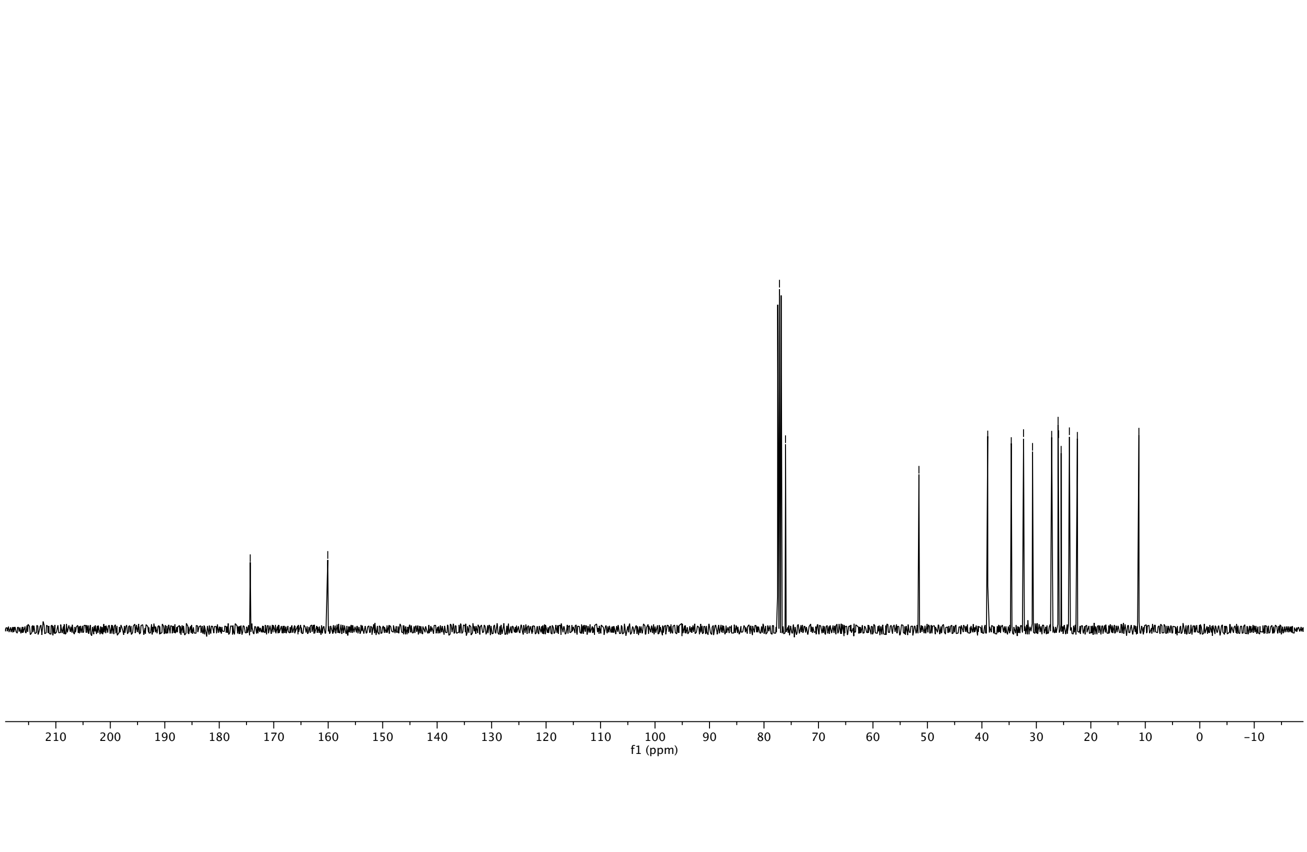
174.3
160.1
77.2 CDCl3
76.0
51.6
38.9
34.6
32.3
30.7
27.2
26.0
25.9
25.4
23.9
22.5
11.2
f1 (ppm)

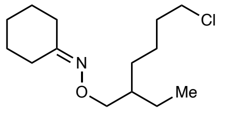
N
O
Cl
Me

3aa

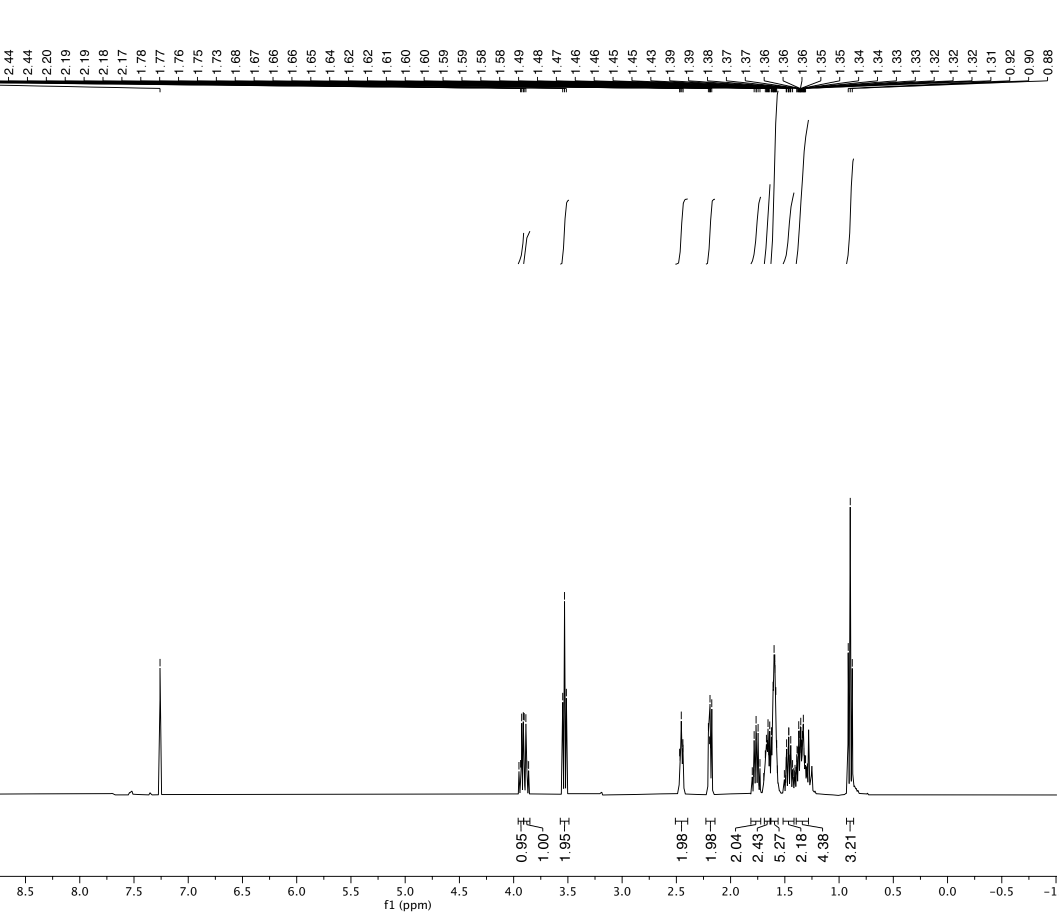
7.26 CDCl3
3.94
3.93
3.91
3.90
3.89
3.55
3.53
3.51
2.47
2.47
2.45
2.44
2.44
2.20
2.19
2.19
2.18
2.17
1.78
1.77
1.76
1.75
1.73
1.68
1.67
1.66
1.66
1.65
1.64
1.62
1.62
1.61
1.60
1.60
1.59
1.59
1.58
1.58
1.49
1.48
1.47
1.46
1.46
1.45
1.45
1.43
1.39
1.39
1.38
1.37
1.37
1.36
1.36
1.36
1.35
1.35
1.34
1.34
1.33
1.33
1.32
1.32
1.32
1.31
0.92
0.90
0.88
0.95
1.00
1.95
1.98
1.98
2.04
2.43
5.27
2.18
4.38
3.21
10.5
10.0
9.5
9.0
8.5
8.0
7.5
7.0
6.5
6.0
5.5
5.0
4.5
4.0
3.5
3.0
2.5
2.0
1.5
1.0
0.5
0.0
−0.5
−1
f1 (ppm)

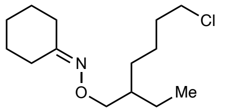
N
O
Cl
Me

3aa

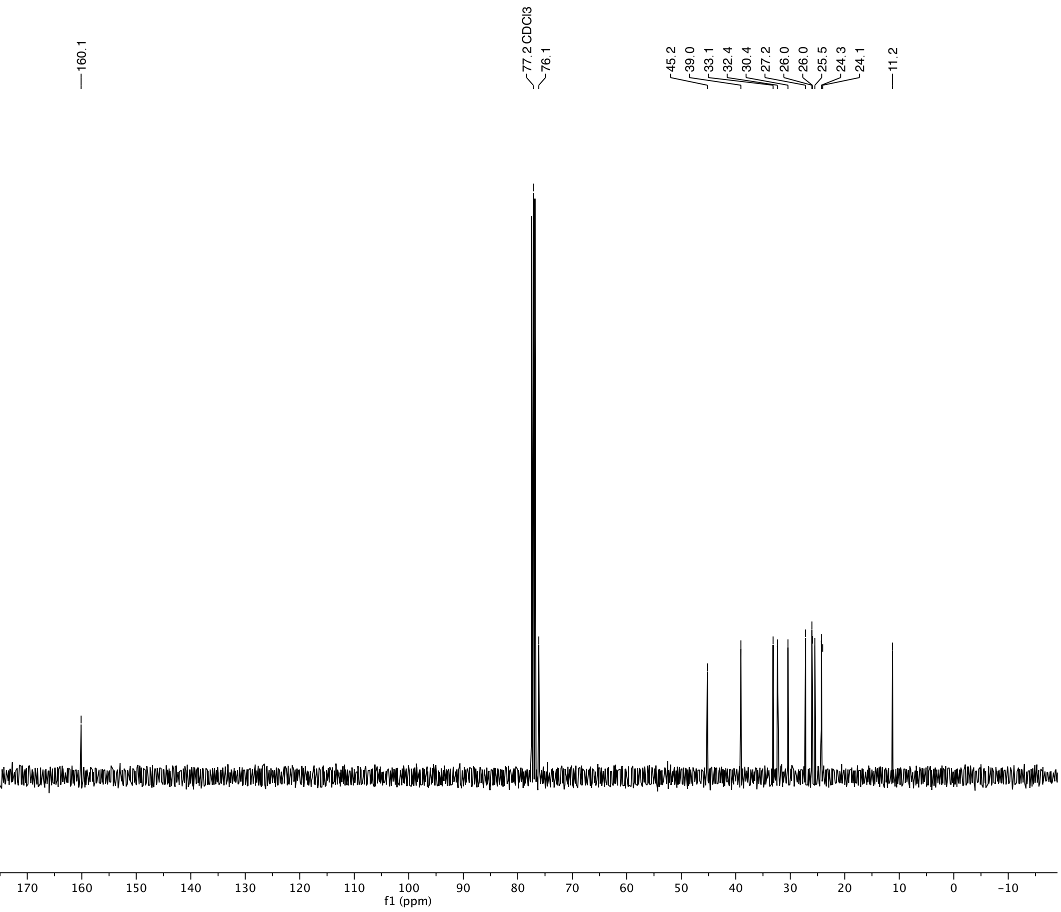
160.1
77.2 CDCl3
76.1
45.2
39.0
33.1
32.4
30.4
27.2
26.0
26.0
25.5
24.3
24.1
11.2
210
200
190
180
170
160
150
140
130
120
110
100
90
80
70
60
50
40
30
20
10
0
−10
f1 (ppm)

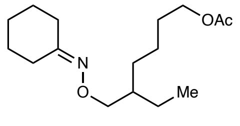
OAc
N
O
Me

3ab

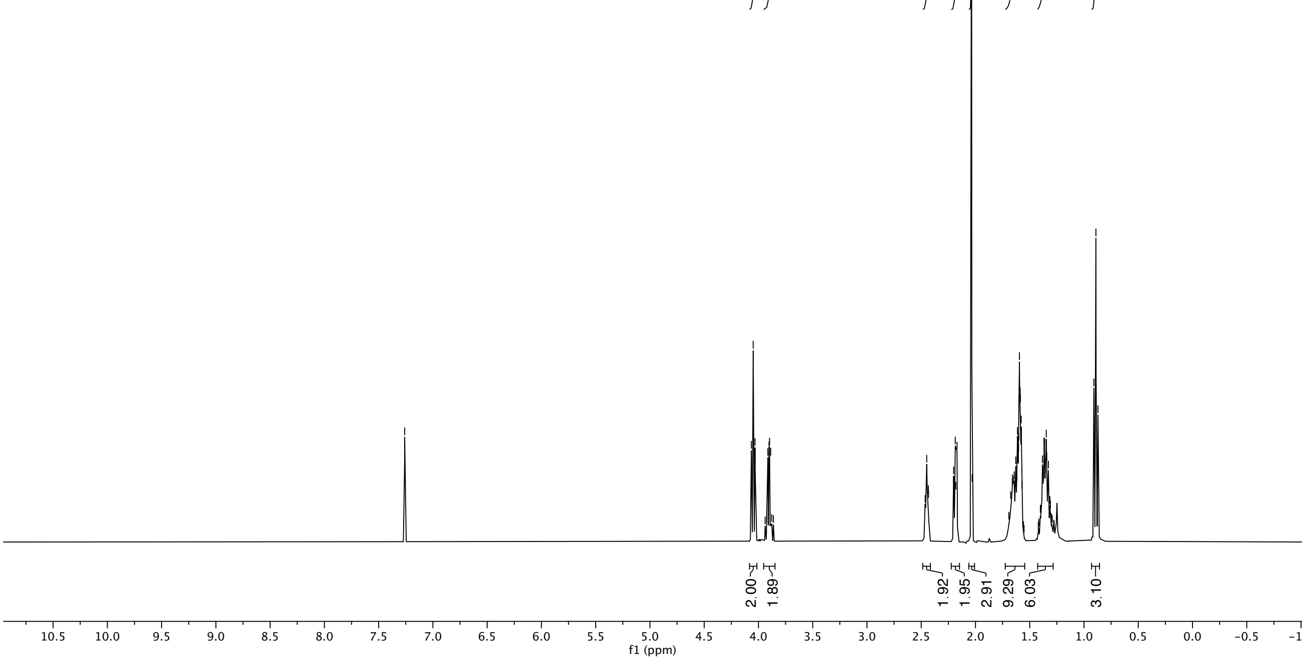
7.26 CDCl3
4.07
4.05
4.03
3.94
3.92
3.91
3.90
3.90
3.89
3.88
3.86
2.46
2.45
2.43
2.43
2.20
2.19
2.18
2.17
2.04
2.03
1.69
1.68
1.66
1.65
1.64
1.63
1.62
1.61
1.61
1.60
1.60
1.59
1.59
1.59
1.58
1.57
1.56
1.55
1.42
1.40
1.40
1.38
1.38
1.37
1.37
1.36
1.36
1.35
1.34
1.33
1.33
1.31
1.31
1.30
1.29
0.91
0.89
0.87
2.00
1.89
1.92
1.95
2.91
9.29
6.03
3.10
f1 (ppm)

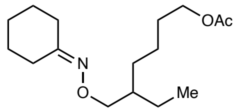
OAc
N
O
Me

3ab

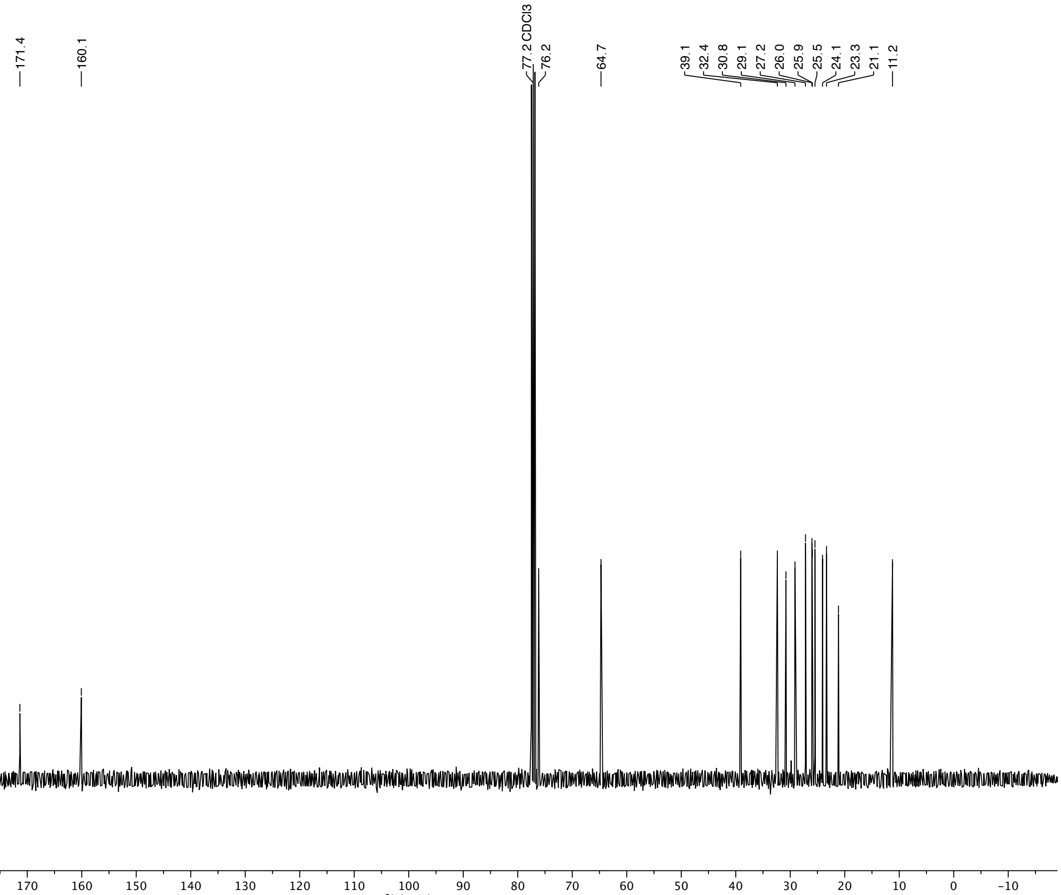
171.4
160.1
77.2 CDCl3
76.2
64.7
39.1
32.4
30.8
29.1
27.2
26.0
25.9
25.5
24.1
23.3
21.1
11.2
f1 (ppm)

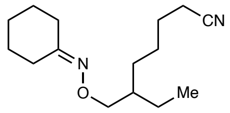
CN
N
O
Me

3ac

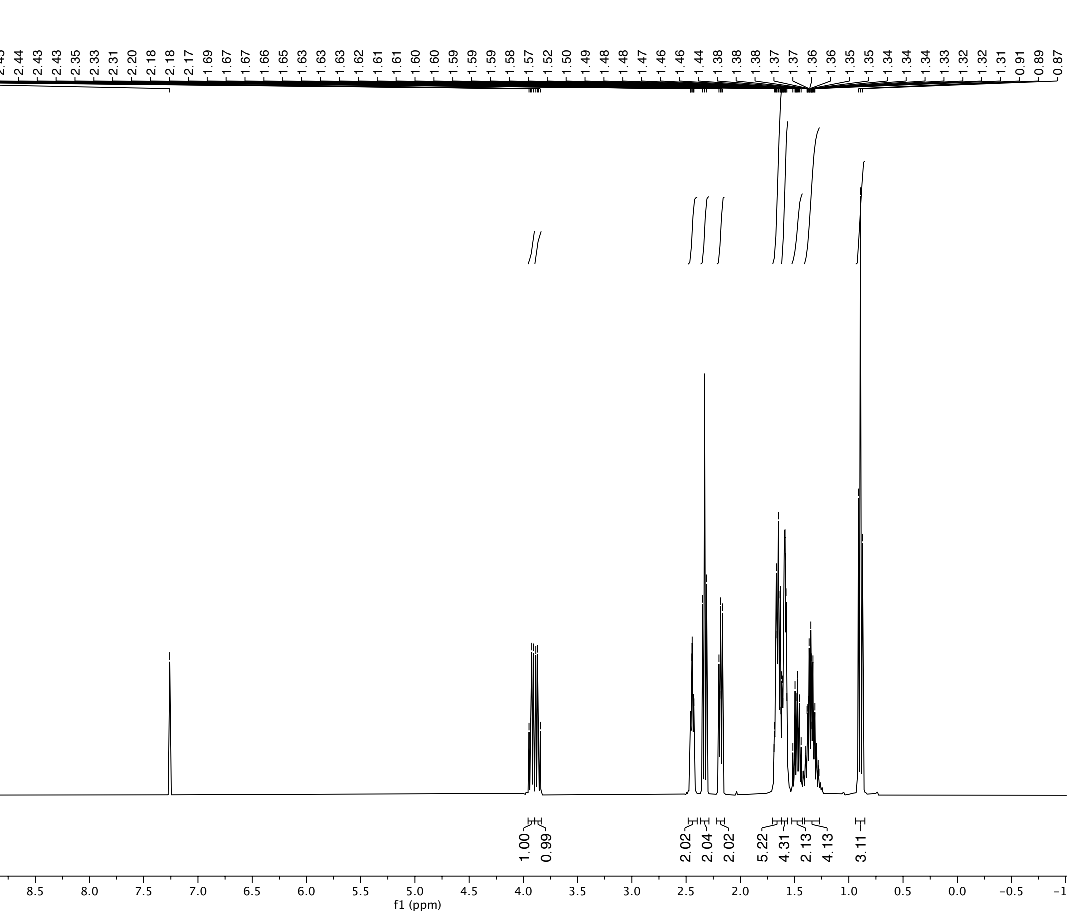
7.26 CDCl3
3.95
3.93
3.92
3.91
3.89
3.87
3.86
3.84
2.46
2.46
2.45
2.44
2.43
2.43
2.35
2.33
2.31
2.20
2.18
2.18
2.17
1.69
1.67
1.67
1.66
1.65
1.63
1.63
1.63
1.62
1.61
1.61
1.60
1.60
1.59
1.59
1.59
1.58
1.57
1.52
1.50
1.49
1.48
1.48
1.47
1.46
1.46
1.44
1.38
1.38
1.38
1.37
1.37
1.36
1.36
1.35
1.35
1.34
1.34
1.34
1.33
1.32
1.32
1.31
0.91
0.89
0.87
1.00
0.99
2.02
2.04
2.02
5.22
4.31
2.13
4.13
3.11
f1 (ppm)

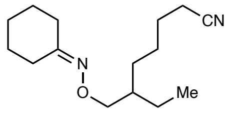
CN
N
O
Me

3ac

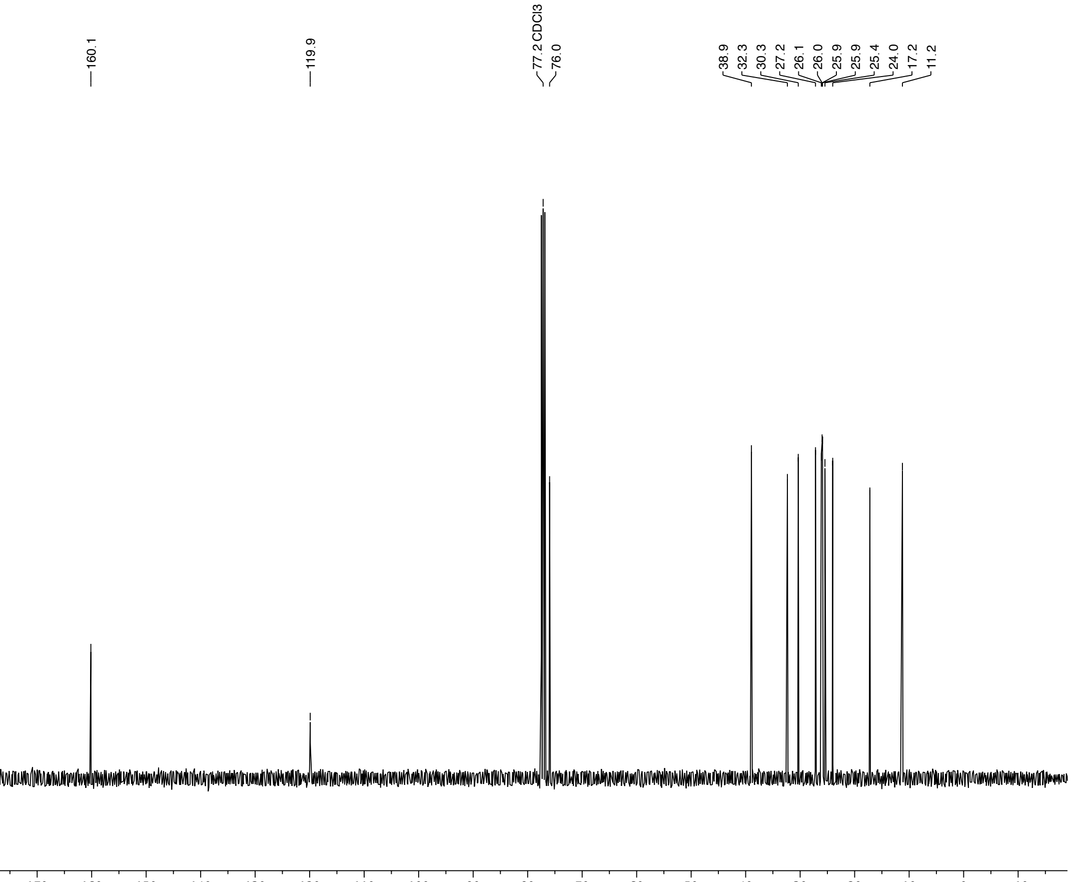
160.1
119.9
77.2 CDCl3
76.0
38.9
32.3
30.3
27.2
26.1
26.0
25.9
25.9
25.4
24.0
17.2
11.2
f1 (ppm)

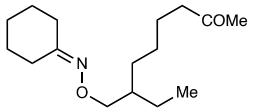
COMe
N
O
Me
3ad

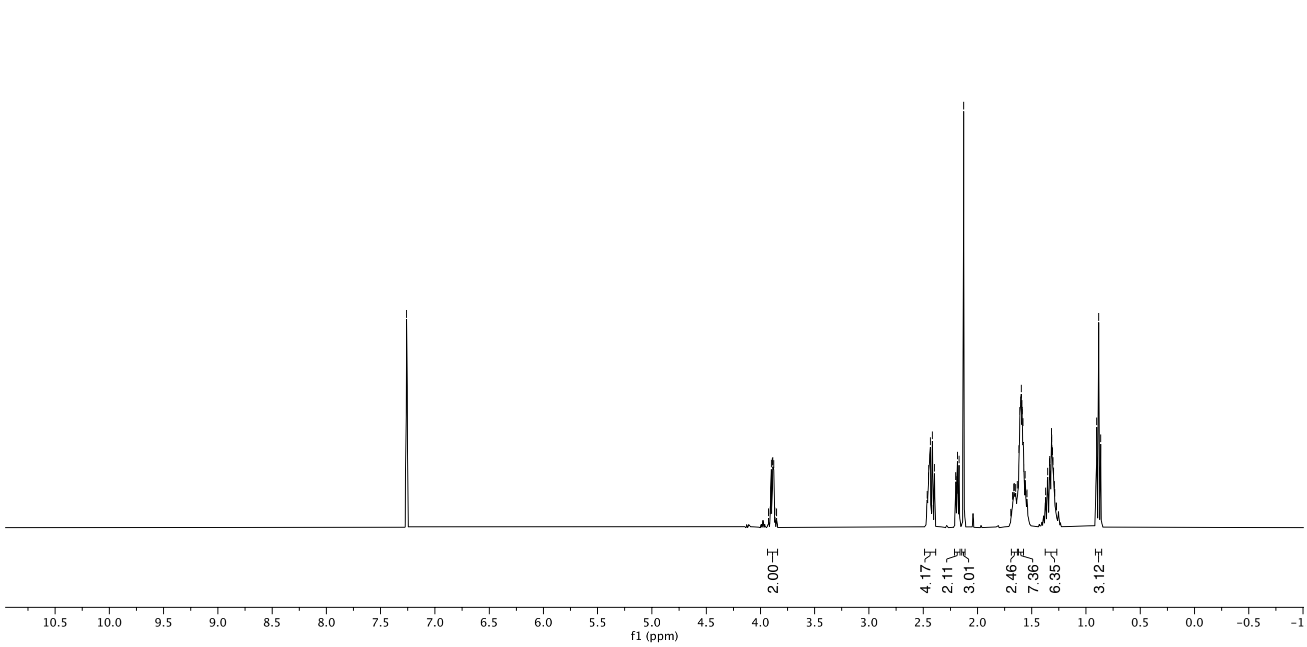
7.26 CDCl3
3.93
3.91
3.90
3.89
3.89
3.88
3.87
3.85
2.46
2.45
2.45
2.43
2.42
2.41
2.40
2.20
2.19
2.19
2.18
2.18
2.17
2.13
1.69
1.68
1.67
1.66
1.65
1.63
1.62
1.61
1.60
1.60
1.59
1.59
1.58
1.57
1.57
1.56
1.55
1.54
1.38
1.37
1.36
1.35
1.34
1.33
1.33
1.32
1.32
1.31
1.31
1.30
1.30
1.29
1.29
1.27
1.27
0.90
0.88
0.86
2.00
4.17
2.11
3.01
2.46
7.36
6.35
3.12
f1 (ppm)

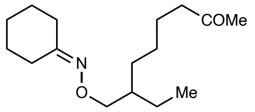
COMe
N
O
Me
3ad

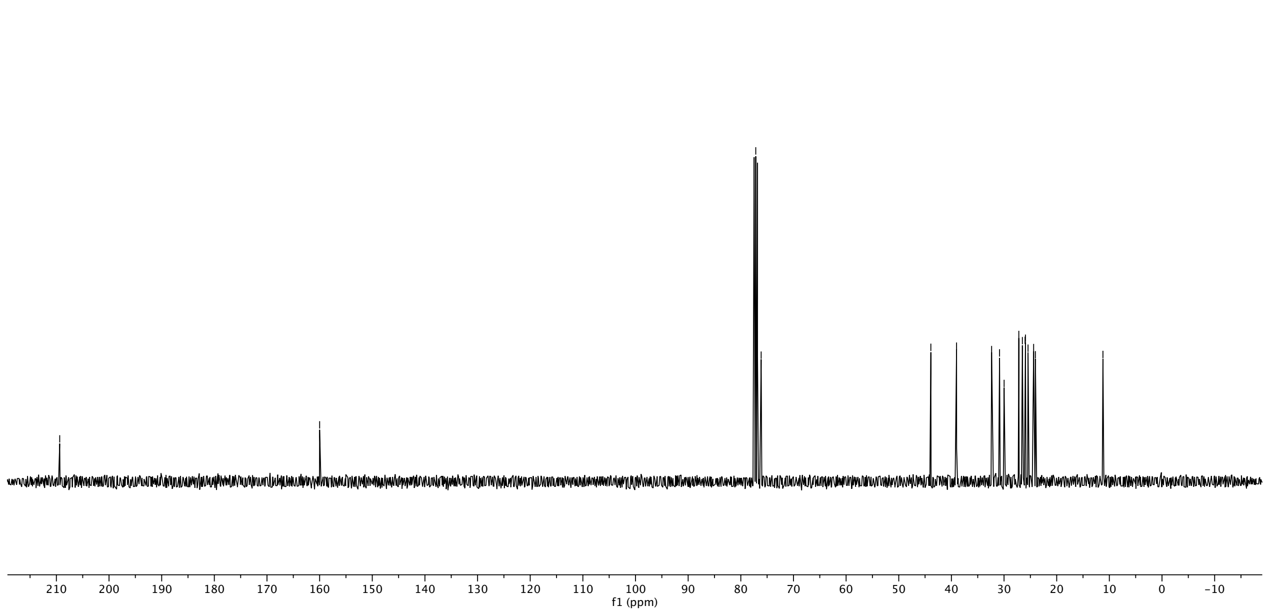
209.4
160.0
77.2 CDCl3
76.1
43.9
39.0
32.4
30.9
30.0
27.2
26.5
26.0
25.9
25.4
24.4
24.0
11.2
f1 (ppm)

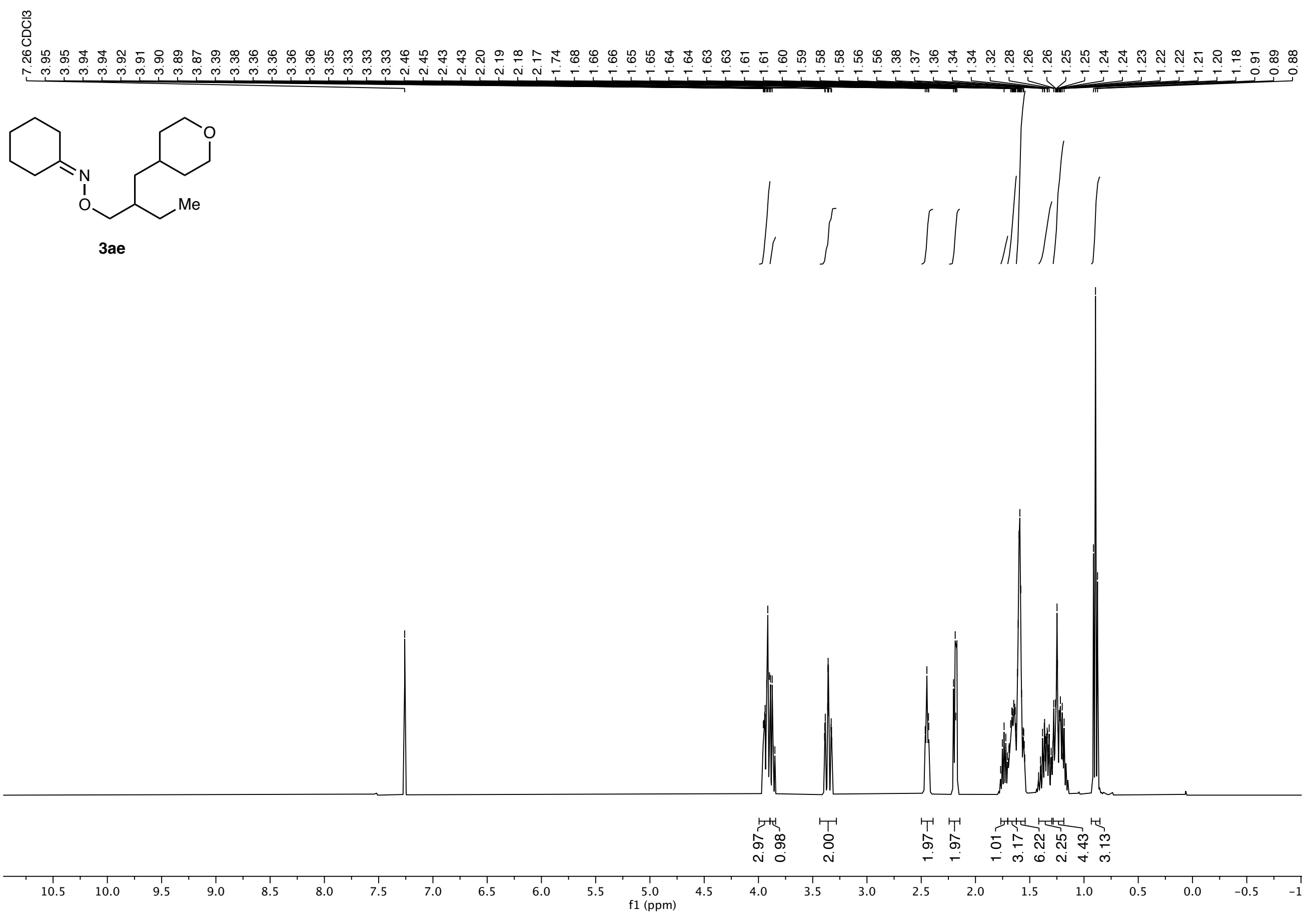
7.26 CDCl3
3.95 3.95 3.94 3.94 3.92 3.91 3.90 3.89 3.87 3.39 3.38 3.36 3.36 3.36 3.36 3.35 3.33 3.33 3.33 2.46 2.45 2.43 2.43 2.20 2.19 2.18 2.17 1.74 1.68 1.66 1.66 1.65 1.65 1.64 1.64 1.63 1.63 1.61 1.61 1.60 1.59 1.58 1.58 1.56 1.56 1.38 1.37 1.36 1.34 1.34 1.32 1.28 1.26 1.26 1.25 1.25 1.24 1.24 1.23 1.22 1.22 1.21 1.20 1.18 0.91 0.89 0.88
N
O
O
Me
3ae
2.97
0.98
2.00
1.97
1.97
1.01
3.17
6.22
2.25
4.43
3.13
10.5 10.0 9.5 9.0 8.5 8.0 7.5 7.0 6.5 6.0 5.5 5.0 4.5 4.0 3.5 3.0 2.5 2.0 1.5 1.0 0.5 0.0 −0.5 −1
f1 (ppm)

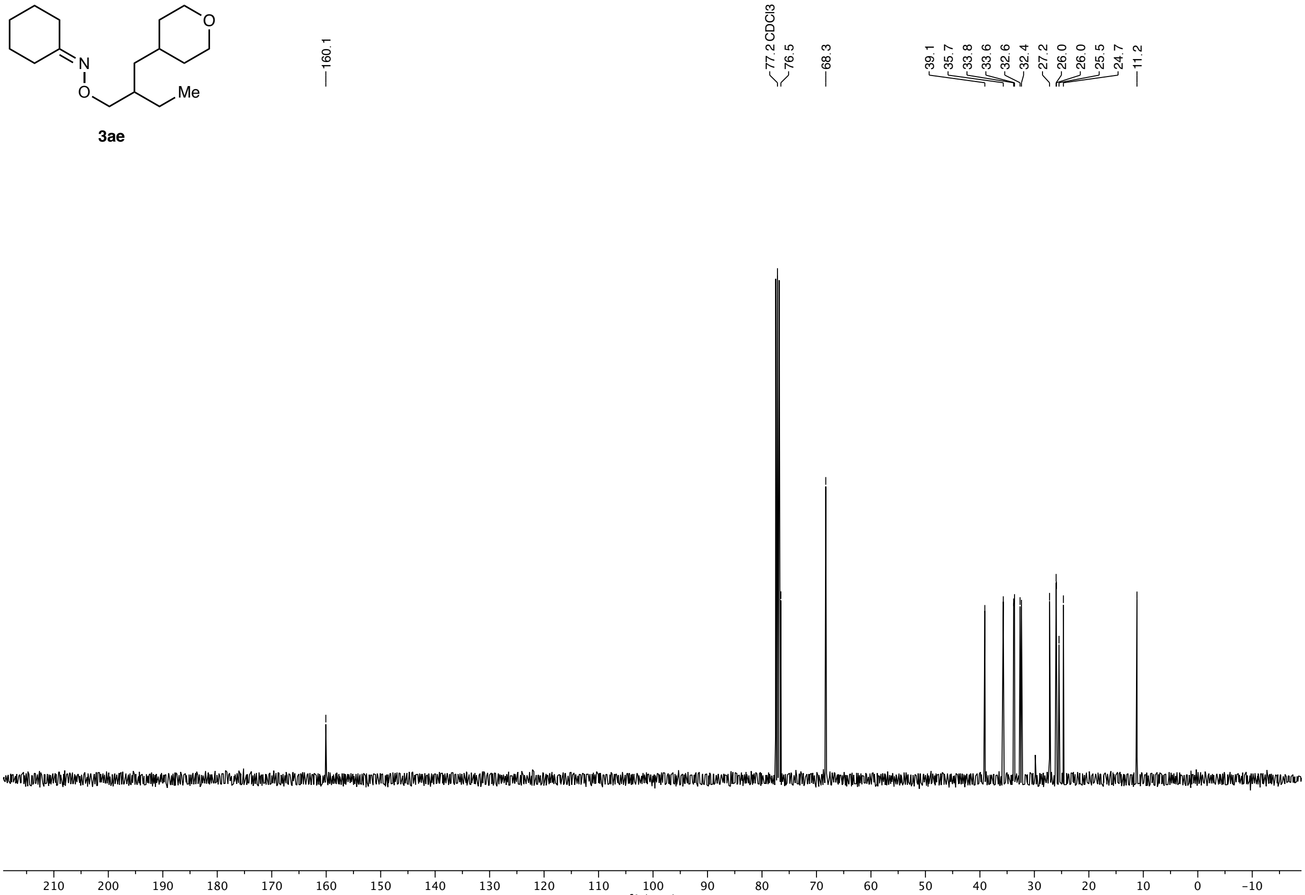
N
O
O
Me
3ae
160.1
77.2 CDCl3
76.5
68.3
39.1
35.7
33.8
33.6
32.6
32.4
27.2
26.0
26.0
25.5
24.7
11.2
210 200 190 180 170 160 150 140 130 120 110 100 90 80 70 60 50 40 30 20 10 0 −10
f1 (ppm)

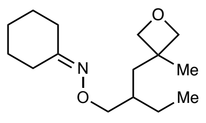

3af

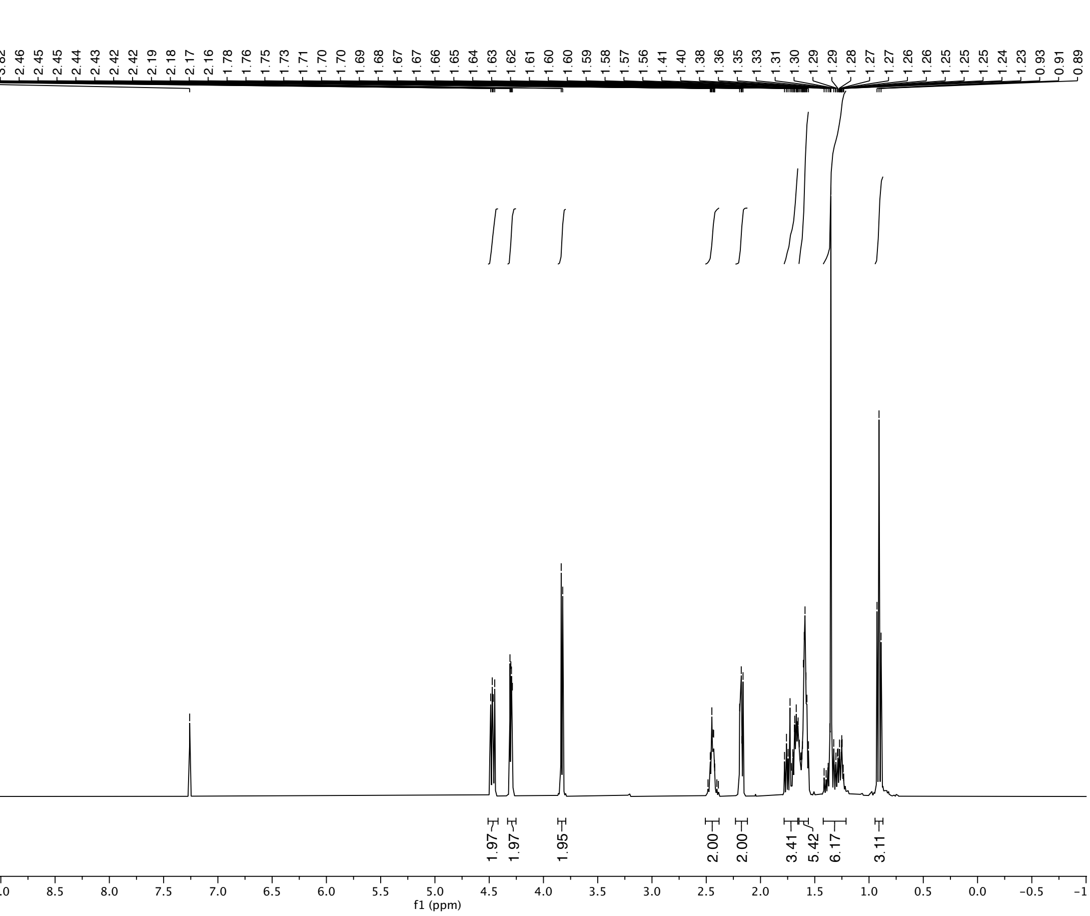

7.26 CDCl3
4.49 4.47 4.46 4.45 4.31 4.30 4.29 4.29 3.84 3.82 2.46 2.45 2.45 2.44 2.43 2.42 2.42 2.19 2.18 2.17 2.16 1.78 1.76 1.75 1.73 1.71 1.70 1.70 1.69 1.68 1.67 1.67 1.66 1.65 1.64 1.63 1.62 1.61 1.60 1.60 1.59 1.58 1.57 1.56 1.41 1.40 1.38 1.36 1.35 1.33 1.31 1.30 1.29 1.29 1.28 1.27 1.27 1.26 1.26 1.25 1.25 1.25 1.24 1.23 0.93 0.91 0.89
1.97 1.97 1.95 2.00 2.00 3.41 5.42 6.17 3.11
f1 (ppm)

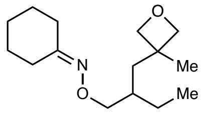

3af

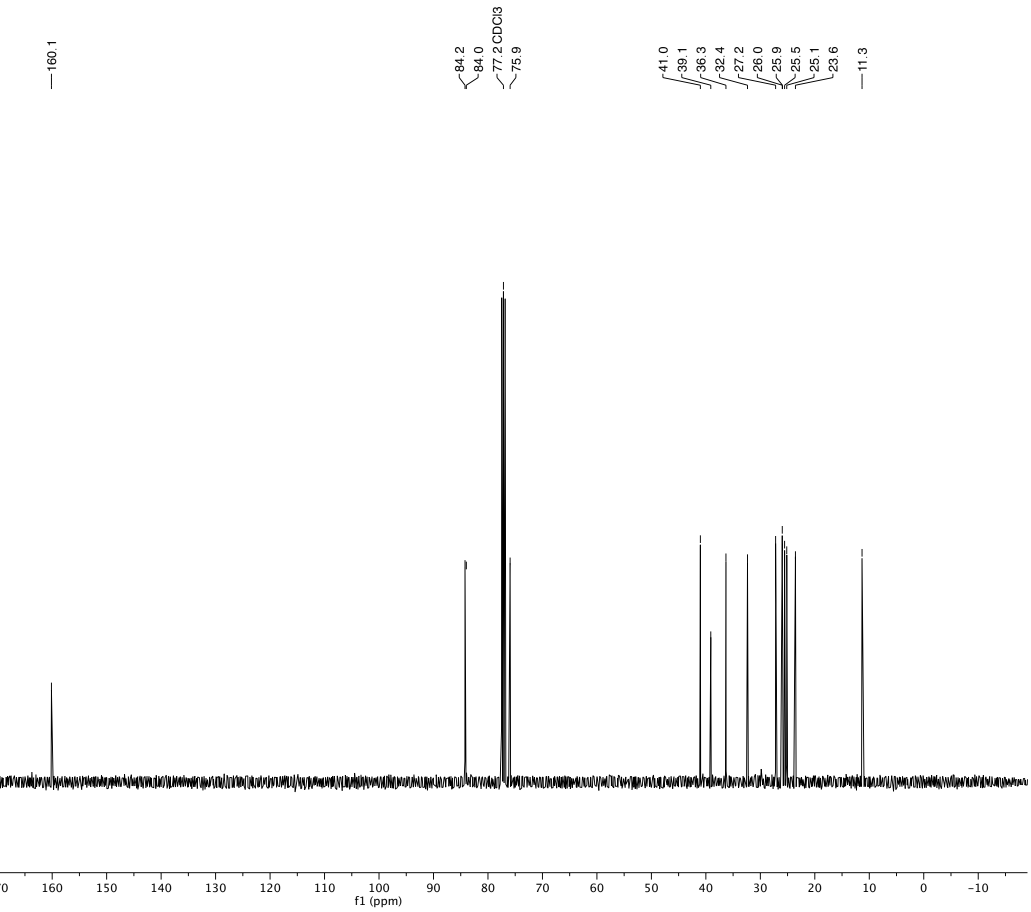

160.1
84.2 84.0 77.2 CDCl3 75.9
41.0 39.1 36.3 32.4 27.2 26.0 25.9 25.5 25.1 23.6
11.3
f1 (ppm)

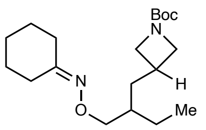
Boc
N
H
N
O
Me

3ag

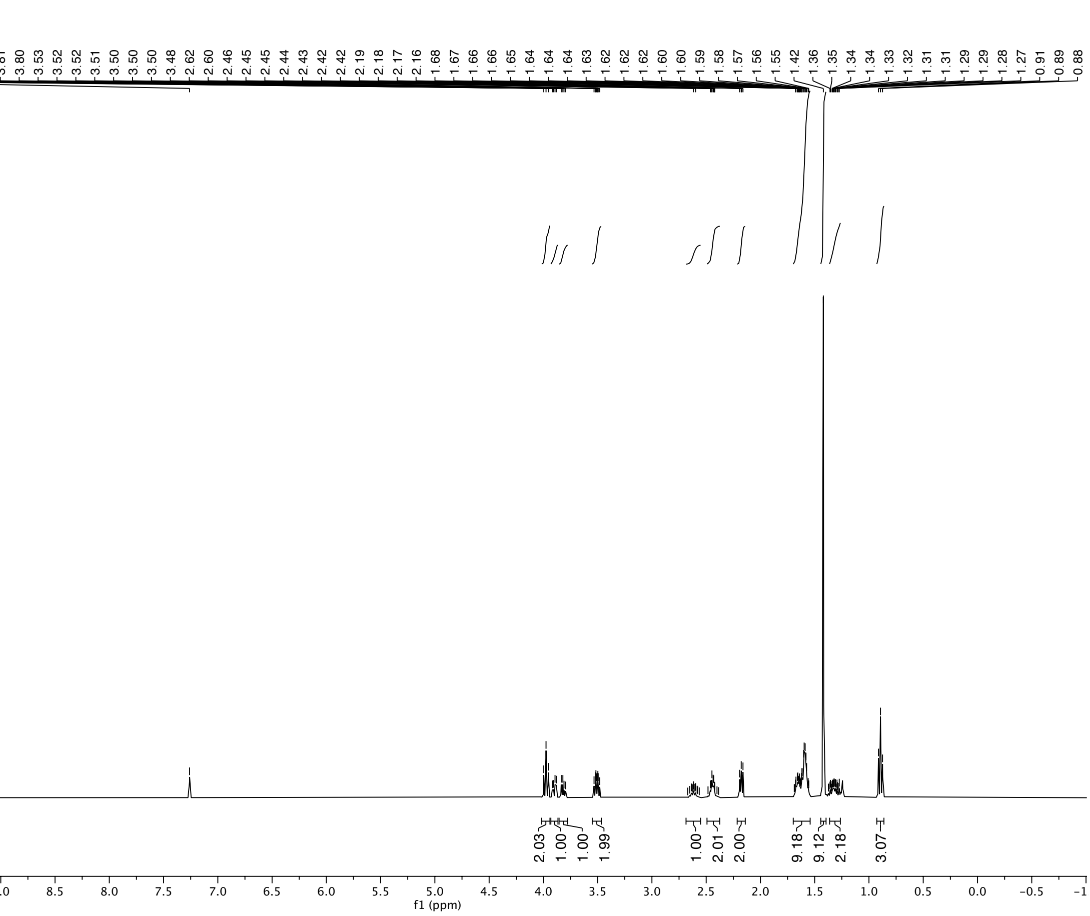
7.26 CDCl3
4.00 3.98 3.96 3.92 3.91 3.89 3.88 3.84 3.82 3.81 3.80 3.53 3.52 3.52 3.51 3.50 3.50 3.50 3.48 2.62 2.60 2.46 2.45 2.45 2.44 2.43 2.42 2.42 2.19 2.18 2.17 2.16 1.68 1.67 1.66 1.66 1.65 1.64 1.64 1.64 1.63 1.62 1.62 1.62 1.60 1.60 1.59 1.58 1.57 1.56 1.55 1.42 1.36 1.35 1.34 1.34 1.33 1.32 1.31 1.31 1.29 1.29 1.28 1.27 0.91 0.89 0.88
2.03 1.00 1.00 1.99 1.00 2.01 2.00 9.18 9.12 2.18 3.07
f1 (ppm)

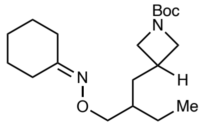
Boc
N
H
N
O
Me

3ag

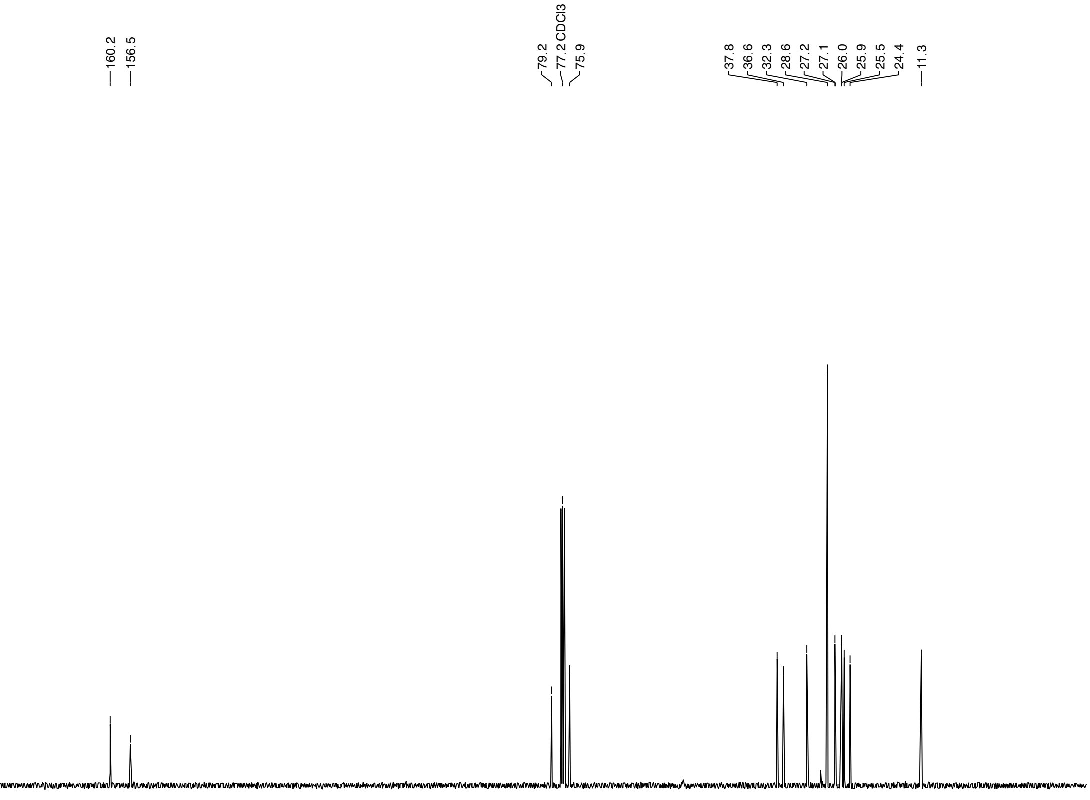
160.2
156.5
79.2
77.2 CDCl3
75.9
37.8 36.6 32.3 28.6 27.2 27.1 26.0 25.9 25.5 24.4
11.3
f1 (ppm)

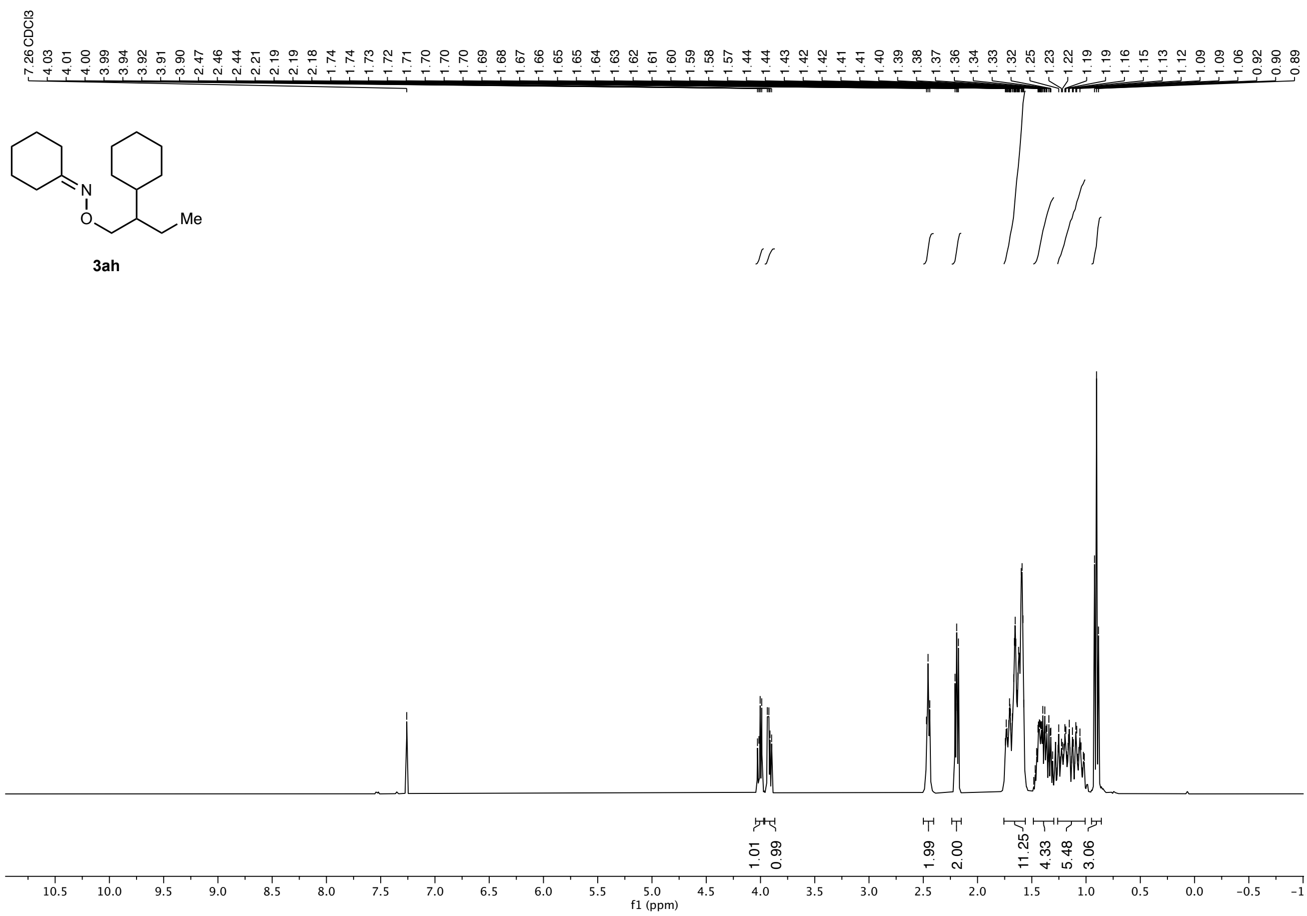
3ah
Me
N
O
7.26 CDCl3
1.01
0.99
1.99
2.00
11.25
4.33
5.48
3.06
f1 (ppm)

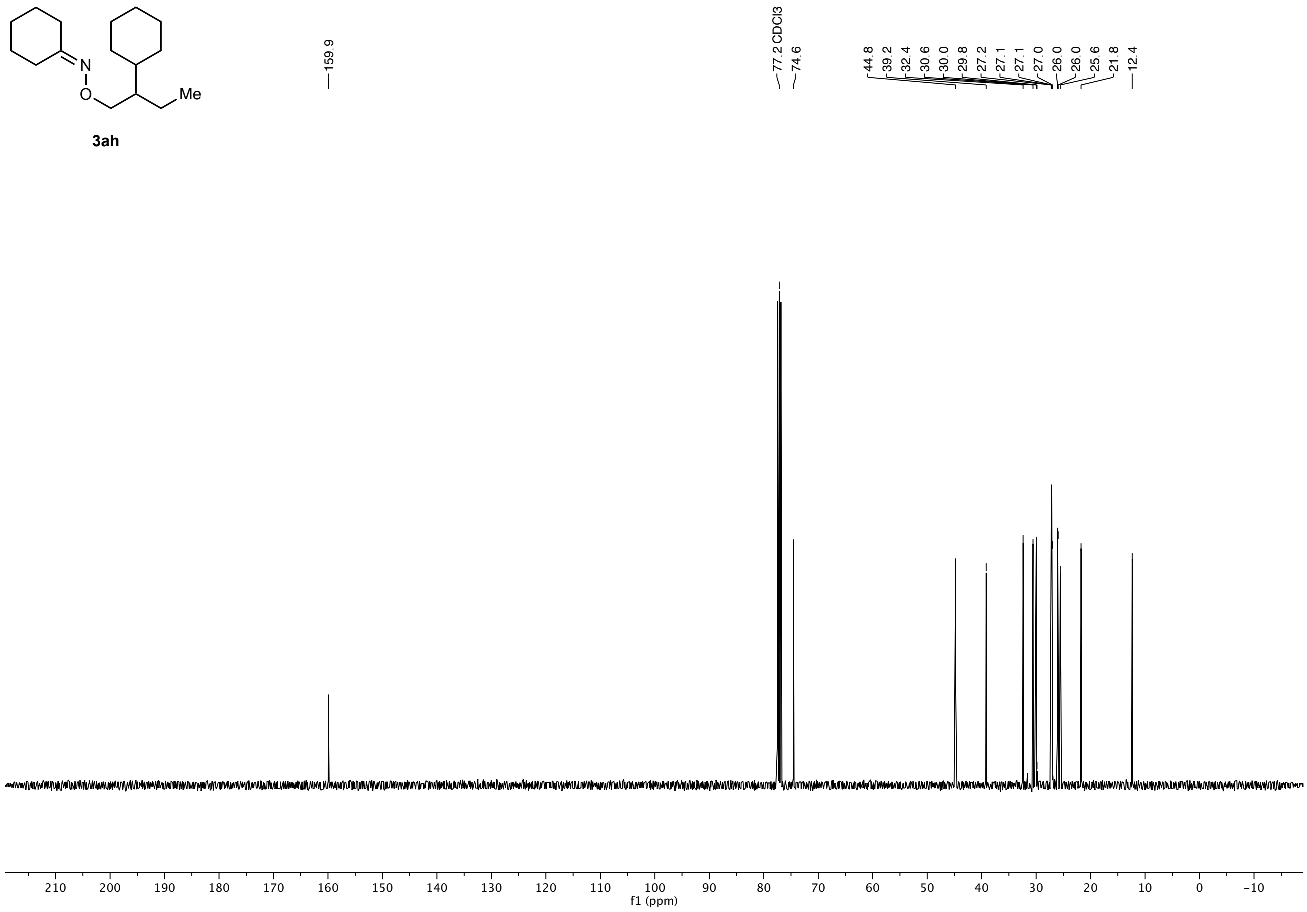
3ah
Me
N
O
159.9
77.2 CDCl3
74.6
44.8
39.2
32.4
30.6
30.0
29.8
27.2
27.1
27.1
27.0
26.0
26.0
25.6
21.8
12.4
f1 (ppm)

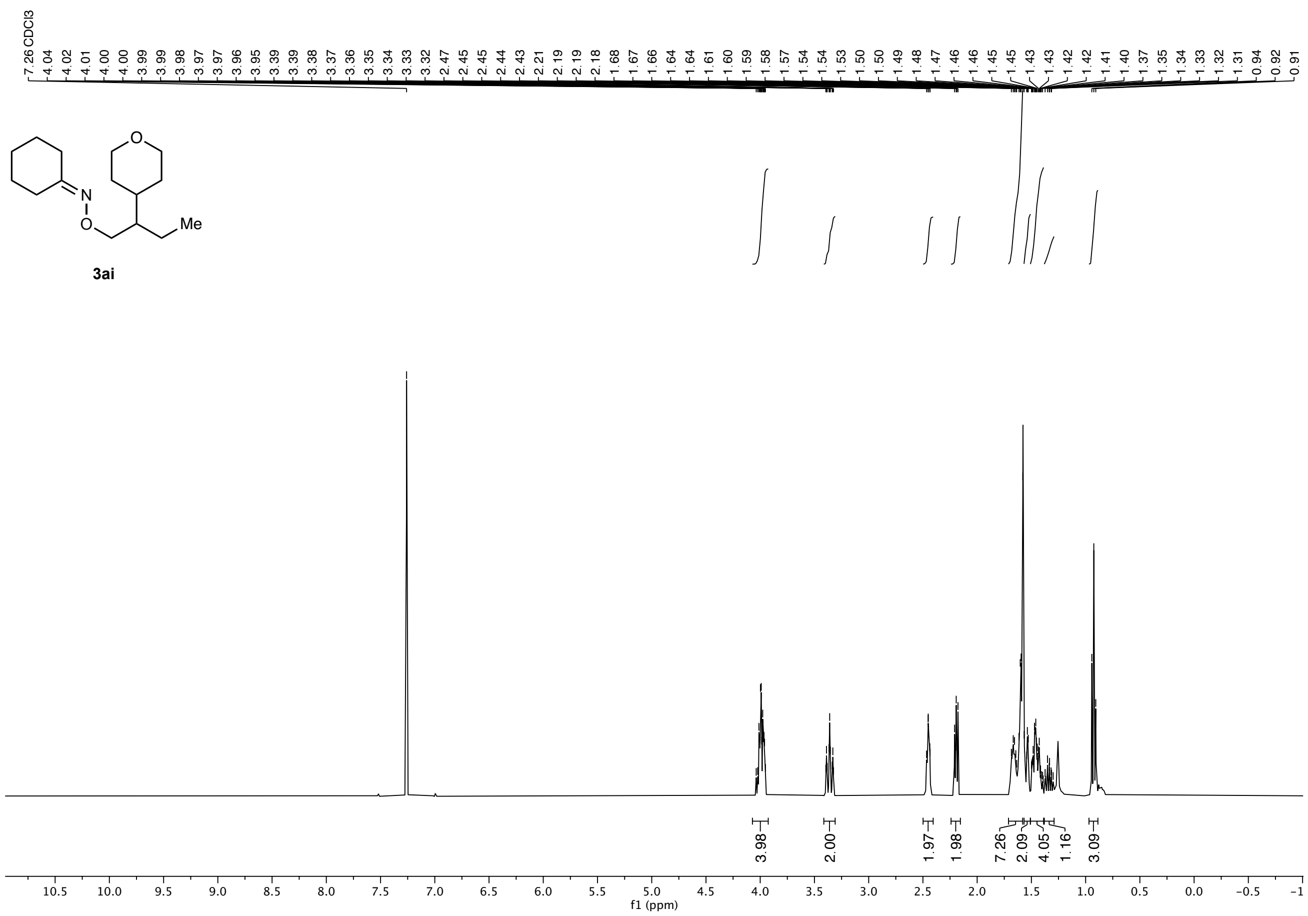
7.26 CDCl3
4.04
4.02
4.01
4.00
4.00
3.99
3.99
3.98
3.97
3.97
3.96
3.95
3.39
3.39
3.38
3.37
3.36
3.35
3.34
3.33
3.32
2.47
2.45
2.45
2.44
2.43
2.21
2.19
2.19
2.18
1.68
1.67
1.66
1.64
1.64
1.61
1.60
1.59
1.58
1.57
1.54
1.54
1.53
1.50
1.50
1.49
1.48
1.47
1.46
1.46
1.45
1.45
1.43
1.43
1.42
1.42
1.41
1.40
1.37
1.35
1.34
1.33
1.32
1.31
0.94
0.92
0.91
N
O
O
Me
3ai
3.98
2.00
1.97
1.98
7.26
2.09
4.05
1.16
3.09
10.5
10.0
9.5
9.0
8.5
8.0
7.5
7.0
6.5
6.0
5.5
5.0
4.5
4.0
3.5
3.0
2.5
2.0
1.5
1.0
0.5
0.0
−0.5
−1
f1 (ppm)

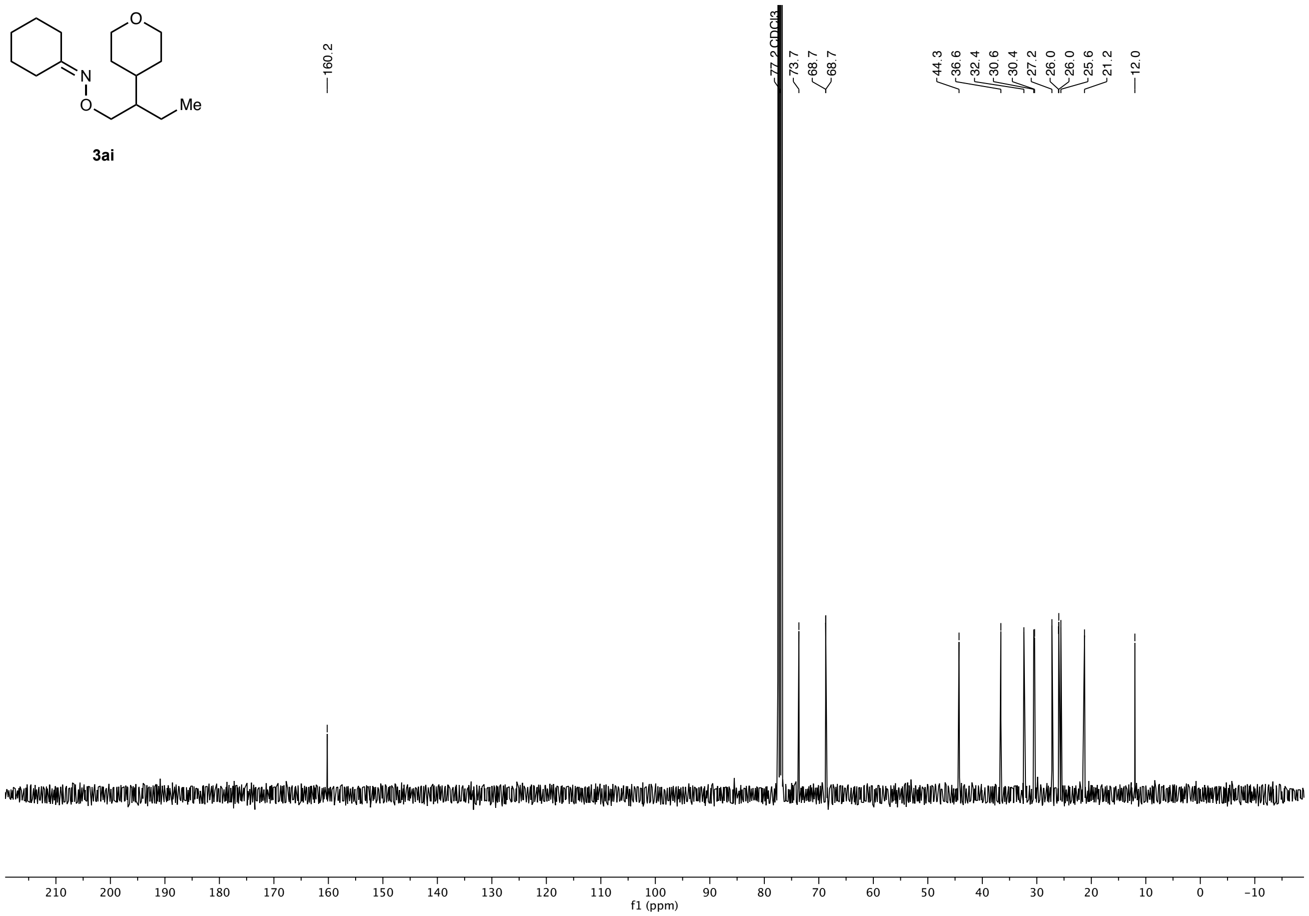
N
O
O
Me
3ai
160.2
77.2 CDCl3
73.7
68.7
68.7
44.3
36.6
32.4
30.6
30.4
27.2
26.0
26.0
25.6
21.2
12.0
210
200
190
180
170
160
150
140
130
120
110
100
90
80
70
60
50
40
30
20
10
0
−10
f1 (ppm)

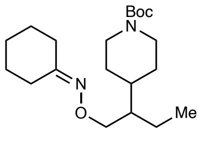

Boc
N
O
Me

3aj

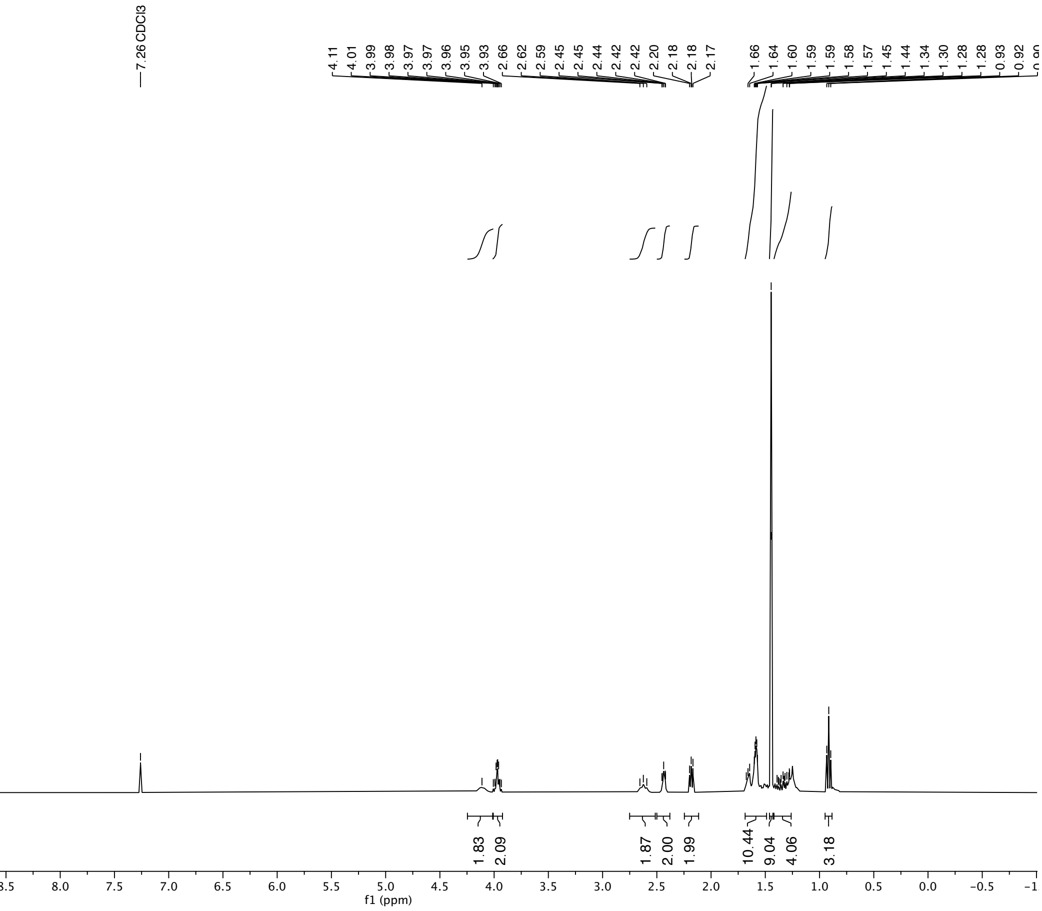

7.26 CDCl3
4.11
4.01
3.99
3.98
3.97
3.97
3.96
3.95
3.93
2.66
2.62
2.59
2.45
2.45
2.44
2.42
2.42
2.20
2.18
2.18
2.17
1.66
1.64
1.60
1.59
1.59
1.58
1.57
1.45
1.44
1.34
1.30
1.28
1.28
0.93
0.92
1.83
2.09
1.87
2.00
1.99
10.44
9.04
4.06
3.18
f1 (ppm)

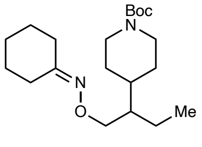

Boc
N
O
Me

3aj

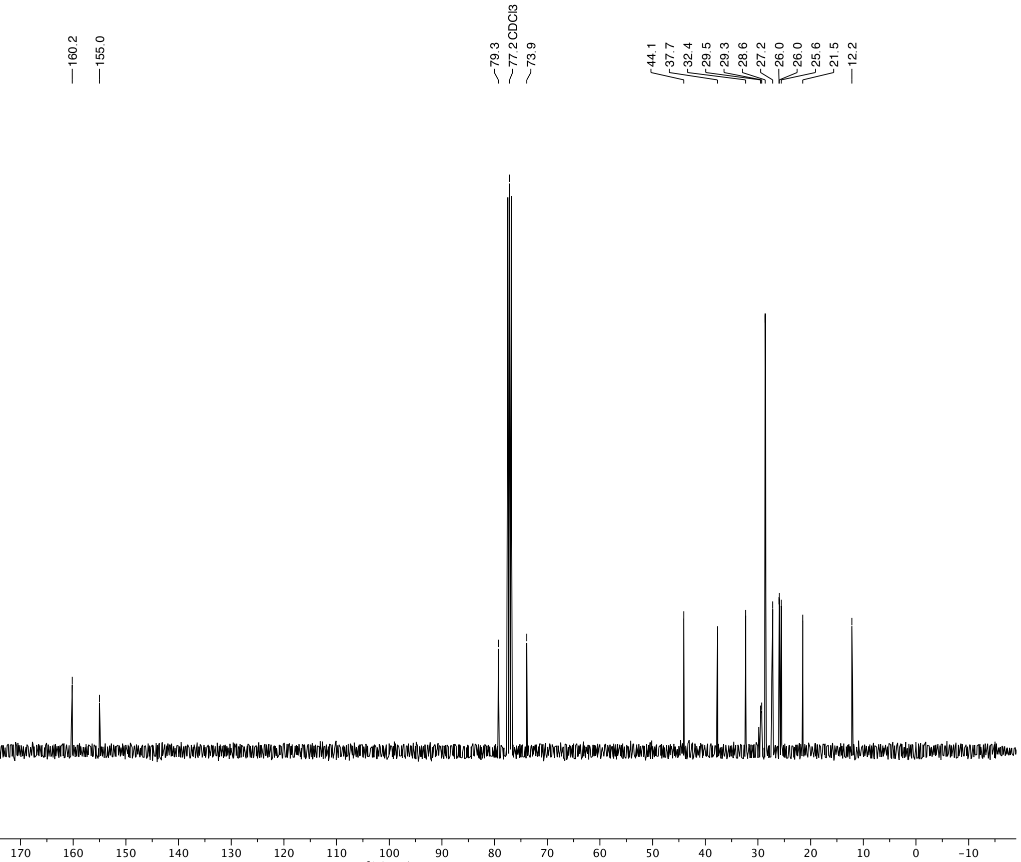

160.2
155.0
79.3
77.2 CDCl3
73.9
44.1
37.7
32.4
29.5
29.3
28.6
27.2
26.0
26.0
25.6
21.5
12.2
f1 (ppm)

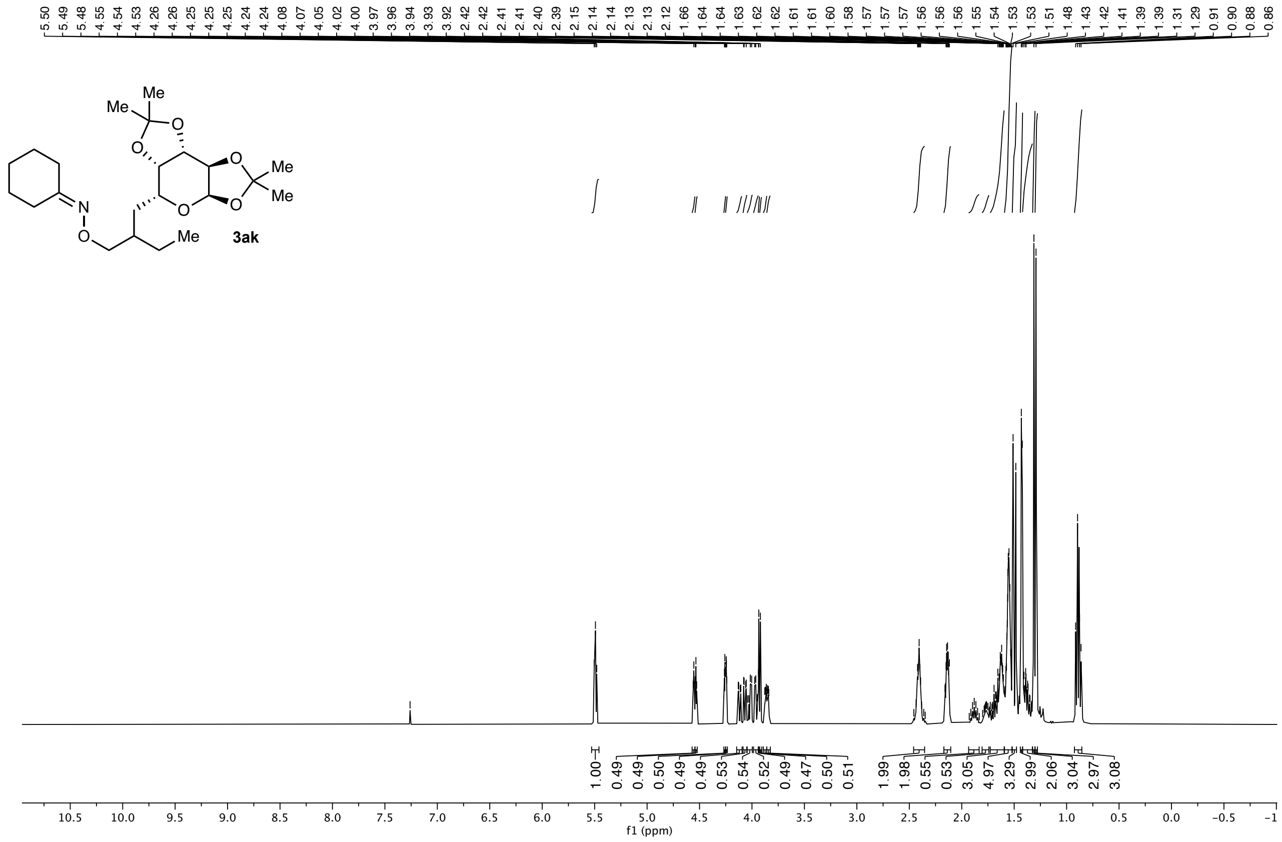
5.50 5.49 5.48 4.55 4.54 4.53 4.26 4.25 4.25 4.24 4.24 4.08 4.07 4.05 4.02 4.00 3.97 3.96 3.94 3.93 3.92 2.42 2.42 2.41 2.41 2.40 2.39 2.15 2.14 2.14 2.13 2.13 2.12 1.66 1.64 1.64 1.63 1.62 1.62 1.61 1.61 1.60 1.58 1.57 1.57 1.56 1.56 1.56 1.55 1.54 1.53 1.53 1.51 1.48 1.43 1.42 1.41 1.39 1.39 1.31 1.29 0.91 0.90 0.88 0.86
Me
Me
O
O
O
O
Me
Me
N
O
O
Me
3ak
1.00 0.49 0.49 0.50 0.49 0.49 0.53 0.54 0.52 0.49 0.47 0.50 0.51 1.99 1.98 0.55 0.53 3.05 4.97 3.29 2.99 2.06 3.04 2.97 3.08
10.5 10.0 9.5 9.0 8.5 8.0 7.5 7.0 6.5 6.0 5.5 5.0 4.5 4.0 3.5 3.0 2.5 2.0 1.5 1.0 0.5 0.0 −0.5
f1 (ppm)

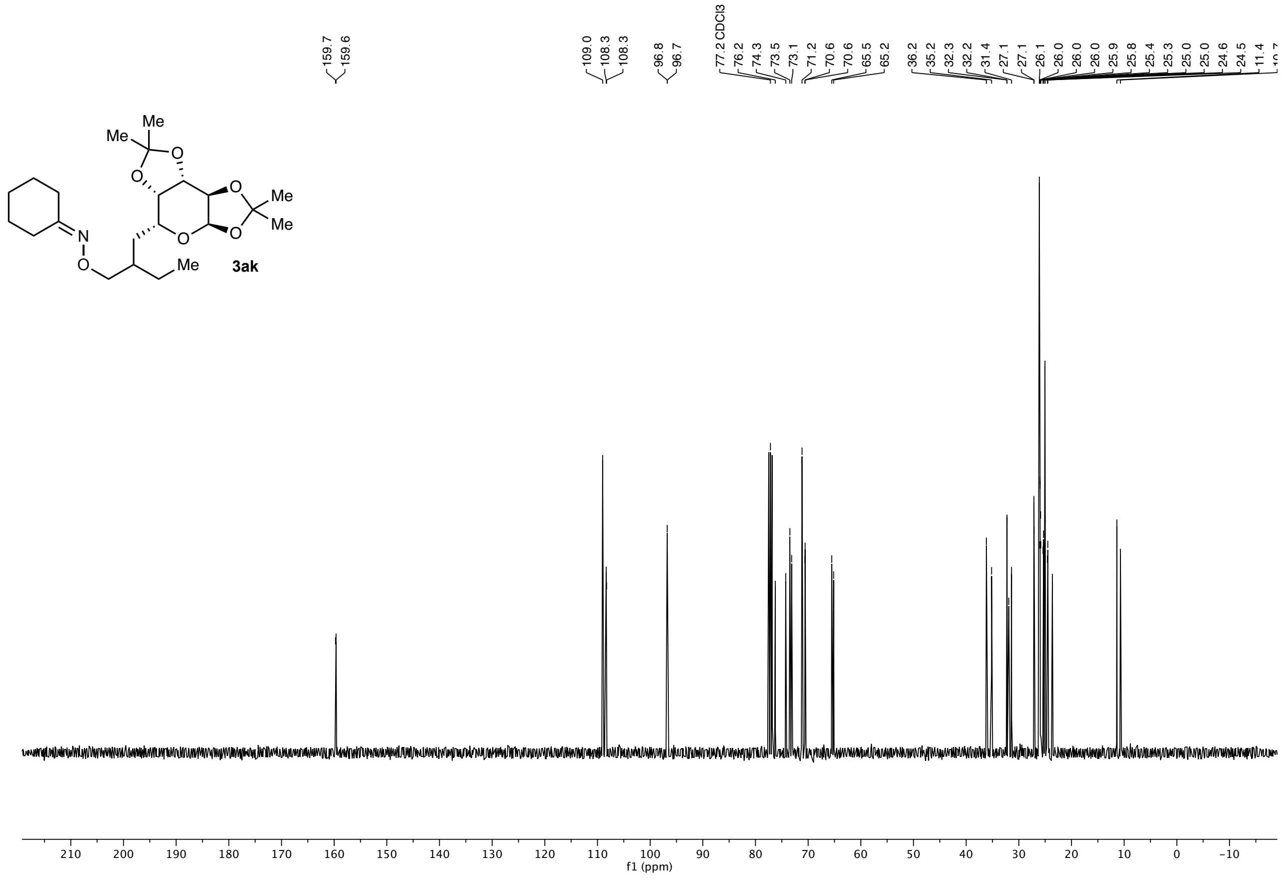
159.7 159.6 109.0 108.3 108.3 96.8 96.7 77.2 CDCl3 76.2 74.3 73.5 73.1 71.2 70.6 70.6 65.5 65.2 36.2 35.2 32.3 32.2 31.4 27.1 27.1 26.1 26.0 26.0 25.9 25.8 25.4 25.3 25.0 25.0 24.6 24.5 11.4
Me
Me
O
O
O
O
Me
Me
N
O
O
Me
3ak
210 200 190 180 170 160 150 140 130 120 110 100 90 80 70 60 50 40 30 20 10 0 −10
f1 (ppm)

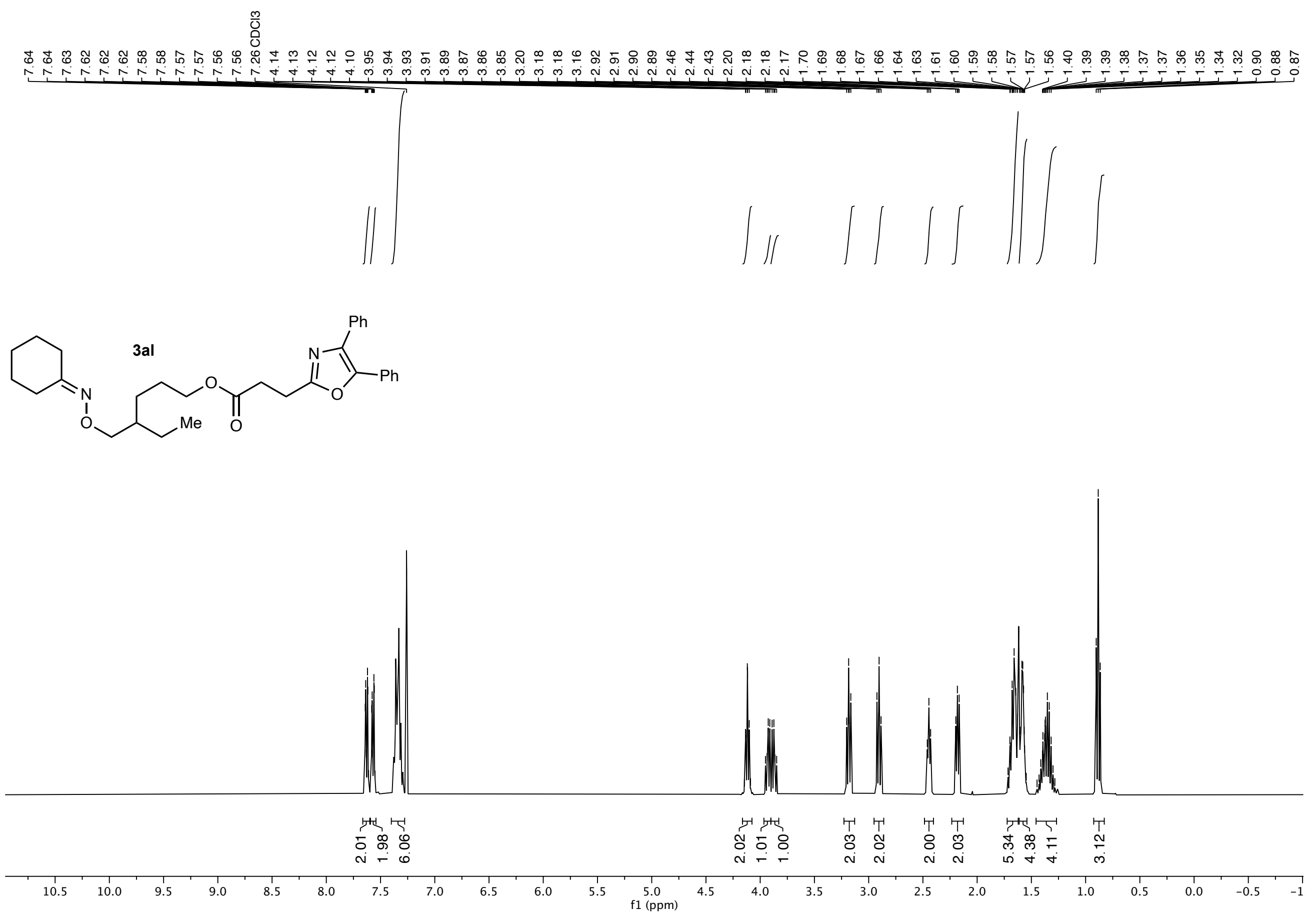
7.64 7.64 7.63 7.62 7.62 7.58 7.58 7.57 7.57 7.56 7.56 7.26 CDCl3 4.14 4.13 4.12 4.12 4.10 3.95 3.94 3.93 3.91 3.89 3.87 3.86 3.85 3.20 3.18 3.18 3.16 2.92 2.91 2.90 2.89 2.46 2.44 2.43 2.20 2.18 2.18 2.17 1.70 1.69 1.68 1.67 1.66 1.64 1.63 1.61 1.60 1.59 1.58 1.57 1.57 1.56 1.40 1.39 1.39 1.38 1.37 1.37 1.36 1.35 1.34 1.32 0.90 0.88 0.87
3al
Ph
N
Ph
O
O
N
O
Me
O
2.01 1.98 6.06 2.02 1.01 1.00 2.03 2.02 2.00 2.03 5.34 4.38 4.11 3.12
10.5 10.0 9.5 9.0 8.5 8.0 7.5 7.0 6.5 6.0 5.5 5.0 4.5 4.0 3.5 3.0 2.5 2.0 1.5 1.0 0.5 0.0 −0.5 −1
f1 (ppm)

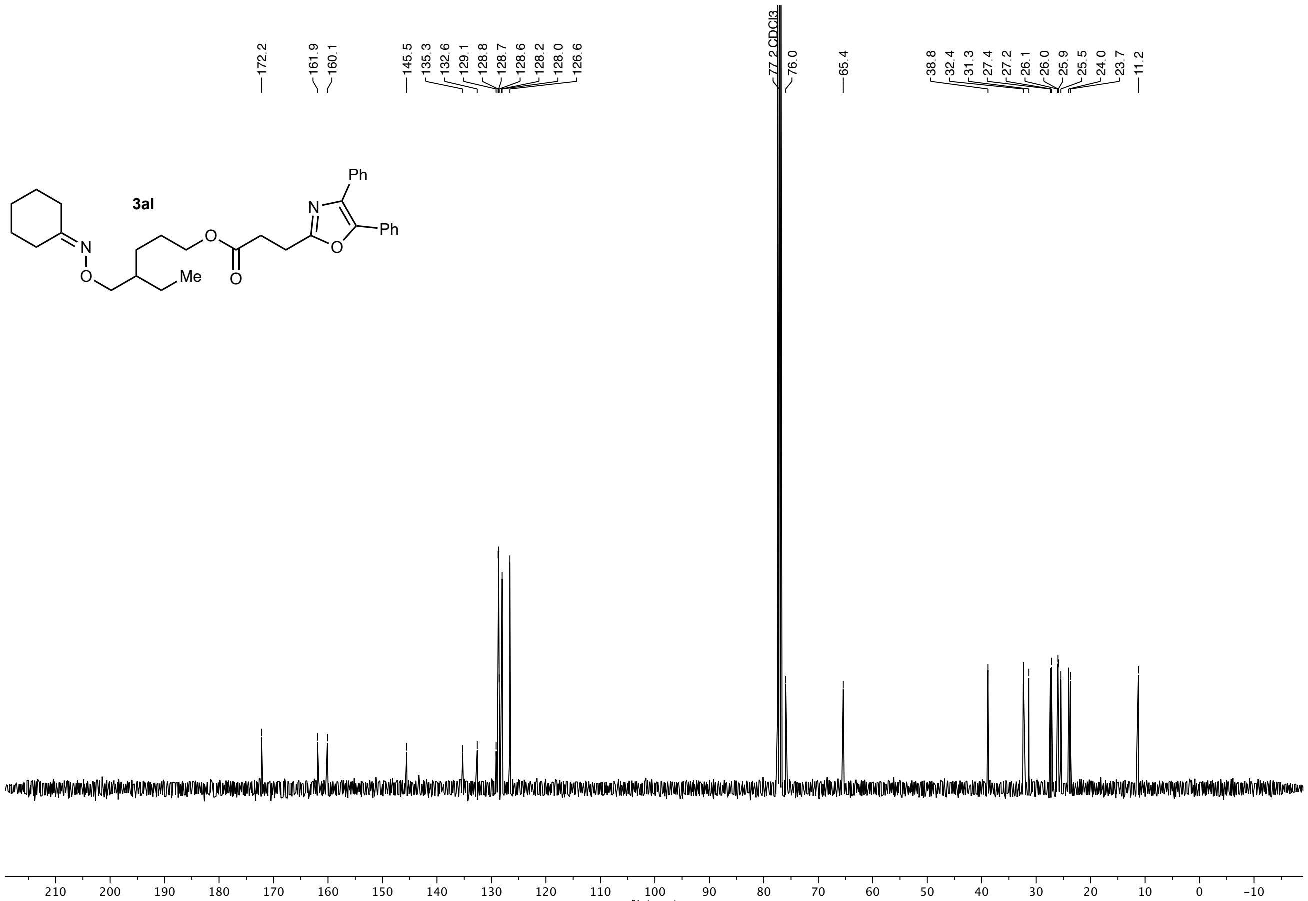
172.2 161.9 160.1 145.5 135.3 132.6 129.1 128.8 128.7 128.6 128.2 128.0 126.6 77.2 CDCl3 76.0 65.4 38.8 32.4 31.3 27.4 27.2 26.1 26.0 25.9 25.5 24.0 23.7 11.2
3al
Ph
N
Ph
O
O
N
O
Me
O
210 200 190 180 170 160 150 140 130 120 110 100 90 80 70 60 50 40 30 20 10 0 −10
f1 (ppm)

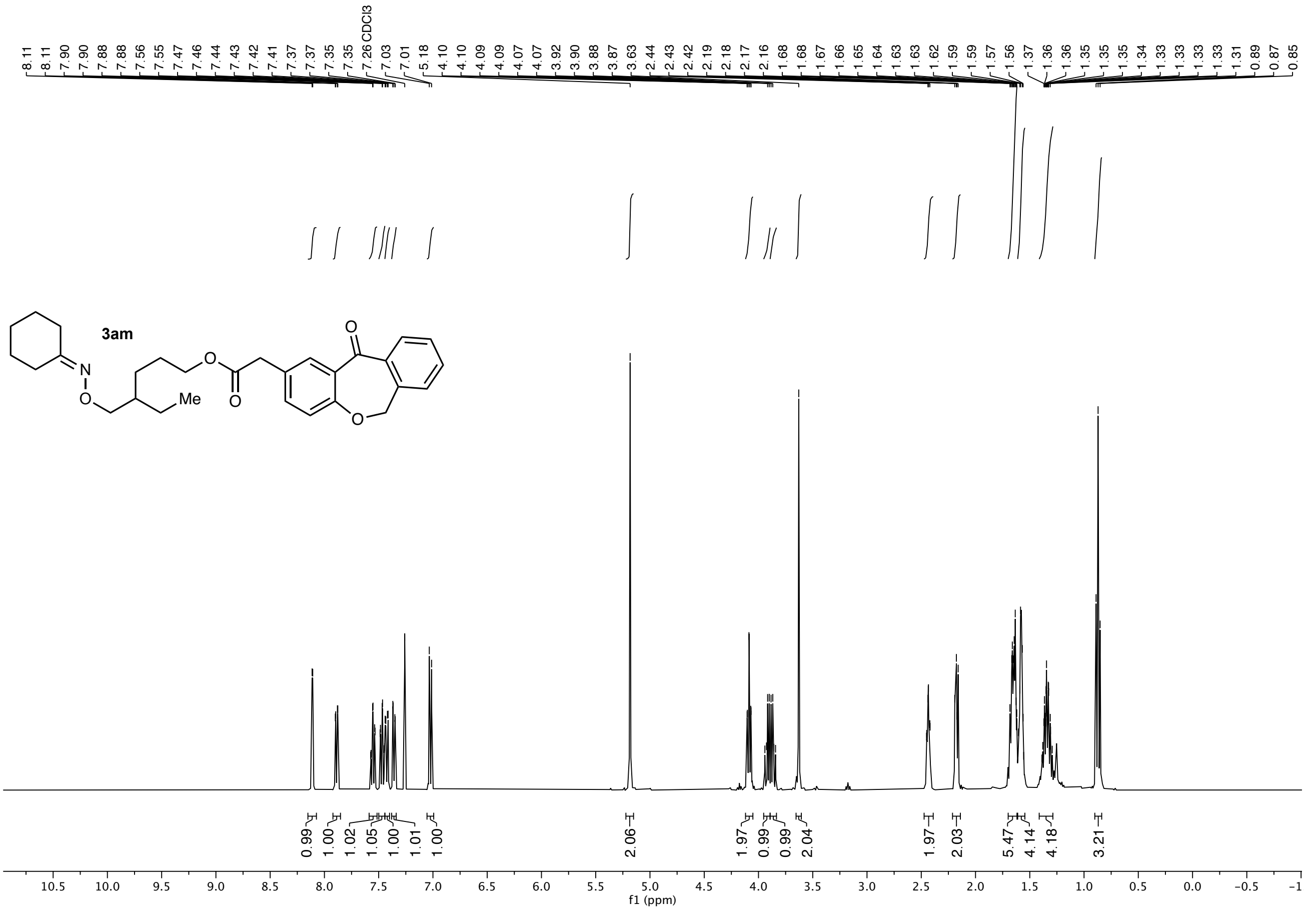
8.11 8.11 7.90 7.90 7.88 7.88 7.56 7.55 7.47 7.46 7.44 7.43 7.42 7.41 7.37 7.37 7.35 7.35 7.26 CDCl3 7.03 7.01 5.18 4.10 4.10 4.09 4.09 4.07 4.07 3.92 3.90 3.88 3.87 3.63 2.44 2.43 2.42 2.19 2.18 2.17 2.16 1.68 1.67 1.66 1.65 1.64 1.63 1.63 1.62 1.59 1.59 1.57 1.56 1.37 1.36 1.36 1.35 1.35 1.34 1.33 1.33 1.33 1.33 1.31 0.89 0.87 0.85
3am
N
O
Me
O
O
O
O
0.99 1.00 1.02 1.05 1.00 1.01 1.00 2.06 1.97 0.99 0.99 2.04 1.97 2.03 5.47 4.14 4.18 3.21
10.5 10.0 9.5 9.0 8.5 8.0 7.5 7.0 6.5 6.0 5.5 5.0 4.5 4.0 3.5 3.0 2.5 2.0 1.5 1.0 0.5 0.0 −0.5 −1
f1 (ppm)

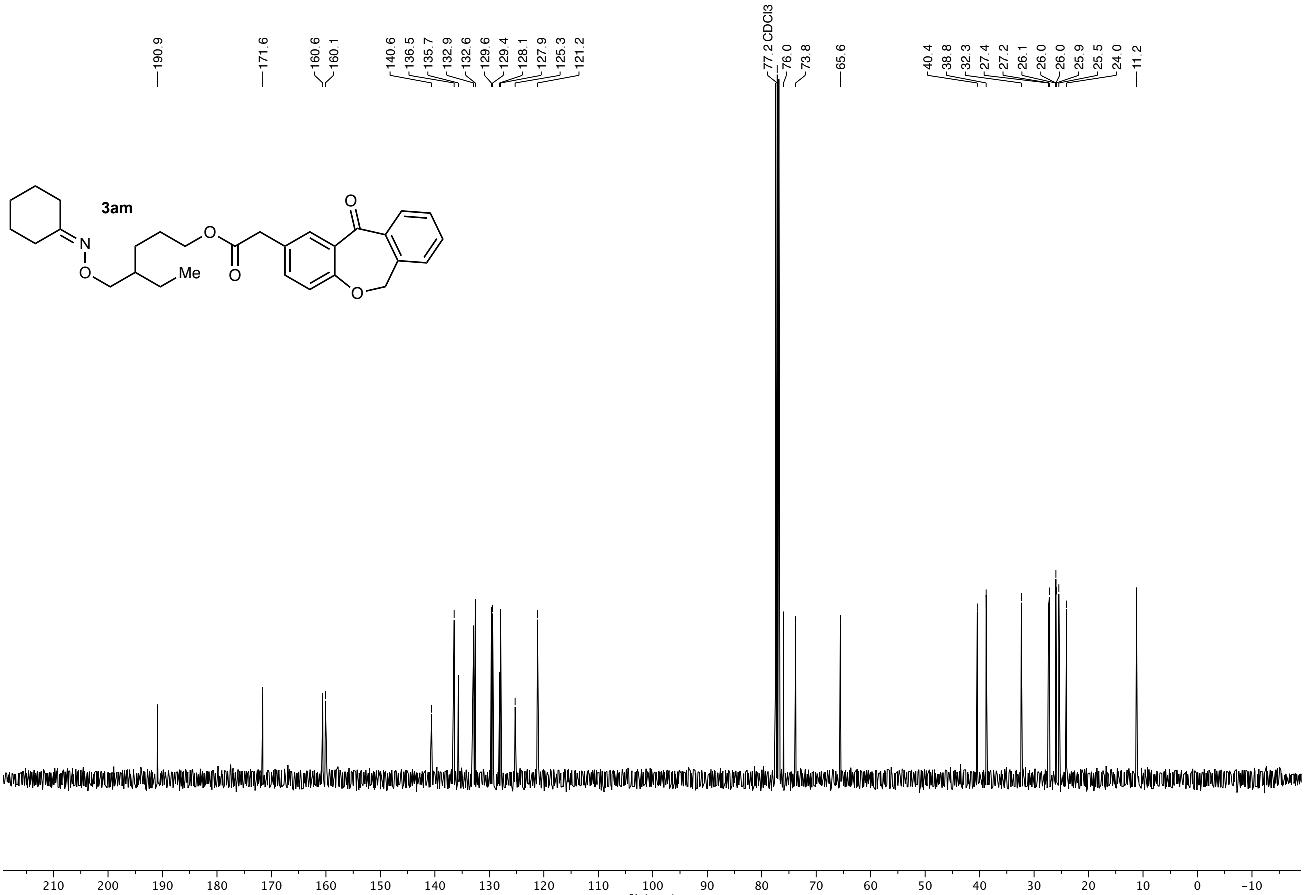
190.9 171.6 160.6 160.1 140.6 136.5 135.7 132.9 132.6 129.6 129.4 128.1 127.9 125.3 121.2 77.2 CDCl3 76.0 73.8 65.6 40.4 38.8 32.3 27.4 27.2 26.1 26.0 26.0 25.9 25.5 24.0 11.2
3am
N
O
Me
O
O
O
O
210 200 190 180 170 160 150 140 130 120 110 100 90 80 70 60 50 40 30 20 10 0 −10
f1 (ppm)

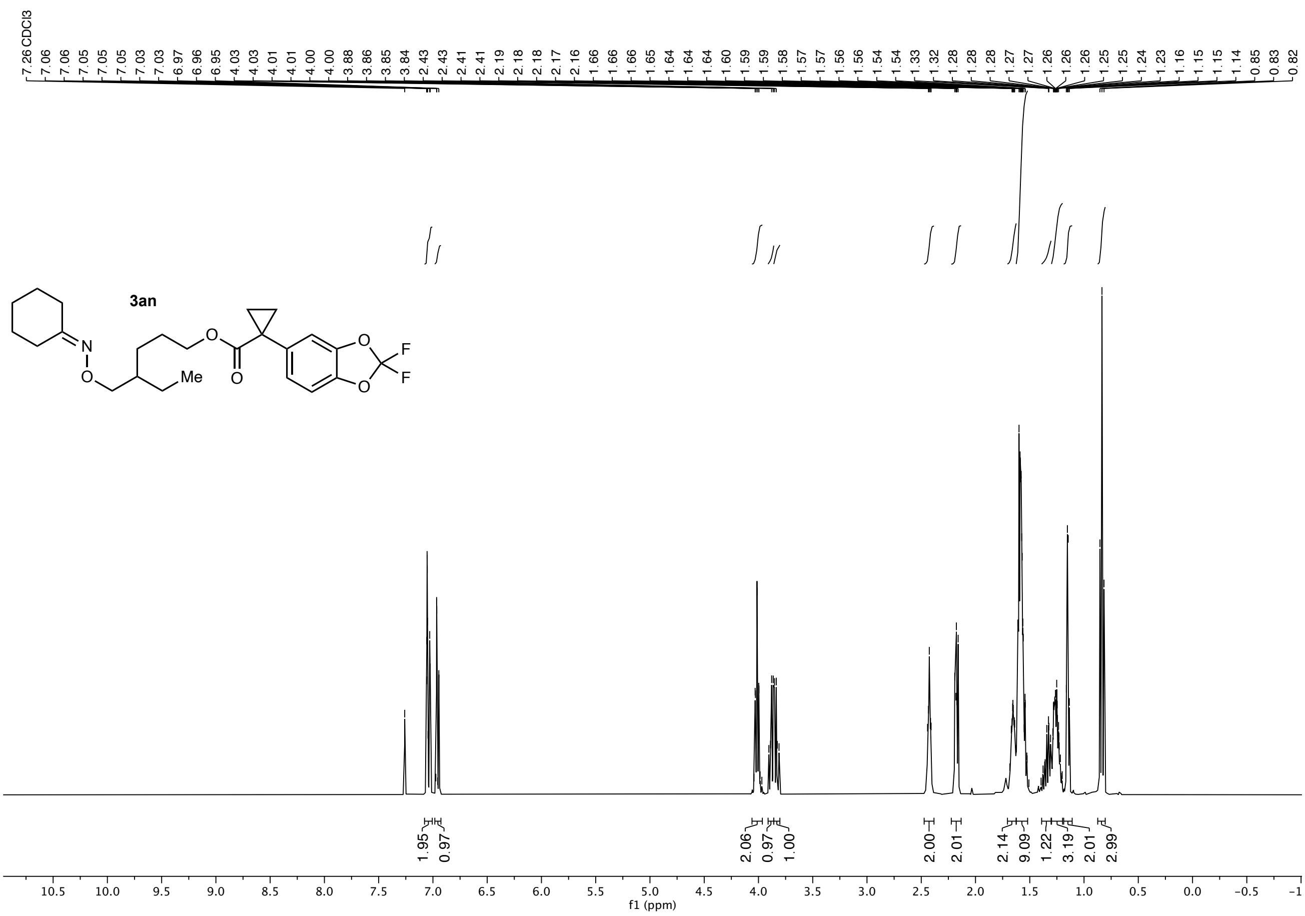
7.26 CDCl3
7.06 7.06 7.05 7.05 7.05 7.03 7.03 6.97 6.96 6.95 4.03 4.01 4.01 4.00 4.00 3.88 3.86 3.85 3.84 2.43 2.43 2.41 2.19 2.18 2.18 2.17 2.16 1.66 1.66 1.65 1.64 1.64 1.60 1.59 1.59 1.58 1.57 1.57 1.56 1.56 1.54 1.54 1.33 1.32 1.28 1.28 1.28 1.27 1.27 1.26 1.26 1.26 1.25 1.25 1.24 1.23 1.16 1.15 1.14 0.85 0.83 0.82
3an
N
O
Me
O
O
O
F
F
O
1.95 0.97 2.06 0.97 1.00 2.00 2.01 2.14 9.09 1.22 3.19 2.01 2.99
10.5 10.0 9.5 9.0 8.5 8.0 7.5 7.0 6.5 6.0 5.5 5.0 4.5 4.0 3.5 3.0 2.5 2.0 1.5 1.0 0.5 0.0 -0.5 -1
f1 (ppm)

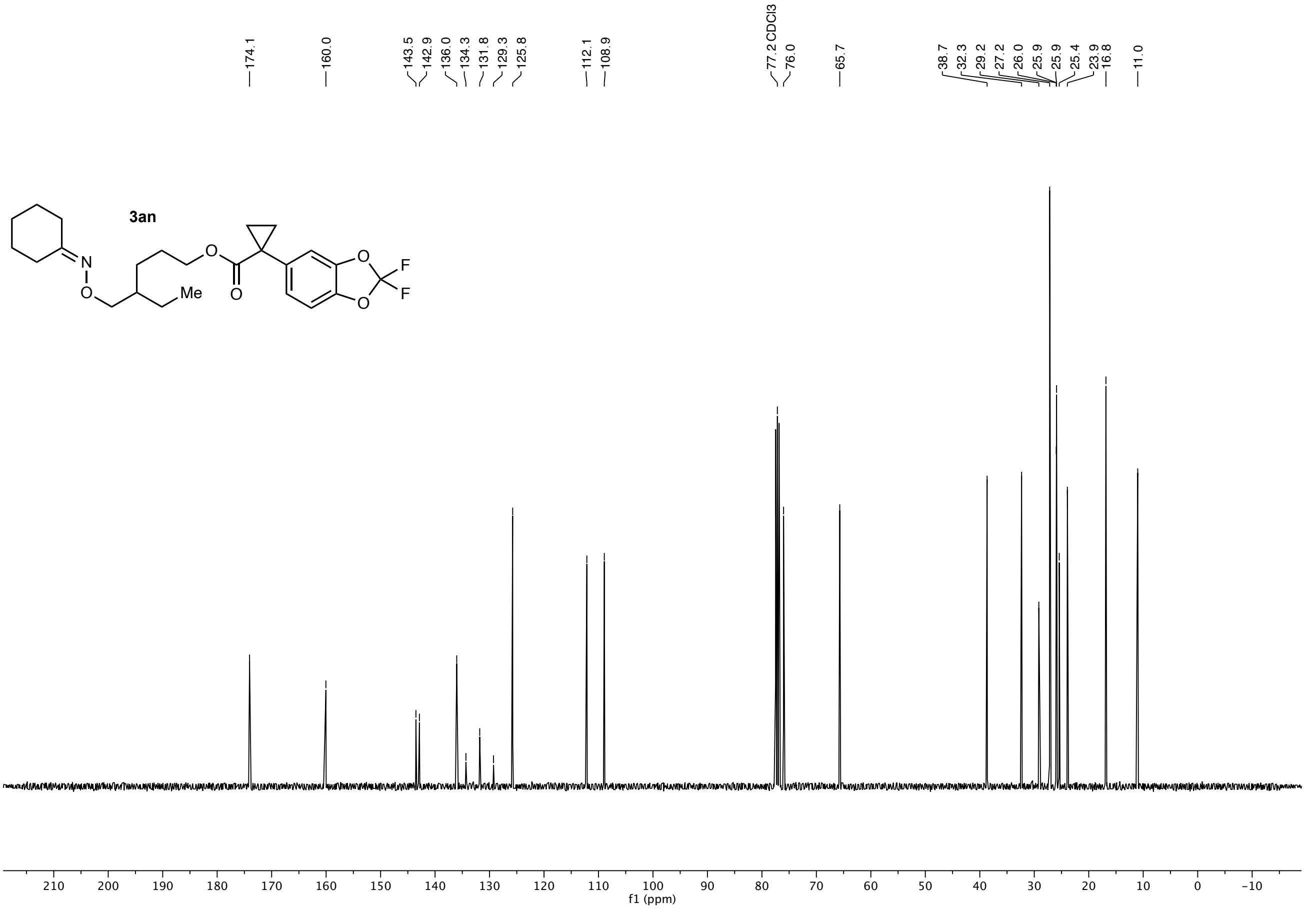
174.1 160.0 143.5 142.9 136.0 134.3 131.8 129.3 125.8 112.1 108.9 77.2 CDCl3 76.0 65.7 38.7 32.3 29.2 27.2 26.0 25.9 25.9 25.4 23.9 16.8 11.0
3an
N
O
Me
O
O
O
F
F
O
210 200 190 180 170 160 150 140 130 120 110 100 90 80 70 60 50 40 30 20 10 0 -10
f1 (ppm)

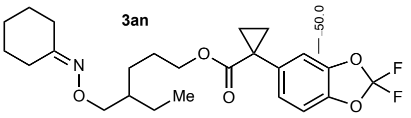
N
O
Me
O
O
O
O
F
F

3an

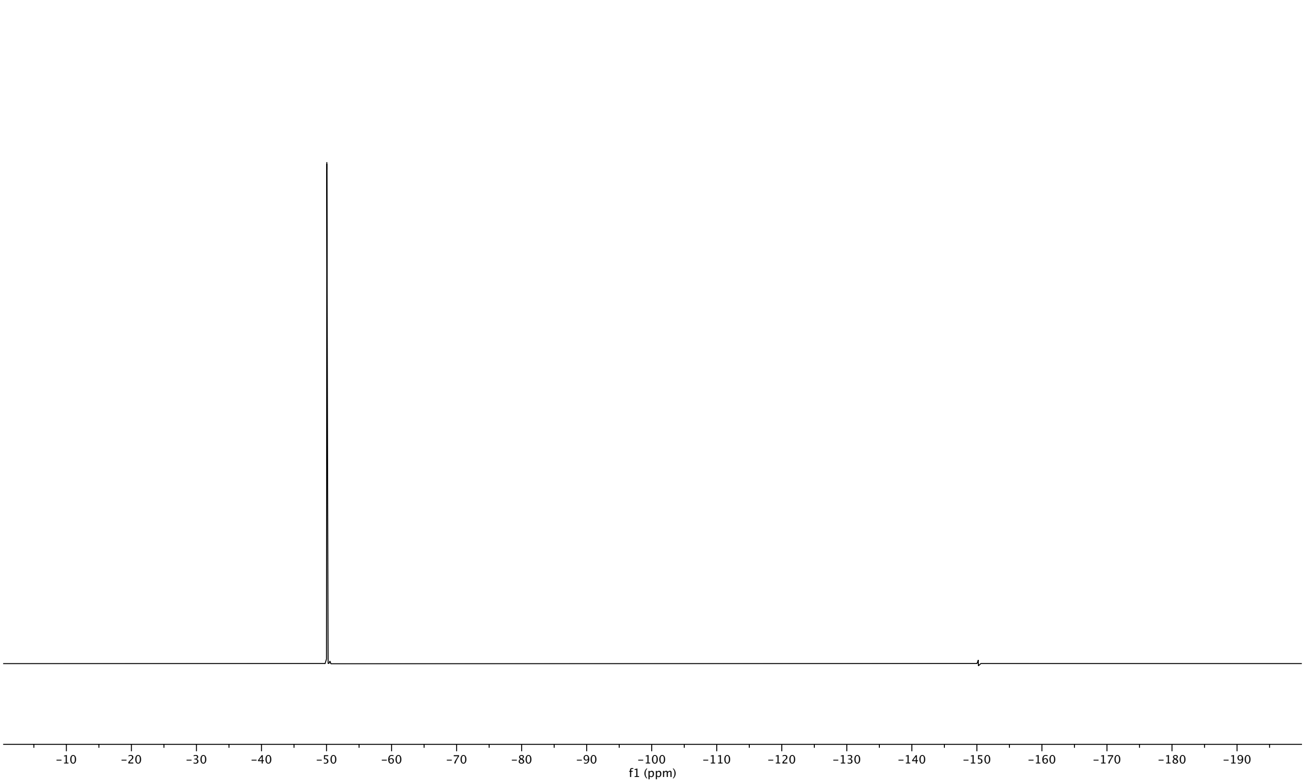
-50.0
-10
-20
-30
-40
-50
-60
-70
-80
-90
-100
-110
-120
-130
-140
-150
-160
-170
-180
-190
f1 (ppm)

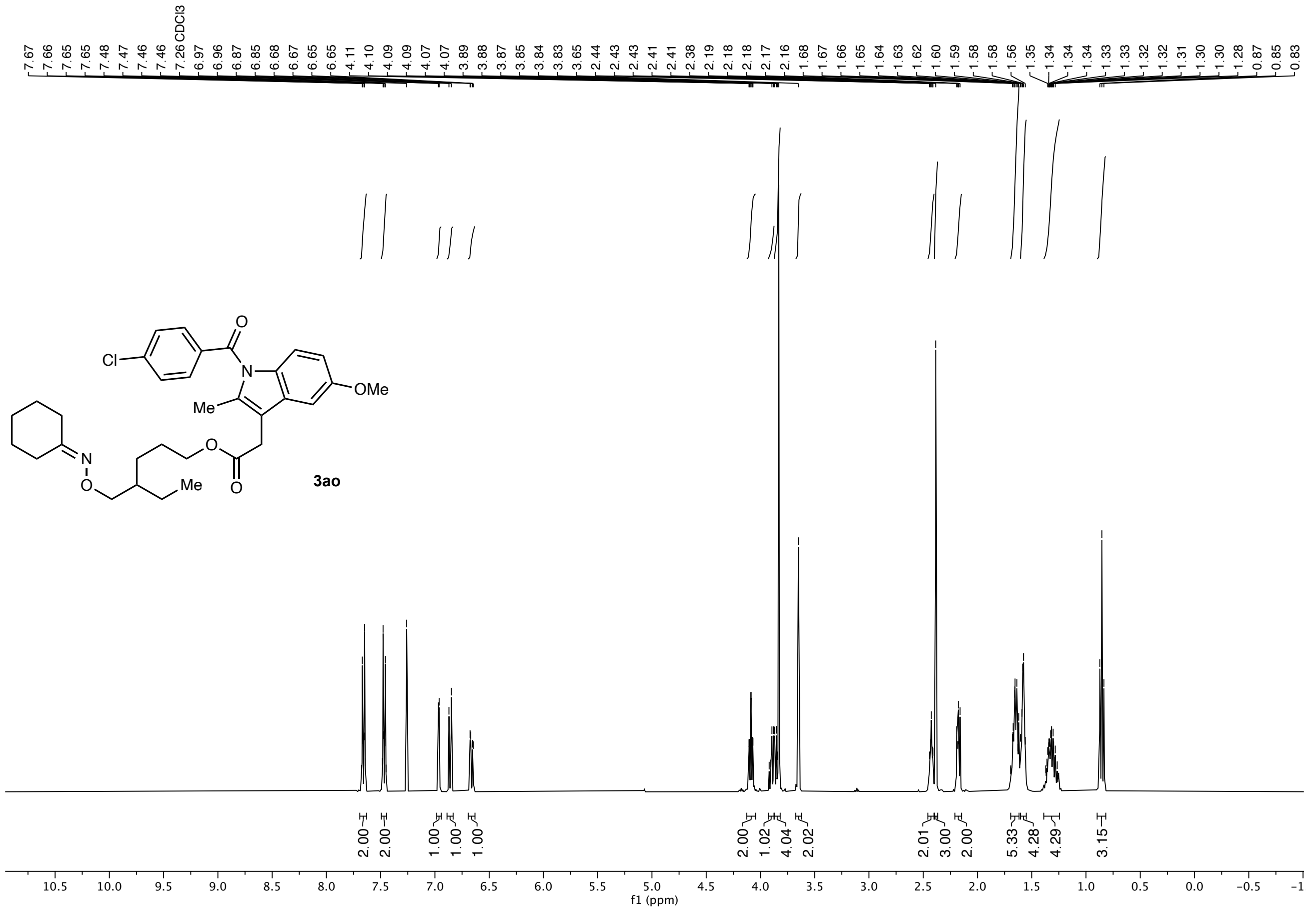
7.67 7.66 7.65 7.65 7.48 7.47 7.46 7.46 7.26 CDCl3 6.97 6.96 6.87 6.85 6.68 6.67 6.65 6.65 4.11 4.10 4.09 4.09 4.07 4.07 3.89 3.88 3.87 3.85 3.84 3.83 3.65 2.44 2.43 2.43 2.41 2.41 2.38 2.19 2.18 2.18 2.17 2.16 1.68 1.67 1.66 1.65 1.64 1.63 1.62 1.60 1.59 1.58 1.58 1.56 1.35 1.34 1.34 1.34 1.33 1.33 1.32 1.32 1.31 1.30 1.28 0.87 0.85 0.83
Cl
O
N
OMe
Me
N
O
O
O
Me
3ao
2.00 2.00 1.00 1.00 1.00 2.00 1.02 4.04 2.02 2.01 3.00 2.00 5.33 4.28 4.29 3.15
f1 (ppm)

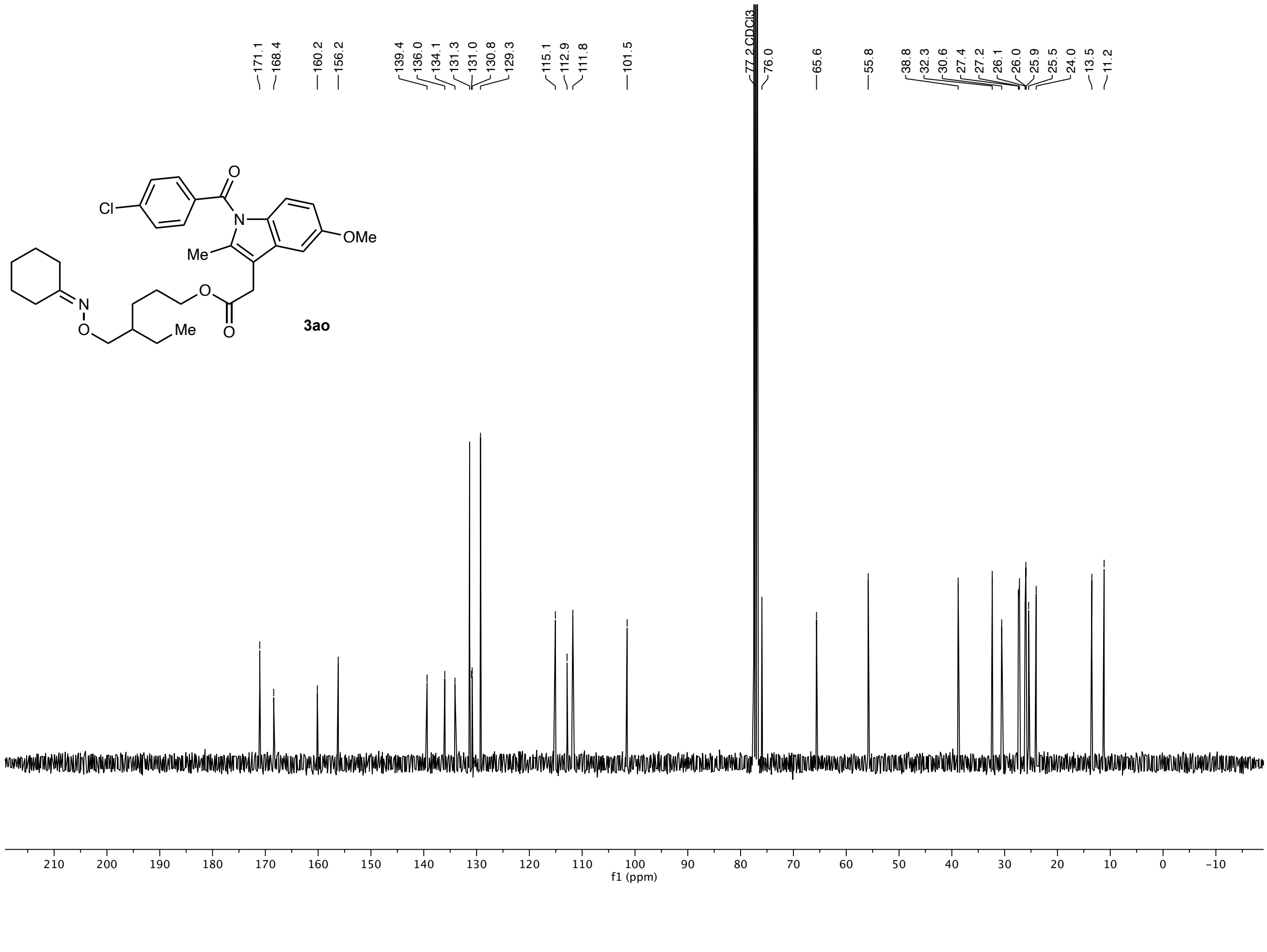
171.1 168.4 160.2 156.2 139.4 136.0 134.1 131.3 131.0 130.8 129.3 115.1 112.9 111.8 101.5 77.2 CDCl3 76.0 65.6 55.8 38.8 32.3 30.6 27.4 27.2 26.1 26.0 25.9 25.5 24.0 13.5 11.2
Cl
O
N
OMe
Me
N
O
O
O
Me
3ao
f1 (ppm)

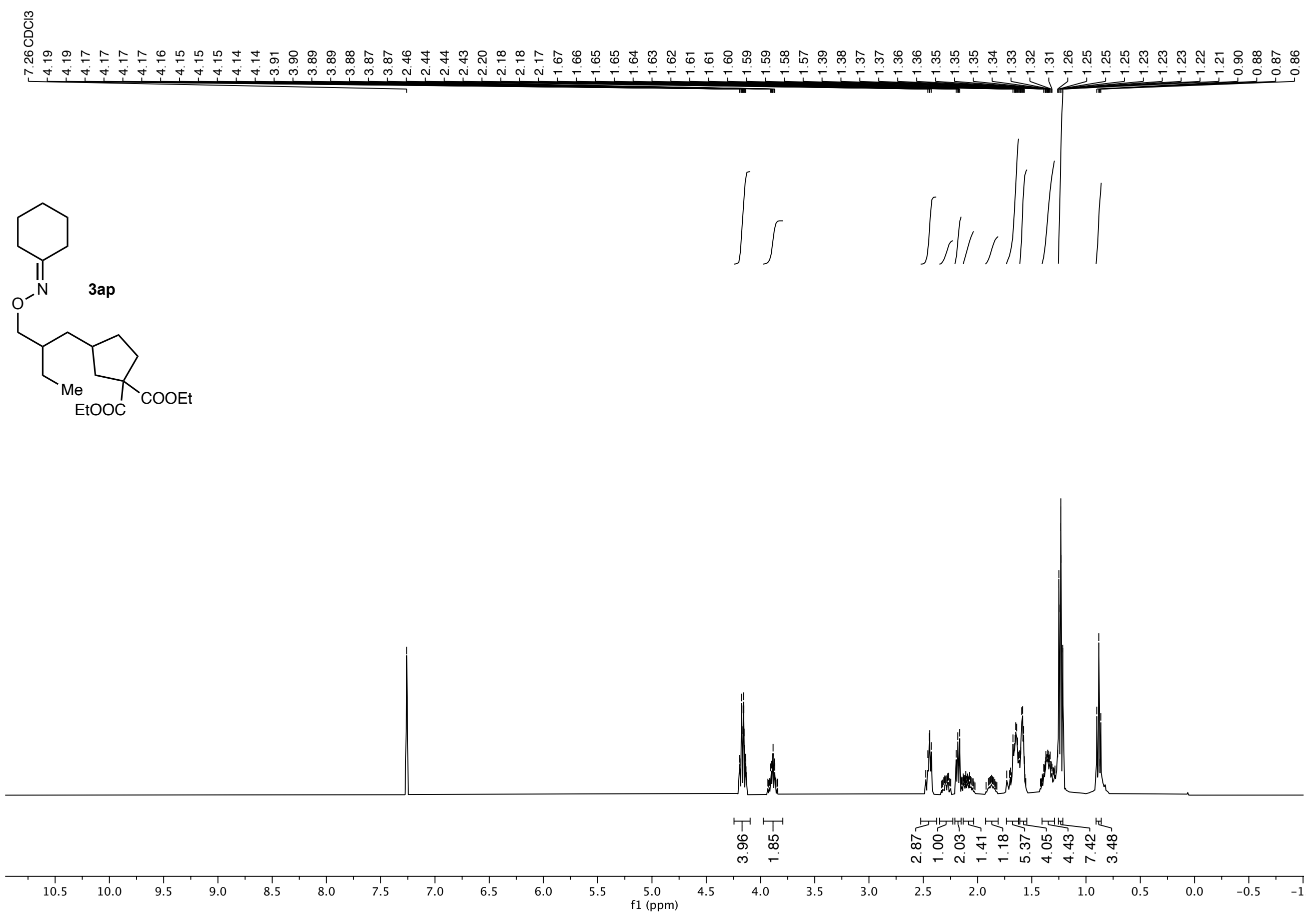
3ap
N
O
Me
COOEt
EtOOC
7.26 CDCl3
4.19 4.17 4.17 4.17 4.16 4.15 4.15 4.14 4.14 3.91 3.90 3.89 3.88 3.87 3.87 2.46 2.44 2.44 2.43 2.20 2.18 2.18 2.17 1.67 1.66 1.65 1.65 1.64 1.63 1.62 1.61 1.61 1.60 1.59 1.59 1.58 1.57 1.39 1.38 1.37 1.37 1.36 1.36 1.35 1.35 1.35 1.34 1.33 1.32 1.31 1.26 1.25 1.25 1.25 1.23 1.23 1.23 1.22 1.21 0.90 0.88 0.87 0.86
3.96 1.85 2.87 1.00 2.03 1.41 1.18 5.37 4.05 4.43 7.42 3.48
f1 (ppm)

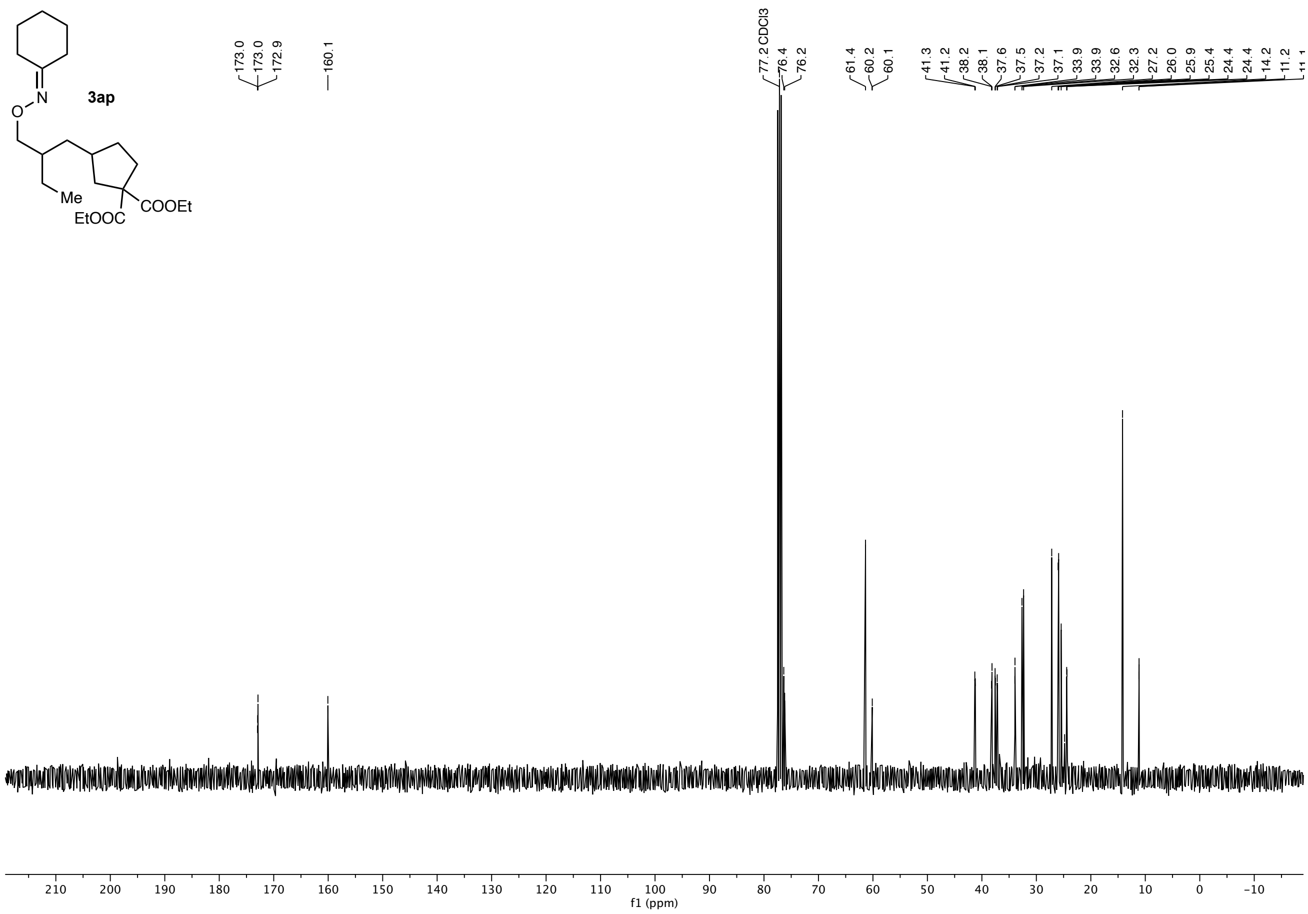
3ap
N
O
Me
COOEt
EtOOC
173.0 173.0 172.9 160.1
77.2 CDCl3 76.4 76.2
61.4 60.2 60.1
41.3 41.2 38.2 38.1 37.6 37.5 37.2 37.1 33.9 33.9 32.6 32.3 27.2 26.0 25.9 25.4 24.4 24.4 14.2 11.2
f1 (ppm)

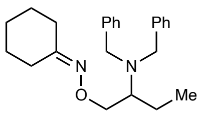
Ph
Ph
N
N
O
Me

5a

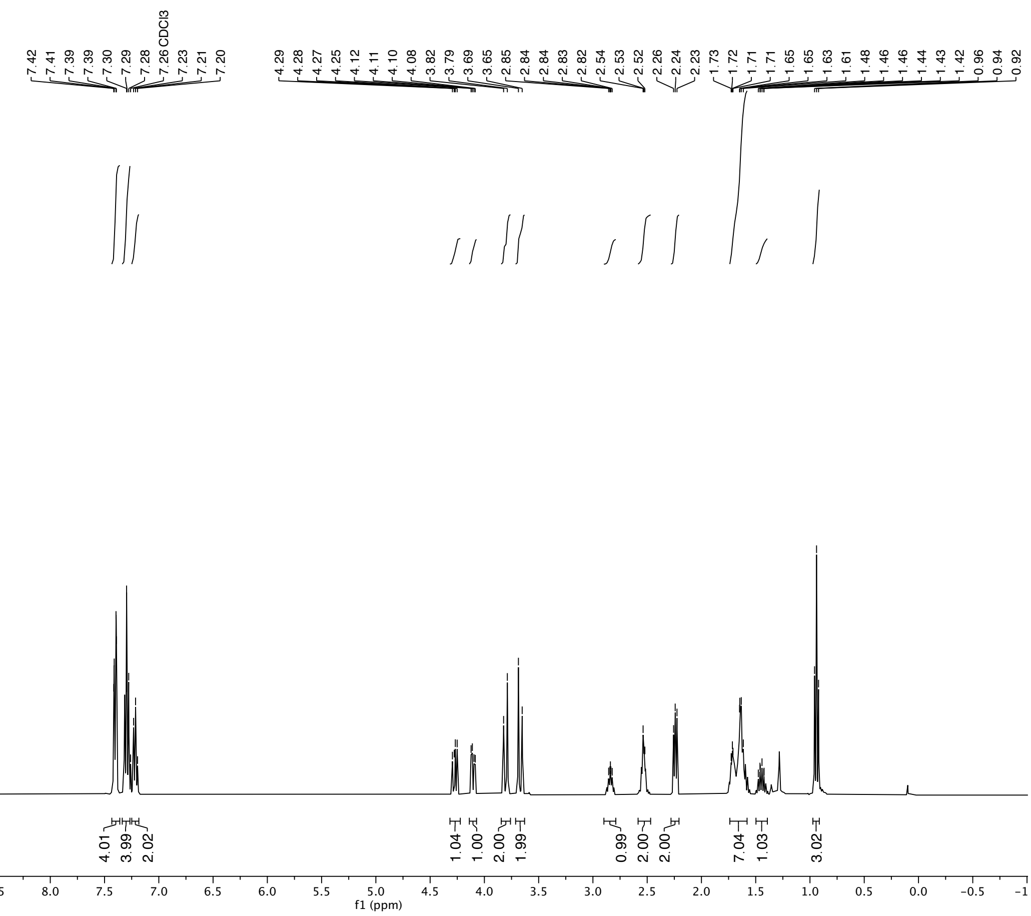
7.42 7.41 7.39 7.39 7.30 7.29 7.28 7.26 CDCl3 7.23 7.21 7.20
4.29 4.28 4.27 4.25 4.12 4.11 4.10 4.08 3.82 3.79 3.69 3.65 2.85 2.84 2.84 2.83 2.83 2.82 2.54 2.53 2.52 2.26 2.24 2.23 1.73 1.72 1.71 1.71 1.65 1.65 1.63 1.61 1.48 1.46 1.46 1.44 1.43 1.42 0.96 0.94 0.92
4.01 3.99 2.02 1.04 1.00 2.00 1.99 0.99 2.00 2.00 7.04 1.03 3.02
10.5 10.0 9.5 9.0 8.5 8.0 7.5 7.0 6.5 6.0 5.5 5.0 4.5 4.0 3.5 3.0 2.5 2.0 1.5 1.0 0.5 0.0 −0.5 −1
f1 (ppm)

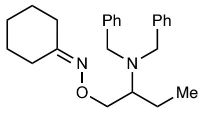
Ph
Ph
N
N
O
Me

5a

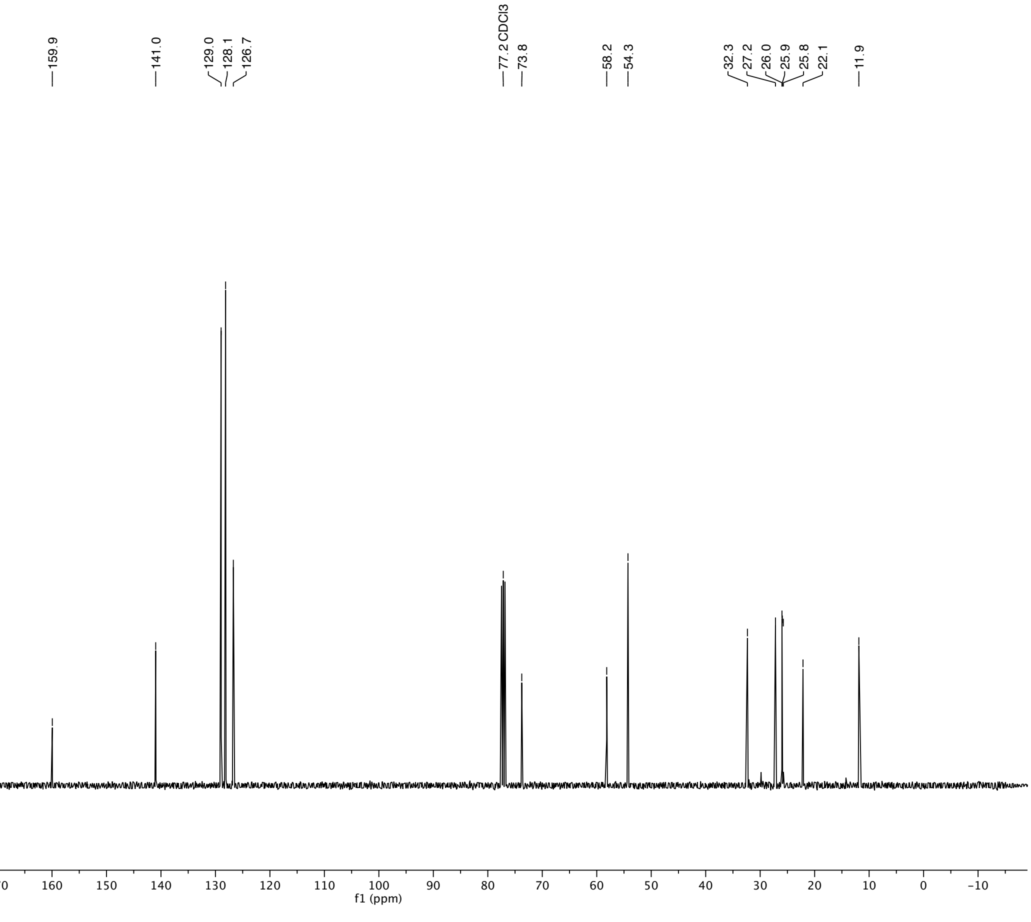
159.9
141.0
129.0 128.1 126.7
77.2 CDCl3
73.8
58.2
54.3
32.3 27.2 26.0 25.9 25.8 22.1
11.9
210 200 190 180 170 160 150 140 130 120 110 100 90 80 70 60 50 40 30 20 10 0 −10
f1 (ppm)

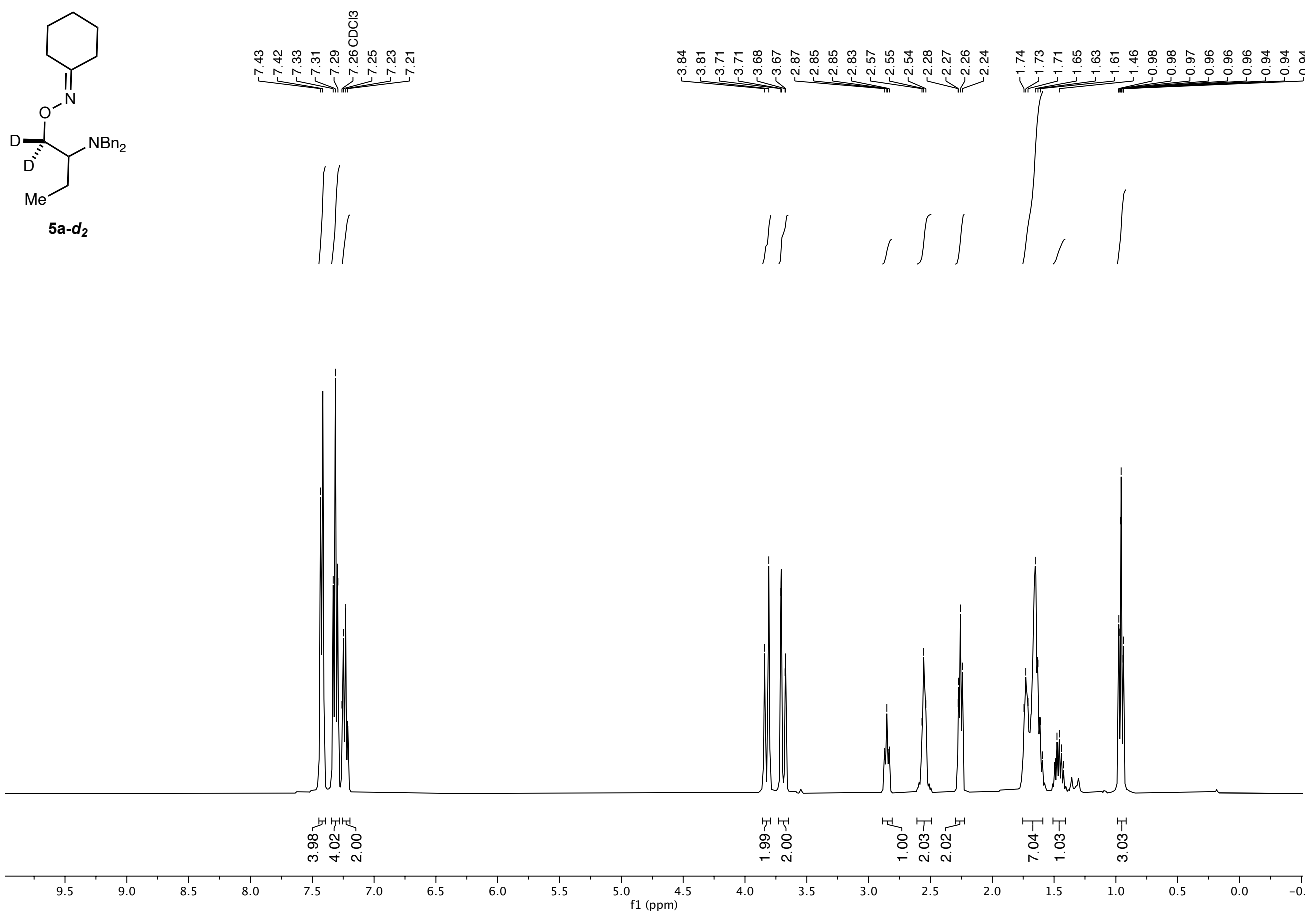

5a-d2
NBn2
Me
7.43 7.42 7.33 7.31 7.29 7.26 CDCl3 7.25 7.23 7.21
3.84 3.81 3.71 3.71 3.68 3.67 2.87 2.85 2.85 2.83 2.57 2.55 2.54 2.28 2.27 2.26 2.24
1.74 1.73 1.71 1.65 1.63 1.61 1.46 0.98 0.98 0.97 0.96 0.96 0.96 0.94 0.94
3.98 4.02 2.00 1.99 2.00 1.00 2.03 2.02 7.04 1.03 3.03
9.5 9.0 8.5 8.0 7.5 7.0 6.5 6.0 5.5 5.0 4.5 4.0 3.5 3.0 2.5 2.0 1.5 1.0 0.5 0.0
f1 (ppm)

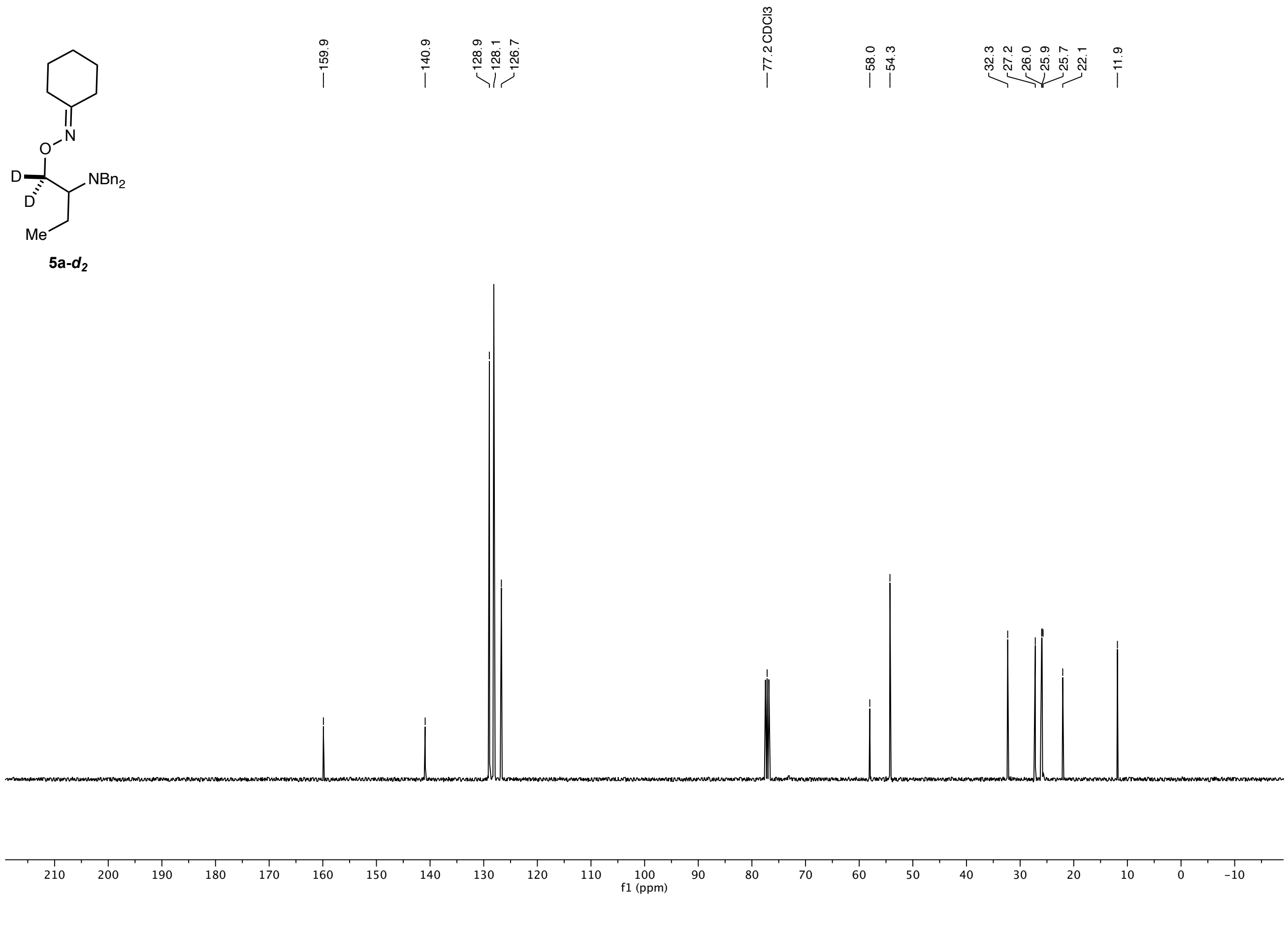

5a-d2
NBn2
Me
159.9
140.9
128.9 128.1 126.7
77.2 CDCl3
58.0 54.3
32.3 27.2 26.0 25.9 25.7 22.1
11.9
210 200 190 180 170 160 150 140 130 120 110 100 90 80 70 60 50 40 30 20 10 0 −10
f1 (ppm)

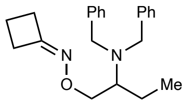
Ph
Ph
N
N
O
Me

5b

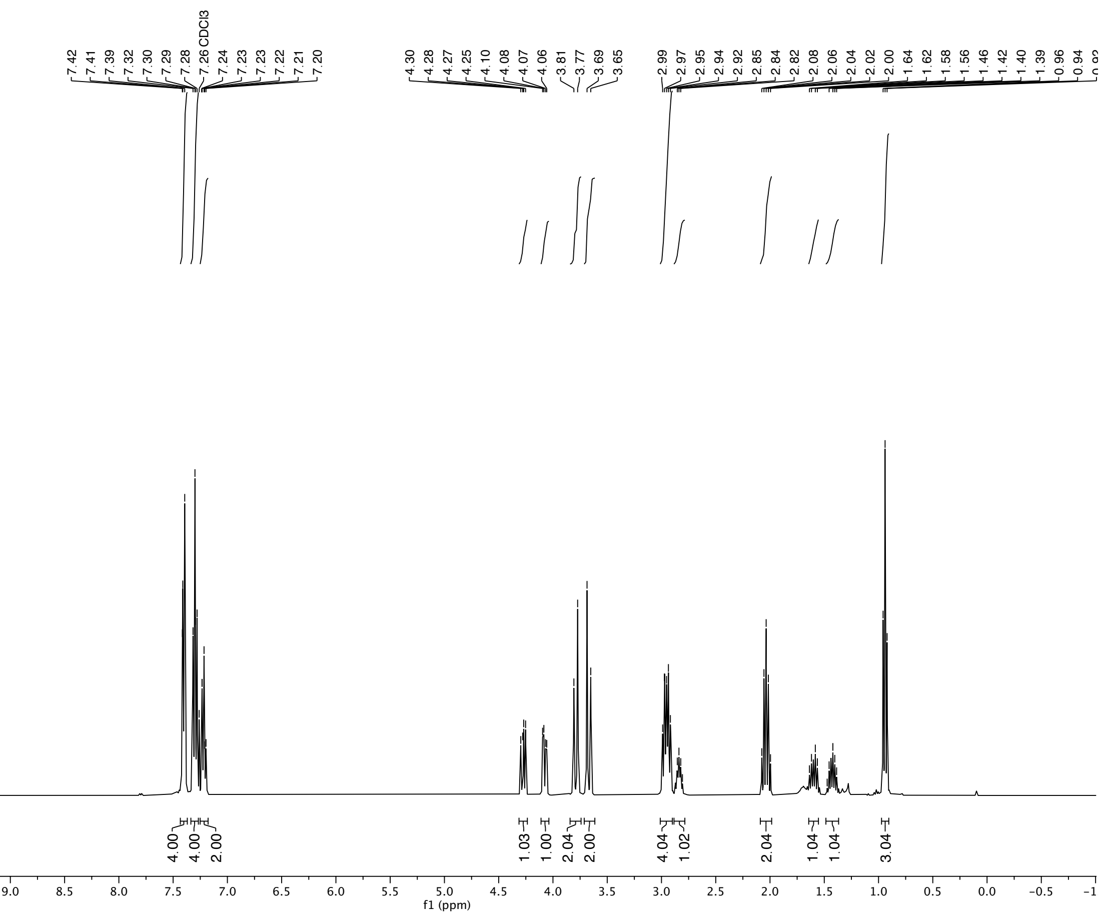
7.42
7.41
7.39
7.32
7.30
7.29
7.28
7.26 CDCl3
7.24
7.23
7.23
7.22
7.21
7.20
4.30
4.28
4.27
4.25
4.10
4.08
4.07
4.06
3.81
3.77
3.69
3.65
2.99
2.97
2.95
2.94
2.92
2.85
2.84
2.82
2.08
2.06
2.04
2.02
2.00
1.64
1.62
1.58
1.56
1.46
1.42
1.40
1.39
0.96
0.94
4.00
4.00
2.00
1.03
1.00
2.04
2.00
4.04
1.02
2.04
1.04
1.04
3.04
10.5
10.0
9.5
9.0
8.5
8.0
7.5
7.0
6.5
6.0
5.5
5.0
4.5
4.0
3.5
3.0
2.5
2.0
1.5
1.0
0.5
0.0
−0.5
f1 (ppm)

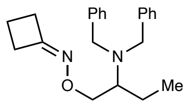
Ph
Ph
N
N
O
Me

5b

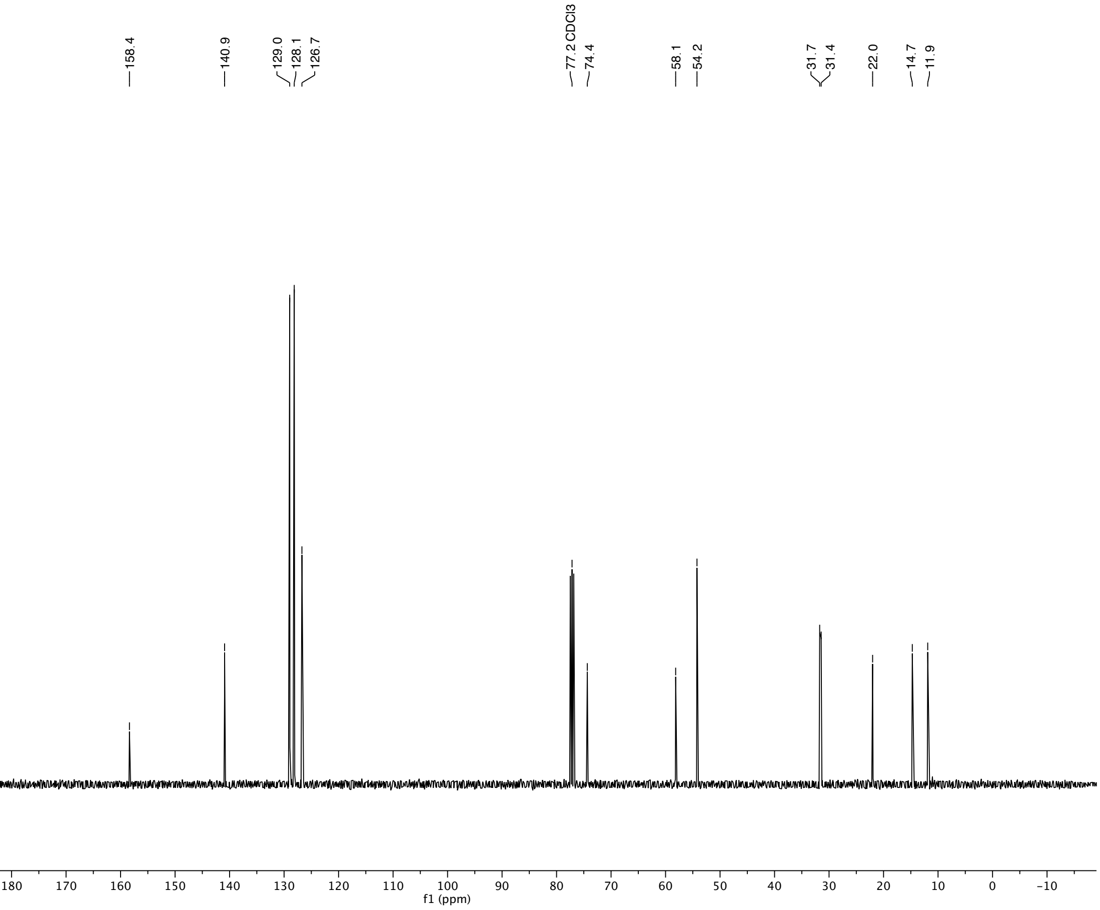
158.4
140.9
129.0
128.1
126.7
77.2 CDCl3
74.4
58.1
54.2
31.7
31.4
22.0
14.7
11.9
210
200
190
180
170
160
150
140
130
120
110
100
90
80
70
60
50
40
30
20
10
0
−10
f1 (ppm)

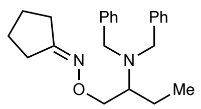
Ph
Ph
N
N
O
Me

5c

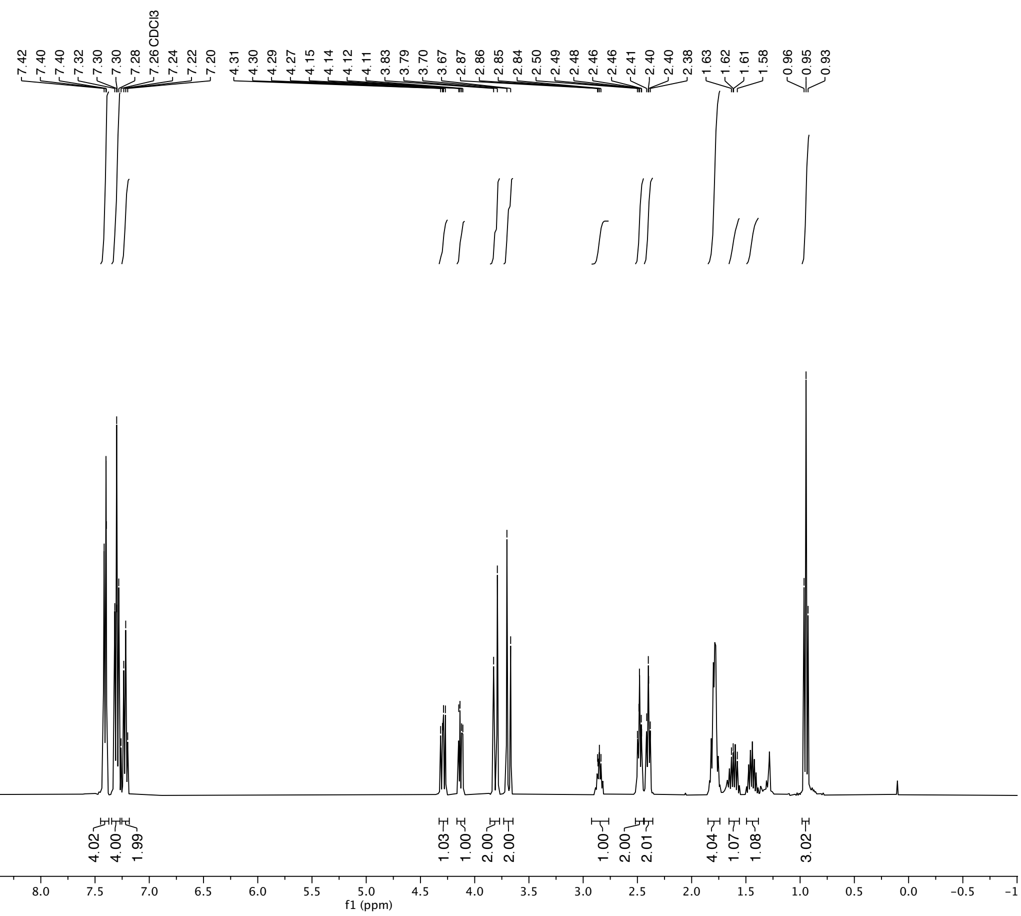
7.42 7.40 7.40 7.32 7.30 7.30 7.28 7.26 CDCl3 7.24 7.22 7.20 4.31 4.30 4.29 4.27 4.15 4.14 4.12 4.11 3.83 3.79 3.70 3.67 2.87 2.86 2.85 2.84 2.50 2.49 2.48 2.46 2.46 2.41 2.40 2.40 2.38 1.63 1.62 1.61 1.58 0.96 0.95 0.93
4.02 4.00 1.99 1.03 1.00 2.00 2.00 1.00 2.00 2.01 4.04 1.07 1.08 3.02
10.5 10.0 9.5 9.0 8.5 8.0 7.5 7.0 6.5 6.0 5.5 5.0 4.5 4.0 3.5 3.0 2.5 2.0 1.5 1.0 0.5 0.0 −0.5 −1
f1 (ppm)

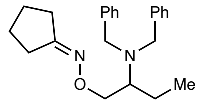
Ph
Ph
N
N
O
Me

5c

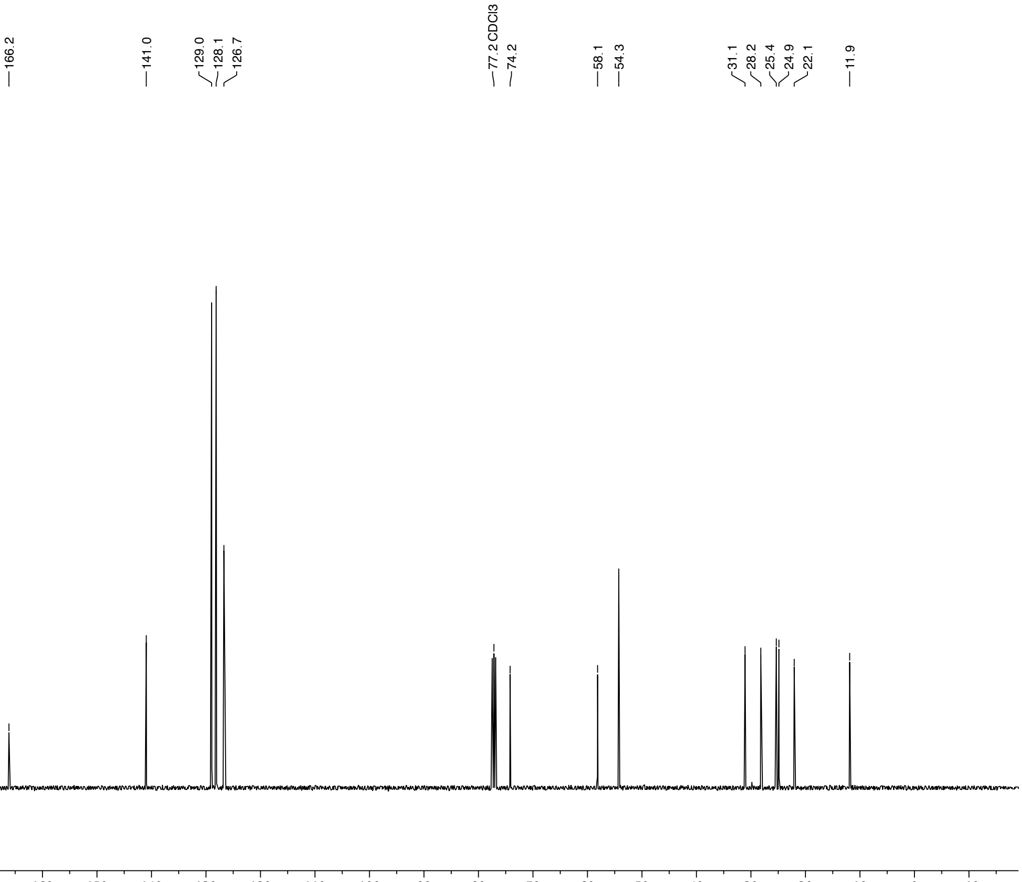
166.2 141.0 129.0 128.1 126.7 77.2 CDCl3 74.2 58.1 54.3 31.1 28.2 25.4 24.9 22.1 11.9
210 200 190 180 170 160 150 140 130 120 110 100 90 80 70 60 50 40 30 20 10 0 −10
f1 (ppm)

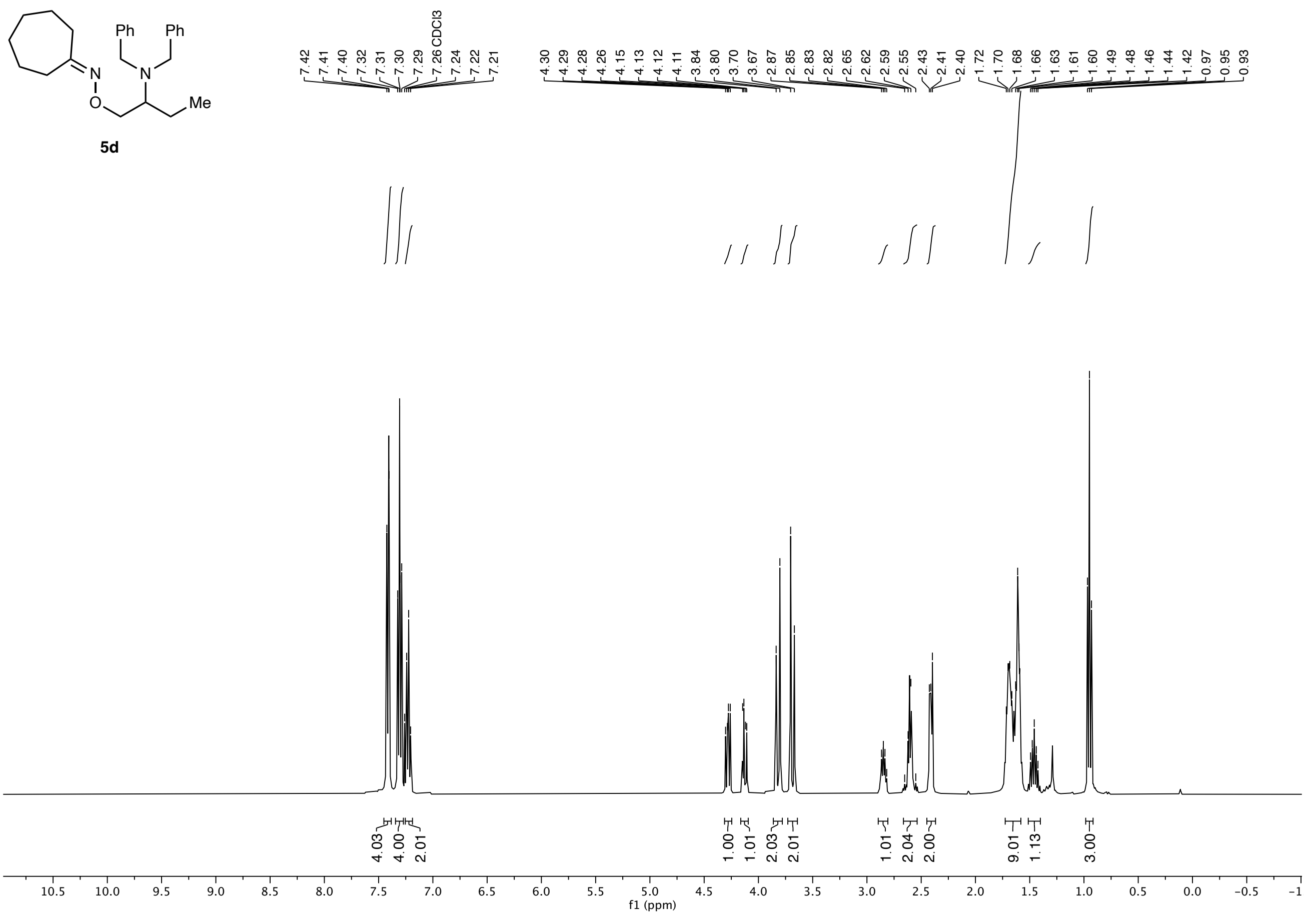
Ph
Ph
N
N
O
Me
7.42 7.41 7.40 7.32 7.31 7.30 7.29 7.26 CDCl3 7.24 7.22 7.21
4.30 4.29 4.28 4.26 4.15 4.13 4.12 4.11 3.84 3.80 3.70 3.67 2.87 2.85 2.83 2.82 2.65 2.62 2.59 2.55 2.43 2.41 2.40 1.72 1.70 1.68 1.66 1.63 1.61 1.60 1.49 1.48 1.46 1.44 1.42 0.97 0.95 0.93
4.03 4.00 2.01 1.00 1.01 2.03 2.01 1.01 2.04 2.00 9.01 1.13 3.00
10.5 10.0 9.5 9.0 8.5 8.0 7.5 7.0 6.5 6.0 5.5 5.0 4.5 4.0 3.5 3.0 2.5 2.0 1.5 1.0 0.5 0.0 −0.5 −1
f1 (ppm)

5d

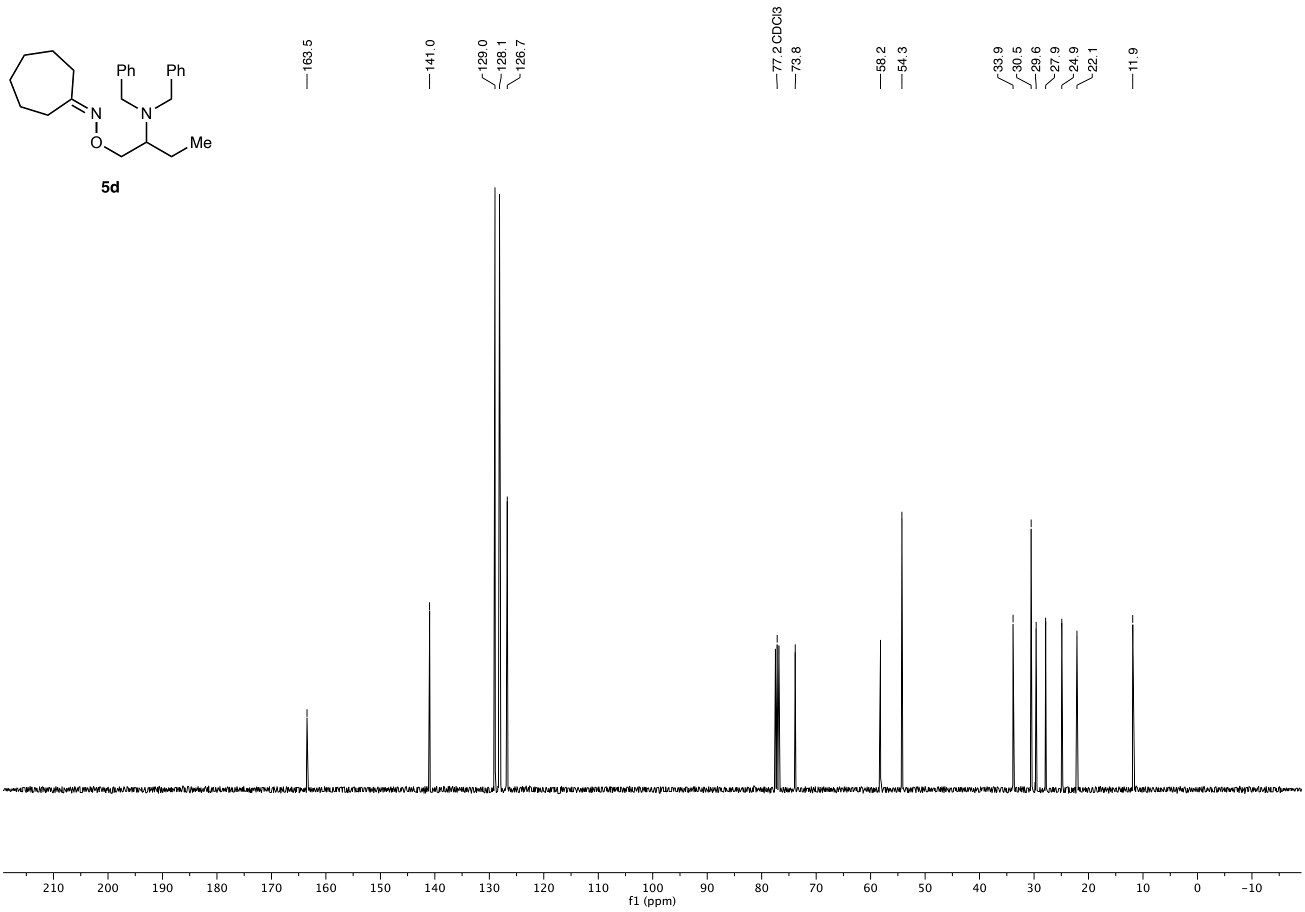
Ph
Ph
N
N
O
Me
163.5
141.0
129.0 128.1 126.7
77.2 CDCl3
73.8
58.2
54.3
33.9 30.5 29.6 27.9 24.9 22.1
11.9
210 200 190 180 170 160 150 140 130 120 110 100 90 80 70 60 50 40 30 20 10 0 −10
f1 (ppm)

5d

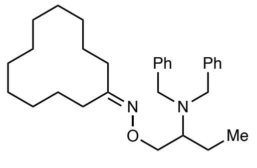

Ph
Ph
N
N
O
Me

5e

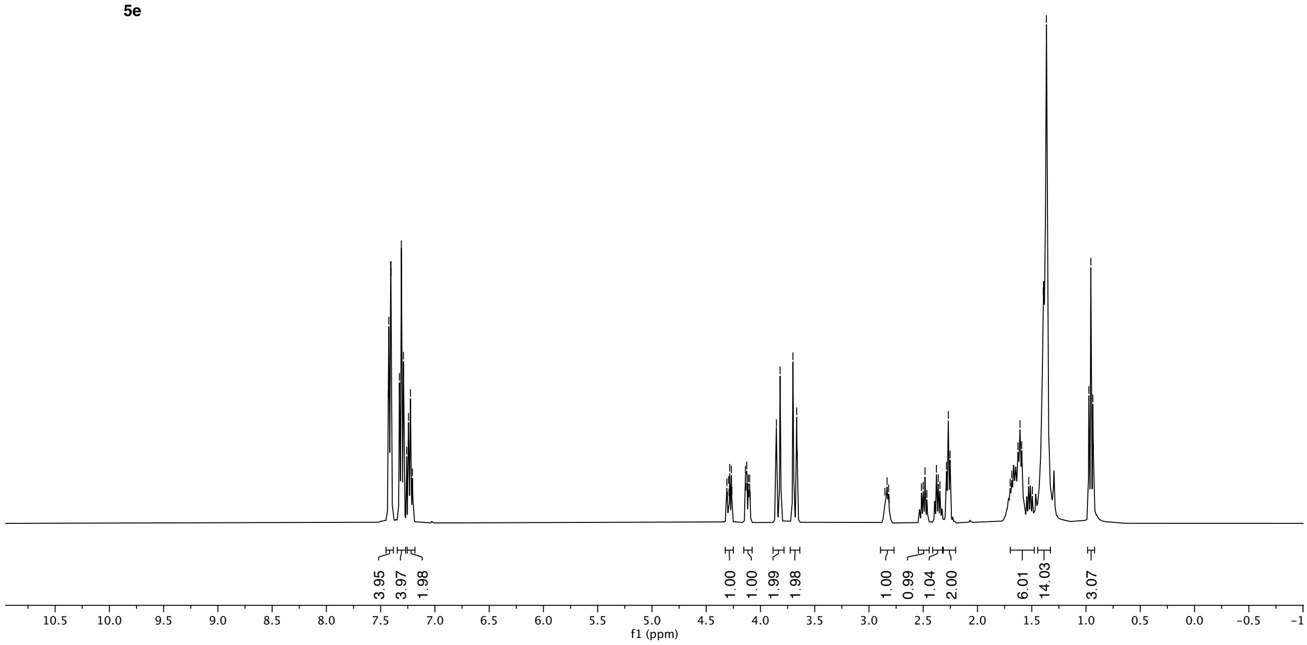

7.43 7.42 7.40 7.33 7.31 7.29 7.26 CDCl3 7.24 7.23 7.21 4.31 4.30 4.29 4.29 4.28 4.28 4.27 4.26 4.14 4.13 4.13 4.12 4.11 4.11 4.10 4.10 3.85 3.82 3.70 3.70 3.67 3.66 2.83 2.52 2.50 2.48 2.38 2.36 2.35 2.29 2.27 2.25 1.70 1.68 1.63 1.61 1.59 1.53 1.39 1.36 0.97 0.97 0.96 0.95 0.94 [illegible]
3.95 3.97 1.98 1.00 1.00 1.99 1.98 1.00 0.99 1.04 2.00 6.01 14.03 3.07
10.5 10.0 9.5 9.0 8.5 8.0 7.5 7.0 6.5 6.0 5.5 5.0 4.5 4.0 3.5 3.0 2.5 2.0 1.5 1.0 0.5 0.0 −0.5 −1
f1 (ppm)

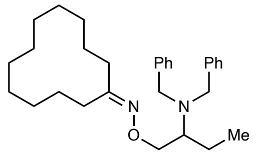

Ph
Ph
N
N
O
Me

5e

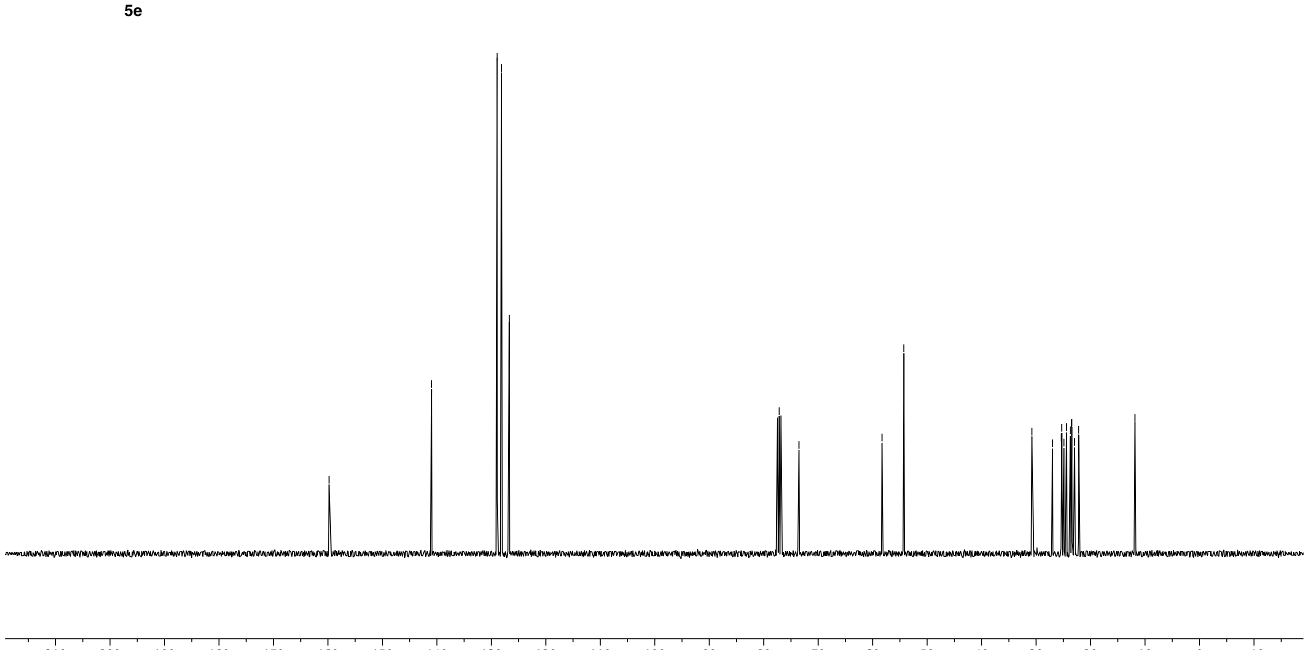

159.8 141.0 128.9 128.1 126.7 77.2 CDCl3 73.5 58.3 54.3 30.8 27.0 25.4 25.3 24.9 24.4 23.7 23.5 23.4 23.4 22.9 22.2 11.9
210 200 190 180 170 160 150 140 130 120 110 100 90 80 70 60 50 40 30 20 10 0 −10
f1 (ppm)

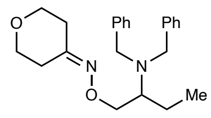
O
N
O
Ph
Ph
N
Me

5f

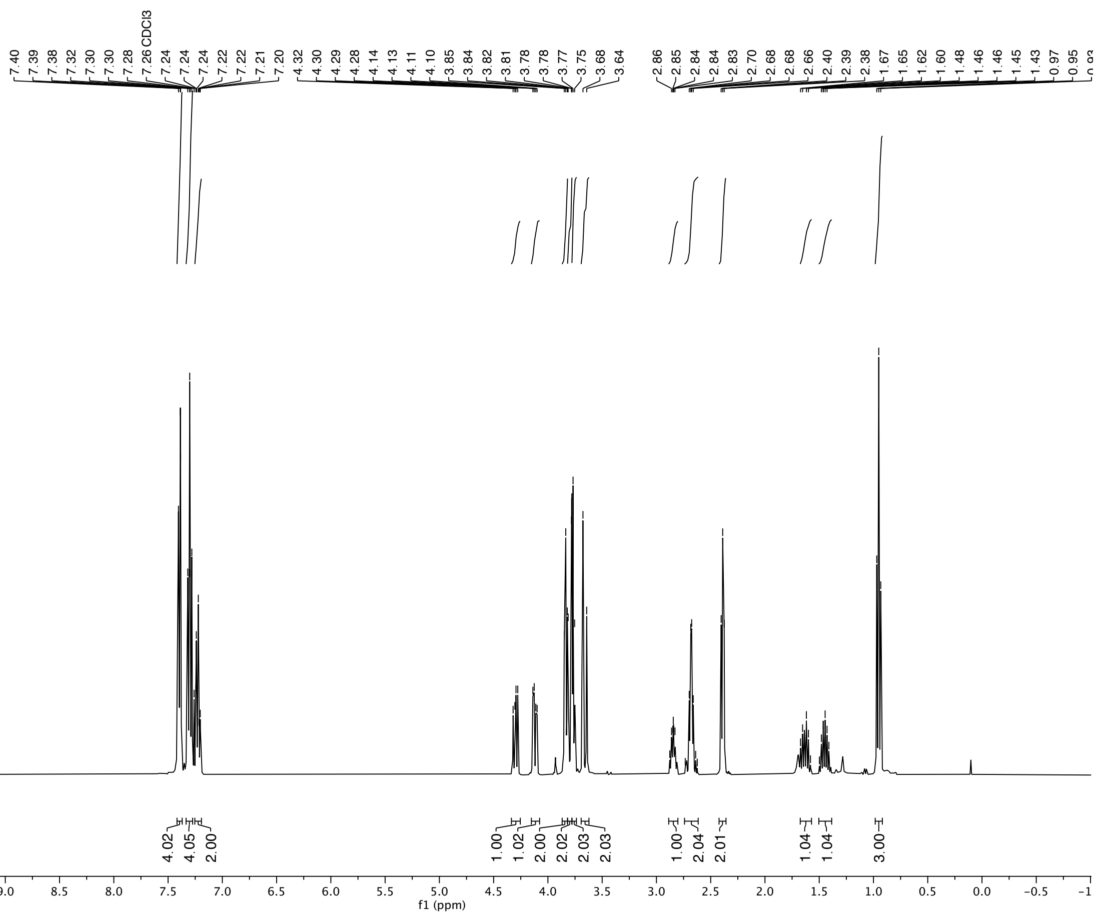
7.40 7.39 7.38 7.32 7.30 7.30 7.28 7.26 CDCl3 7.24 7.24 7.24 7.22 7.22 7.21 7.20
4.32 4.30 4.29 4.28 4.14 4.13 4.11 4.10 3.85 3.84 3.82 3.81 3.78 3.78 3.77 3.75 3.68 3.64
2.86 2.85 2.84 2.84 2.83 2.70 2.68 2.68 2.66 2.40 2.39 2.38 1.67 1.65 1.62 1.60 1.48 1.46 1.46 1.45 1.43 0.97 0.95
4.02 4.05 2.00
1.00 1.02 2.00 2.02 2.03 2.03
1.00 2.04 2.01
1.04 1.04
3.00
10.5 10.0 9.5 9.0 8.5 8.0 7.5 7.0 6.5 6.0 5.5 5.0 4.5 4.0 3.5 3.0 2.5 2.0 1.5 1.0 0.5 0.0 −0.5 −1
f1 (ppm)

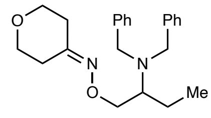
O
N
O
Ph
Ph
N
Me

5f

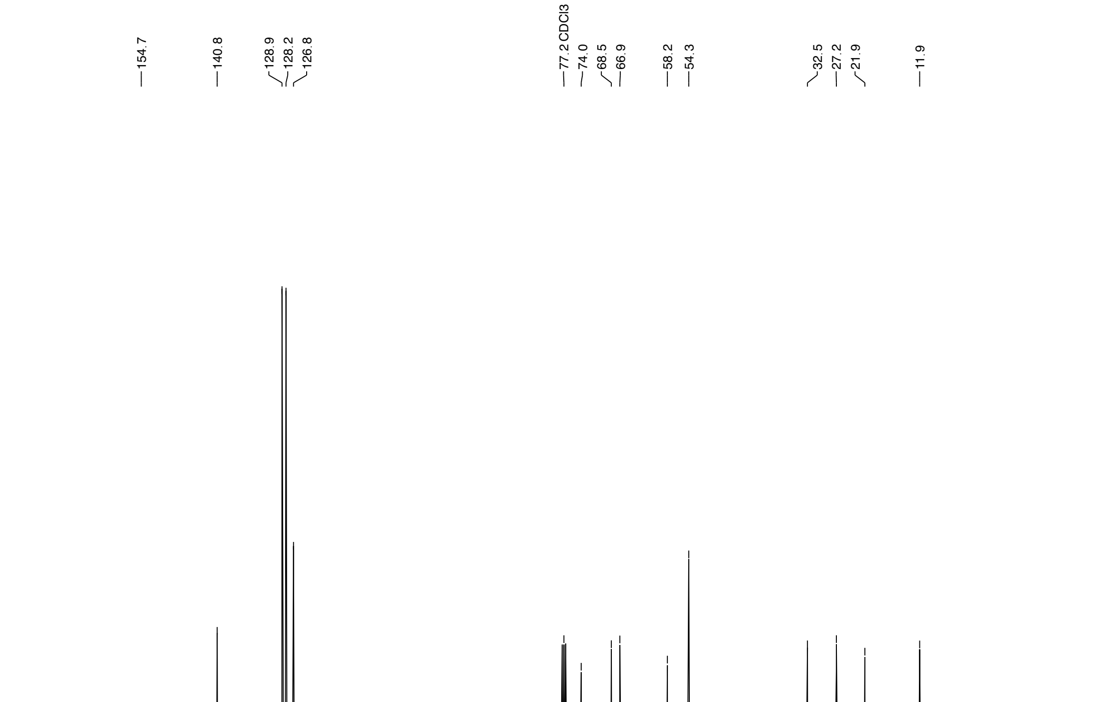
154.7
140.8
128.9 128.2 126.8
77.2 CDCl3
74.0 68.5 66.9
58.2 54.3
32.5 27.2 21.9
11.9
210 200 190 180 170 160 150 140 130 120 110 100 90 80 70 60 50 40 30 20 10 0 −10
f1 (ppm)

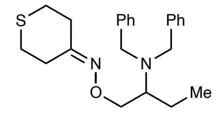
S
N
O
Ph
Ph
N
Me

5g

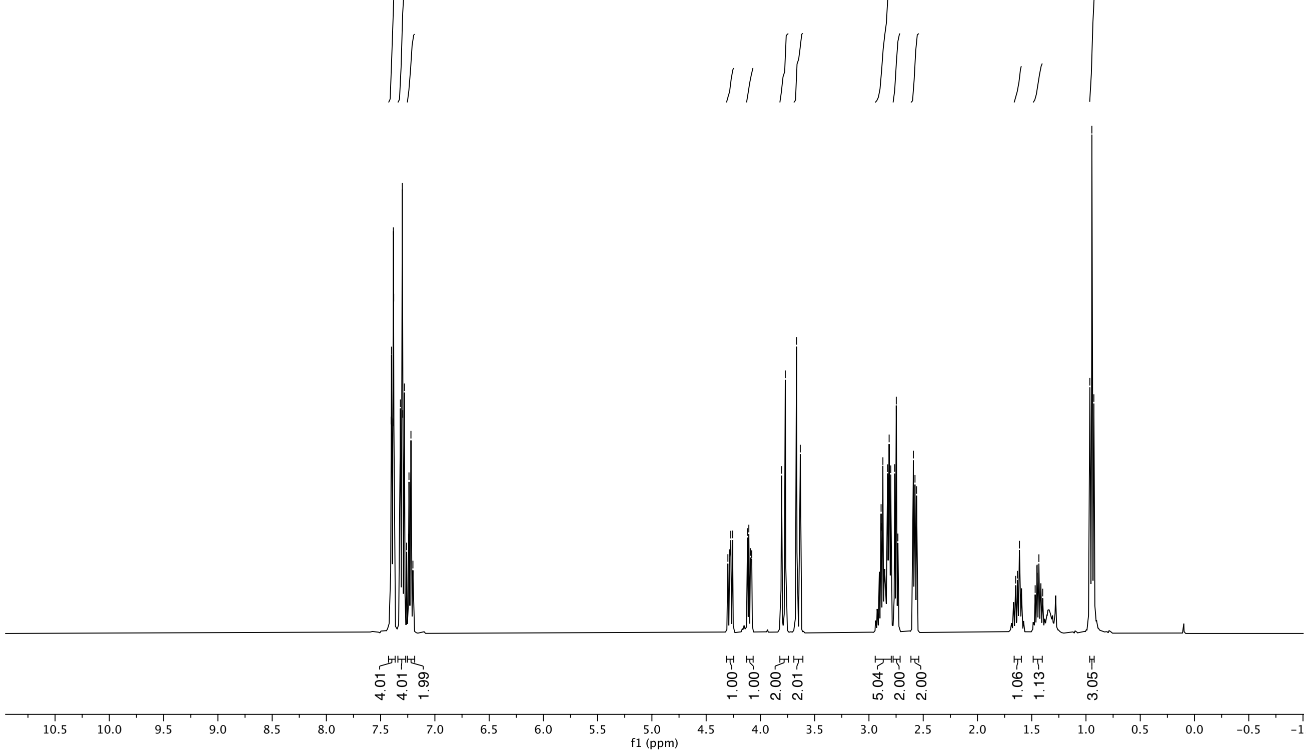
7.40 7.40 7.39 7.38 7.38 7.32 7.32 7.30 7.30 7.28 7.26 CDCl3 7.24 7.24 7.24 7.22 7.20
4.30 4.28 4.27 4.26 4.12 4.11 4.09 4.08 3.81 3.77 3.67 3.63
2.89 2.89 2.89 2.87 2.87 2.83 2.81 2.80 2.76 2.75 2.73 2.59 2.58 2.58 2.57 2.56 1.63 1.61 1.45 1.45 1.43 0.96 0.95
4.01 4.01 1.99 1.00 1.00 2.00 2.01 5.04 2.00 2.00 1.06 1.13 3.05
10.5 10.0 9.5 9.0 8.5 8.0 7.5 7.0 6.5 6.0 5.5 5.0 4.5 4.0 3.5 3.0 2.5 2.0 1.5 1.0 0.5 0.0 −0.5 −1
f1 (ppm)

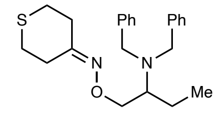
S
N
O
Ph
Ph
N
Me

5g

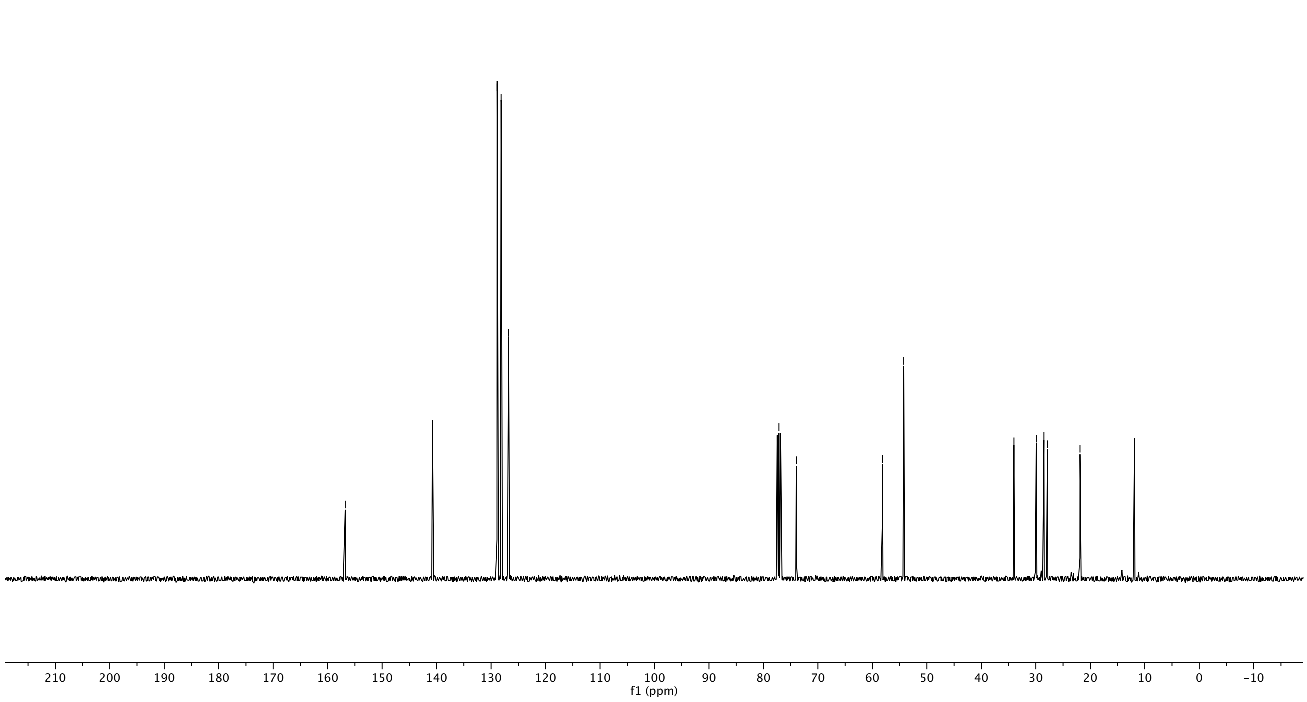
156.8
140.8
128.9 128.2 126.8
77.2 CDCl3 74.0
58.2 54.2
34.0 29.9 28.5 27.9 21.9
11.9
210 200 190 180 170 160 150 140 130 120 110 100 90 80 70 60 50 40 30 20 10 0 −10
f1 (ppm)

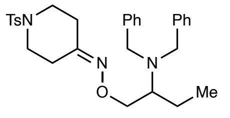
TsN
Ph
Ph
N
N
O
Me

5h

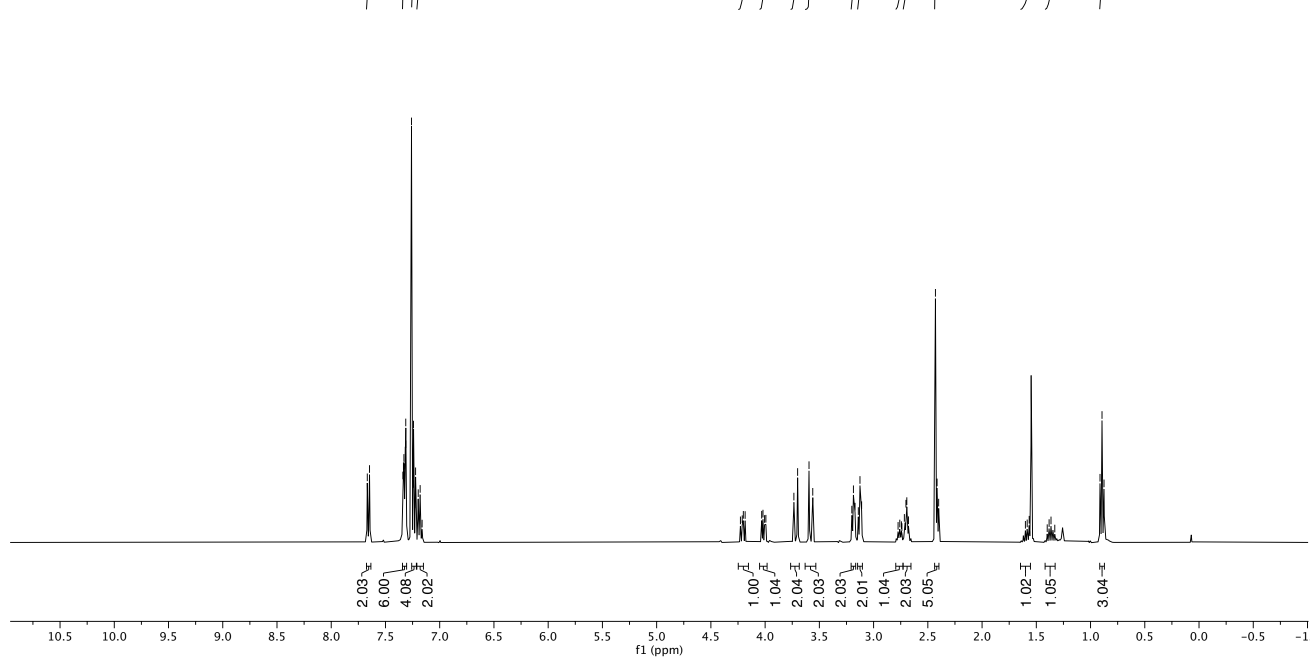
7.67
7.65
7.34
7.33
7.33
7.32
7.32
7.31
7.31
7.26 CDCl3
7.24
7.24
7.23
7.22
7.20
7.18
7.16
4.23
4.21
4.20
4.18
4.03
4.02
4.01
3.99
3.73
3.70
3.60
3.56
3.20
3.19
3.17
3.14
3.13
3.11
2.77
2.76
2.74
2.74
2.72
2.70
2.69
2.68
2.43
2.41
2.40
1.60
1.58
1.57
1.38
1.36
0.91
0.89
[illegible]
2.03
6.00
4.08
2.02
1.00
1.04
2.04
2.03
2.01
1.04
2.03
5.05
1.02
1.05
3.04
10.5
10.0
9.5
9.0
8.5
8.0
7.5
7.0
6.5
6.0
5.5
5.0
4.5
4.0
3.5
3.0
2.5
2.0
1.5
1.0
0.5
0.0
−0.5
[illegible]
f1 (ppm)

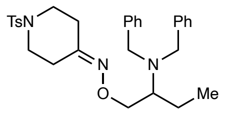
TsN
Ph
Ph
N
N
O
Me

5h

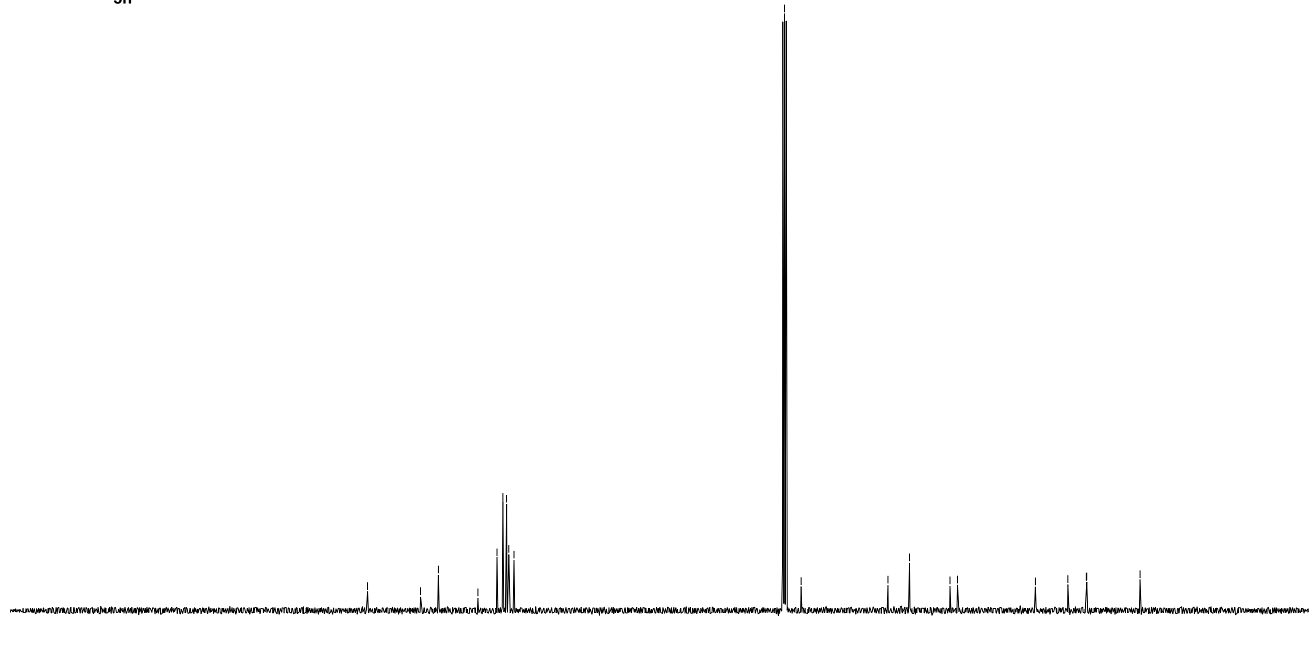
153.7
144.0
140.7
133.4
129.9
128.9
128.2
127.8
126.8
77.2 CDCl3
74.1
58.2
54.2
46.8
45.4
31.1
25.1
21.8
21.7
11.9
210
200
190
180
170
160
150
140
130
120
110
100
90
80
70
60
50
40
30
20
10
0
−10
f1 (ppm)

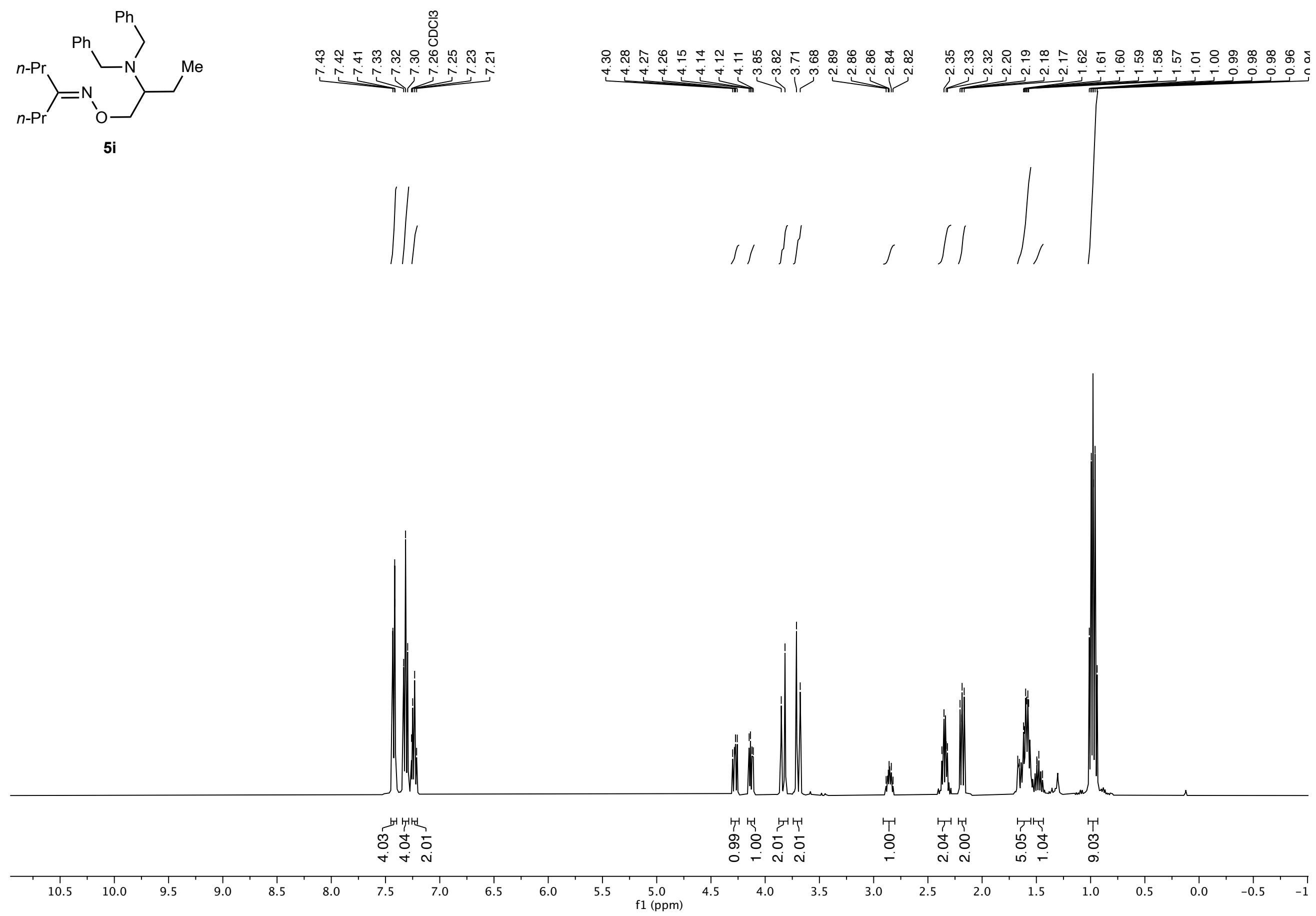
Ph
Ph
N
Me
n-Pr
N
O
n-Pr
5i
7.43 7.42 7.41 7.33 7.32 7.30 7.26 CDCl3 7.25 7.23 7.21
4.30 4.28 4.27 4.26 4.15 4.14 4.12 4.11 3.85 3.82 3.71 3.68
2.89 2.86 2.86 2.84 2.82
2.35 2.33 2.32 2.20 2.19 2.18 2.17 1.62 1.61 1.60 1.59 1.58 1.57 1.01 1.00 0.99 0.98 0.98 0.96
4.03 4.04 2.01 0.99 1.00 2.01 2.01 1.00 2.04 2.00 5.05 1.04 9.03
10.5 10.0 9.5 9.0 8.5 8.0 7.5 7.0 6.5 6.0 5.5 5.0 4.5 4.0 3.5 3.0 2.5 2.0 1.5 1.0 0.5 0.0 −0.5
f1 (ppm)

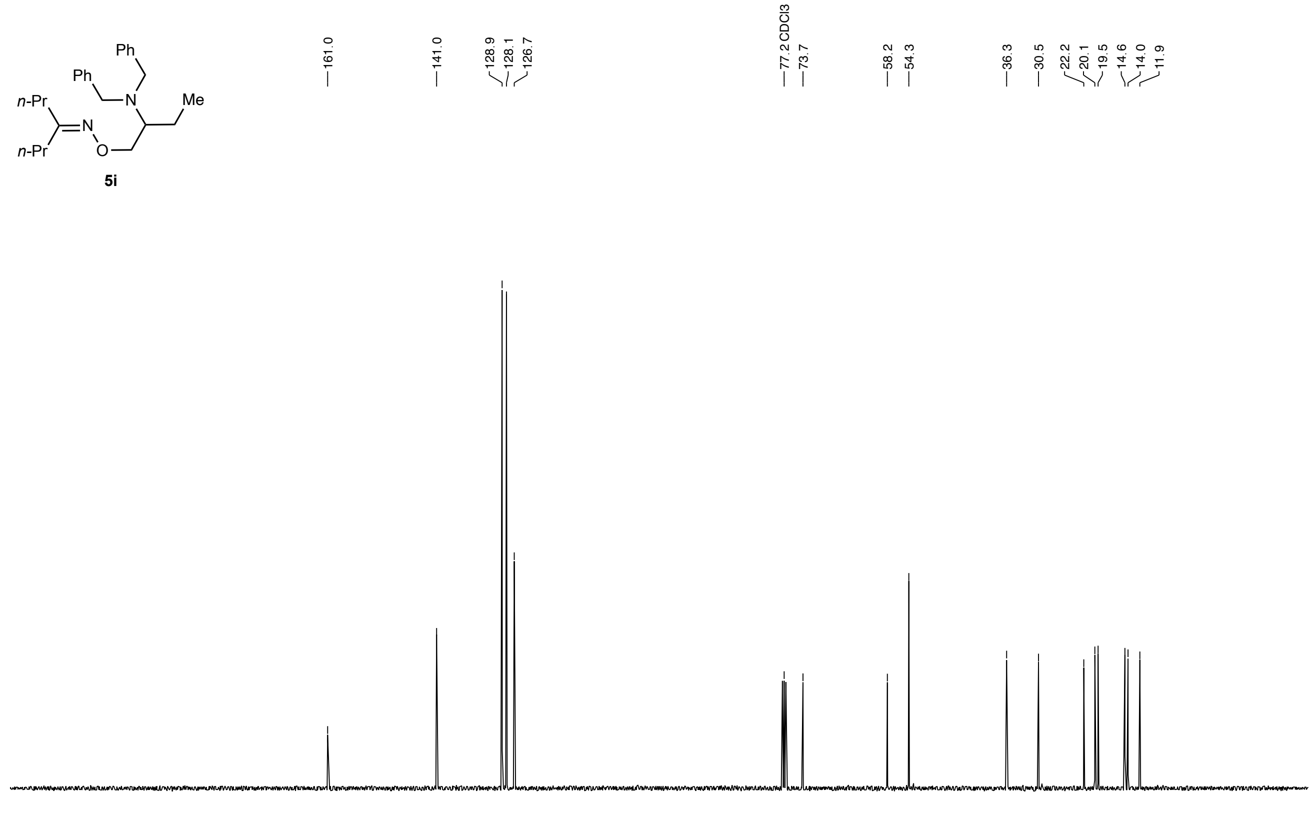
Ph
Ph
N
Me
n-Pr
N
O
n-Pr
5i
161.0
141.0
128.9 128.1 126.7
77.2 CDCl3
73.7
58.2
54.3
36.3
30.5
22.2 20.1 19.5 14.6 14.0 11.9
210 200 190 180 170 160 150 140 130 120 110 100 90 80 70 60 50 40 30 20 10 0 −10
f1 (ppm)

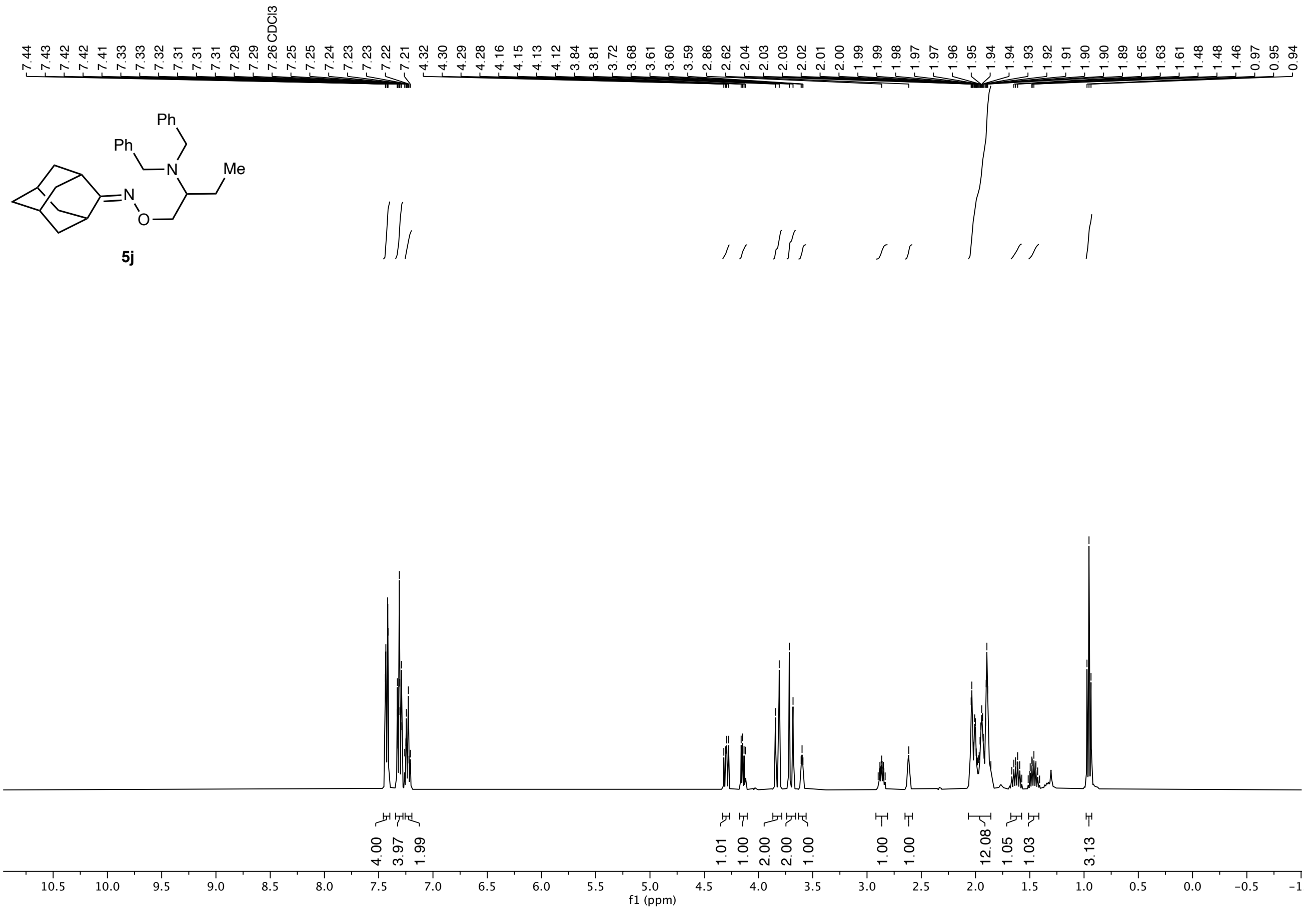

7.44 7.43 7.42 7.42 7.41 7.33 7.33 7.32 7.31 7.31 7.31 7.29 7.29 7.26 CDCl3 7.25 7.25 7.24 7.23 7.23 7.22 7.21 4.32 4.30 4.29 4.28 4.16 4.15 4.13 4.12 3.84 3.81 3.72 3.68 3.61 3.60 3.59 2.86 2.62 2.04 2.03 2.03 2.02 2.01 2.00 1.99 1.98 1.97 1.97 1.96 1.95 1.94 1.94 1.93 1.92 1.91 1.90 1.90 1.89 1.65 1.63 1.61 1.48 1.48 1.46 0.97 0.95 0.94
Ph
Ph
N
Me
N
O
5j
4.00
3.97
1.99
1.01
1.00
2.00
2.00
1.00
1.00
1.00
12.08
1.05
1.03
3.13
f1 (ppm)

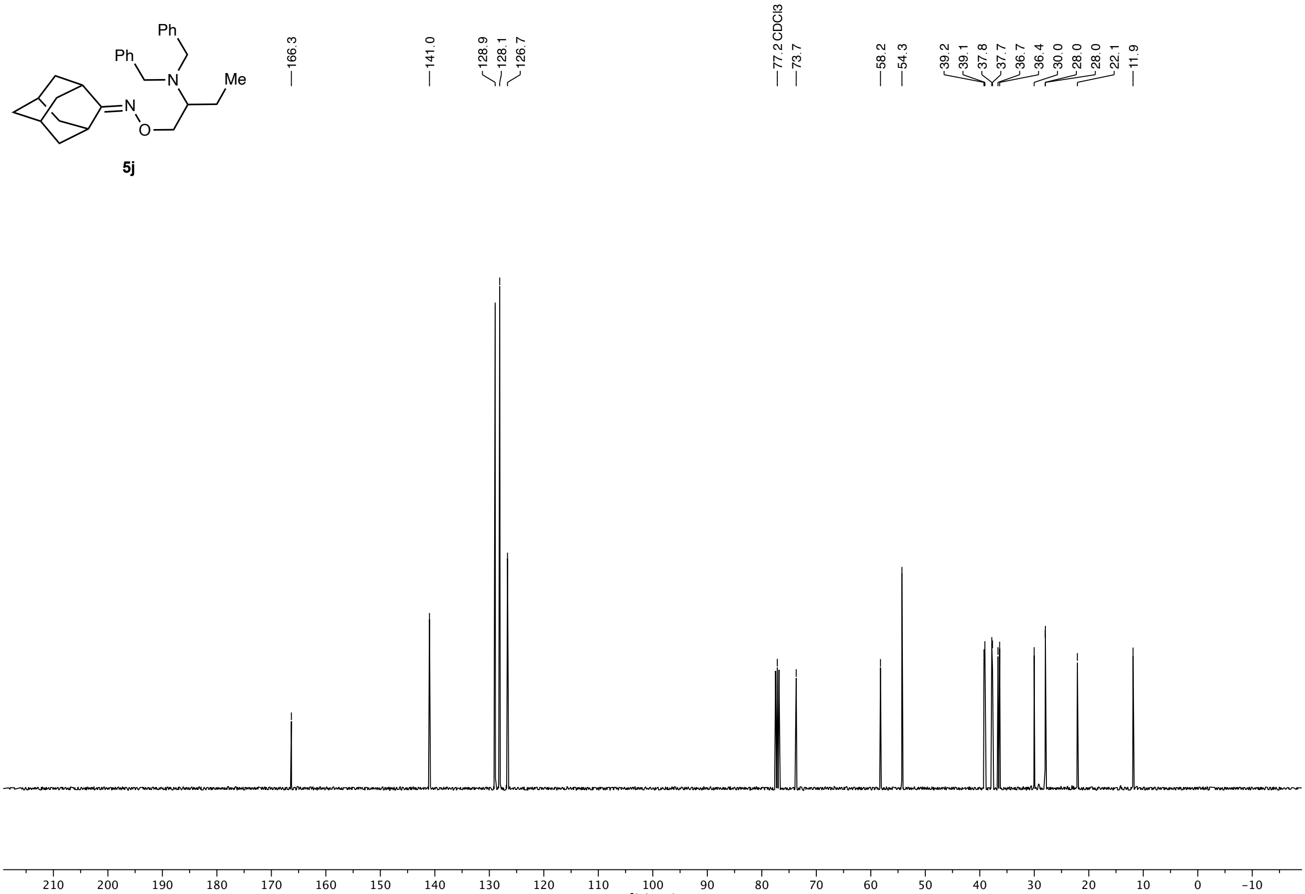

Ph
Ph
N
Me
N
O
5j
166.3
141.0
128.9
128.1
126.7
77.2 CDCl3
73.7
58.2
54.3
39.2
39.1
37.8
37.7
36.7
36.4
30.0
28.0
28.0
22.1
11.9
f1 (ppm)

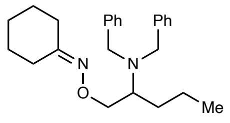

5k

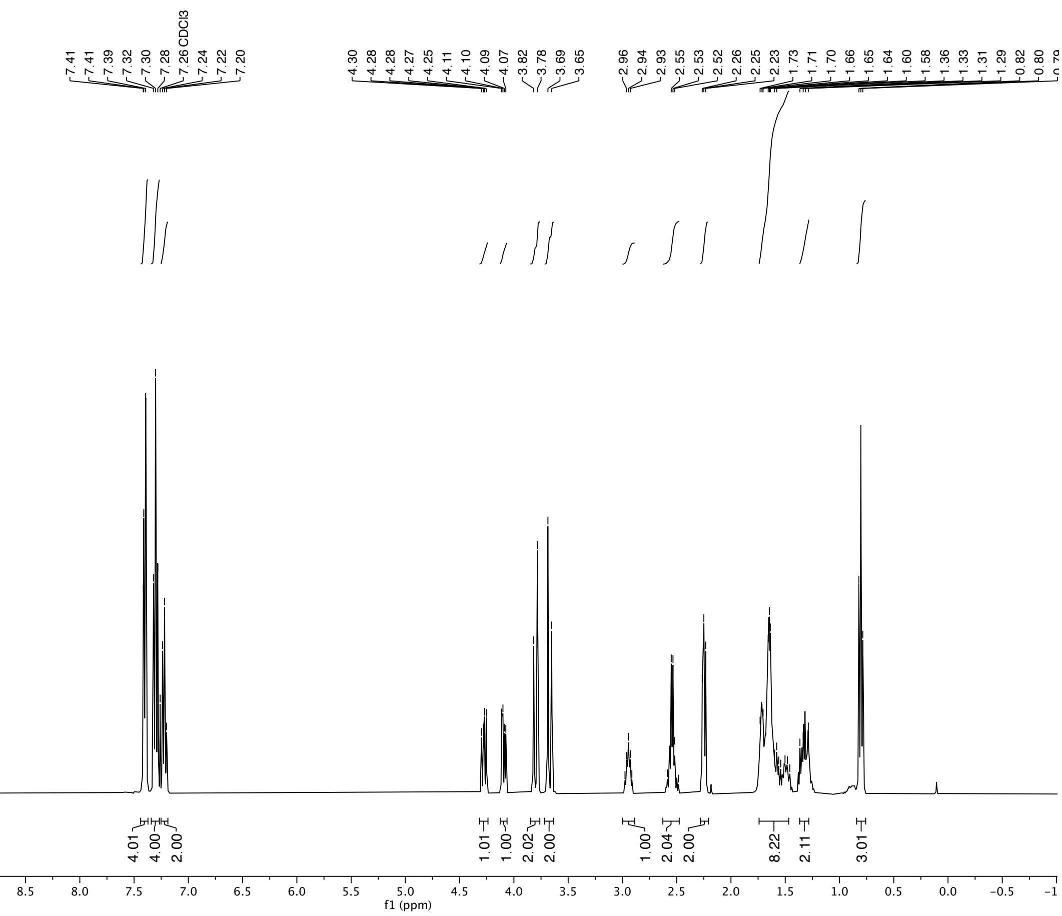
7.41
7.41
7.39
7.32
7.30
7.28
7.26 CDCl3
7.24
7.22
7.20
4.30
4.28
4.28
4.27
4.25
4.11
4.10
4.09
4.07
3.82
3.78
3.69
3.65
2.96
2.94
2.93
2.55
2.53
2.52
2.26
2.25
2.23
1.73
1.71
1.70
1.66
1.65
1.64
1.60
1.58
1.36
1.33
1.31
1.29
0.82
0.80
4.01
4.00
2.00
1.01
1.00
2.02
2.00
1.00
2.04
2.00
8.22
2.11
3.01
f1 (ppm)

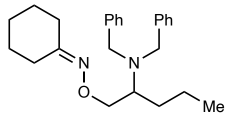

5k

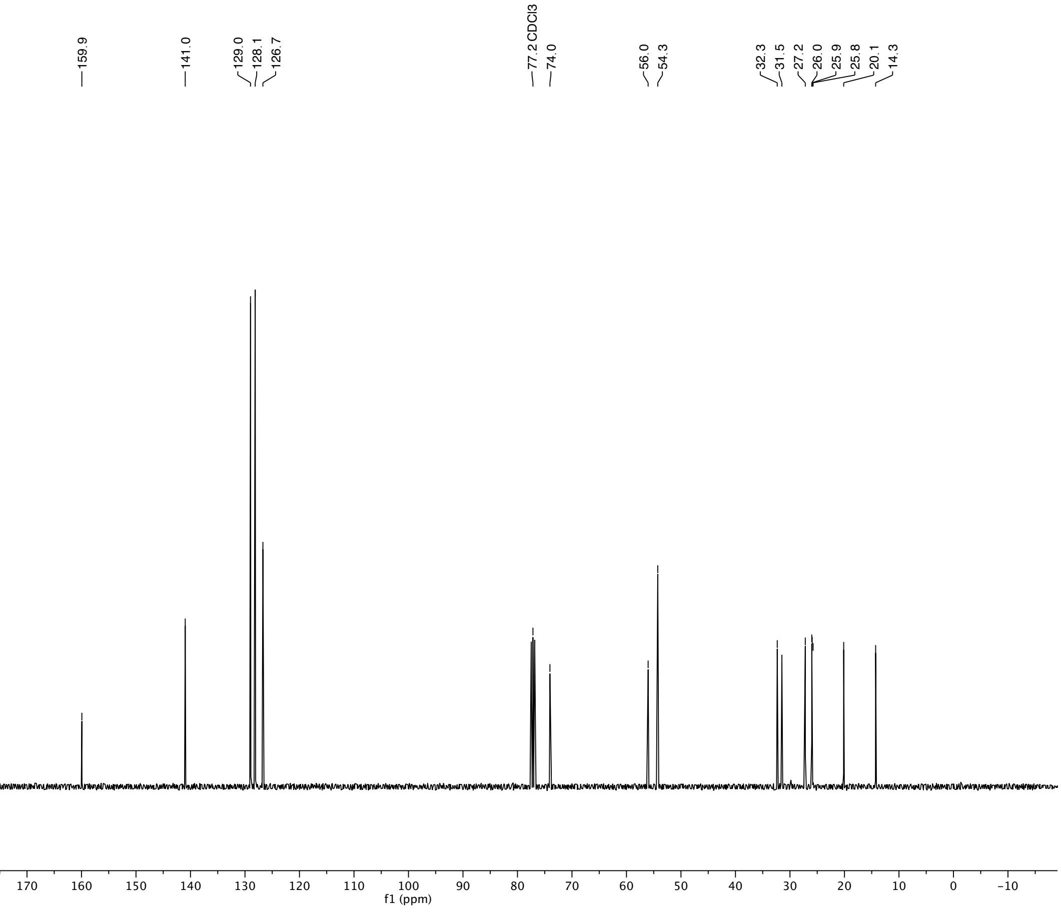
159.9
141.0
129.0
128.1
126.7
77.2 CDCl3
74.0
56.0
54.3
32.3
31.5
27.2
26.0
25.9
25.8
20.1
14.3
f1 (ppm)

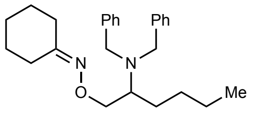
Ph
Ph
N
N
O
Me

5l

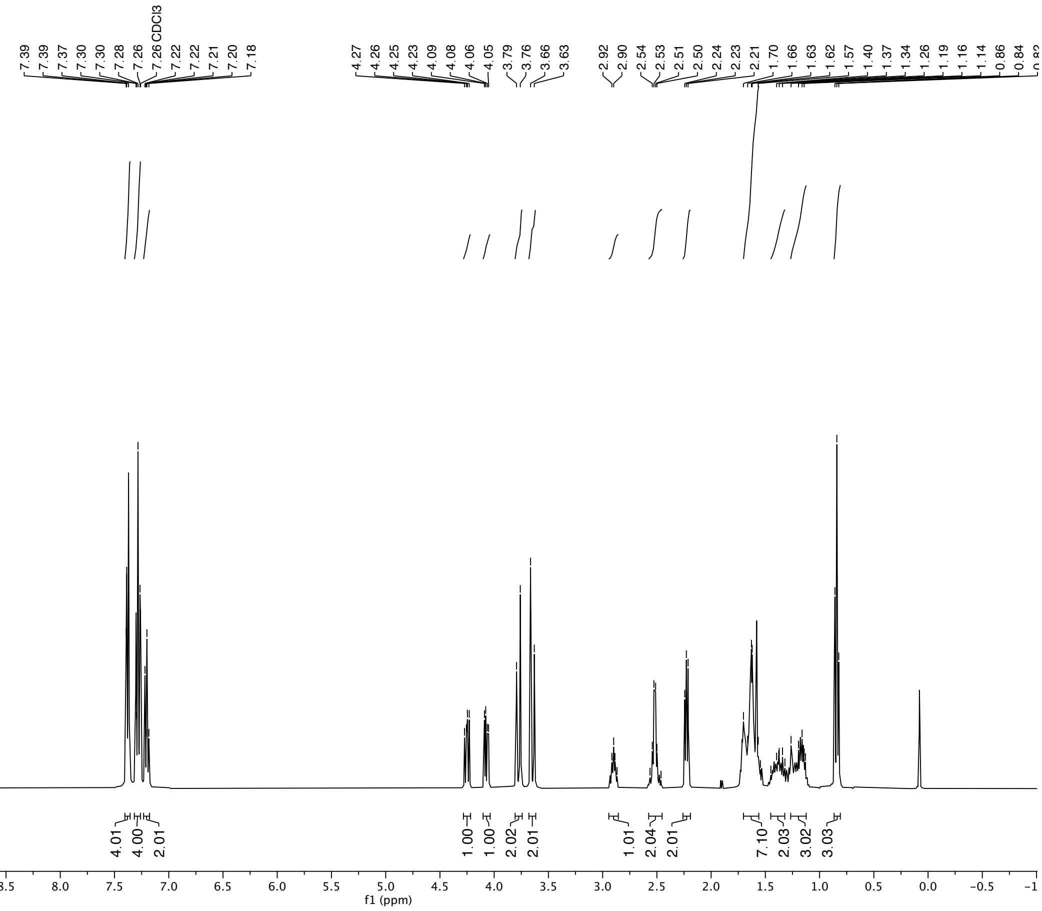
7.39 7.39 7.37 7.30 7.30 7.28 7.26 7.26 CDCl3 7.22 7.22 7.21 7.20 7.18
4.27 4.26 4.25 4.23 4.09 4.08 4.06 4.05 3.79 3.76 3.66 3.63
2.92 2.90 2.54 2.53 2.51 2.50 2.24 2.23 2.21 1.70 1.66 1.63 1.62 1.57 1.40 1.37 1.34 1.26 1.19 1.16 1.14 0.86 0.84 [illegible]
4.01 4.00 2.01 1.00 1.00 2.02 2.01 1.01 2.04 2.01 7.10 2.03 3.02 3.03
f1 (ppm)

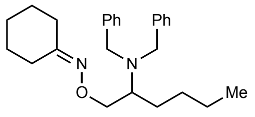
Ph
Ph
N
N
O
Me

5l

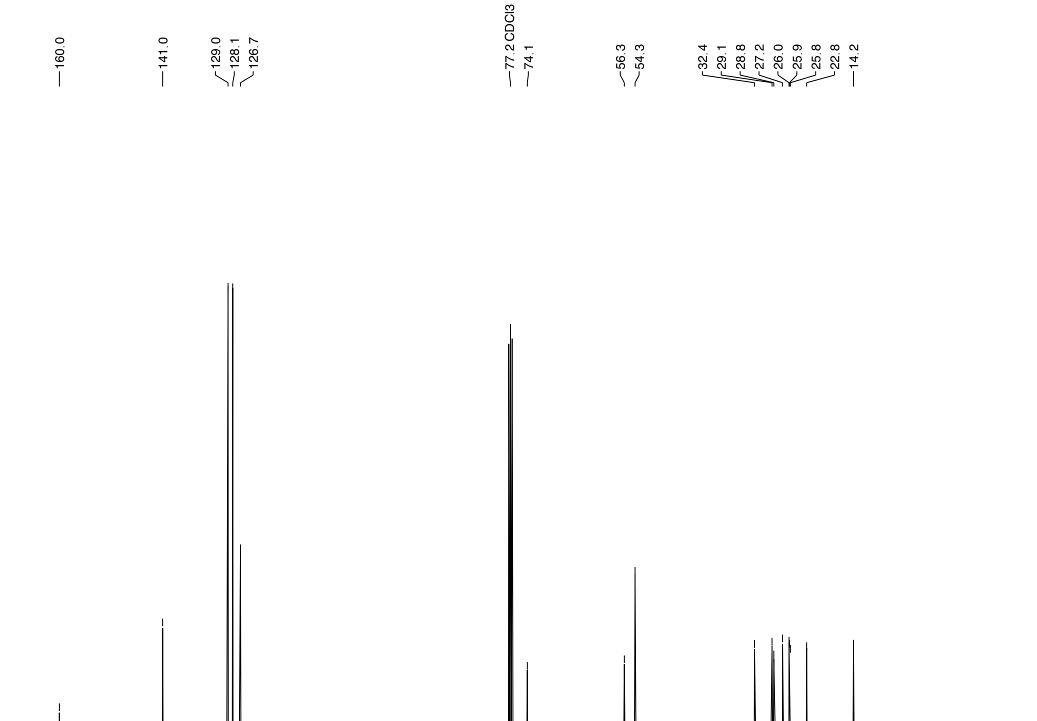
160.0
141.0
129.0 128.1 126.7
77.2 CDCl3 74.1
56.3 54.3
32.4 29.1 28.8 27.2 26.0 25.9 25.8 22.8 14.2
f1 (ppm)

5m

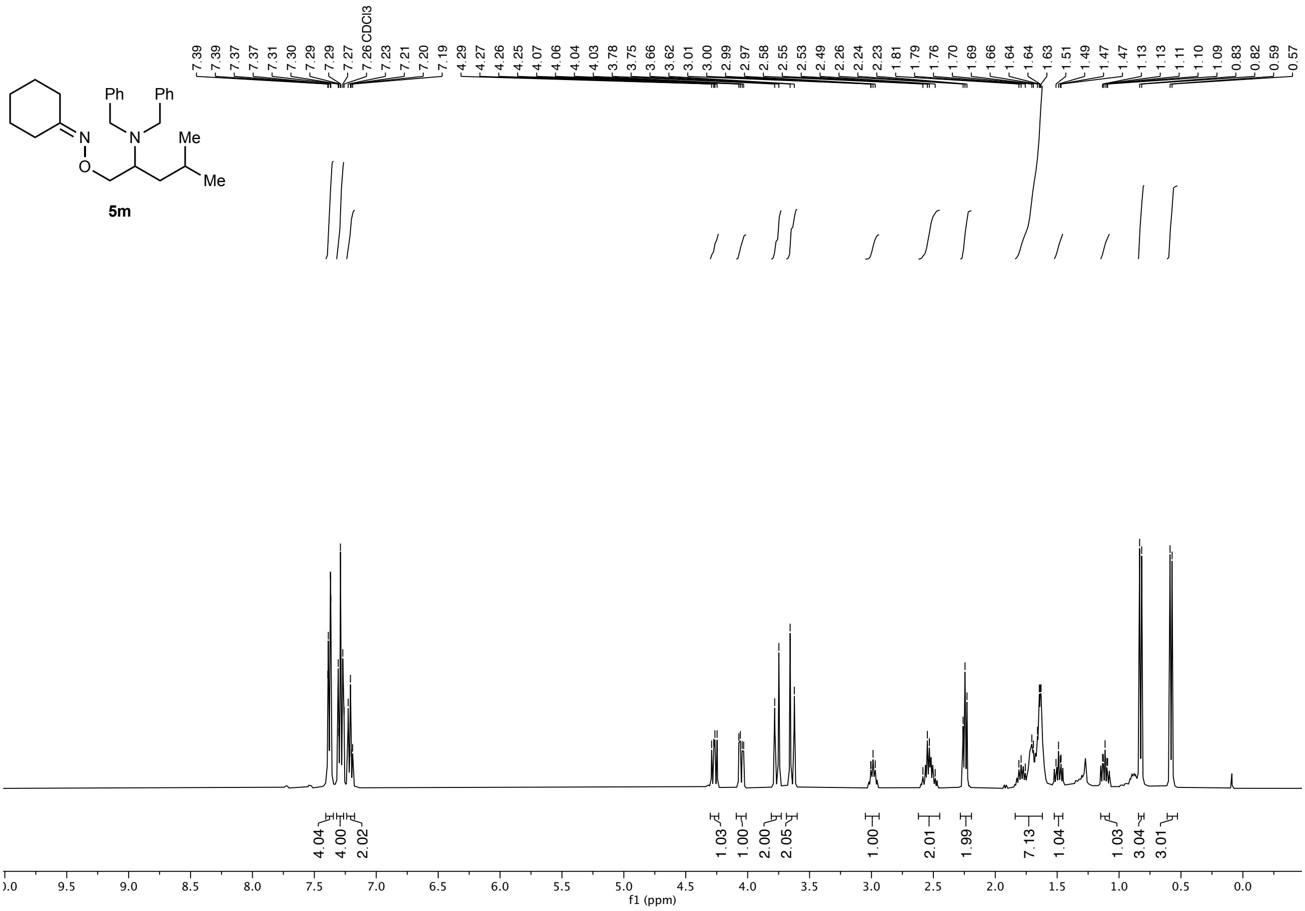
7.39 7.39 7.37 7.37 7.31 7.30 7.29 7.29 7.27 7.26 CDCl3 7.23 7.21 7.20 7.19 4.29 4.27 4.26 4.25 4.07 4.06 4.04 4.03 3.78 3.75 3.66 3.62 3.01 3.00 2.99 2.97 2.58 2.55 2.53 2.49 2.26 2.24 2.23 1.81 1.79 1.76 1.70 1.69 1.66 1.64 1.64 1.63 1.51 1.49 1.47 1.47 1.13 1.13 1.11 1.10 1.09 0.83 0.82 0.59 0.57
Ph
Ph
N
O
Me
Me
4.04 4.00 2.02 1.03 1.00 2.00 2.05 1.00 2.01 1.99 7.13 1.04 1.03 3.04 3.01
f1 (ppm)

5m

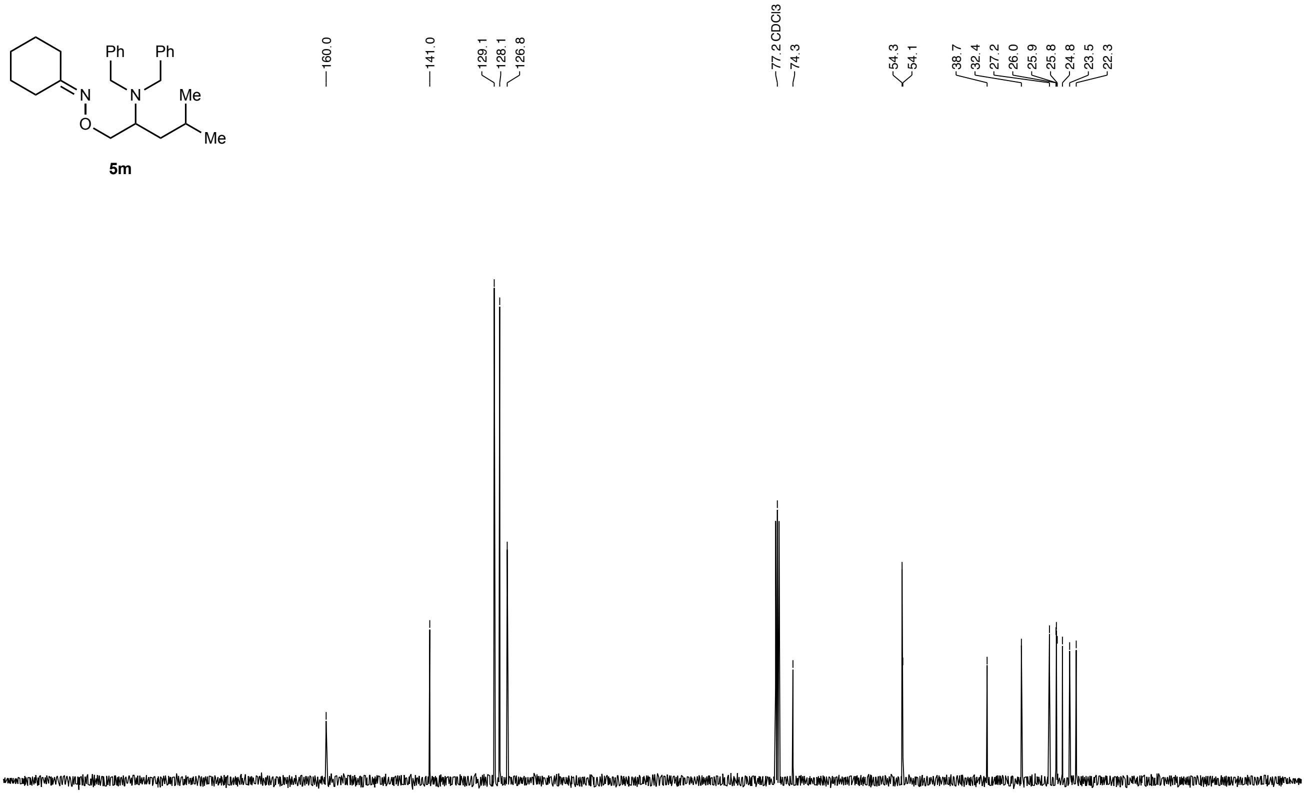
160.0 141.0 129.1 128.1 126.8 77.2 CDCl3 74.3 54.3 54.1 38.7 32.4 27.2 26.0 25.9 25.8 24.8 23.5 22.3
Ph
Ph
N
O
Me
Me
f1 (ppm)

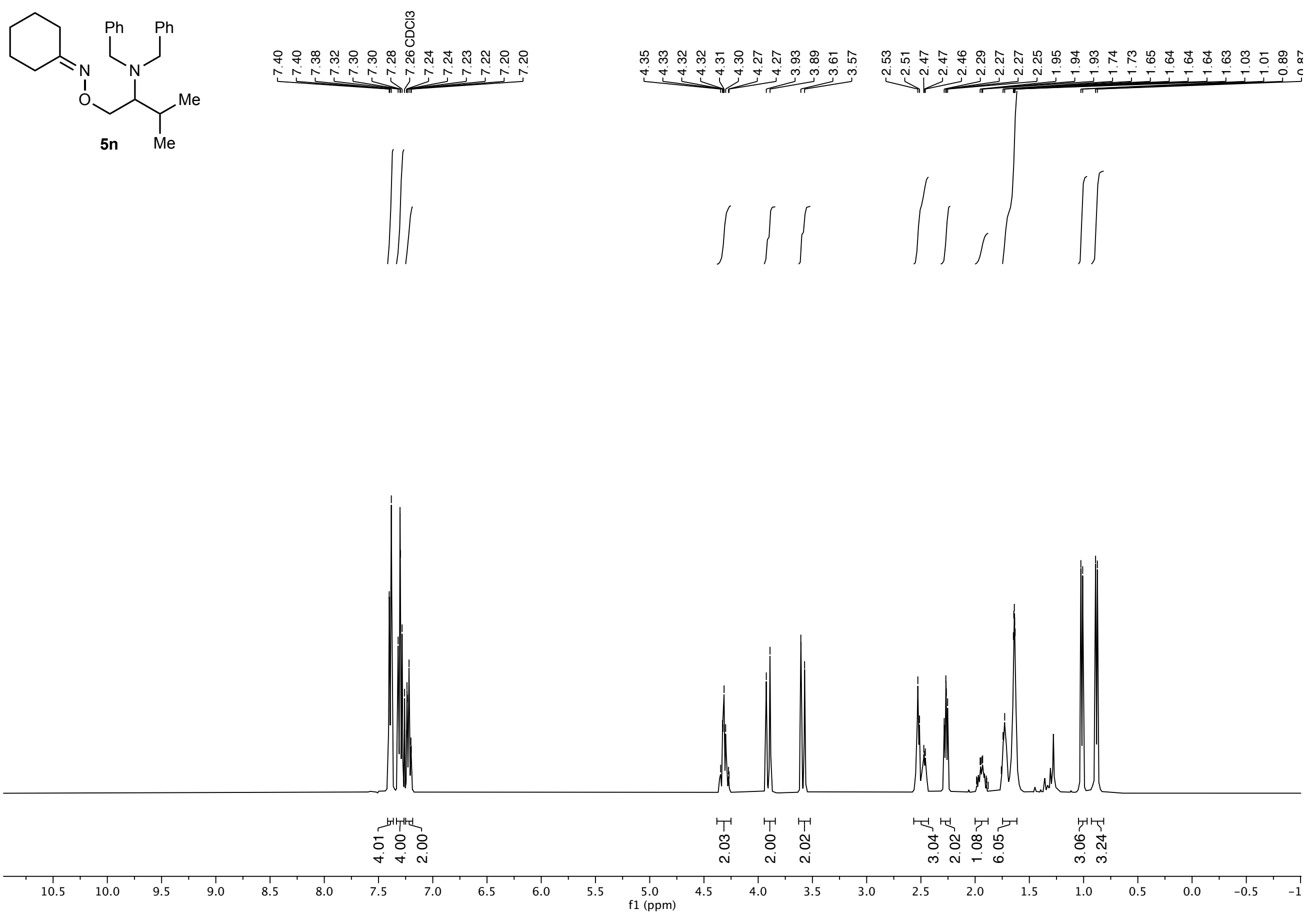
Ph
Ph
N
N
O
Me
Me
5n
7.40 7.40 7.38 7.32 7.30 7.30 7.28 7.26 CDCl3 7.24 7.24 7.23 7.22 7.20 7.20
4.35 4.33 4.32 4.32 4.31 4.30 4.27 4.27 3.93 3.89 3.61 3.57
2.53 2.51 2.47 2.47 2.46 2.29 2.27 2.27 2.25 1.95 1.94 1.93 1.74 1.73 1.65 1.64 1.64 1.64 1.63 1.03 1.01 0.89
4.01 4.00 2.00 2.03 2.00 2.02 3.04 2.02 1.08 6.05 3.06 3.24
f1 (ppm)

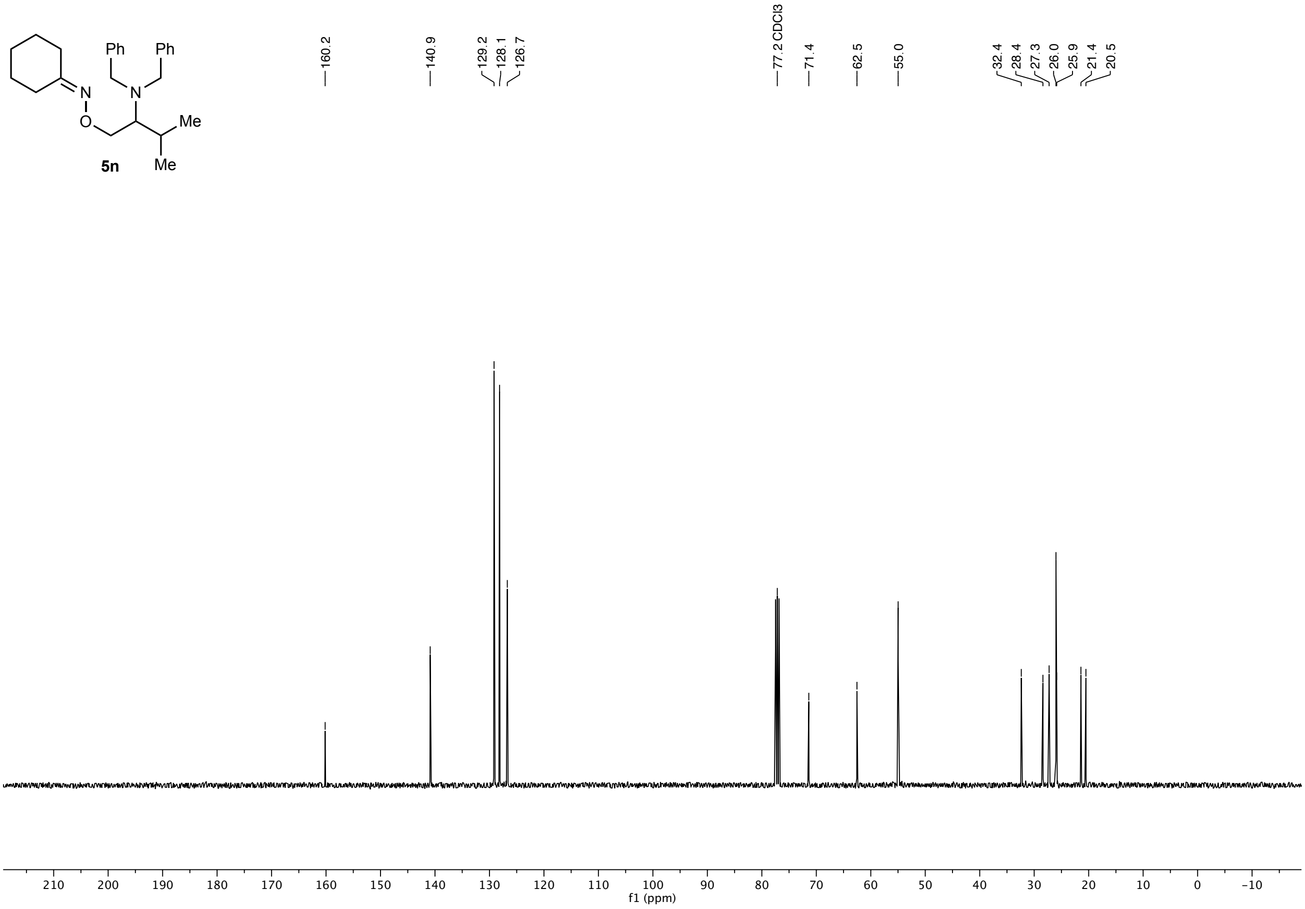
Ph
Ph
N
N
O
Me
Me
5n
160.2
140.9
129.2 128.1 126.7
77.2 CDCl3
71.4
62.5
55.0
32.4 28.4 27.3 26.0 25.9 21.4 20.5
f1 (ppm)

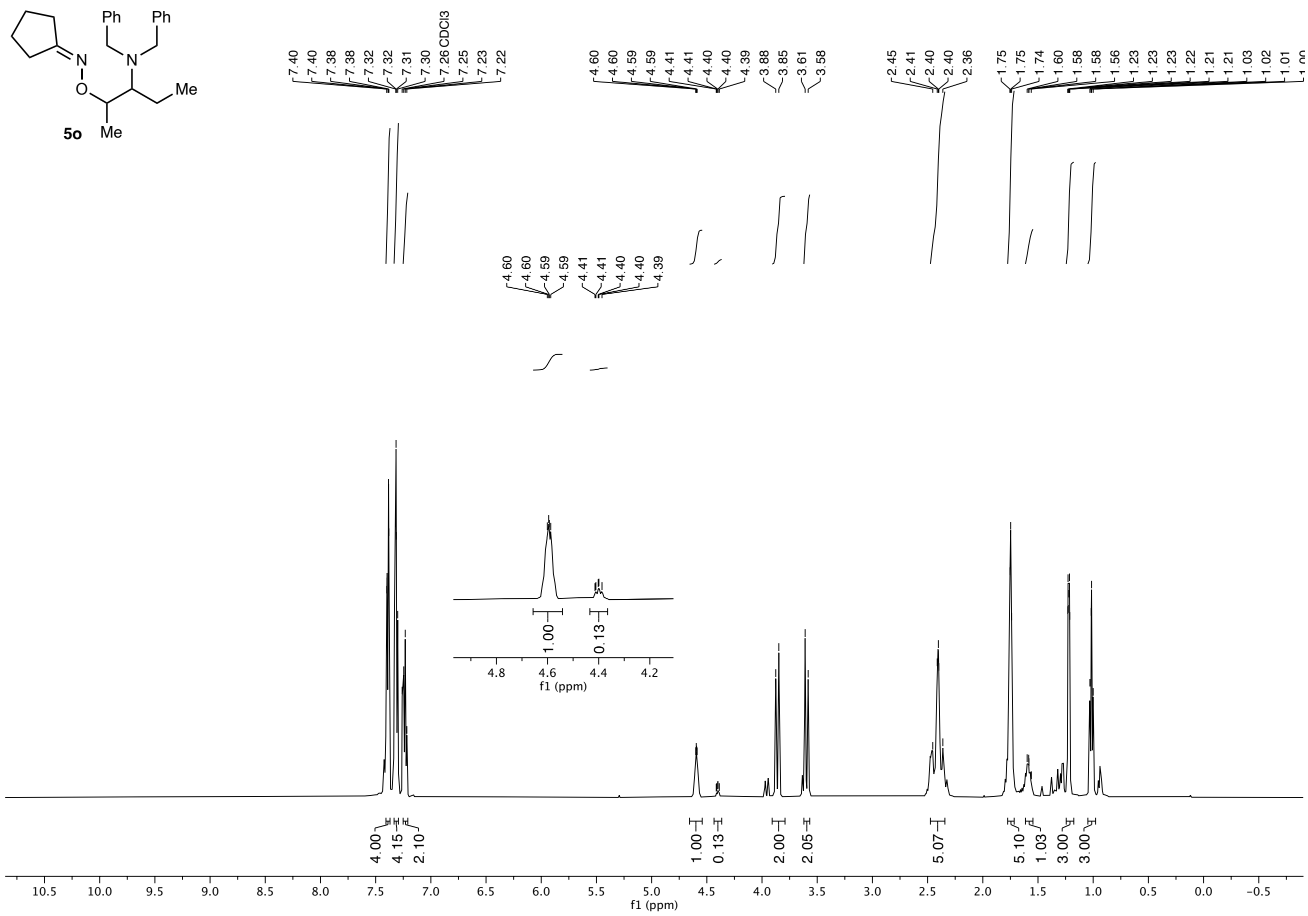
Ph
Ph
N
O
N
Me
Me
5o
7.40 7.40 7.38 7.38 7.32 7.32 7.31 7.30 7.26 CDCl3 7.25 7.23 7.22
4.60 4.60 4.59 4.59 4.41 4.41 4.40 4.40 4.39 3.88 3.85 3.61 3.58
2.45 2.41 2.40 2.40 2.36
1.75 1.75 1.74 1.60 1.58 1.58 1.56 1.23 1.23 1.23 1.22 1.21 1.21 1.03 1.02 1.01
4.60 4.60 4.59 4.59 4.41 4.41 4.40 4.40 4.39
1.00
0.13
4.8 4.6 4.4 4.2
f1 (ppm)
4.00 4.15 2.10
1.00 0.13
2.00 2.05
5.07
5.10 1.03 3.00 3.00
10.5 10.0 9.5 9.0 8.5 8.0 7.5 7.0 6.5 6.0 5.5 5.0 4.5 4.0 3.5 3.0 2.5 2.0 1.5 1.0 0.5 0.0 -0.5
f1 (ppm)

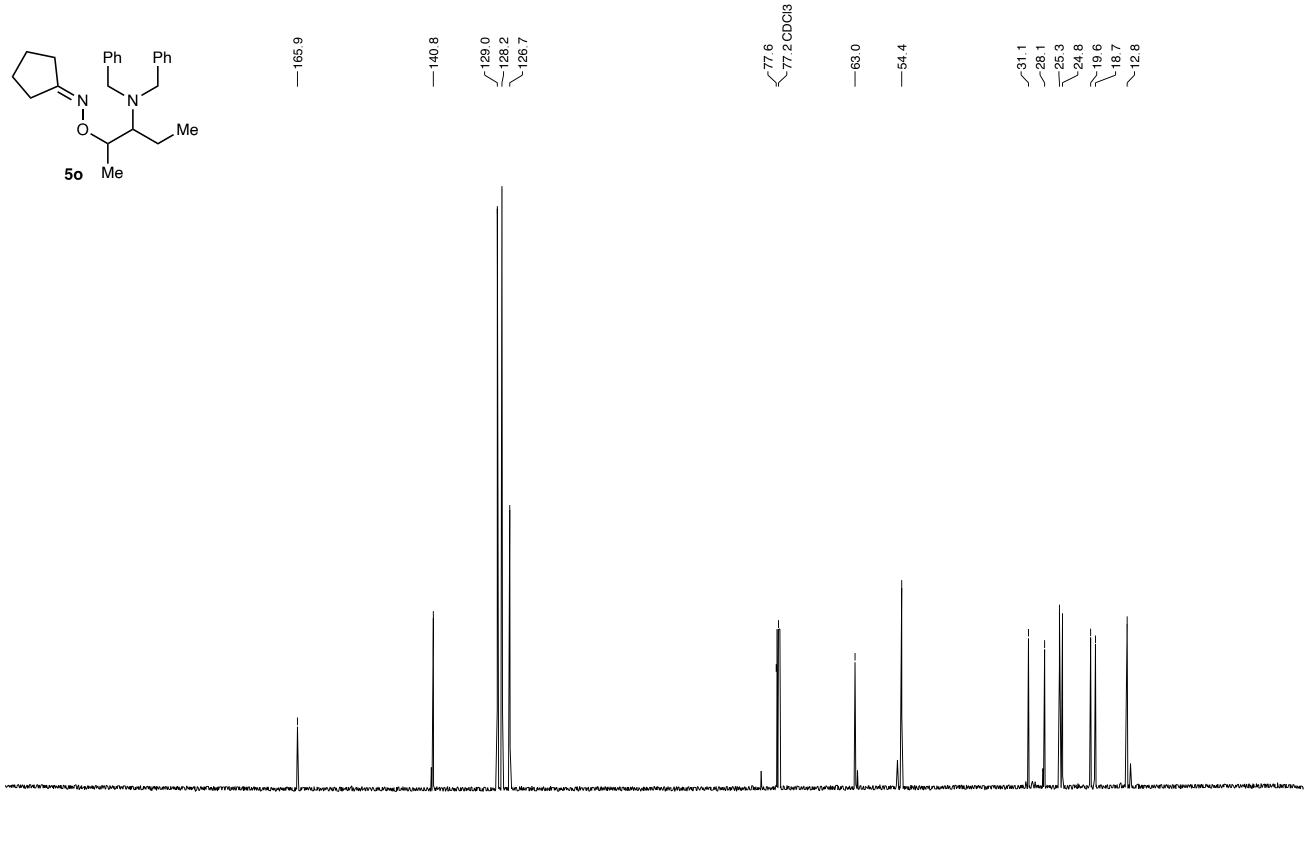
Ph
Ph
N
O
N
Me
Me
5o
165.9
140.8
129.0 128.2 126.7
77.6 77.2 CDCl3
63.0
54.4
31.1 28.1 25.3 24.8 19.6 18.7 12.8
210 200 190 180 170 160 150 140 130 120 110 100 90 80 70 60 50 40 30 20 10 0 -10
f1 (ppm)

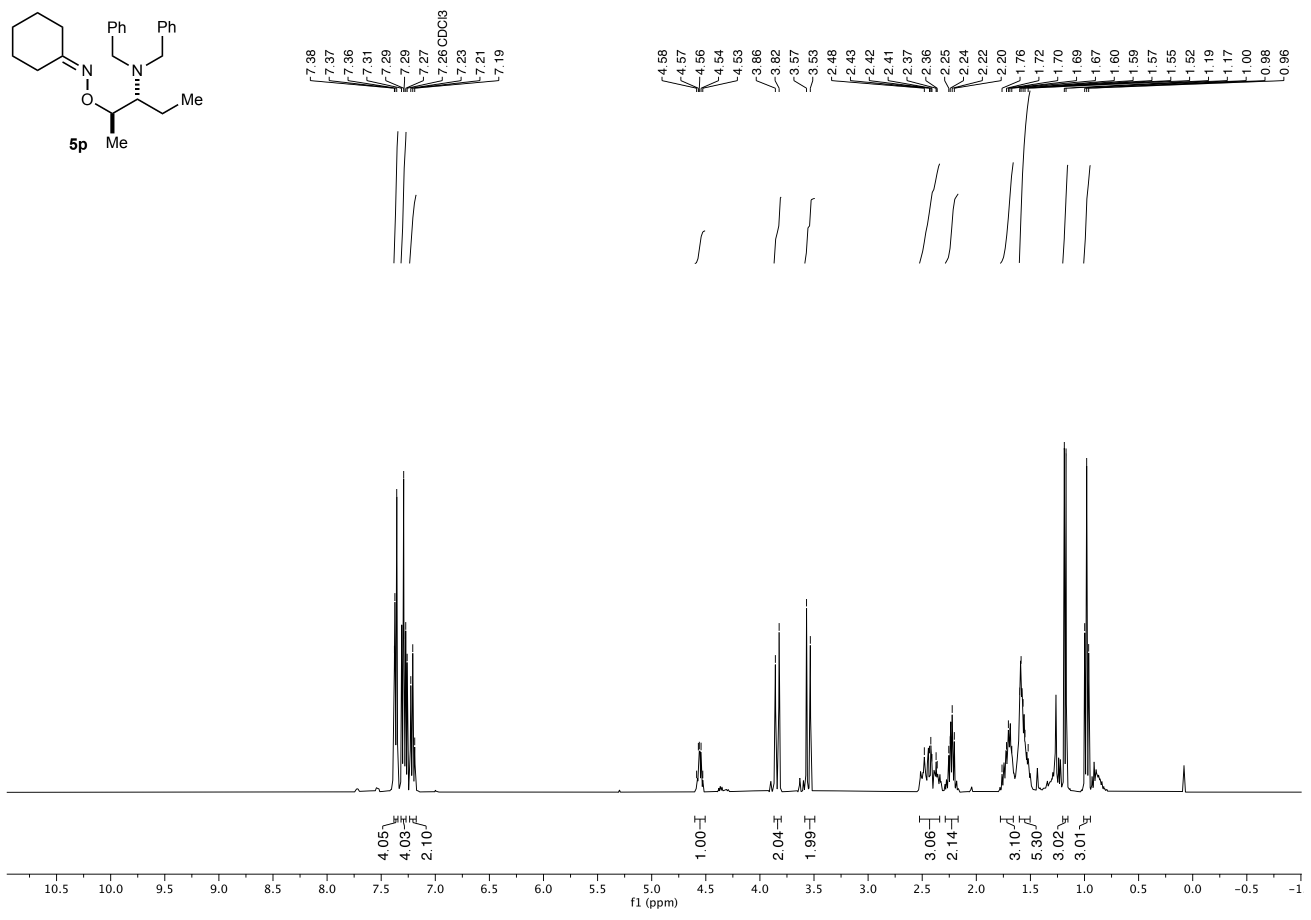

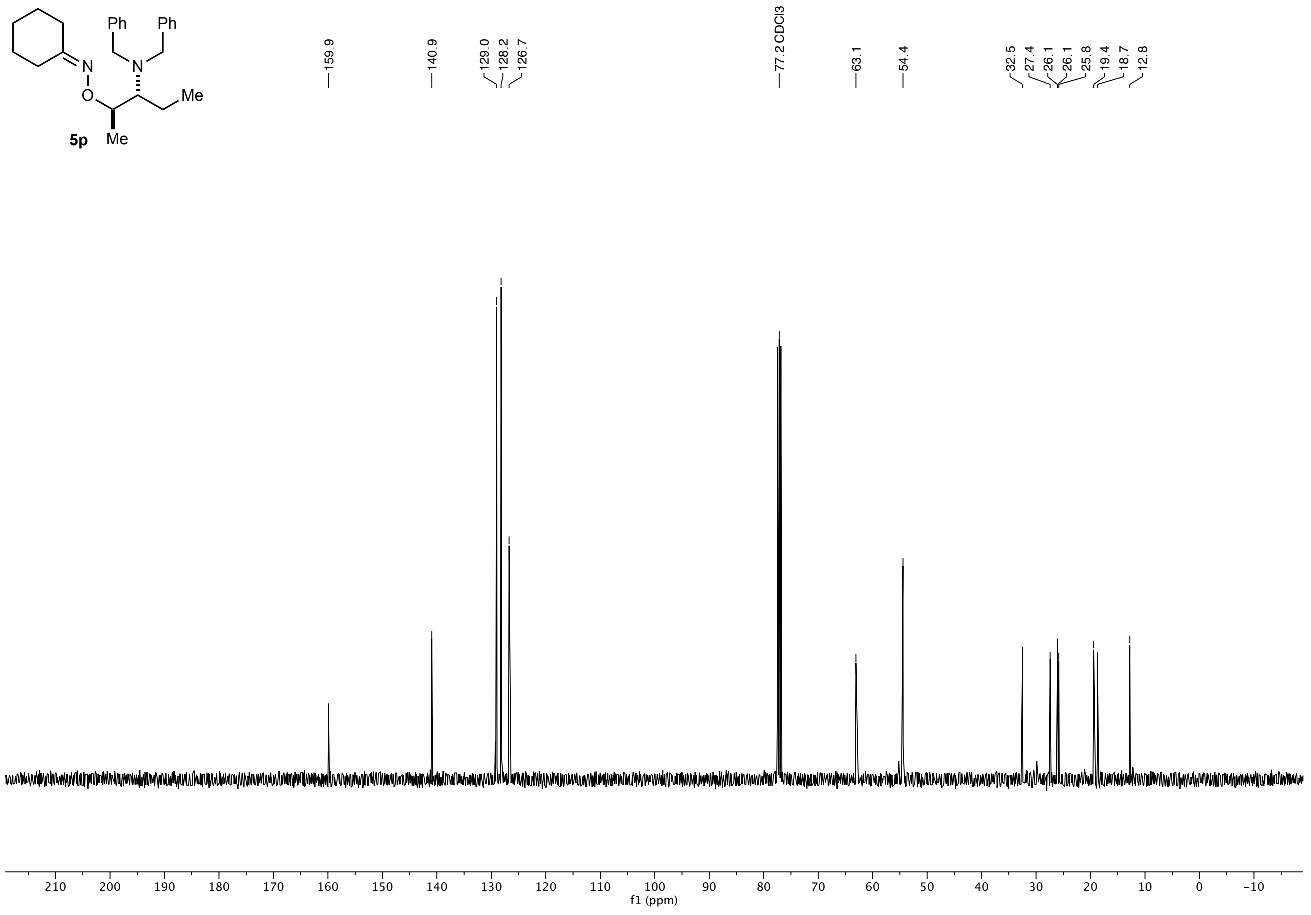

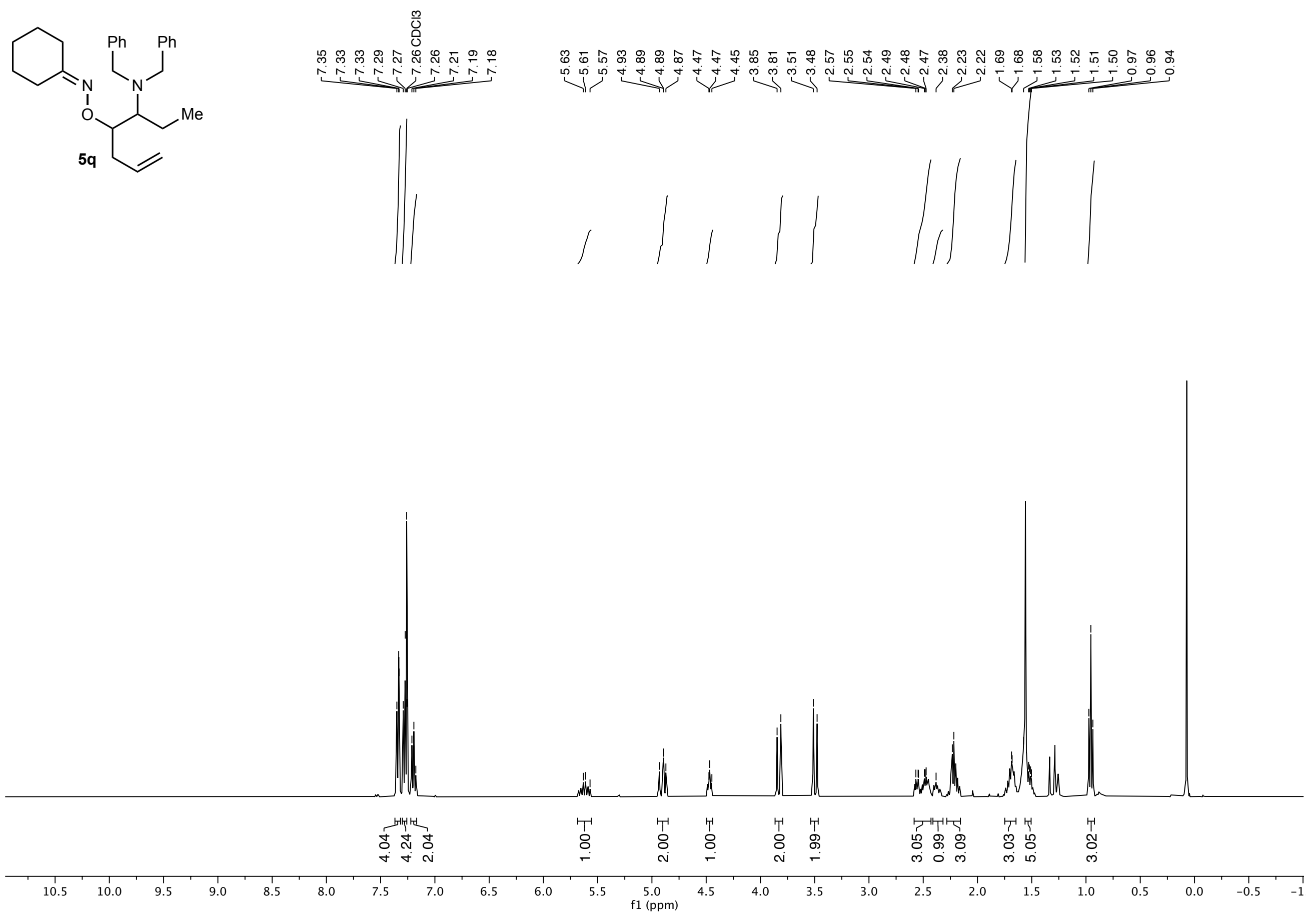
Ph
Ph
N
N
O
Me
5q
7.35 7.33 7.33 7.29 7.27 7.26 CDCl3 7.26 7.21 7.19 7.18
5.63 5.61 5.57 4.93 4.89 4.89 4.87 4.47 4.47 4.45 3.85 3.81 3.51 3.48 2.57 2.55 2.54 2.49 2.48 2.47 2.38 2.23 2.22 1.69 1.68 1.58 1.53 1.52 1.51 1.50 0.97 0.96 0.94
4.04 4.24 2.04 1.00 2.00 1.00 2.00 1.99 3.05 0.99 3.09 3.03 5.05 3.02
f1 (ppm)

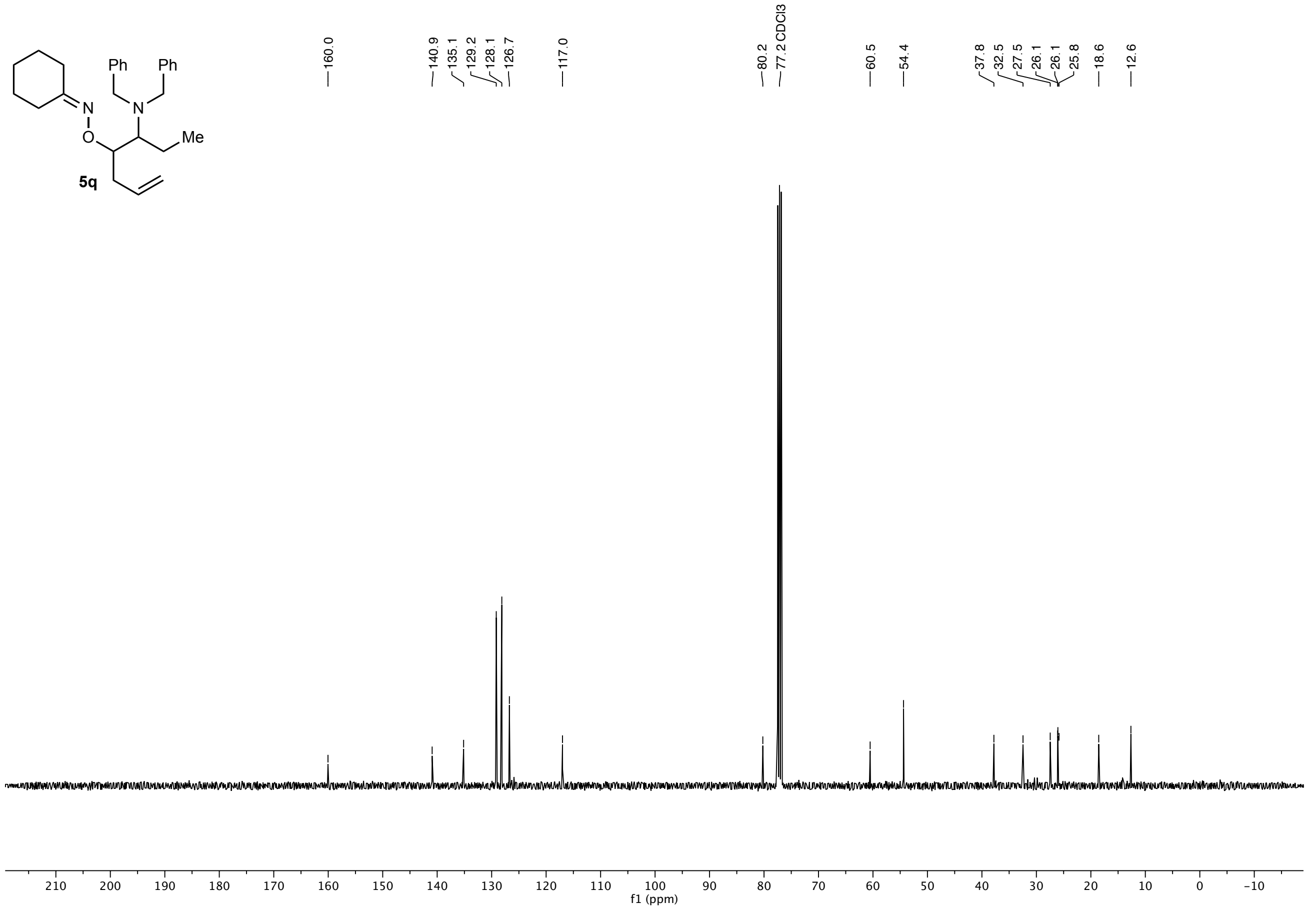
Ph
Ph
N
N
O
Me
5q
160.0 140.9 135.1 129.2 128.1 126.7 117.0 80.2 77.2 CDCl3 60.5 54.4 37.8 32.5 27.5 26.1 26.1 25.8 18.6 12.6
f1 (ppm)

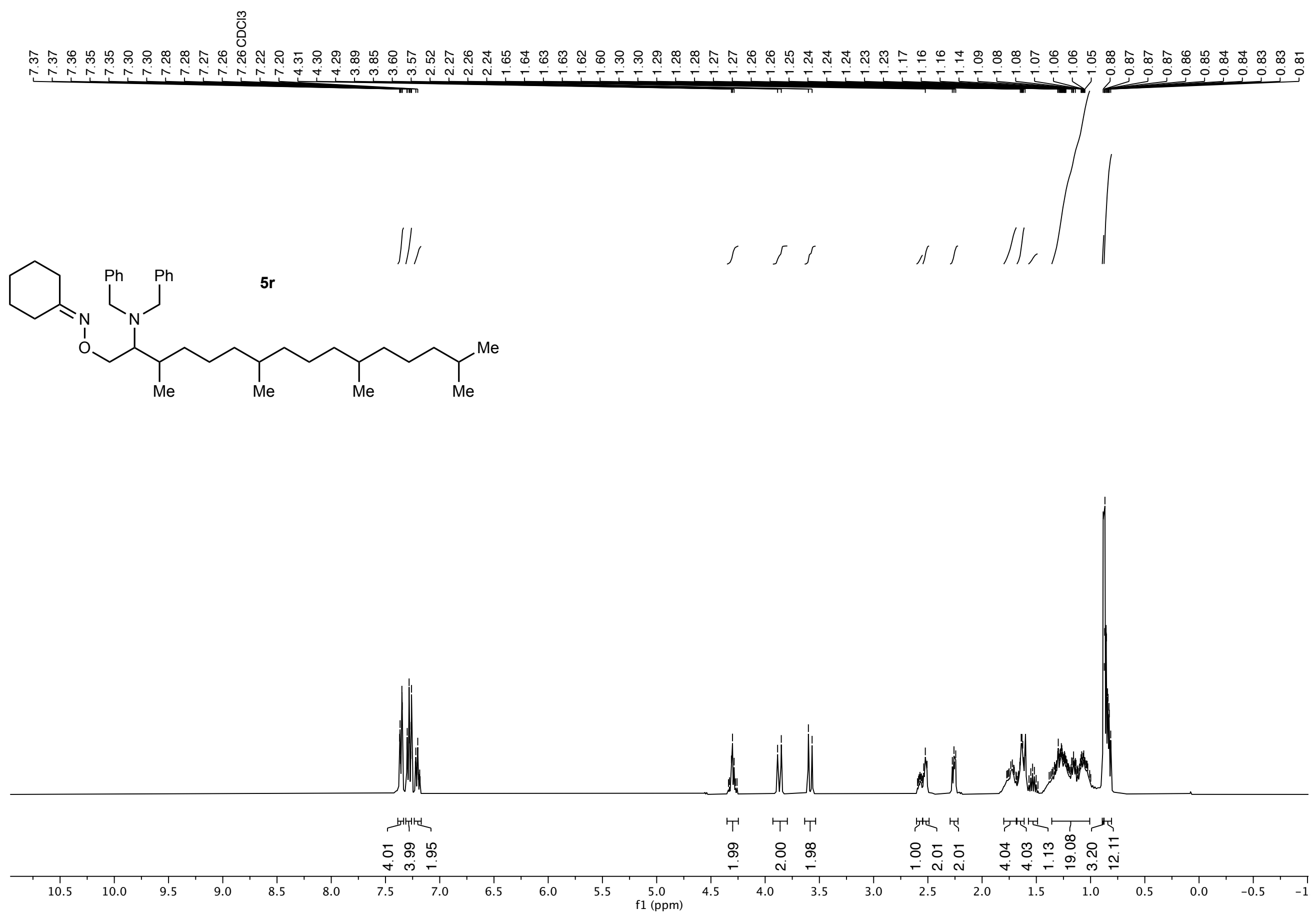
7.37 7.37 7.36 7.35 7.35 7.30 7.30 7.28 7.28 7.27 7.26 7.26 CDCl3 7.22 7.20 4.31 4.30 4.29 3.89 3.85 3.60 3.57 2.52 2.27 2.26 2.24 1.65 1.64 1.63 1.63 1.62 1.60 1.30 1.30 1.29 1.28 1.28 1.27 1.27 1.26 1.26 1.25 1.24 1.24 1.24 1.23 1.23 1.17 1.16 1.16 1.14 1.09 1.08 1.08 1.07 1.06 1.06 1.05 0.88 0.87 0.87 0.87 0.86 0.85 0.84 0.84 0.83 0.83 0.81
Ph
Ph
N
N
O
Me
Me
Me
Me
Me
5r
4.01
3.99
1.95
1.99
2.00
1.98
1.00
2.01
2.01
4.04
4.03
1.13
19.08
3.20
12.11
f1 (ppm)

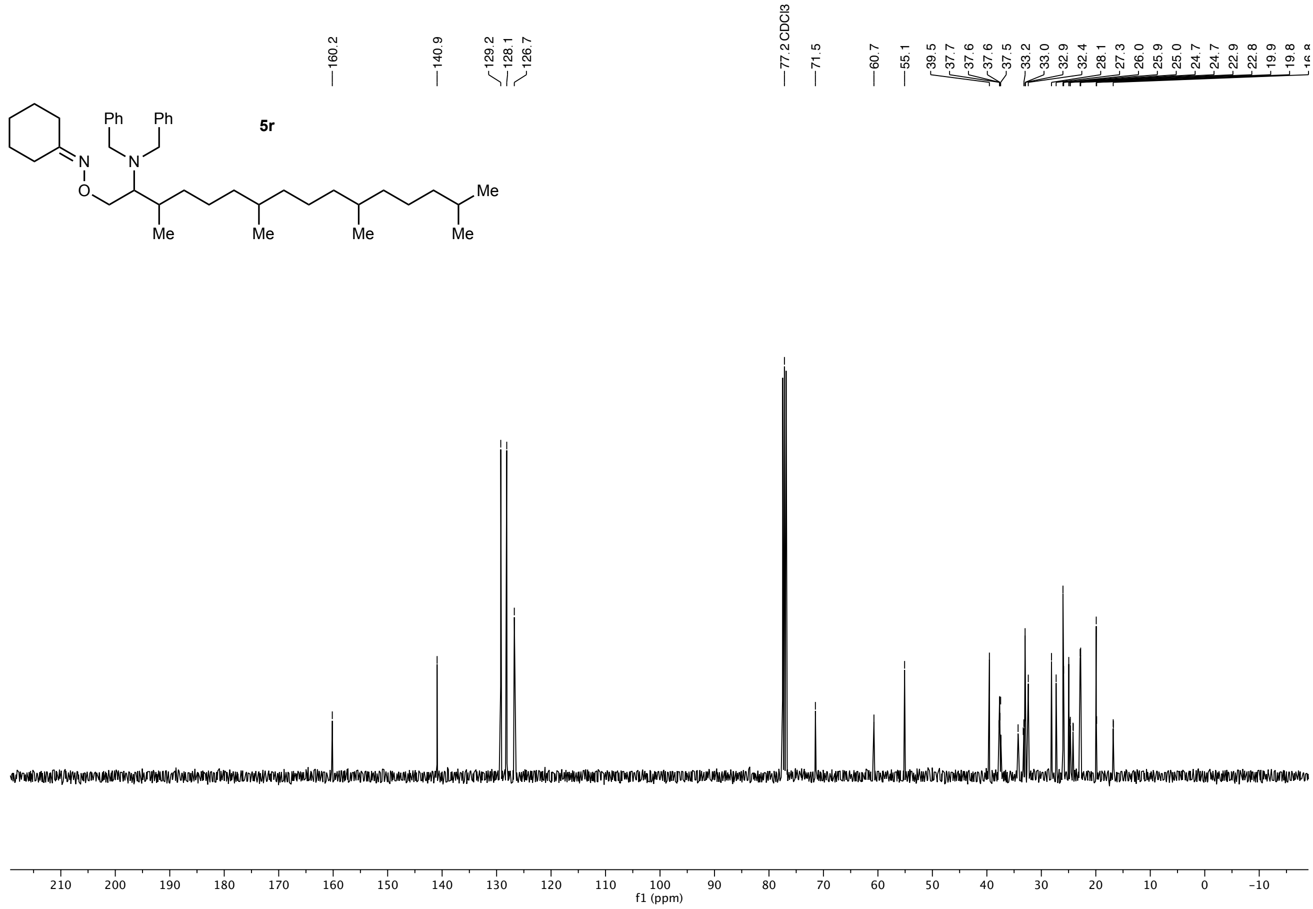
160.2
140.9
129.2
128.1
126.7
77.2 CDCl3
71.5
60.7
55.1
39.5 37.7 37.6 37.6 37.5 33.2 33.0 32.9 32.4 28.1 27.3 26.0 25.9 25.0 24.7 24.7 22.9 22.8 19.9 19.8
Ph
Ph
N
N
O
Me
Me
Me
Me
Me
5r
f1 (ppm)

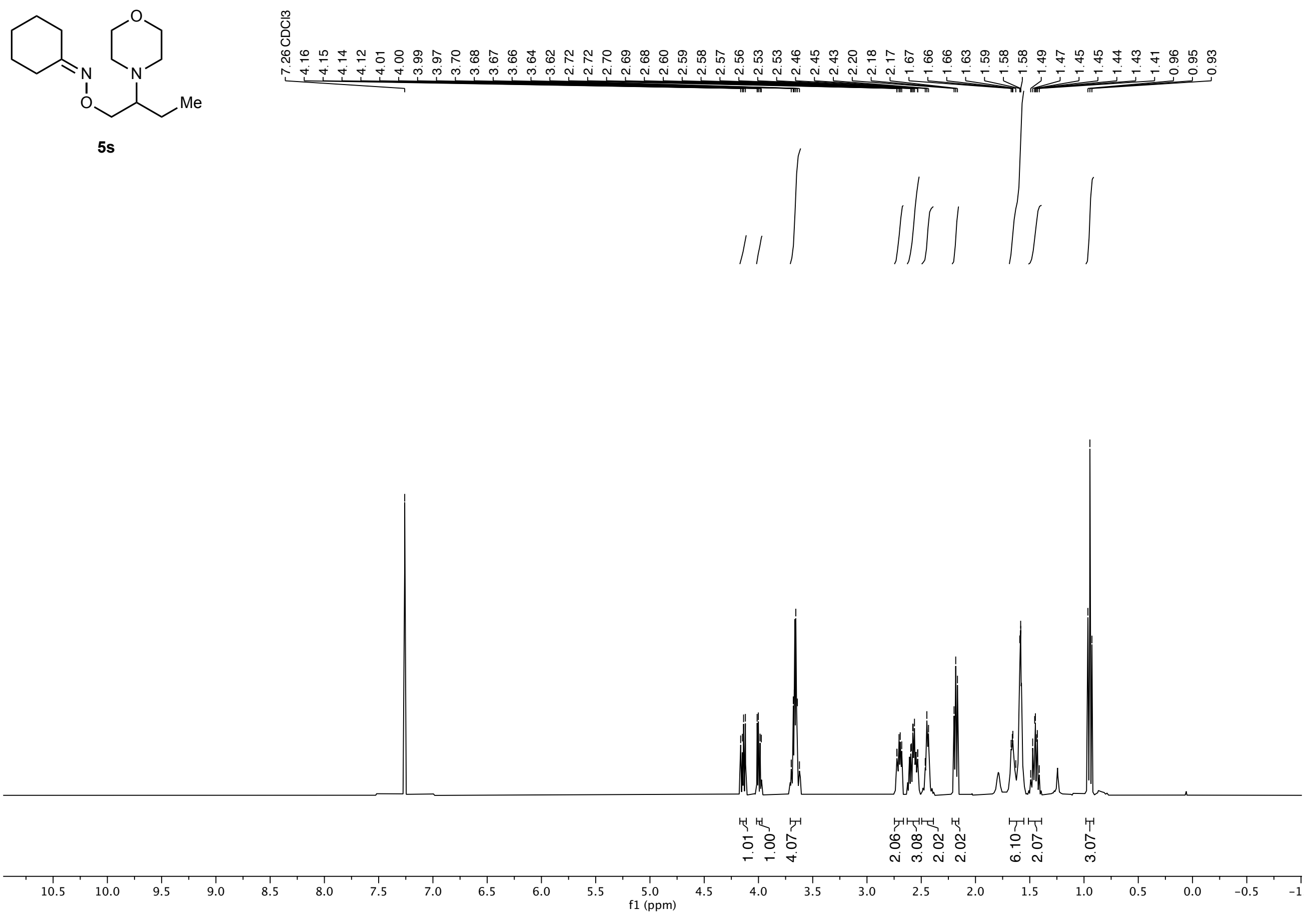
O
N
N
O
Me
7.26 CDCl3
4.16
4.15
4.14
4.12
4.01
4.00
3.99
3.97
3.70
3.68
3.67
3.66
3.64
3.62
2.72
2.72
2.70
2.69
2.68
2.60
2.59
2.58
2.57
2.56
2.53
2.53
2.46
2.45
2.43
2.20
2.18
2.17
1.67
1.66
1.66
1.63
1.59
1.58
1.58
1.49
1.47
1.45
1.45
1.44
1.43
1.41
0.96
0.95
0.93
1.01
1.00
4.07
2.06
3.08
2.02
2.02
6.10
2.07
3.07
10.5
10.0
9.5
9.0
8.5
8.0
7.5
7.0
6.5
6.0
5.5
5.0
4.5
4.0
3.5
3.0
2.5
2.0
1.5
1.0
0.5
0.0
−0.5
−1
f1 (ppm)

5s

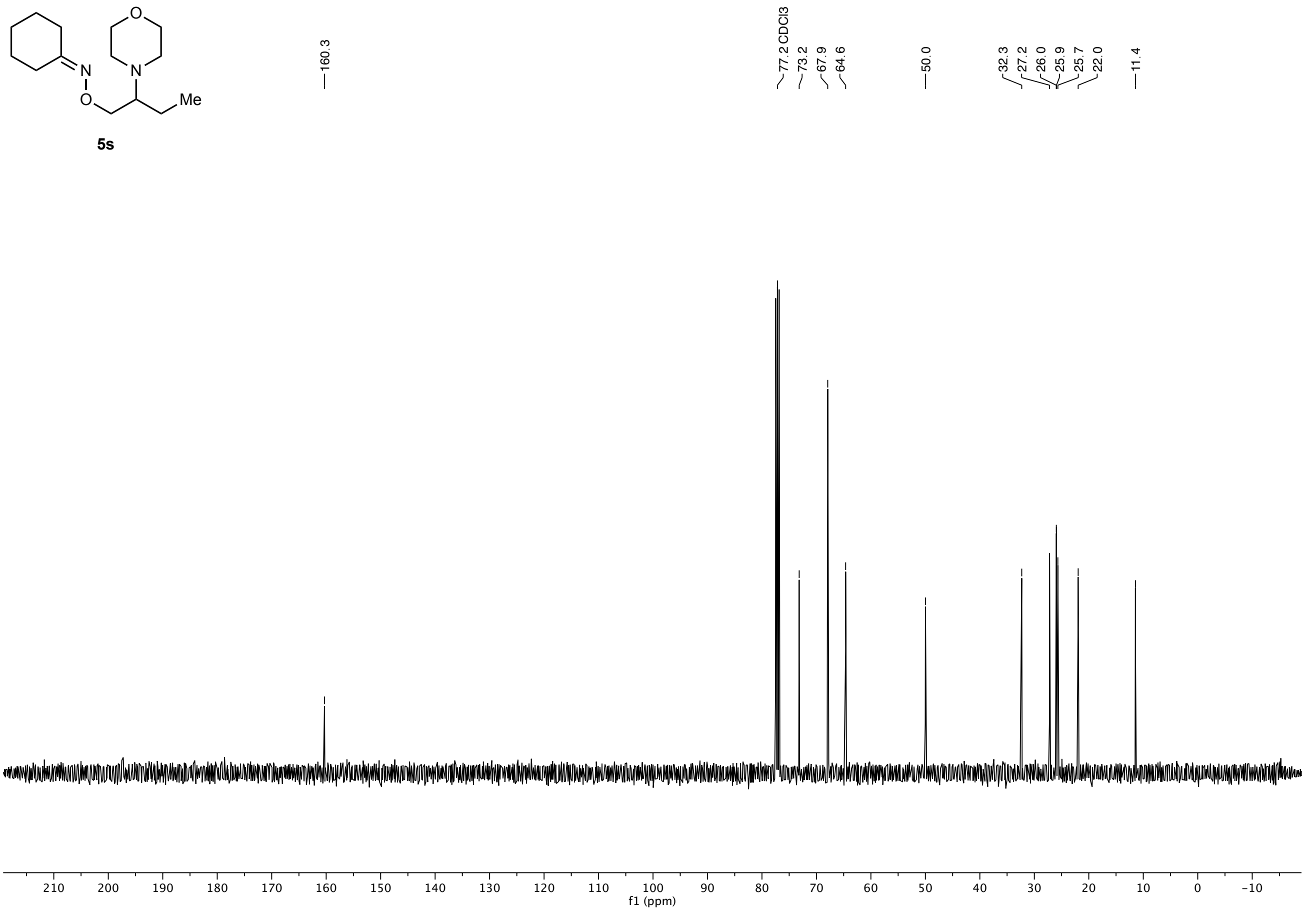
O
N
N
O
Me
160.3
77.2 CDCl3
73.2
67.9
64.6
50.0
32.3
27.2
26.0
25.9
25.7
22.0
11.4
210
200
190
180
170
160
150
140
130
120
110
100
90
80
70
60
50
40
30
20
10
0
−10
f1 (ppm)

5s

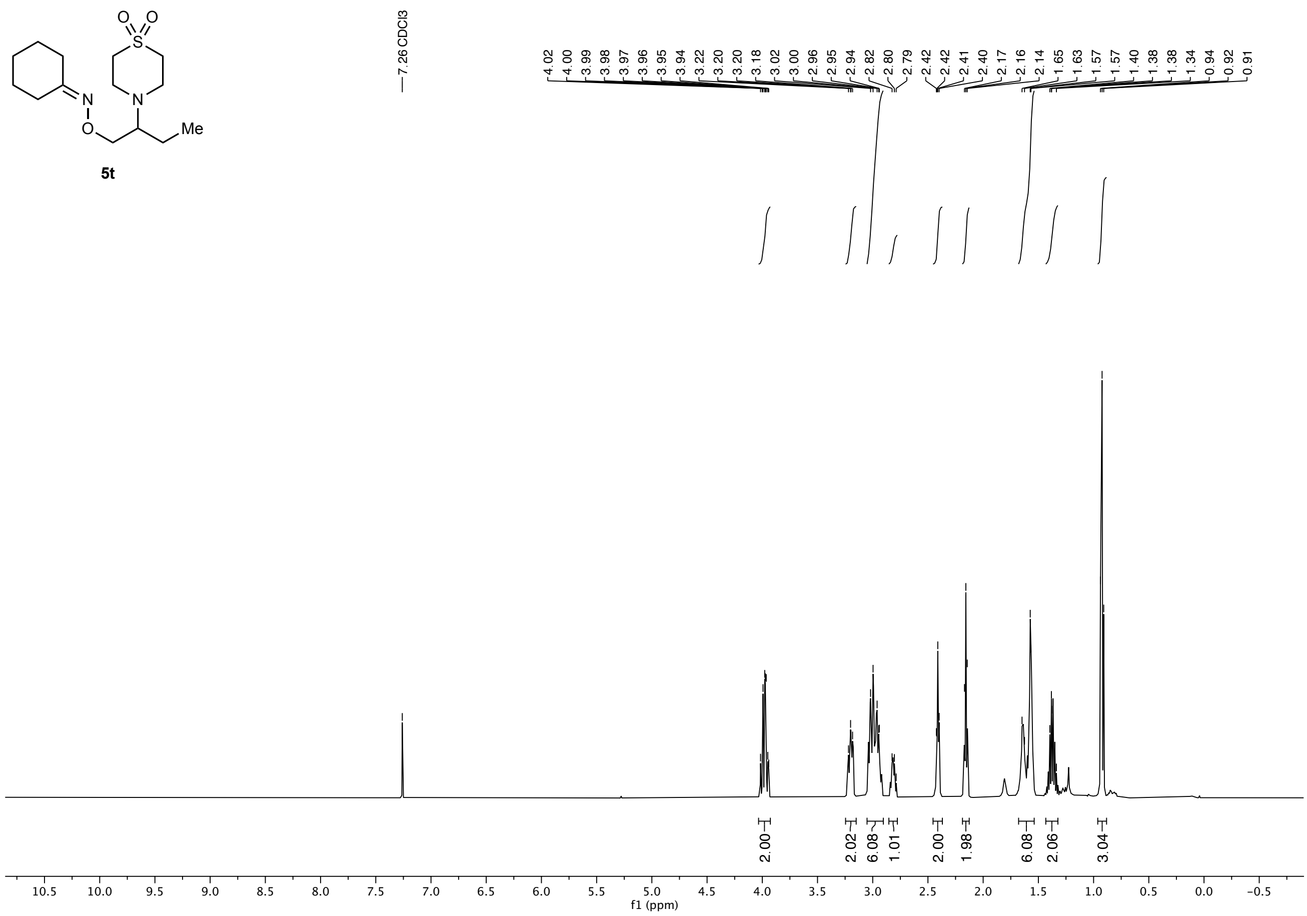
5t
Me
7.26 CDCl3
4.02 4.00 3.99 3.98 3.97 3.96 3.95 3.94 3.22 3.20 3.20 3.18 3.02 3.00 2.96 2.95 2.94 2.82 2.80 2.79 2.42 2.42 2.41 2.40 2.17 2.16 2.14 1.65 1.63 1.57 1.57 1.40 1.38 1.38 1.34 0.94 0.92 0.91
2.00 2.02 6.08 1.01 2.00 1.98 6.08 2.06 3.04
f1 (ppm)

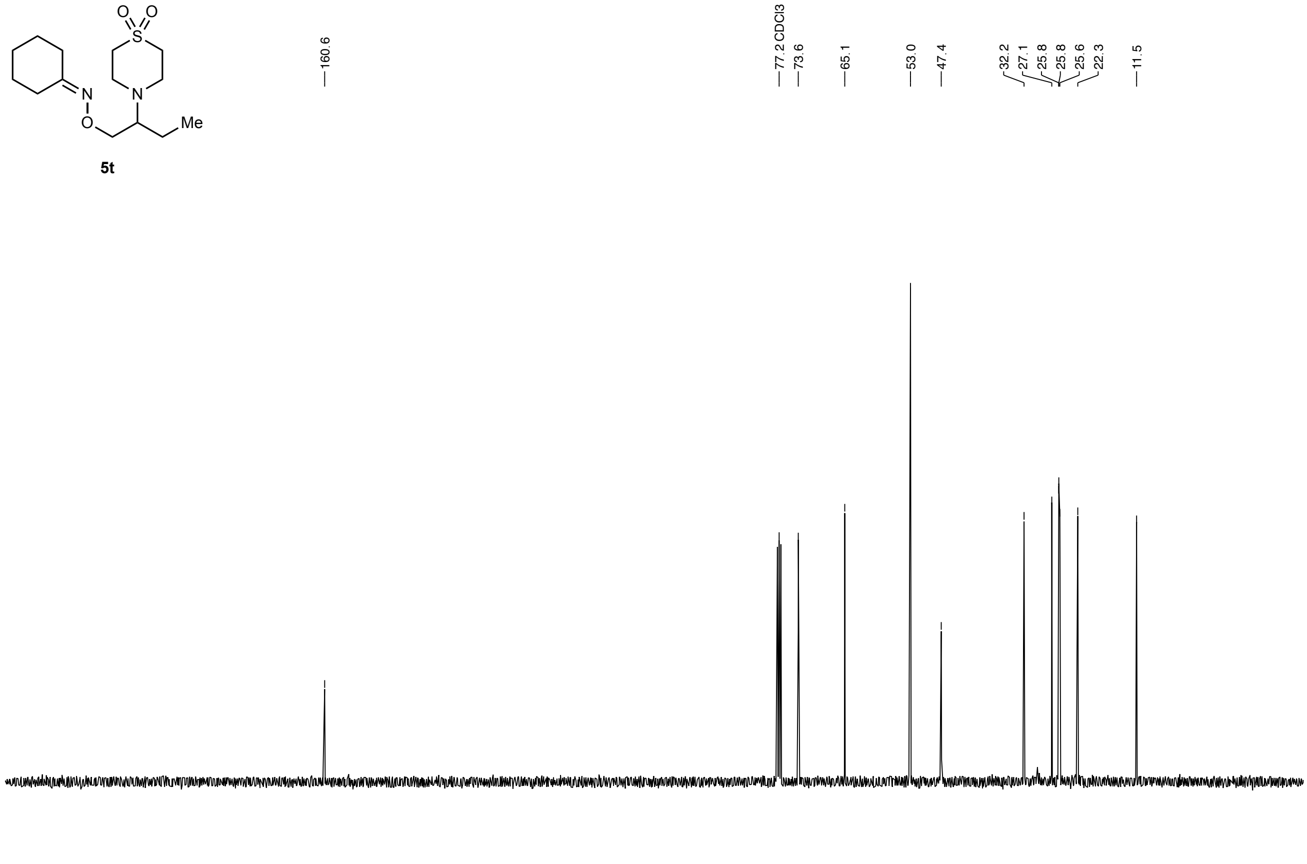
5t
Me
160.6
77.2 CDCl3
73.6
65.1
53.0
47.4
32.2 27.1 25.8 25.8 25.6 22.3
11.5
f1 (ppm)

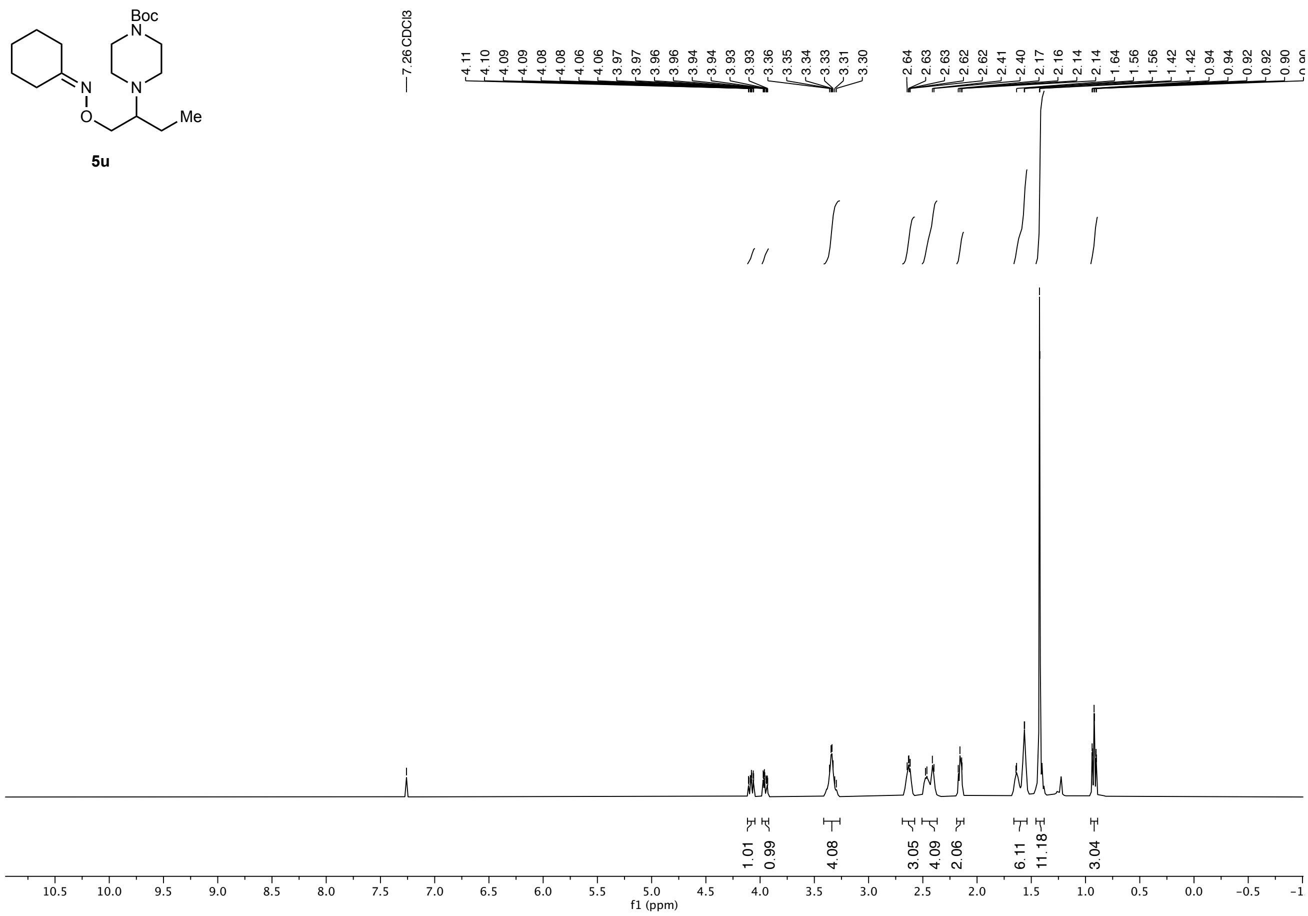
Boc
N
N
N
O
Me
5u
7.26 CDCl3
4.11 4.10 4.09 4.09 4.08 4.08 4.06 4.06 3.97 3.97 3.96 3.96 3.94 3.94 3.93 3.93 3.36 3.35 3.34 3.33 3.31 3.30
2.64 2.63 2.63 2.62 2.62 2.41 2.40 2.17 2.16 2.14 2.14 1.64 1.56 1.56 1.42 1.42 0.94 0.94 0.92 0.92 0.90
1.01 0.99 4.08 3.05 4.09 2.06 6.11 11.18 3.04
f1 (ppm)

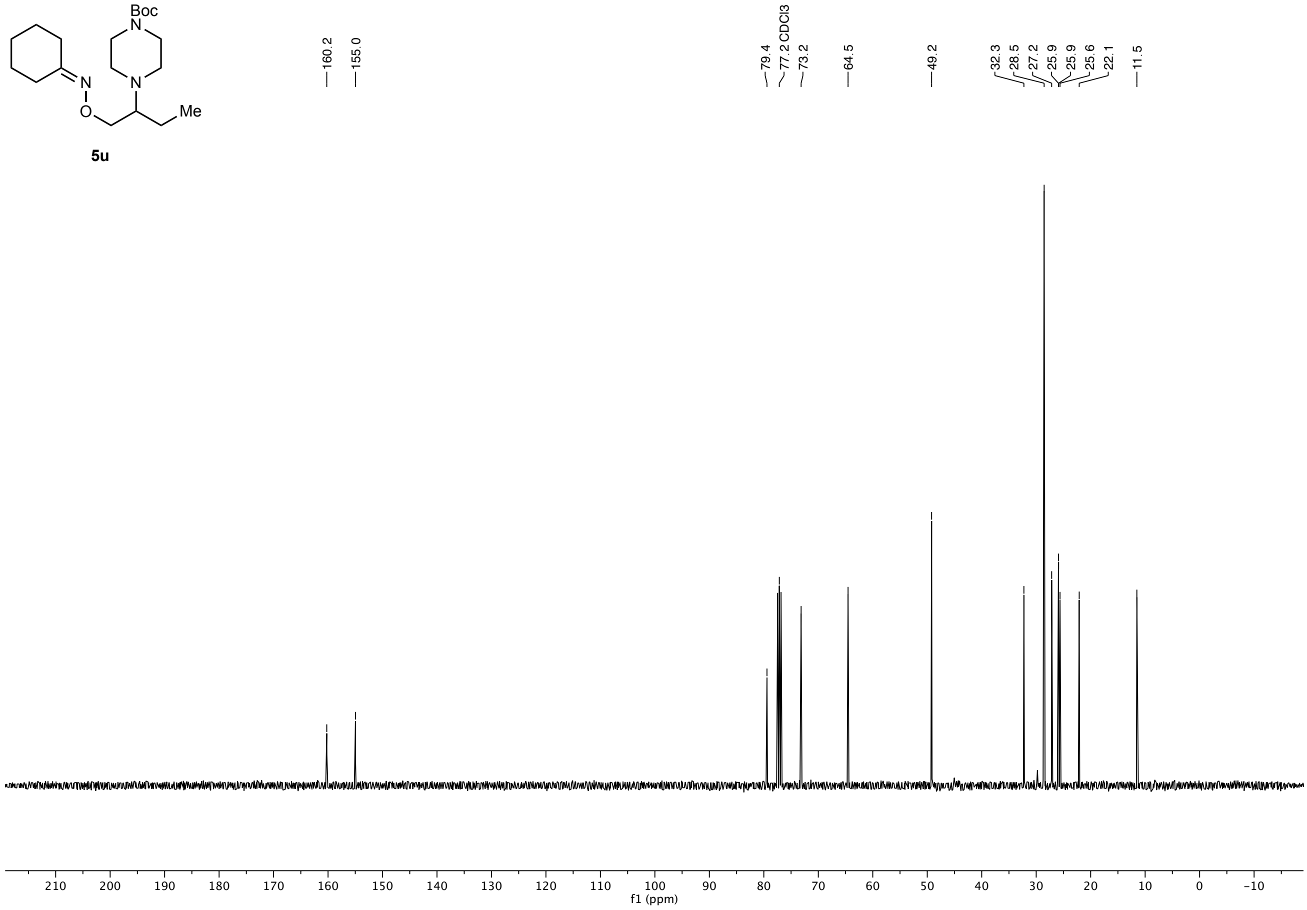
Boc
N
N
N
O
Me
5u
160.2
155.0
79.4
77.2 CDCl3
73.2
64.5
49.2
32.3 28.5 27.2 25.9 25.9 25.6 22.1
11.5
f1 (ppm)

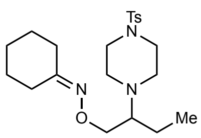

5v

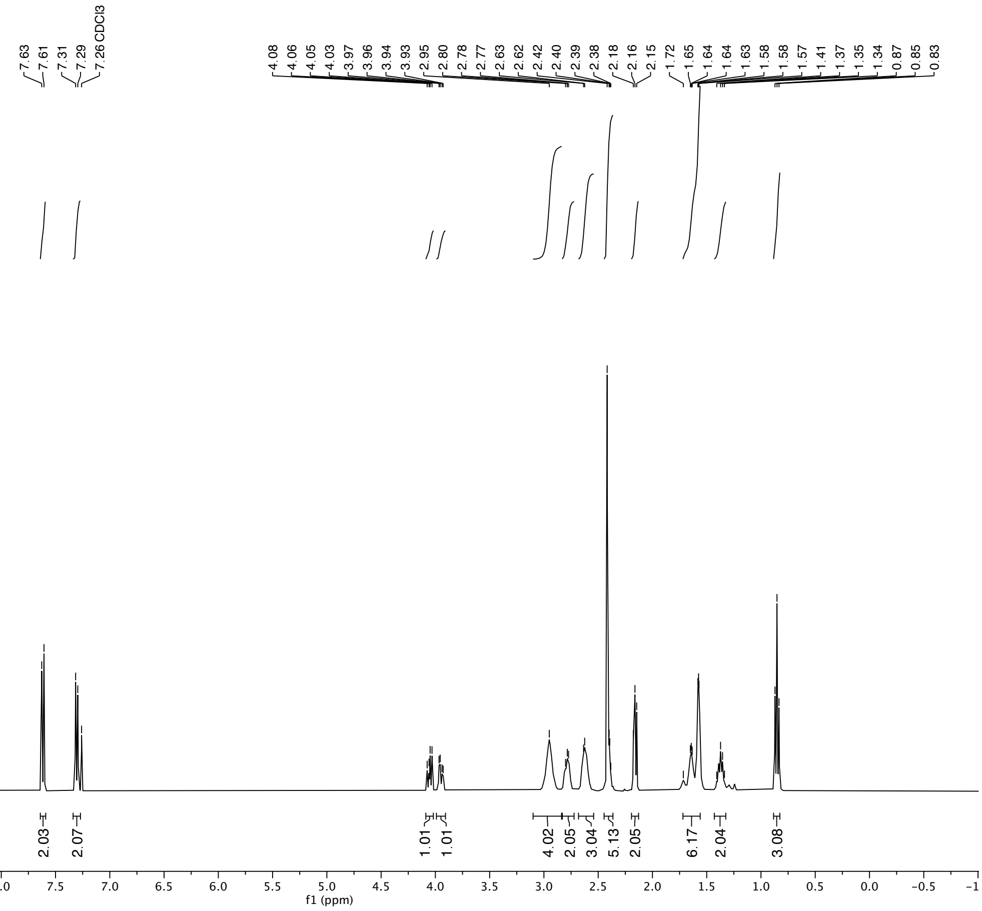
7.63
7.61
7.31
7.29
7.26 CDCl3
4.08
4.06
4.05
4.03
3.97
3.96
3.94
3.93
2.95
2.80
2.78
2.77
2.63
2.62
2.42
2.40
2.39
2.38
2.18
2.16
2.15
1.72
1.65
1.64
1.64
1.63
1.58
1.58
1.57
1.41
1.37
1.35
1.34
0.87
0.85
0.83
2.03
2.07
1.01
1.01
4.02
2.05
3.04
5.13
2.05
6.17
2.04
3.08
f1 (ppm)

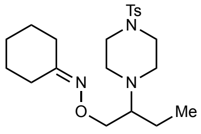

5v

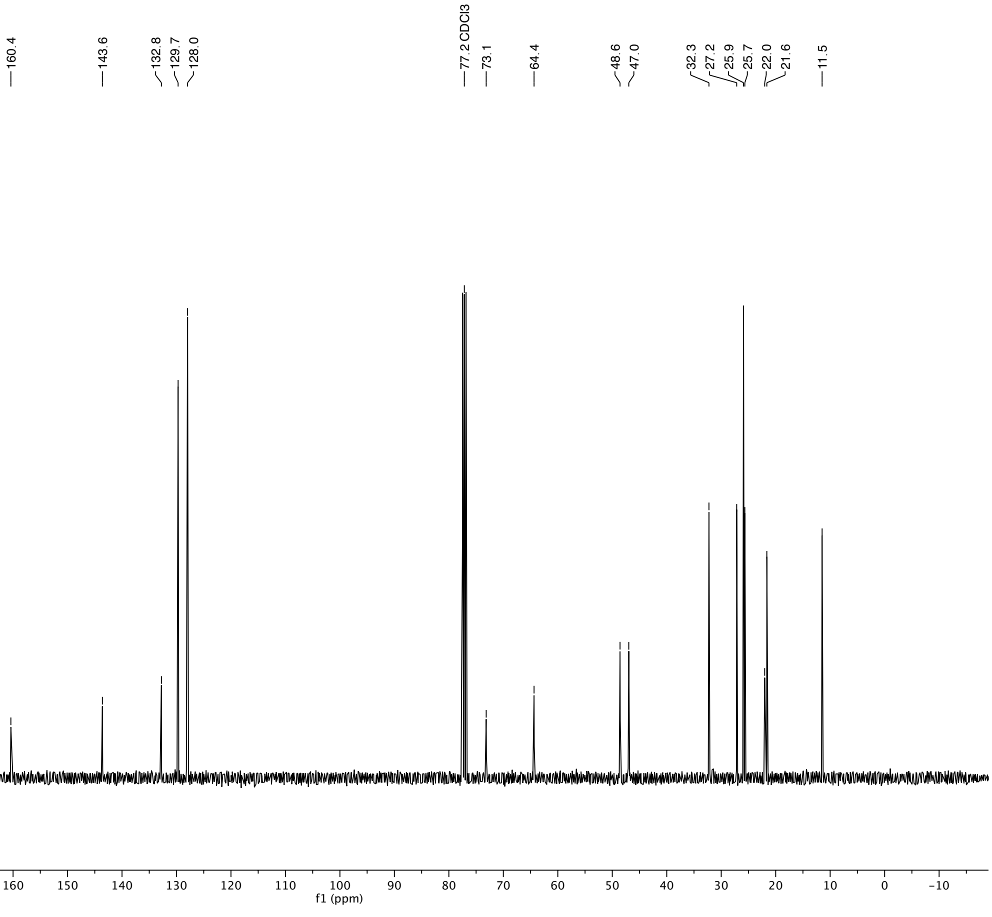
160.4
143.6
132.8
129.7
128.0
77.2 CDCl3
73.1
64.4
48.6
47.0
32.3
27.2
25.9
25.7
22.0
21.6
11.5
f1 (ppm)

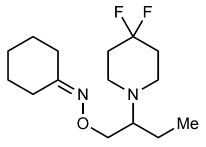
F F
N
O
N
Me

5w

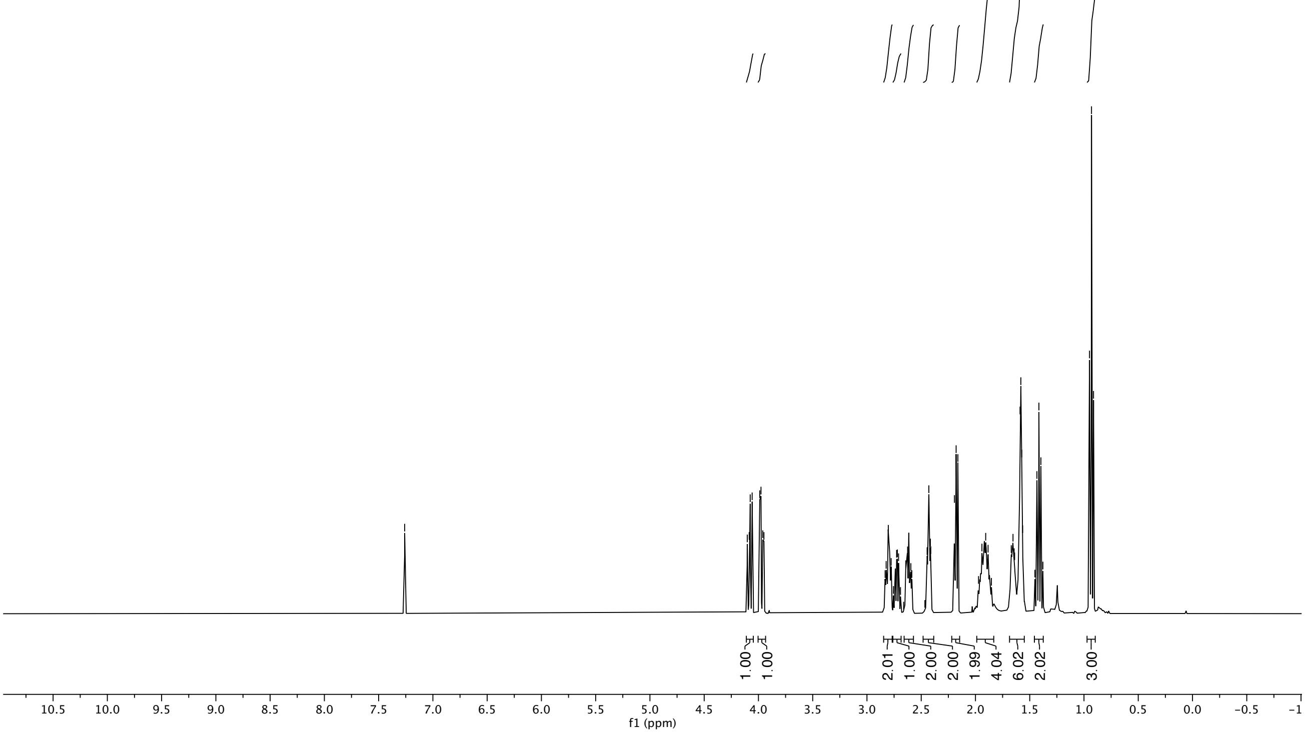
7.26 CDCl3
4.10 4.09 4.08 4.06 3.99 3.98 3.96 3.95 2.83 2.82 2.80 2.78 2.76 2.73 2.72 2.71 2.69 2.66 2.64 2.63 2.60 2.59 2.47 2.45 2.43 2.42 2.41 2.19 2.18 2.16 1.97 1.94 1.91 1.88 1.85 1.67 1.65 1.64 1.64 1.59 1.58 1.57 1.57 1.45 1.43 1.42 1.40 1.38 0.95 0.93 0.91
1.00 1.00 2.01 1.00 2.00 2.00 1.99 4.04 6.02 2.02 3.00
f1 (ppm)

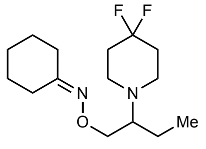
F F
N
O
N
Me

5w

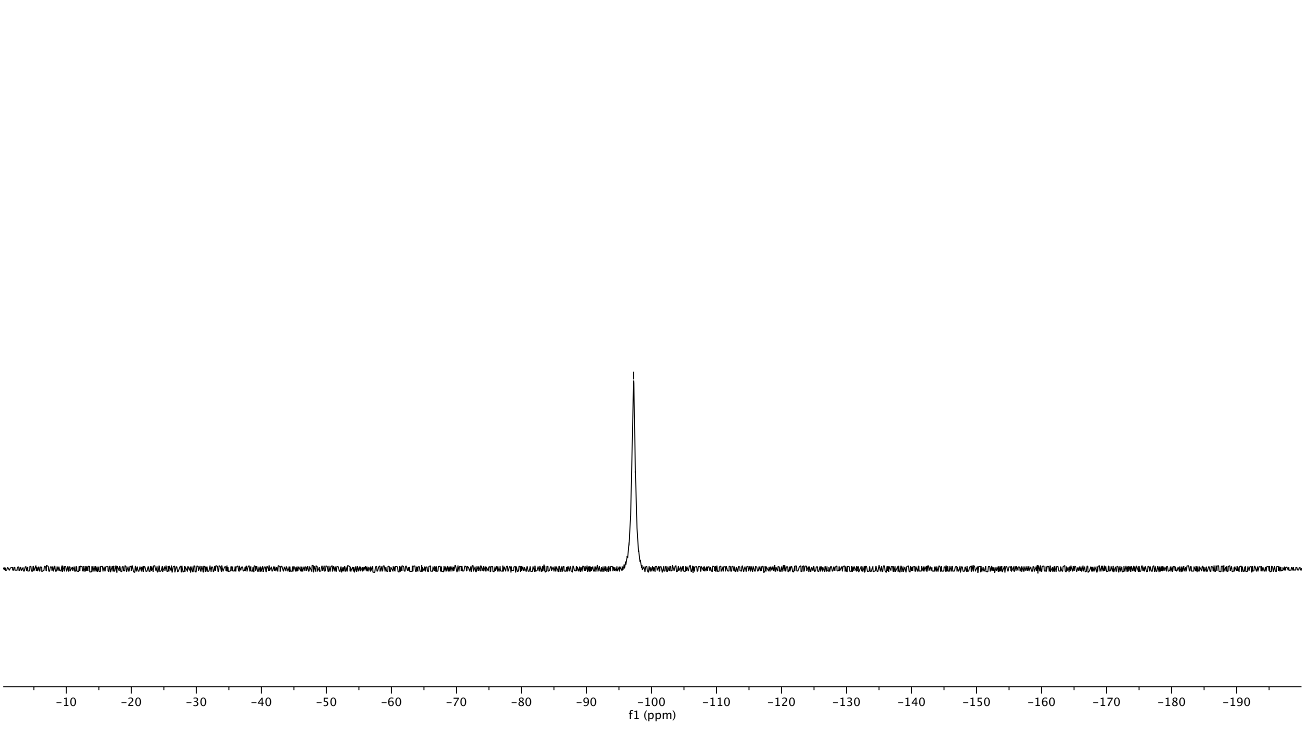
-97.3
f1 (ppm)

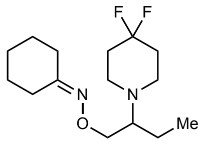

5w

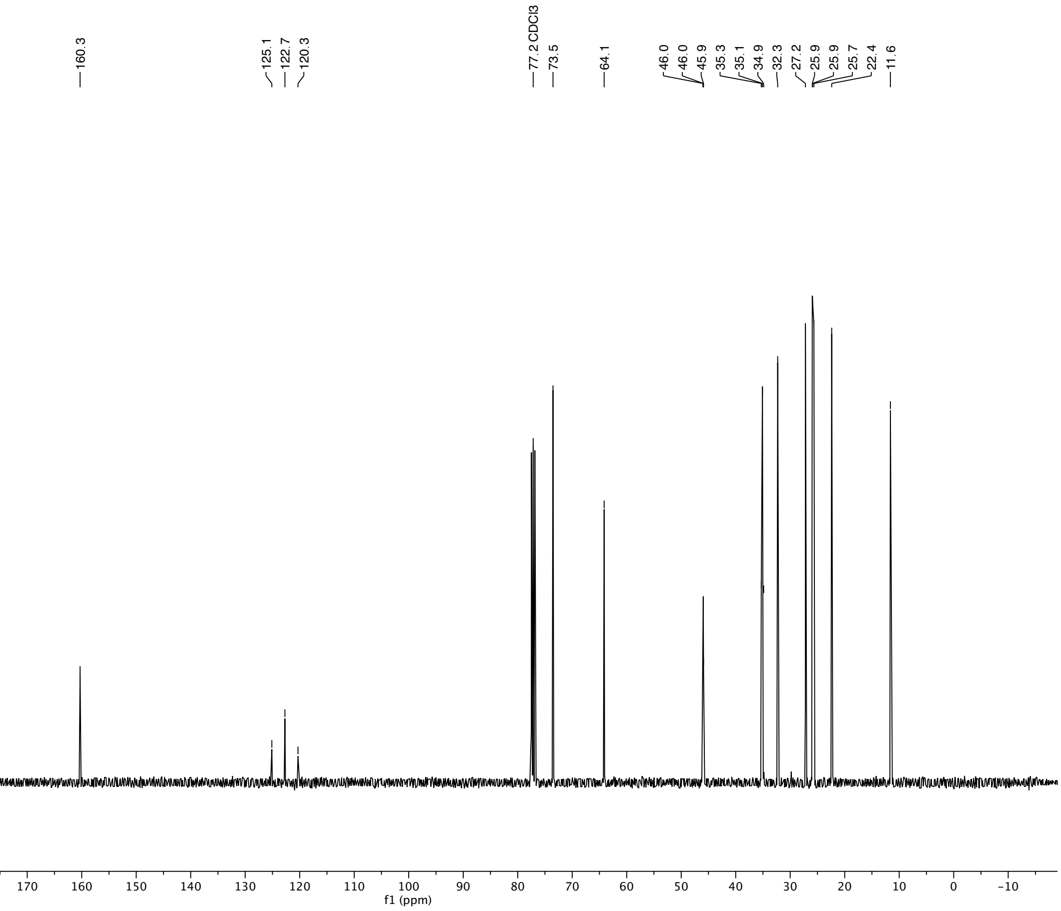

160.3
125.1
122.7
120.3
77.2 CDCl3
73.5
64.1
46.0
46.0
45.9
35.3
35.1
34.9
32.3
27.2
25.9
25.9
25.7
22.4
11.6
f1 (ppm)

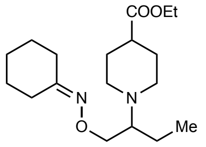
COOEt
N
N
O
Me

5x

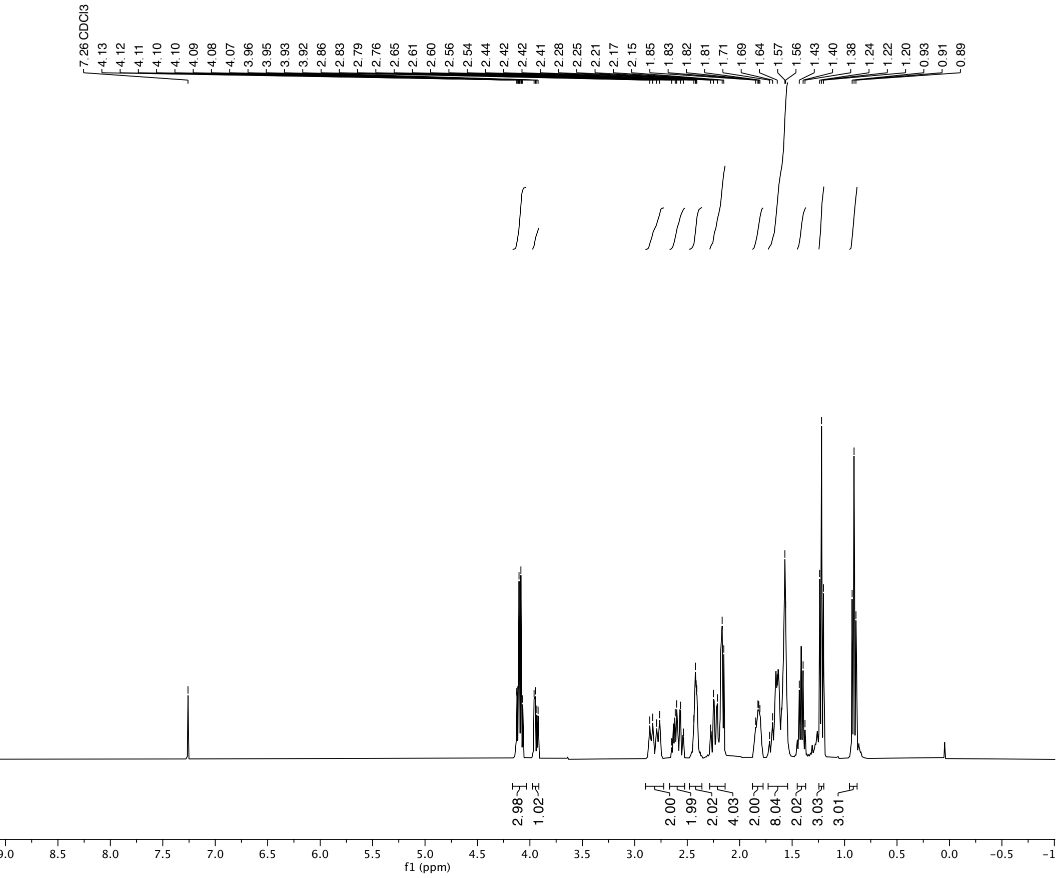
7.26 CDCl3
4.13
4.12
4.11
4.10
4.10
4.09
4.08
4.07
3.96
3.95
3.93
3.92
2.86
2.83
2.79
2.76
2.65
2.61
2.60
2.56
2.54
2.44
2.42
2.42
2.41
2.28
2.25
2.21
2.17
2.15
1.85
1.83
1.82
1.81
1.71
1.69
1.64
1.57
1.56
1.43
1.40
1.38
1.24
1.22
1.20
0.93
0.91
0.89
2.98
1.02
2.00
1.99
2.02
4.03
2.00
8.04
2.02
3.03
3.01
f1 (ppm)

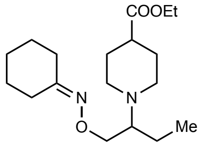
COOEt
N
N
O
Me

5x

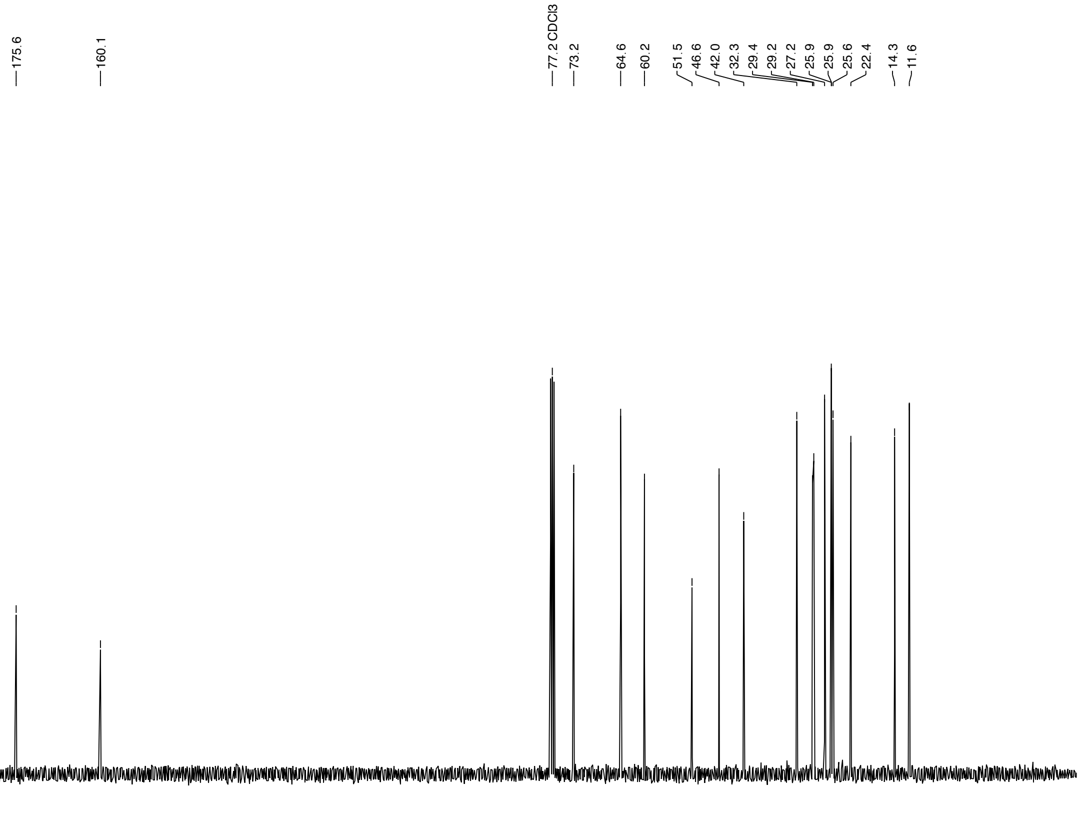
175.6
160.1
77.2 CDCl3
73.2
64.6
60.2
51.5
46.6
42.0
32.3
29.4
29.2
27.2
25.9
25.9
25.6
22.4
14.3
11.6
f1 (ppm)

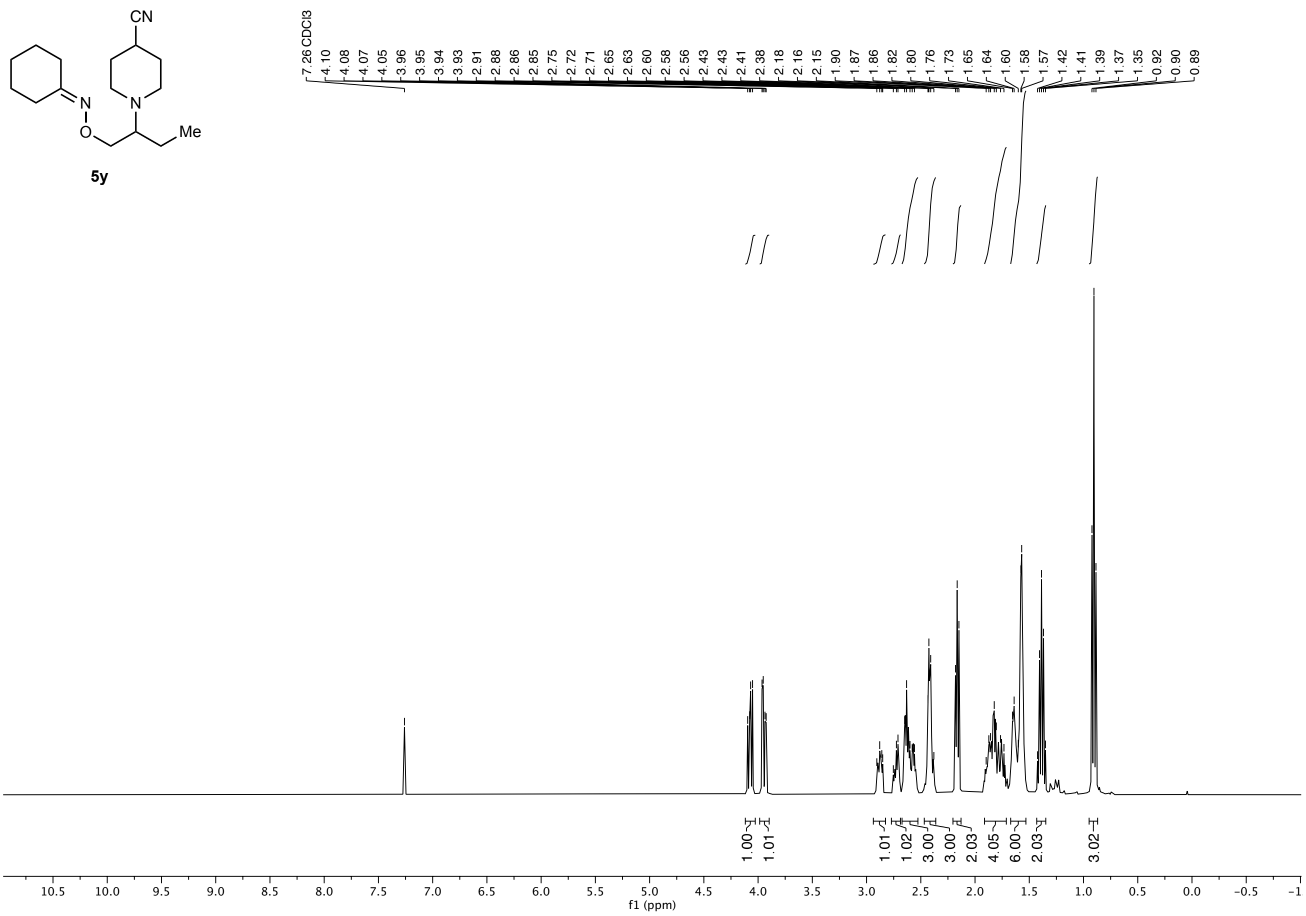
CN
N
N
O
Me
5y
7.26 CDCl3
4.10
4.08
4.07
4.05
3.96
3.95
3.94
3.93
2.91
2.88
2.86
2.85
2.75
2.72
2.71
2.65
2.63
2.60
2.58
2.56
2.43
2.43
2.41
2.38
2.18
2.16
2.15
1.90
1.87
1.86
1.82
1.80
1.76
1.73
1.65
1.64
1.60
1.58
1.57
1.42
1.41
1.39
1.37
1.35
0.92
0.90
0.89
1.00
1.01
1.01
1.02
3.00
3.00
2.03
4.05
6.00
2.03
3.02
f1 (ppm)

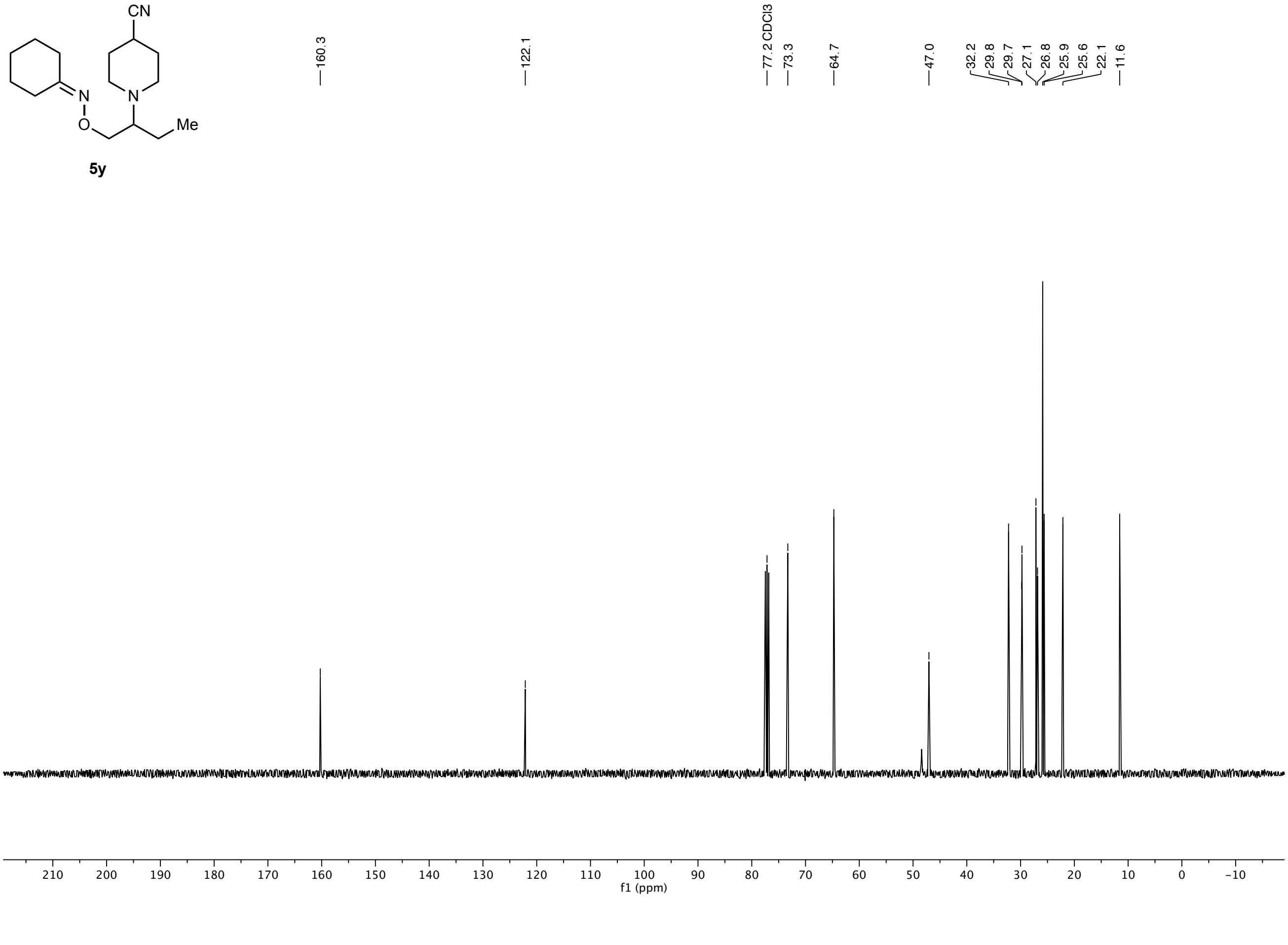
CN
N
N
O
Me
5y
160.3
122.1
77.2 CDCl3
73.3
64.7
47.0
32.2
29.8
29.7
27.1
26.8
25.9
25.6
22.1
11.6
f1 (ppm)

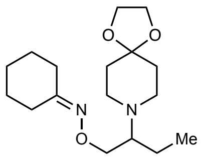
O
O
N
N
O
Me

5z

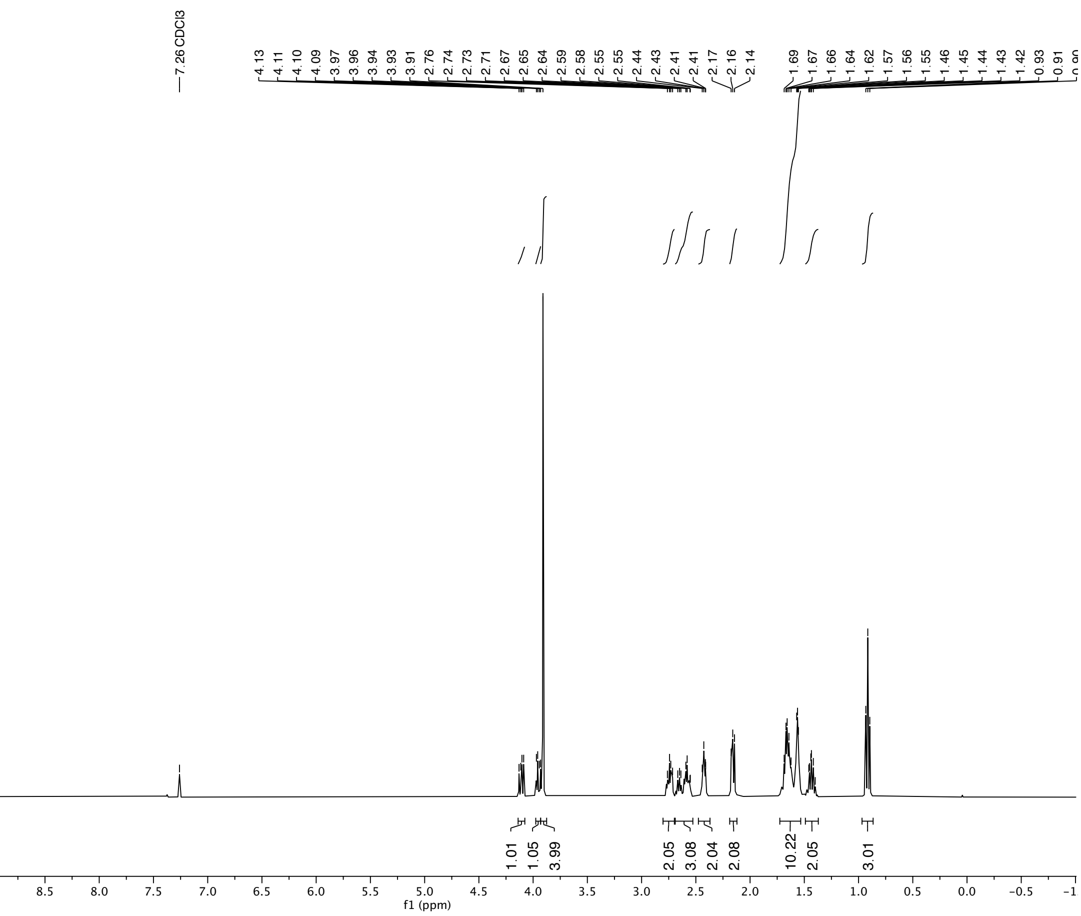
7.26 CDCl3
4.13 4.11 4.10 4.09 3.97 3.96 3.94 3.93 3.91 2.76 2.74 2.73 2.71 2.67 2.65 2.64 2.59 2.58 2.55 2.55 2.44 2.43 2.41 2.41 2.17 2.16 2.14
1.69 1.67 1.66 1.64 1.62 1.57 1.56 1.55 1.46 1.45 1.44 1.43 1.42 0.93 0.91
1.01 1.05 3.99 2.05 3.08 2.04 2.08 10.22 2.05 3.01
f1 (ppm)

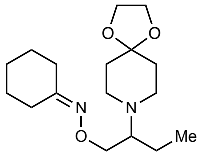
O
O
N
N
O
Me

5z

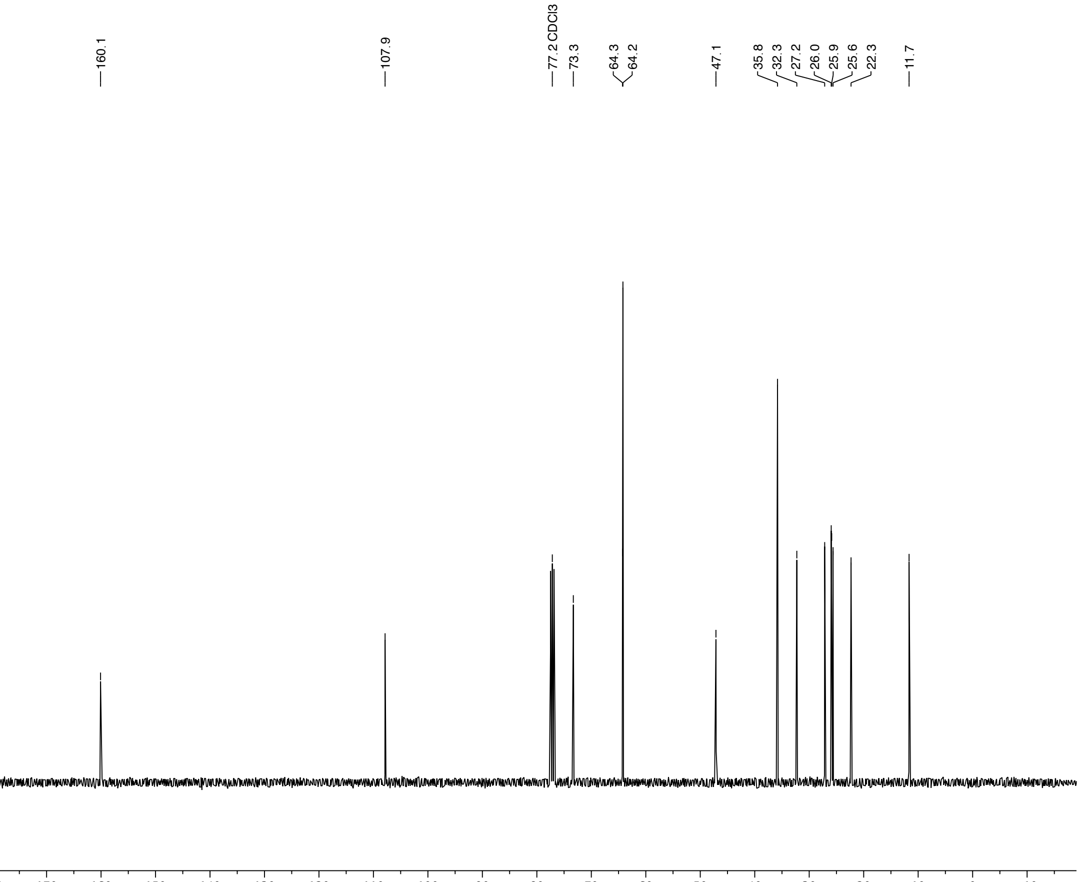
160.1
107.9
77.2 CDCl3
73.3
64.3 64.2
47.1
35.8 32.3 27.2 26.0 25.9 25.6 22.3
11.7
f1 (ppm)

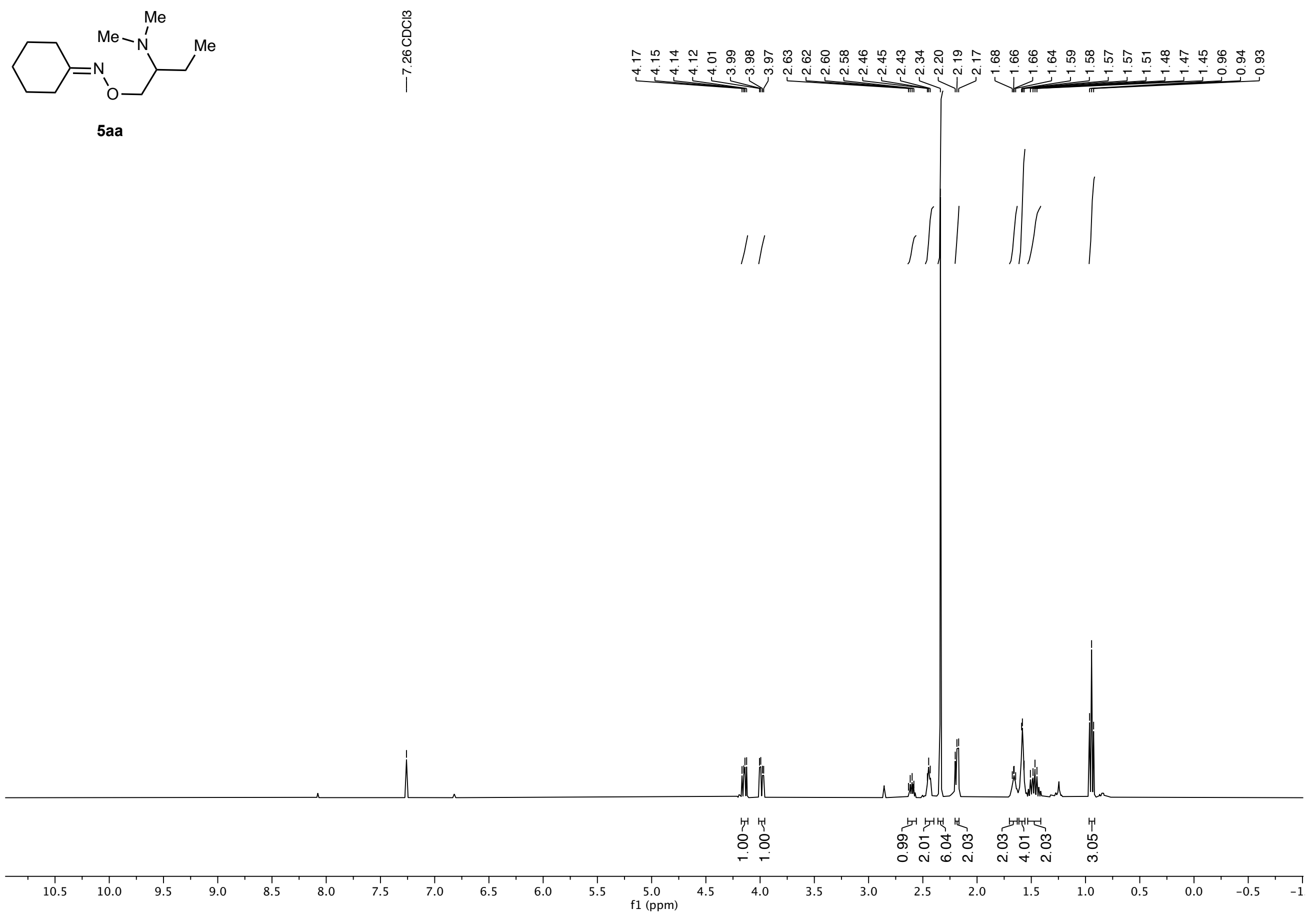
5aa
7.26 CDCl3
4.17 4.15 4.14 4.12 4.01 3.99 3.98 3.97 2.63 2.62 2.60 2.58 2.46 2.45 2.43 2.34 2.20 2.19 2.17 1.68 1.66 1.66 1.64 1.59 1.58 1.57 1.57 1.51 1.48 1.47 1.45 0.96 0.94 0.93
1.00 1.00 0.99 2.01 6.04 2.03 2.03 4.01 2.03 3.05
f1 (ppm)

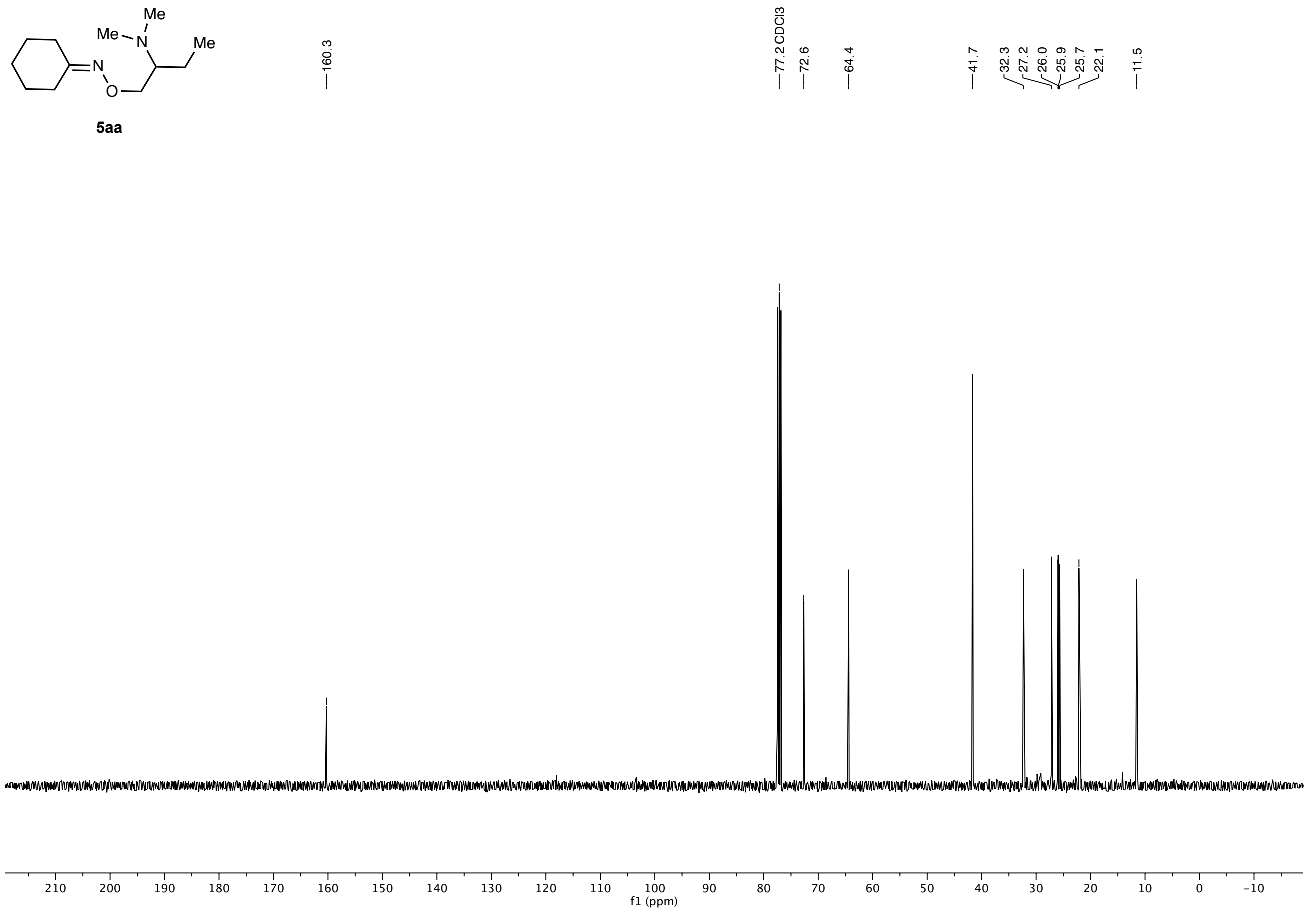
5aa
160.3
77.2 CDCl3
72.6
64.4
41.7
32.3 27.2 26.0 25.9 25.7 22.1
11.5
f1 (ppm)

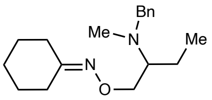

Bn
Me
N
Me
N
O

5ab

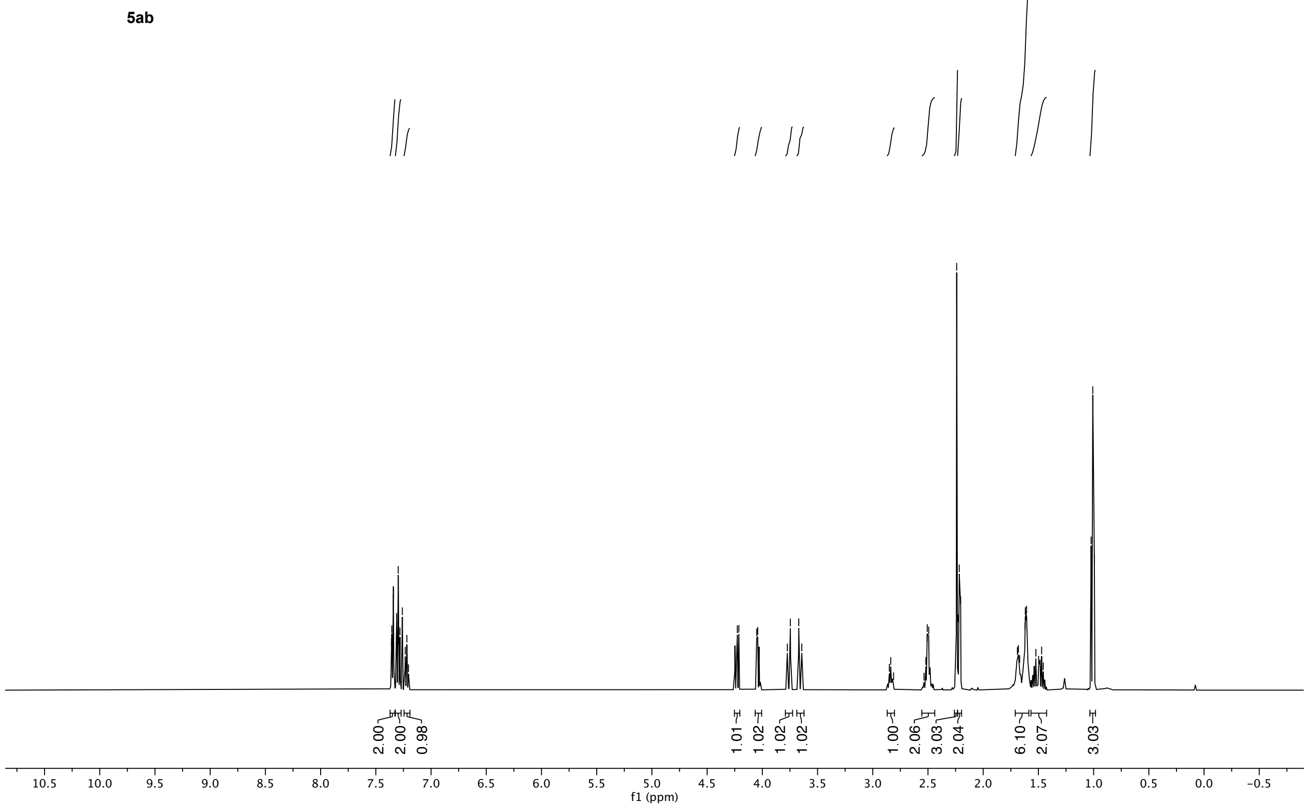

7.36 7.36 7.34 7.34 7.31 7.30 7.29 7.28 7.26 CDCl3 7.23 7.22 7.20
4.23 4.21 4.05 4.04 3.77 3.75 3.67 3.64 2.85 2.84 2.81 2.54 2.52 2.51 2.51 2.49 2.24 2.23 2.22 2.20 1.69 1.68 1.67 1.62 1.62 1.61 1.61 1.61 1.52 1.50 1.47 1.46 1.02 1.01 0.99
2.00 2.00 0.98 1.01 1.02 1.02 1.02 1.00 2.06 3.03 2.04 6.10 2.07 3.03
f1 (ppm)

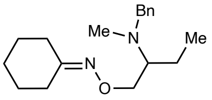

Bn
Me
N
Me
N
O

5ab

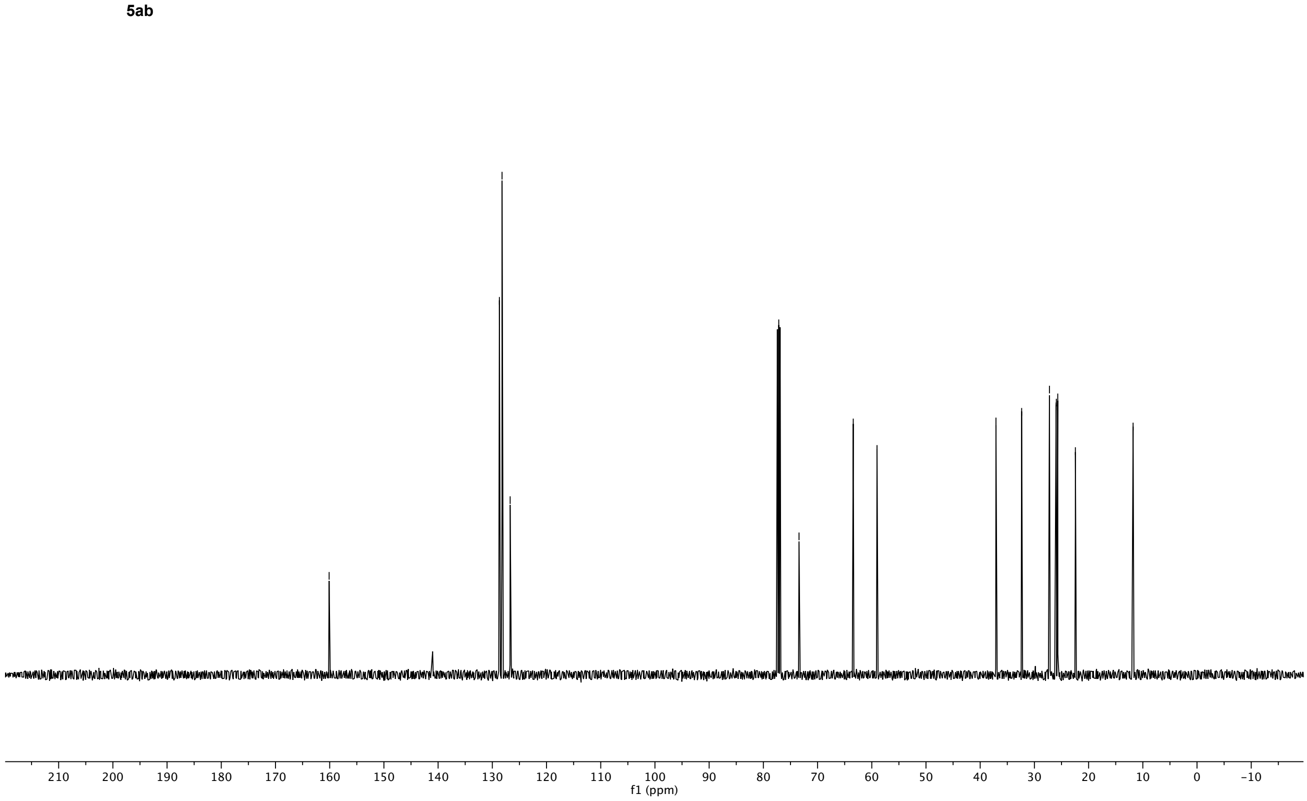

160.1
128.7 128.2 126.7
77.2 CDCl3
73.4
63.4
59.0
37.1 32.4 27.2 26.0 26.0 25.7 22.4
11.8
f1 (ppm)

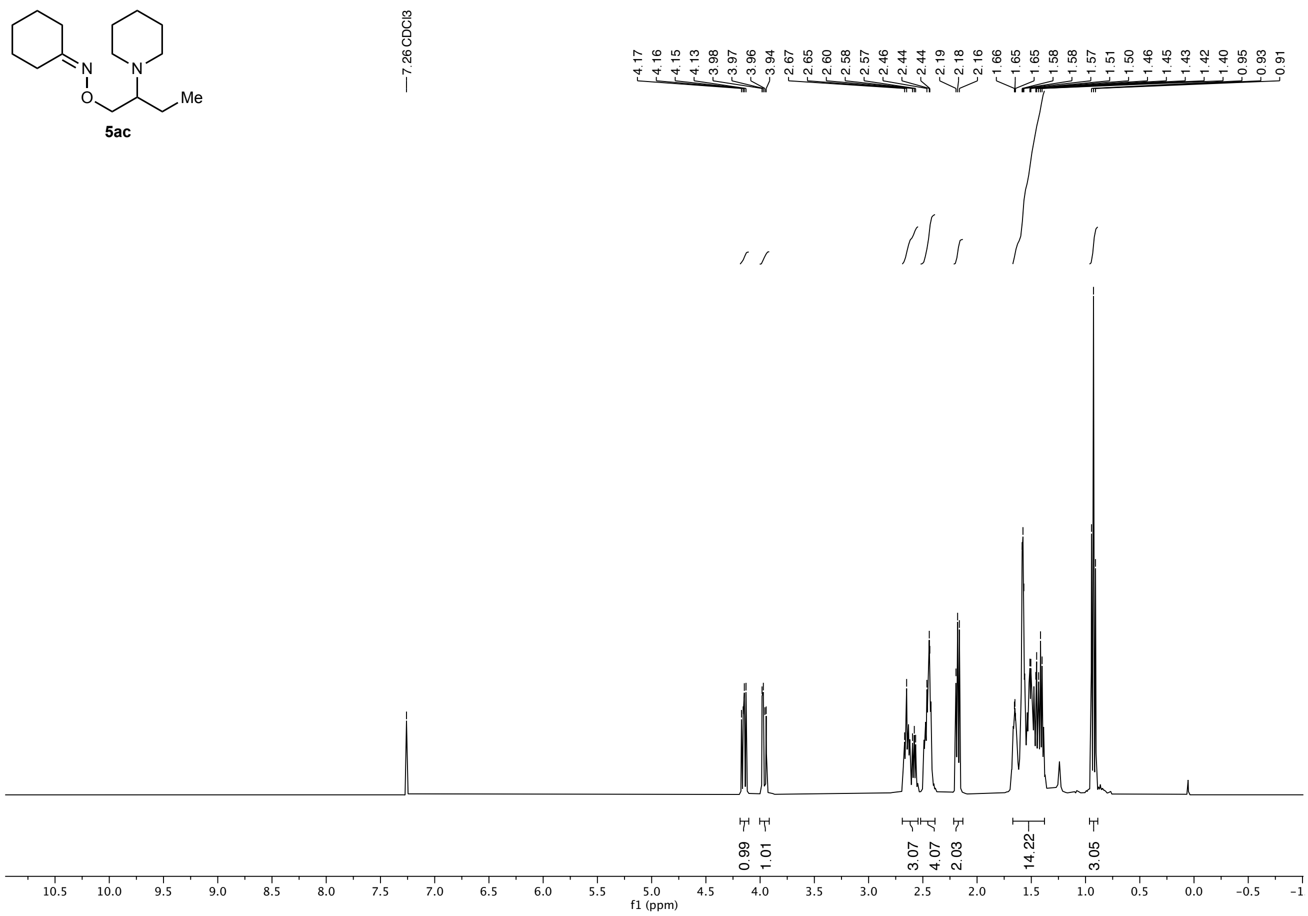

5ac
N
O
Me
7.26 CDCl3
4.17 4.16 4.15 4.13 3.98 3.97 3.96 3.94 2.67 2.65 2.60 2.58 2.57 2.46 2.44 2.44 2.19 2.18 2.16 1.66 1.65 1.65 1.58 1.58 1.57 1.51 1.50 1.46 1.45 1.43 1.42 1.40 0.95 0.93 0.91
0.99
1.01
3.07
4.07
2.03
14.22
3.05
10.5 10.0 9.5 9.0 8.5 8.0 7.5 7.0 6.5 6.0 5.5 5.0 4.5 4.0 3.5 3.0 2.5 2.0 1.5 1.0 0.5 0.0 −0.5 −1
f1 (ppm)

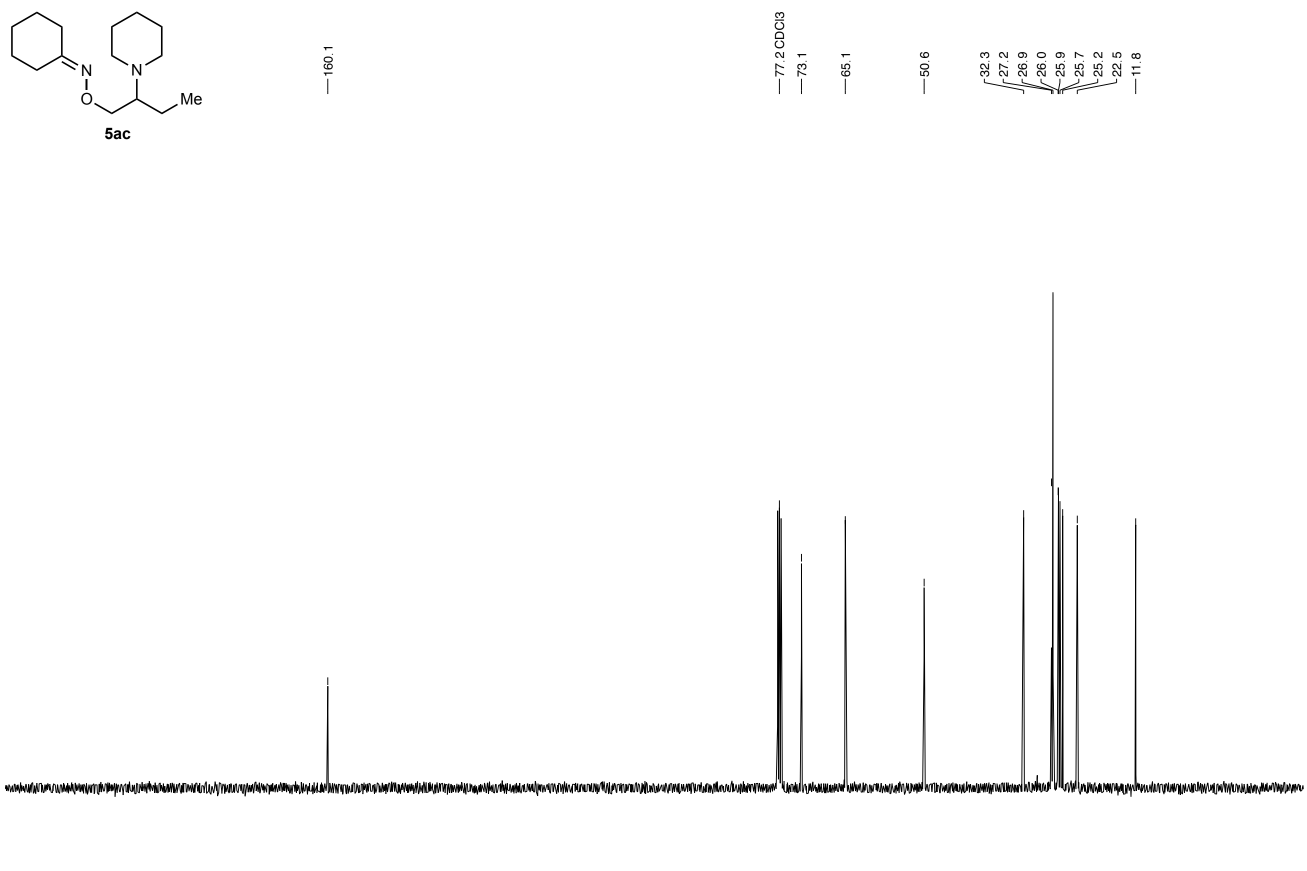

5ac
N
O
Me
160.1
77.2 CDCl3
73.1
65.1
50.6
32.3 27.2 26.9 26.0 25.9 25.7 25.2 22.5
11.8
210 200 190 180 170 160 150 140 130 120 110 100 90 80 70 60 50 40 30 20 10 0 −10
f1 (ppm)

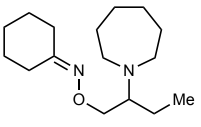
N
O
N
Me

5ad

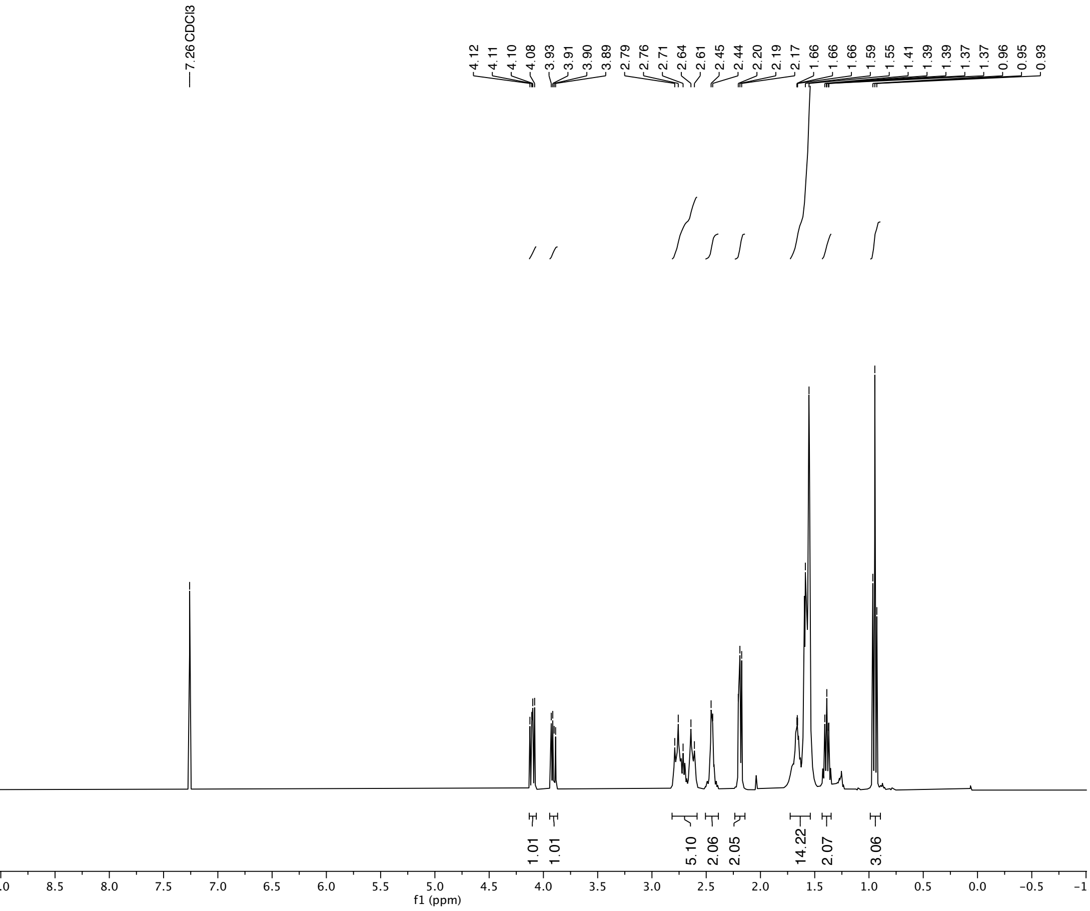
7.26 CDCl3
4.12
4.11
4.10
4.08
3.93
3.91
3.90
3.89
2.79
2.76
2.71
2.64
2.61
2.45
2.44
2.20
2.19
2.17
1.66
1.66
1.66
1.59
1.55
1.41
1.39
1.39
1.37
1.37
0.96
0.95
0.93
1.01
1.01
5.10
2.06
2.05
14.22
2.07
3.06
f1 (ppm)

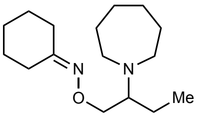
N
O
N
Me

5ad

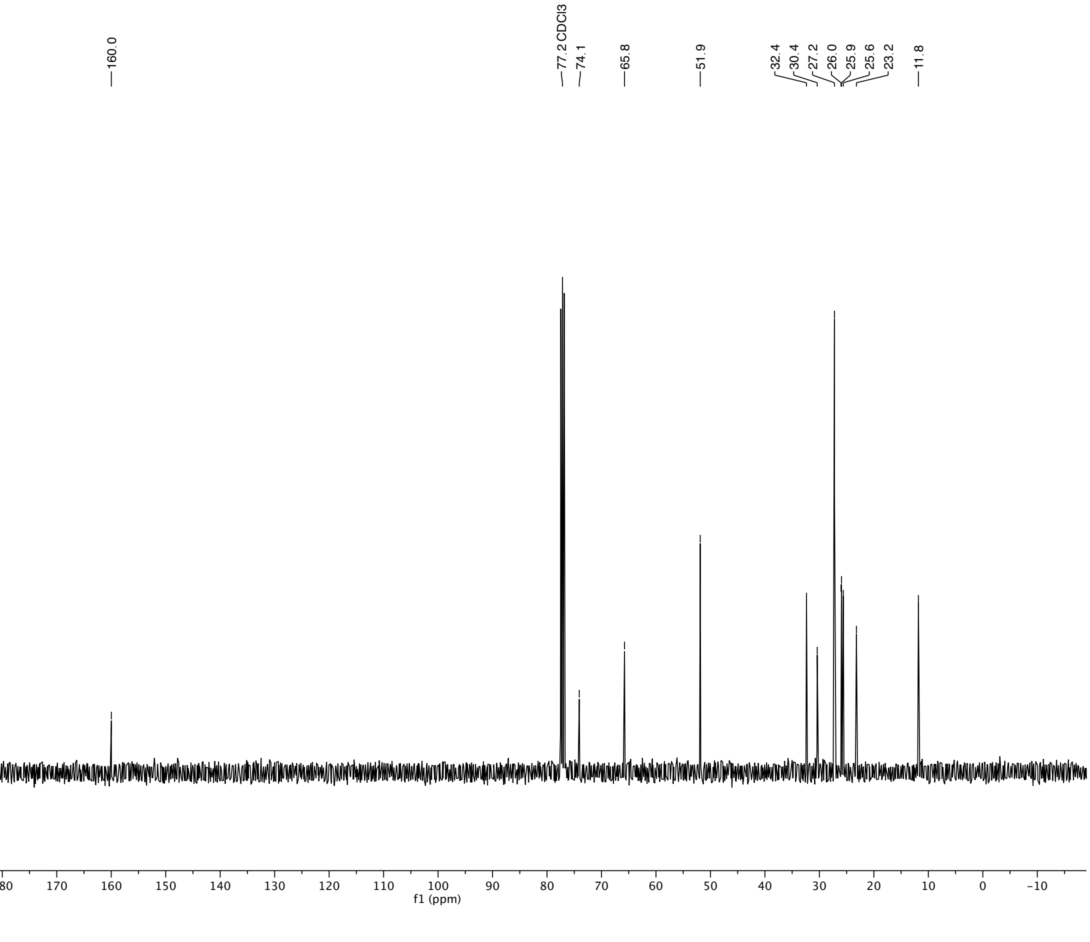
160.0
77.2 CDCl3
74.1
65.8
51.9
32.4
30.4
27.2
26.0
25.9
25.6
23.2
11.8
f1 (ppm)

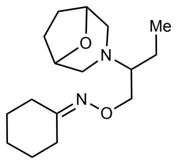

Me
O
N
N
O

5ae

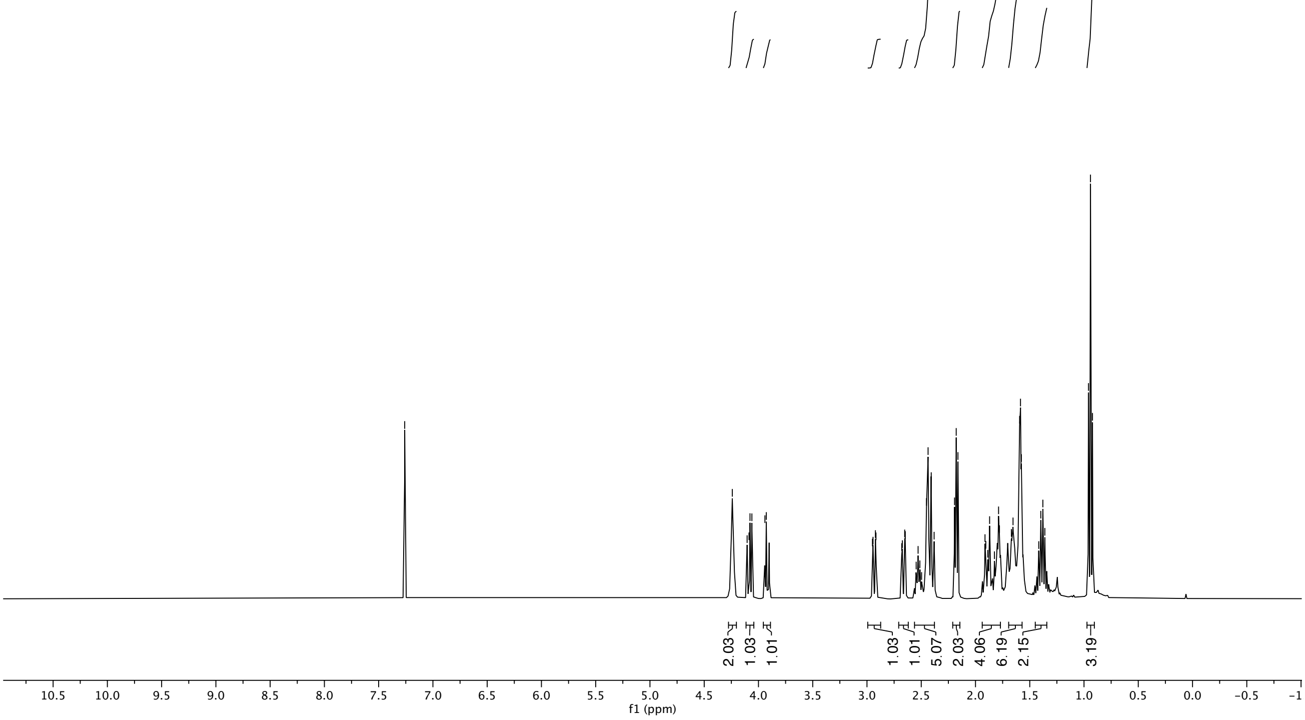

7.26 CDCl3
4.24 4.10 4.09 4.08 4.06 3.94 3.93 2.95 2.94 2.92 2.92 2.68 2.67 2.65 2.65 2.55 2.53 2.51 2.50 2.45 2.44 2.41 2.38 2.19 2.18 2.16 1.91 1.90 1.89 1.87 1.83 1.79 1.78 1.67 1.65 1.59 1.59 1.58 1.42 1.40 1.38 1.36 0.96 0.94 0.92
2.03 1.03 1.01 1.03 1.01 5.07 2.03 4.06 6.19 2.15 3.19
f1 (ppm)

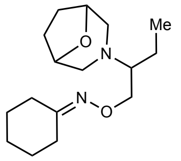

Me
O
N
N
O

5ae

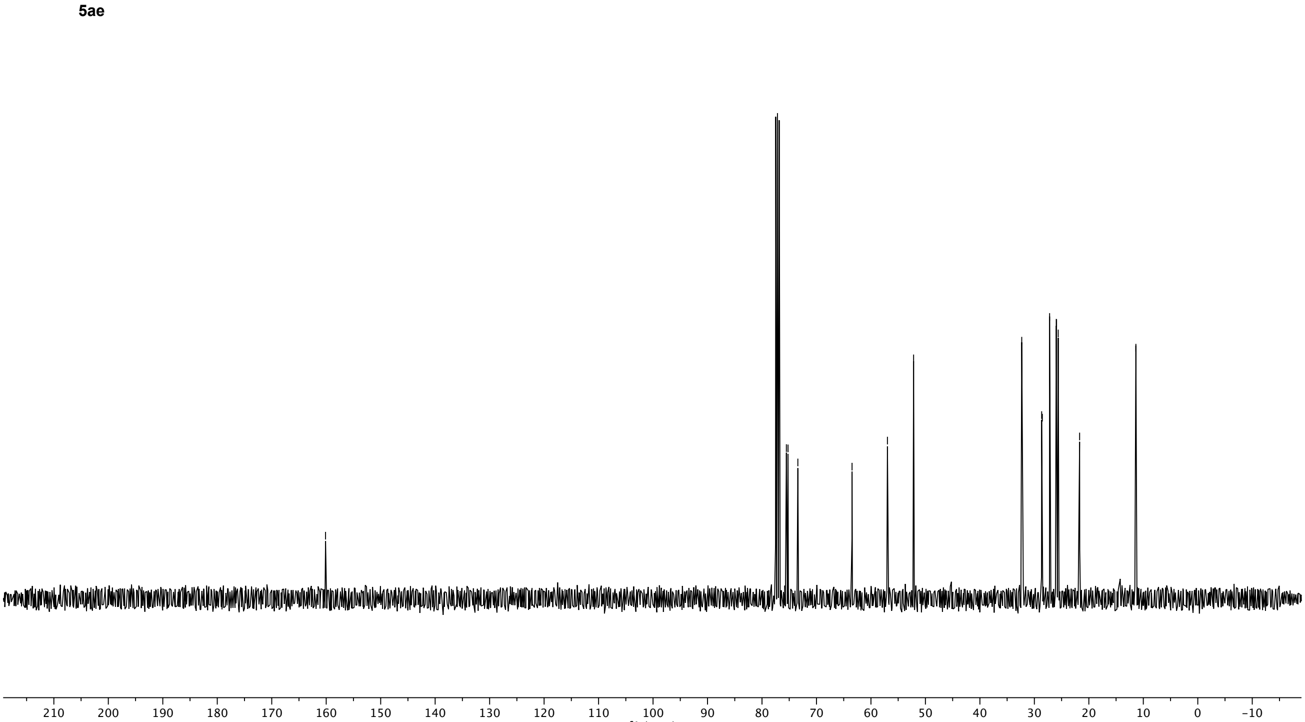

160.1
77.2 CDCl3 75.5 75.2 73.4
63.5 57.0 52.2
32.3 28.7 28.5 27.2 26.0 25.9 25.6 21.7 11.4
f1 (ppm)

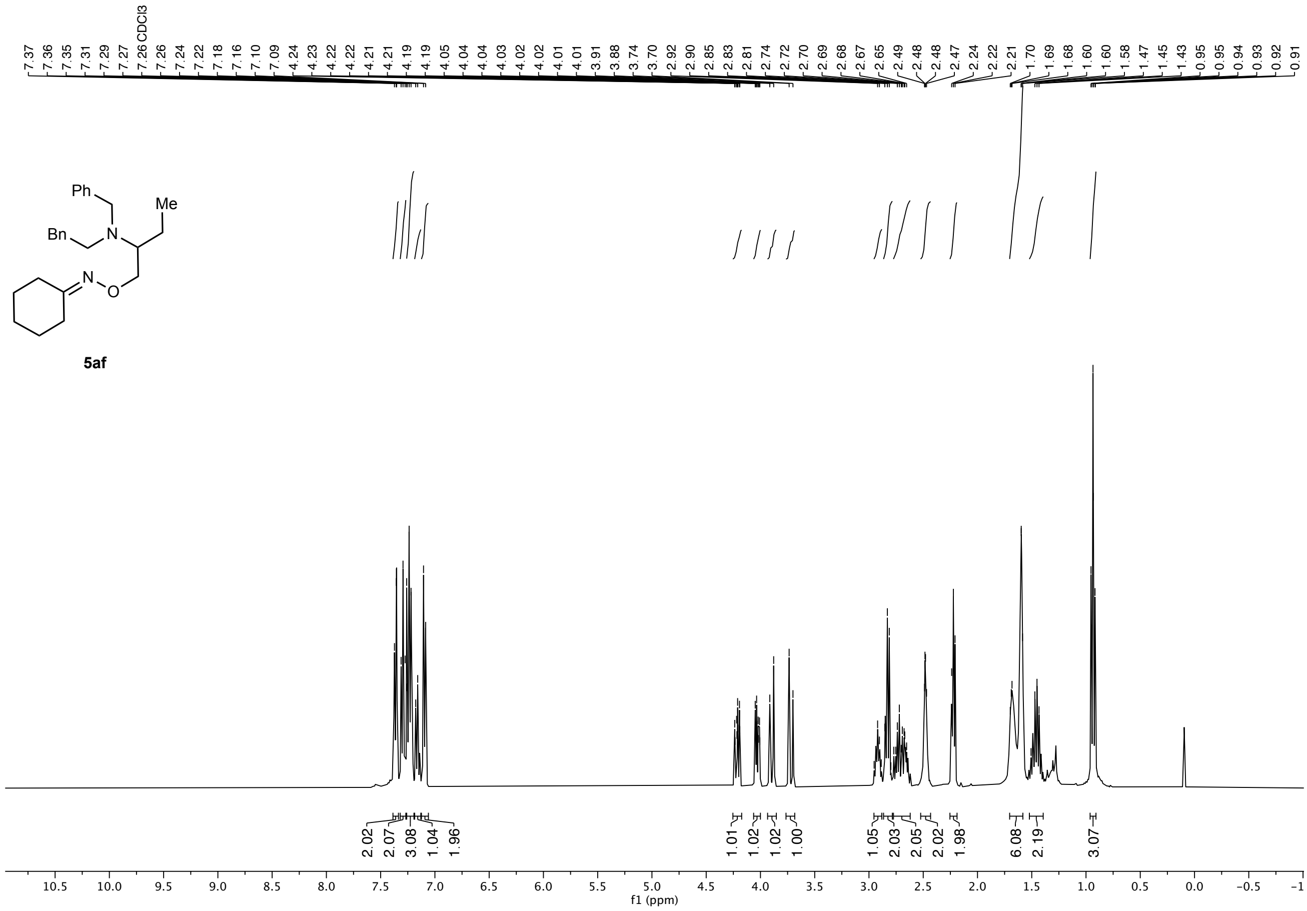
7.37 7.36 7.35 7.31 7.29 7.27 7.26 CDCl3 7.24 7.22 7.18 7.16 7.10 7.09 4.24 4.23 4.22 4.22 4.21 4.21 4.19 4.19 4.05 4.04 4.04 4.03 4.02 4.02 4.01 4.01 3.91 3.88 3.74 3.70 2.92 2.90 2.85 2.83 2.81 2.74 2.72 2.70 2.69 2.68 2.67 2.65 2.49 2.48 2.48 2.47 2.24 2.22 2.21 1.70 1.69 1.68 1.60 1.60 1.58 1.47 1.45 1.43 0.95 0.94 0.93 0.92 0.91
Ph
Me
Bn
N
O
5af
2.02 2.07 3.08 1.04 1.96 1.01 1.02 1.02 1.00 1.05 2.03 2.05 2.02 1.98 6.08 2.19 3.07
f1 (ppm)

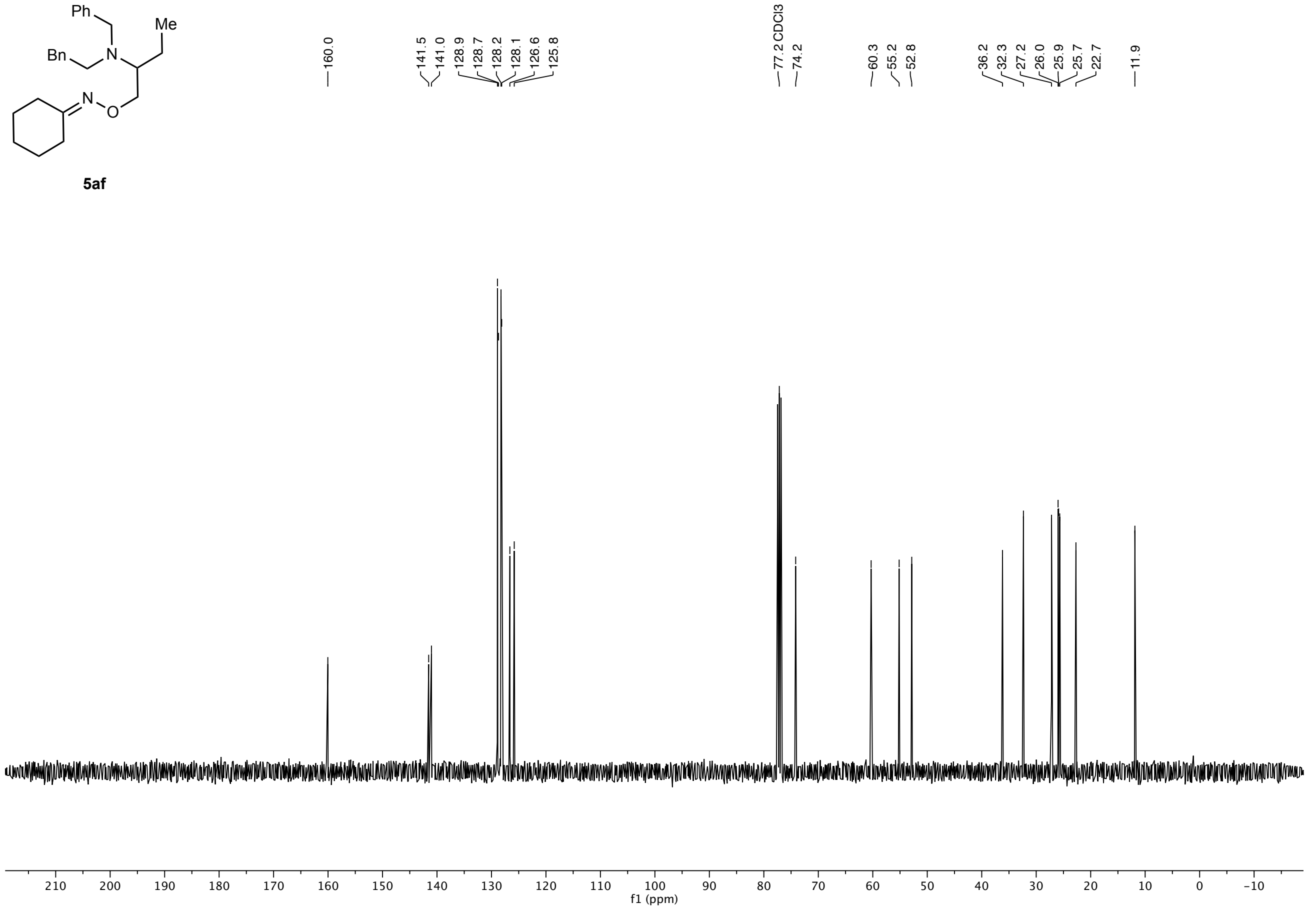
160.0 141.5 141.0 128.9 128.7 128.2 128.1 126.6 125.8 77.2 CDCl3 74.2 60.3 55.2 52.8 36.2 32.3 27.2 26.0 25.9 25.7 22.7 11.9
Ph
Me
Bn
N
O
5af
f1 (ppm)

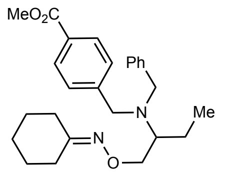

MeO2C
Ph
N
Me
N
O

5ag

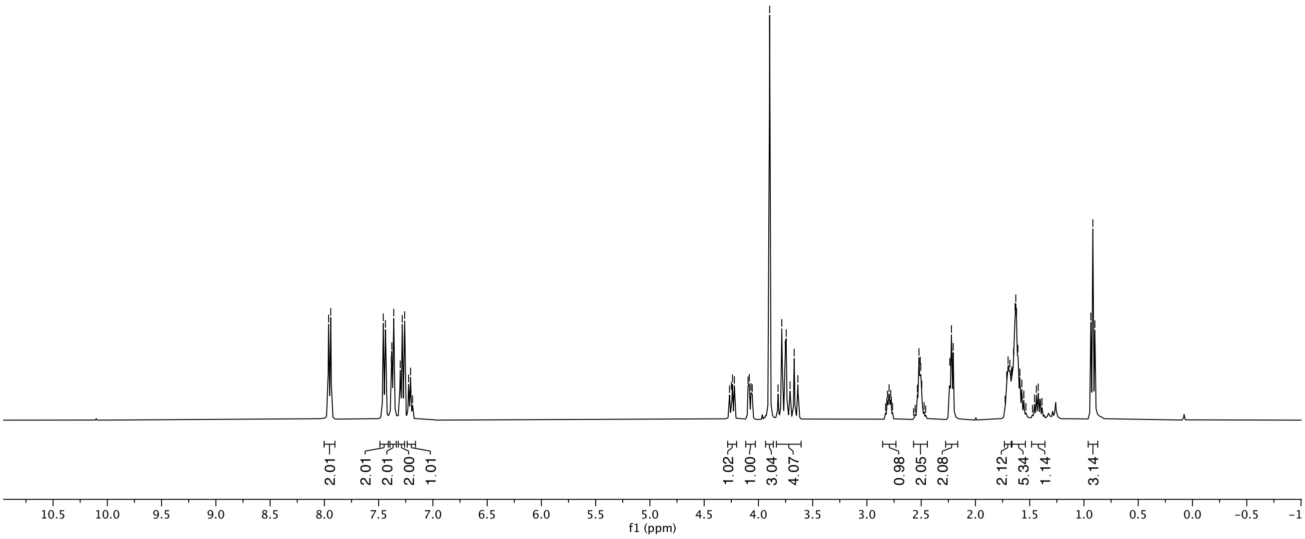

7.96 7.94 7.46 7.44 7.38 7.38 7.36 7.30 7.28 7.26 7.26 CDCl3 7.22 7.21 7.21 7.19 4.27 4.25 4.24 4.22 4.10 4.08 4.07 4.06 3.90 3.82 3.78 3.75 3.74 3.71 3.67 3.64 2.82 2.81 2.80 2.78 2.78 2.76 2.53 2.52 2.51 2.50 2.49 2.47 2.24 2.22 2.22 2.21 1.73 1.71 1.71 1.70 1.68 1.67 1.66 1.65 1.63 1.63 1.62 1.61 1.59 1.57 1.55 1.46 1.44 1.42 1.39 0.94 0.92 0.90
2.01 2.01 2.01 2.00 1.01 1.02 1.00 3.04 4.07 0.98 2.05 2.08 2.12 5.34 1.14 3.14
10.5 10.0 9.5 9.0 8.5 8.0 7.5 7.0 6.5 6.0 5.5 5.0 4.5 4.0 3.5 3.0 2.5 2.0 1.5 1.0 0.5 0.0 -0.5 -1
f1 (ppm)

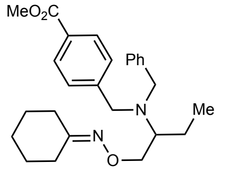

MeO2C
Ph
N
Me
N
O

5ag

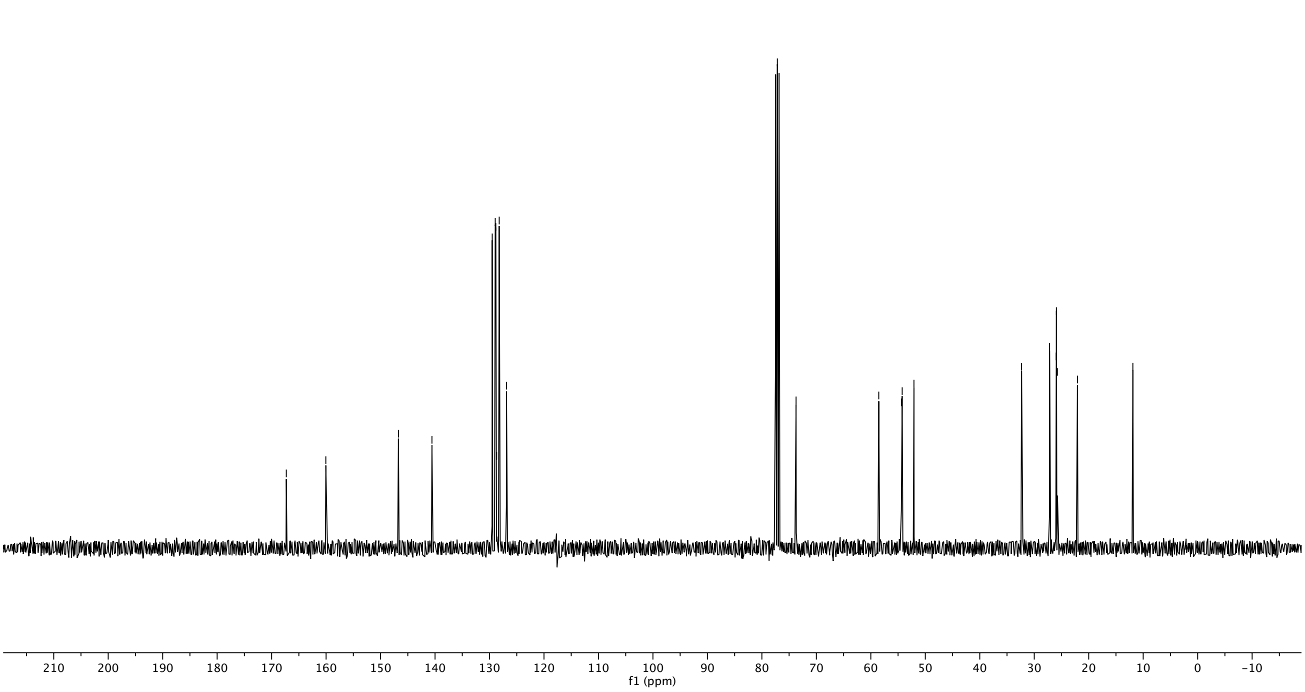

167.3 160.0 146.7 140.6 129.5 128.9 128.8 128.7 128.2 126.9 77.2 CDCl3 73.7 58.5 54.4 54.2 52.1 32.3 27.2 26.0 25.9 25.8 22.1 11.9
210 200 190 180 170 160 150 140 130 120 110 100 90 80 70 60 50 40 30 20 10 0 -10
f1 (ppm)

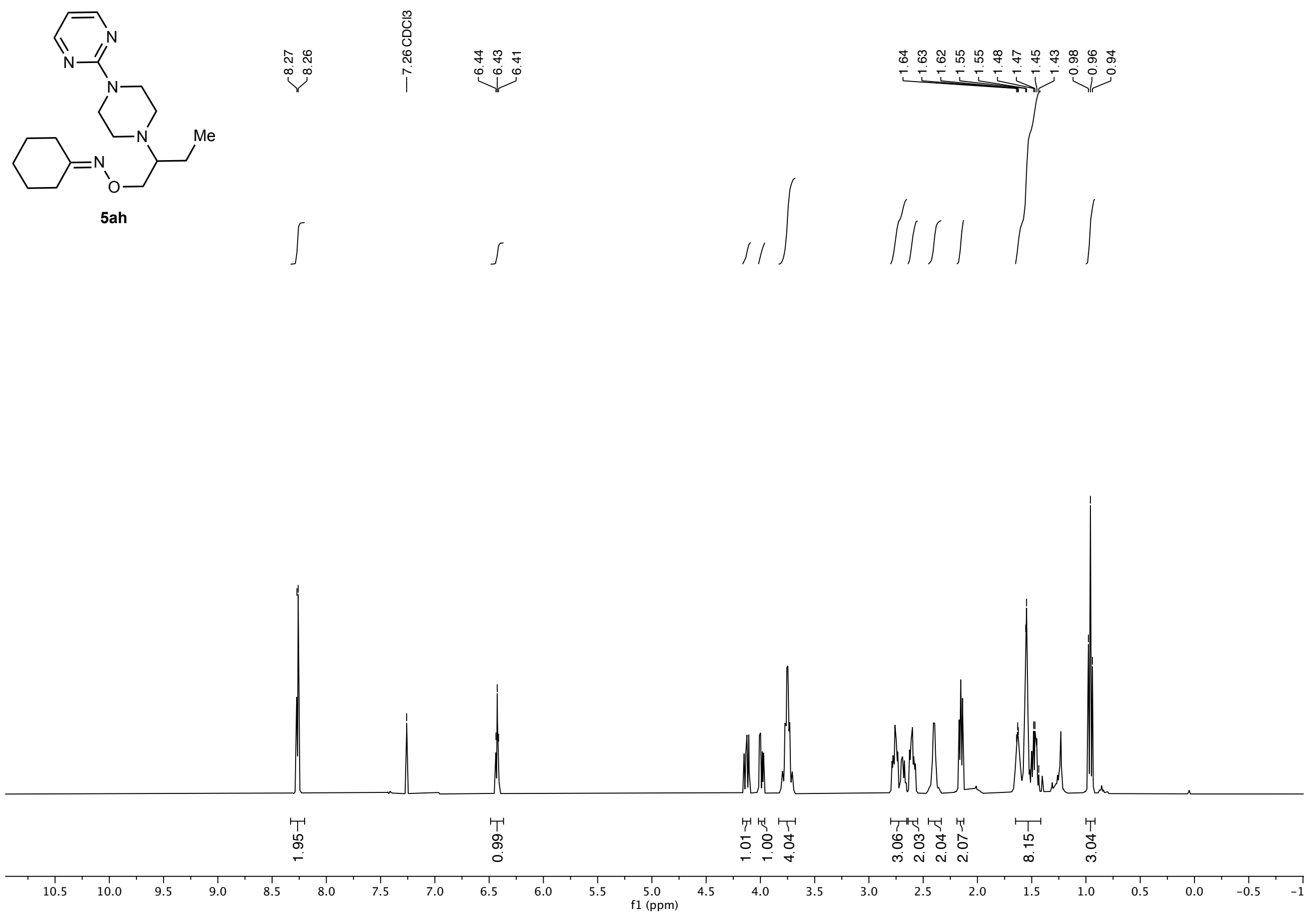
5ah
8.27
8.26
7.26 CDCl3
6.44
6.43
6.41
1.64
1.63
1.62
1.55
1.55
1.48
1.47
1.45
1.43
0.98
0.96
0.94
1.95
0.99
1.01
1.00
4.04
3.06
2.03
2.04
2.07
8.15
3.04
f1 (ppm)

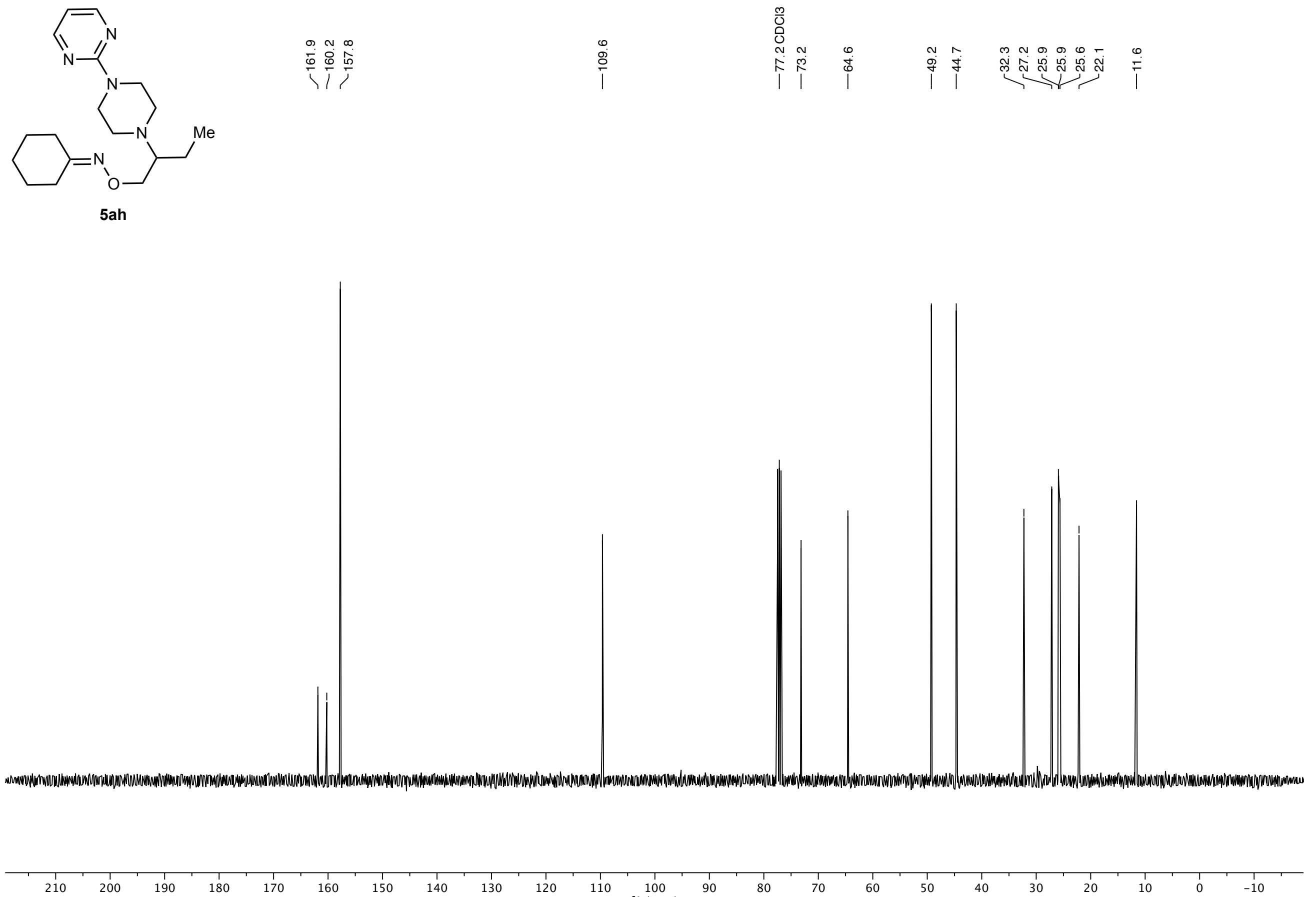
5ah
161.9
160.2
157.8
109.6
77.2 CDCl3
73.2
64.6
49.2
44.7
32.3
27.2
25.9
25.9
25.6
22.1
11.6
f1 (ppm)

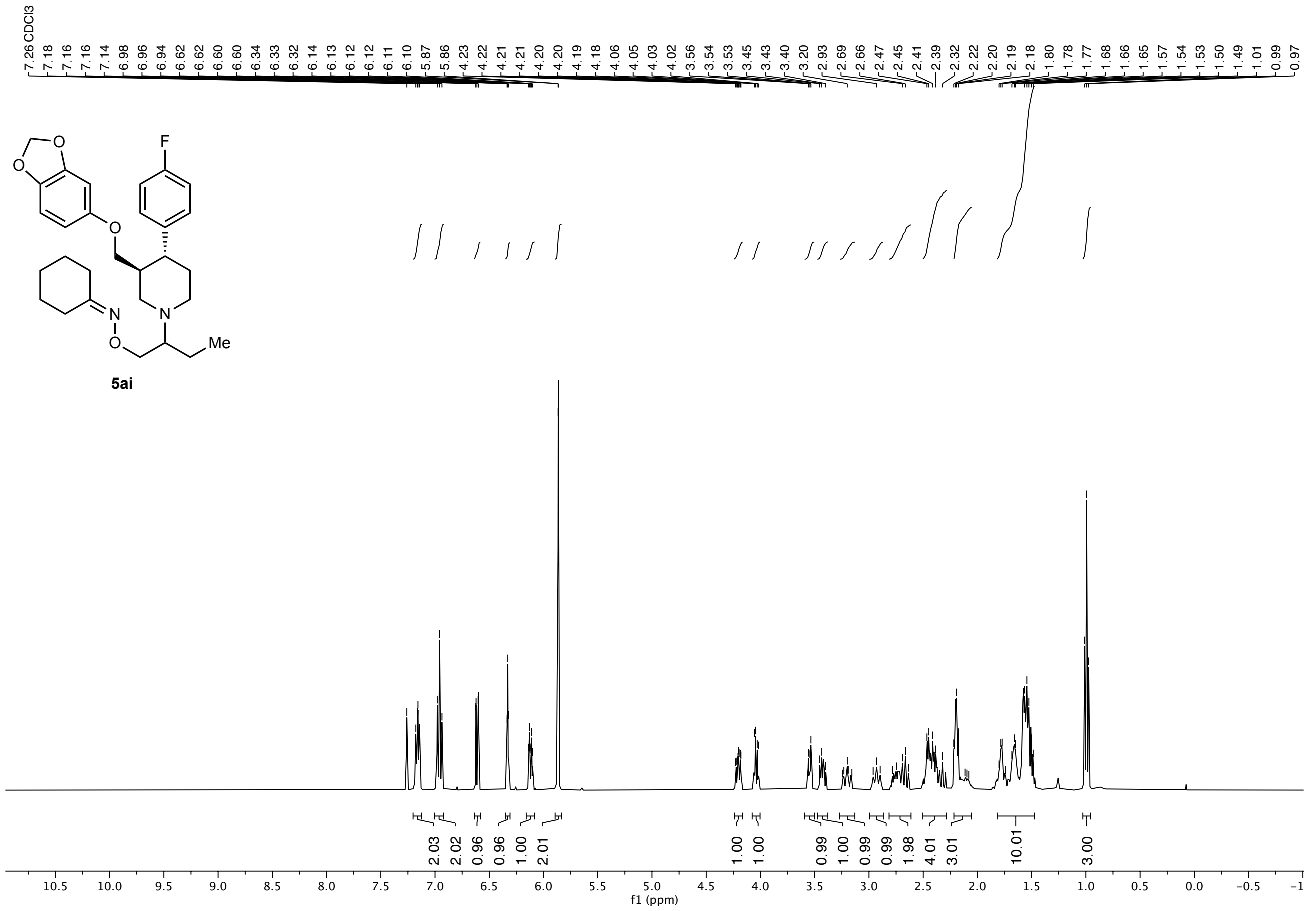
7.26 CDCl3
7.18
7.16
7.16
7.14
6.98
6.96
6.94
6.62
6.60
6.60
6.34
6.33
6.32
6.14
6.13
6.12
6.12
6.11
6.10
5.87
5.86
4.23
4.22
4.21
4.21
4.20
4.20
4.19
4.18
4.06
4.05
4.03
4.02
3.56
3.54
3.53
3.45
3.43
3.40
3.20
2.93
2.69
2.66
2.47
2.45
2.41
2.39
2.32
2.22
2.20
2.19
2.18
1.80
1.78
1.77
1.68
1.66
1.65
1.57
1.54
1.53
1.50
1.49
1.01
0.99
0.97
F
O
O
O
N
O
N
Me
5ai
2.03
2.02
0.96
0.96
1.00
2.01
1.00
1.00
0.99
1.00
0.99
0.99
1.98
4.01
3.01
10.01
3.00
10.5 10.0 9.5 9.0 8.5 8.0 7.5 7.0 6.5 6.0 5.5 5.0 4.5 4.0 3.5 3.0 2.5 2.0 1.5 1.0 0.5 0.0 −0.5 −1
f1 (ppm)

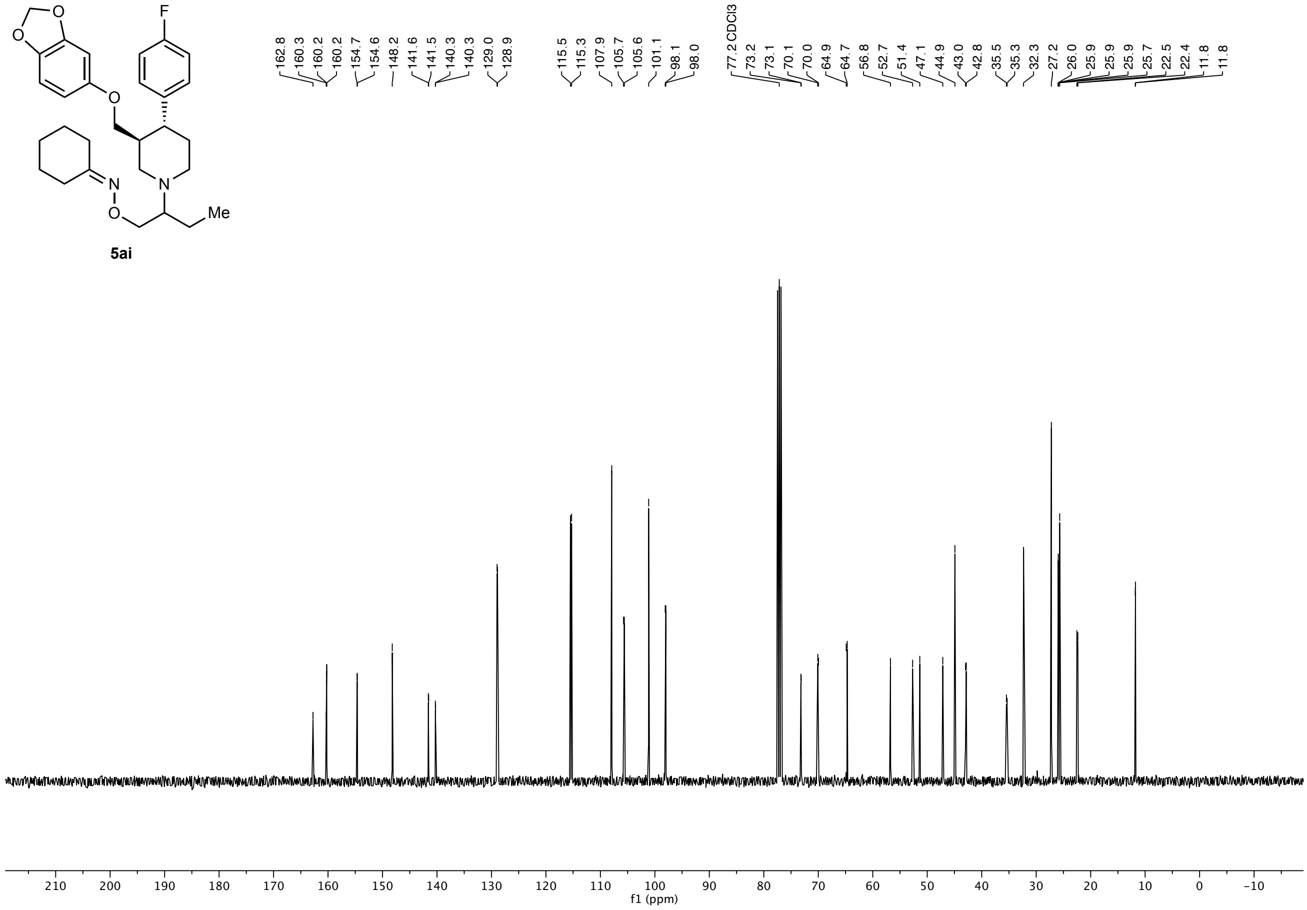
F
O
O
O
N
O
N
Me
5ai
162.8
160.3
160.2
160.2
154.7
154.6
148.2
141.6
141.5
140.3
140.3
129.0
128.9
115.5
115.3
107.9
105.7
105.6
101.1
98.1
98.0
77.2 CDCl3
73.2
73.1
70.1
70.0
64.9
64.7
56.8
52.7
51.4
47.1
44.9
43.0
42.8
35.5
35.3
32.3
27.2
26.0
25.9
25.9
25.9
25.7
22.5
22.4
11.8
11.8
210 200 190 180 170 160 150 140 130 120 110 100 90 80 70 60 50 40 30 20 10 0 −10
f1 (ppm)

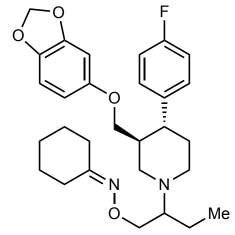

5ai

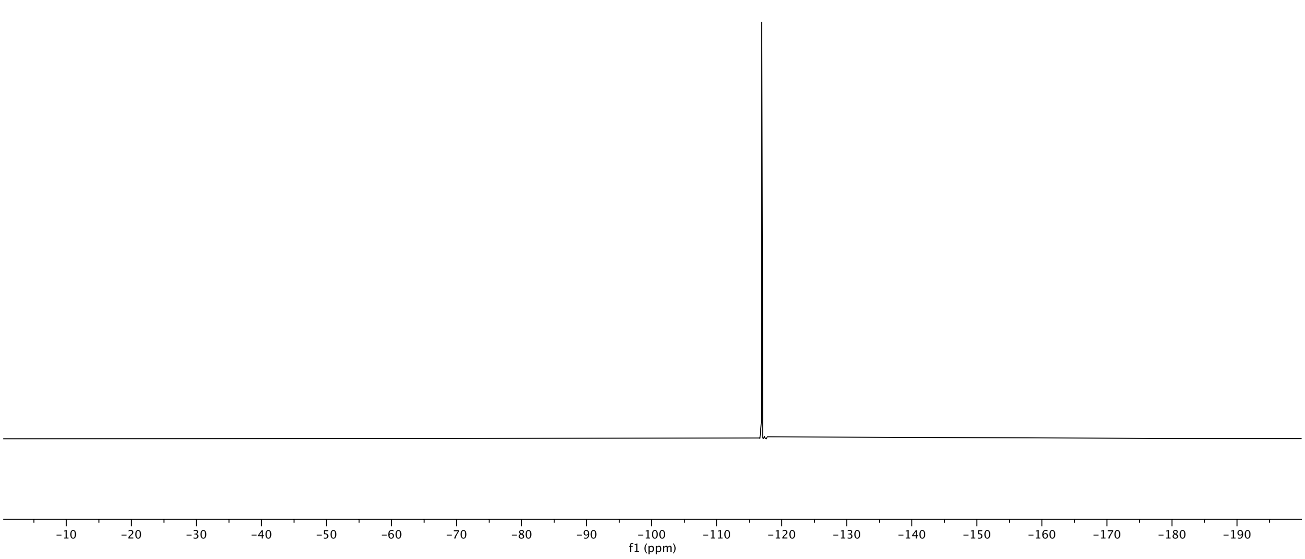

-116.92
-116.93
-10
-20
-30
-40
-50
-60
-70
-80
-90
-100
-110
-120
-130
-140
-150
-160
-170
-180
-190
f1 (ppm)

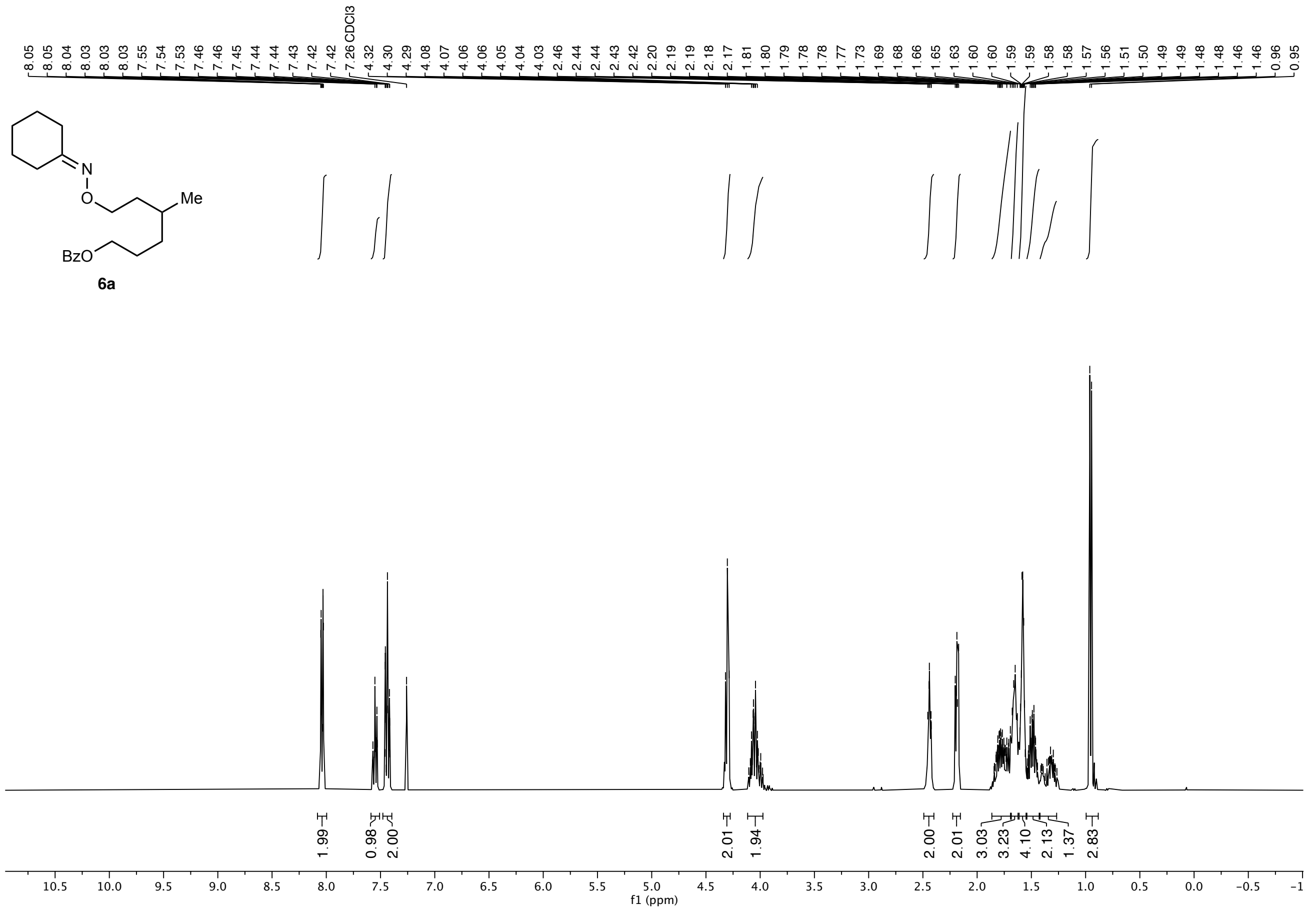
8.05 8.05 8.04 8.03 8.03 8.03 7.55 7.54 7.53 7.46 7.46 7.45 7.44 7.44 7.43 7.42 7.42 7.26 CDCl3 4.32 4.30 4.29 4.08 4.07 4.06 4.06 4.05 4.04 4.03 2.46 2.44 2.44 2.43 2.42 2.20 2.19 2.19 2.18 2.17 1.81 1.80 1.79 1.78 1.78 1.77 1.73 1.69 1.68 1.66 1.65 1.63 1.60 1.60 1.59 1.59 1.59 1.58 1.58 1.57 1.56 1.51 1.50 1.49 1.49 1.48 1.48 1.46 1.46 0.96 0.95
N
O
Me
BzO
6a
1.99 0.98 2.00 2.01 1.94 2.00 2.01 3.03 3.23 4.10 2.13 1.37 2.83
10.5 10.0 9.5 9.0 8.5 8.0 7.5 7.0 6.5 6.0 5.5 5.0 4.5 4.0 3.5 3.0 2.5 2.0 1.5 1.0 0.5 0.0 −0.5 −1
f1 (ppm)

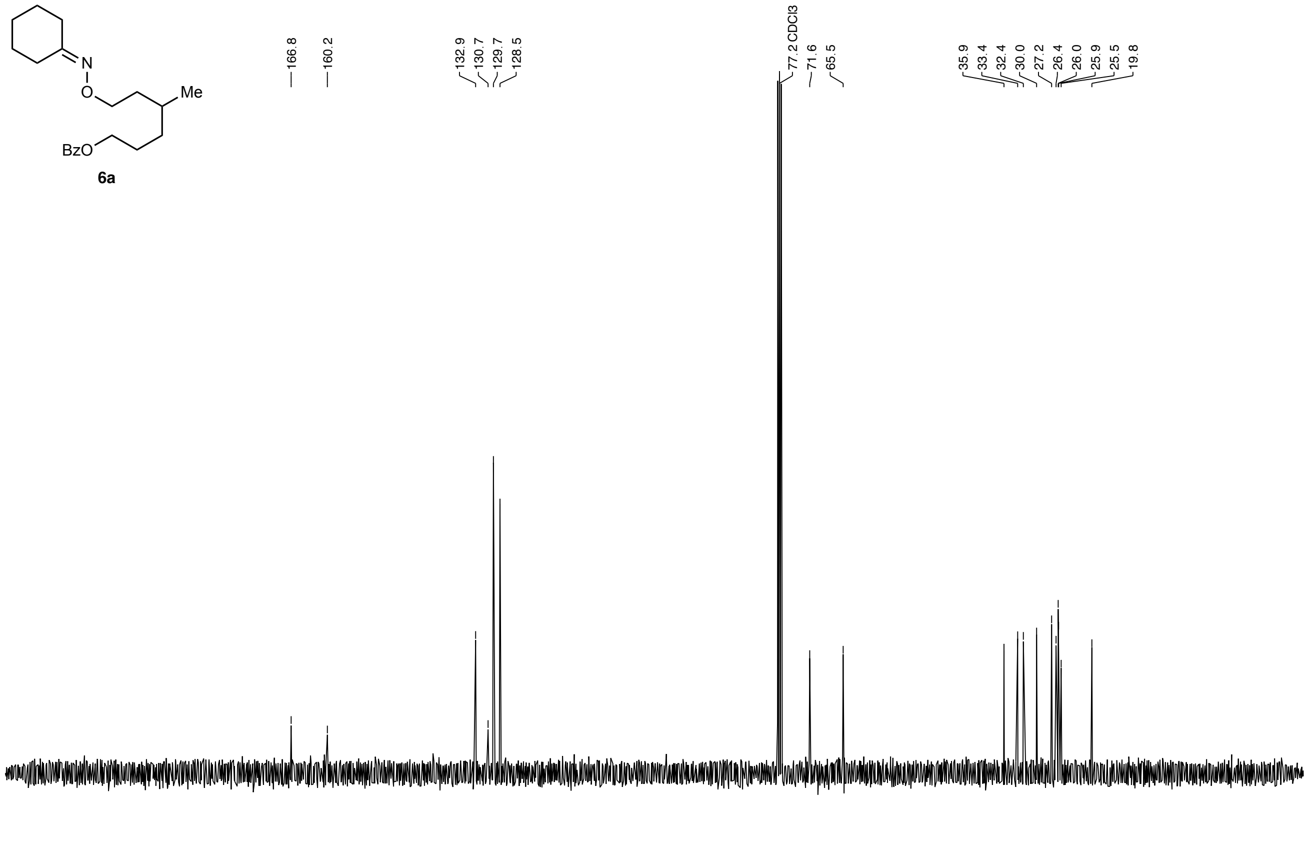
166.8 160.2 132.9 130.7 129.7 128.5 77.2 CDCl3 71.6 65.5 35.9 33.4 32.4 30.0 27.2 26.4 26.0 25.9 25.5 19.8
N
O
Me
BzO
6a
210 200 190 180 170 160 150 140 130 120 110 100 90 80 70 60 50 40 30 20 10 0 −10
f1 (ppm)

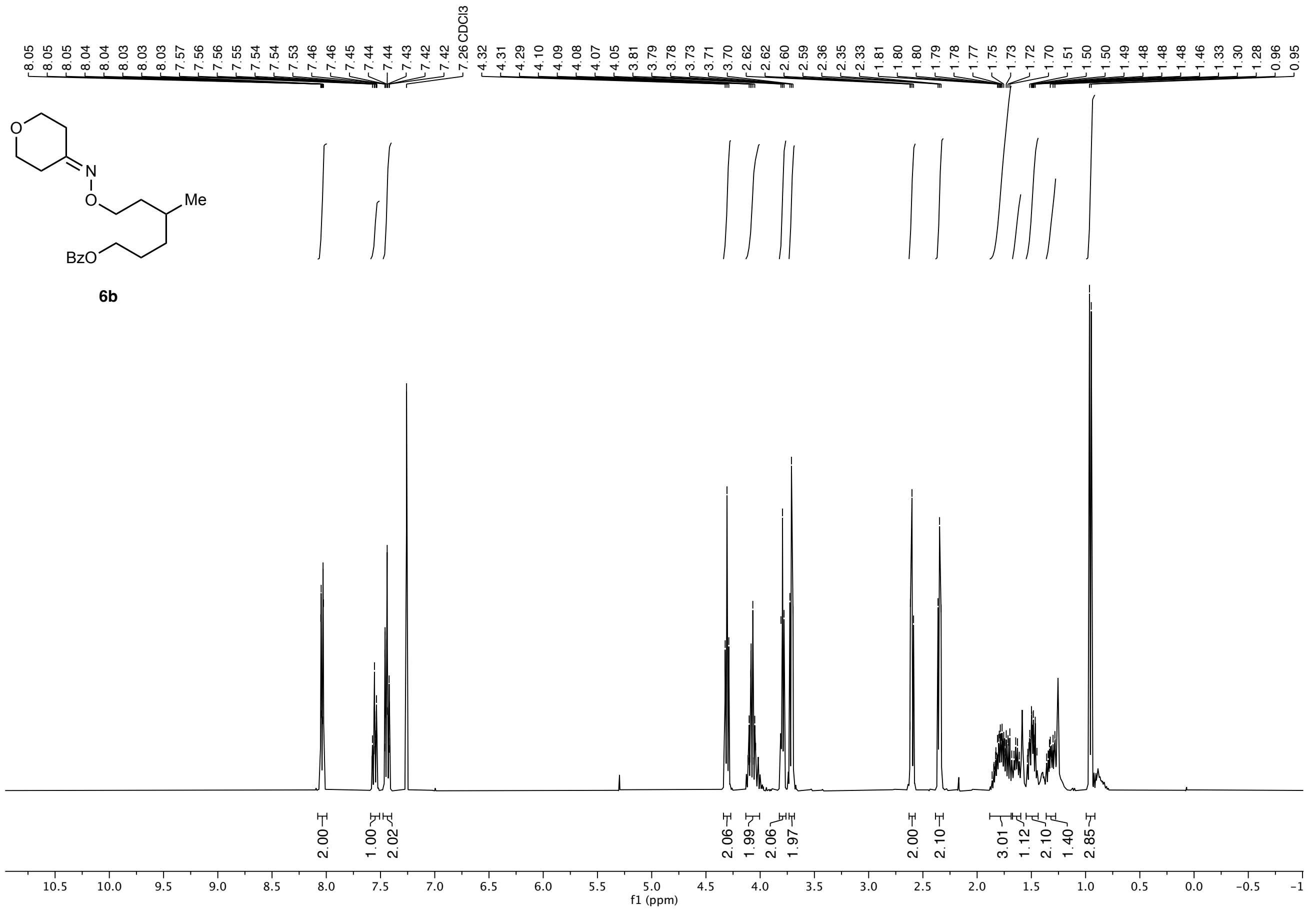
O
N
O
Me
BzO
8.05 8.05 8.05 8.04 8.04 8.03 8.03 8.03 7.57 7.56 7.56 7.55 7.54 7.54 7.53 7.46 7.46 7.45 7.44 7.44 7.43 7.42 7.42 7.26 CDCl3 4.32 4.31 4.29 4.10 4.09 4.08 4.07 4.05 3.81 3.79 3.78 3.73 3.71 3.70 2.62 2.62 2.60 2.59 2.36 2.35 2.33 1.81 1.80 1.80 1.79 1.78 1.77 1.75 1.73 1.72 1.70 1.51 1.50 1.49 1.48 1.48 1.48 1.46 1.33 1.30 1.28 0.96 0.95
2.00 1.00 2.02 2.06 1.99 2.06 1.97 2.00 2.10 3.01 1.12 2.10 1.40 2.85
10.5 10.0 9.5 9.0 8.5 8.0 7.5 7.0 6.5 6.0 5.5 5.0 4.5 4.0 3.5 3.0 2.5 2.0 1.5 1.0 0.5 0.0 −0.5 −1
f1 (ppm)

6b

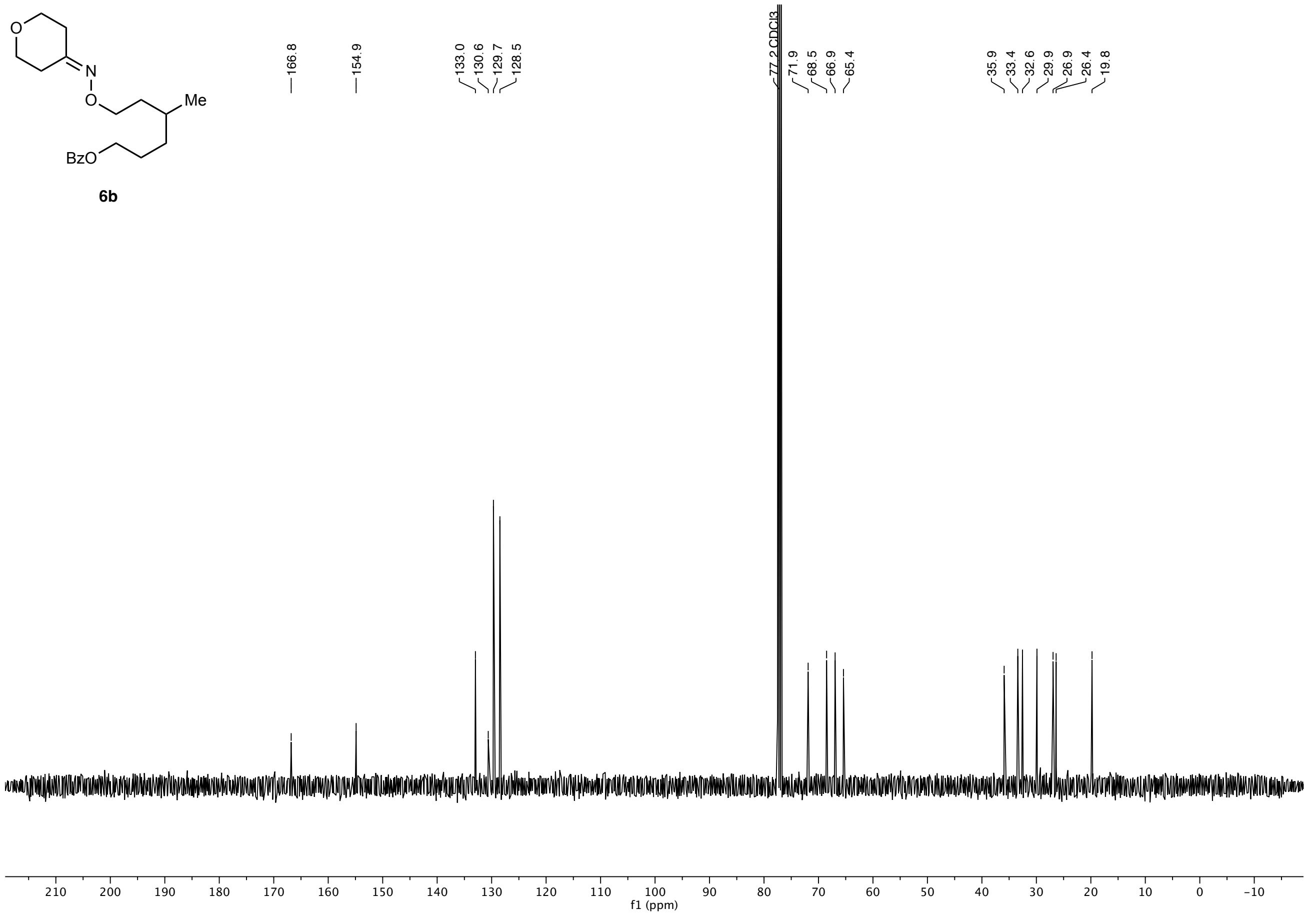
O
N
O
Me
BzO
166.8
154.9
133.0 130.6 129.7 128.5
77.2 CDCl3
71.9 68.5 66.9 65.4
35.9 33.4 32.6 29.9 26.9 26.4 19.8
210 200 190 180 170 160 150 140 130 120 110 100 90 80 70 60 50 40 30 20 10 0 −10
f1 (ppm)

6b

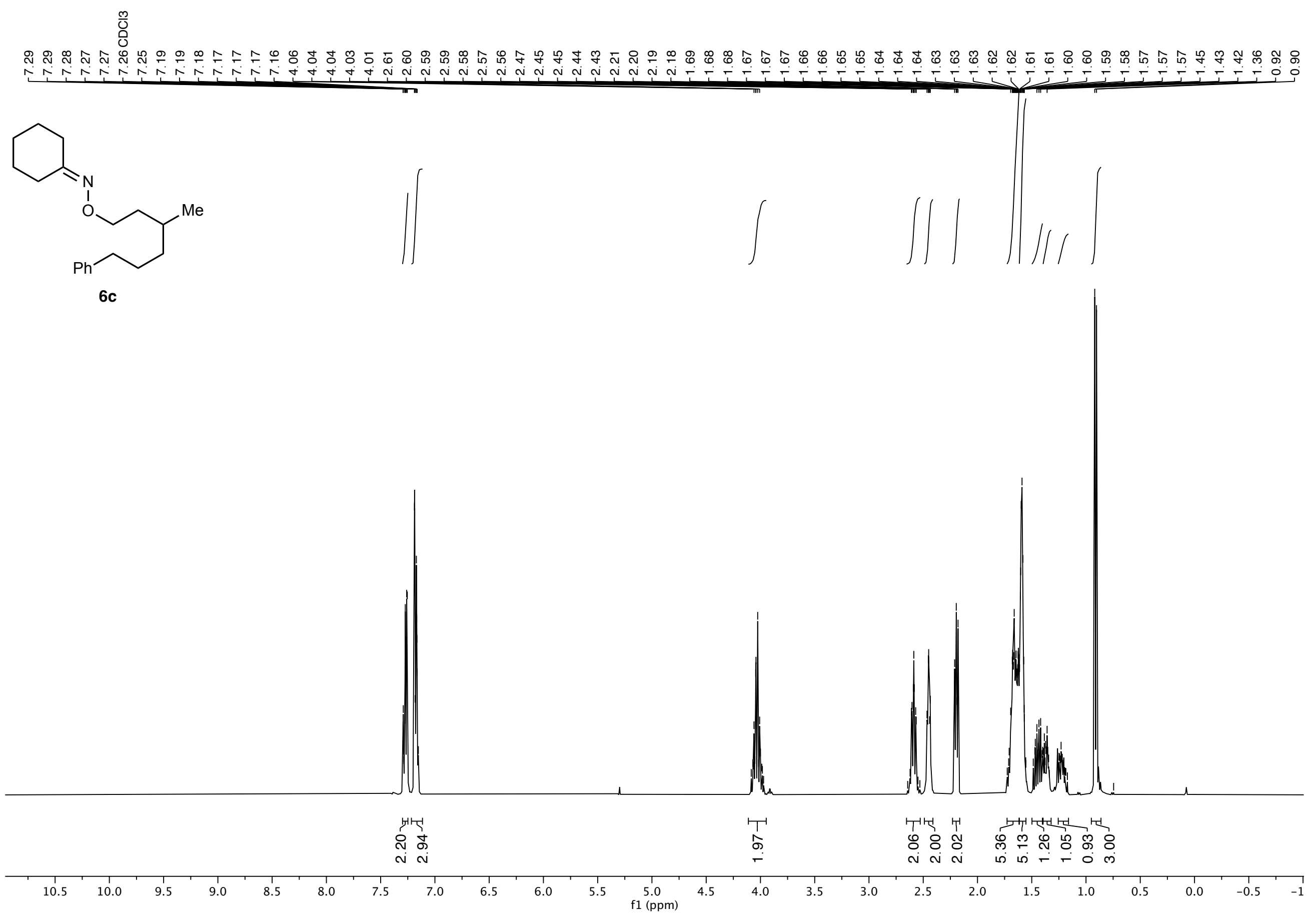
7.29 7.29 7.28 7.27 7.27 7.26 CDCl3 7.25 7.19 7.19 7.18 7.17 7.17 7.17 7.16 4.06 4.04 4.04 4.03 4.01 2.61 2.60 2.59 2.59 2.58 2.57 2.56 2.47 2.45 2.45 2.44 2.43 2.21 2.20 2.19 2.18 1.69 1.68 1.68 1.67 1.67 1.67 1.66 1.66 1.65 1.65 1.64 1.64 1.64 1.63 1.63 1.63 1.62 1.62 1.61 1.61 1.60 1.60 1.59 1.58 1.57 1.57 1.57 1.45 1.43 1.42 1.36 0.92 0.90
N
O
Me
Ph
6c
2.20 2.94 1.97 2.06 2.00 2.02 5.36 5.13 1.26 1.05 0.93 3.00
10.5 10.0 9.5 9.0 8.5 8.0 7.5 7.0 6.5 6.0 5.5 5.0 4.5 4.0 3.5 3.0 2.5 2.0 1.5 1.0 0.5 0.0 −0.5 −1
f1 (ppm)

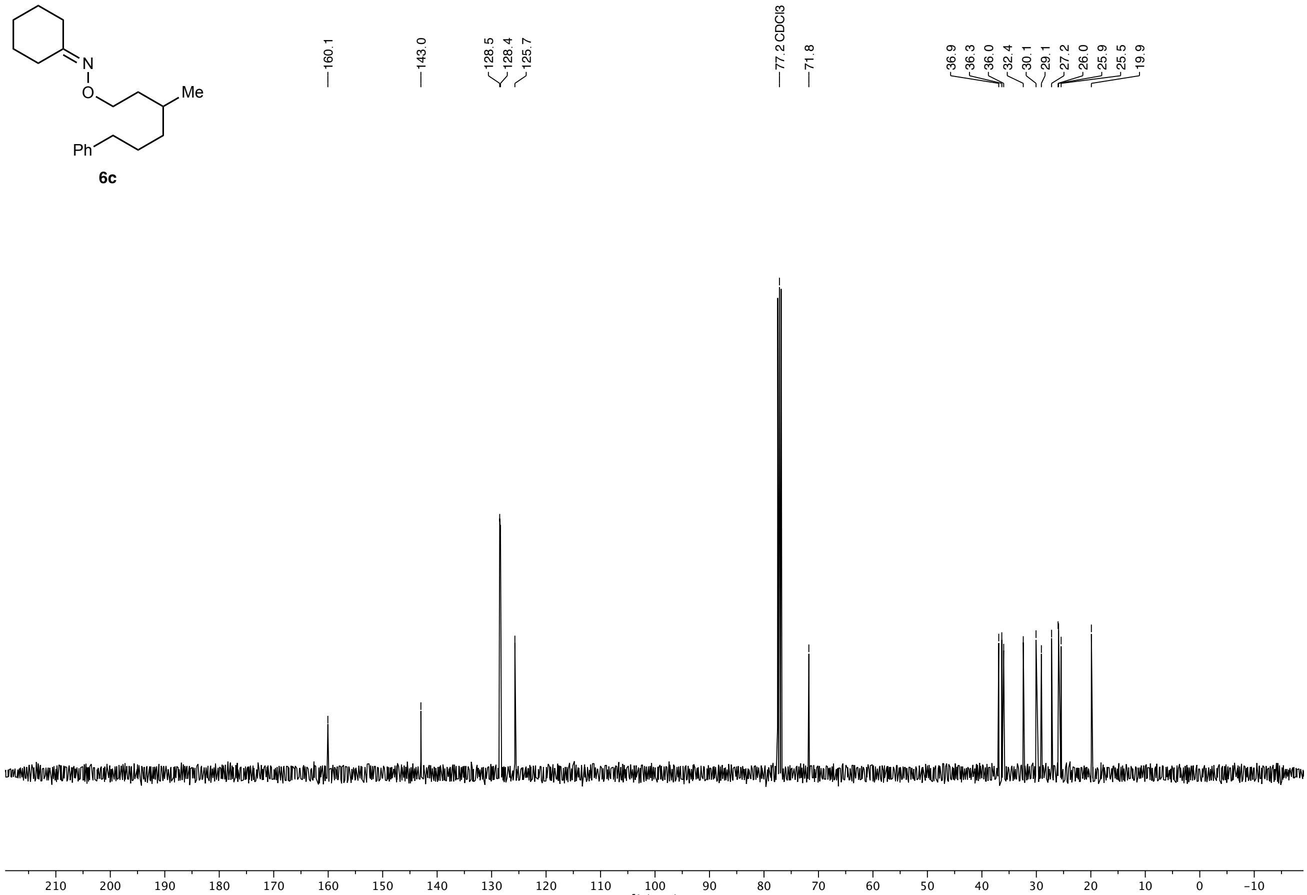
N
O
Me
Ph
6c
160.1
143.0
128.5 128.4 125.7
77.2 CDCl3
71.8
36.9 36.3 36.0 32.4 30.1 29.1 27.2 26.0 25.9 25.5 19.9
210 200 190 180 170 160 150 140 130 120 110 100 90 80 70 60 50 40 30 20 10 0 −10
f1 (ppm)

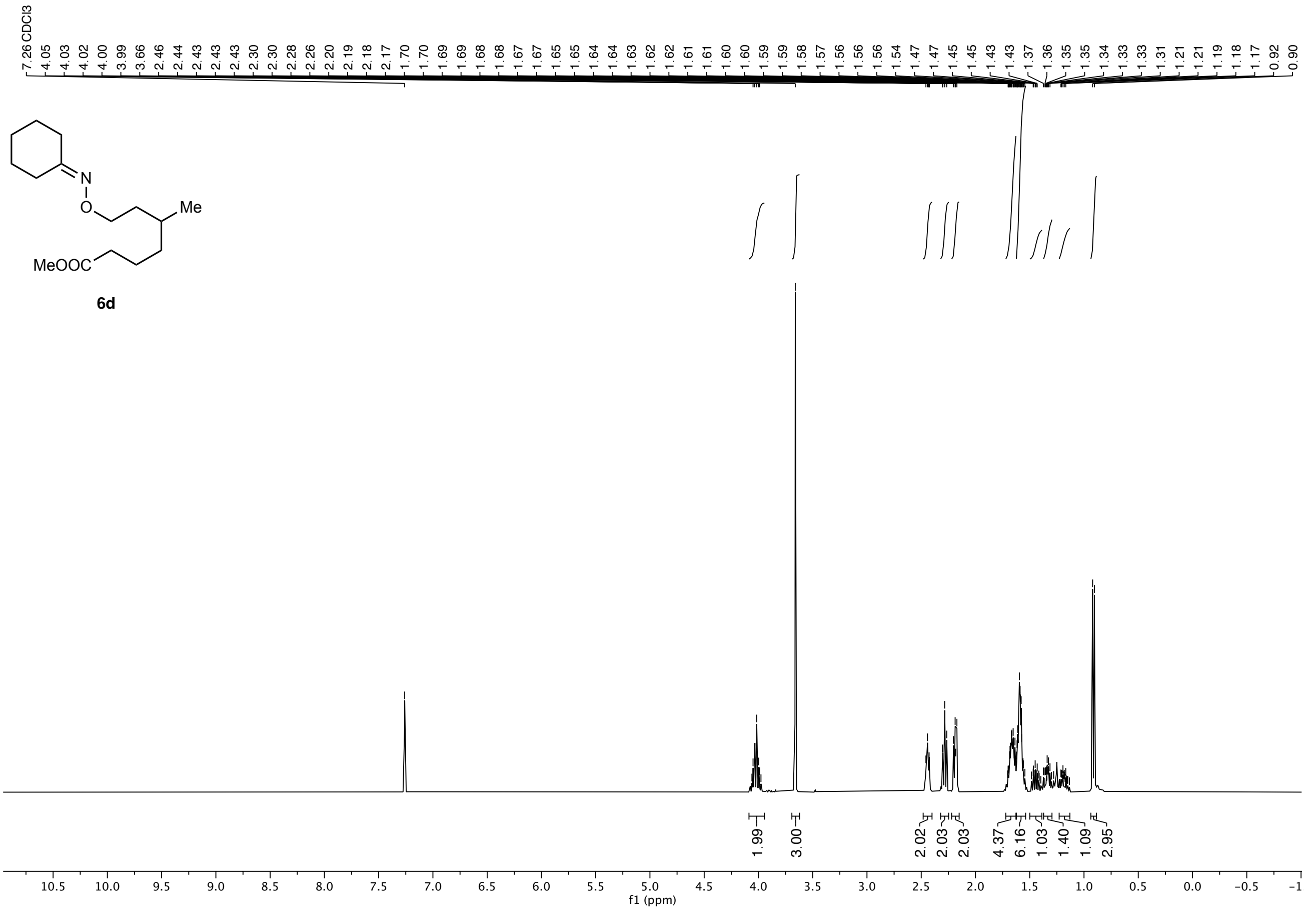
7.26 CDCl3
4.05
4.03
4.02
4.00
3.99
3.66
2.46
2.44
2.43
2.43
2.30
2.30
2.28
2.26
2.20
2.19
2.18
2.17
1.70
1.70
1.69
1.69
1.68
1.68
1.67
1.67
1.65
1.65
1.64
1.64
1.63
1.62
1.62
1.61
1.61
1.60
1.60
1.59
1.59
1.58
1.57
1.56
1.56
1.56
1.54
1.47
1.47
1.45
1.45
1.43
1.43
1.37
1.36
1.35
1.35
1.34
1.33
1.33
1.31
1.21
1.21
1.19
1.18
1.17
0.92
0.90
N
O
Me
MeOOC
6d
1.99
3.00
2.02
2.03
2.03
4.37
6.16
1.03
1.40
1.09
2.95
f1 (ppm)

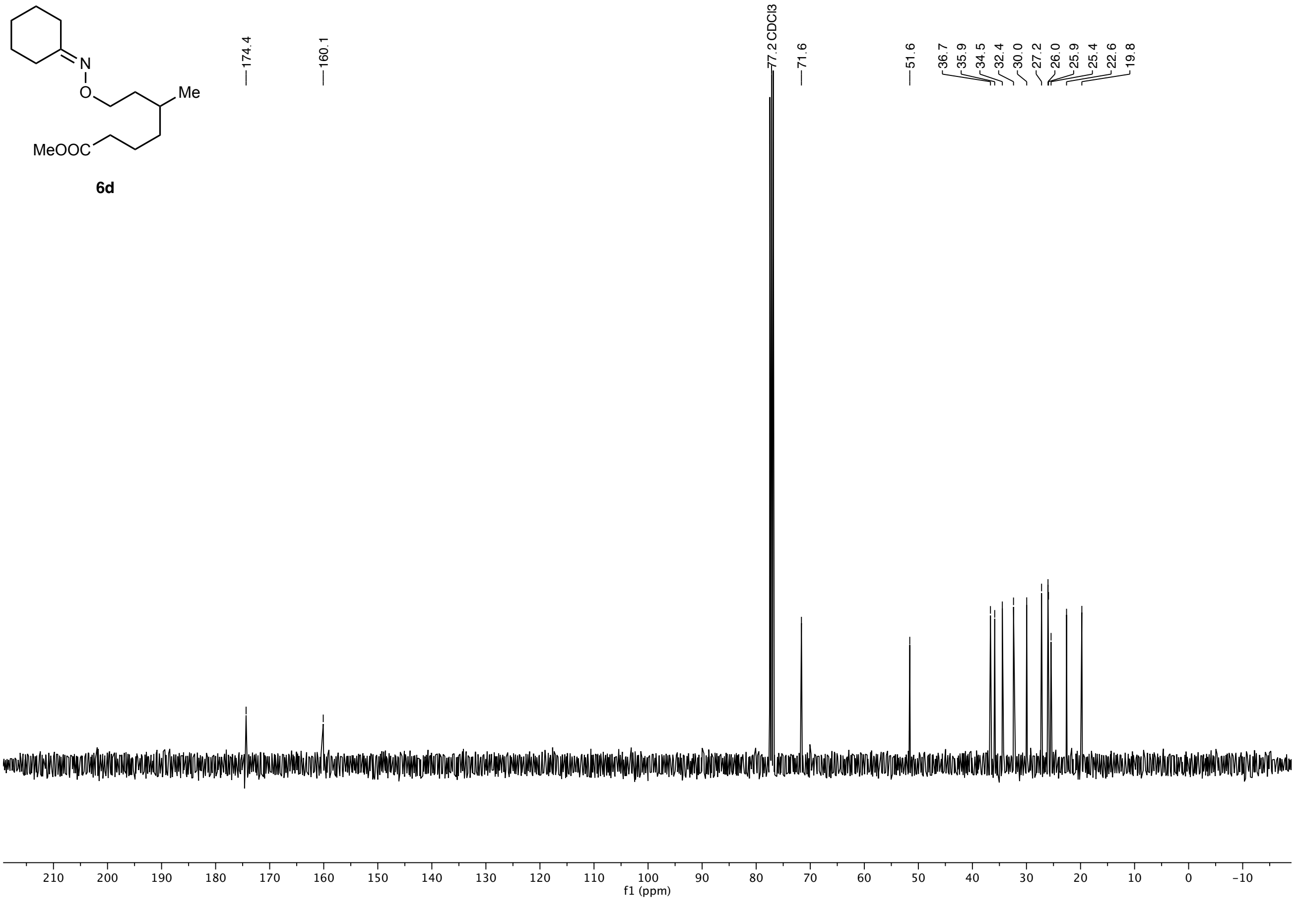
N
O
Me
MeOOC
6d
174.4
160.1
77.2 CDCl3
71.6
51.6
36.7
35.9
34.5
32.4
30.0
27.2
26.0
25.9
25.4
22.6
19.8
f1 (ppm)

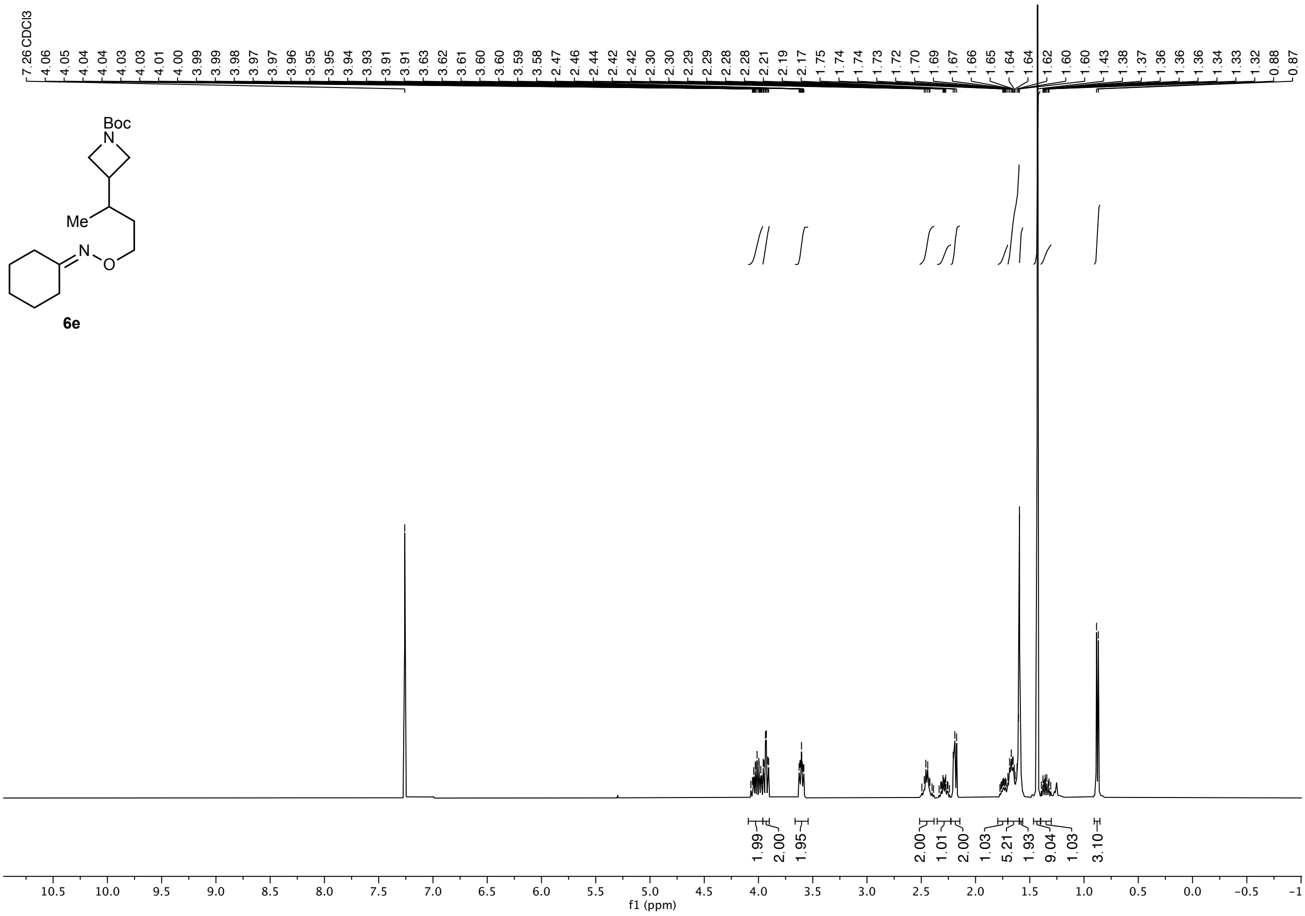
7.26 CDCl3
4.06 4.05 4.04 4.04 4.03 4.03 4.01 4.00 3.99 3.99 3.98 3.97 3.97 3.96 3.95 3.95 3.94 3.93 3.91 3.91 3.63 3.62 3.61 3.60 3.60 3.59 3.58 2.47 2.46 2.44 2.42 2.42 2.30 2.30 2.29 2.29 2.28 2.28 2.21 2.19 2.17 1.75 1.74 1.74 1.73 1.72 1.70 1.69 1.67 1.66 1.65 1.64 1.64 1.62 1.60 1.60 1.43 1.38 1.37 1.36 1.36 1.34 1.33 1.32 0.88 0.87
Boc
N
Me
N
O
6e
1.99 2.00 1.95 2.00 1.01 2.00 1.03 5.21 1.93 9.04 1.03 3.10
10.5 10.0 9.5 9.0 8.5 8.0 7.5 7.0 6.5 6.0 5.5 5.0 4.5 4.0 3.5 3.0 2.5 2.0 1.5 1.0 0.5 0.0 −0.5 −1
f1 (ppm)

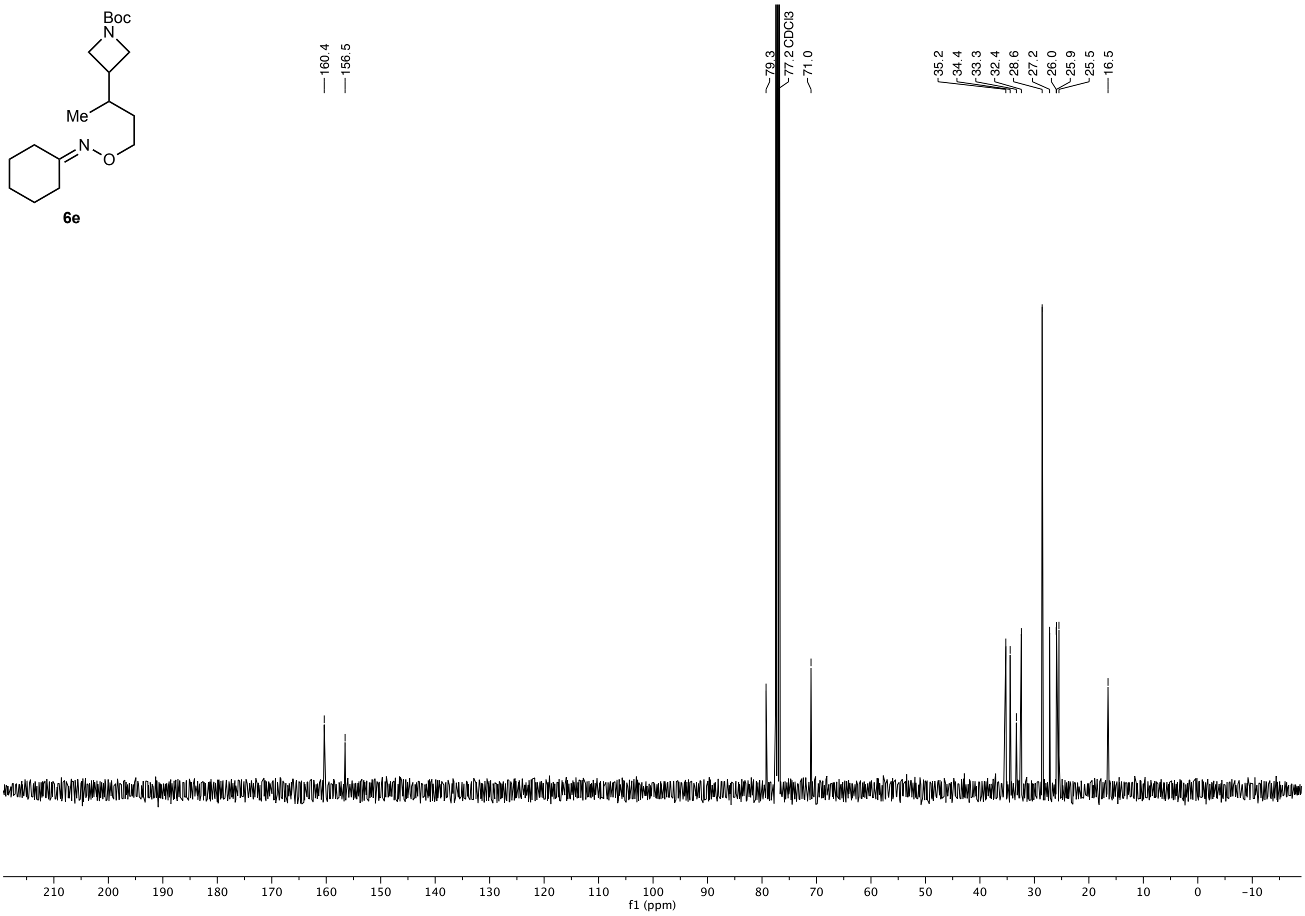
Boc
N
Me
N
O
6e
160.4
156.5
79.3
77.2 CDCl3
71.0
35.2 34.4 33.3 32.4 28.6 27.2 26.0 25.9 25.5 16.5
210 200 190 180 170 160 150 140 130 120 110 100 90 80 70 60 50 40 30 20 10 0 −10
f1 (ppm)

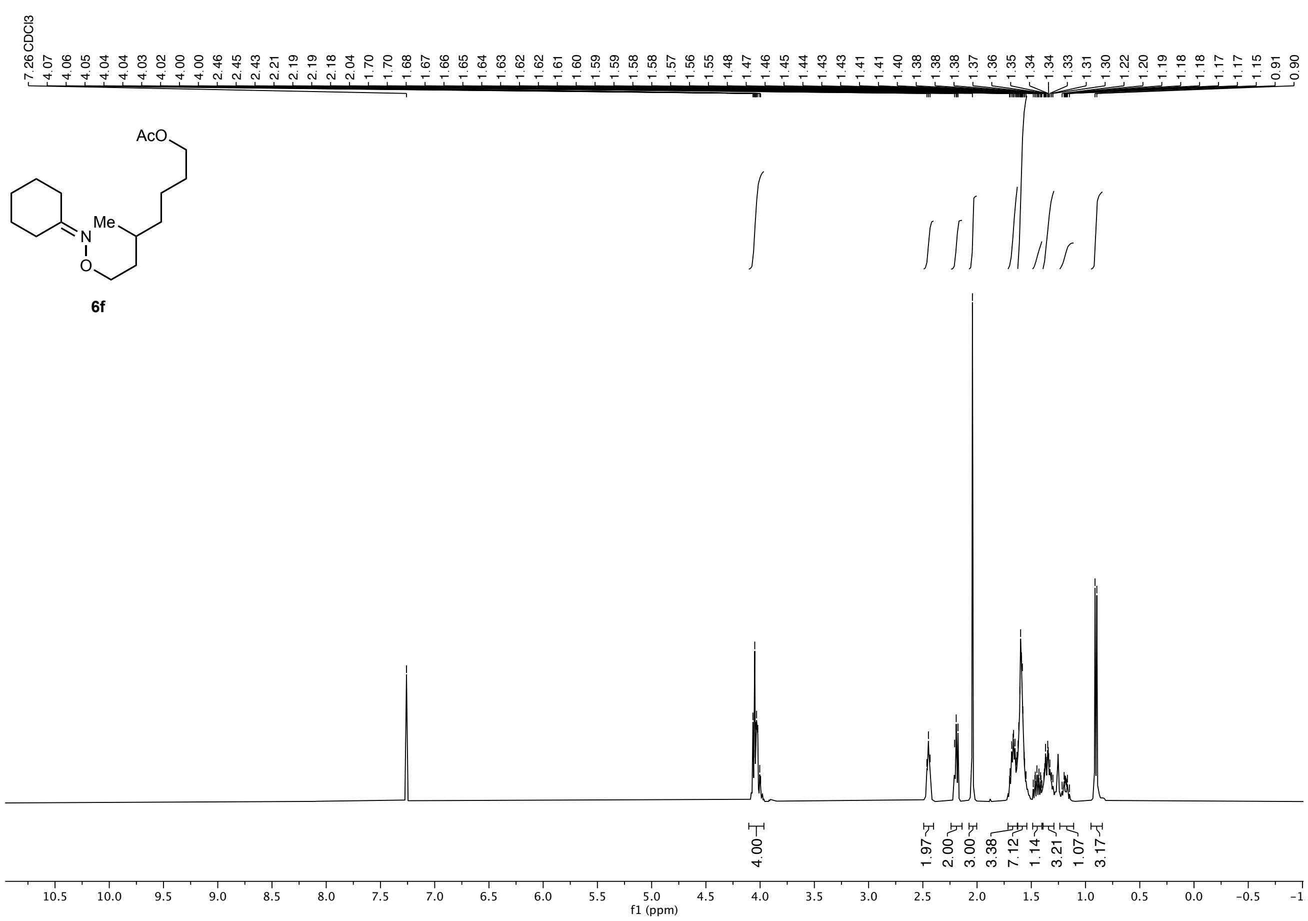

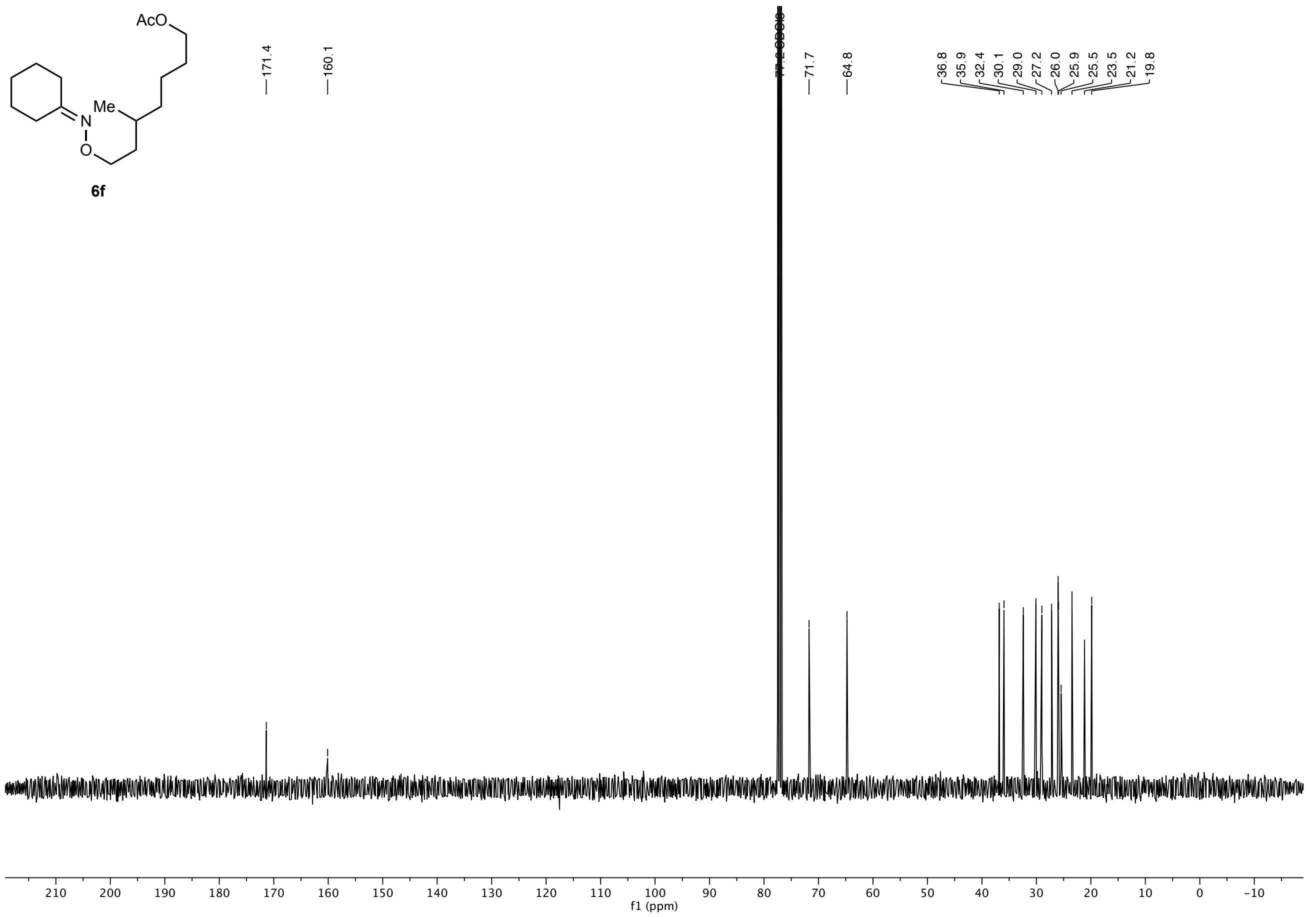

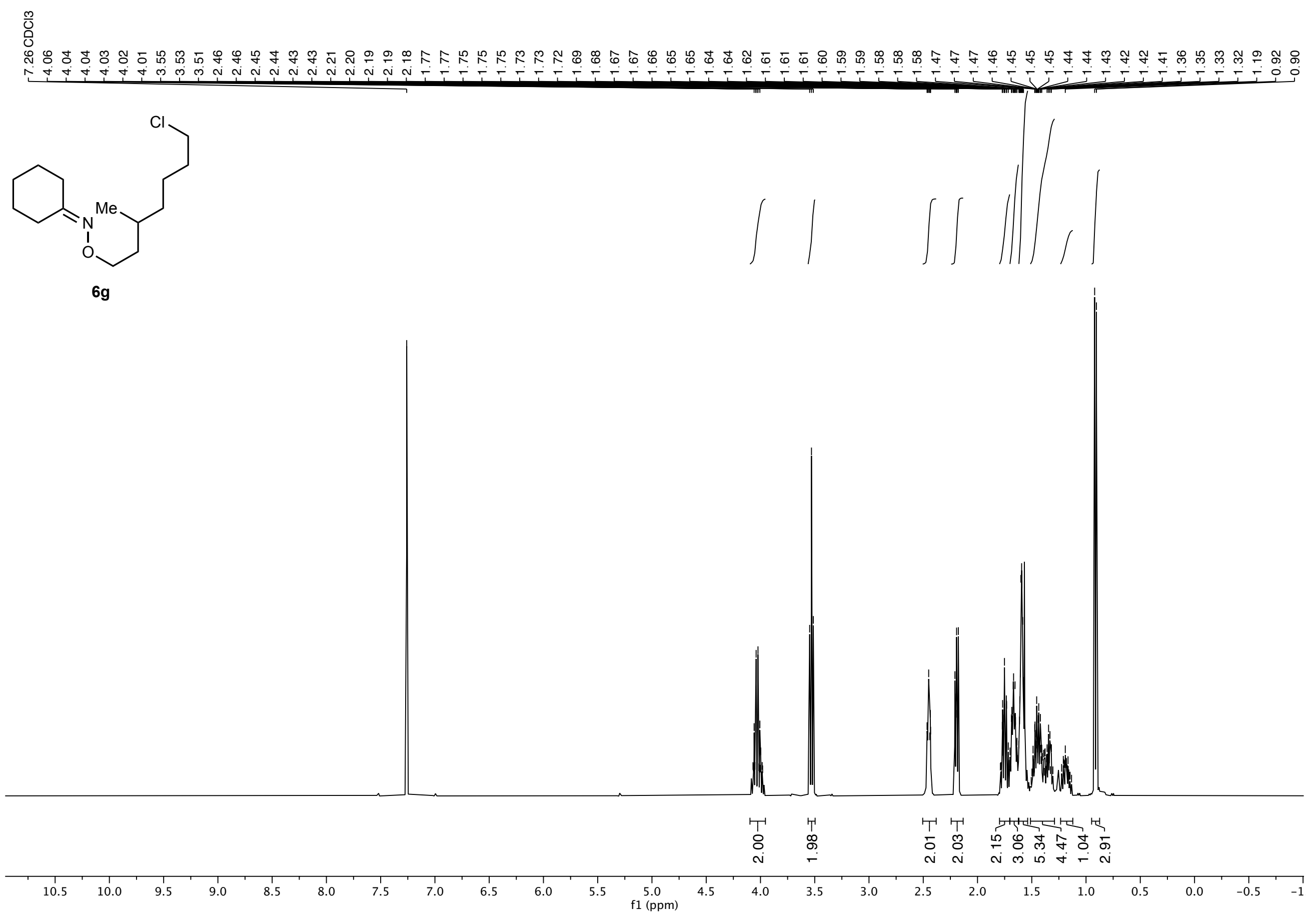
7.26 CDCl3
4.06 4.04 4.04 4.03 4.02 4.01 3.55 3.53 3.51 2.46 2.46 2.45 2.44 2.44 2.43 2.43 2.21 2.20 2.19 2.19 2.18 1.77 1.77 1.75 1.75 1.75 1.73 1.73 1.72 1.69 1.68 1.67 1.67 1.66 1.65 1.65 1.64 1.64 1.62 1.61 1.61 1.61 1.60 1.59 1.59 1.58 1.58 1.58 1.47 1.47 1.47 1.46 1.45 1.45 1.45 1.45 1.44 1.44 1.43 1.43 1.42 1.42 1.41 1.36 1.35 1.33 1.32 1.19 0.92 0.90
Cl
Me
N
O
6g
2.00
1.98
2.01
2.03
2.15
3.06
5.34
4.47
1.04
2.91
f1 (ppm)

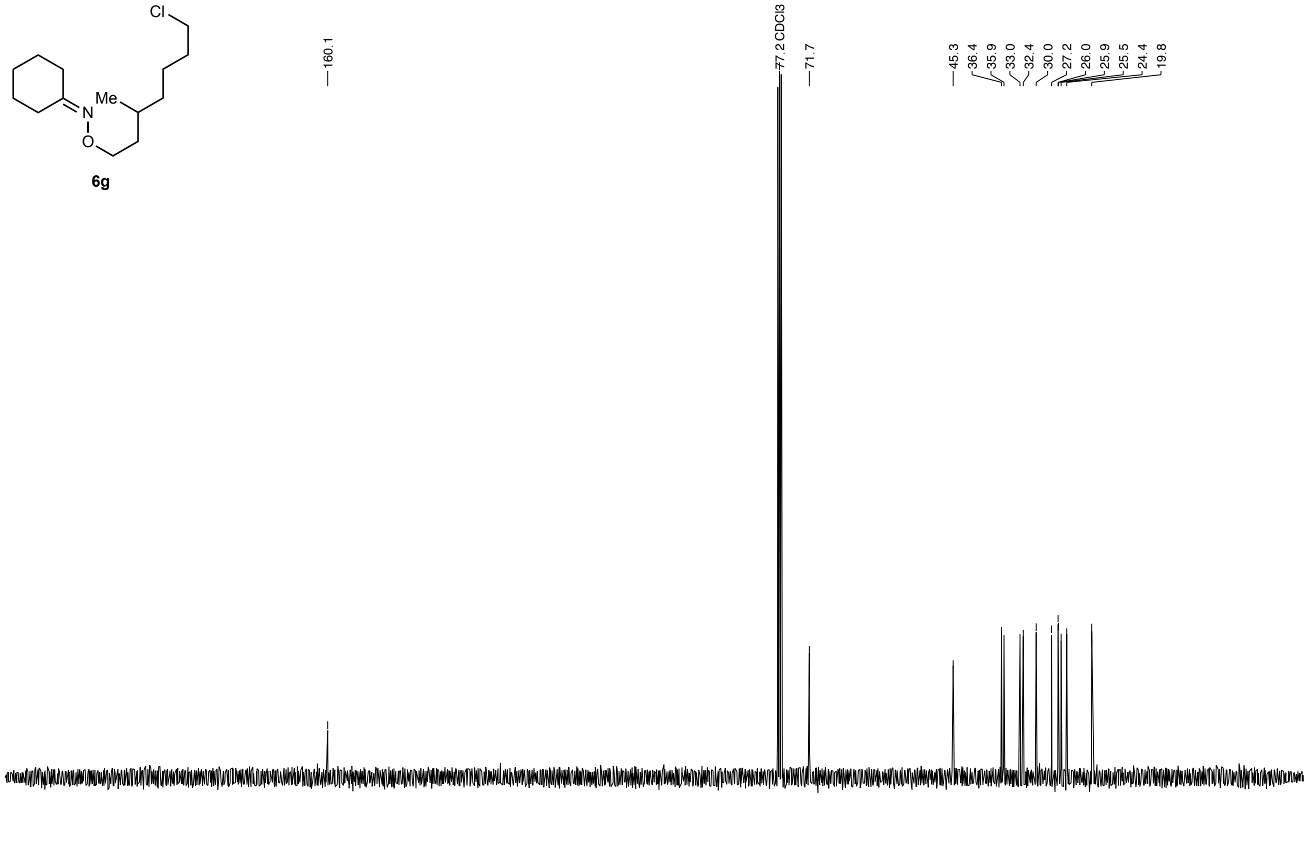
160.1
77.2 CDCl3
71.7
45.3 36.4 35.9 33.0 32.4 30.0 27.2 26.0 25.9 25.5 24.4 19.8
Cl
Me
N
O
6g
f1 (ppm)

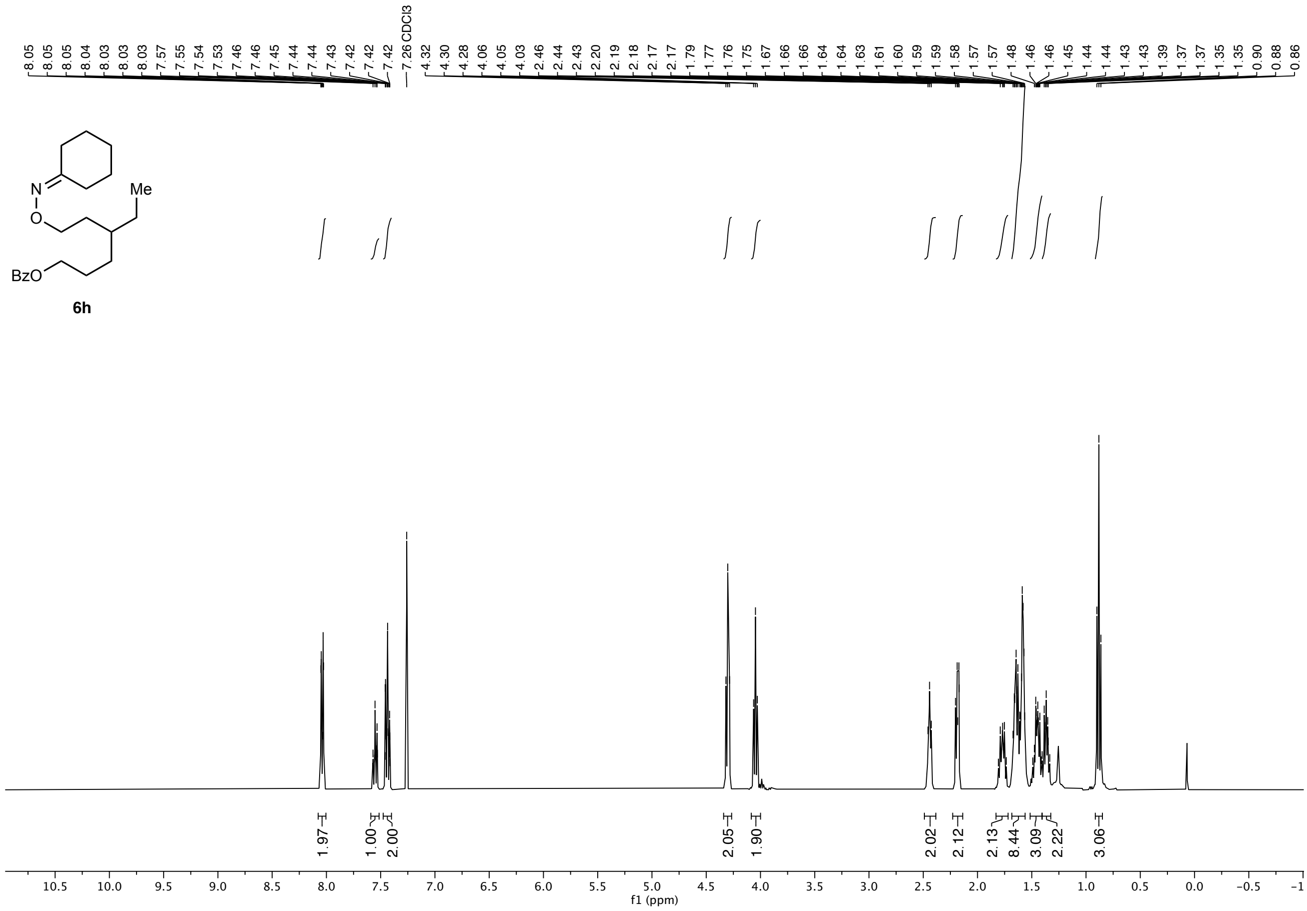

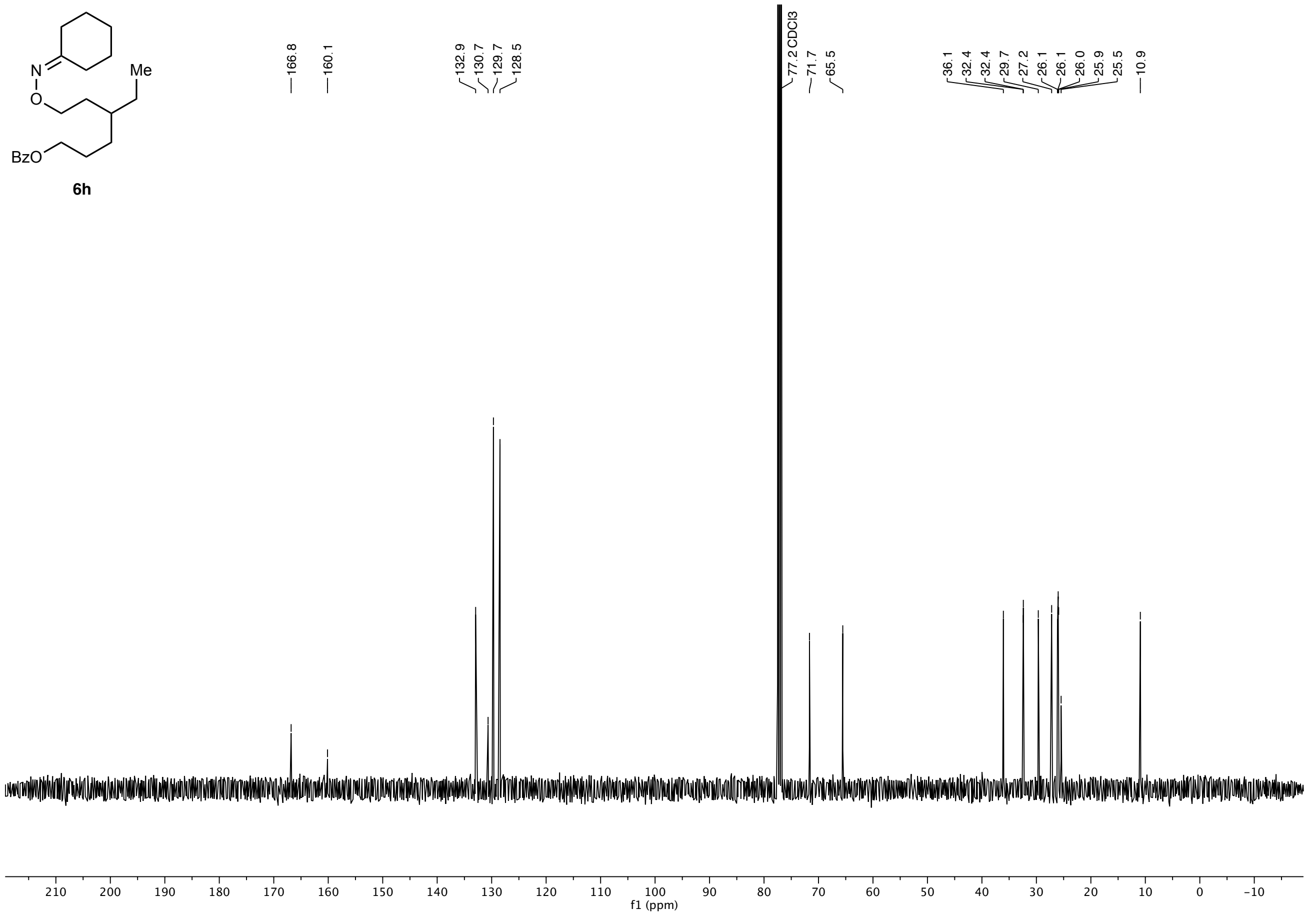

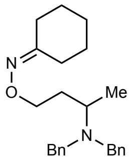
N
O
Me
N
Bn
Bn

7a

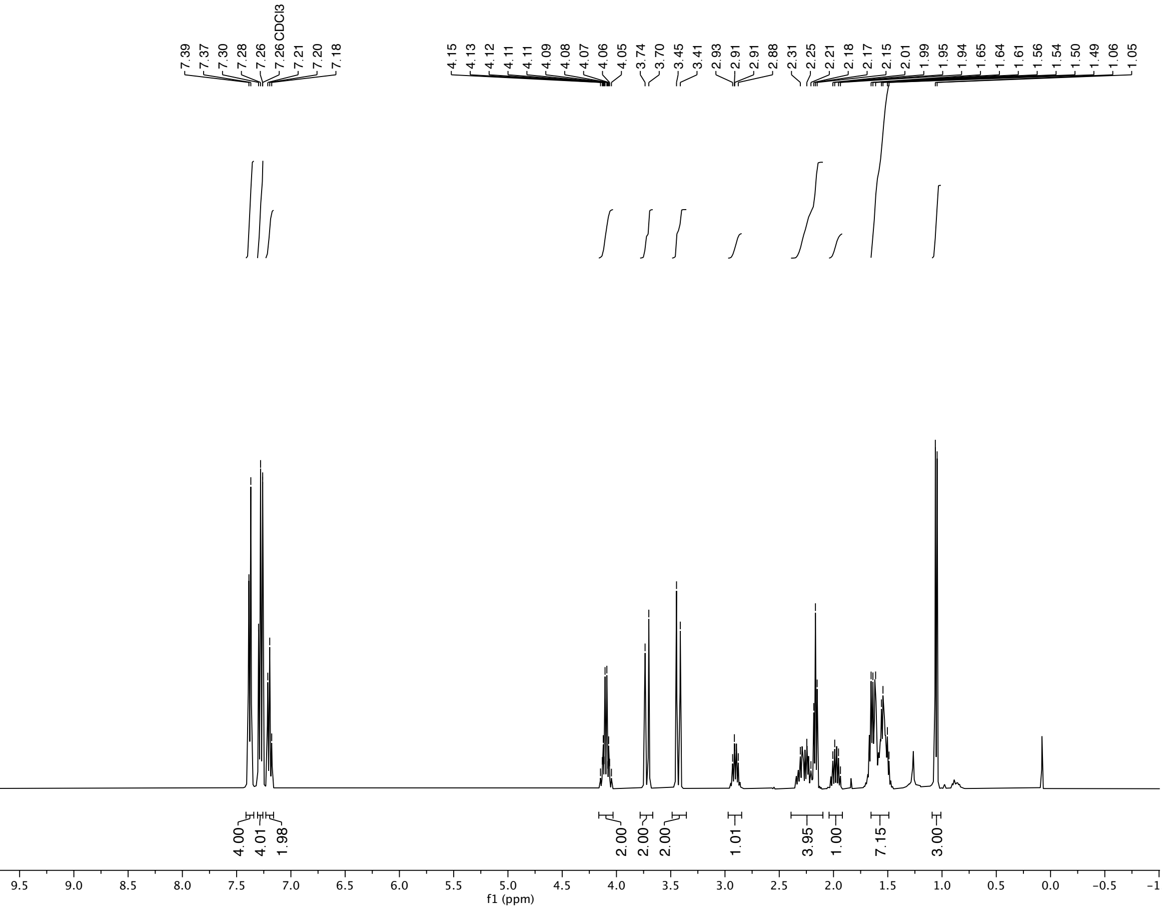
7.39 7.37 7.30 7.28 7.26 7.26 CDCl3 7.21 7.20 7.18
4.15 4.13 4.12 4.11 4.11 4.09 4.08 4.07 4.06 4.05 3.74 3.70 3.45 3.41 2.93 2.91 2.91 2.88 2.31 2.25 2.21 2.18 2.17 2.15 2.01 1.99 1.95 1.94 1.65 1.64 1.61 1.56 1.54 1.50 1.49 1.06 1.05
4.00 4.01 1.98 2.00 2.00 2.00 1.01 3.95 1.00 7.15 3.00
10.5 10.0 9.5 9.0 8.5 8.0 7.5 7.0 6.5 6.0 5.5 5.0 4.5 4.0 3.5 3.0 2.5 2.0 1.5 1.0 0.5 0.0 −0.5 −1
f1 (ppm)

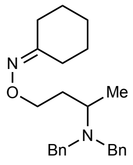
N
O
Me
N
Bn
Bn

7a

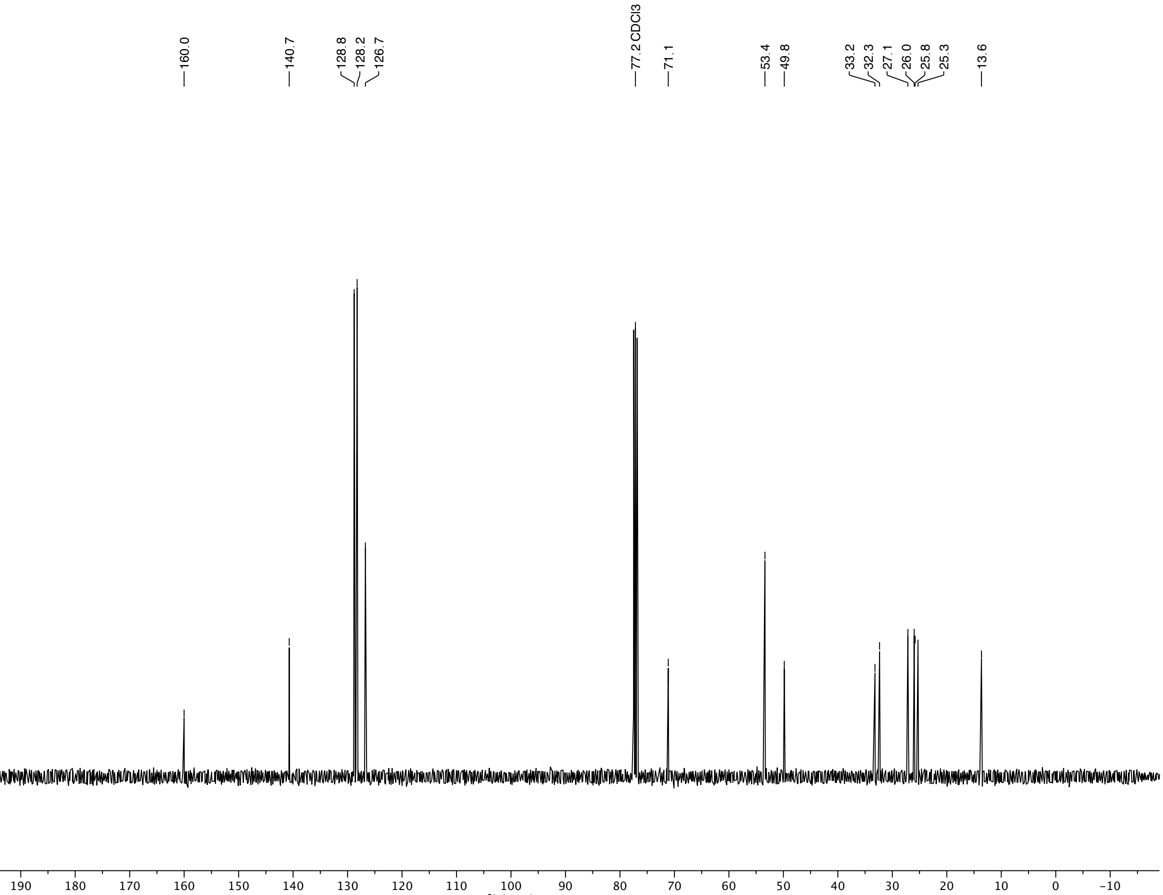
160.0
140.7
128.8 128.2 126.7
77.2 CDCl3
71.1
53.4 49.8
33.2 32.3 27.1 26.0 25.8 25.3
13.6
210 200 190 180 170 160 150 140 130 120 110 100 90 80 70 60 50 40 30 20 10 0 −10
f1 (ppm)

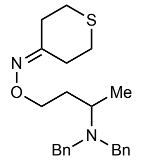
S
N
O
Me
N
Bn
Bn

7b

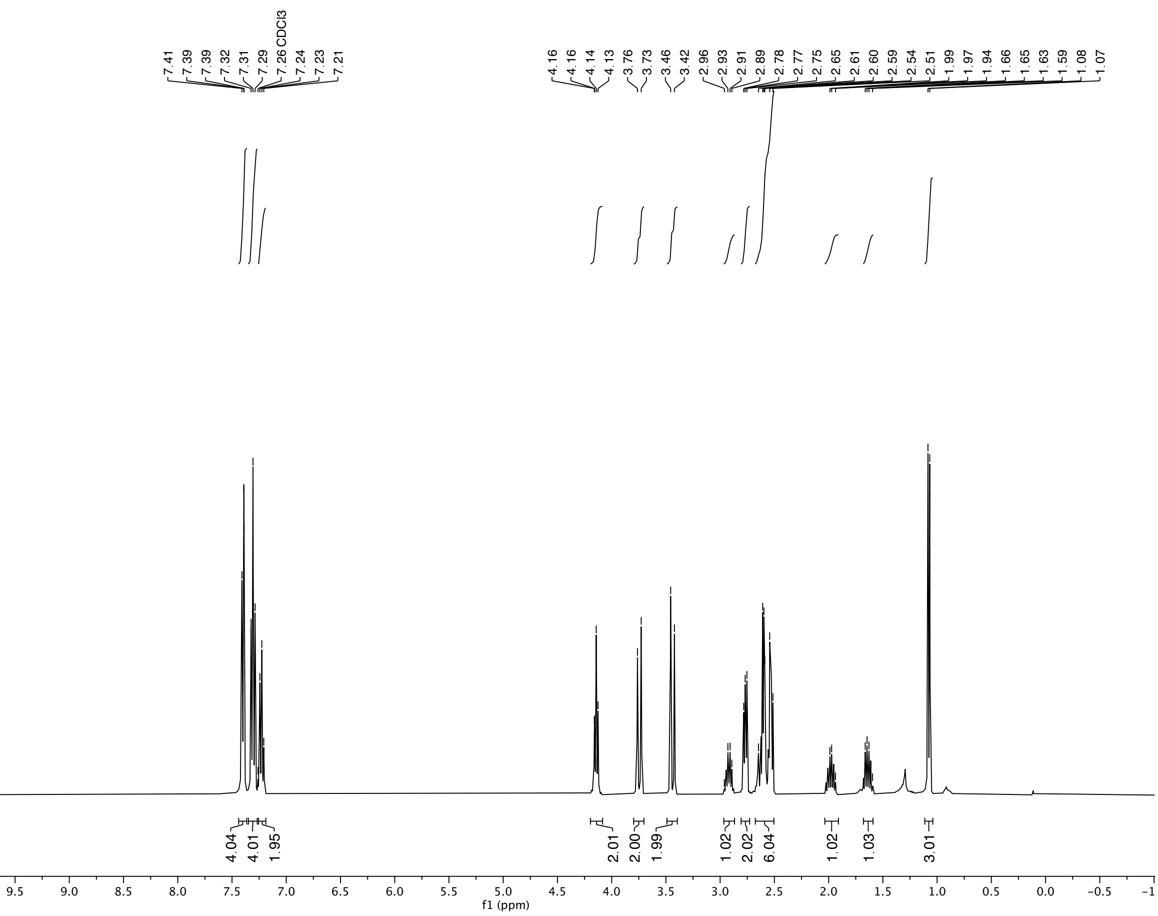
7.41 7.39 7.39 7.32 7.31 7.29 7.26 CDCl3 7.24 7.23 7.21
4.16 4.16 4.14 4.13 3.76 3.73 3.46 3.42 2.96 2.93 2.91 2.89 2.78 2.77 2.75 2.65 2.61 2.60 2.59 2.54 2.51 1.99 1.97 1.94 1.66 1.65 1.63 1.59 1.08 1.07
4.04 4.01 1.95 2.01 2.00 1.99 1.02 2.02 6.04 1.02 1.03 3.01
10.5 10.0 9.5 9.0 8.5 8.0 7.5 7.0 6.5 6.0 5.5 5.0 4.5 4.0 3.5 3.0 2.5 2.0 1.5 1.0 0.5 0.0 −0.5 −1
f1 (ppm)

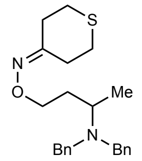
S
N
O
Me
N
Bn
Bn

7b

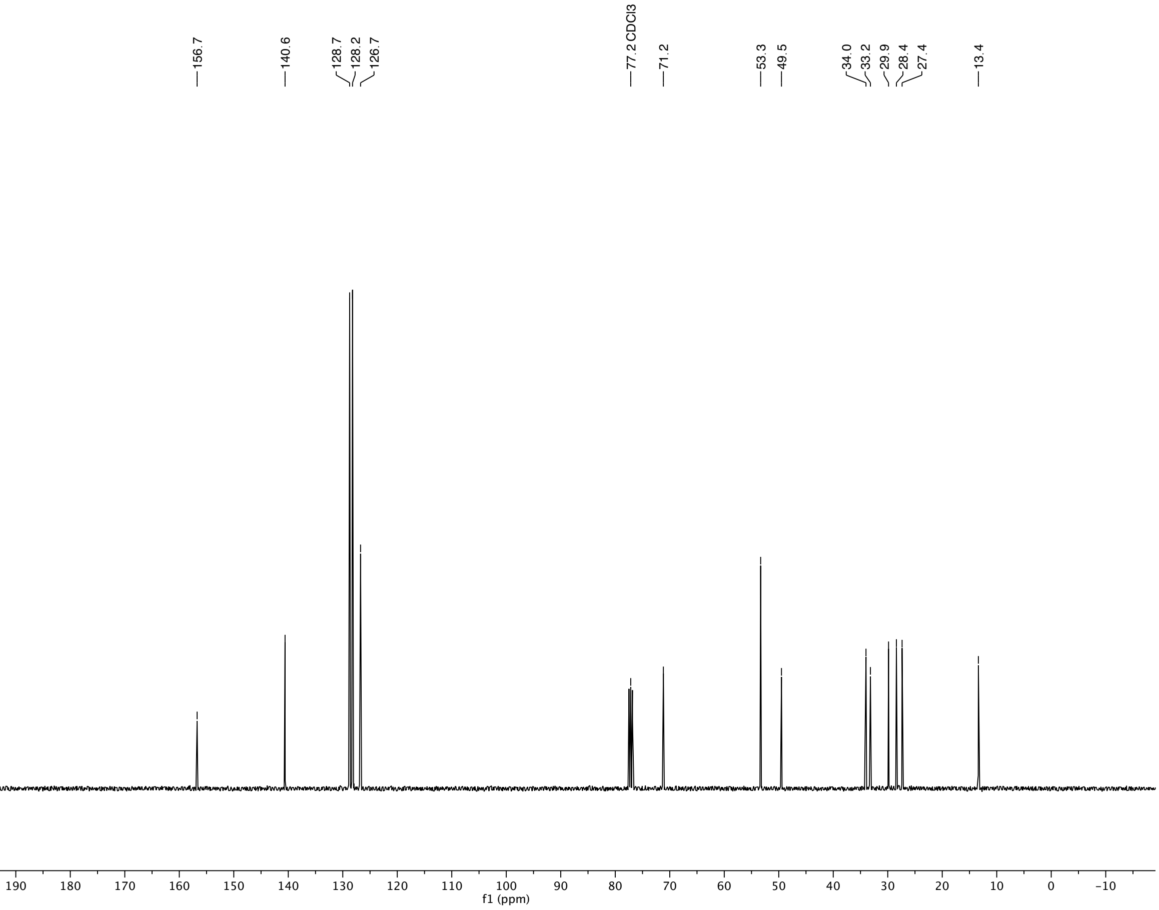
156.7
140.6
128.7 128.2 126.7
77.2 CDCl3
71.2
53.3 49.5
34.0 33.2 29.9 28.4 27.4
13.4
210 200 190 180 170 160 150 140 130 120 110 100 90 80 70 60 50 40 30 20 10 0 −10
f1 (ppm)

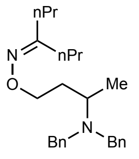
nPr
N
nPr
O
Me
N
Bn
Bn

7c

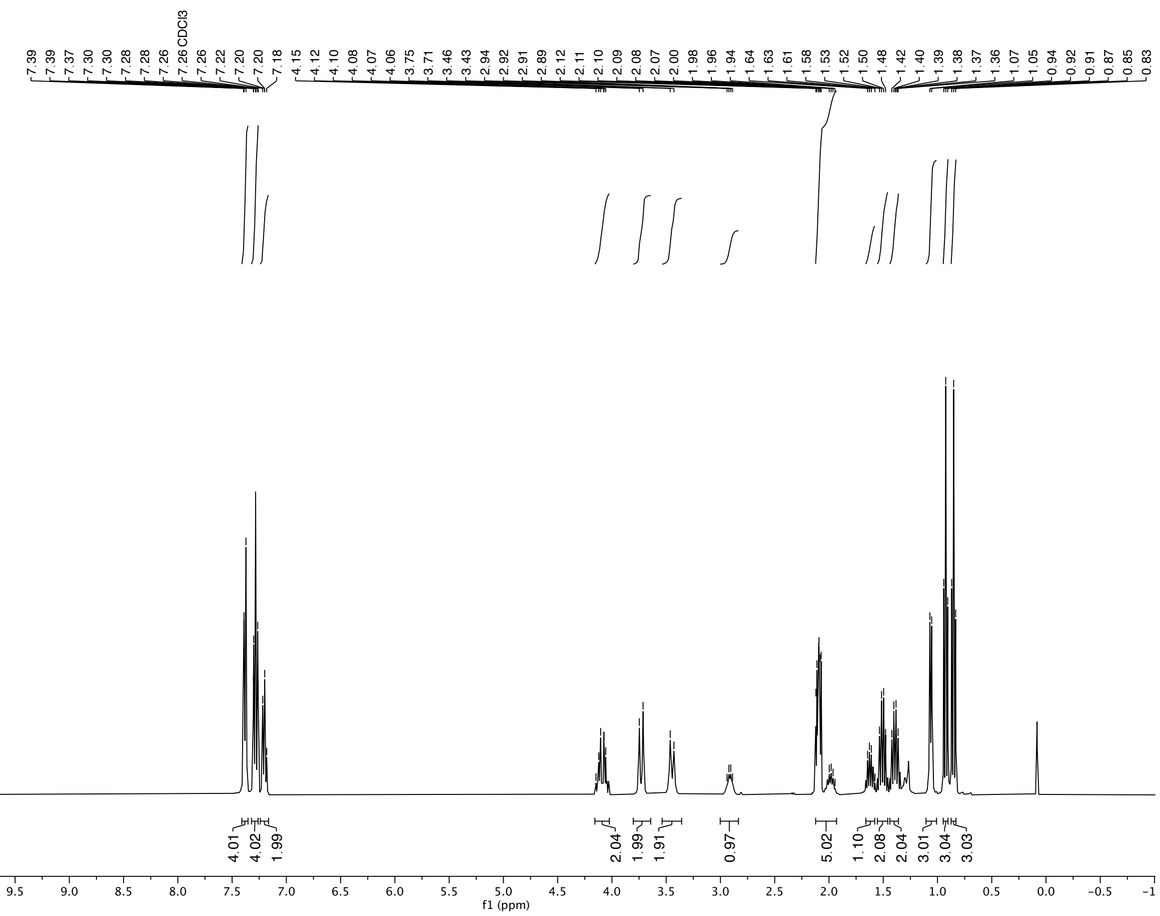
7.39 7.39 7.37 7.30 7.30 7.28 7.28 7.26 7.26 CDCl3 7.26 7.22 7.20 7.20 7.18 4.15 4.12 4.10 4.08 4.07 4.06 3.75 3.71 3.46 3.43 2.94 2.92 2.91 2.89 2.12 2.11 2.10 2.09 2.08 2.07 2.00 1.98 1.96 1.94 1.64 1.63 1.61 1.58 1.53 1.52 1.50 1.48 1.42 1.40 1.39 1.38 1.37 1.36 1.07 1.05 0.94 0.92 0.91 0.87 0.85 0.83
4.01 4.02 1.99 2.04 1.99 1.91 0.97 5.02 1.10 2.08 2.04 3.01 3.04 3.03
10.5 10.0 9.5 9.0 8.5 8.0 7.5 7.0 6.5 6.0 5.5 5.0 4.5 4.0 3.5 3.0 2.5 2.0 1.5 1.0 0.5 0.0 -0.5 -1
f1 (ppm)

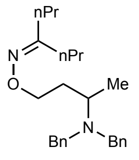
nPr
N
nPr
O
Me
N
Bn
Bn

7c

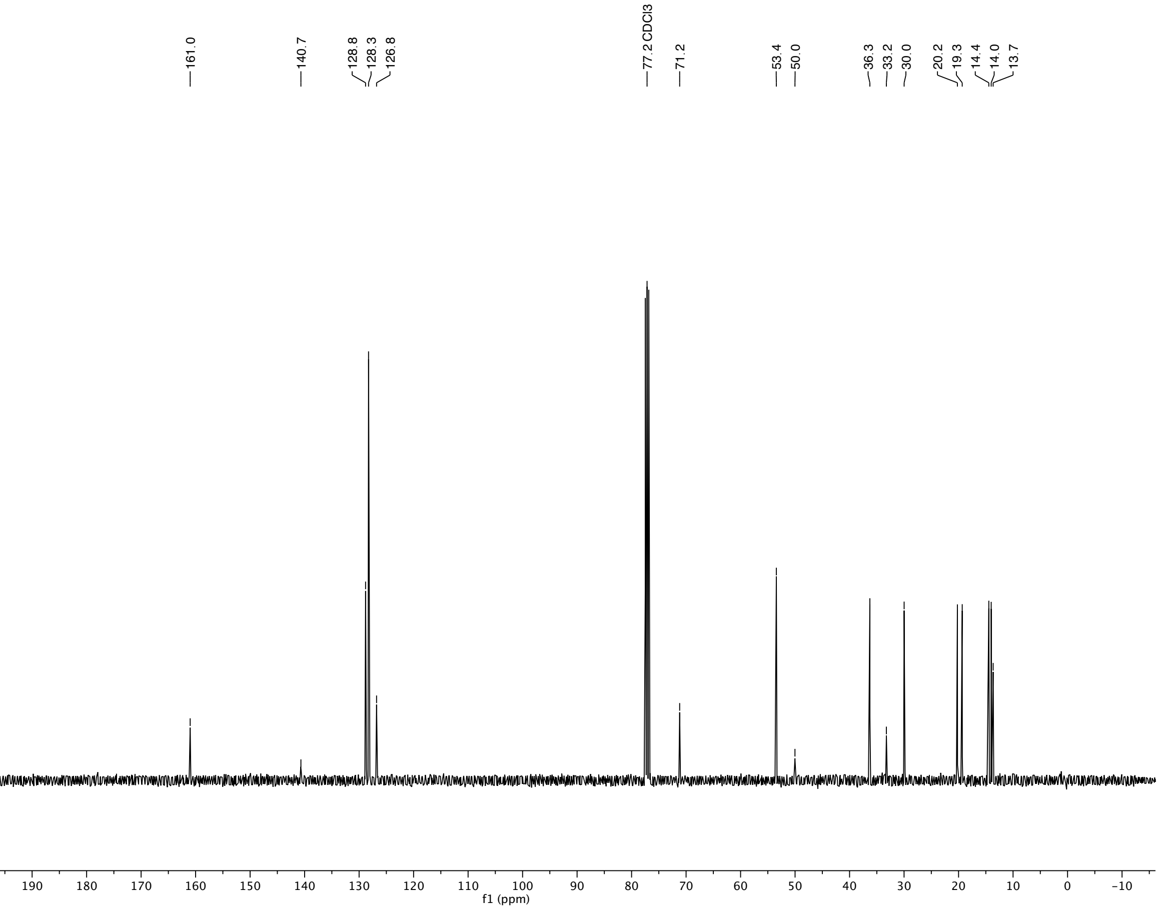
161.0 140.7 128.8 128.3 126.8 77.2 CDCl3 71.2 53.4 50.0 36.3 33.2 30.0 20.2 19.3 14.4 14.0 13.7
220 210 200 190 180 170 160 150 140 130 120 110 100 90 80 70 60 50 40 30 20 10 0 -10
f1 (ppm)

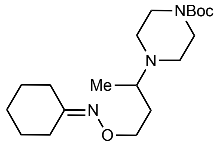

7d

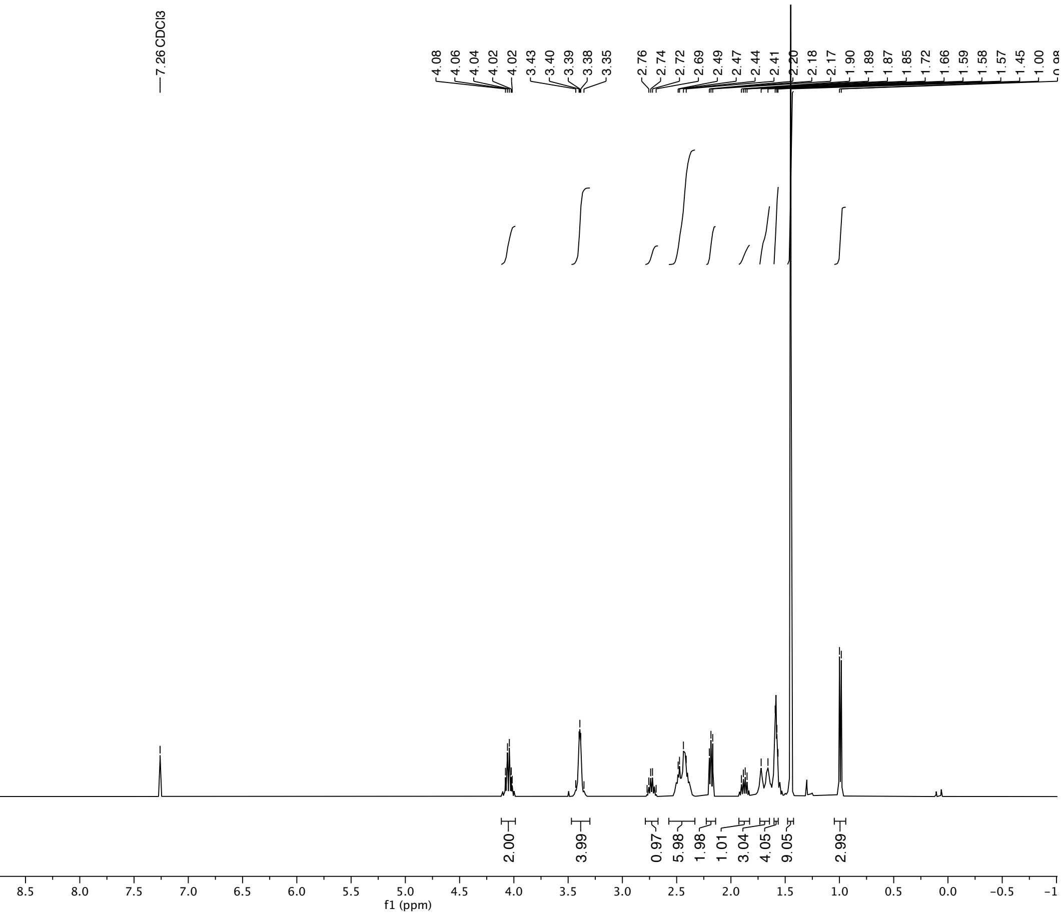

7.26 CDCl3
4.08
4.06
4.04
4.02
4.02
3.43
3.40
3.39
3.38
3.35
2.76
2.74
2.72
2.69
2.49
2.47
2.44
2.41
2.20
2.18
2.17
1.90
1.89
1.87
1.85
1.72
1.66
1.59
1.58
1.57
1.45
1.00
2.00
3.99
0.97
5.98
1.98
1.01
3.04
4.05
9.05
2.99
f1 (ppm)

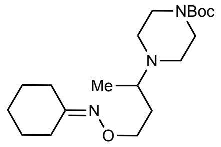

7d

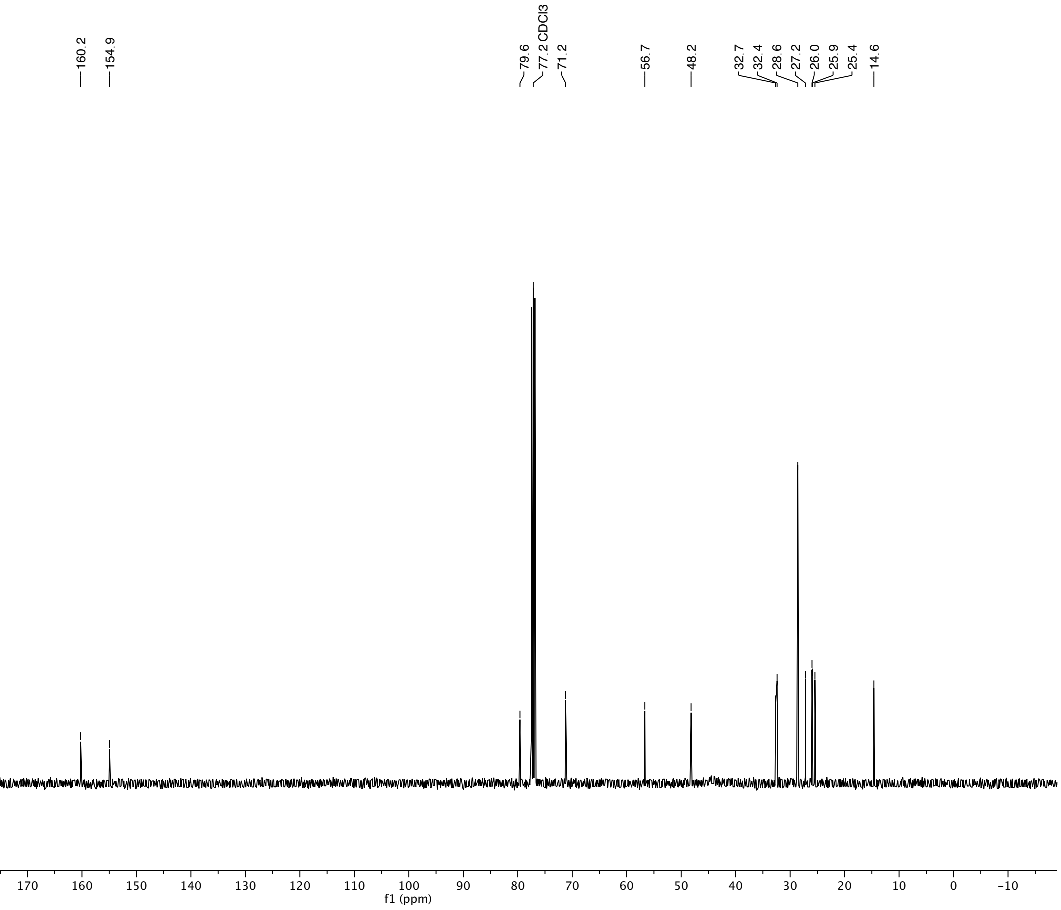

160.2
154.9
79.6
77.2 CDCl3
71.2
56.7
48.2
32.7
32.4
28.6
27.2
26.0
25.9
25.4
14.6
f1 (ppm)

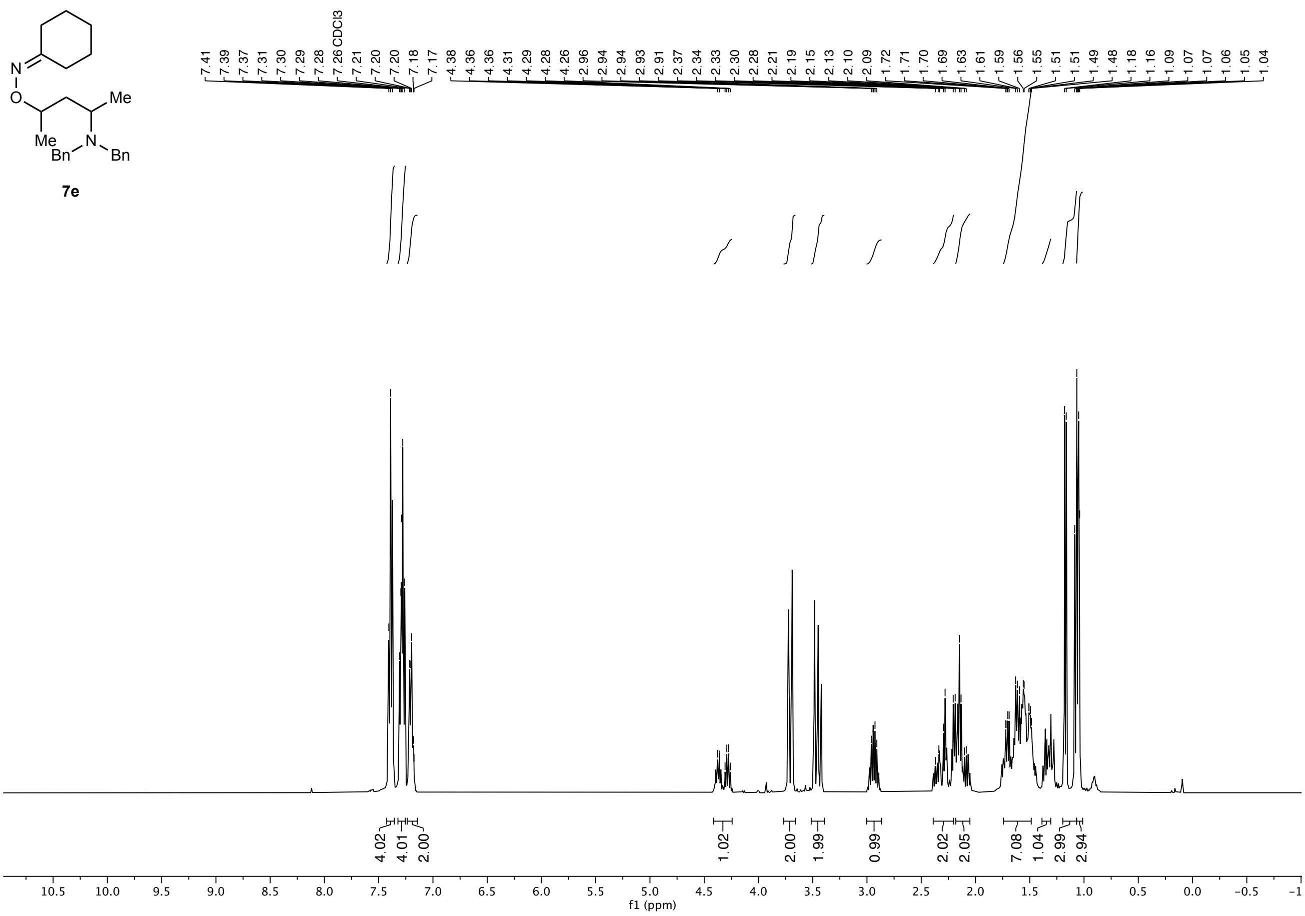
N
O
Me
Me
Bn
N
Bn
7.41 7.39 7.37 7.31 7.30 7.29 7.28 7.26 CDCl3 7.21 7.20 7.20 7.18 7.17
4.38 4.36 4.36 4.31 4.29 4.28 4.26 2.96 2.94 2.94 2.93 2.91 2.37 2.34 2.33 2.30 2.28 2.21 2.19 2.15 2.13 2.10 2.09 1.72 1.71 1.70 1.69 1.63 1.61 1.59 1.56 1.55 1.51 1.51 1.49 1.48 1.18 1.16 1.09 1.07 1.07 1.06 1.05 1.04
4.02 4.01 2.00 1.02 2.00 1.99 0.99 2.02 2.05 7.08 1.04 2.99 2.94
f1 (ppm)

7e

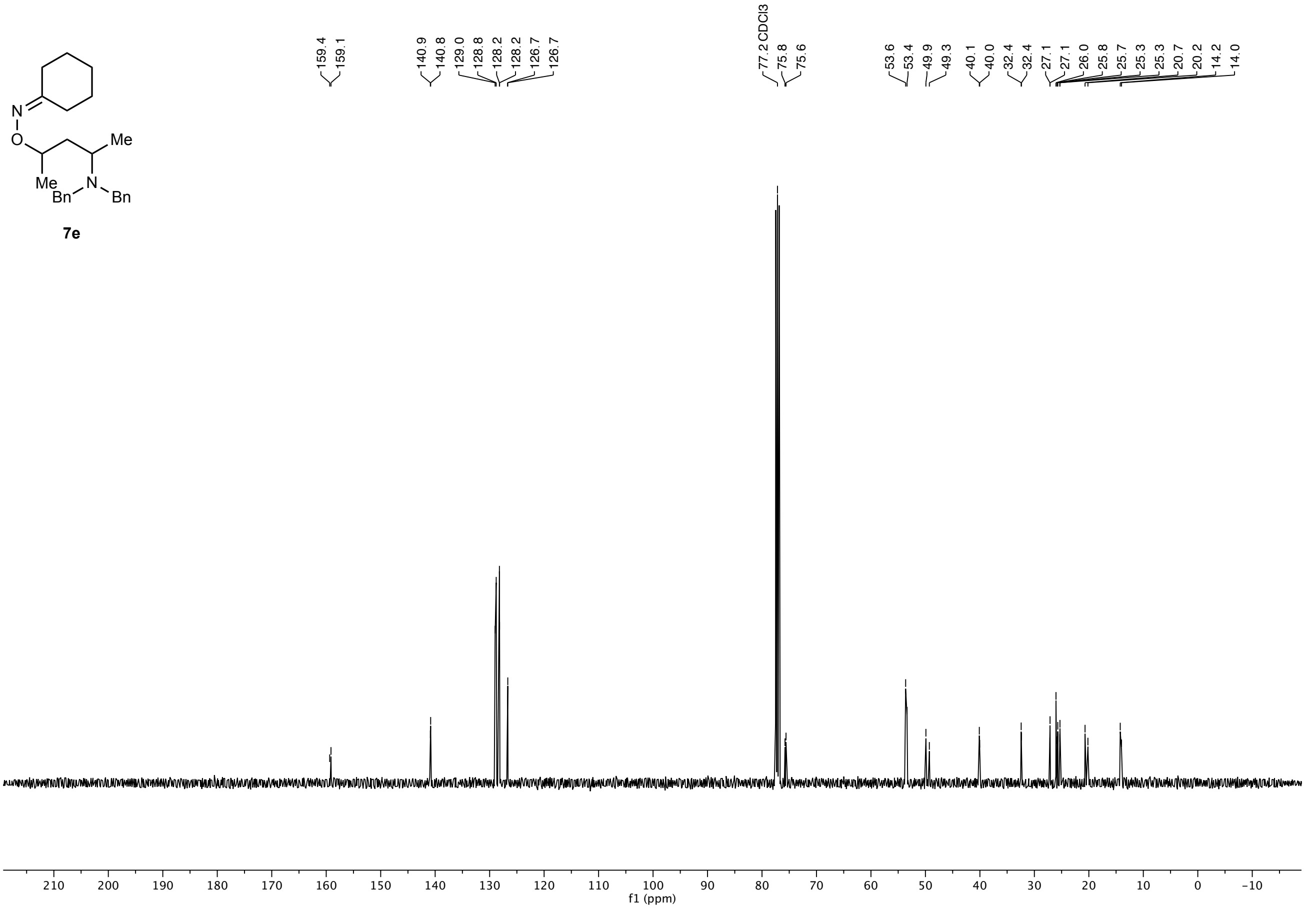
N
O
Me
Me
Bn
N
Bn
159.4 159.1 140.9 140.8 129.0 128.8 128.2 128.2 126.7 126.7
77.2 CDCl3 75.8 75.6
53.6 53.4 49.9 49.3 40.1 40.0 32.4 32.4 27.1 27.1 26.0 25.8 25.7 25.3 25.3 20.7 20.2 14.2 14.0
f1 (ppm)

7e

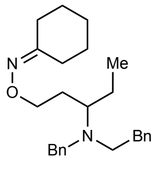
Me
N
O
Bn
N
Bn

7f

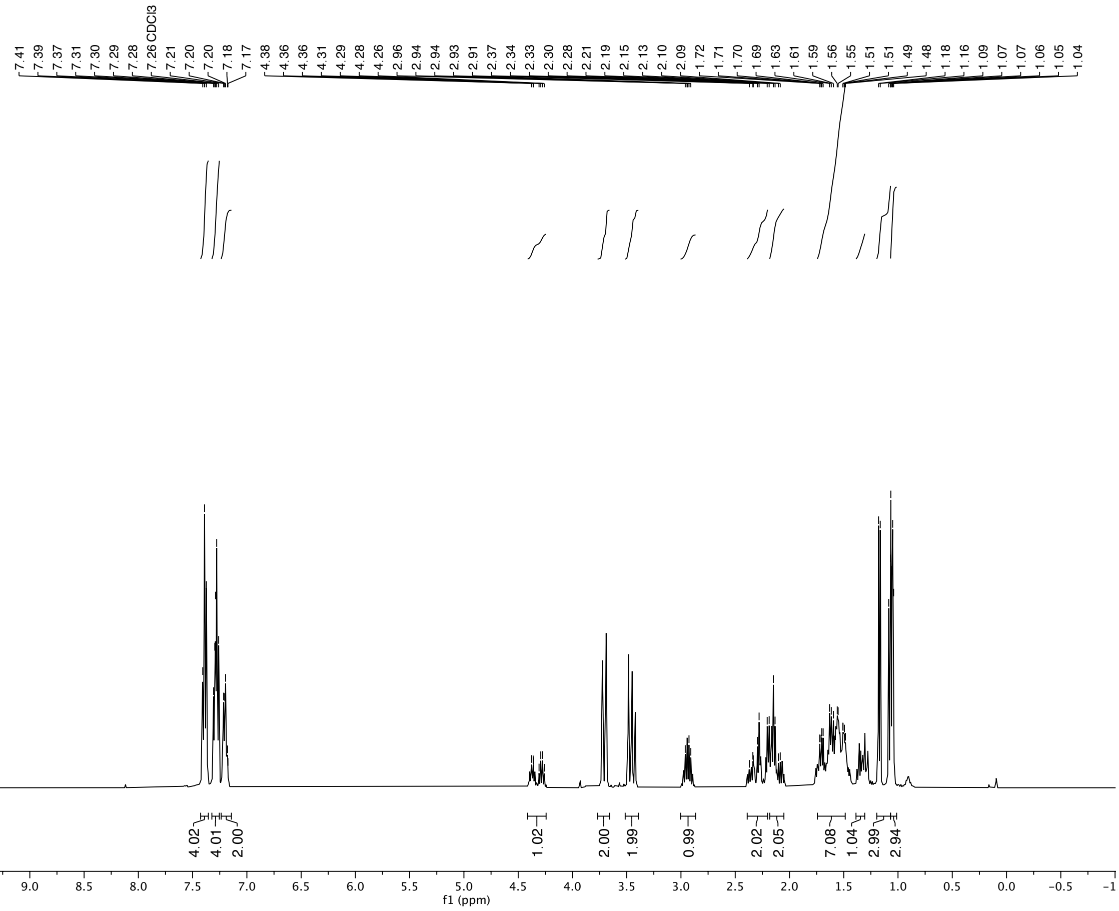
7.41 7.39 7.37 7.31 7.30 7.29 7.28 7.26 CDCl3 7.21 7.20 7.18 7.17 4.38 4.36 4.36 4.31 4.29 4.28 4.26 2.96 2.94 2.94 2.93 2.91 2.37 2.34 2.33 2.30 2.28 2.21 2.19 2.15 2.13 2.10 2.09 1.72 1.71 1.70 1.69 1.63 1.61 1.59 1.56 1.55 1.51 1.51 1.49 1.48 1.18 1.16 1.09 1.07 1.07 1.06 1.05 1.04
4.02 4.01 2.00 1.02 2.00 1.99 0.99 2.02 2.05 7.08 1.04 2.99 2.94
f1 (ppm)

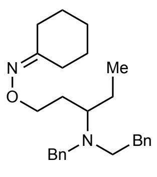
Me
N
O
Bn
N
Bn

7f

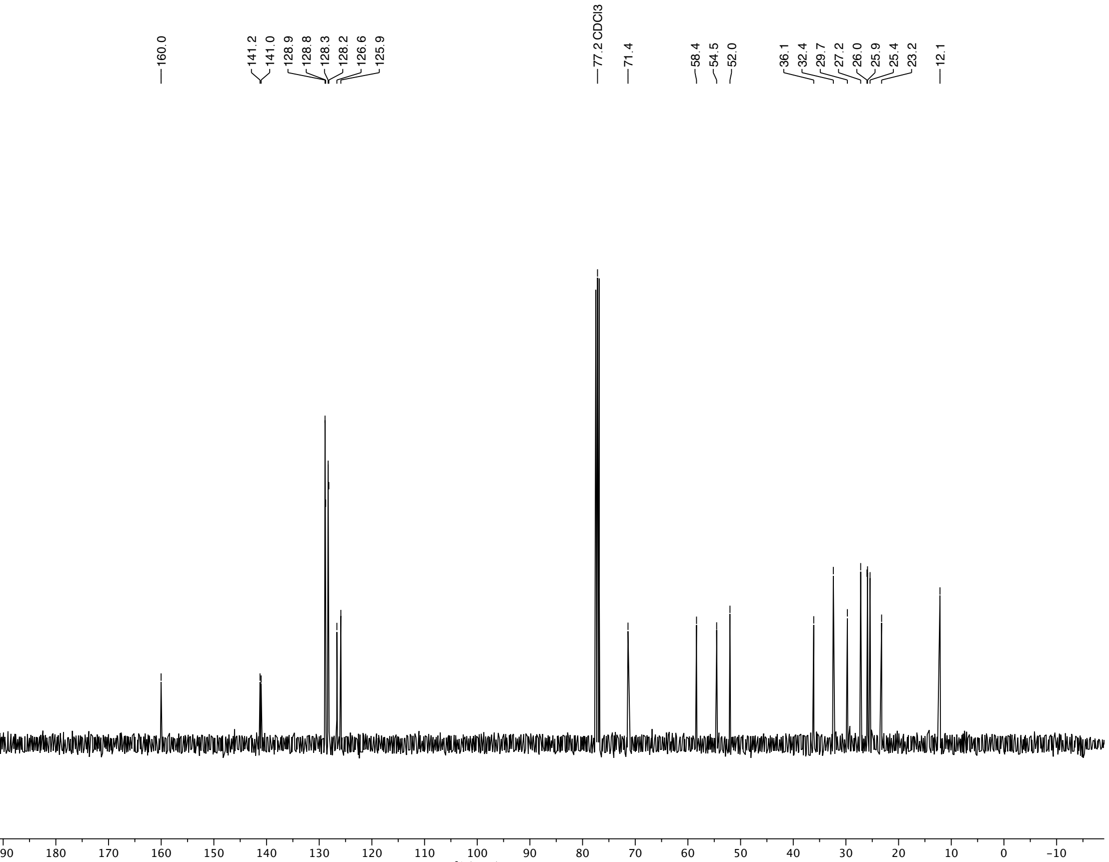
160.0 141.2 141.0 128.9 128.8 128.3 128.2 126.6 125.9 77.2 CDCl3 71.4 58.4 54.5 52.0 36.1 32.4 29.7 27.2 26.0 25.9 25.4 23.2 12.1
f1 (ppm)

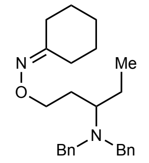
N
O
Me
Bn
N
Bn

7g

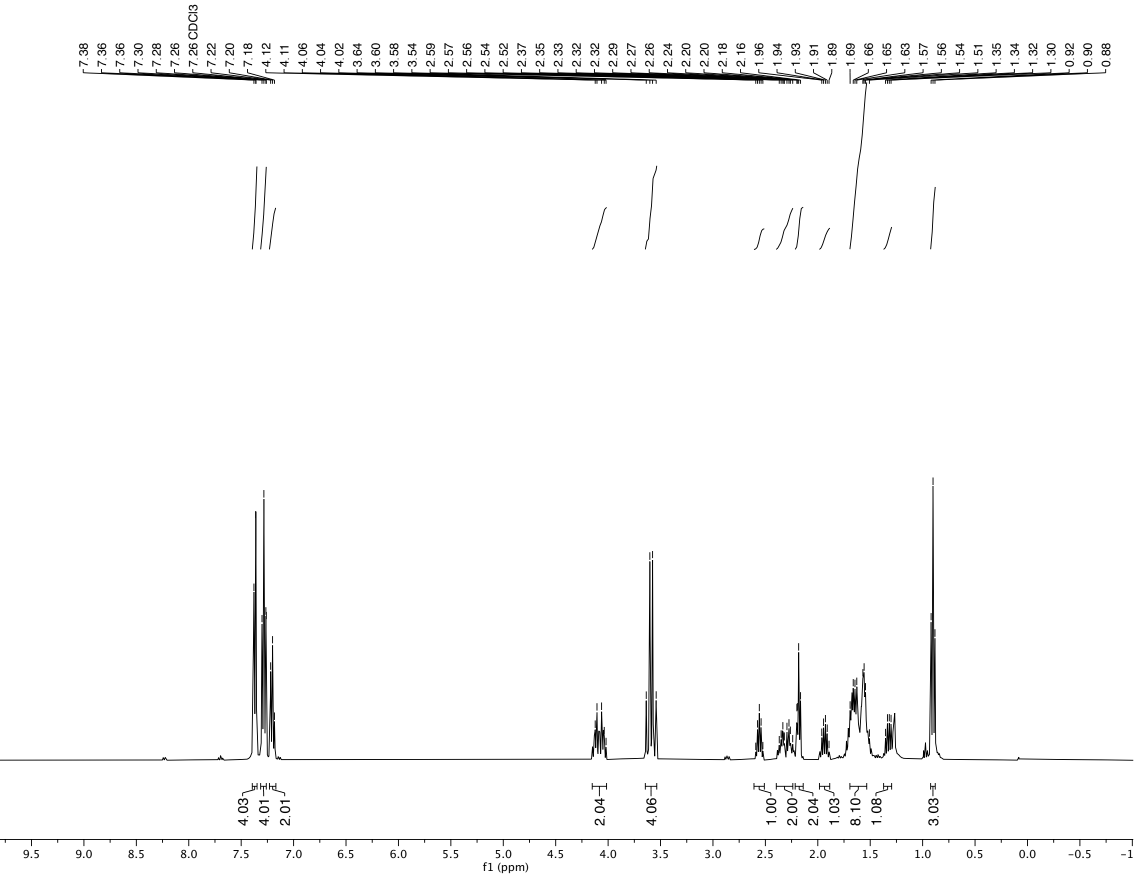
7.38 7.36 7.36 7.30 7.28 7.26 7.26 CDCl3 7.22 7.20 7.18 4.12 4.11 4.06 4.04 4.02 3.64 3.60 3.58 3.54 2.59 2.57 2.56 2.54 2.52 2.37 2.35 2.33 2.32 2.32 2.29 2.27 2.26 2.24 2.20 2.20 2.18 2.16 1.96 1.94 1.93 1.91 1.89 1.69 1.66 1.65 1.63 1.57 1.56 1.54 1.51 1.35 1.34 1.32 1.30 0.92 0.90 0.88
4.03
4.01
2.01
2.04
4.06
1.00
2.00
2.04
1.03
8.10
1.08
3.03
10.5 10.0 9.5 9.0 8.5 8.0 7.5 7.0 6.5 6.0 5.5 5.0 4.5 4.0 3.5 3.0 2.5 2.0 1.5 1.0 0.5 0.0 −0.5 −1
f1 (ppm)

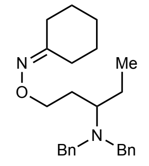
N
O
Me
Bn
N
Bn

7g

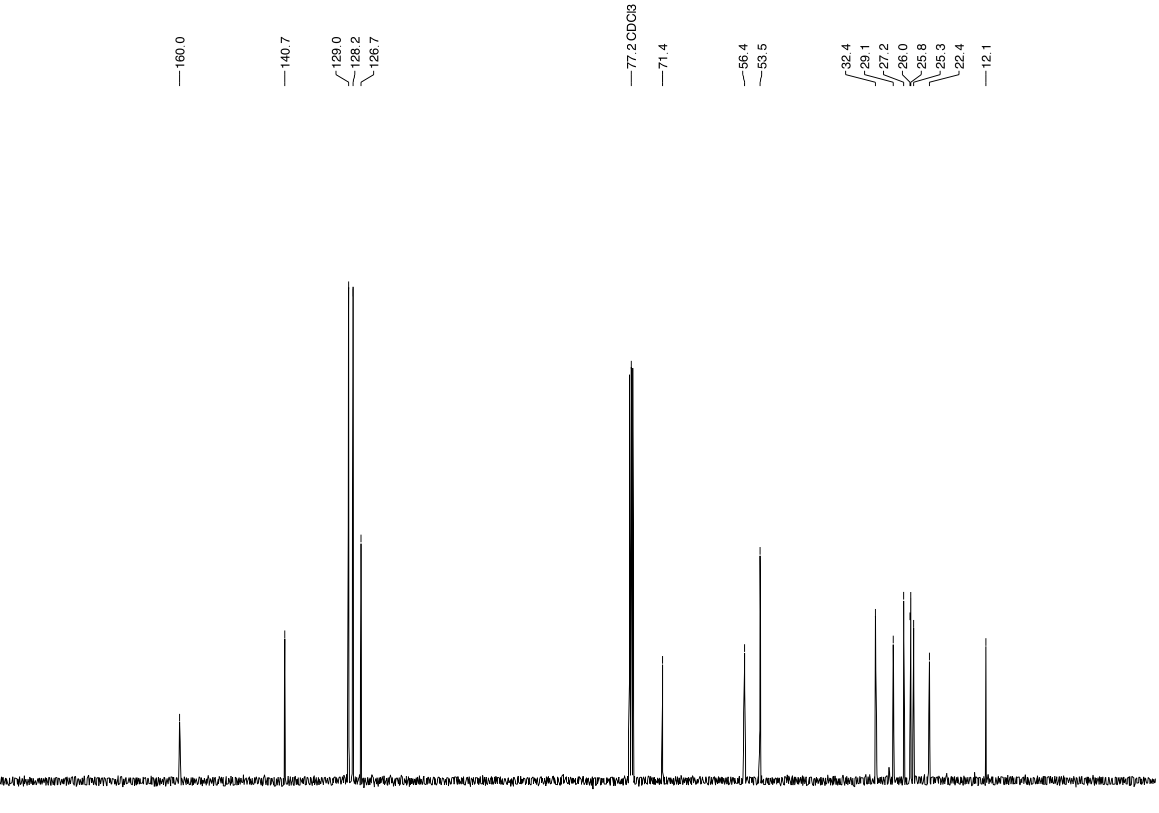
160.0
140.7
129.0
128.2
126.7
77.2 CDCl3
71.4
56.4
53.5
32.4
29.1
27.2
26.0
25.8
25.3
22.4
12.1
210 200 190 180 170 160 150 140 130 120 110 100 90 80 70 60 50 40 30 20 10 0 −10
f1 (ppm)

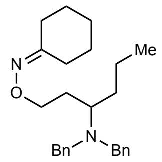
Me
N
O
Bn
N
Bn

7h

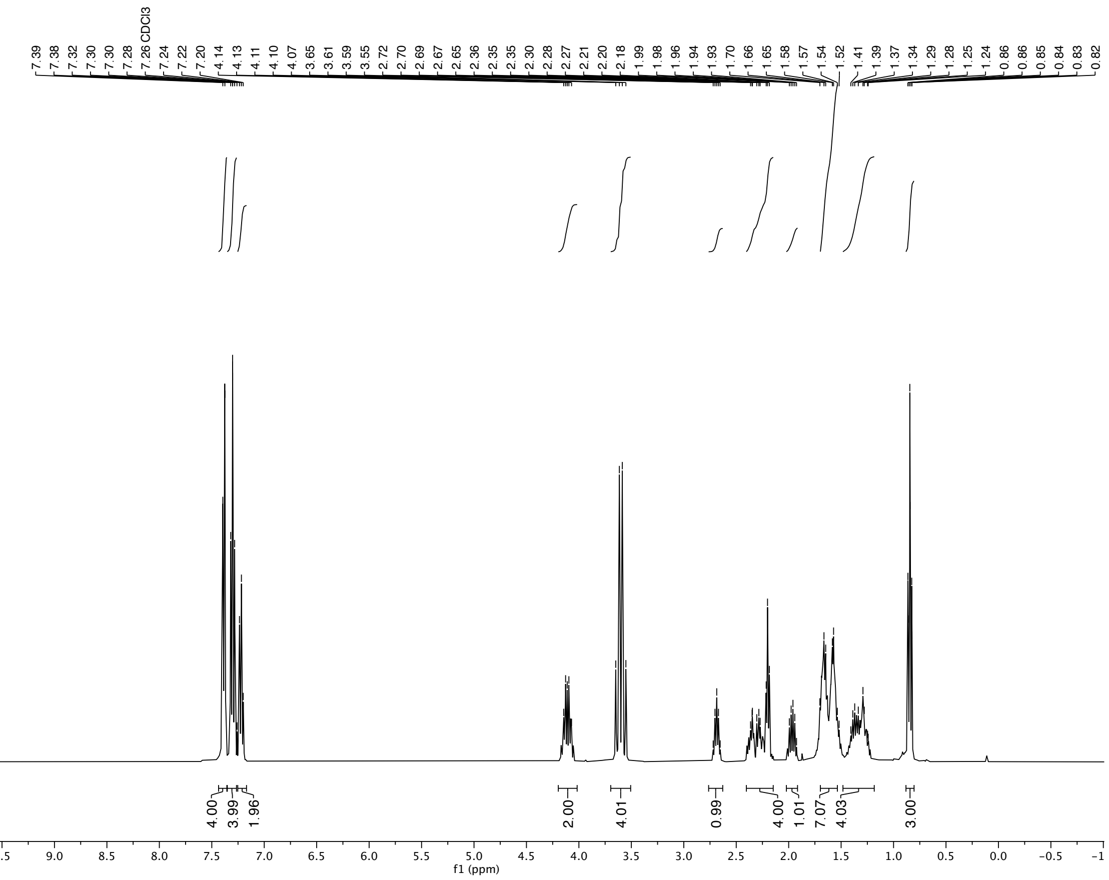
7.39
7.38
7.32
7.30
7.30
7.28
7.26 CDCl3
7.24
7.22
7.20
4.14
4.13
4.11
4.10
4.07
3.65
3.61
3.59
3.55
2.72
2.70
2.69
2.67
2.65
2.36
2.35
2.35
2.30
2.28
2.27
2.21
2.20
2.18
1.99
1.98
1.96
1.94
1.93
1.70
1.66
1.65
1.58
1.57
1.54
1.52
1.41
1.39
1.37
1.34
1.29
1.28
1.25
1.24
0.86
0.86
0.85
0.84
0.83
0.82
4.00
3.99
1.96
2.00
4.01
0.99
4.00
1.01
7.07
4.03
3.00
f1 (ppm)

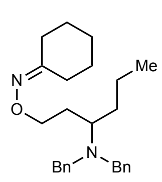
Me
N
O
Bn
N
Bn

7h

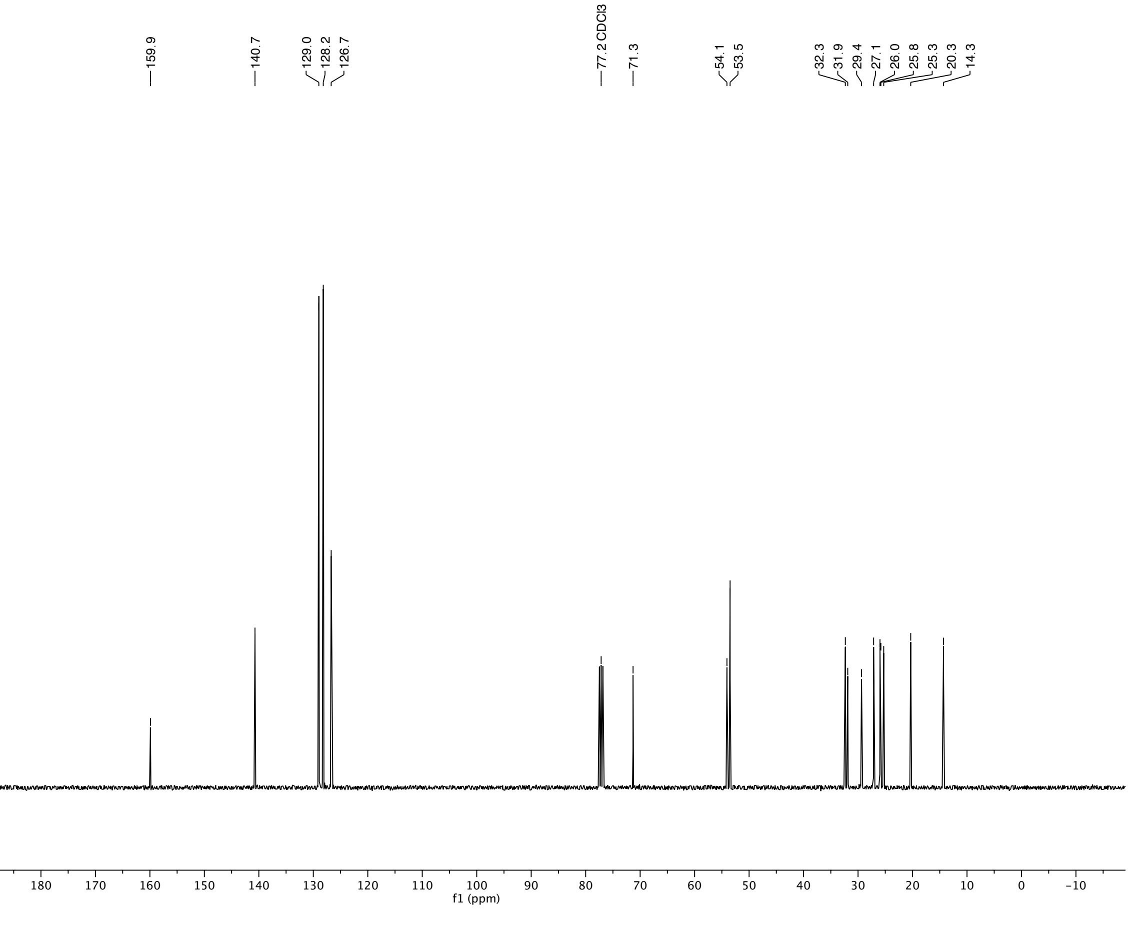
159.9
140.7
129.0
128.2
126.7
77.2 CDCl3
71.3
54.1
53.5
32.3
31.9
29.4
27.1
26.0
25.8
25.3
20.3
14.3
f1 (ppm)

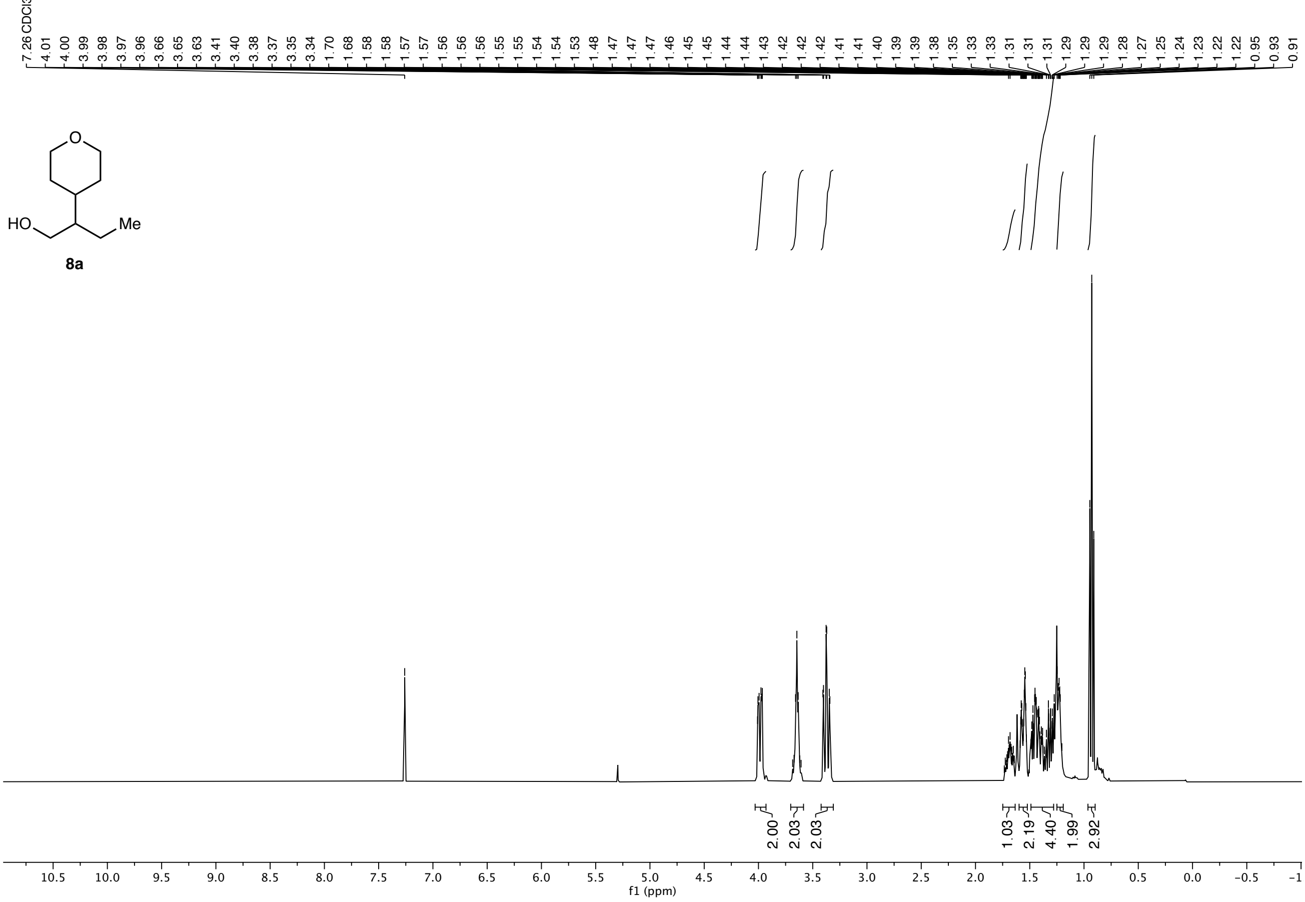
7.26 CDCl3
4.01 4.00 3.99 3.98 3.97 3.96 3.66 3.65 3.63 3.41 3.40 3.38 3.37 3.35 3.34 1.70 1.68 1.58 1.58 1.57 1.57 1.56 1.56 1.56 1.55 1.55 1.54 1.54 1.53 1.48 1.47 1.47 1.47 1.46 1.45 1.45 1.44 1.44 1.43 1.42 1.42 1.42 1.41 1.41 1.40 1.39 1.39 1.38 1.35 1.33 1.33 1.33 1.31 1.31 1.31 1.29 1.29 1.29 1.28 1.27 1.25 1.24 1.23 1.22 1.22 0.95 0.93 0.91
O
HO
Me
8a
2.00
2.03
2.03
1.03
2.19
4.40
1.99
2.92
10.5 10.0 9.5 9.0 8.5 8.0 7.5 7.0 6.5 6.0 5.5 5.0 4.5 4.0 3.5 3.0 2.5 2.0 1.5 1.0 0.5 0.0 −0.5 −1
f1 (ppm)

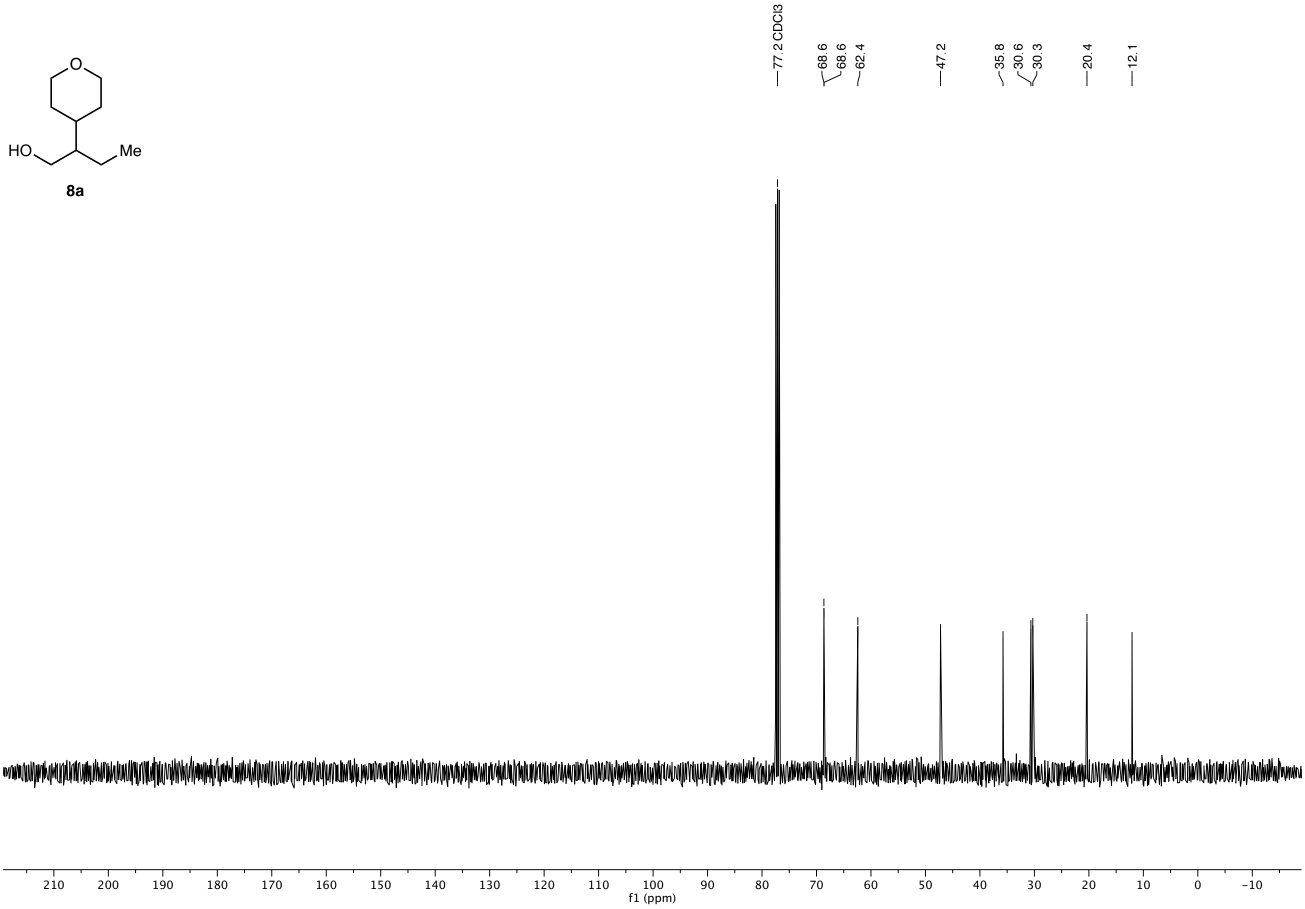
77.2 CDCl3
68.6
68.6
62.4
47.2
35.8
30.6
30.3
20.4
12.1
O
HO
Me
8a
210 200 190 180 170 160 150 140 130 120 110 100 90 80 70 60 50 40 30 20 10 0 −10
f1 (ppm)

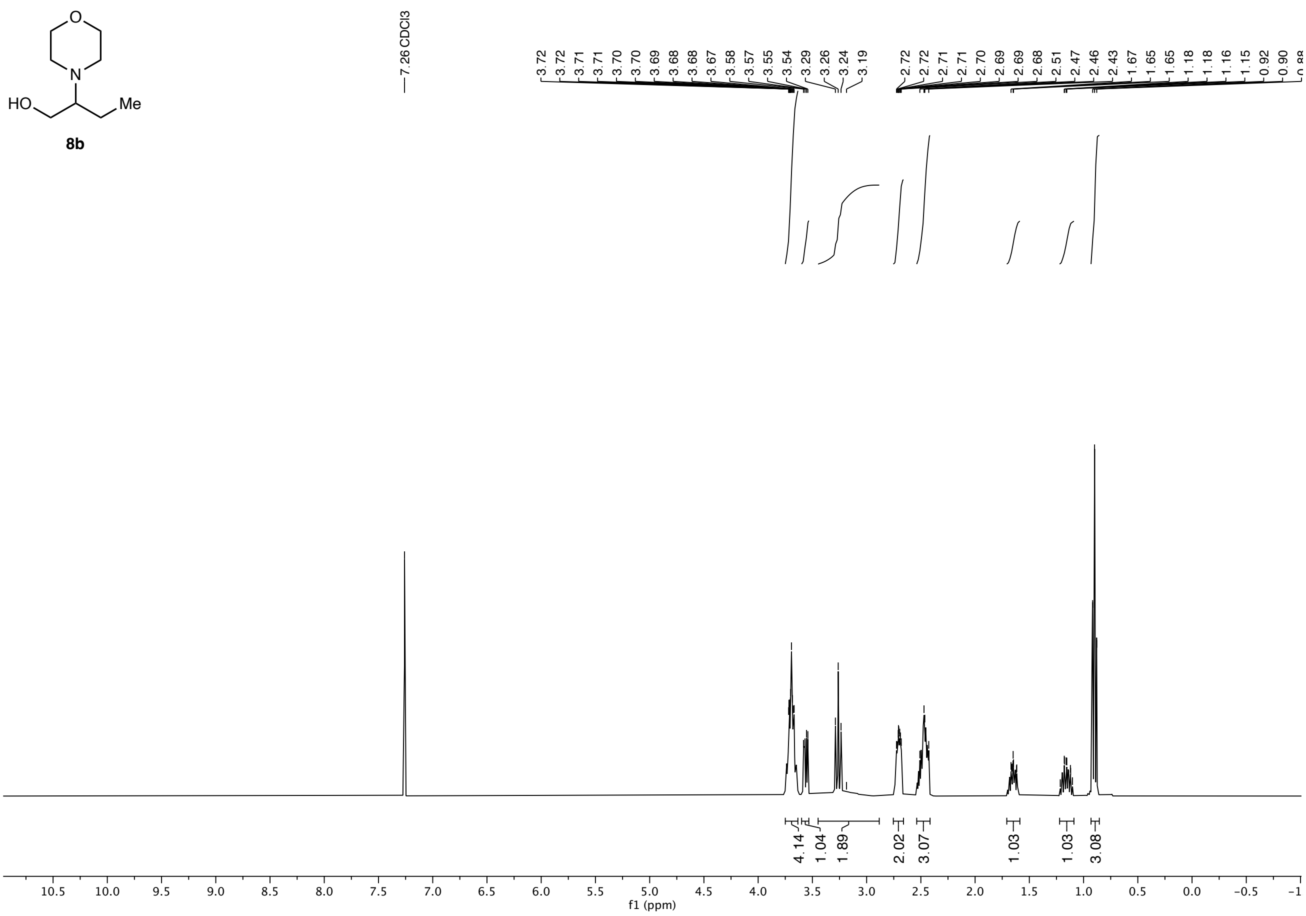
HO
N
O
Me
7.26 CDCl3
3.72
3.72
3.71
3.71
3.70
3.70
3.69
3.68
3.68
3.67
3.58
3.57
3.55
3.54
3.29
3.26
3.24
3.19
2.72
2.72
2.71
2.71
2.70
2.69
2.69
2.68
2.51
2.47
2.46
2.43
1.67
1.65
1.65
1.18
1.18
1.16
1.15
0.92
0.90
4.14
1.04
1.89
2.02
3.07
1.03
1.03
3.08
f1 (ppm)

8b

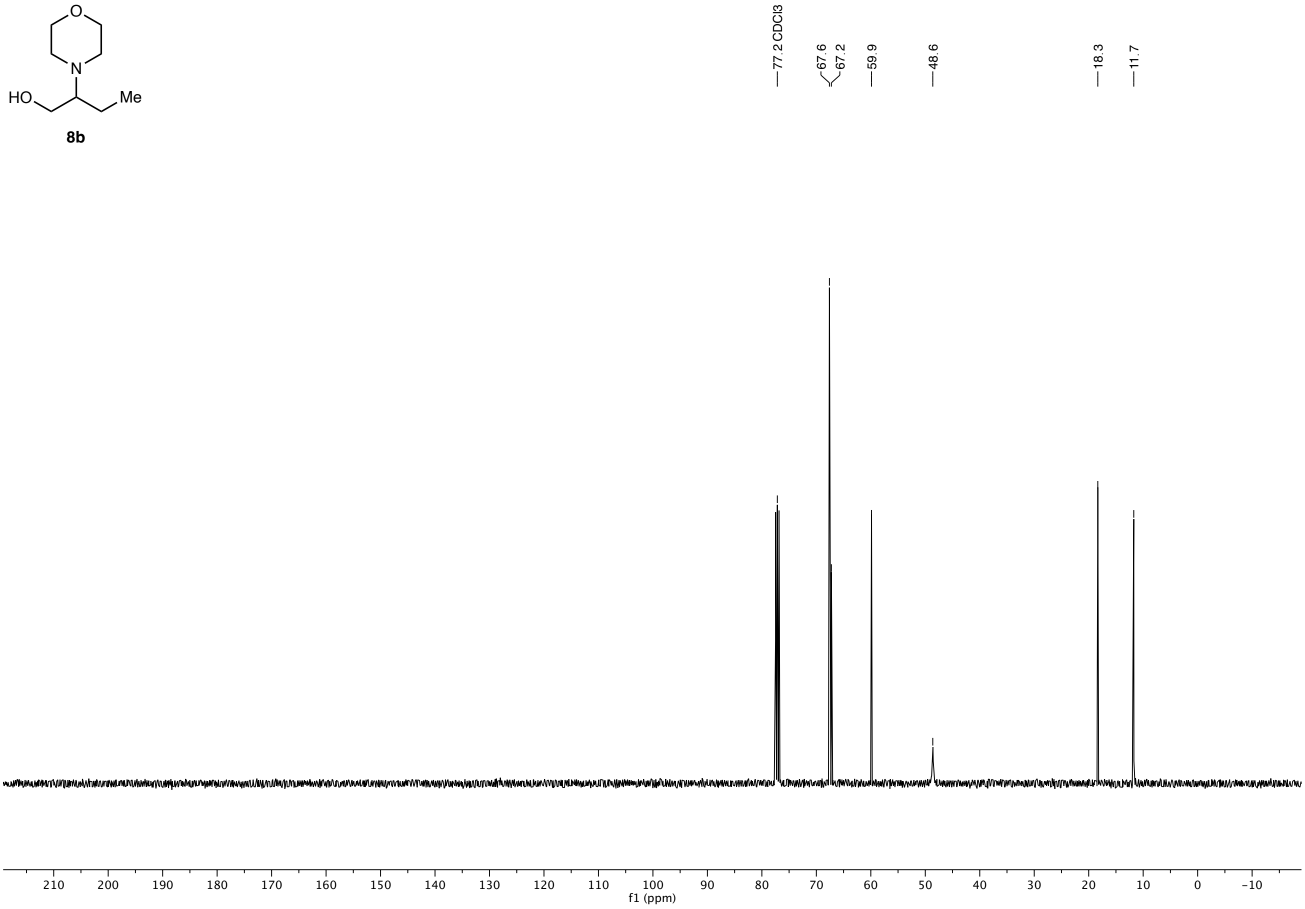
HO
N
O
Me
77.2 CDCl3
67.6
67.2
59.9
48.6
18.3
11.7
f1 (ppm)

8b

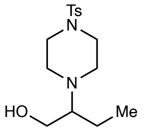
Ts
N
N
HO
Me

8c

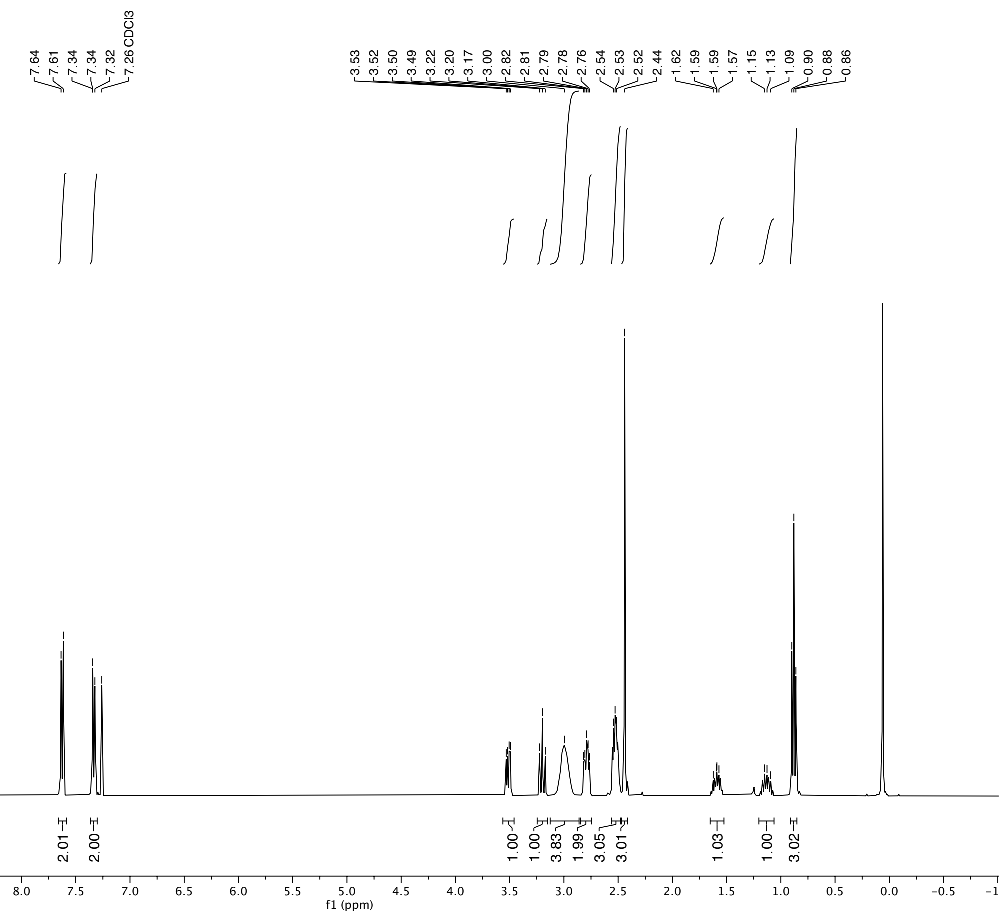
7.64
7.61
7.34
7.34
7.32
7.26 CDCl3
3.53
3.52
3.50
3.49
3.22
3.20
3.17
3.00
2.82
2.81
2.79
2.78
2.76
2.54
2.53
2.52
2.44
1.62
1.59
1.59
1.57
1.15
1.13
1.09
0.90
0.88
0.86
2.01
2.00
1.00
1.00
3.83
1.99
3.05
3.01
1.03
1.00
3.02
f1 (ppm)

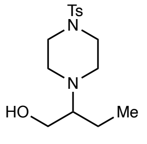
Ts
N
N
HO
Me

8c

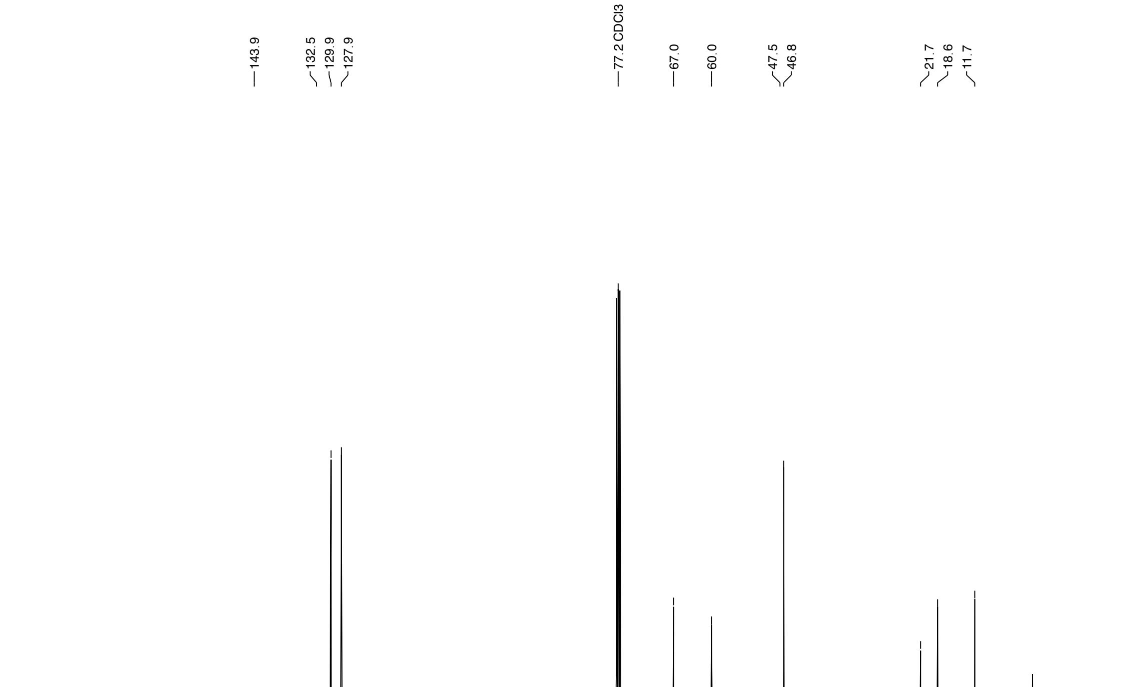
143.9
132.5
129.9
127.9
77.2 CDCl3
67.0
60.0
47.5
46.8
21.7
18.6
11.7
f1 (ppm)

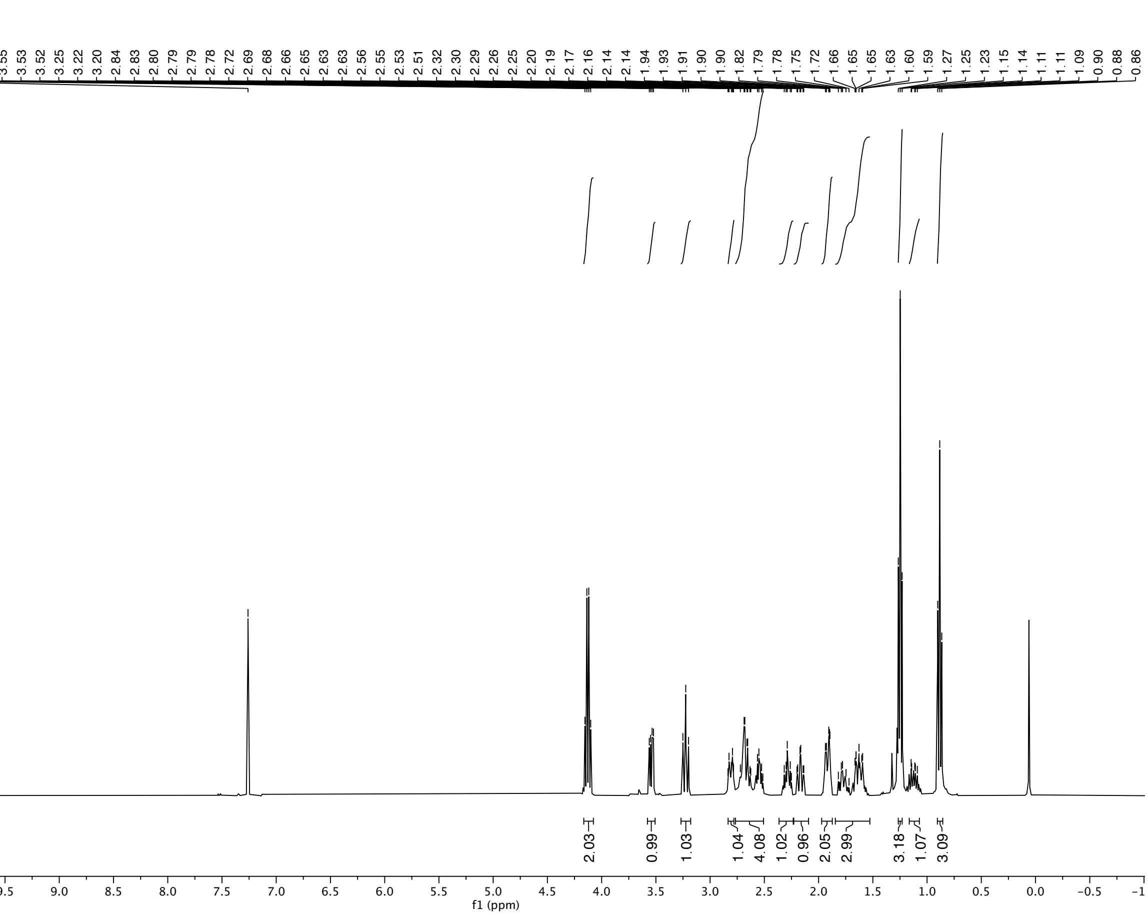
7.26 CDCl3
4.15
4.14
4.12
4.10
3.56
3.55
3.53
3.52
3.25
3.22
3.20
2.84
2.83
2.80
2.79
2.79
2.78
2.72
2.69
2.68
2.66
2.65
2.63
2.63
2.56
2.55
2.53
2.51
2.32
2.30
2.29
2.26
2.25
2.20
2.19
2.17
2.16
2.14
1.94
1.93
1.91
1.90
1.82
1.79
1.78
1.75
1.72
1.66
1.65
1.65
1.63
1.60
1.59
1.27
1.25
1.23
1.15
1.14
1.11
1.09
0.90
0.88
0.86
2.03
0.99
1.03
1.04
4.08
1.02
0.96
2.05
2.99
3.18
1.07
3.09
f1 (ppm)

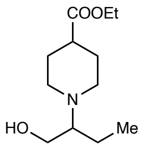
COOEt
N
HO
Me

8d

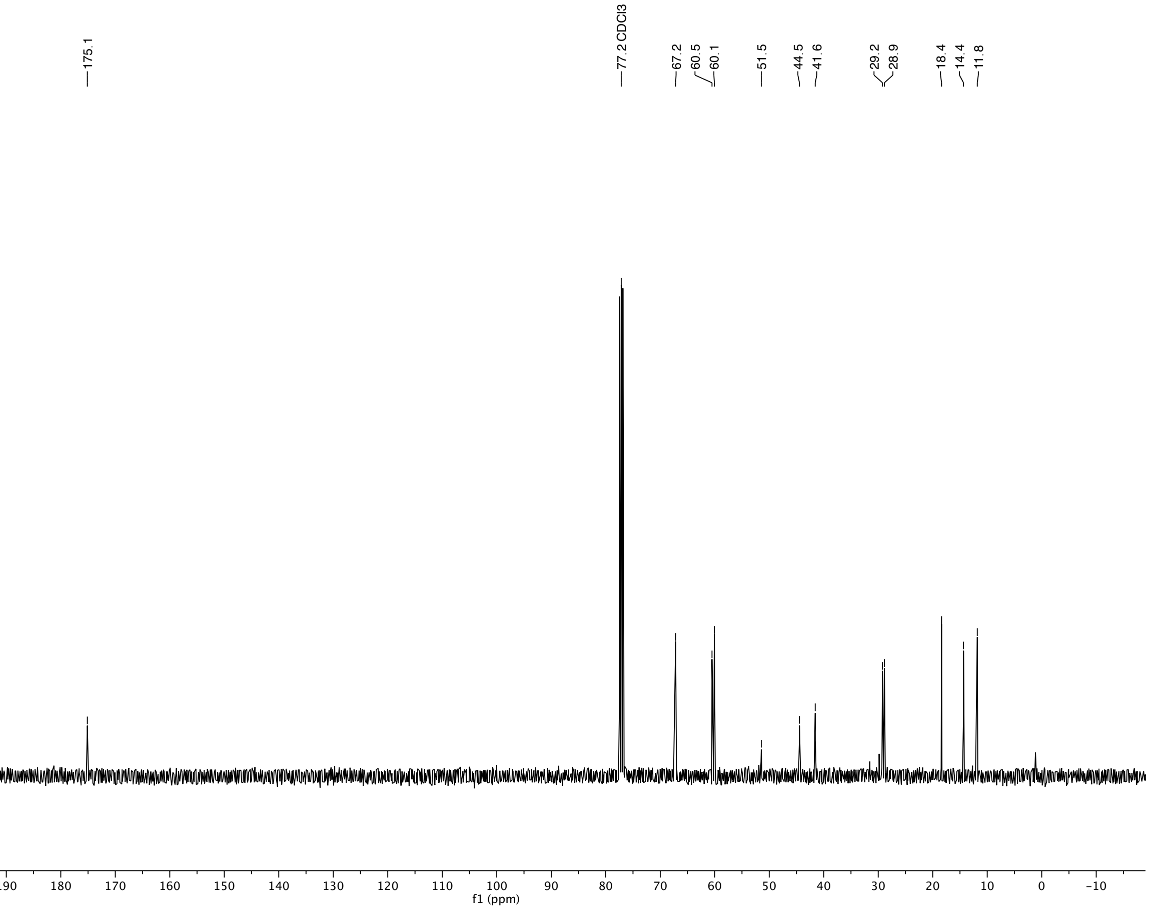
175.1
77.2 CDCl3
67.2
60.5
60.1
51.5
44.5
41.6
29.2
28.9
18.4
14.4
11.8
f1 (ppm)

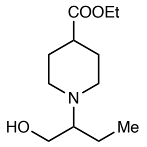
COOEt
N
HO
Me

8d

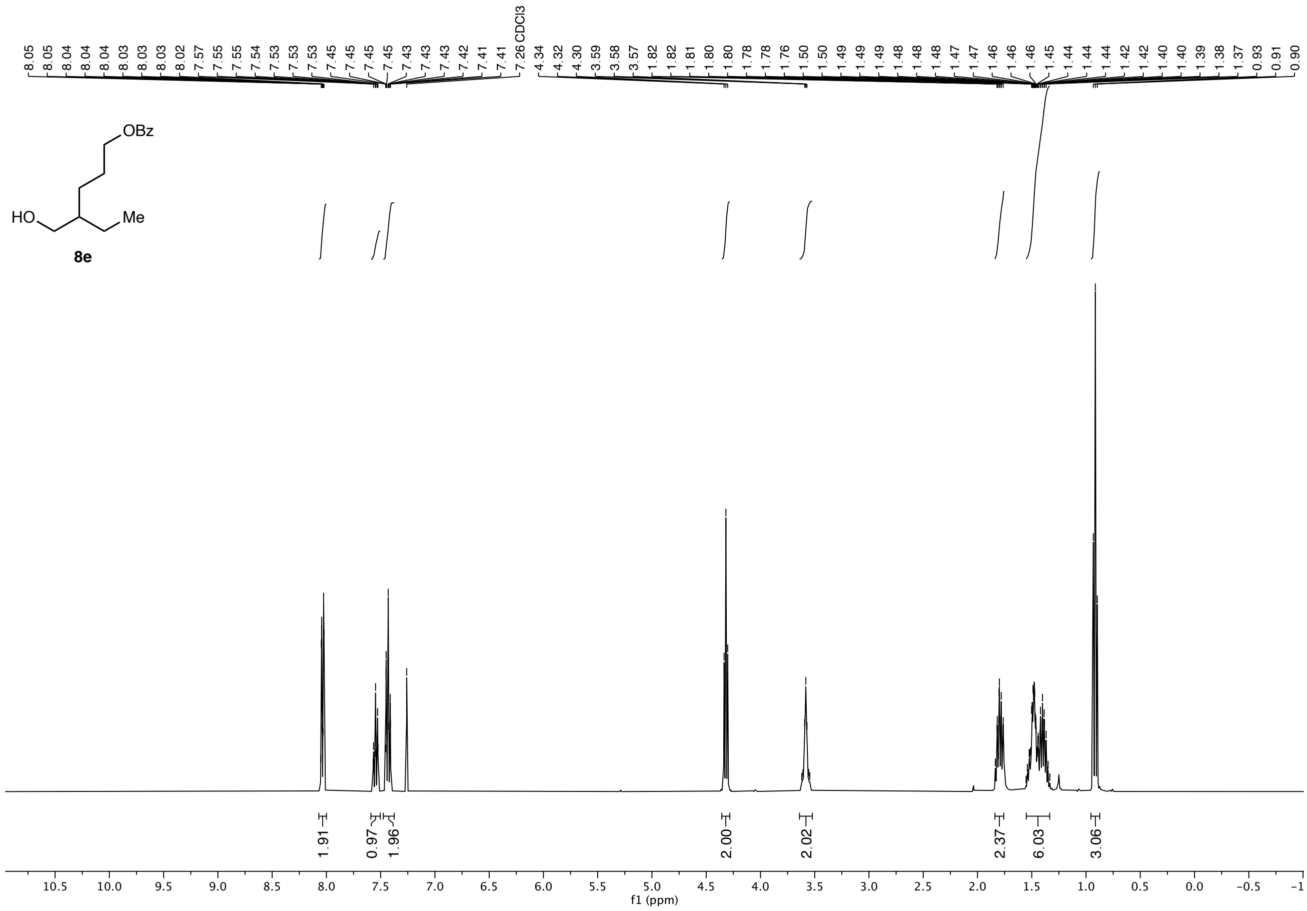
8.05 8.05 8.04 8.04 8.03 8.03 8.03 8.02 7.57 7.55 7.55 7.54 7.53 7.53 7.53 7.45 7.45 7.45 7.45 7.43 7.43 7.43 7.42 7.41 7.41 7.26 CDCl3 4.34 4.32 4.30 3.59 3.58 3.57 1.82 1.82 1.81 1.80 1.80 1.78 1.78 1.76 1.50 1.50 1.49 1.49 1.49 1.48 1.48 1.48 1.47 1.47 1.46 1.46 1.46 1.45 1.44 1.44 1.44 1.42 1.42 1.40 1.40 1.39 1.38 1.37 0.93 0.91 0.90
OBz
HO
Me
8e
1.91
0.97
1.96
2.00
2.02
2.37
6.03
3.06
f1 (ppm)

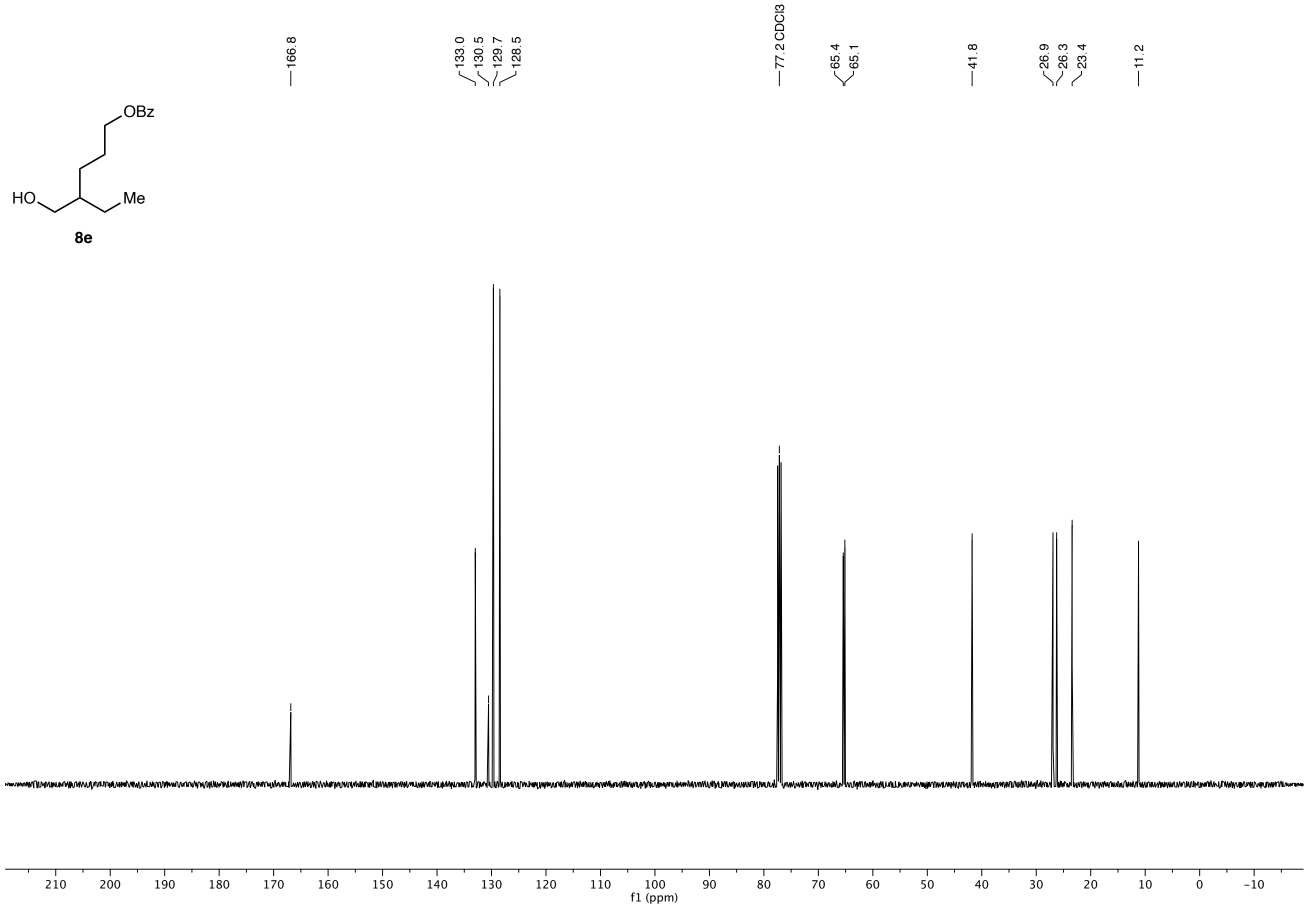
166.8
133.0 130.5 129.7 128.5
77.2 CDCl3
65.4 65.1
41.8
26.9 26.3 23.4
11.2
OBz
HO
Me
8e
f1 (ppm)

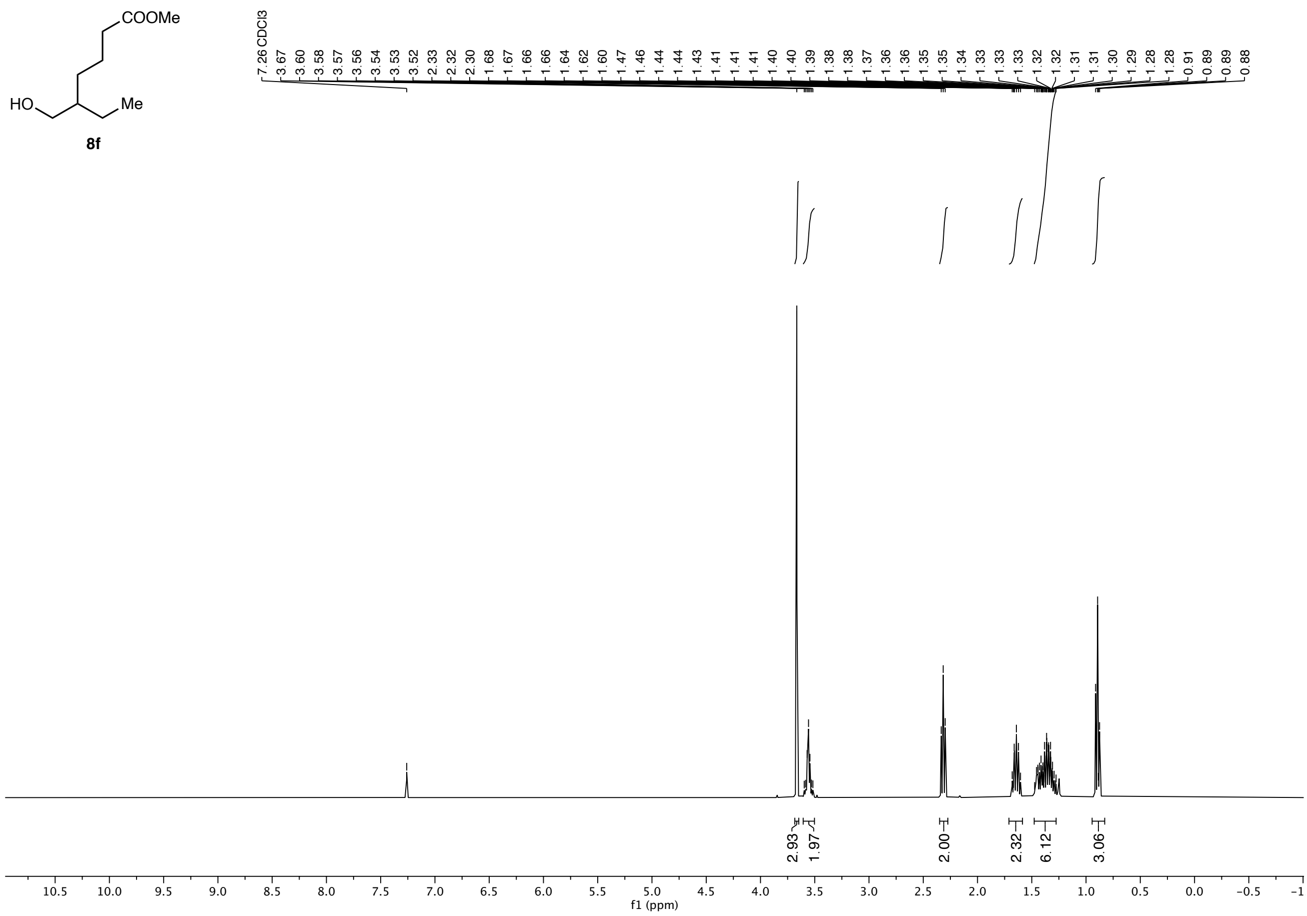
COOMe
HO
Me
8f
7.26 CDCl3
3.67
3.60
3.58
3.57
3.56
3.54
3.53
3.52
2.33
2.32
2.30
1.68
1.67
1.66
1.66
1.64
1.62
1.60
1.47
1.46
1.44
1.44
1.43
1.41
1.41
1.41
1.40
1.40
1.39
1.38
1.38
1.37
1.36
1.36
1.35
1.35
1.34
1.33
1.33
1.33
1.32
1.32
1.31
1.31
1.30
1.29
1.28
1.28
0.91
0.89
0.89
0.88
2.93
1.97
2.00
2.32
6.12
3.06
f1 (ppm)

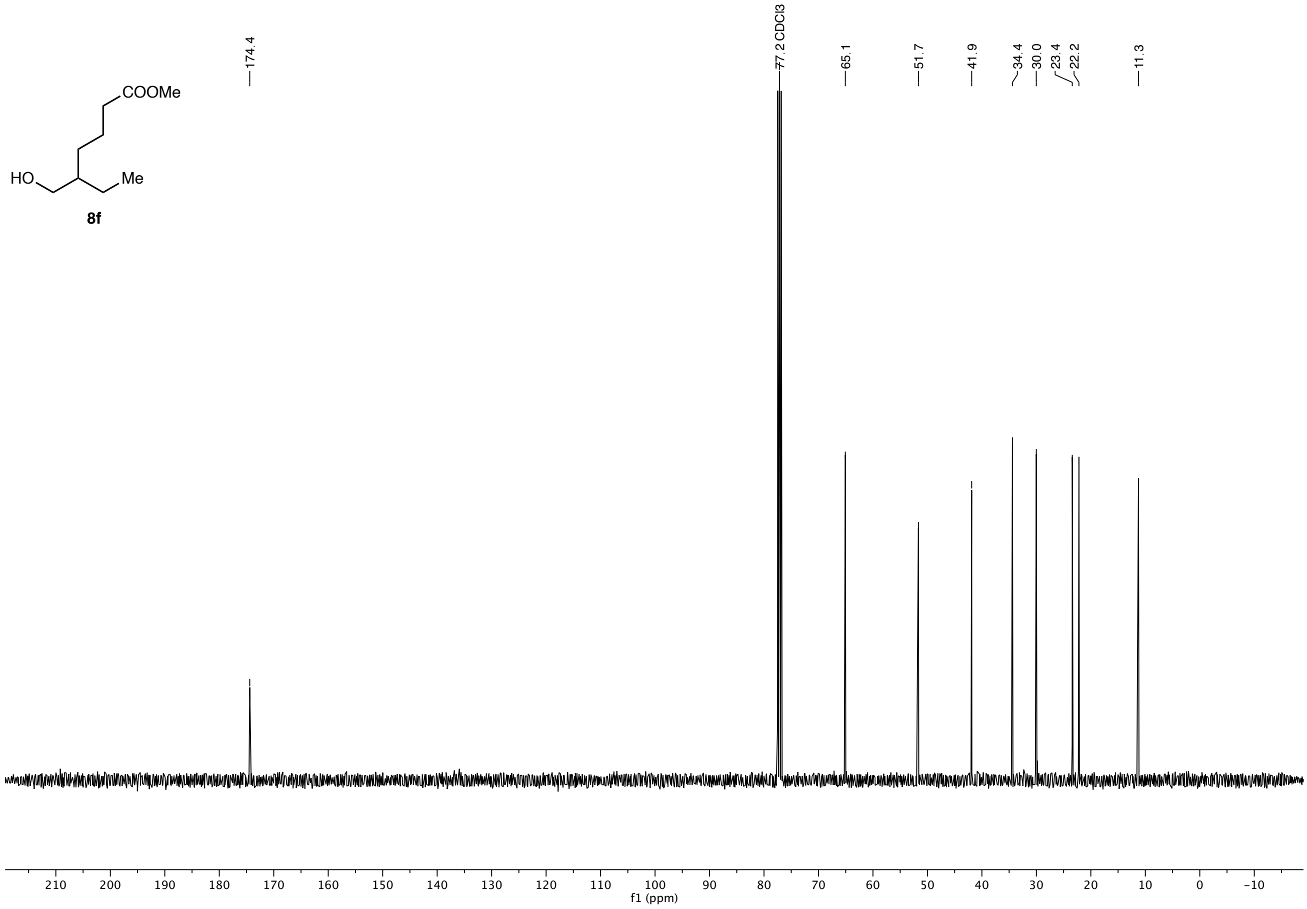
COOMe
HO
Me
8f
174.4
77.2 CDCl3
65.1
51.7
41.9
34.4
30.0
23.4
22.2
11.3
f1 (ppm)

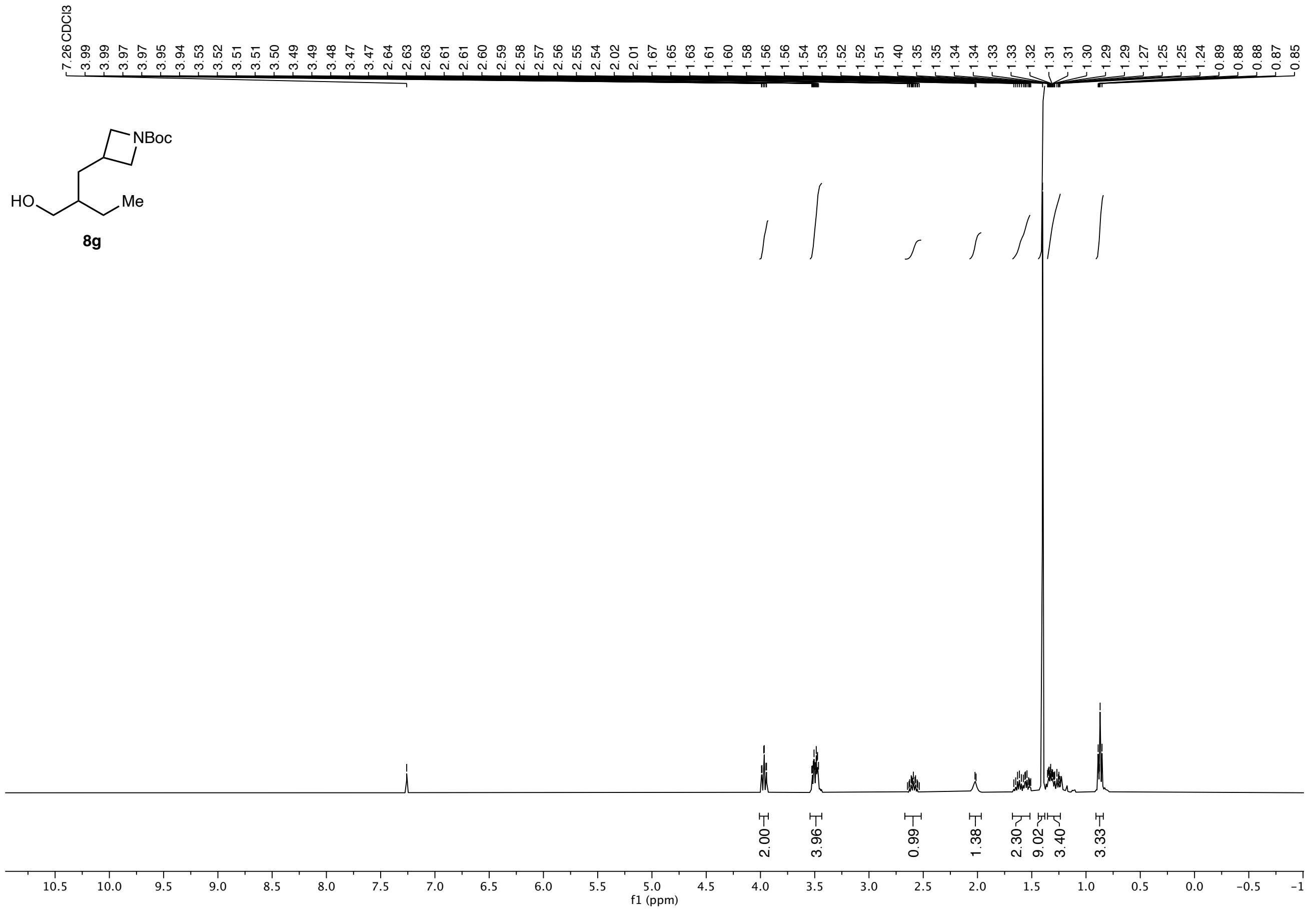
7.26 CDCl3
3.99 3.99 3.97 3.97 3.95 3.94 3.53 3.52 3.51 3.51 3.50 3.49 3.49 3.48 3.47 3.47 2.64 2.63 2.63 2.61 2.61 2.60 2.59 2.58 2.57 2.56 2.55 2.54 2.02 2.01 1.67 1.65 1.63 1.61 1.60 1.58 1.56 1.56 1.54 1.53 1.52 1.52 1.51 1.40 1.35 1.35 1.34 1.34 1.33 1.33 1.32 1.31 1.31 1.30 1.29 1.29 1.27 1.25 1.25 1.24 0.89 0.88 0.88 0.87 0.85
NBoc
HO
Me
8g
2.00
3.96
0.99
1.38
2.30
9.02
3.40
3.33
f1 (ppm)

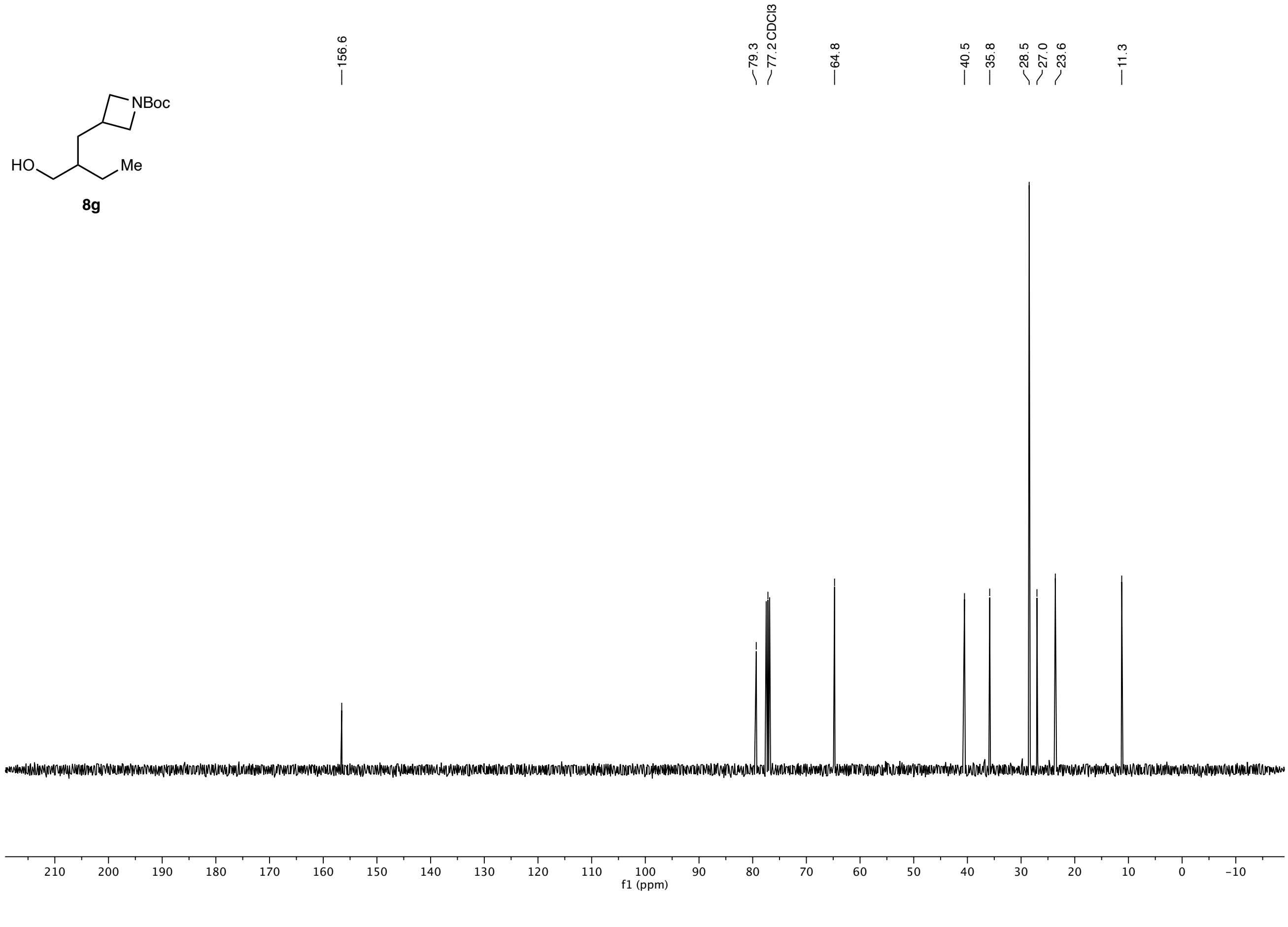
156.6
79.3
77.2 CDCl3
64.8
40.5
35.8
28.5
27.0
23.6
11.3
NBoc
HO
Me
8g
f1 (ppm)

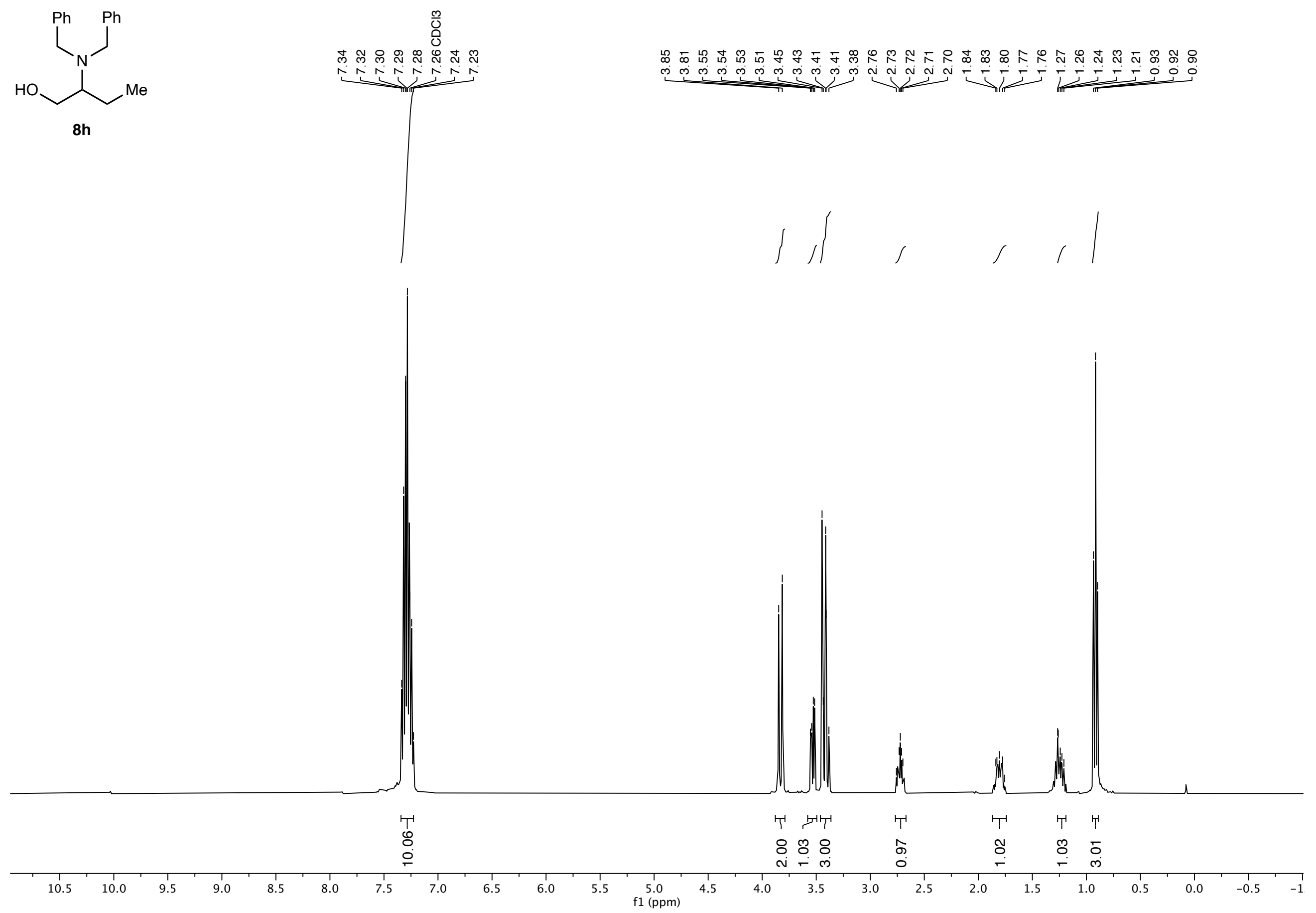
Ph
Ph
N
HO
Me
8h
7.34
7.32
7.30
7.29
7.28
7.26 CDCl3
7.24
7.23
3.85
3.81
3.55
3.54
3.53
3.51
3.45
3.43
3.41
3.41
3.38
2.76
2.73
2.72
2.71
2.70
1.84
1.83
1.80
1.77
1.76
1.27
1.26
1.24
1.23
1.21
0.93
0.92
0.90
10.06
2.00
1.03
3.00
0.97
1.02
1.03
3.01
f1 (ppm)

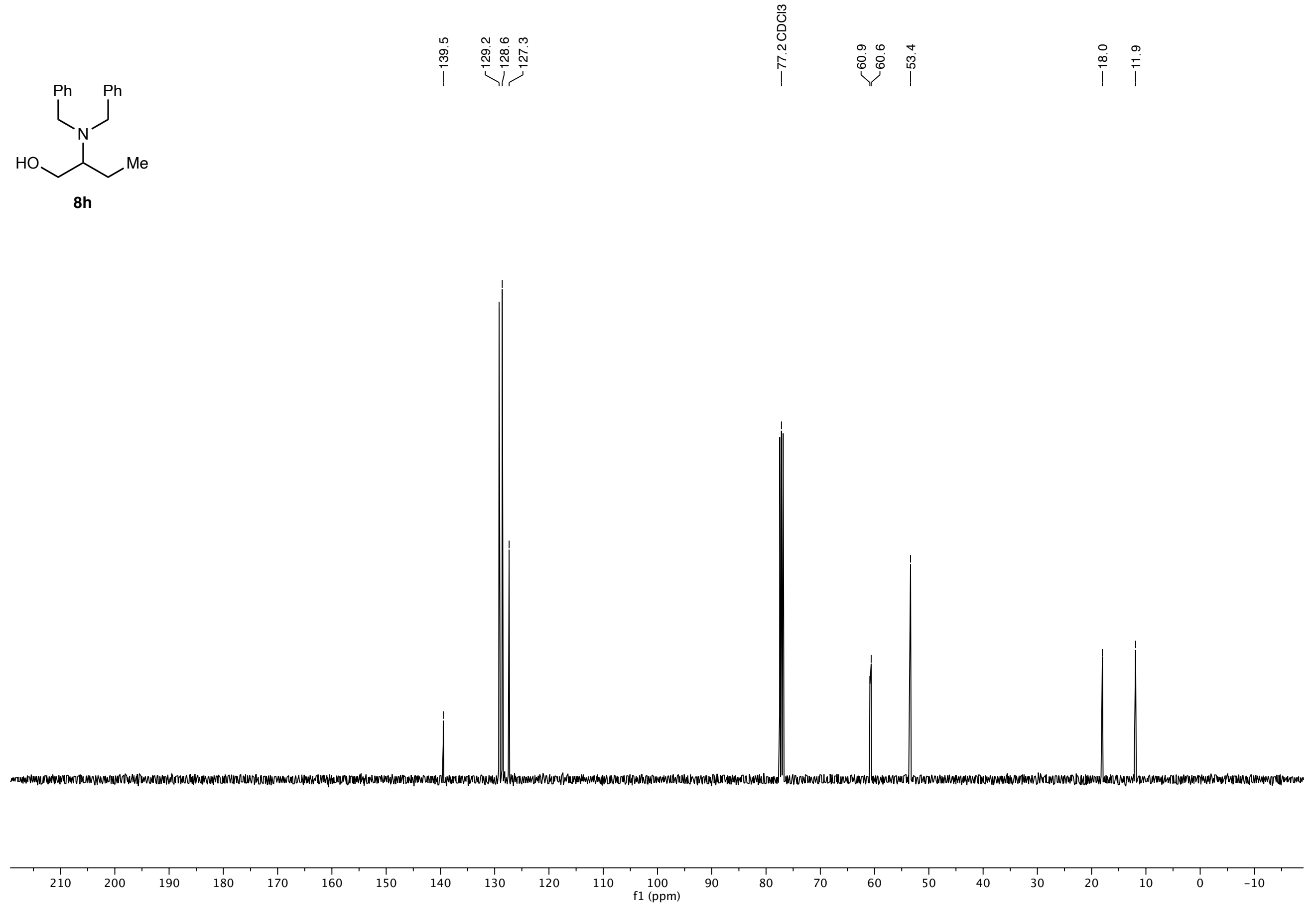
139.5
129.2
128.6
127.3
77.2 CDCl3
60.9
60.6
53.4
18.0
11.9
Ph
Ph
N
HO
Me
8h
f1 (ppm)

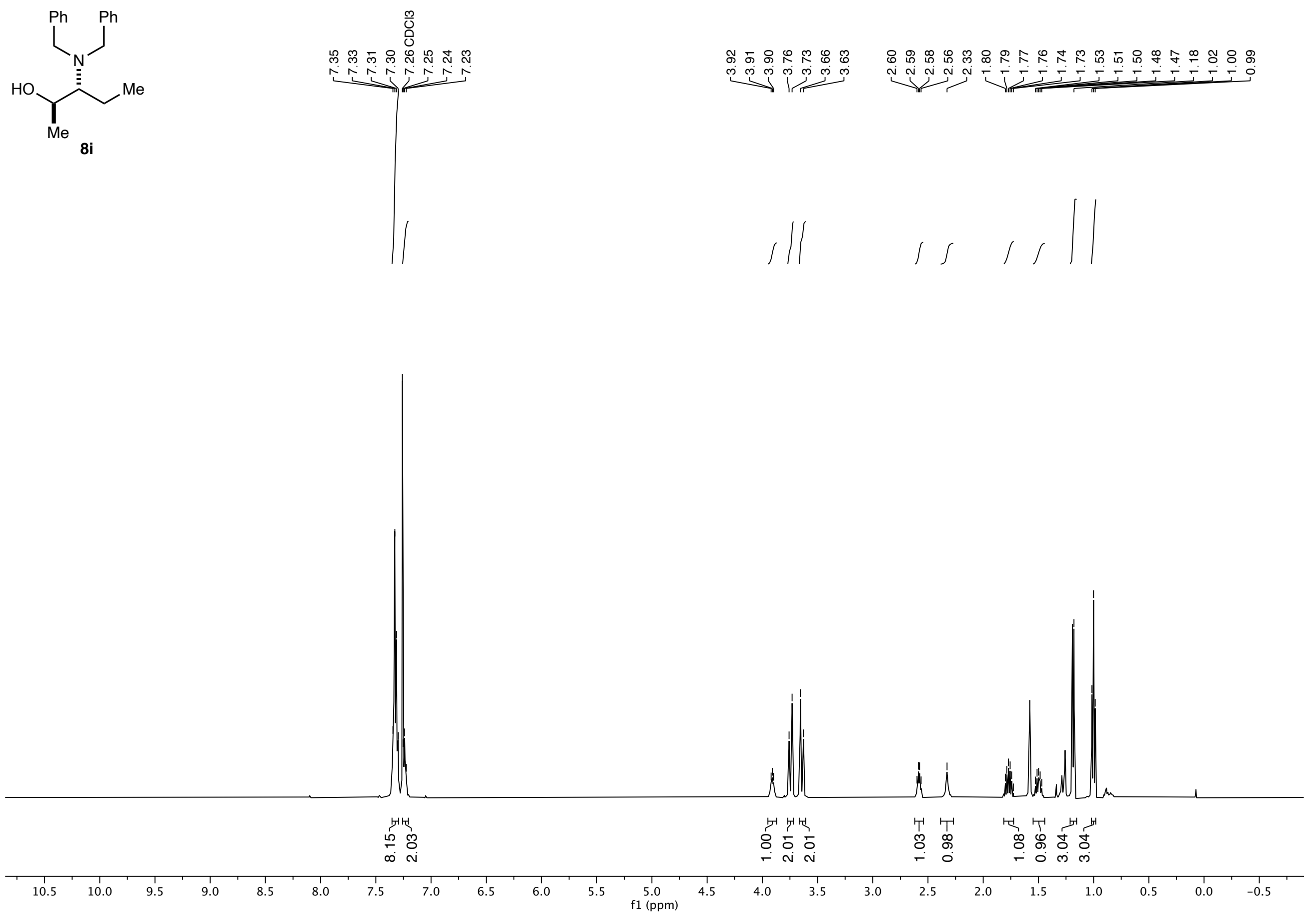
Ph
Ph
N
HO
Me
Me
8i
7.35
7.33
7.31
7.30
7.26 CDCl3
7.25
7.24
7.23
3.92
3.91
3.90
3.76
3.73
3.66
3.63
2.60
2.59
2.58
2.56
2.33
1.80
1.79
1.77
1.76
1.74
1.73
1.53
1.51
1.50
1.48
1.47
1.18
1.02
1.00
0.99
8.15
2.03
1.00
2.01
2.01
1.03
0.98
1.08
0.96
3.04
3.04
f1 (ppm)

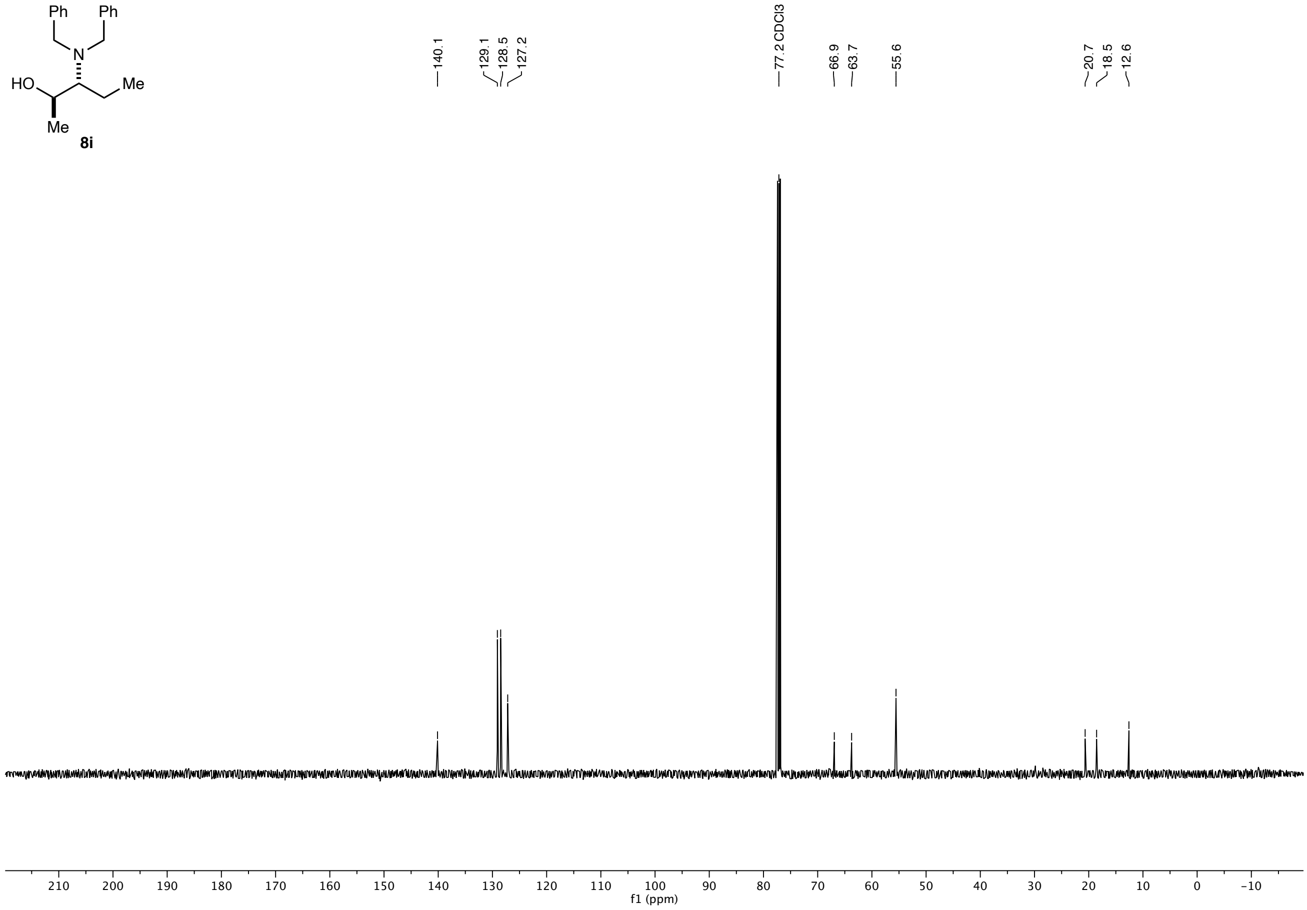
Ph
Ph
N
HO
Me
Me
8i
140.1
129.1
128.5
127.2
77.2 CDCl3
66.9
63.7
55.6
20.7
18.5
12.6
f1 (ppm)

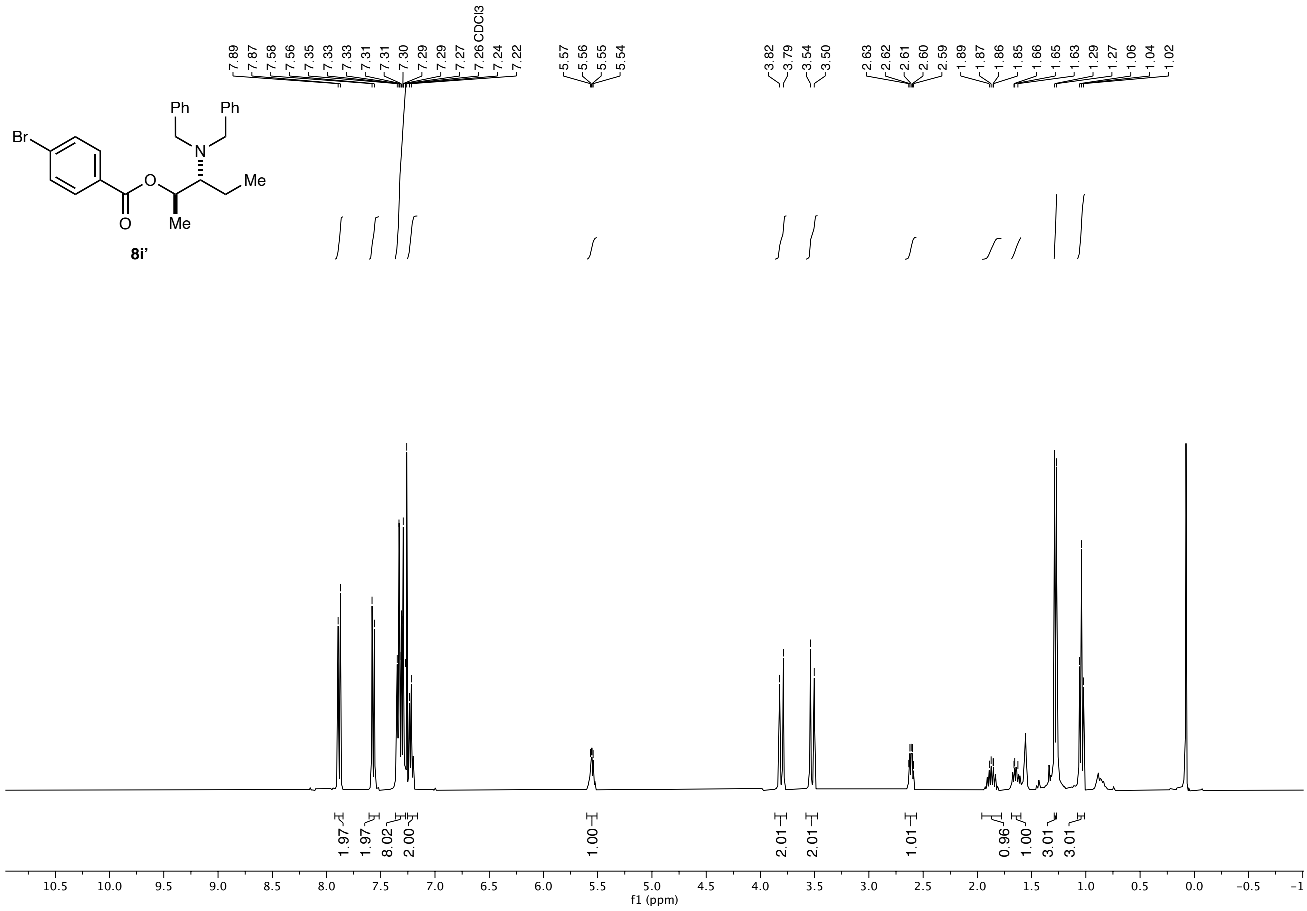
Br
Ph Ph
N
O
Me
O
Me
8i’
7.89 7.87 7.58 7.56 7.35 7.33 7.31 7.31 7.30 7.29 7.29 7.27 7.26 CDCl3 7.24 7.22
5.57 5.56 5.55 5.54
3.82 3.79 3.54 3.50
2.63 2.62 2.61 2.60 2.59 1.89 1.87 1.86 1.85 1.66 1.65 1.63 1.29 1.27 1.06 1.04 1.02
1.97 1.97 8.02 2.00 1.00 2.01 2.01 1.01 0.96 1.00 3.01 3.01
f1 (ppm)

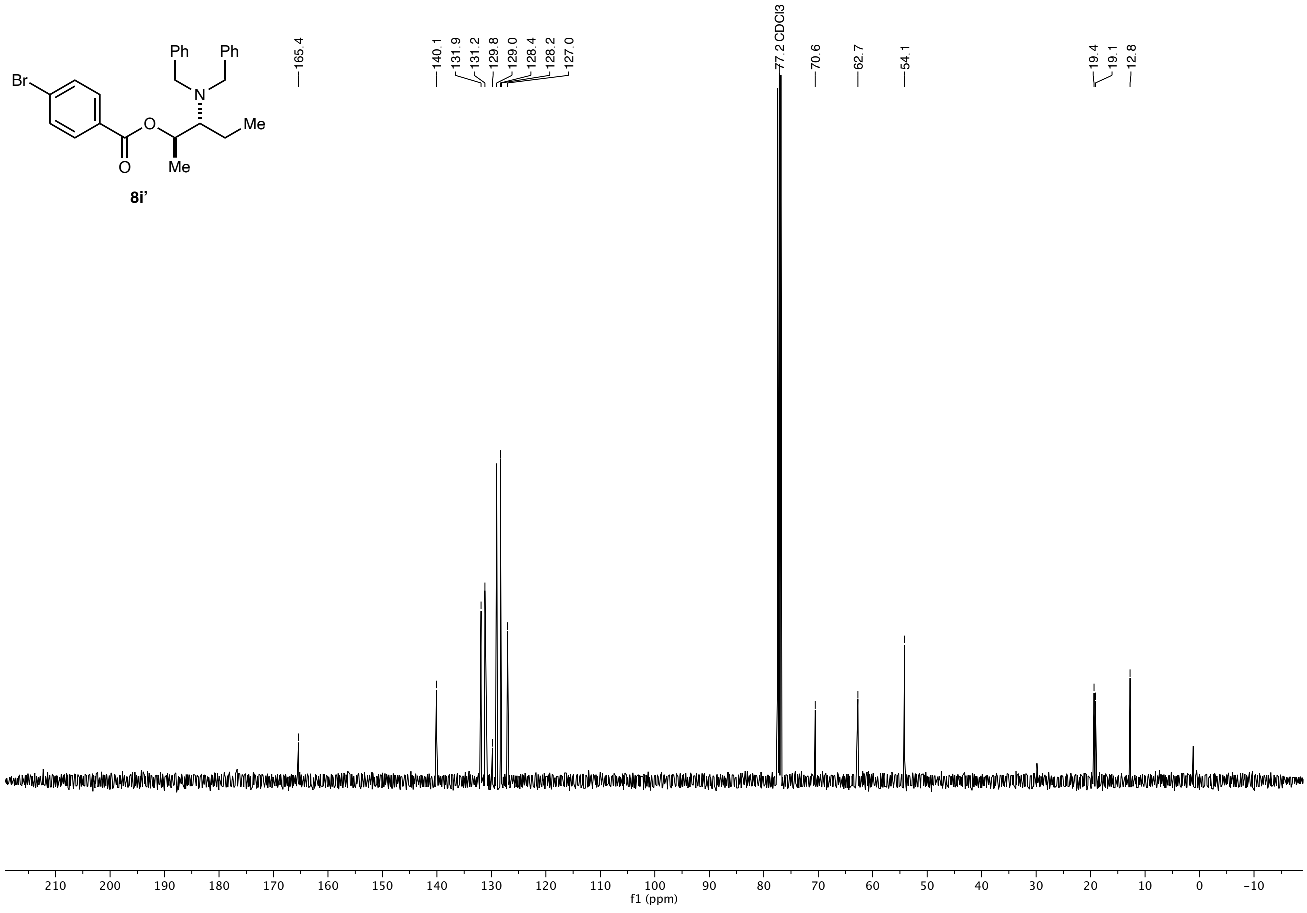
Br
Ph Ph
N
O
Me
O
Me
8i’
165.4
140.1 131.9 131.2 129.8 129.0 128.4 128.2 127.0
77.2 CDCl3
70.6
62.7
54.1
19.4 19.1 12.8
f1 (ppm)

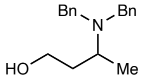
Bn
N
Bn
HO
Me

8j

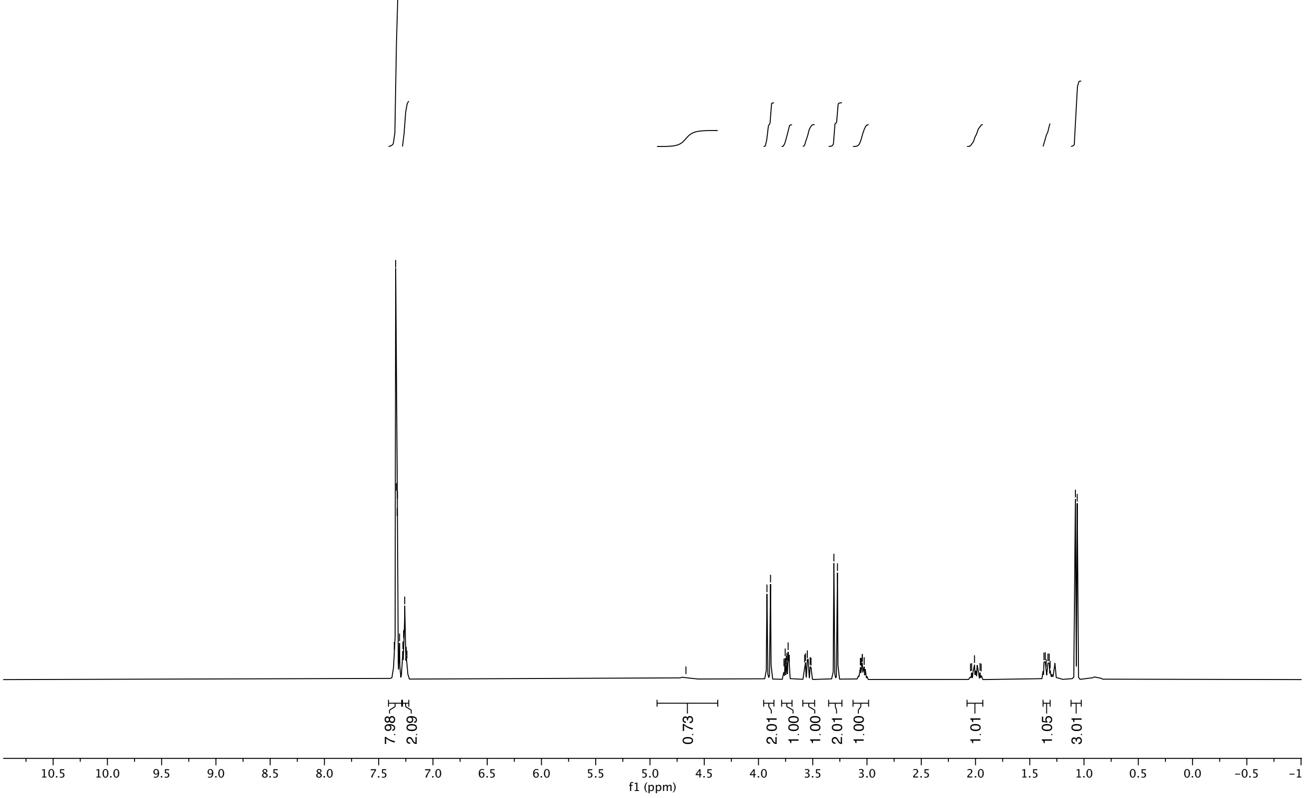
7.36 7.34 7.34 7.33 7.33 7.31 7.28 7.27 7.26 7.26 CDCl3 7.25 7.24
4.67 3.92 3.89 3.77 3.75 3.74 3.74 3.73 3.72 3.57 3.57 3.55 3.52 3.52 3.30 3.27 3.06 3.05 3.04 3.04 3.03 2.05 2.04 2.01 1.96 1.95 1.37 1.36 1.35 1.33 1.32 1.31 1.08 1.06
7.98 2.09 0.73 2.01 1.00 1.00 2.01 1.00 1.01 1.05 3.01
f1 (ppm)

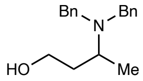
Bn
N
Bn
HO
Me

8j

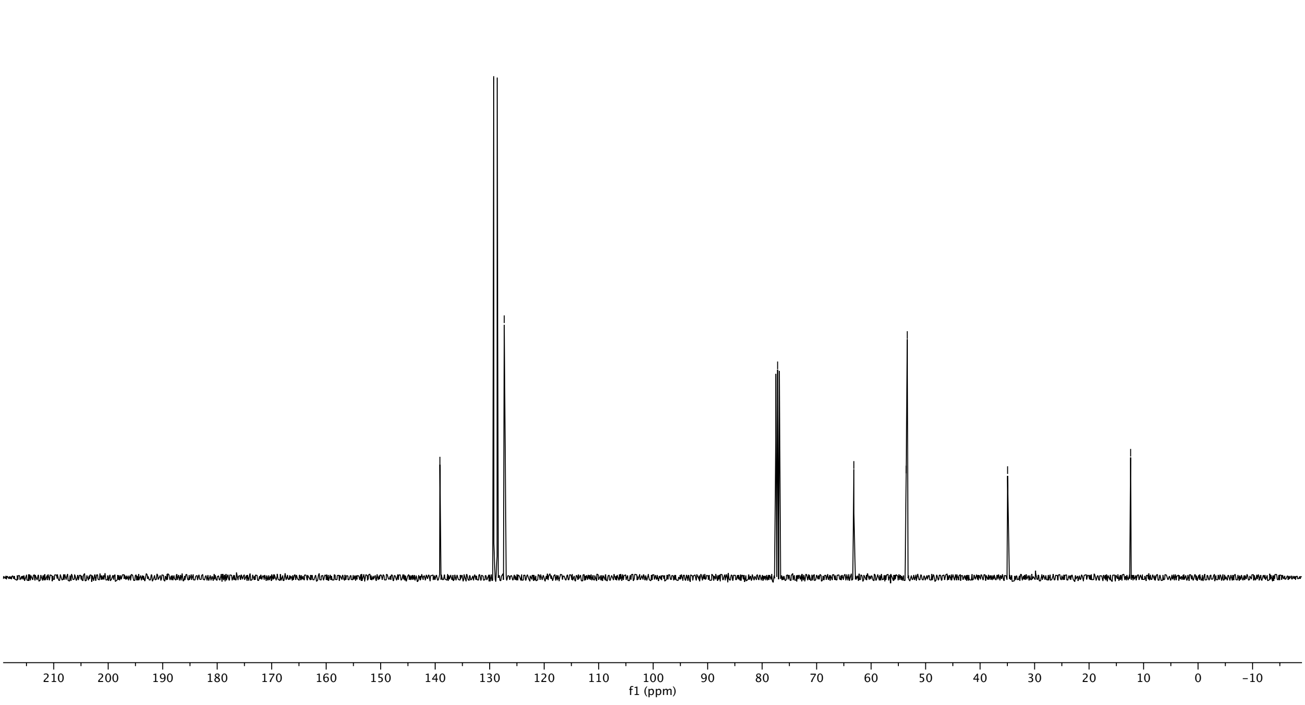
139.1
129.3 128.6 127.3
77.2 CDCl3
63.2
53.6 53.4
35.0
12.4
f1 (ppm)

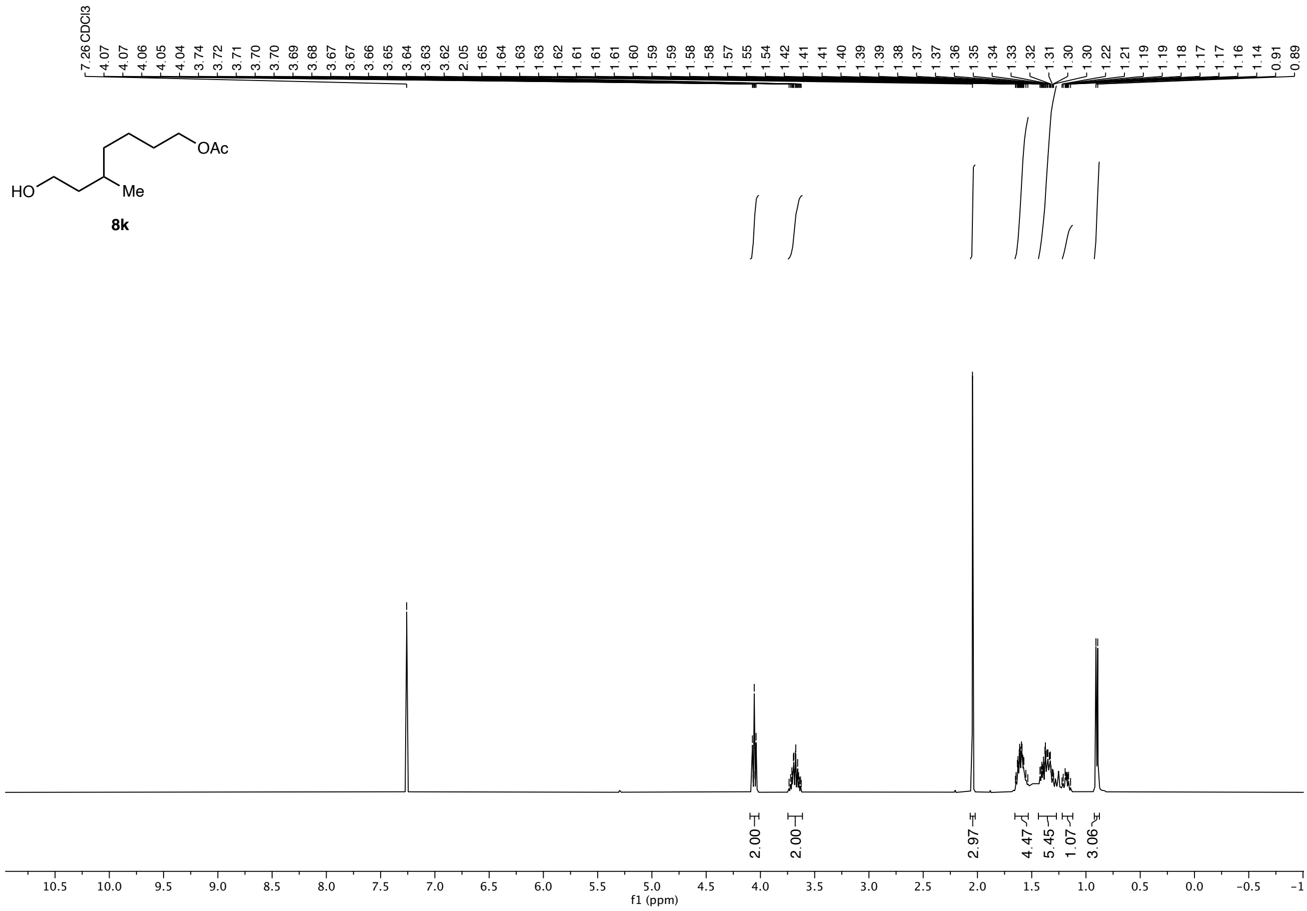
7.26 CDCl3
4.07
4.07
4.06
4.05
4.04
3.74
3.72
3.71
3.70
3.70
3.69
3.68
3.67
3.67
3.66
3.65
3.64
3.63
3.62
2.05
1.65
1.64
1.63
1.63
1.62
1.61
1.61
1.61
1.60
1.59
1.59
1.58
1.58
1.57
1.55
1.54
1.42
1.41
1.41
1.40
1.39
1.39
1.38
1.37
1.37
1.36
1.35
1.34
1.33
1.32
1.31
1.30
1.30
1.22
1.21
1.19
1.19
1.18
1.17
1.17
1.16
1.14
0.91
0.89
OAc
HO
Me
8k
2.00
2.00
2.97
4.47
5.45
1.07
3.06
f1 (ppm)

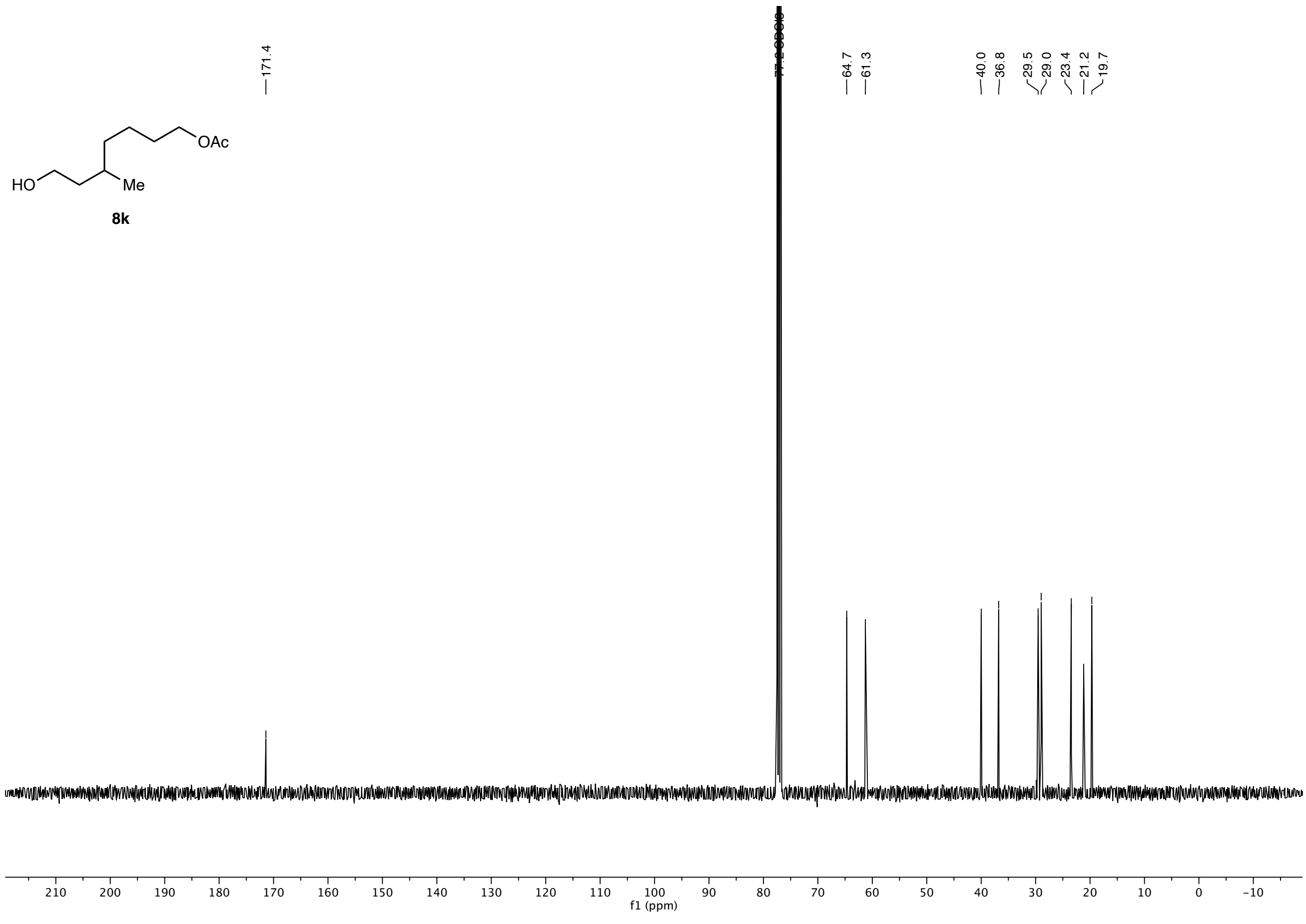
171.4
64.7
61.3
40.0
36.8
29.5
29.0
23.4
21.2
19.7
OAc
HO
Me
8k
f1 (ppm)
